# The gynecologic tumor risk related to GLP-1 receptor agonists and SGLT2 inhibitors use: a network meta-analysis of 91 randomized controlled trials

**DOI:** 10.1186/s13045-025-01750-x

**Published:** 2025-11-27

**Authors:** Ping-Tao Tseng, Bing-Yan Zeng, Chih-Wei Hsu, Cheuk-Kwan Sun, Mein-Woei Suen, Andre F. Carvalho, Brendon Stubbs, Yen-Wen Chen, Tien-Yu Chen, Wei-Te Lei, Po-Huang Chen, Jiann-Jy Chen, Yow-Ling Shiue, Bing-Syuan Zeng, Kuan-Pin Su, Chih-Sung Liang

**Affiliations:** 1 Institute of Precision Medicine, National Sun Yat-sen University, 70 Lienhai Rd, Kaohsiung City, 80424 Taiwan; 2 Institute of Biomedical Sciences, National Sun Yat-sen University, Kaohsiung, Taiwan; 3 Department of Psychology, College of Medical and Health Science, Asia University, Taichung, Taiwan; 4 Prospect Clinic for Otorhinolaryngology & Neurology, No. 252, Nanzixin Road, Nanzi District, Kaohsiung City, 81166 Taiwan; 5 Department of Internal Medicine, E-Da Dachang Hospital, I-Shou University, Kaohsiung, Taiwan; 6 Department of Psychiatry, Kaohsiung Chang Gung Memorial Hospital and Chang Gung University College of Medicine, Kaohsiung, Taiwan; 7 Department of Emergency Medicine, E-Da Dachang Hospital, I-Shou University, Kaohsiung, Taiwan; 8 School of Medicine for International Students, I-Shou University, Kaohsiung, Taiwan; 9 Gender Equality Education and Research Center, Asia University, Taichung, Taiwan; 10 Department of Medical Research, Asia University Hospital, Asia University, Taichung, Taiwan; 11 Department of Medical Research, China Medical University Hospital, China Medical University, Taichung, Taiwan; 12 Innovation in Mental and Physical Health and Clinical Treatment (IMPACT) Strategic Research Centre, School of Medicine, Barwon Health, Deakin University, Geelong, VIC Australia; 13 Department of Psychological Medicine, Institute of Psychiatry, Psychology and Neuroscience, King’s College London, London, UK; 14 Department of Sport, University of Vienna, Vienna, Austria; 15 Department of Psychiatry, Tri-Service General Hospital, Taipei, Taiwan; 16 Department of Psychiatry, College of Medicine, National Defense Medical University, Taipei, Taiwan; 17 Division of Pediatric Allergy, Immunology, and Rheumatology, Department of Pediatrics, Hsinchu Municipal MacKay Children’s Hospital, Hsinchu City, Taiwan; 18 Graduate Institute of Clinical Medical Sciences, College of Medicine, Chang Gung University, Taoyuan City, Taiwan; 19 Division of Hematology and Oncology, Department of Internal Medicine, Tri-Service General Hospital; School of Medicine, National Defense Medical University, Taipei, Taiwan; 20 Department of Internal Medicine, E-Da Cancer Hospital, I-Shou University, No.21, Yida Road, Jiaosu Village, Yanchao District, Kaohsiung, 82445 Taiwan; 21 Office of Research and Development, Asia University, Taichung, Taiwan; 22 College of Medicine, China Medical University, Taichung, Taiwan; 23 An-Nan Hospital, China Medical University, Tainan, Taiwan; 24 Department of Psychiatry, Beitou Branch, Tri-Service General Hospital; School of Medicine, National Defense Medical University, No. 60, Xinmin Road, Beitou District, Taipei City, 112003 Taiwan; 25 Department of Psychiatry, National Defense Medical University, Taipei, Taiwan

**Keywords:** Network meta-analysis, GLP-1 receptor agonist, SGLT2 inhibitor, Tirzepatide, Gynecologic, Uterine, Tumor, Malignant

## Abstract

**Background:**

Concerns have emerged regarding the oncogenic potential of glucagon-like peptide-1 (GLP-1) receptor agonists and sodium-glucose co-transporter 2 (SGLT2) inhibitors, particularly in relation to gynecologic malignancies. To date, no consensus has been reached on regimen-specific risks. This network meta-analysis (NMA) evaluated and compared the incidence of gynecologic tumors associated with various GLP-1 receptor agonists and SGLT2 inhibitors.

**Methods:**

Following a Cochrane-recommended confirmatory approach, we conducted a frequentist-based NMA using data from randomized controlled trials (RCTs) including female participants. Systematic searches of major databases were conducted through March 3, 2025. The primary outcome was the incidence of gynecologic tumors; drop-out rates served as an acceptability endpoint. Bayesian sensitivity analyses were conducted for validation.

**Results:**

This NMA included 91 RCTs comprising 224,986 participants (89,558 females). Only high-dose tirzepatide (15mg/week) was associated with significantly increased overall gynecologic tumor risk compared to controls [odds ratio (OR) = 2.37, 95% confidence interval (CI): 1.04–5.39; absolute risk difference= 0.57%; number needed to harm = 176]. Site-specific analysis noticed that both high-dose tirzepatide (15mg/week) (OR = 4.65, 95% CI: 1.28–16.94) and low-dose tirzepatide (5mg/week) (OR = 4.61, 95% CI: 1.07–19.90) were associated with a significantly elevated risk of intra-uterus tumor. No other regimen demonstrated a significant elevation in risk.

**Conclusions:**

Tirzepatide appears to be associated with gynecologic tumor risk, particularly intra-uterus neoplasms. Given that these agents are frequently prescribed to individuals with predisposing risk factors, personalized risk–benefit evaluation is warranted. However, more data are needed to confirm whether this association is causal and not due to chance/bias or not. Besides, longitudinal research is essential to elucidate underlying mechanisms and guide clinical decision-making.

**Trial registration: PROSPERO CRD420251003016:**

The study protocol was approved by the Institutional Review Board of the Tri-Service General Hospital, National Defense Medical Center (TSGHIRB E202516007).

**Supplementary Information:**

The online version contains supplementary material available at 10.1186/s13045-025-01750-x.

## Introduction

Glucagon-like peptide-1 (GLP-1) receptor agonists and sodium–glucose co-transporter 2 (SGLT2) inhibitors are modern antidiabetic agents that offer novel mechanisms of glycemic regulation, distinct from traditional therapies [1]. Nonetheless, recent investigations have raised safety concerns, with emerging evidence implicating a possible link between these agents and tumorigenesis [2, 3]. A growing number of case series, clinical investigations, and real-world data analyses have pointed to a potential association between their use and cancer development [4–6]. Laboratory-based research has proposed several plausible biological mechanisms for this effect. For instance, SGLT2 inhibitors may exert anti-apoptotic activity via AMPK/SIRT1 signaling [7, 8] or ERK1/2 activation through calcium-mediated pathways [9], both of which may follow a dose-response pattern [10, 11]. Similarly, GLP-1 receptor agonists have been shown to interfere with apoptotic regulation via BCL2/BAX/caspase-3 pathways [12] or influence gene expression through epigenetic modifications such as DNA demethylation of tumor suppressor loci [13].

Gynecologic tumors—which include malignancies of the uterus, cervix, endometrium, ovary, breast, vagina, and vulva—are prevalent among individuals who meet the indications for GLP-1 receptor agonists or SGLT2 inhibitors [14–16]. For example, in the meta-analysis by Friberg et al. [17], the authors summarized that diabetes was statistically significantly associated with an increased risk of endometrial cancer with a risk ratio of 2.10. Similarly, in another meta-analysis by Qin et al. [18], they noticed a positive association between obesity and the risk/prevalence of uterine tumors with an odds ratio (OR) of 1.19. Notably, several clinical trials have hinted at an increased incidence of gynecologic neoplasms among patients using these agents, although statistical significance was not consistently achieved [19–21]. In scenarios where trial data yield inconclusive findings due to design or power limitations, meta-analyses serve as critical tools to uncover patterns across heterogeneous datasets. By integrating results from large-scale studies and clinical databases, meta-analyses are regarded as robust indicators of real-world therapeutic outcomes and have frequently informed subsequent research priorities [22, 23].

However, existing meta-analyses in this field offer conflicting interpretations [24–27]. Many prior efforts employed conventional pairwise comparisons and were limited by several methodological constraints. For example, some pooled drugs of different classes or doses into broad categories, potentially obscuring drug-specific carcinogenic signals—especially those linked to agents with unique pharmacokinetic or pharmacodynamic profiles such as tirzepatide. Others stratified analyses by compound but failed to account for dose-dependent variability [24–26]. This limitation is notable given evidence from experimental models where glucose-dependent insulinotropic peptide (GIP) agonists, such as tirzepatide, have been shown to promote tumor proliferation in a dose-sensitive manner, particularly in colorectal cancer cell lines [28].

To address these gaps, we designed a comprehensive network meta-analysis (NMA) that stratifies GLP-1 receptor agonists and SGLT2 inhibitors by both agent and dosage. This approach enables a refined comparison of gynecologic tumor risks attributable to individual regimens. Our current NMA is conceptually aligned with our prior investigations on the neurodegenerative [29], metastatic cancer prevention [30], and auditory [31] risks associated with these agents. To our knowledge, no prior NMA has systematically evaluated site-specific gynecologic tumor risks across different agents and dosages. Therefore, this study aims to clarify the comparative risk profiles of these treatments in relation to gynecologic tumor development.

## Methods

This network meta-analysis (NMA) was performed using a confirmatory strategy based on Cochrane recommendations [32], targeting predefined adverse outcomes—specifically, the incidence of gynecologic tumors. In brief, we listed targeted adverse events as outcomes of interest (i.e. incidence of gynecologic tumors based on biological plausibility here) in our review protocol as our main interest. As notified in Cochrane manuals “*review authors should assume adverse events to be measured regularly and consistently in studies*” [32], we strictly focused on randomized controlled trials (RCTs) with systematically report of adverse events or explicitly evaluating target outcomes [33]. The reporting adhered to the Preferred Reporting Items for Systematic Reviews and Meta-Analyses guidelines, including the extension for network meta-analyses (PRISMA NMA) [34] (see eTable 1). This study protocol was registered with PROSPERO (registration number CRD420251003016) and received ethics approval from the Institutional Review Board of the Tri-Service General Hospital, National Defense Medical Center (TSGHIRB E202516007).

### Database search strategy and study selection

A systematic search was conducted across PubMed, Embase, ClinicalKey, Cochrane CENTRAL, ProQuest, ScienceDirect, Web of Science, and ClinicalTrials.gov to identify eligible trials published on or before March 3, 2025 (eTable 2). Two independent reviewers (PT Tseng and BY Zeng) independently screened the titles and abstracts. Any disagreements were resolved through discussion with a third reviewer (YW Chen). Additionally, reference lists of key meta-analyses and systematic reviews were examined to identify potentially overlooked trials [3, 24–27, 35–39]. No language restrictions were applied in the search.

#### Eligibility Criteria

The selection process was guided by the PICOS framework:

- **Population**: Subjects without pre-existing gynecologic malignancies (details below);
- **Intervention**: Use of GLP-1 receptor agonists or SGLT2 inhibitors across any dose range;
- **Comparator**: Placebo, usual care, active agents, or alternate doses;
- **Outcome**: Incidence of gynecologic tumors;
- **Study Design**: Only RCTs with systematically report of adverse events or explicitly evaluating target outcomes were eligible.

RCTs enrolling participants with known gynecologic tumors at baseline were excluded to focus on causality and preventive evaluation. To reduce bias from selective outcome reporting, only trials that either systematically recorded adverse events or explicitly evaluated target outcomes were included [33]. Inclusion criteria required: (1) RCTs excluding subjects with baseline gynecologic malignancies (definitions below), (2) trials investigating GLP-1 receptor agonists or SGLT2 inhibitors, (3) human subject involvement, and (4) structured safety data collection or direct outcome evaluation. Studies were excluded if they: (1) were not RCTs, (2) enrolled participants with established gynecologic tumors at baseline, (3) lacked interventions involving GLP-1 receptor agonists or SGLT2 inhibitors, (4) did not assess relevant outcomes, or (5) were preclinical/animal studies.

#### Risk of bias assessment

Two investigators independently evaluated trial quality using the Cochrane Risk of Bias Tool version 1.0 [40]. Disagreements were resolved through consultation with a third reviewer. The inter-rater reliability coefficient was 0.88.

#### Outcome measures

Recognizing that most included RCTs were not originally designed to track gynecologic cancer incidence, we limited our analysis to data involving female participants only. Outcomes from male participants were excluded to prevent dilution of event rates. As distinctions between benign and malignant tumors varied by region and time, both were grouped under “gynecologic tumors” for consistency. These included neoplasms of the intra-uterus, cervix, endometrium, ovary, breast, vagina, and vulva [14, 15]. Although breast cancer is sometimes classified under general oncology, we followed the National Cancer Institute’s designation of breast neoplasms as part of the gynecologic category [15]. Further, since intra-uterus consisted of uterine tumors and endometrial tumors [41], we arranged a subgroup analysis of uterine tumors and endometrial tumors. Acceptability was secondarily assessed via drop-out rates, encompassing discontinuation from any cause. We choose drop-out rates to represent acceptability based on our previous large scale NMAs [42–44].

Dosing classifications for each agent were adopted from their respective source trials [19–21, 45–128]:

- **Canagliflozin:** Low: 100 mg; High: 300mg.
- **Dapagliflozin:** Low: 2.5mg; Medium: 5 mg; High: 10mg.
- **Dulaglutide:** Low: < 1.5 mg; Medium: 1.5mg; High: >1.5mg.
- **Efpeglenatide:** Low: 2 mg; Medium: 4 mg; High: 6mg.
- **Empagliflozin:** Low: 1-10mg; High: 25-50mg.
- **Ertugliflozin:** Low: 5 mg; High: 15mg.
- **Injectable Semaglutide:** Low: 0.05-0.05.05.05.05.05.05.05.5mg; Medium: 1.0mg; High: 2.4mg.
- **Tirzepatide:** Low 5 mg; Medium: 10 mg; High: 15mg.

### Data extraction and management

Two reviewers (PT Tseng and BY Zeng) independently extracted study characteristics, including demographic variables, trial design, intervention protocols, outcome data, and adverse event reporting. When data were incomplete, attempts were made to contact the original investigators. Data handling adhered to the Cochrane Handbook and other established methodological guidelines [129].

#### Statistical analysis

We used a random-effects model for inter-study variability and multi-arm designs [130]. Analyses were conducted using MetaInsight version 4.0.2 (Complex Reviews Support Unit, NIHR, UK), which leverages the netmeta R package [131]. In cases where events occurred in only one arm, continuity corrections were applied; studies with zero events in both groups were omitted to minimize distortion [132, 133]. Effect sizes were presented as ORs with 95% confidence intervals (95%CIs), accompanied by forest plots [134]. We computed rankings and estimates for both direct and indirect comparisons. Consistency between direct and indirect effects was assessed using node-splitting techniques, which are especially useful in network structures with overlapping comparisons [131, 135]. A two-sided p-value < 0.05 was deemed statistically significant. Between-study heterogeneity was quantified using tau estimates, and inconsistency was assessed via loop-specific, node-splitting, and design-by-treatment interaction models [136]. Potential publication bias was evaluated through visual inspection of comparison-adjusted funnel plots and Egger’s tests.

#### Sensitivity analyses

To verify the robustness of findings, we conducted subgroup analyses focusing on individual gynecologic tumor types (uterine, cervical, endometrial, ovarian, breast, vaginal, and vulvar tumors). We also replicated all primary analyses using Bayesian-based NMA models to evaluate the convergence of treatment effects. Risk ranking was visualized via SUCRA plots, including radial and Rank-O-Gram formats [137]. Deviation from treatment assumptions was tested using deviation models [138]. The certainty of evidence was further graded using the GRADE framework [139].

### Ethical compliance

This investigation was conducted in accordance with the ethical standards outlined in the Declaration of Helsinki.

## Results

### Study eligibility and selection

**Fig. 1:**
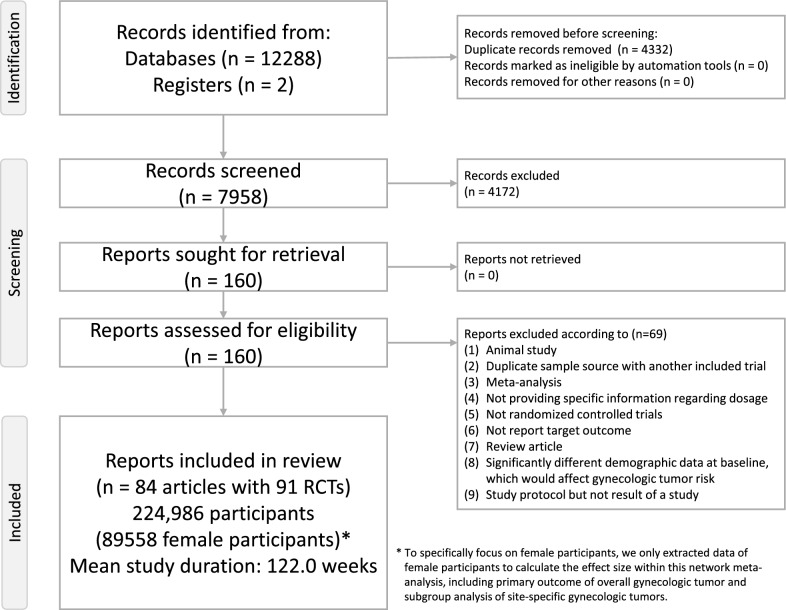
PRISMA2020 Flowchart of current network meta-analysis. Figure 1 depicted the whole flowchart of the current network meta-analysis * We only extracted female data to analyze outcome of gynecologic tumor

Figure 1 displays the study selection process. After excluding 69 studies that did not meet inclusion criteria (eTable 3), a total of 84 articles covering 91 randomized controlled trials (RCTs) were included for final analysis (eTable 4) [19–21, 45–128]. These trials enrolled a combined total of 224,986 participants, among whom 89,558 were female. The average age of enrolled subjects was 62.0 years (ranging from 41.2 to 71.9), and the mean study duration was 122.0 weeks (range: 24–281 weeks).

As anticipated, most RCTs were not originally powered or designed to detect gynecologic tumor incidence, and enrolled both male and female subjects. Therefore, we extracted female-specific data to compute all effect sizes, including the primary outcome (overall gynecologic tumors) and the secondary outcomes in subgroup analyses.

Altogether, 29 experimental conditions were evaluated, consisting of one control arm and 28 treatment groups using different GLP-1 receptor agonists or SGLT2 inhibitors across various dosage tiers. GLP-1 receptor agonists included tirzepatide, efpeglenatide, liraglutide, albiglutide, dulaglutide, exenatide, semaglutide, and lixisenatide. The SGLT2 inhibitors studied were bexagliflozin, canagliflozin, empagliflozin, ertugliflozin, dapagliflozin, and sotagliflozin.

### Primary outcome: Risk of overall gynecologic tumor

**Fig. 2:**
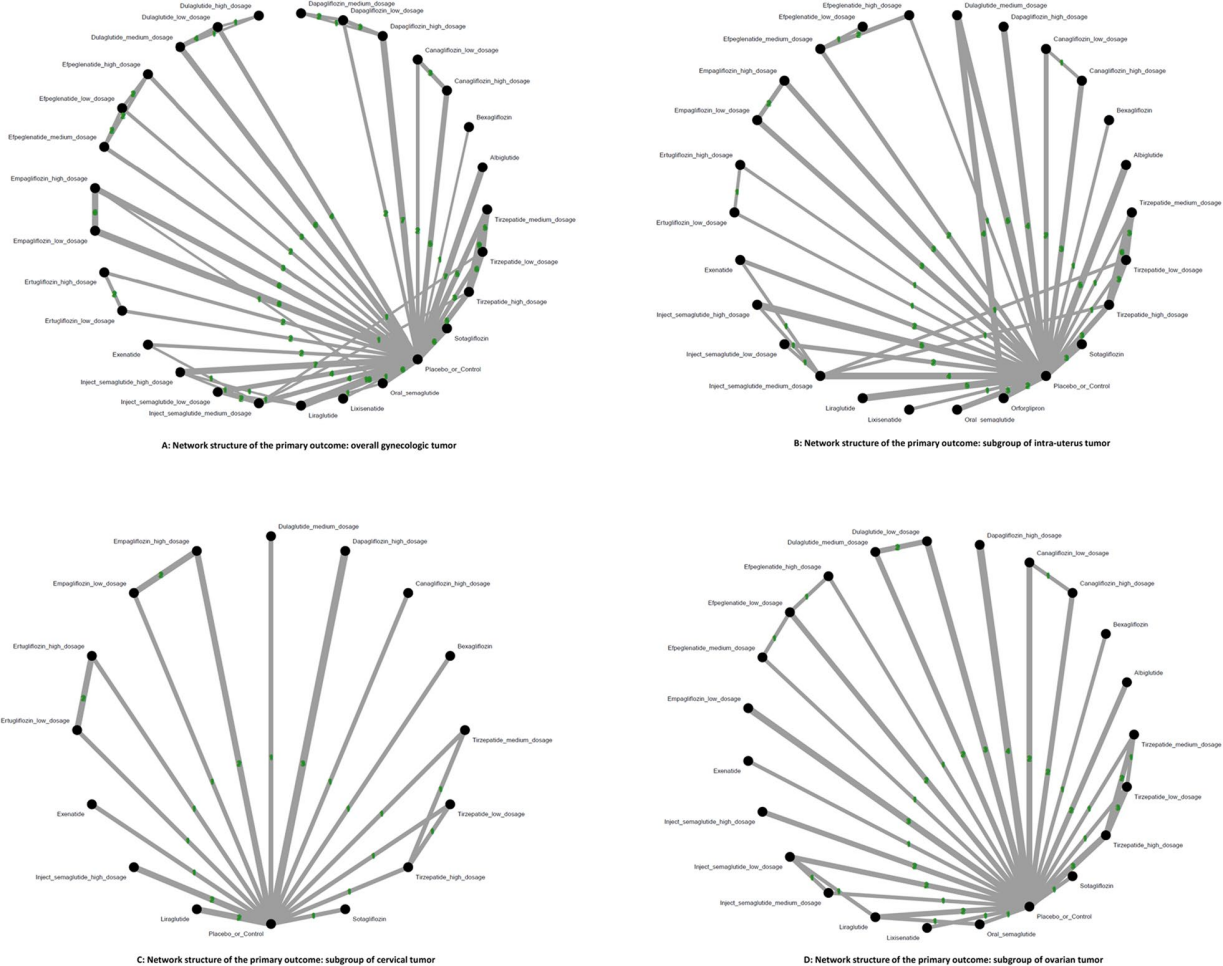

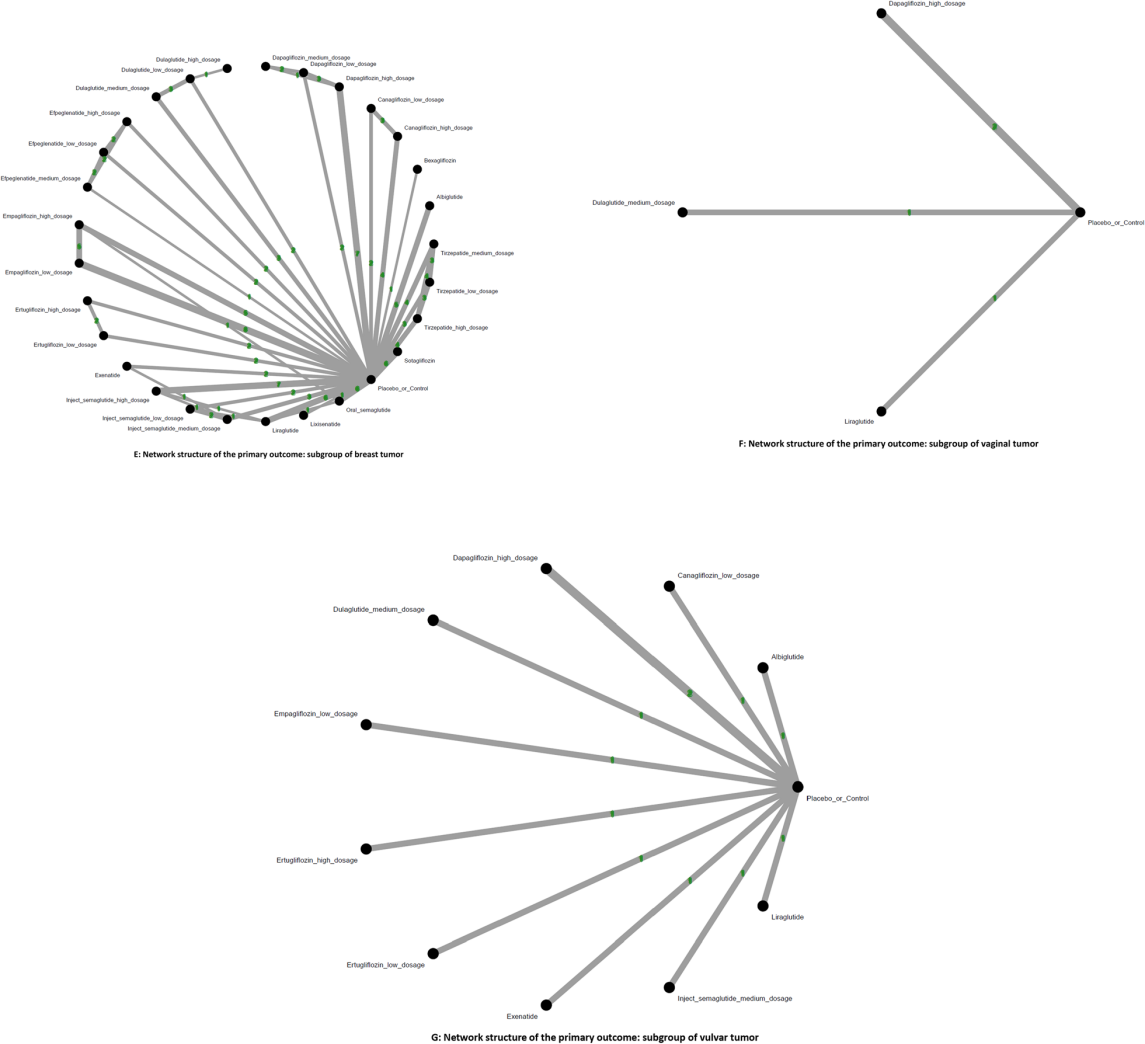
**A: **Network structure of the primary outcome: overall gynecologic tumor The overall structure of the network meta-analysis. The lines between nodes represent direct comparisons from various trials, with the numbers over the lines indicating the number of trials providing these comparisons for each specific treatment. The thickness of the lines corresponds to the number of trials linked to the network. **B: **Network structure of the primary outcome: subgroup of intra-uterus tumor The overall structure of the network meta-analysis. The lines between nodes represent direct comparisons from various trials, with the numbers over the lines indicating the number of trials providing these comparisons for each specific treatment. The thickness of the lines corresponds to the number of trials linked to the network. **C: **Network structure of the primary outcome: subgroup of cervical tumor The overall structure of the network meta-analysis. The lines between nodes represent direct comparisons from various trials, with the numbers over the lines indicating the number of trials providing these comparisons for each specific treatment. The thickness of the lines corresponds to the number of trials linked to the network. **D: **Network structure of the primary outcome: subgroup of ovarian tumor The overall structure of the network meta-analysis. The lines between nodes represent direct comparisons from various trials, with the numbers over the lines indicating the number of trials providing these comparisons for each specific treatment. The thickness of the lines corresponds to the number of trials linked to the network. **E: **Network structure of the primary outcome: subgroup of breast tumor The overall structure of the network meta-analysis. The lines between nodes represent direct comparisons from various trials, with the numbers over the lines indicating the number of trials providing these comparisons for each specific treatment. The thickness of the lines corresponds to the number of trials linked to the network. **F: **Network structure of the primary outcome: subgroup of vaginal tumor The overall structure of the network meta-analysis. The lines between nodes represent direct comparisons from various trials, with the numbers over the lines indicating the number of trials providing these comparisons for each specific treatment. The thickness of the lines corresponds to the number of trials linked to the network. **G: **Network structure of the primary outcome: subgroup of vulvar tumor The overall structure of the network meta-analysis. The lines between nodes represent direct comparisons from various trials, with the numbers over the lines indicating the number of trials providing these comparisons for each specific treatment. The thickness of the lines corresponds to the number of trials linked to the network

**Fig. 3:**
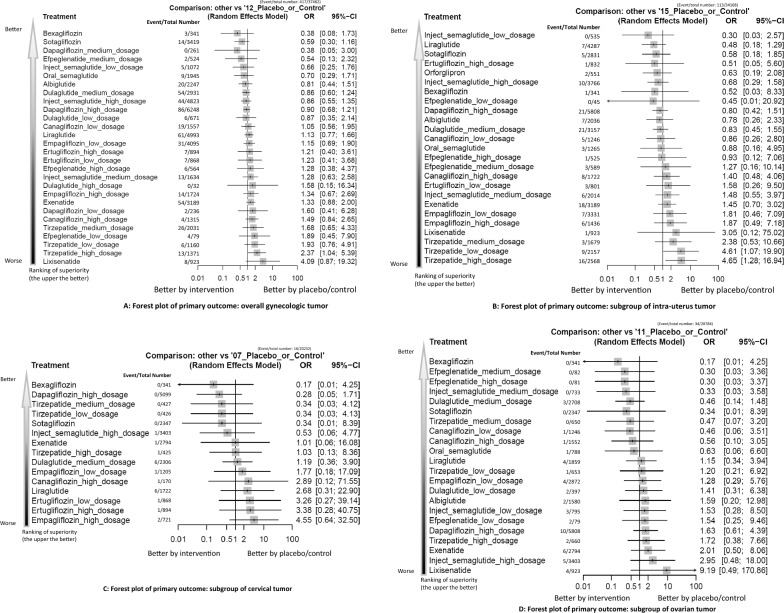

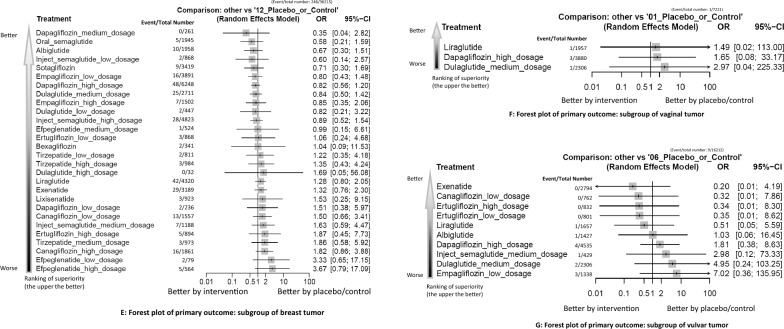
**A: **Forest plot of primary outcome: overall gynecologic tumorWhen the effect size (expressed as odds ratio) is less than 1, the specified treatment is associated with fewer events of target outcome compared to placebo/controls. B: Forest plot of primary outcome: subgroup of intra-uterus tumor When the effect size (expressed as odds ratio) is less than 1, the specified treatment is associated with fewer events of target outcome compared to placebo/controls. **C: **Forest plot of primary outcome: subgroup of cervical tumor When the effect size (expressed as odds ratio) is less than 1, the specified treatment is associated with fewer events of target outcome compared to placebo/controls. **D: **Forest plot of primary outcome: subgroup of ovarian tumor When the effect size (expressed as odds ratio) is less than 1, the specified treatment is associated with fewer events of target outcome compared to placebo/controls. **E: **Forest plot of primary outcome: subgroup of breast tumor When the effect size (expressed as odds ratio) is less than 1, the specified treatment is associated with fewer events of target outcome compared to placebo/controls. **F: **Forest plot of primary outcome: subgroup of vaginal tumor When the effect size (expressed as odds ratio) is less than 1, the specified treatment is associated with fewer events of target outcome compared to placebo/controls. **G: **Forest plot of primary outcome: subgroup of vulvar tumor When the effect size (expressed as odds ratio) is less than 1, the specified treatment is associated with fewer events of target outcome compared to placebo/controls

Our primary analysis revealed that only high-dose tirzepatide (15mg/week) demonstrated a statistically significant association with increased risk of overall gynecologic tumors, compared to controls [OR = 2.37, 95% CI: 1.04–5.39; absolute risk difference (ARD) = 0.57%; number needed to harm (NNH) = 176]. The average dosages of tirzepatide received before developing overall gynecologic tumors were 1067.0 mg (counted by “dosage x average treatment duration”). Among all treatments assessed, lixisenatide showed the highest point estimate of risk [OR = 4.09, 95% CI: 0.87–19.32], although it did not reach statistical significance (Figs. 2A, 3A, eFigure 3 A, Table 1).

**Table 1:**
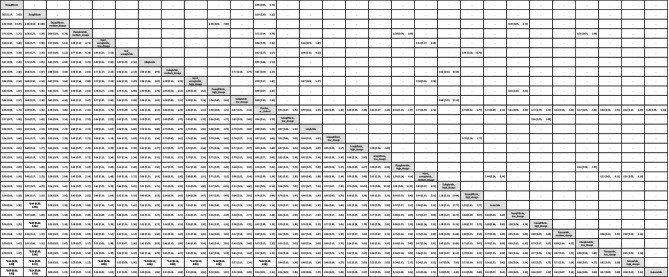
League table of the primary outcome: overall gynecologic tumor

Data presents as OR [95%CIs]. Pairwise (upper-right portion) and network (lower-left portion) meta-analysis results are presented as estimate effect sizes for the outcome of events of overall gynecologic tumor. Interventions are reported in order of mean ranking of beneficially prophylactic effect on events of overall gynecologic tumor, and outcomes are expressed as odds ratio (OR) (95% confidence intervals) (95%CIs). For the pairwise meta-analyses, OR of less than 1 indicates that the treatment specified in the row got more beneficial effect than that specified in the column. For the network meta-analysis (NMA), OR of less than 1 indicates that the treatment specified in the column got more beneficial effect than that specified in the row. Bold results marked with * indicate statistical significance

### Subgroup analyses: Site-specific gynecologic tumors

In subgroup evaluations focused on tumor sites, high-dose tirzepatide (15mg/week) and low-dose tirzepatide (5mg/week) were the only regimens associated with a significantly elevated risk—specifically for intra-uterus tumors [OR = 4.65, 95% CI: 1.28–16.94, OR = 4.61, 95% CI: 1.07–19.90.07.90, respectively]. The average dosages of tirzepatide received before developing uterine tumors were 1206.3 mg (counted by “dosage x average treatment duration”) (Figs. 2B, 3B, eFig. 3B, and Table 2).

**Table 2:**
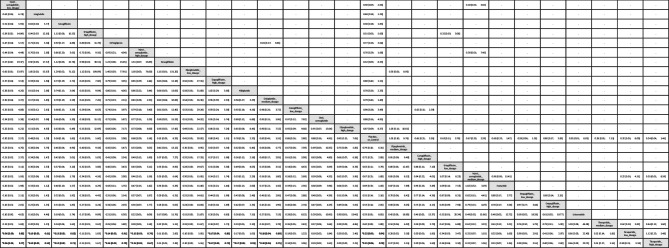
League table of the primary outcome: subgroup of intra-uterus tumor

Data presents as OR [95%CIs]. Pairwise (upper-right portion) and network (lower-left portion) meta-analysis results are presented as estimate effect sizes for the outcome of events of intra-uterus tumor. Interventions are reported in order of mean ranking of beneficially prophylactic effect on events of intra-uterus tumor, and outcomes are expressed as odds ratio (OR) (95% confidence intervals) (95%CIs). For the pairwise meta-analyses, OR of less than 1 indicates that the treatment specified in the row got more beneficial effect than that specified in the column. For the network meta-analysis (NMA), OR of less than 1 indicates that the treatment specified in the column got more beneficial effect than that specified in the row. Bold results marked with * indicate statistical significance

For all other gynecologic tumor subtypes—including cervical (Figs. 2C, 3C), ovarian (Figs. 2D, 3D), breast (Figs. 2E, 3E), vaginal (Figs. 2F, 3F), and vulvar tumors (Figs. 2G, 3G)—none of the investigated medications showed statistically significant differences compared to the control arms (see corresponding eFigures 3C–3G and Tables 3, 4, 5, 6, 7). Finally, in the subgroup analysis of uterine tumor and endometrial tumor, we noticed that only high-dose tirzepatide (15mg/week) was associated with a significantly elevated risk—specifically for uterine tumors [OR = 5.65, 95% CI: 1.21–26.50] but not endometrial tumor (eFigure 1A-1B, eFigure 2A-2B, and eTable 5A-5B).

**Table 3:**
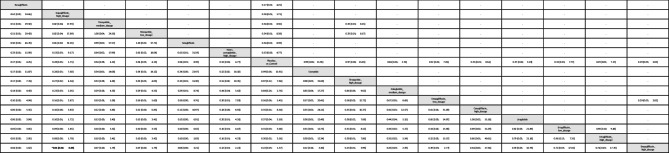
League table of the primary outcome: subgroup of cervical tumor

Data presents as OR [95%CIs]. Pairwise (upper-right portion) and network (lower-left portion) meta-analysis results are presented as estimate effect sizes for the outcome of events of cervical tumor. Interventions are reported in order of mean ranking of beneficially prophylactic effect on events of cervical tumor, and outcomes are expressed as odds ratio (OR) (95% confidence intervals) (95%CIs). For the pairwise meta-analyses, OR of less than 1 indicates that the treatment specified in the row got more beneficial effect than that specified in the column. For the network meta-analysis (NMA), OR of less than 1 indicates that the treatment specified in the column got more beneficial effect than that specified in the row. Bold results marked with * indicate statistical significance

**Table 4:**
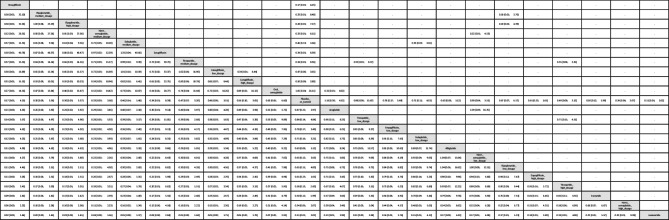
League table of the primary outcome: subgroup of ovarian tumor

Data presents as OR [95%CIs]. Pairwise (upper-right portion) and network (lower-left portion) meta-analysis results are presented as estimate effect sizes for the outcome of events of ovarian tumor. Interventions are reported in order of mean ranking of beneficially prophylactic effect on events of ovarian tumor, and outcomes are expressed as odds ratio (OR) (95% confidence intervals) (95%CIs). For the pairwise meta-analyses, OR of less than 1 indicates that the treatment specified in the row got more beneficial effect than that specified in the column. For the network meta-analysis (NMA), OR of less than 1 indicates that the treatment specified in the column got more beneficial effect than that specified in the row

**Table 5:**
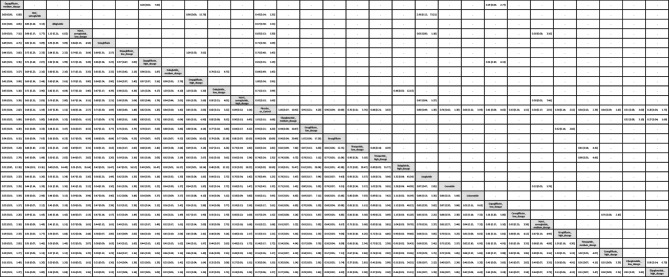
League table of the primary outcome: subgroup of breast tumor

Data presents as OR [95%CIs]. Pairwise (upper-right portion) and network (lower-left portion) meta-analysis results are presented as estimate effect sizes for the outcome of events of breast tumor. Interventions are reported in order of mean ranking of beneficially prophylactic effect on events of breast tumor, and outcomes are expressed as odds ratio (OR) (95% confidence intervals) (95%CIs). For the pairwise meta-analyses, OR of less than 1 indicates that the treatment specified in the row got more beneficial effect than that specified in the column. For the network meta-analysis (NMA), OR of less than 1 indicates that the treatment specified in the column got more beneficial effect than that specified in the row. Bold results marked with * indicate statistical significance

**Table 6:**
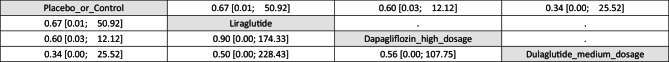
League table of the primary outcome: subgroup of vaginal tumor

Data presents as OR [95%CIs]. Pairwise (upper-right portion) and network (lower-left portion) meta-analysis results are presented as estimate effect sizes for the outcome of events of vaginal tumor. Interventions are reported in order of mean ranking of beneficially prophylactic effect on events of vaginal tumor, and outcomes are expressed as odds ratio (OR) (95% confidence intervals) (95%CIs). For the pairwise meta-analyses, OR of less than 1 indicates that the treatment specified in the row got more beneficial effect than that specified in the column. For the network meta-analysis (NMA), OR of less than 1 indicates that the treatment specified in the column got more beneficial effect than that specified in the row. Bold results marked with * indicate statistical significance

**Table 7:**
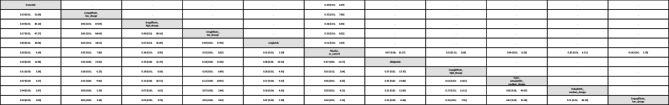
League table of the primary outcome: subgroup of vulvar tumor

Data presents as OR [95%CIs]. Pairwise (upper-right portion) and network (lower-left portion) meta-analysis results are presented as estimate effect sizes for the outcome of events of vulvar tumor. Interventions are reported in order of mean ranking of beneficially prophylactic effect on events of vulvar tumor, and outcomes are expressed as odds ratio (OR) (95% confidence intervals) (95%CIs). For the pairwise meta-analyses, OR of less than 1 indicates that the treatment specified in the row got more beneficial effect than that specified in the column. For the network meta-analysis (NMA), OR of less than 1 indicates that the treatment specified in the column got more beneficial effect than that specified in the row

### Acceptability analysis: Drop-out rates

Drop-out rates, used here as a proxy for overall acceptability, were assessed based on available data from the original trial populations. Unfortunately, subgroup drop-out information specific to female participants was not reported in the included trials. Thus, our drop-out analyses reflect overall trial-level data.

Several treatments demonstrated significantly lower drop-out rates than controls. These included:

- Low-dose tirzepatide (5mg/week) [OR = 0.64, 95% CI: 0.49–0.83]
- High-dose canagliflozin (300mg/day) [OR = 0.66, 95% CI: 0.52–0.84]
- Low-dose canagliflozin (100mg/day) [OR = 0.68, 95% CI: 0.52–0.89]
- High-dose tirzepatide (15mg/week) [OR = 0.72, 95% CI: 0.57–0.92]
- High-dose injectable semaglutide (2.4mg/week) [OR = 0.73, 95% CI: 0.58–0.91]
- Medium-dose tirzepatide (10mg/week) [OR = 0.73, 95% CI: 0.58–0.93]
- Medium-dose injectable semaglutide (1.0mg/week) [OR = 0.75, 95% CI: 0.57–0.97]
- Low-dose empagliflozin (1–10mg/day) [OR = 0.82, 95% CI: 0.70–0.97]

Among all evaluated options, low-dose tirzepatide had the most favorable drop-out profile (eFigure 1 C, eFigure 2 C, eFigure 3G, and eTable 5 C).

### Sensitivity analyses: Bayesian NMA

The results of the primary frequentist NMA were consistent with those obtained through Bayesian-based sensitivity models (eFigure 4A–4H). Risk ranking using SUCRA values also supported the primary findings (eTable 6A–6H, eFigure 5A–5P). Assessment of network coherence via deviation models indicated no significant model inconsistencies (eFigure 6A–6X).

### Risk of bias and certainty of evidence

No substantial publication bias was detected through visual inspection of the comparison-adjusted funnel plots or by Egger’s regression analysis (eFigure 7A–7B). According to our risk of bias evaluation using Cochrane criteria, 78.6% of the assessed items (501 out of 637) were rated as low risk, 15.9% (101/637) as unclear, and 5.5% (35/637) as high risk (eFigures 8A–8B). Inconsistency testing, including loop-specific analysis, node-splitting, and design-by-treatment interaction models, did not reveal major violations of consistency assumptions (eTable 7A–7I). Measures of between-study heterogeneity showed acceptable levels across most comparisons (eTable 8). Using GRADE criteria, the overall certainty of evidence was deemed moderate to high across outcomes (eTable 9A–9H).

## Discussion

To our knowledge, this is the first network meta-analysis (NMA) to comprehensively evaluate gynecologic tumor risk from GLP-1 receptor agonists and SGLT2 inhibitors, stratified by both agent and dosage, with exclusive focus on female participants. Our findings show that tirzepatide at 15mg/week—a dosage approved in clinical practice [140] —was significantly associated with increased risk of both overall gynecologic tumors and intra-uterus tumor. Besides, the average cumulated dosage tirzepatide received before developing overall gynecologic tumors or intra-uterus tumor was 1067.0 mg and 1206.3 mg, respectively, which meant that subjects receiving tirzepatide at 15mg/week would need 71.1 weeks and 80.4 weeks to achieve it. These results support the hypothesis that carcinogenic potential may depend not only on drug class but also on specific regimen and dosages.

The most clinically significant observation in this study is that only tirzepatide, and only at its highest dose (15mg/week), was associated with a significant elevation in gynecologic tumor risk, specifically intra-uterus tumor, which differed from the results in the recent retrospective cohort study by Dai et al. [141]. In that report by Dai et al., the authors pooled various GLP-1 receptor agonists with different pharmacology properties into one group and found that GLP-1 receptor agonists were associated with a reduced risk of endometrial cancer and ovarian cancer [141]. This approach of pooling various medications with different pharmacologic properties without accounting for differences in pharmacologic characteristics or dosages (i.e. grouping pure GLP-1 agonists with GLP-1/GIP agonists) risks inconclusive results due to underlying heterogeneity within the comparison group. Specifically, in the report by Dai et al., although the authors demonstrated reduced risks of endometrial cancer (hazard ratio [HR], 0.75 [95% CI, 0.57–0.99.57.99]; *p* = 0.05) and ovarian cancer (HR, 0.53 [95% CI, 0.29–0.96.29.96]; *p* = 0.04) by GLP-1 receptor agonist prescription, the wide confidence interval and borderline *p* value might diminish the confidence of their findings [141]. For example, Dicembrini et al. found no significant association between SGLT2 inhibitors and breast cancer [24], while subsequent meta-analyses by Spiazzi et al. and Xu et al. extended the analysis to other tumor types (endometrial and uterine) and still observed no significant risks [25, 26]. On the GLP-1 side, Piccoli et al. similarly reported no significant risk of breast cancer across agents [27]. Silverii et al. demonstrated that overall GLP-1 receptor agonists might reduce uterine cancer risk in obese patients [142]. Although a few studies stratified results by compound [24–26], most did not incorporate dose-specific effects—potentially overlooking subtle but meaningful differences in safety. Finally, Kamrul-Hasan et al. demonstrated the insignificant association between different dosages of tirzepatide and risks of breast cancers, ovarian cancers, and uterine cancers [143]. However, the heterogeneity in the control arms might limit the statistical significance in the meta-analysis by Kamrul-Hasan [143]. This is important because drug safety profiles may be highly dose-sensitive, with certain adverse effects manifesting only at higher thresholds [144]. Indeed, Prabakaran et al. found that GLP-1 receptor agonists promoted cell proliferation in vitro in a dose-dependent fashion [28].

Our study advances this field by leveraging NMA methodology to dissect compound- and dose-specific risks. The association we found between high-dose tirzepatide and gynecologic tumors implies that not all GLP-1 receptor agonists are equally safe in this context, and that dose escalation may shift the risk–benefit balance unfavorably. These insights could aid clinicians in selecting safer regimens, particularly for patients with underlying risk factors for gynecologic malignancy.

Tirzepatide represents a unique agent within this therapeutic class, often referred to as a "twin-cretin" because of its dual activity as both GLP-1 and glucose-dependent insulinotropic polypeptide (GIP) receptor agonist [145]. While this dual mechanism enhances glycemic and weight-loss benefits, GIP signaling may also pose oncologic concerns. Prior studies have indicated that GIP receptor activation can stimulate growth in various tumor types, particularly solid tumors [146]. In a preclinical model, Prabakaran et al. demonstrated that GIP agonism enhanced colorectal cancer cell proliferation in a dose-responsive manner [28]. This raises legitimate concerns regarding the carcinogenic potential of high-dose tirzepatide [147].

In particular, tirzepatide may increase the synthesis of insulin, IGF-1, IGF-2, and their binding proteins—factors that have been linked to elevated cancer risk, especially in patients with prior pancreatic injury [147]. Moreover, in genetically predisposed populations, such as those with a family history of medullary thyroid carcinoma or multiple endocrine neoplasia type 2, tirzepatide might further elevate thyroid cancer risk through GIP-mediated pathways [148]. Interestingly, uterine tissues appear to express minimal GIP receptor activity [149], suggesting that alternate mechanisms may drive the observed association with uterine tumors. One hypothesis is that tirzepatide may act synergistically with circulating glucose or insulin levels to activate growth factor–like pathways and stimulate cell proliferation through pleiotropic mechanisms [150]. Besides, the diverse result of tirzepatide in uterine tumor and endometrial tumor might reflect the fact that tirzepatide would exert diverse effects on uterine and endometrial cells. Nevertheless, there is currently no direct clinical or mechanistic study linking tirzepatide to uterine tumorigenesis. Therefore, prospective clinical and translational studies are needed to further investigate this association.

## Strengths and limitations

This study possesses several methodological strengths. First, by utilizing an NMA framework, we were able to compare multiple agents simultaneously while maintaining internal consistency across studies. Second, the strict inclusion of randomized controlled trials (RCTs) enhanced the validity of our findings by minimizing confounding. Third, our approach excluded trials involving patients with baseline gynecologic tumors, allowing us to focus on potential causality rather than recurrence or progression. Fourth, we conducted detailed subgroup analyses by tumor site, enabling more precise interpretation of risk across gynecologic tumor subtypes. Finally, robustness was confirmed through Bayesian-based sensitivity analyses, which corroborated our primary findings.

Nonetheless, certain limitations should be acknowledged. The average trial duration in our dataset was 122.0 weeks. While this may seem adequate, tumor development—particularly for solid tumors—often requires longer latency periods [151]. Therefore, longer-term follow-up studies would be valuable to confirm our observations. Second, although our RCT-focused design enhanced internal validity, it inherently excluded observational data that might offer insights into late-onset events. However, RCTs are less prone to confounding and reporting bias. Third, variation in diagnostic criteria across countries and trials may have introduced heterogeneity. Unlike standardized tumor surveillance protocols, adverse event reporting is typically less rigorous, which may lead to underreporting or misclassification of tumor events. Specifically, many of the included RCTs were designed to assess cardiometabolic endpoints—not tumor risk—so relevant variables such as tumor family history or hormonal status were not adjusted for in the original designs. To overcome these inherent limitations (diagnostic criteria variation and potentially missing report of target adverse events), we adopted confirmatory approach based on Cochrane recommendations [32] and strictly focused on RCTs with systematically report of adverse events or explicitly evaluating target outcomes [33]. These adaptation strategies had been widely used in various high impact articles, which discovered new insight regarding medication adverse events from RCTs not designed for target adverse events [152, 153]. While this may affect effect size precision, it does not negate the utility of our findings as hypothesis-generating evidence. Moreover, our use of a Cochrane-endorsed confirmatory strategy strengthens the interpretability of tumor signals derived from adverse event data.

## Conclusion

This NMA highlights a potential association between tirzepatide and gynecologic tumor risk, especially intra-uterus tumors, at the commonly used 15mg/week dose. While most GLP-1 receptor agonists and SGLT2 inhibitors appear oncologically safe, our results underscore that regimen-specific risks must not be overlooked. Given that many candidates for these agents already have predisposing risk factors for cancer, treatment decisions should be individualized. Besides, clinicians should carefully monitor the possibility of gynecologic tumors, such as intra-uterus tumors, when their patients receiving 15mg/week tirzepatide for about 71.1 to 80.4 weeks (about 1.3 years). However, more data are needed to confirm whether this association is causal and not due to chance/bias or not. Therefore, our findings call for further long-term trials and mechanistic studies to clarify the oncogenic potential of tirzepatide and to guide safer clinical use.

## Supplementary Information

Additional file 1

Supplementary Material 2

## Data Availability

All the data of the current study were available at reasonable request to the corresponding authors

## Abbreviations

95%CIs: 95% Confidence intervals
GLP-1 agonist: Glucagon-like peptide-1 agonist
NMA: Network meta-analysis
OR: Odds ratio
RCT: Randomized controlled trial
SGLT2 inhibitor: Sodium–glucose cotransporter 2 inhibitor
