# Supplementary material for: The gynecologic tumor risk related to GLP-1 receptor agonists and SGLT2 inhibitors use: a network meta-analysis of 91 randomized controlled trials
Source: J Hematol Oncol. 2025 Nov 27;18:109. doi: 10.1186/s13045-025-01750-x (PMC12661836; doi:10.1186/s13045-025-01750-x)
Supplement: Supplementary file 1 — Additional file 1 [file 13045_2025_1750_MOESM1_ESM.docx]

**List of content in supplement materials**

**The Gynecologic Tumor Risk Related to GLP-1 Receptor Agonists and SGLT2 Inhibitors Use**

**A Network Meta-Analysis of 91 Randomized Controlled Trials**

*Ping-Tao Tseng, et al.*

| eFigure 1 | (A) Network structure of NMA of the primary outcome: subgroup of uterine tumor |
| --- | --- |
|  | (B) Network structure of NMA of the primary outcome: subgroup of endometrial tumor |
|  | (C) Network structure of NMA of acceptability: drop-out rate |
| eFigure 2 | (A) Forest plot of NMA of the primary outcome: subgroup of uterine tumor |
|  | (B) Forest plot of NMA of the primary outcome: subgroup of endometrial tumor |
|  | (C) Forest plot of NMA of acceptability: drop-out rate |
| eFigure 3 | (A) Individual study result of primary outcome: overall gynecologic tumor |
|  | (B) Individual study result of primary outcome: subgroup of intra-uterus tumor |
|  | (C) Individual study result of primary outcome: subgroup of cervical tumor |
|  | (D) Individual study result of primary outcome: subgroup of ovarian tumor |
|  | (E) Individual study result of primary outcome: subgroup of breast tumor |
|  | (F) Individual study result of primary outcome: subgroup of vaginal tumor |
|  | (G) Individual study result of primary outcome: subgroup of vulvar tumor |
|  | (H) Individual study result of acceptability: drop-out rate |
| eFigure 4 | (A) Bayesian-based forest plot of NMA of primary outcome: overall gynecologic tumor |
|  | (B) Bayesian-based forest plot of NMA of primary outcome: subgroup of intra-uterus tumor |
|  | (C) Bayesian-based forest plot of NMA of primary outcome: subgroup of cervical tumor |
|  | (D) Bayesian-based forest plot of NMA of primary outcome: subgroup of ovarian tumor |
|  | (E) Bayesian-based forest plot of NMA of primary outcome: subgroup of breast tumor |
|  | (F) Bayesian-based forest plot of NMA of primary outcome: subgroup of vaginal tumor |
|  | (G) Bayesian-based forest plot of NMA of primary outcome: subgroup of vulvar tumor |
|  | (H) Bayesian-based forest plot of NMA of acceptability: drop-out rate |
| eFigure 5 | (A) Bayesian-based Litmus Rank-O-Gram rank plot of primary outcome: overall gynecologic tumor |
|  | (B) Bayesian-based radial surface under the cumulative ranking of primary outcome: overall gynecologic tumor |
|  | (C) Bayesian-based Litmus Rank-O-Gram rank plot of primary outcome: subgroup of intra-uterus tumor |
|  | (D) Bayesian-based radial surface under the cumulative ranking of primary outcome: subgroup of intra-uterus tumor |
|  | (E) Bayesian-based Litmus Rank-O-Gram rank plot of primary outcome: subgroup of cervical tumor |
|  | (F) Bayesian-based radial surface under the cumulative ranking of primary outcome: subgroup of cervical tumor |
|  | (G) Bayesian-based Litmus Rank-O-Gram rank plot of primary outcome: subgroup of ovarian tumor |
|  | (H) Bayesian-based radial surface under the cumulative ranking of primary outcome: subgroup of ovarian tumor |
|  | (I) Bayesian-based Litmus Rank-O-Gram rank plot of primary outcome: subgroup of breast tumor |
|  | (J) Bayesian-based radial surface under the cumulative ranking of primary outcome: subgroup of breast tumor |
|  | (K) Bayesian-based Litmus Rank-O-Gram rank plot of primary outcome: subgroup of vaginal tumor |
|  | (L) Bayesian-based radial surface under the cumulative ranking of primary outcome: subgroup of vaginal tumor |
|  | (M) Bayesian-based Litmus Rank-O-Gram rank plot of primary outcome: subgroup of vulvar tumor |
|  | (N) Bayesian-based radial surface under the cumulative ranking of primary outcome: subgroup of vulvar tumor |
|  | (O) Bayesian-based Litmus Rank-O-Gram rank plot of acceptability: drop-out rate |
|  | (P) Bayesian-based radial surface under the cumulative ranking of acceptability: drop-out rate |
| eFigure 6 | (A) Bayesian-based residual deviance NMA/UME model of primary outcome: overall gynecologic tumor |
|  | (B) Bayesian-based per-arm residual deviance of primary outcome: overall gynecologic tumor |
|  | (C) Bayesian-based leverage plot of primary outcome: overall gynecologic tumor |
|  | (D) Bayesian-based residual deviance NMA/UME model of primary outcome: subgroup of intra-uterus tumor |
|  | (E) Bayesian-based per-arm residual deviance of primary outcome: subgroup of intra-uterus tumor |
|  | (F) Bayesian-based leverage plot of primary outcome: subgroup of intra-uterus tumor |
|  | (G) Bayesian-based residual deviance NMA/UME model of primary outcome: subgroup of cervical tumor |
|  | (H) Bayesian-based per-arm residual deviance of primary outcome: subgroup of cervical tumor |
|  | (I) Bayesian-based leverage plot of primary outcome: subgroup of cervical tumor |
|  | (J) Bayesian-based residual deviance NMA/UME model of primary outcome: subgroup of ovarian tumor |
|  | (K) Bayesian-based per-arm residual deviance of primary outcome: subgroup of ovarian tumor |
|  | (L) Bayesian-based leverage plot of primary outcome: subgroup of ovarian tumor |
|  | (M) Bayesian-based residual deviance NMA/UME model of primary outcome: subgroup of breast tumor |
|  | (N) Bayesian-based per-arm residual deviance of primary outcome: subgroup of breast tumor |
|  | (O) Bayesian-based leverage plot of primary outcome: subgroup of breast tumor |
|  | (P) Bayesian-based residual deviance NMA/UME model of primary outcome: subgroup of vaginal tumor |
|  | (Q) Bayesian-based per-arm residual deviance of primary outcome: subgroup of vaginal tumor |
|  | (R) Bayesian-based leverage plot of primary outcome: subgroup of vaginal tumor |
|  | (S) Bayesian-based residual deviance NMA/UME model of primary outcome: subgroup of vulvar tumor |
|  | (T) Bayesian-based per-arm residual deviance of primary outcome: subgroup of vulvar tumor |
|  | (U) Bayesian-based leverage plot of primary outcome: subgroup of vulvar tumor |
|  | (V) Bayesian-based residual deviance NMA/UME model of acceptability: drop-out rate |
|  | (W) Bayesian-based per-arm residual deviance of acceptability: drop-out rate |
|  | (X) Bayesian-based leverage plot of acceptability: drop-out rate |
| eFigure 7 | (A) Funnel plot of current network meta-analysis of overall gynecologic tumor |
|  | (B) Egger test of current network meta-analysis of overall gynecologic tumor |
| eFigure 8 | (A) Overview of risk of bias |
|  | (B) Detailed risk of bias in each study |
| eTable 1 | PRISMA 2020 checklist of the current network meta-analysis |
| eTable 2 | Keyword used in each database and search results |
| eTable 3 | Excluded studies and reason |
| eTable 4 | Characteristics of the included studies |
| eTable 5 | (A) League table of NMA of the primary outcome: subgroup of uterine tumor |
|  | (B) League table of NMA of the primary outcome: subgroup of endometrial tumor |
|  | (C) League table of NMA of acceptability: drop-out rate |
| eTable 6 | (A) SUCRA (Surface under the cumulative ranking) of primary outcome: overall gynecologic tumor |
|  | (B) SUCRA (Surface under the cumulative ranking) of primary outcome: subgroup of intra-uterus tumor |
|  | (C) SUCRA (Surface under the cumulative ranking) of primary outcome: subgroup of cervical tumor |
|  | (D) SUCRA (Surface under the cumulative ranking) of primary outcome: subgroup of ovarian tumor |
|  | (E) SUCRA (Surface under the cumulative ranking) of primary outcome: subgroup of breast tumor |
|  | (F) SUCRA (Surface under the cumulative ranking) of primary outcome: subgroup of vaginal tumor |
|  | (G) SUCRA (Surface under the cumulative ranking) of primary outcome: subgroup of vulvar tumor |
|  | (H) SUCRA (Surface under the cumulative ranking) of acceptability: drop-out rate |
| eTable 7 | (A) Side-splitting model inconsistency of primary outcome: overall gynecologic tumor |
|  | (B) Side-splitting model inconsistency of primary outcome: subgroup of intra-uterus tumor |
|  | (C) Side-splitting model inconsistency of primary outcome: subgroup of cervical tumor |
|  | (D) Side-splitting model inconsistency of primary outcome: subgroup of ovarian tumor |
|  | (E) Side-splitting model inconsistency of primary outcome: subgroup of breast tumor |
|  | (F) Side-splitting model inconsistency of primary outcome: subgroup of vaginal tumor |
|  | (G) Side-splitting model inconsistency of primary outcome: subgroup of vulvar tumor |
|  | (H) Side-splitting model inconsistency of acceptability: drop-out rate |
|  | (I) Design-by-treatment model and loop inconsistency of primary outcome: overall gynecologic tumor |
| eTable 8 | Heterogeneity of primary outcome: overall gynecologic tumor |
| eTable 9 | (A) GRADE of primary outcome: overall gynecologic tumor |
|  | (B) GRADE of primary outcome: subgroup of intra-uterus tumor |
|  | (C) GRADE of primary outcome: subgroup of cervical tumor |
|  | (D) GRADE of primary outcome: subgroup of ovarian tumor |
|  | (E) GRADE of primary outcome: subgroup of breast tumor |
|  | (F) GRADE of primary outcome: subgroup of vaginal tumor |
|  | (G) GRADE of primary outcome: subgroup of vulvar tumor |
|  | (H) GRADE of acceptability: drop-out rate |

**eFigure 1A Network structure of NMA of the primary outcome: subgroup of uterine tumor**

**
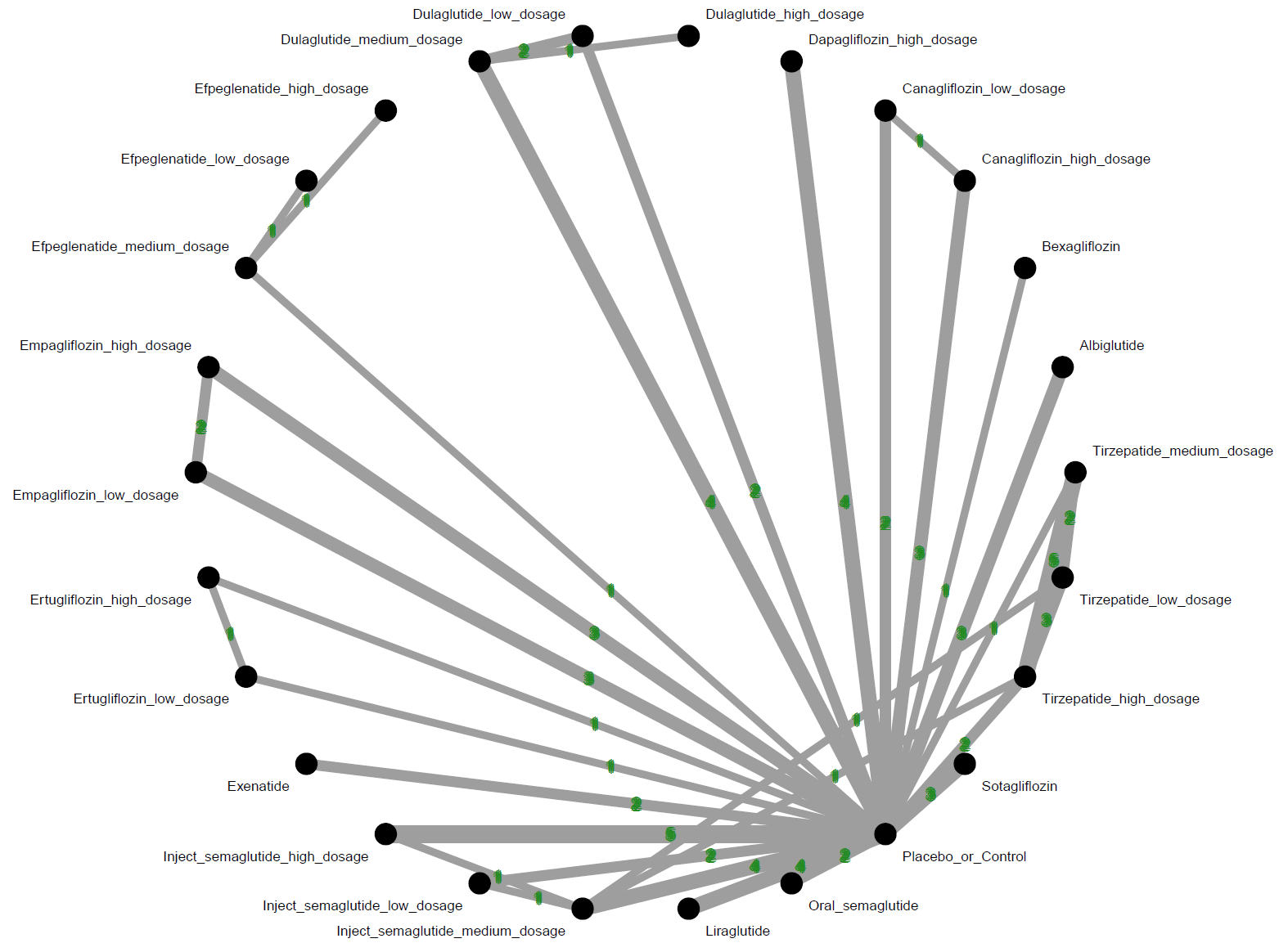
**

**eFigure 1B Network structure of NMA of the primary outcome: subgroup of endometrial tumor**

**
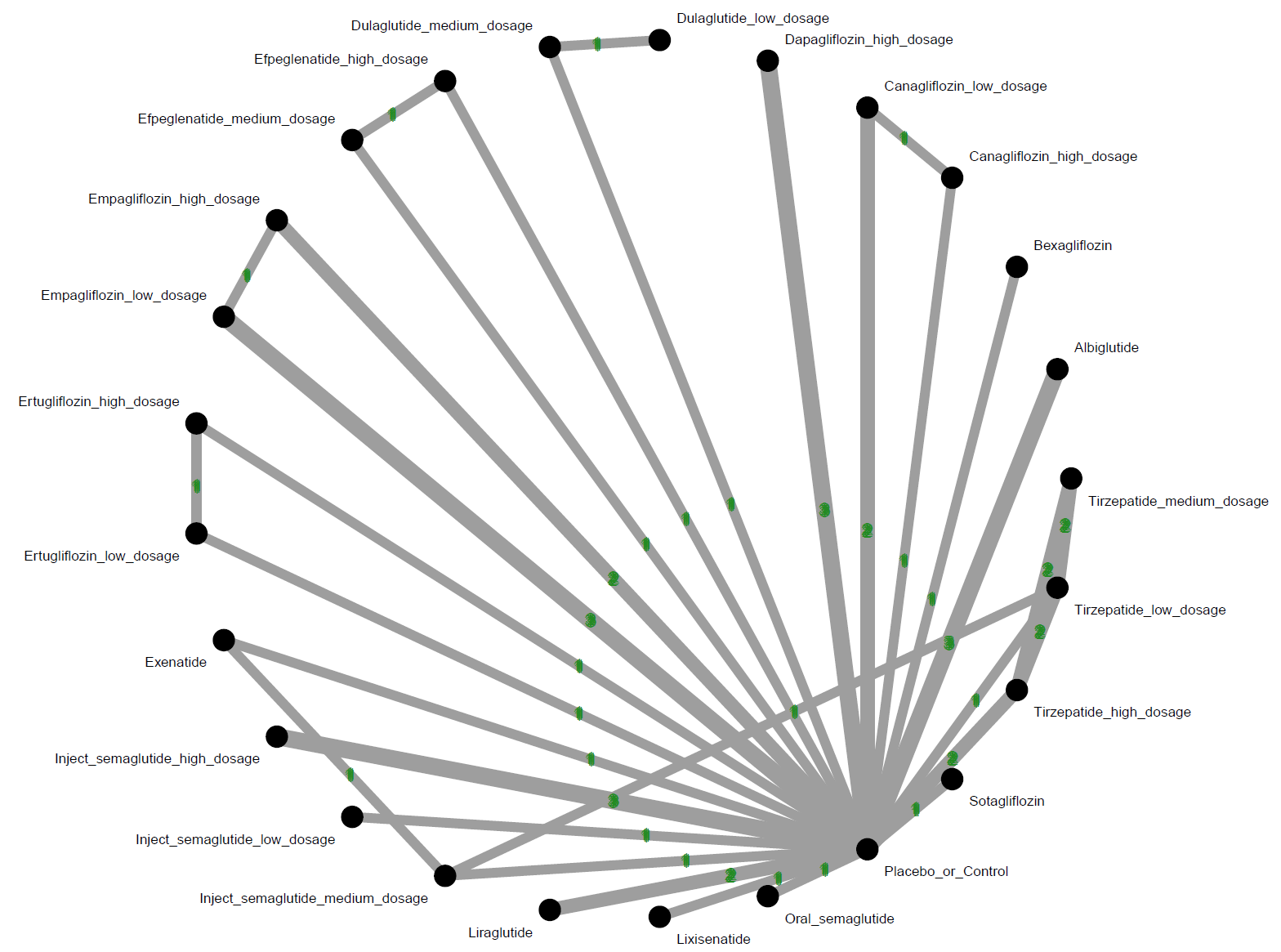
**

**eFigure 1C Network structure of NMA of acceptability: drop-out rate***

**
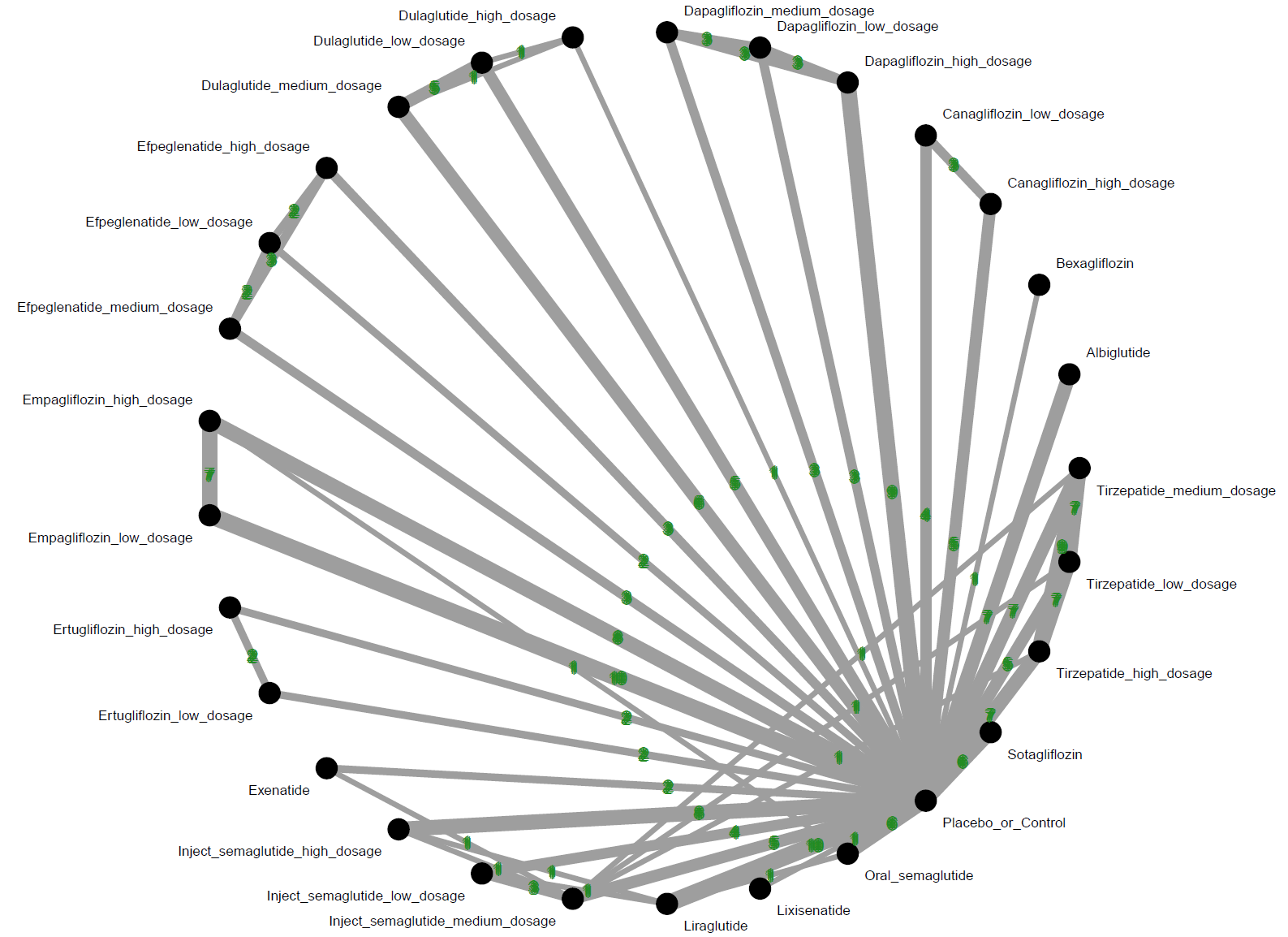
**

**Figure legend of eFigure 1A-1C**

The structure of the network meta-analysis. The lines between nodes represent direct comparisons from various trials, with the numbers over the lines indicating the number of trials providing these comparisons for each specific treatment. The thickness of the lines corresponds to the number of trials linked to the network.

* The outcome of drop-out rate here was calculated according to drop-out rate data from the original composition of subjects from included studies because there were no any studies provided specific information regarding drop-out rate data in female subgroup.

***Abbreviation for eFigure 1A-1C:***

*95%CIs: 95% confidence intervals; GLP-1 agonist: glucagon-like peptide-1 agonist; NMA: network meta-analysis; OR: odds ratio; RCT: randomized controlled trial; SGLT2 inhibitor: sodium–glucose cotransporter 2 inhibitor*

**eFigure 2A Forest plot of NMA of the primary outcome: subgroup of uterine tumor**

**
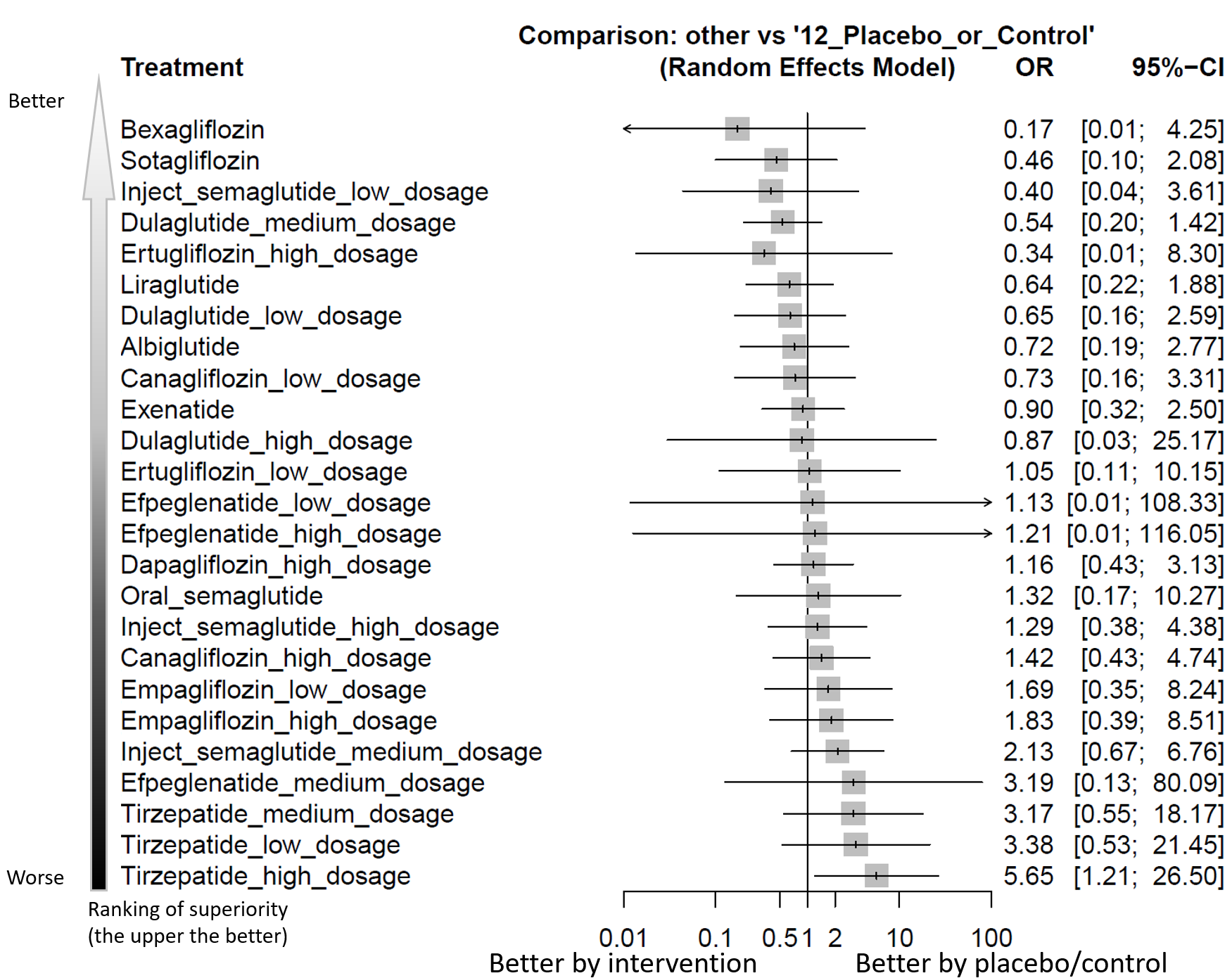
**

**eFigure 2B Forest plot of NMA of the primary outcome: subgroup of endometrial tumor**

**
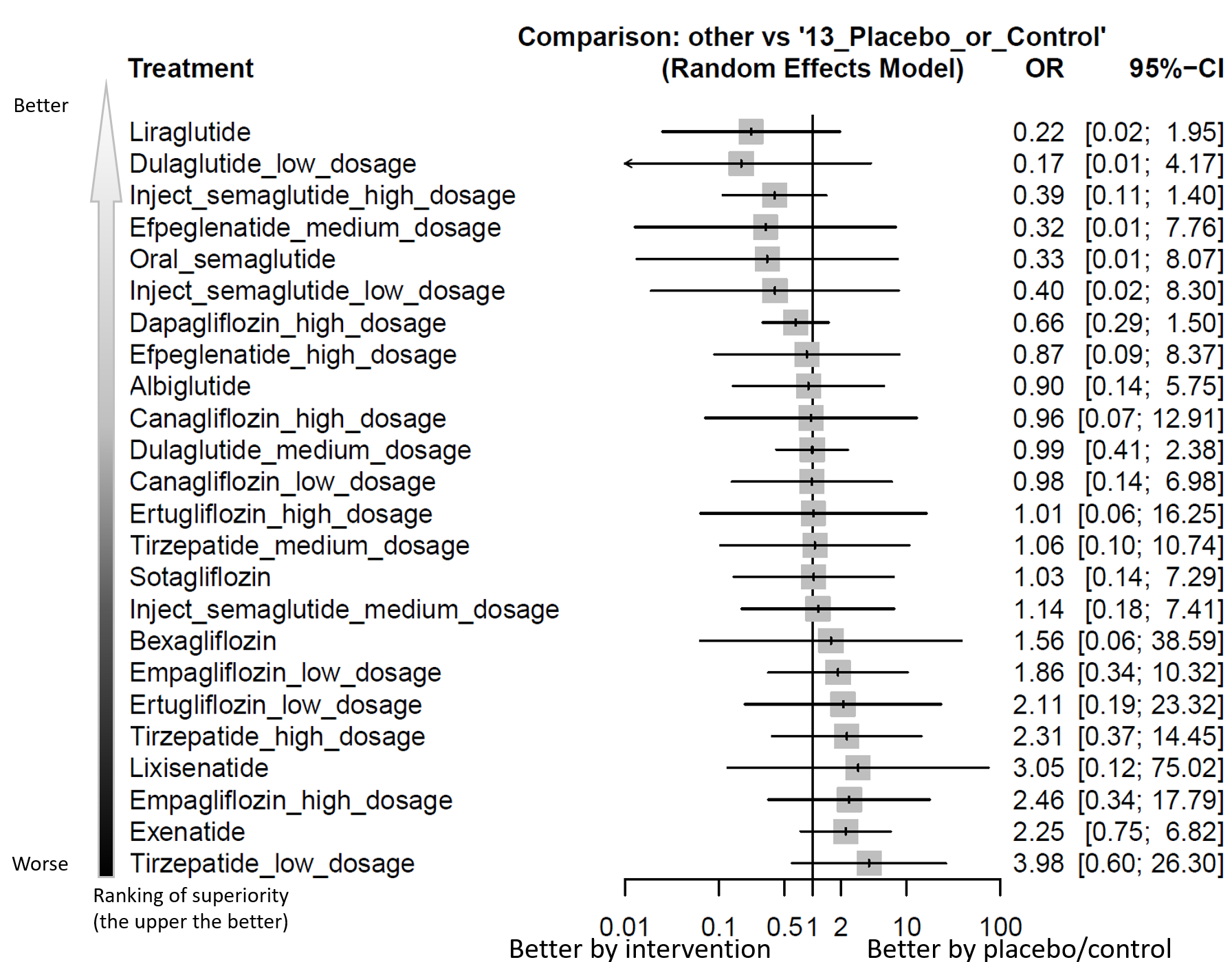
**

**eFigure 2C Forest plot of NMA of acceptability: drop-out rate***

**
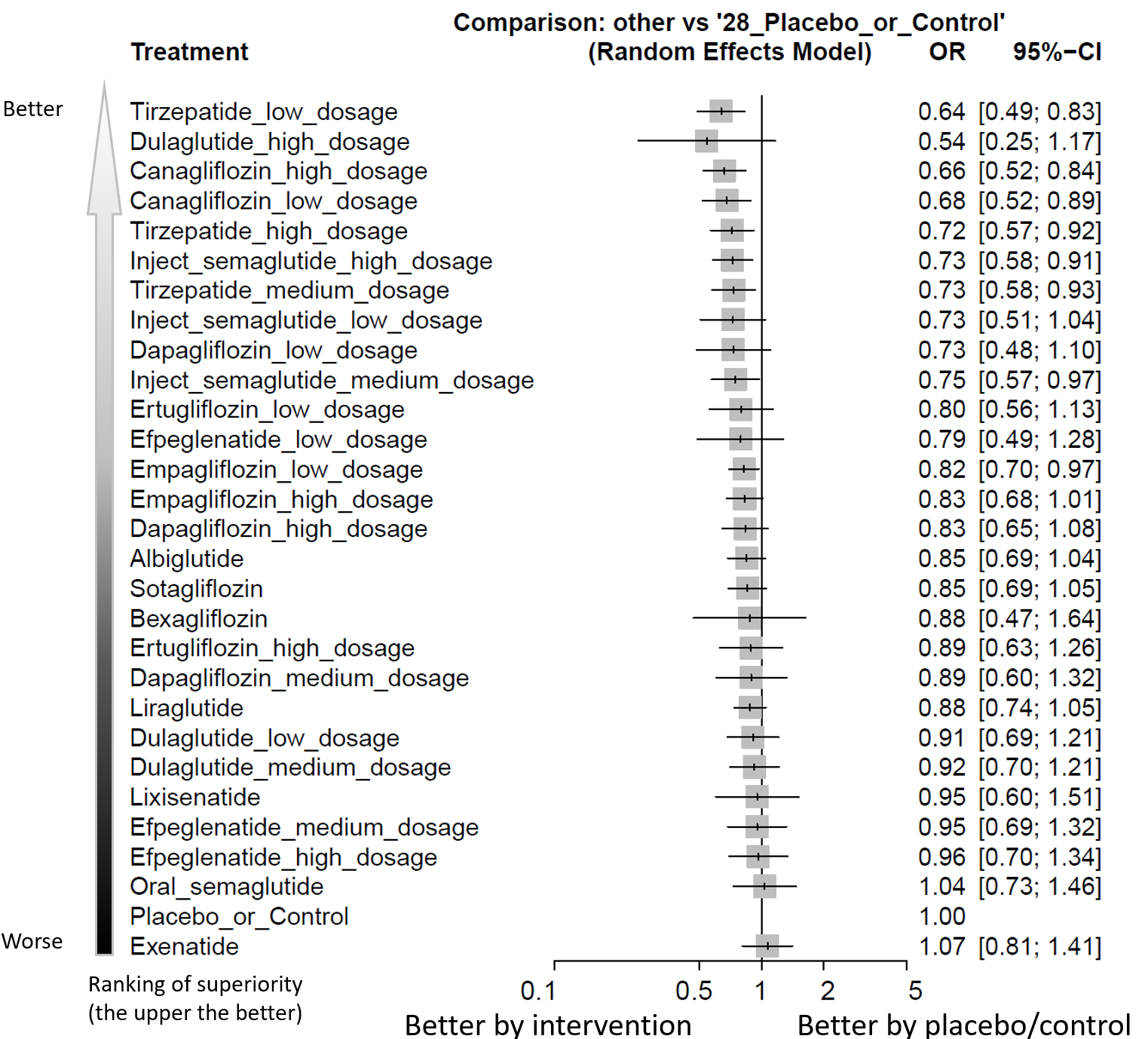
**

**Figure legend of eFigure 2A-2C**

* The outcome of drop-out rate here was calculated according to drop-out rate data from the original composition of subjects from included studies because there were no any studies provided specific information regarding drop-out rate data in female subgroup.

***Abbreviation for eFigure 2A-2C:***

*95%CIs: 95% confidence intervals; GLP-1 agonist: glucagon-like peptide-1 agonist; NMA: network meta-analysis; OR: odds ratio; RCT: randomized controlled trial; SGLT2 inhibitor: sodium–glucose cotransporter 2 inhibitor*

**eFigure 3A Individual study result of primary outcome: overall gynecologic tumor**

**
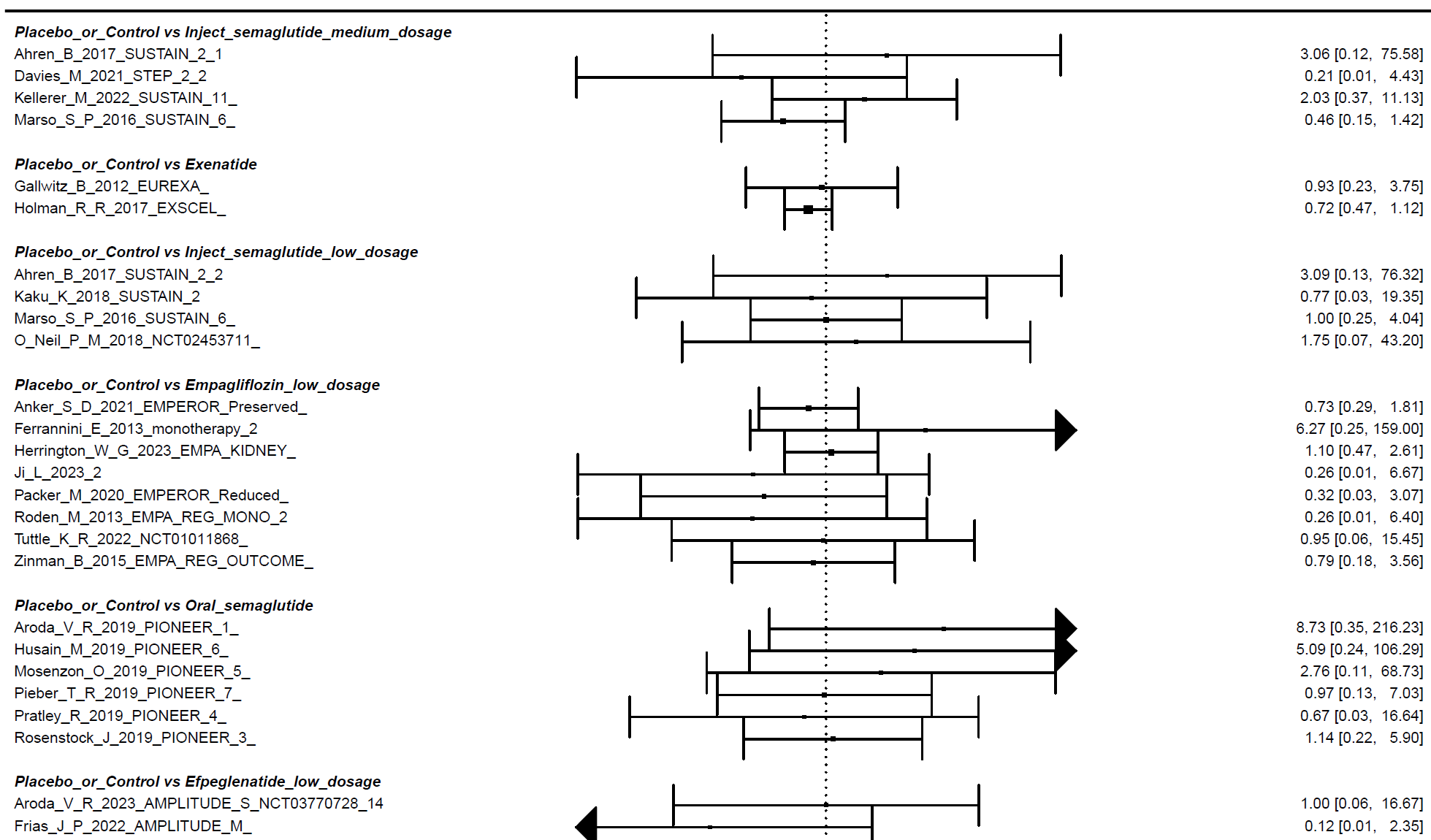
**

**
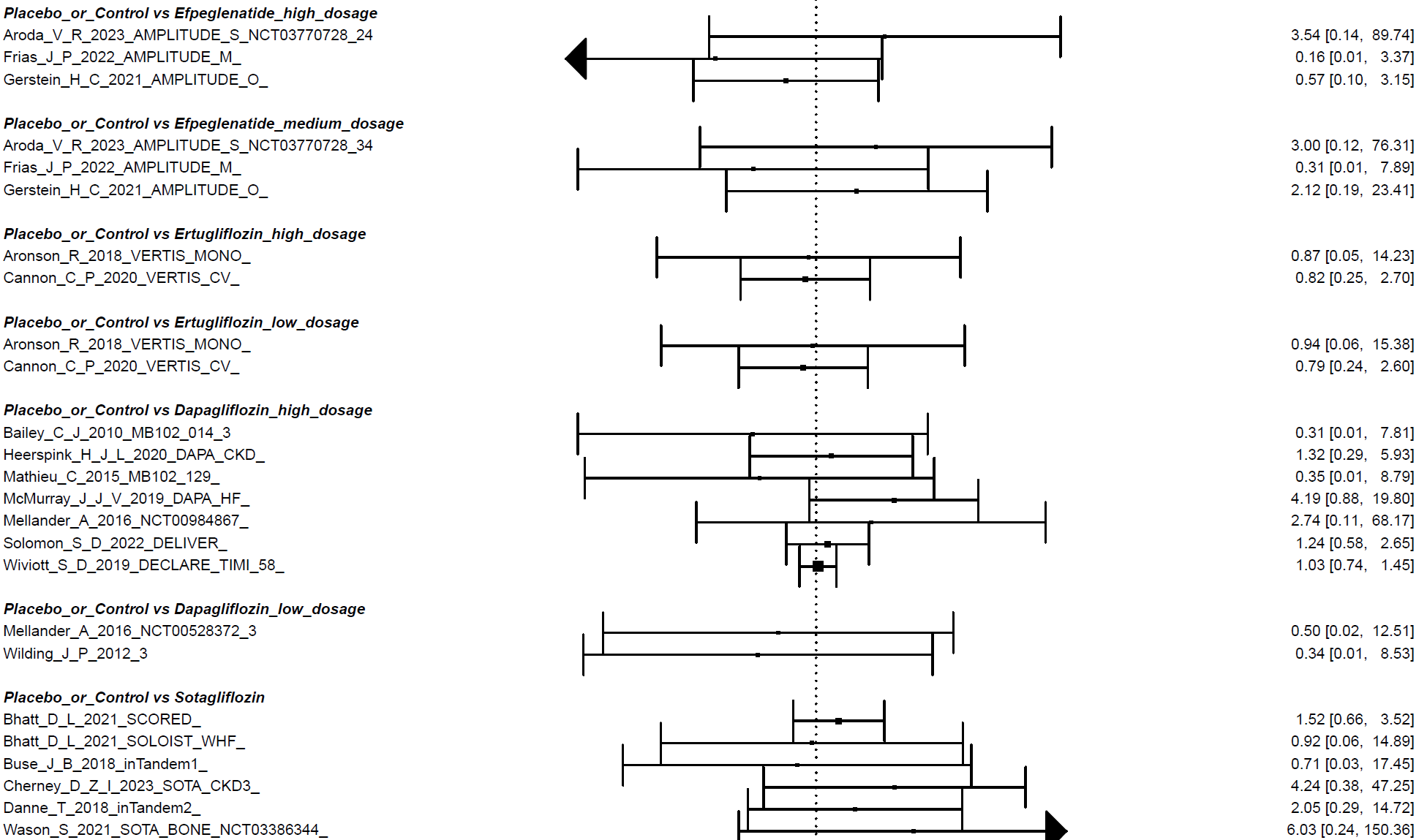
**

**
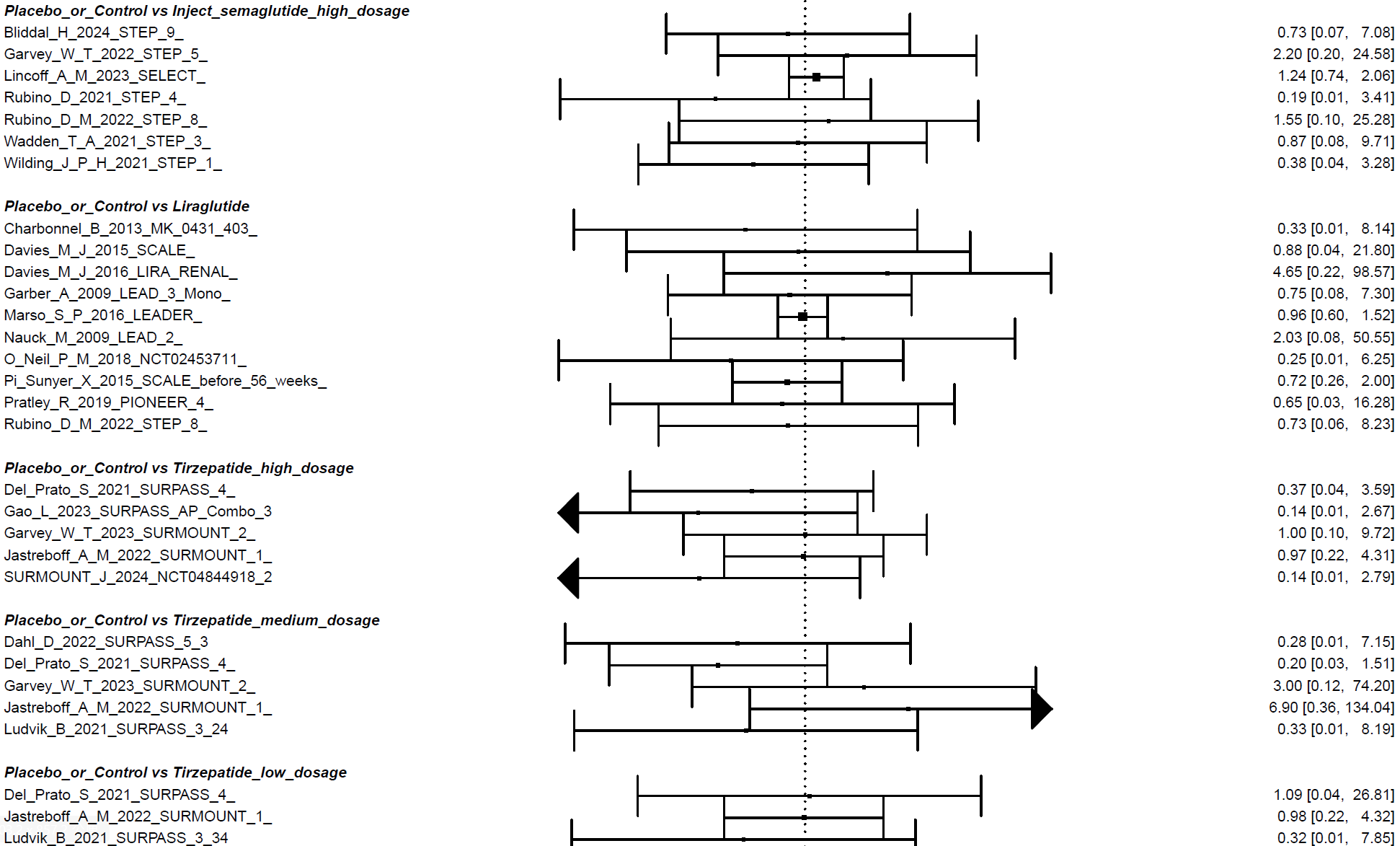
**

**
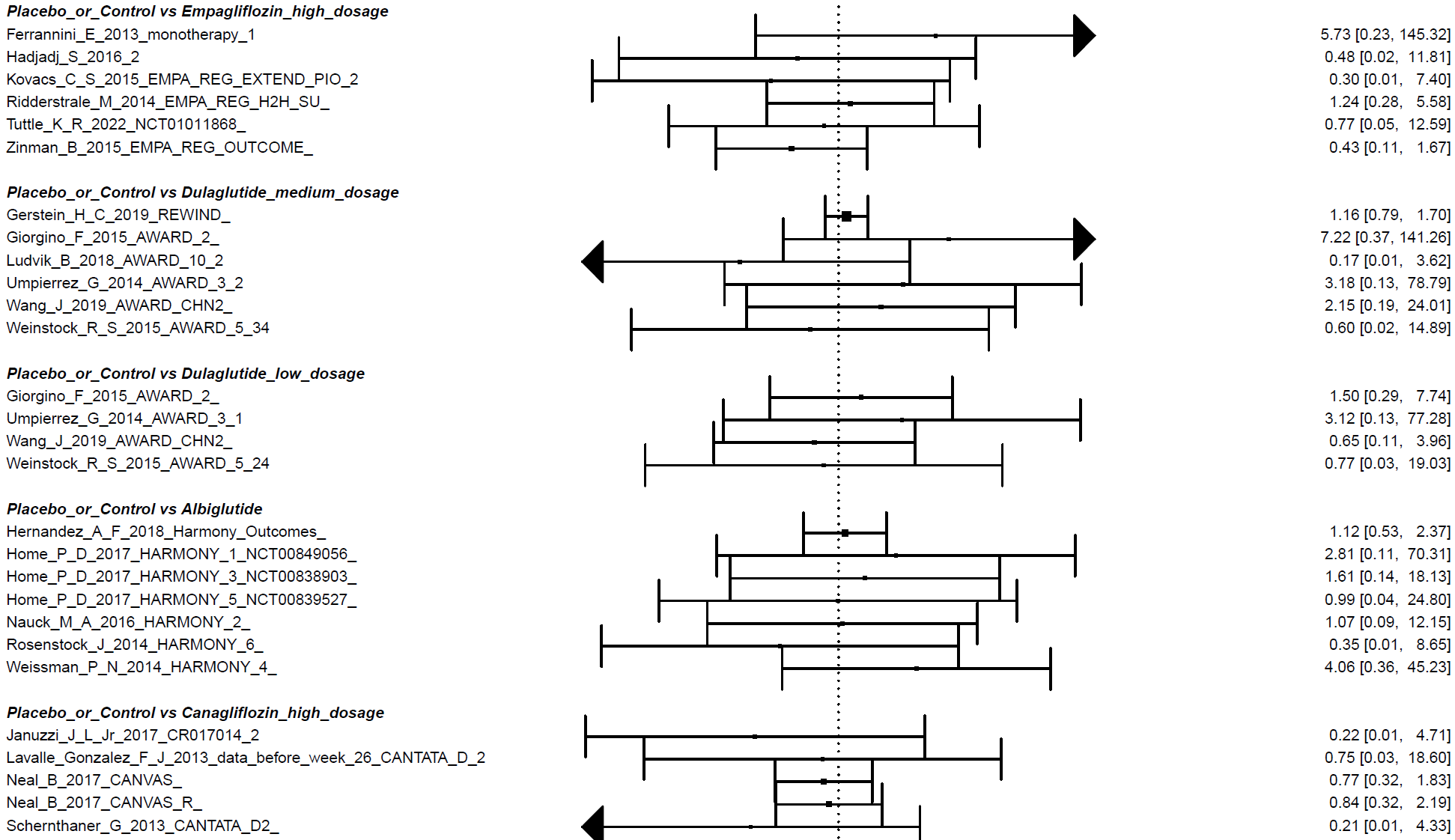
**

**
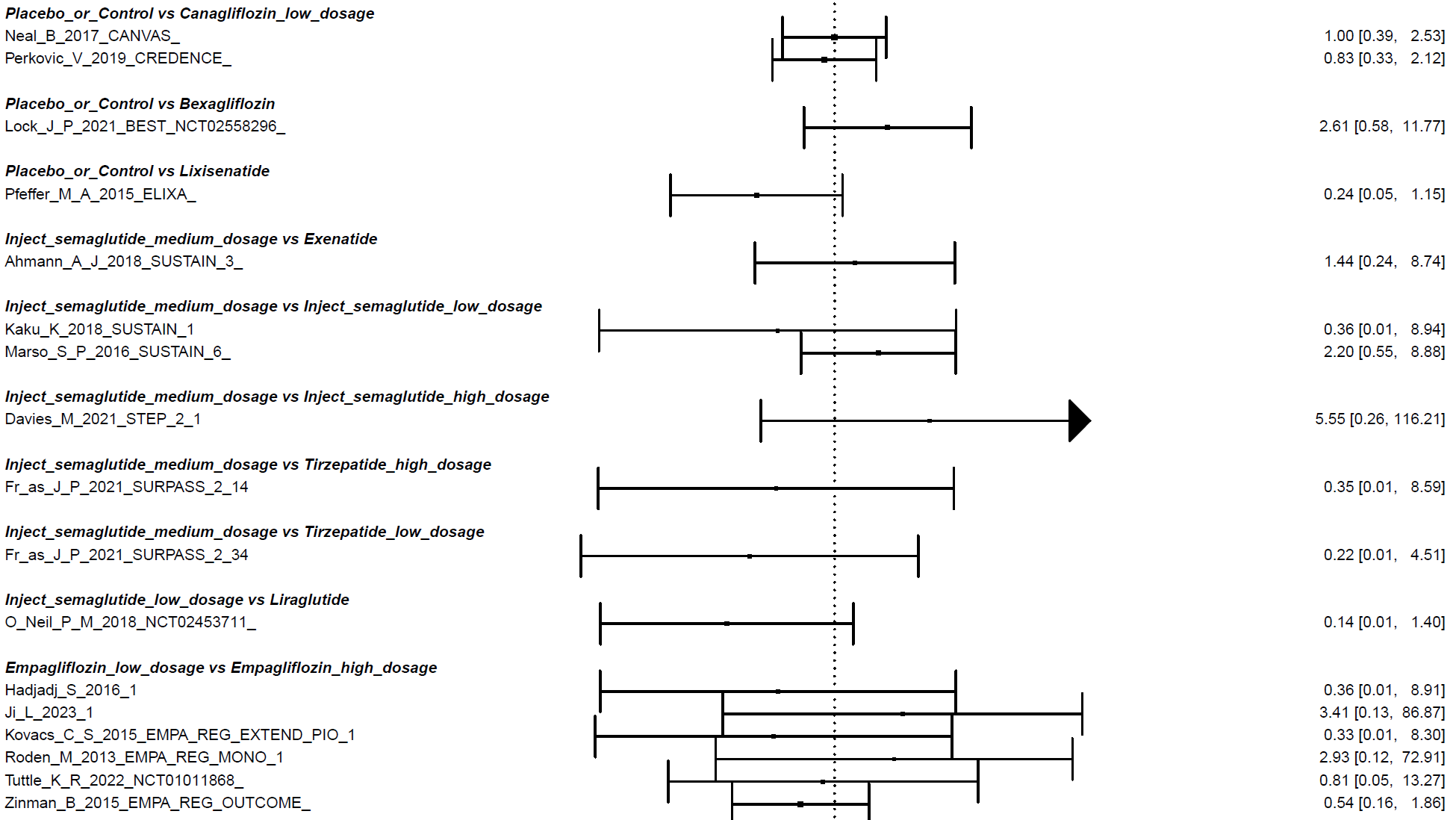
**

**
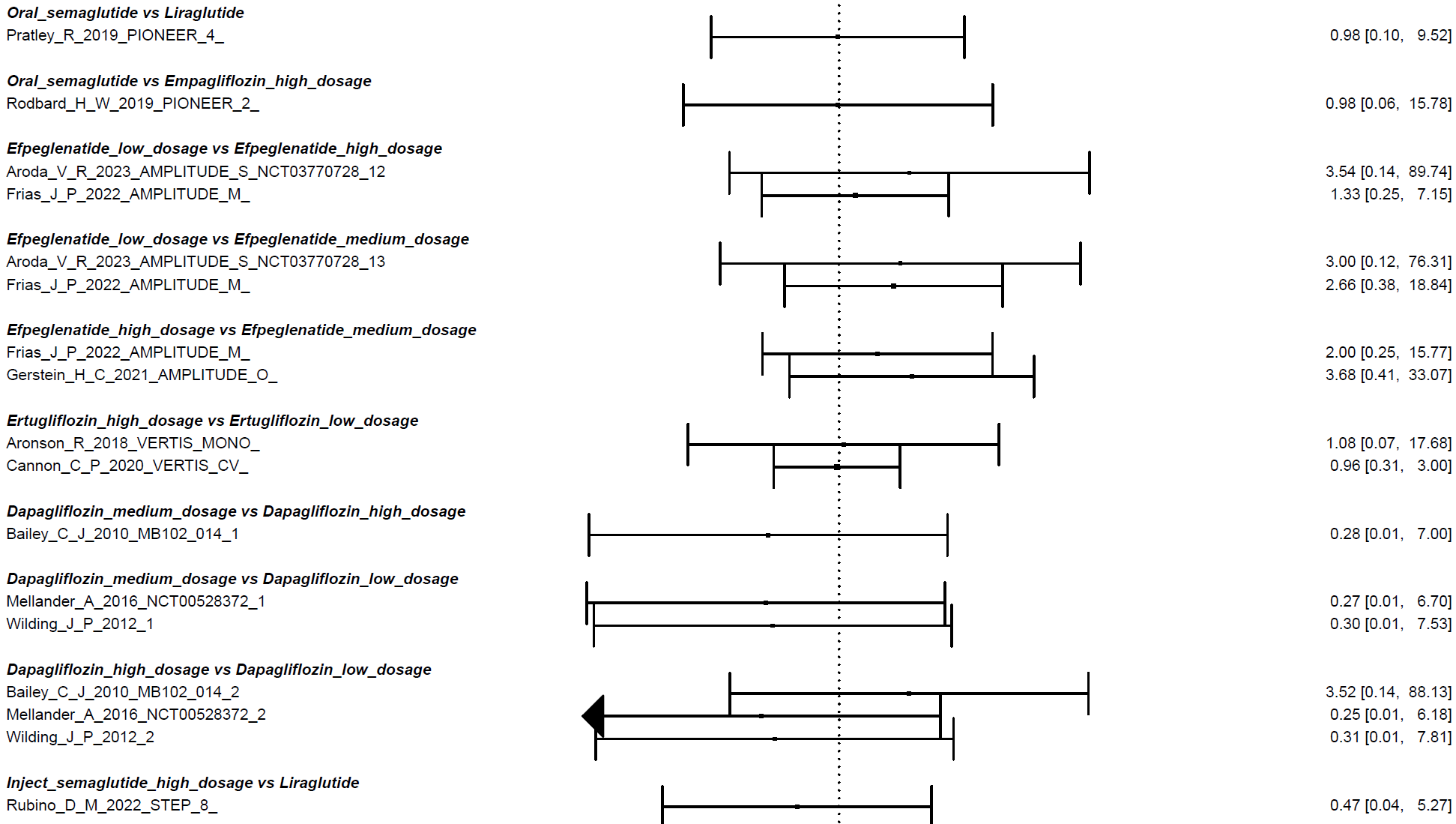
**

**
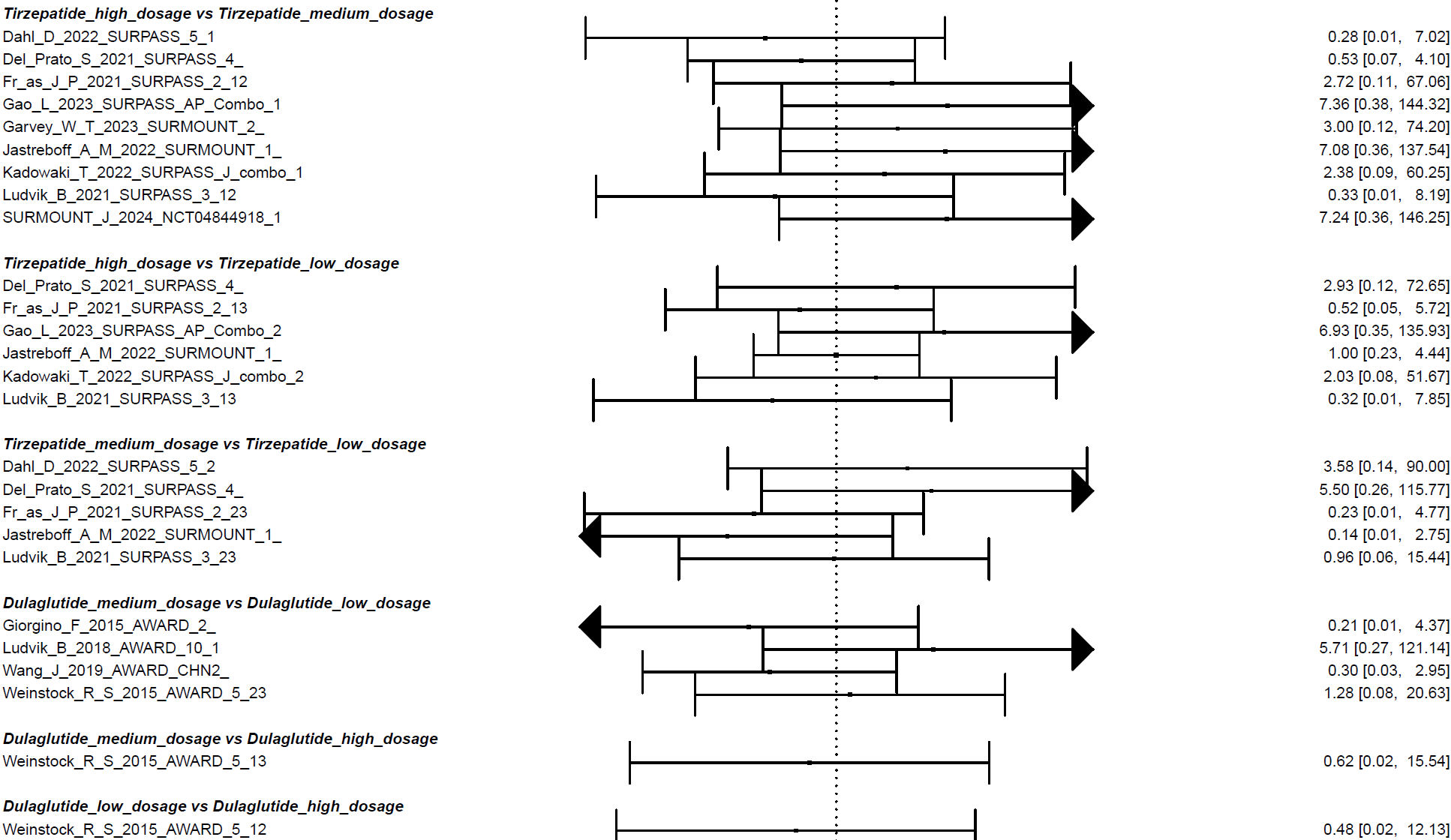
**

**
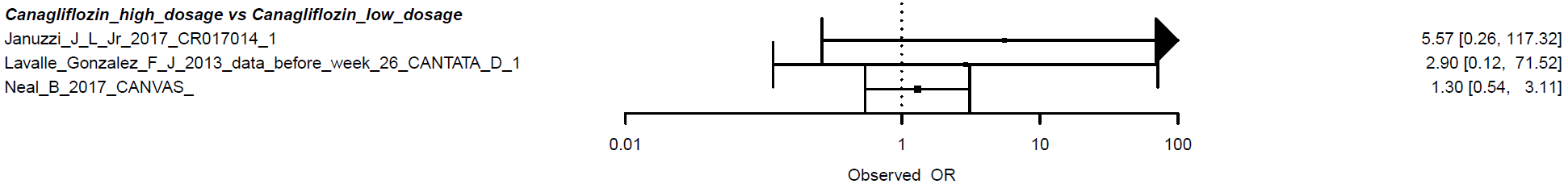
**

**eFigure 3B Individual study result of primary outcome: subgroup of intra-uterus tumor**

**
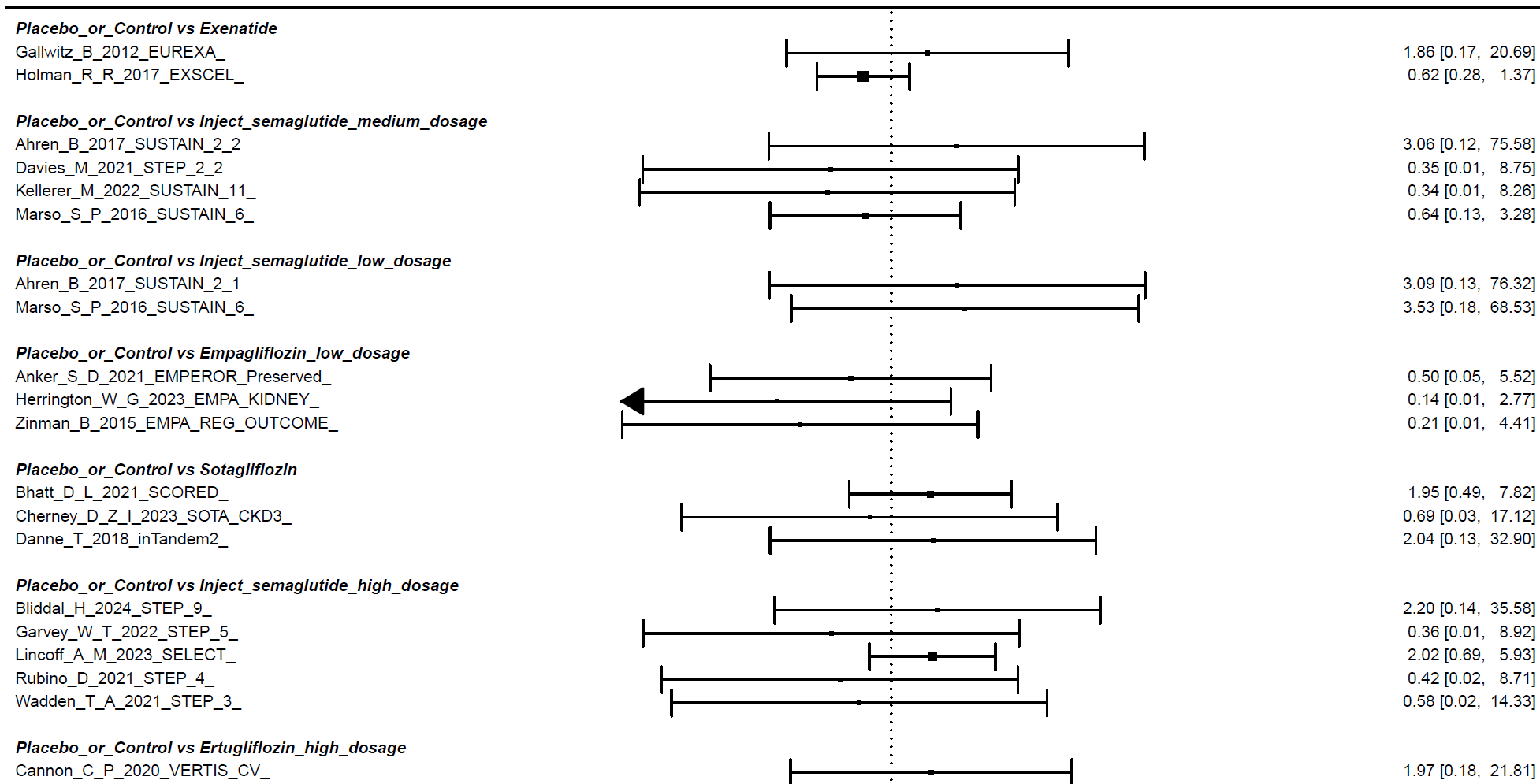
**

**
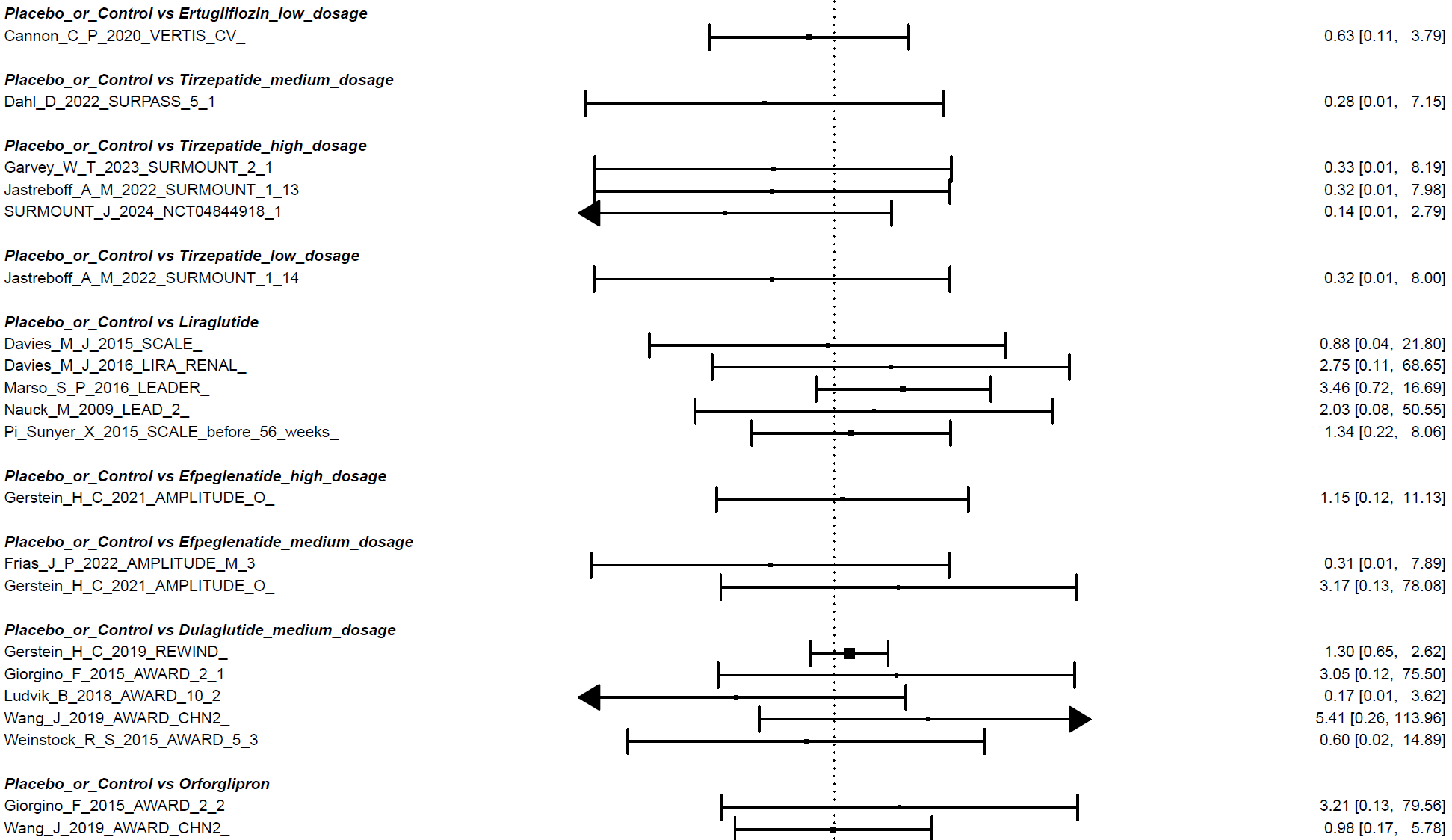
**

**
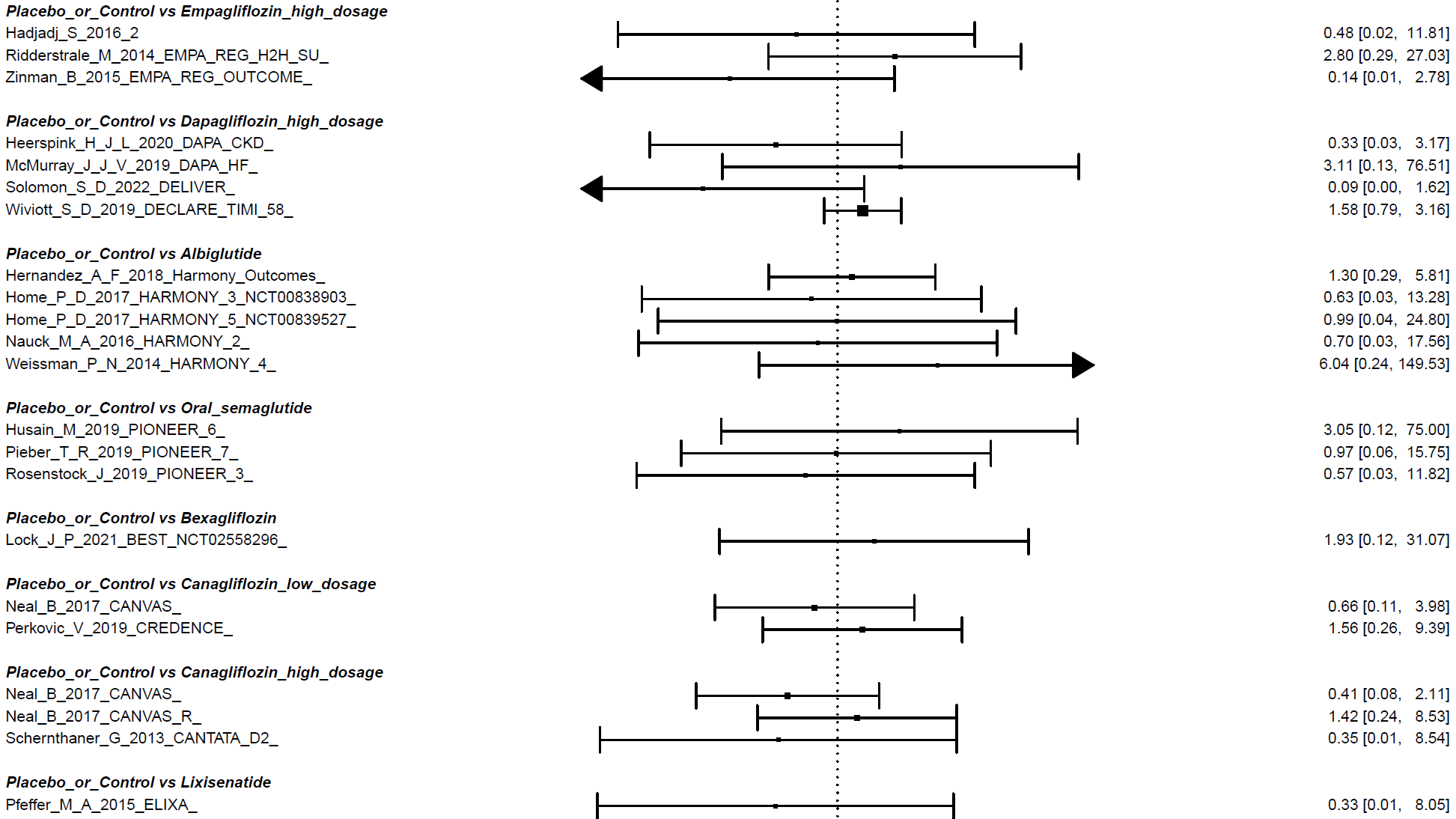
**

**
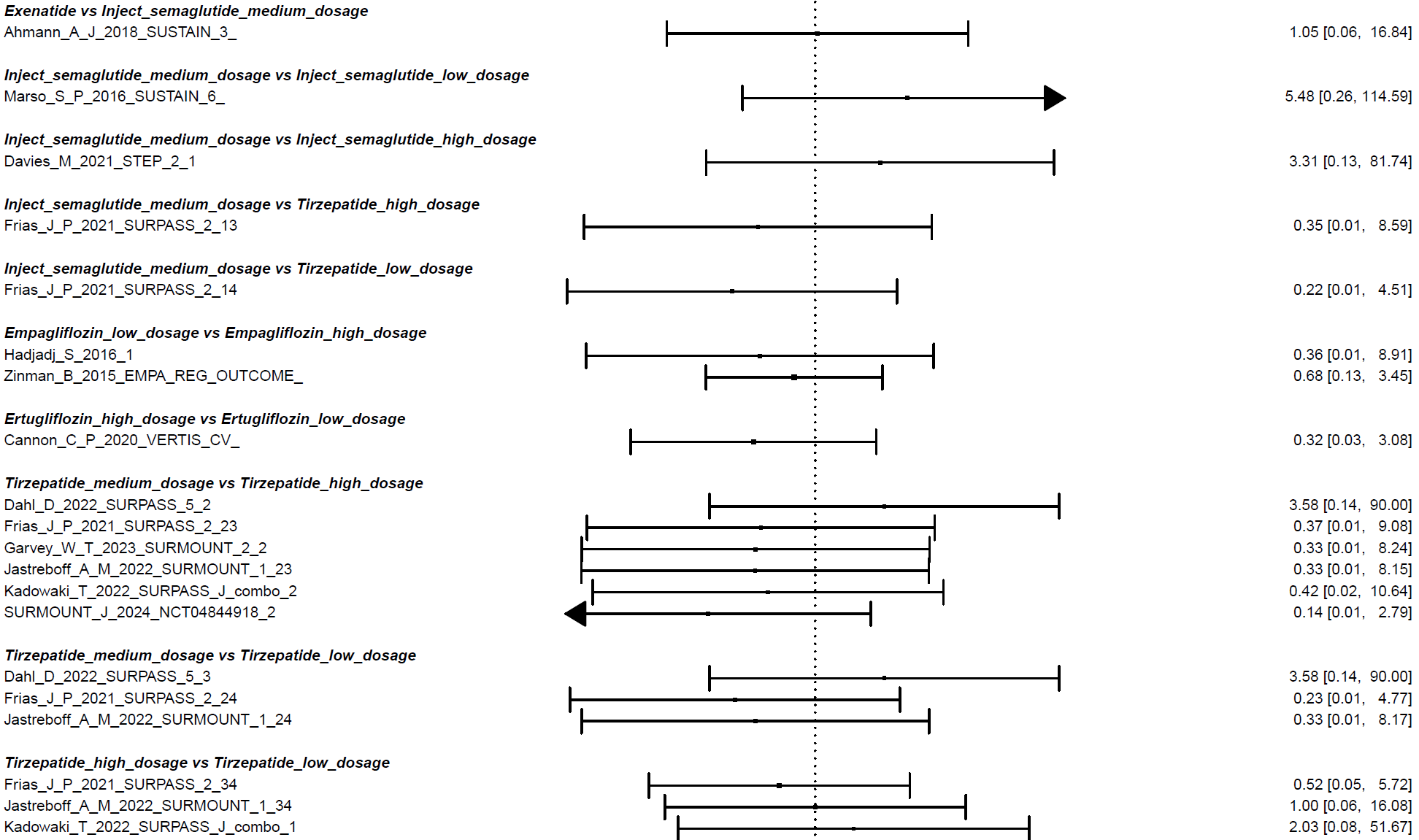
**

**
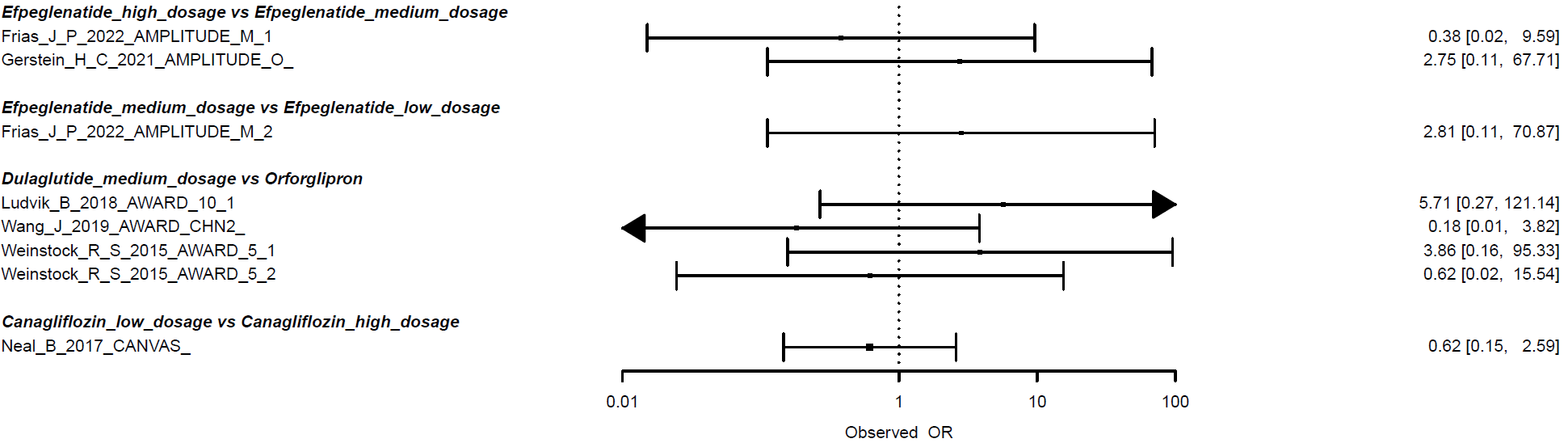
**

**eFigure 3C Individual study result of primary outcome: subgroup of cervical tumor**

**
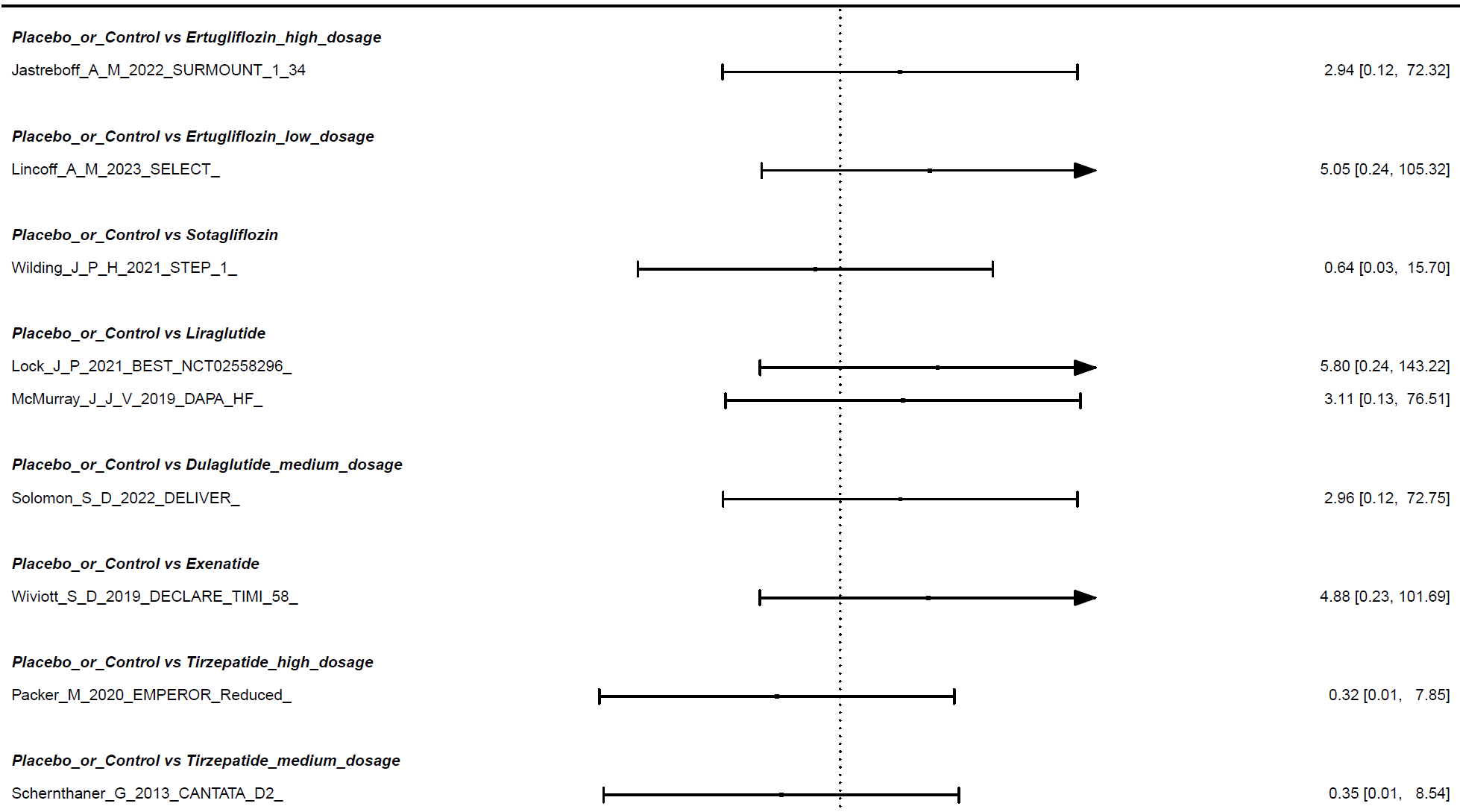
**

**
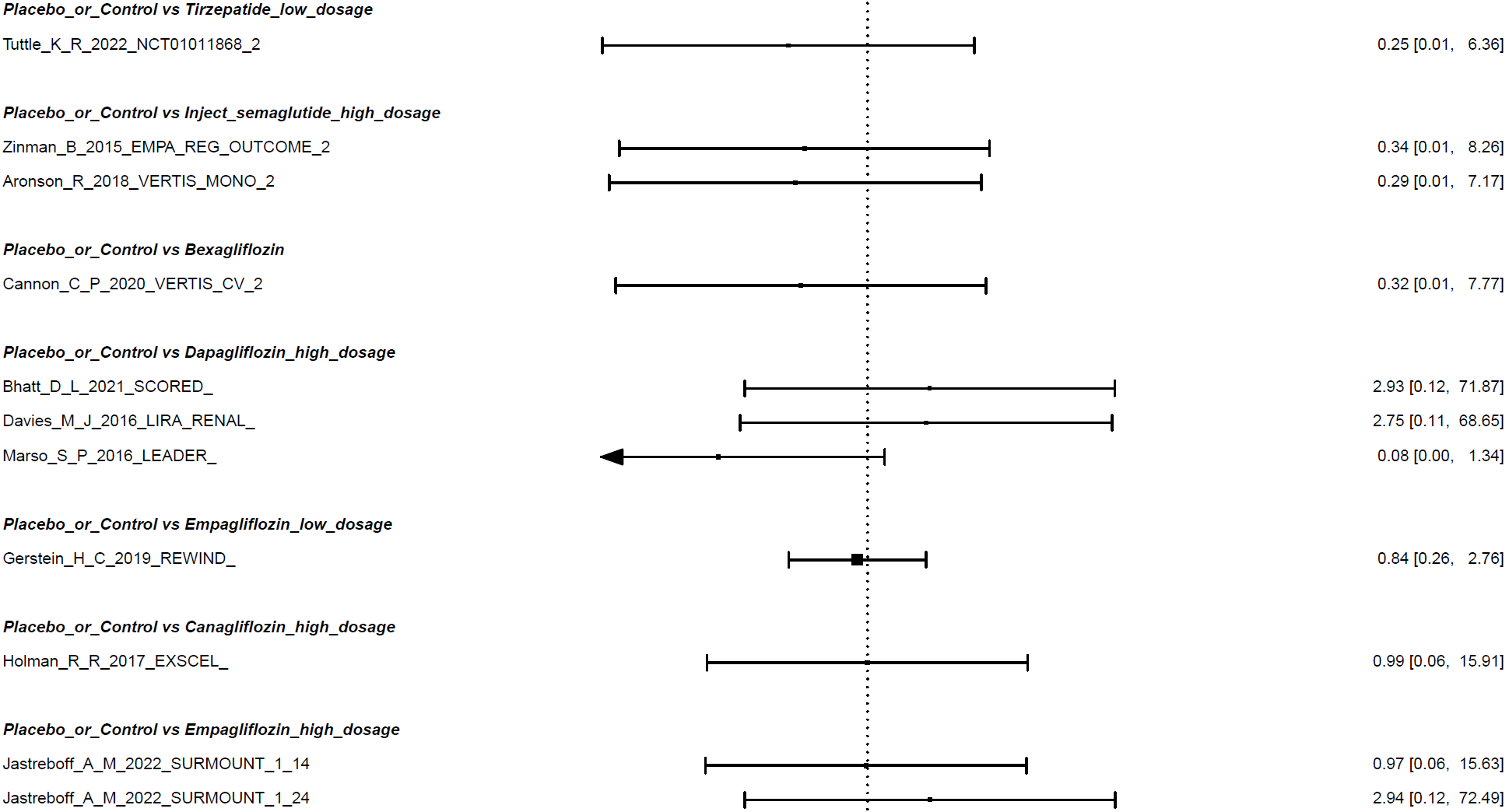
**

**
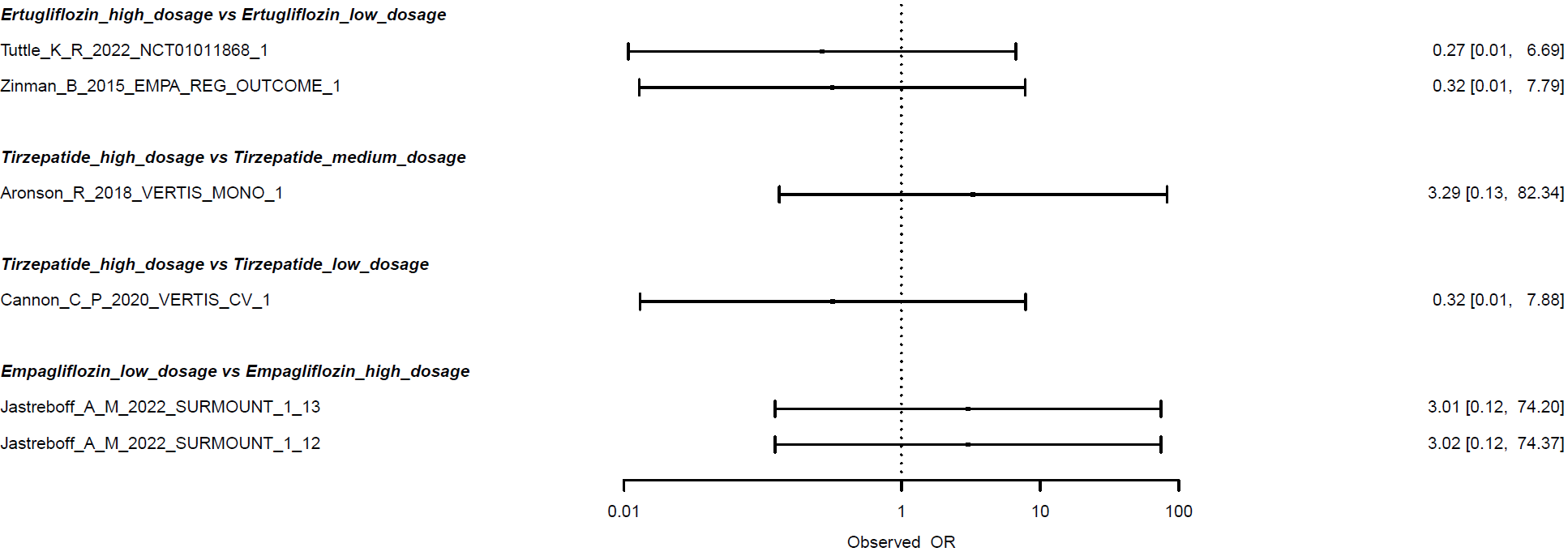
**

**eFigure 3D Individual study result of primary outcome: subgroup of ovarian tumor**

**
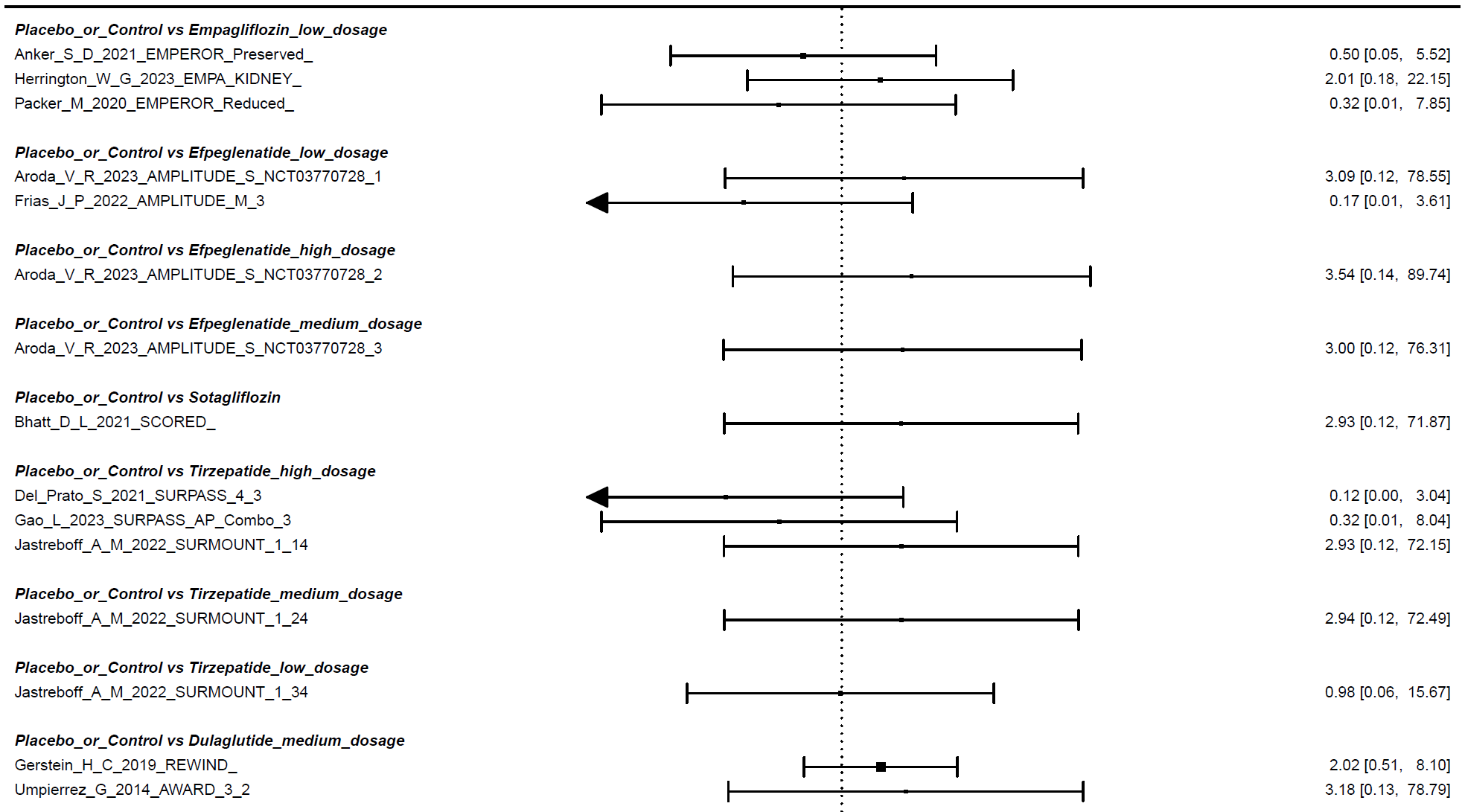
**

**
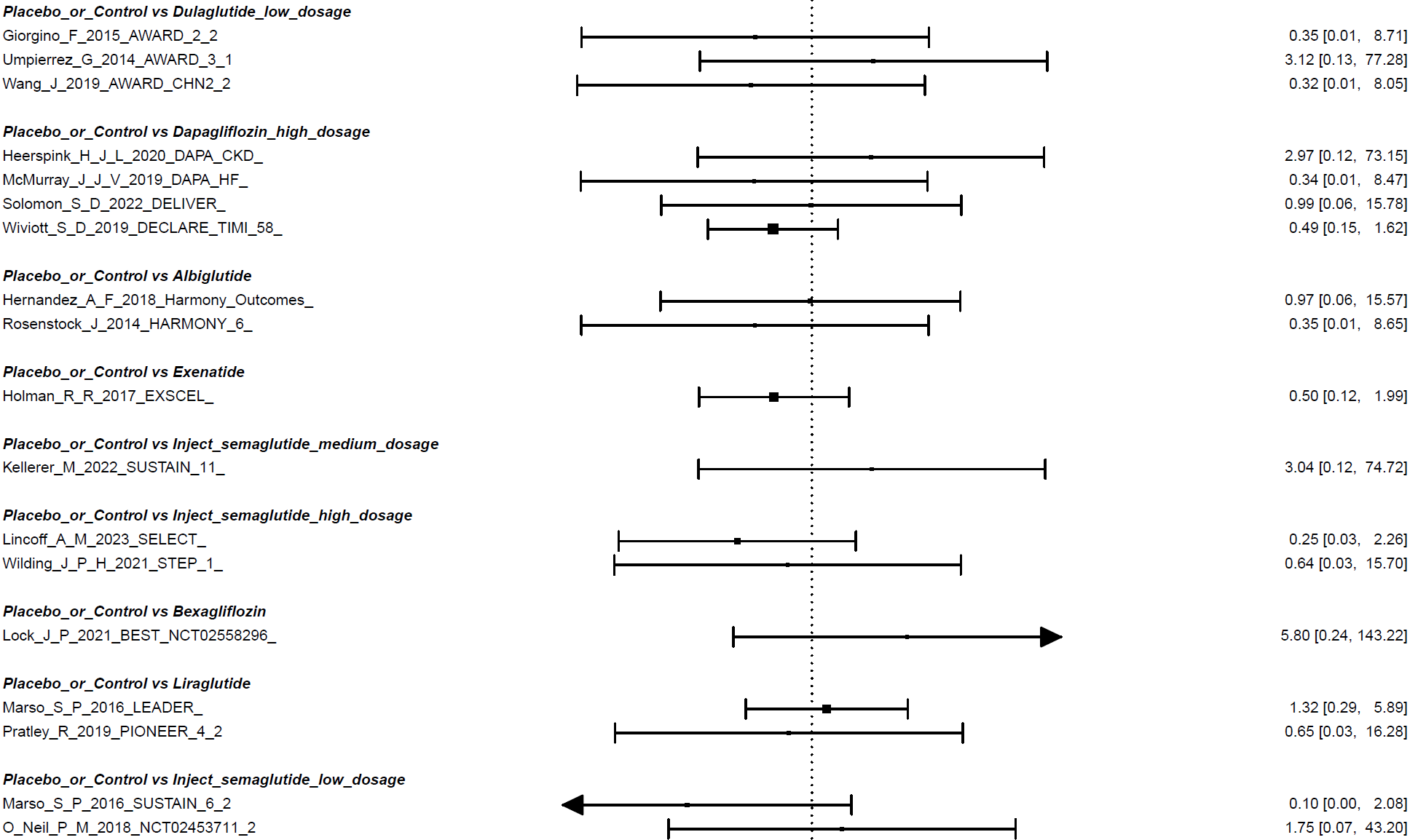
**

**
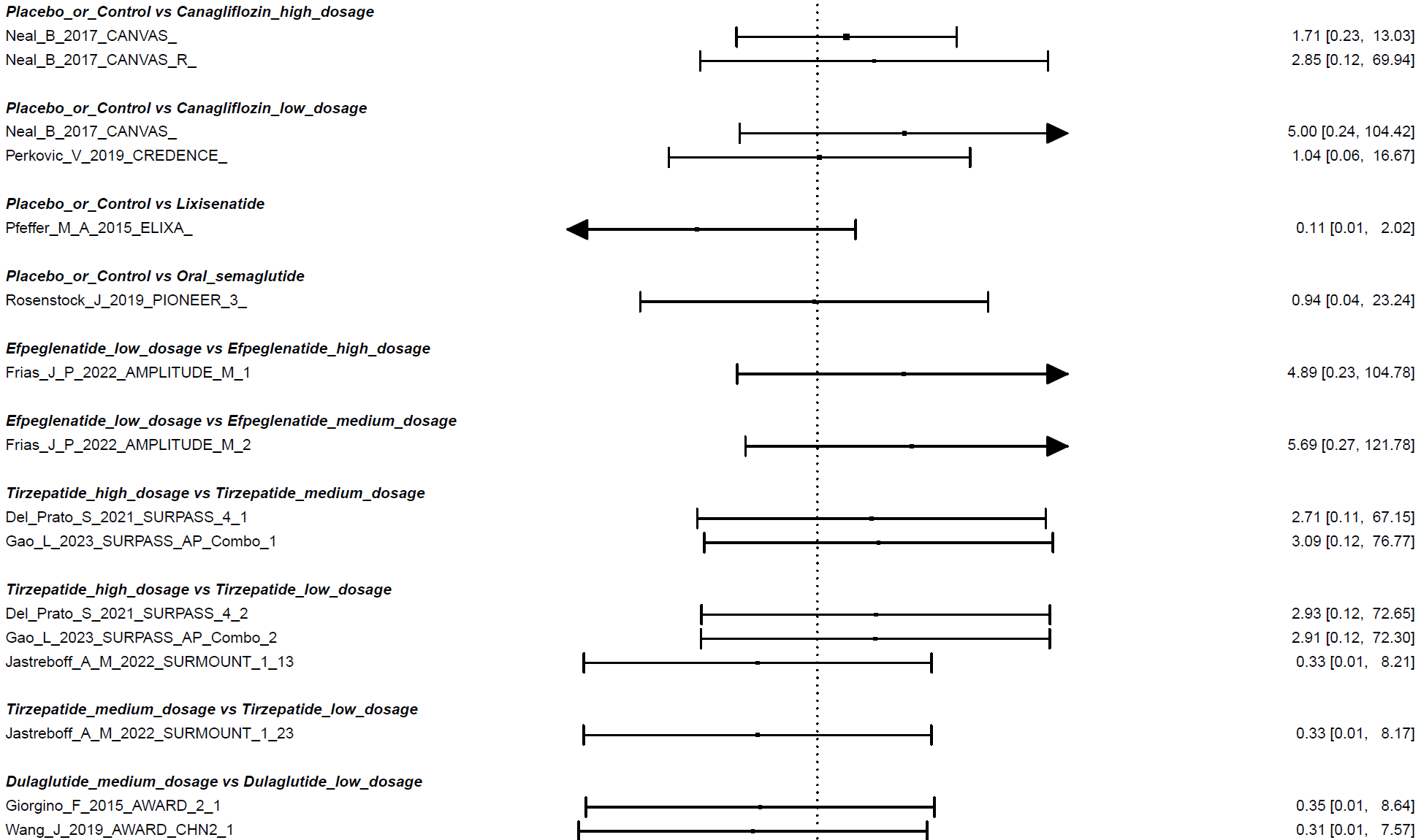
**

**
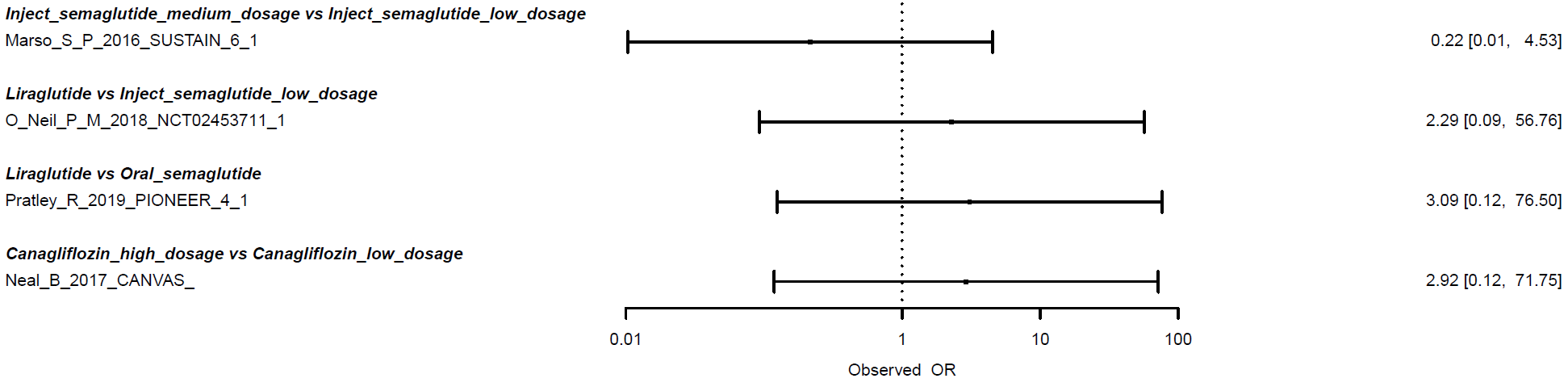
**

**eFigure 3E Individual study result of primary outcome: subgroup of breast tumor**

**
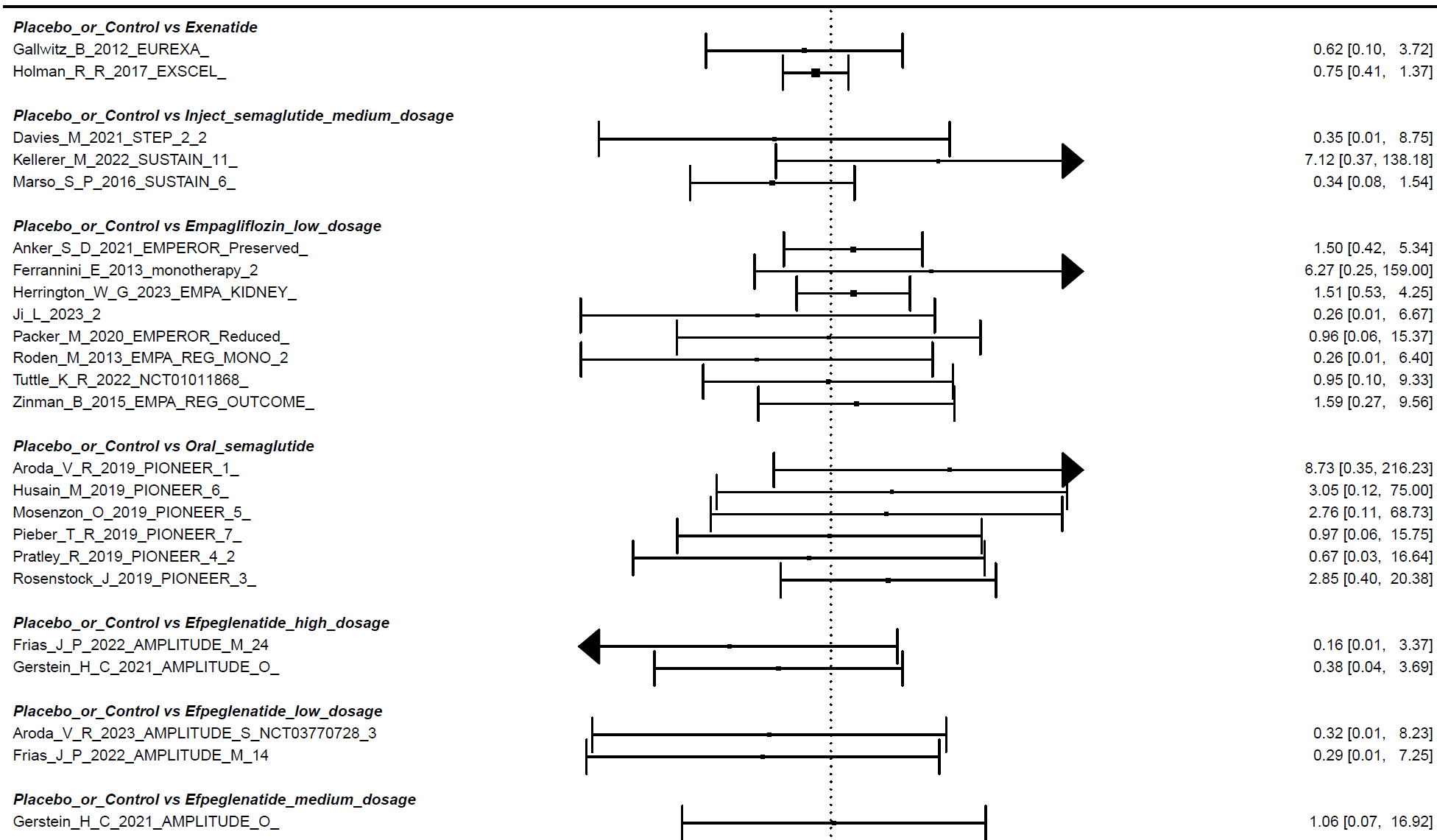
**

**
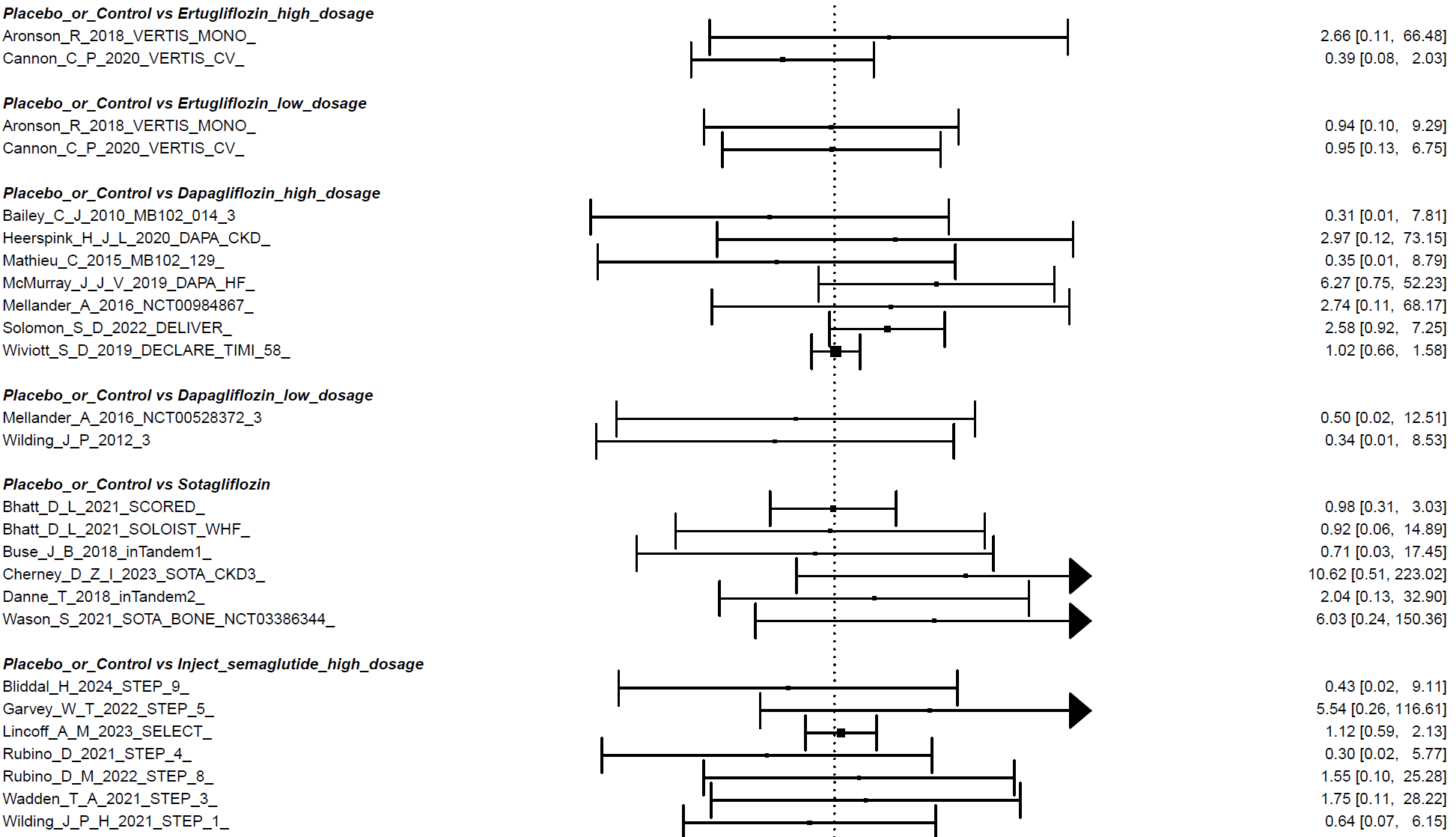
**

**
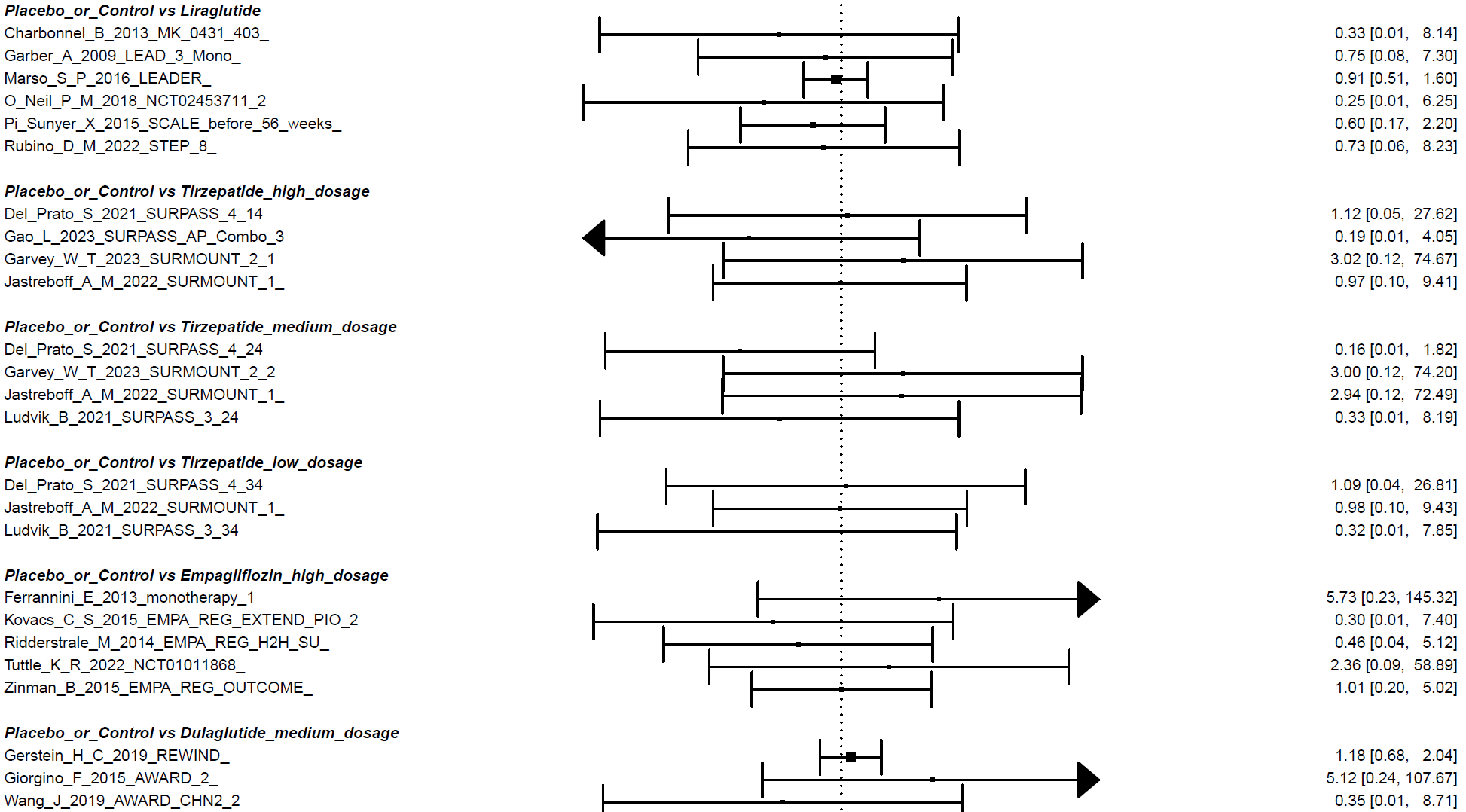
**

**
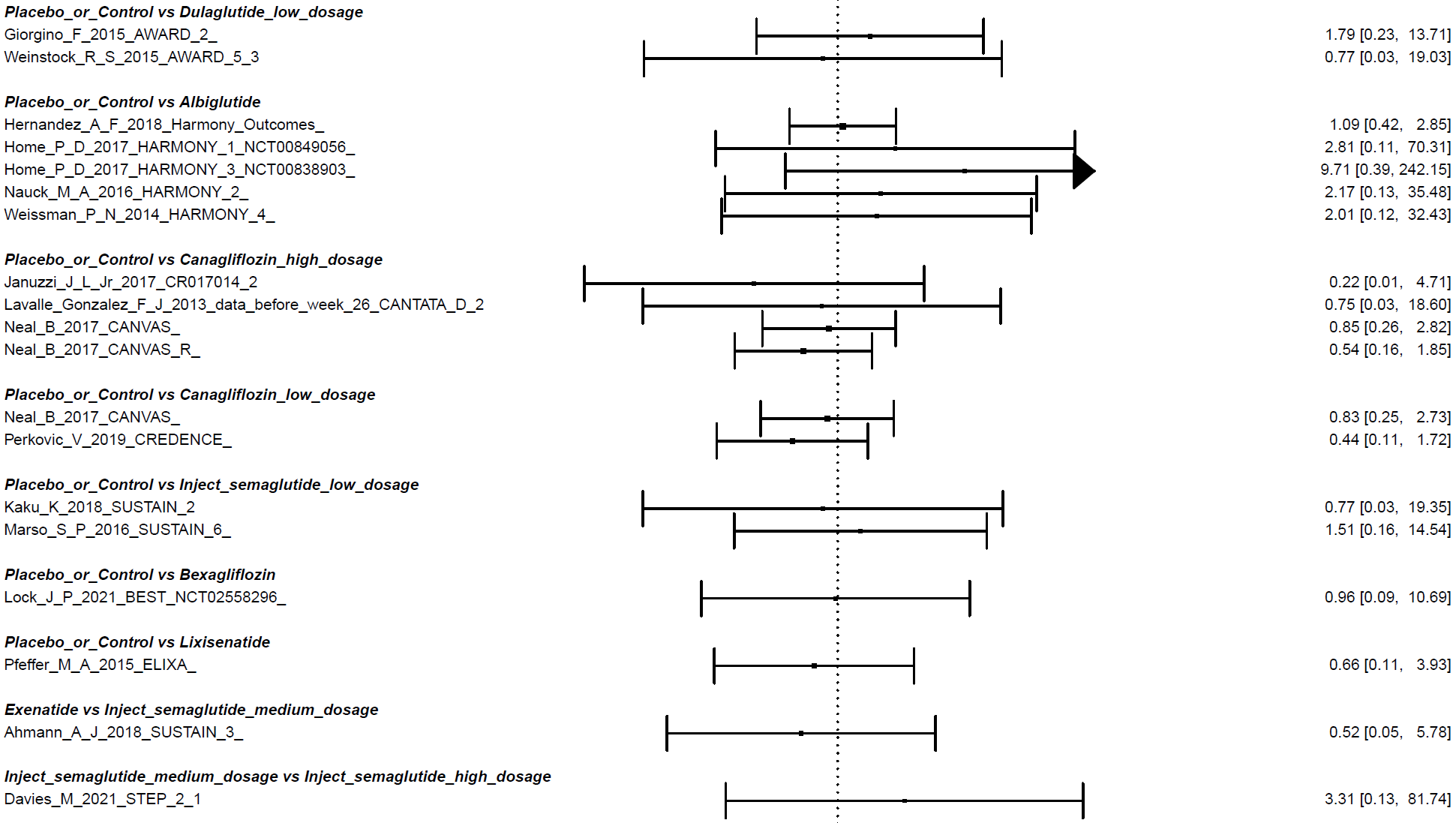
**

**
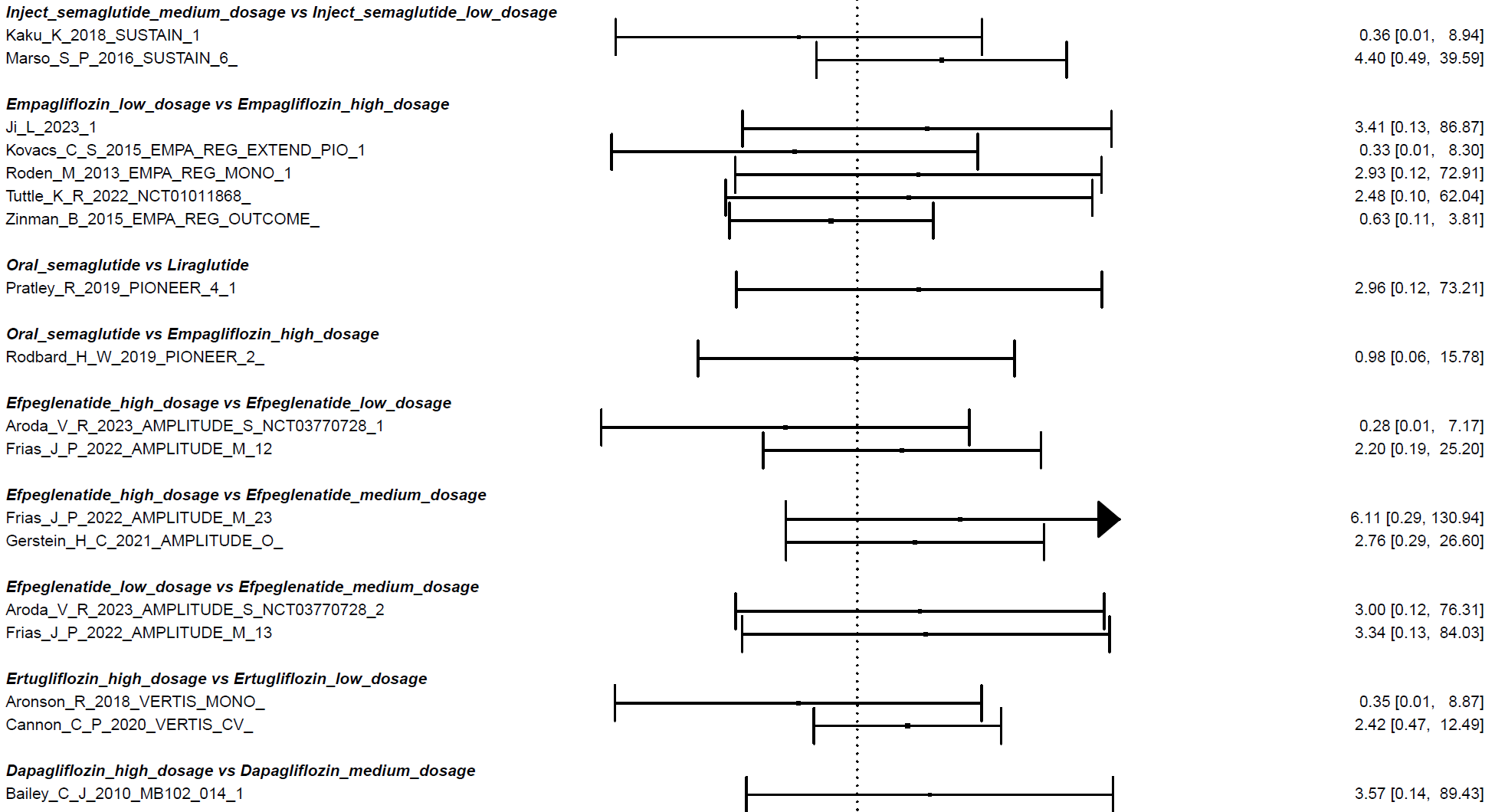
**

**
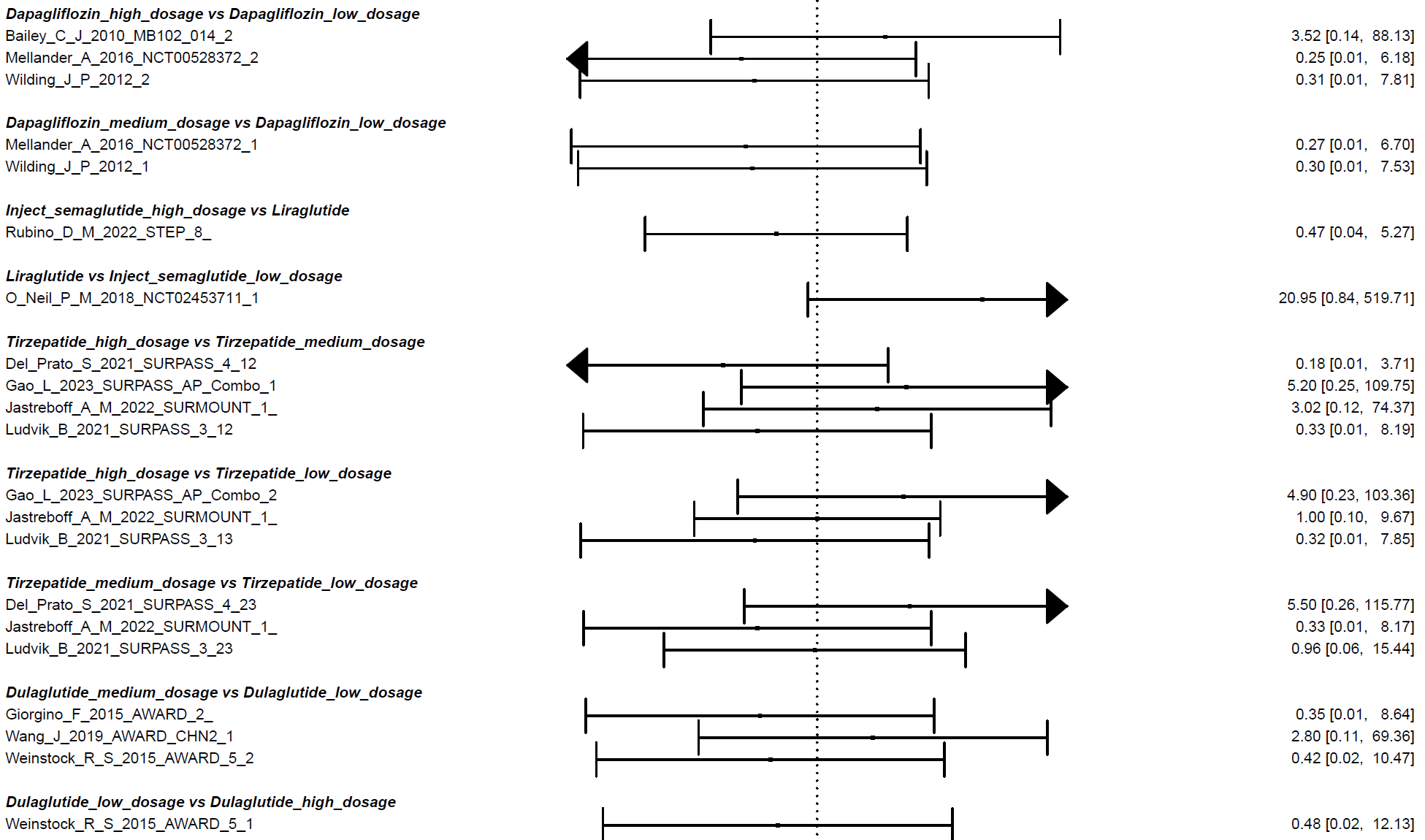
**

**
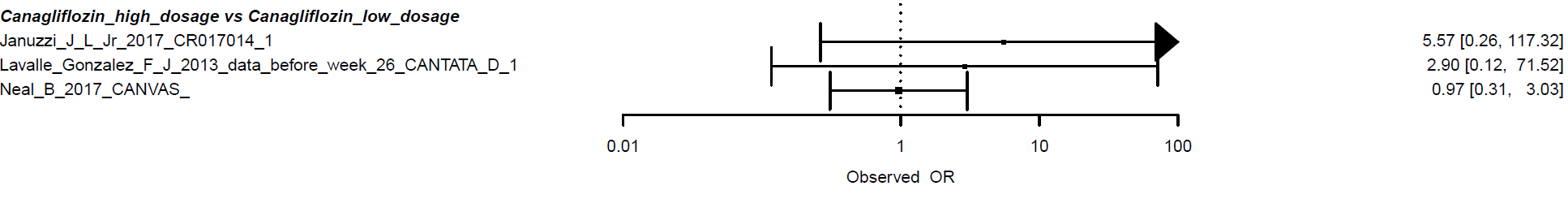
**

**eFigure 3F Individual study result of primary outcome: subgroup of vaginal tumor**

**
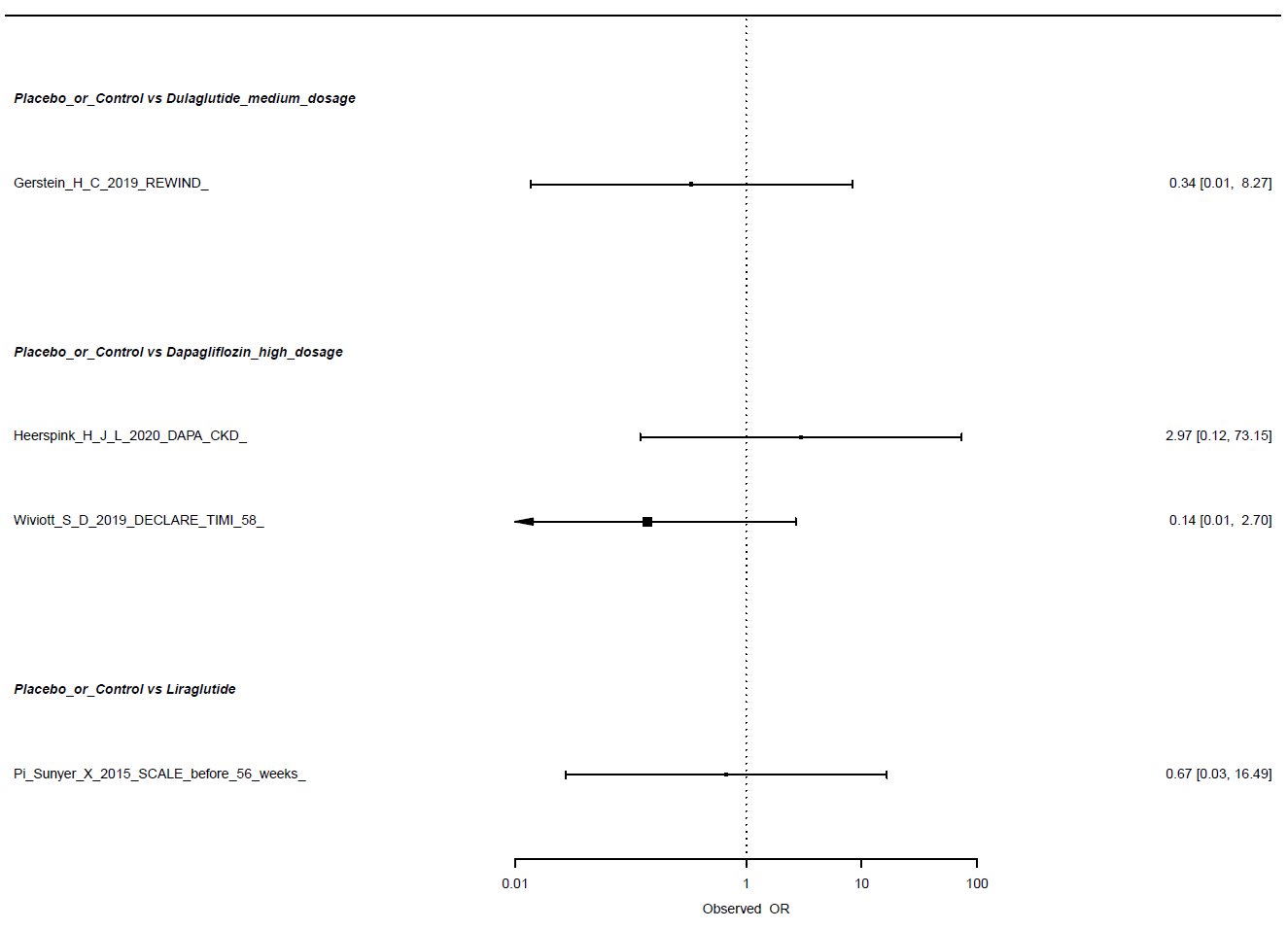
**

**eFigure 3G Individual study result of primary outcome: subgroup of vulvar tumor**

**
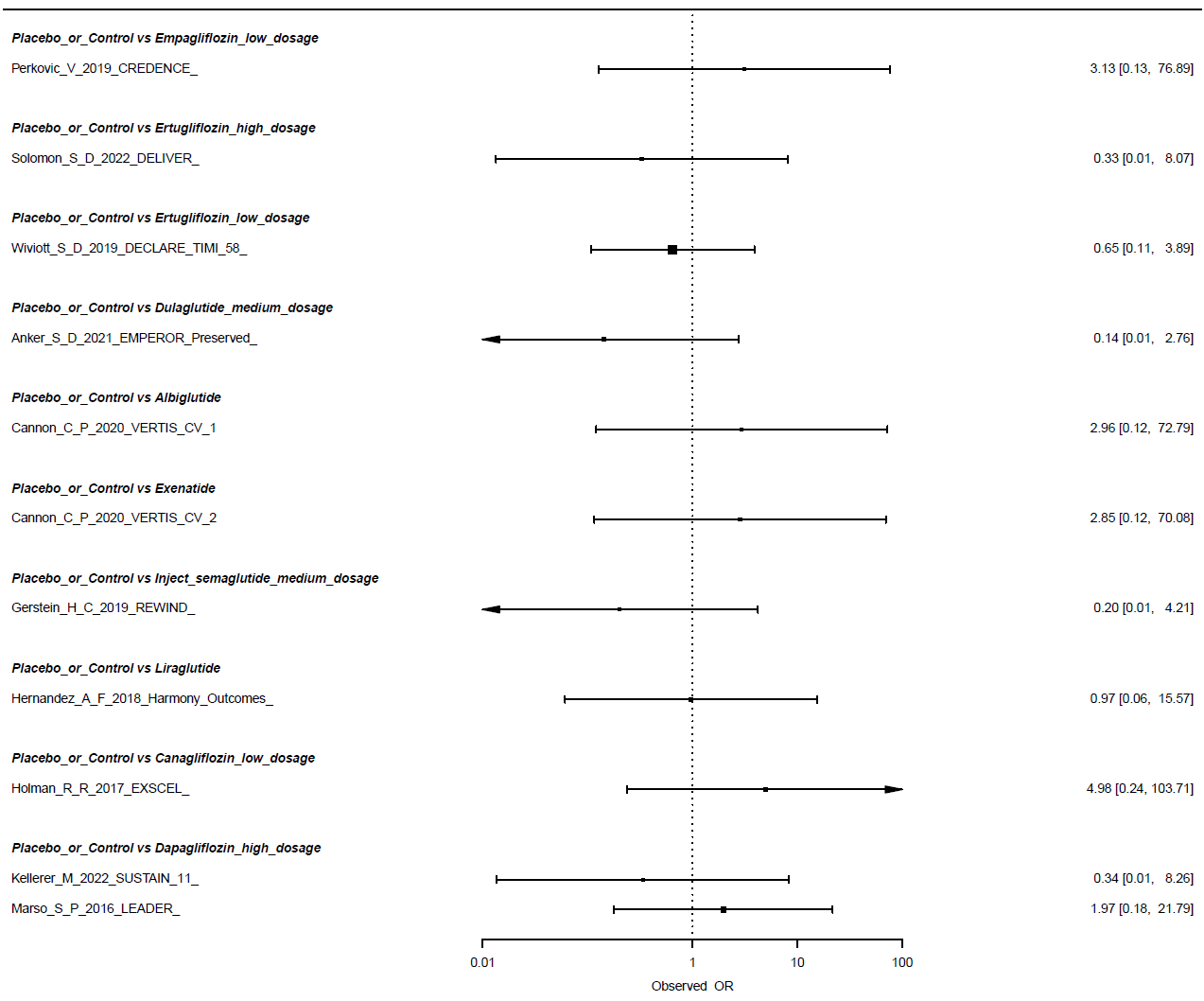
**

**eFigure 3H Individual study result of acceptability: drop-out rate***

**
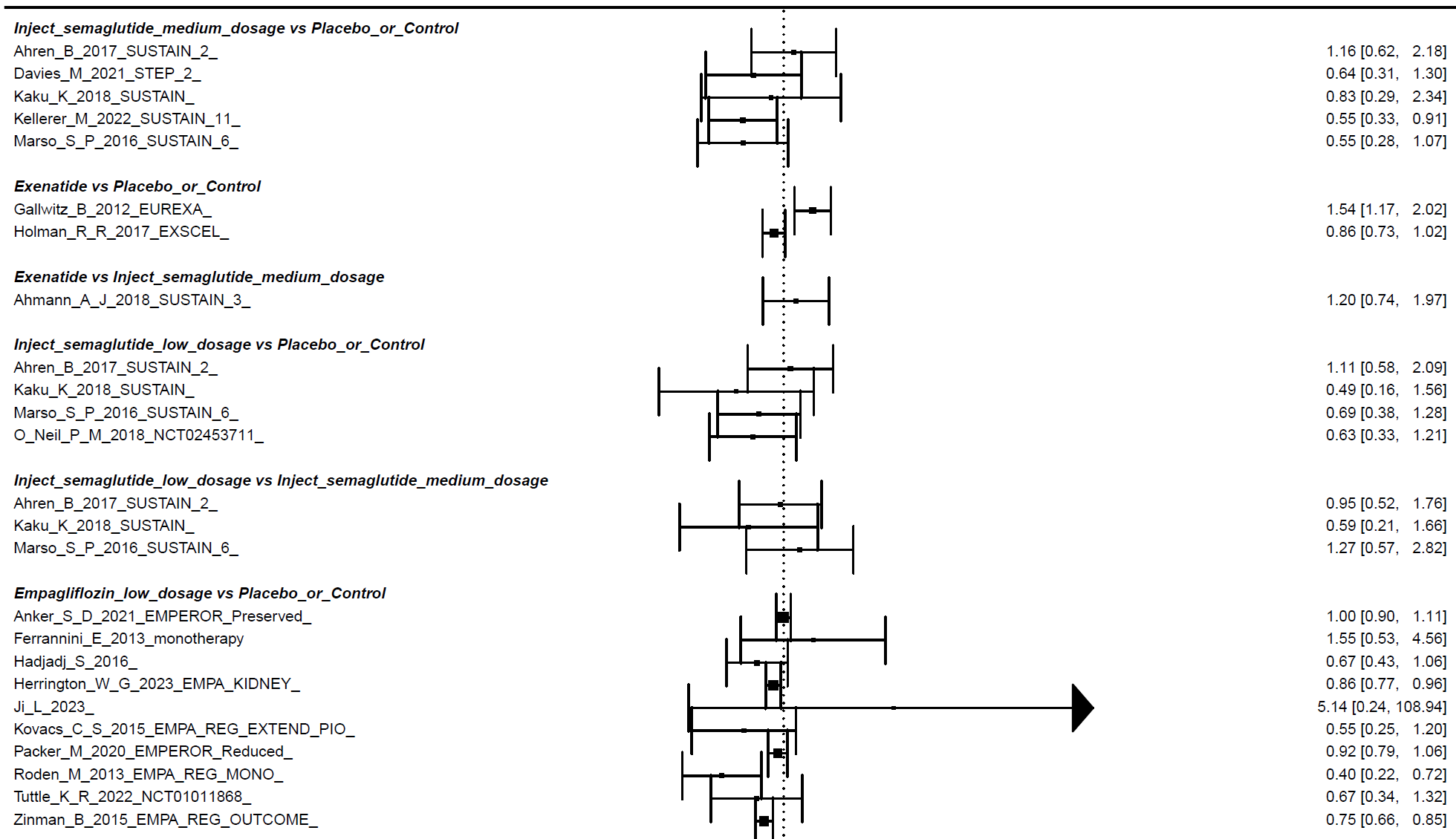
**

**
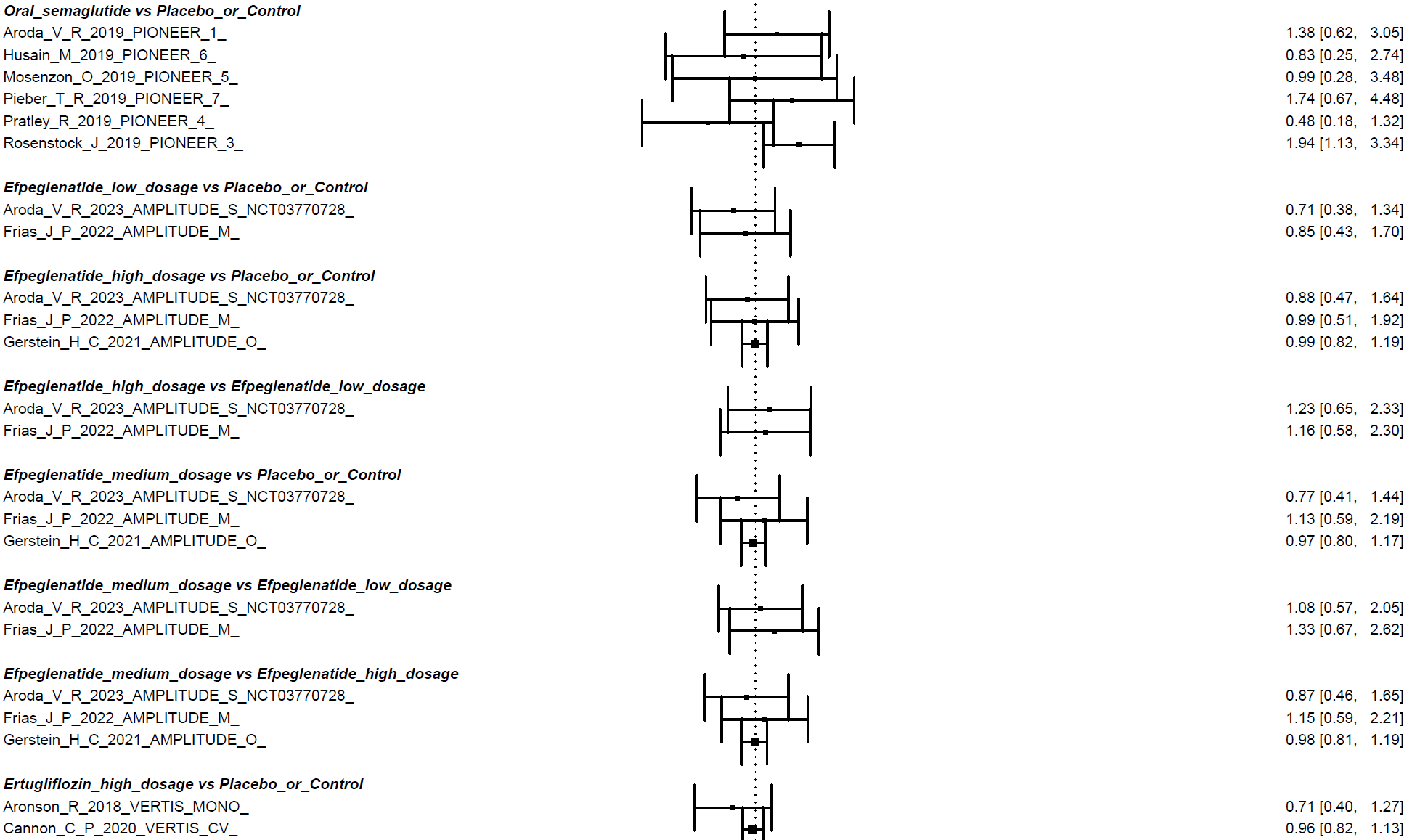
**

**
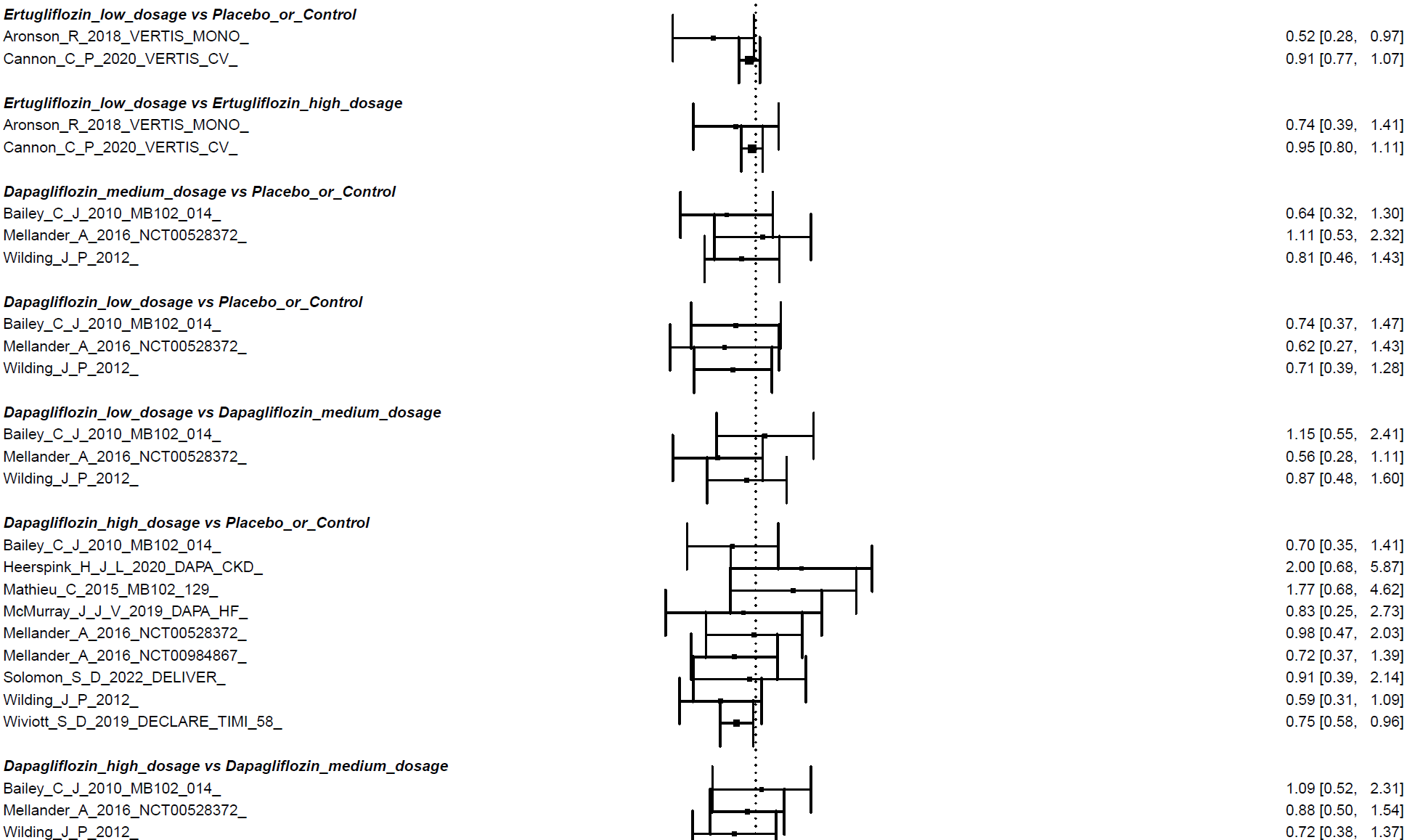
**

**
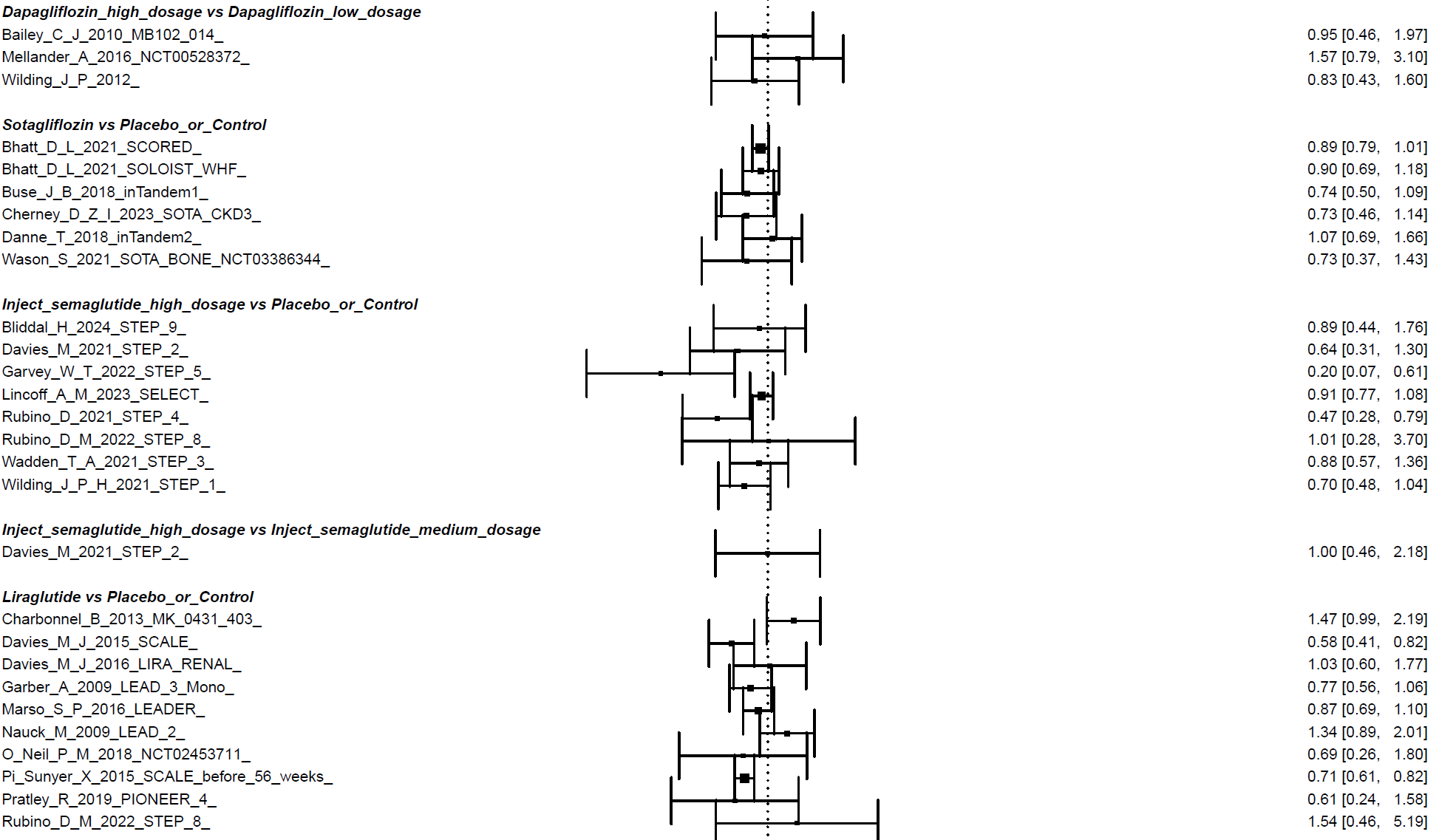
**

**
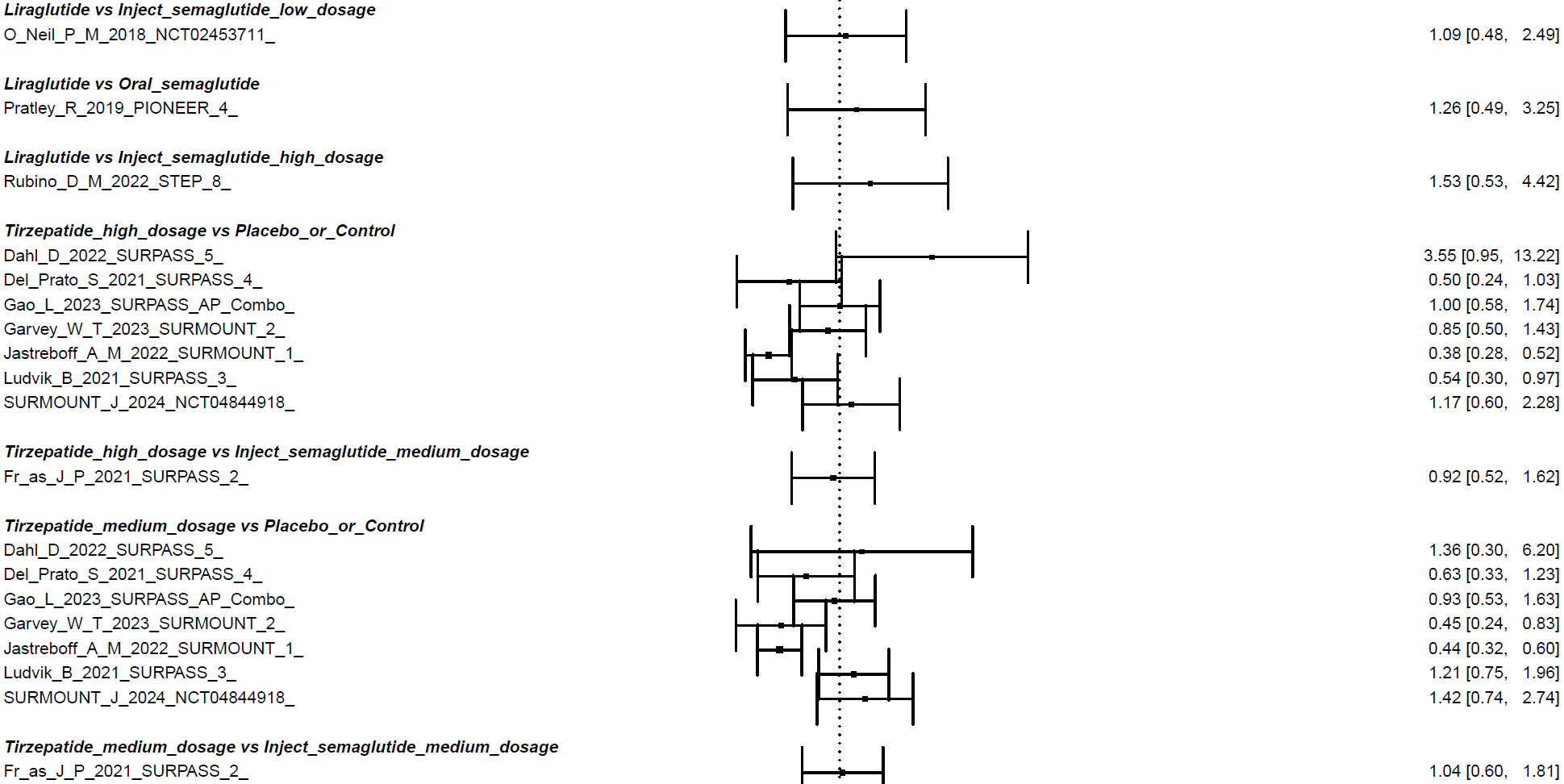
**

**
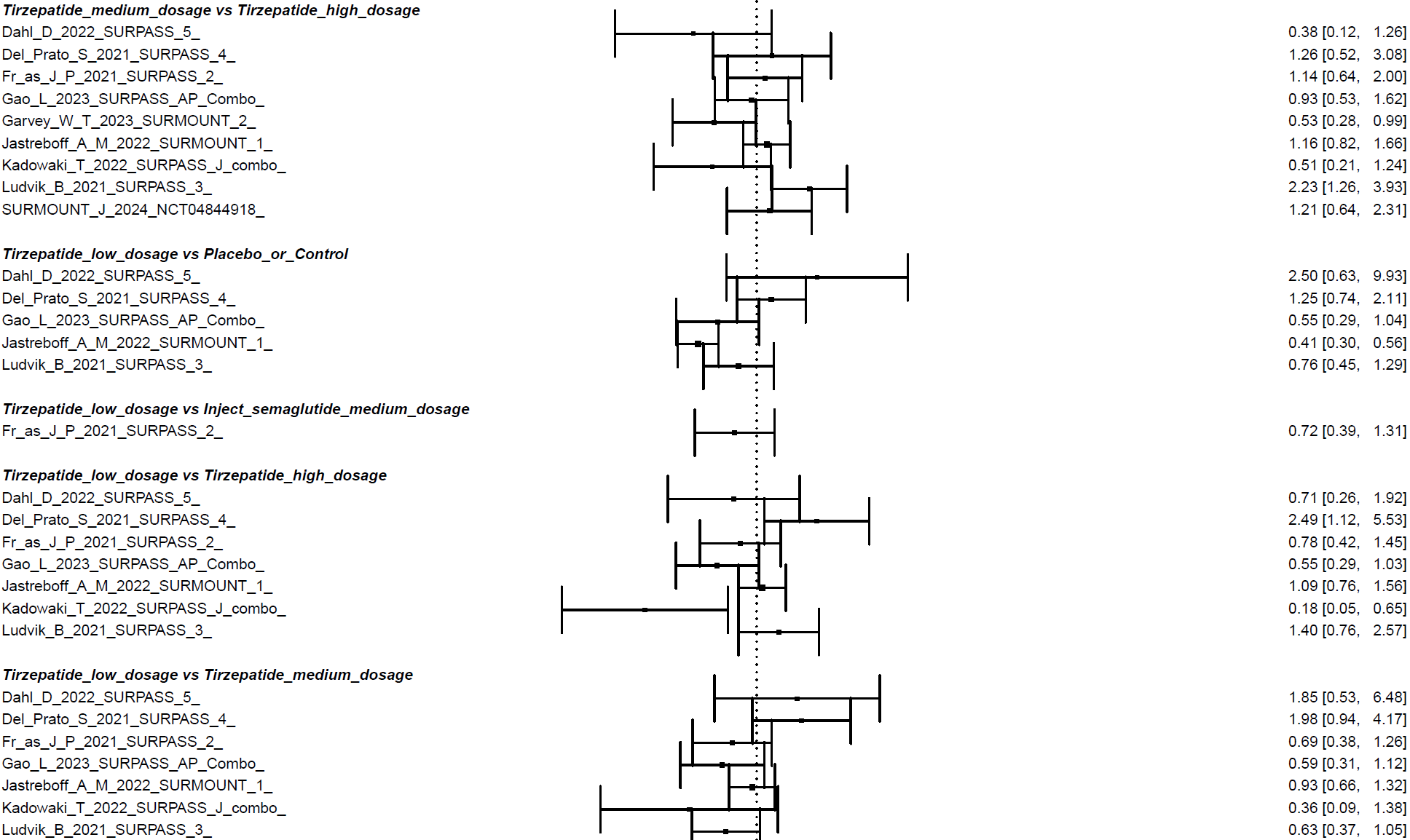
**

**
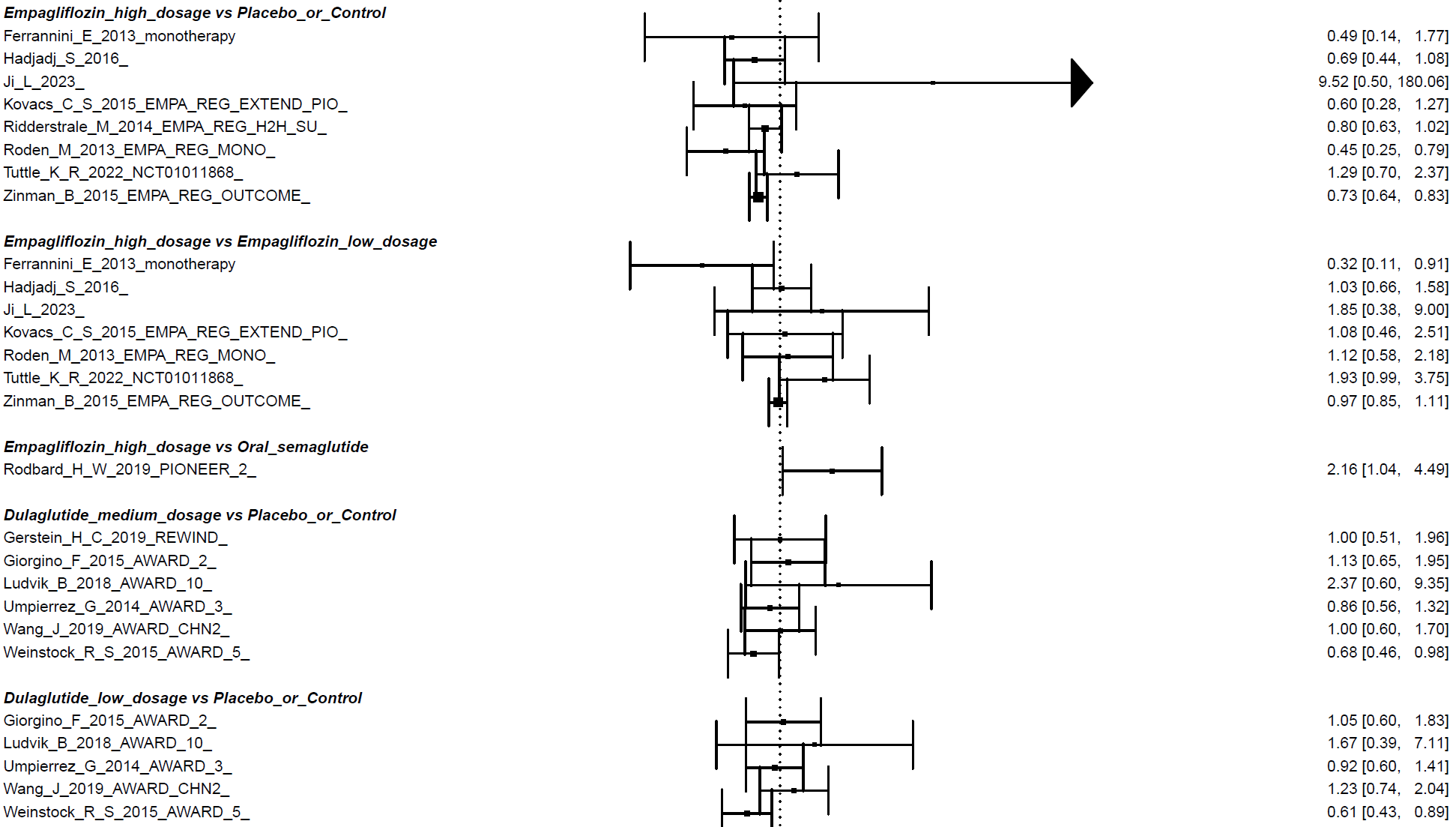
**

**
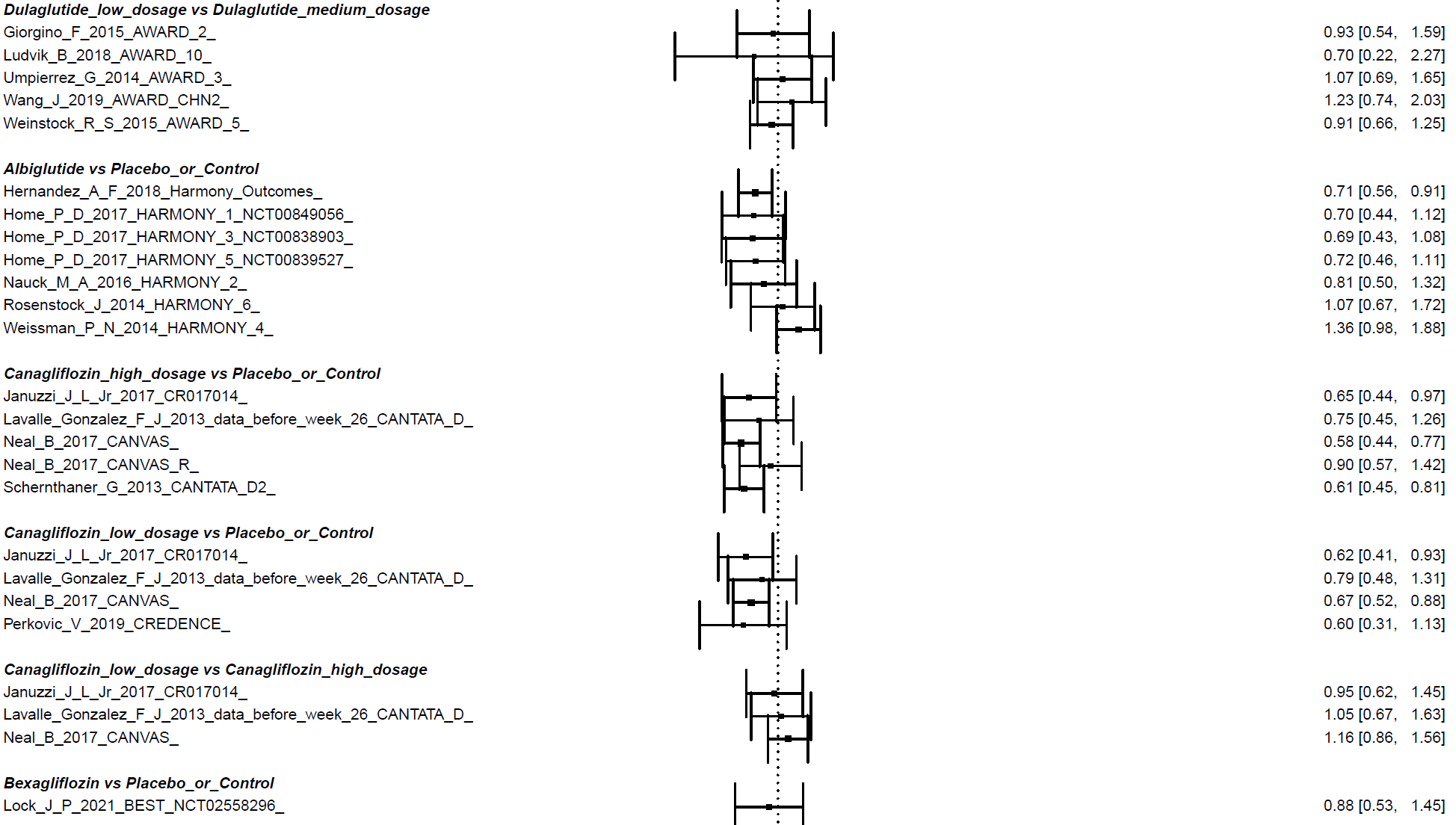
**

**
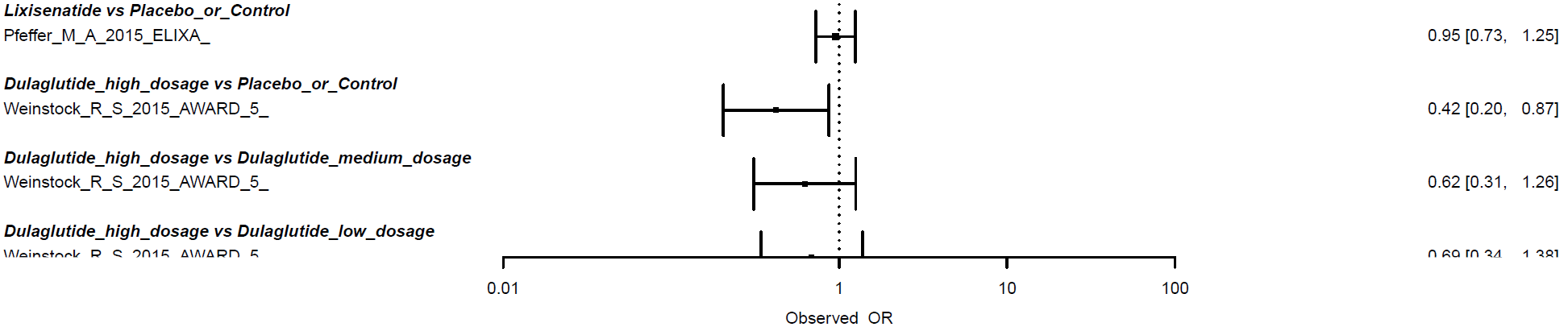
**

**Figure legend of eFigure 3H**

* The outcome of drop-out rate here was calculated according to drop-out rate data from the original composition of subjects from included studies because there were no any studies provided specific information regarding drop-out rate data in female subgroup.

***Abbreviation for eFigure 3A-3H:***

*95%CIs: 95% confidence intervals; GLP-1 agonist: glucagon-like peptide-1 agonist; NMA: network meta-analysis; OR: odds ratio; RCT: randomized controlled trial; SGLT2 inhibitor: sodium–glucose cotransporter 2 inhibitor*

**eFigure 4A Bayesian-based forest plot of NMA of primary outcome: overall gynecologic tumor**

**
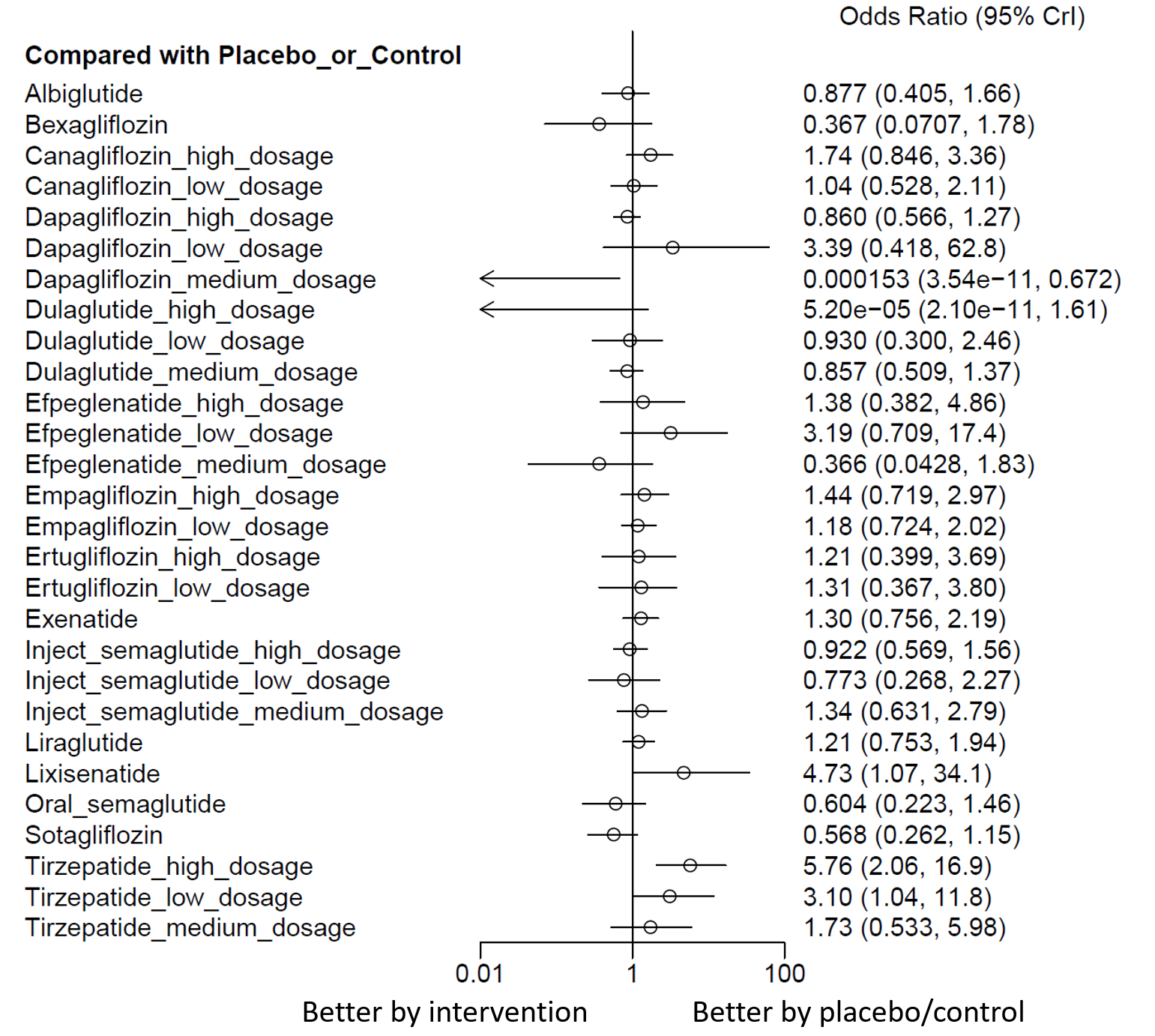
**

**eFigure 4B Bayesian-based forest plot of NMA of primary outcome: subgroup of intra-uterus tumor**

**
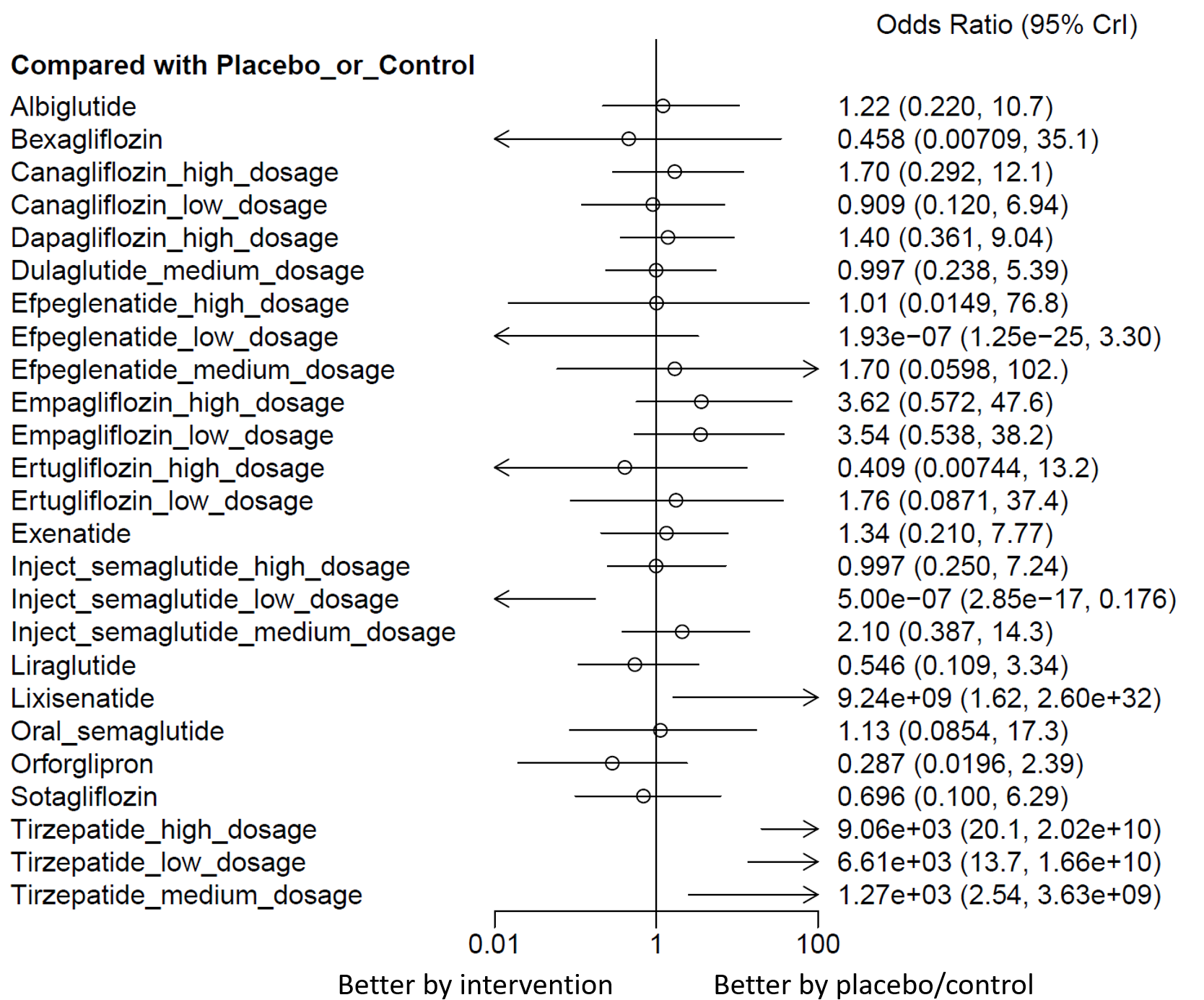
**

**eFigure 4C Bayesian-based forest plot of NMA of primary outcome: subgroup of cervical tumor**

**
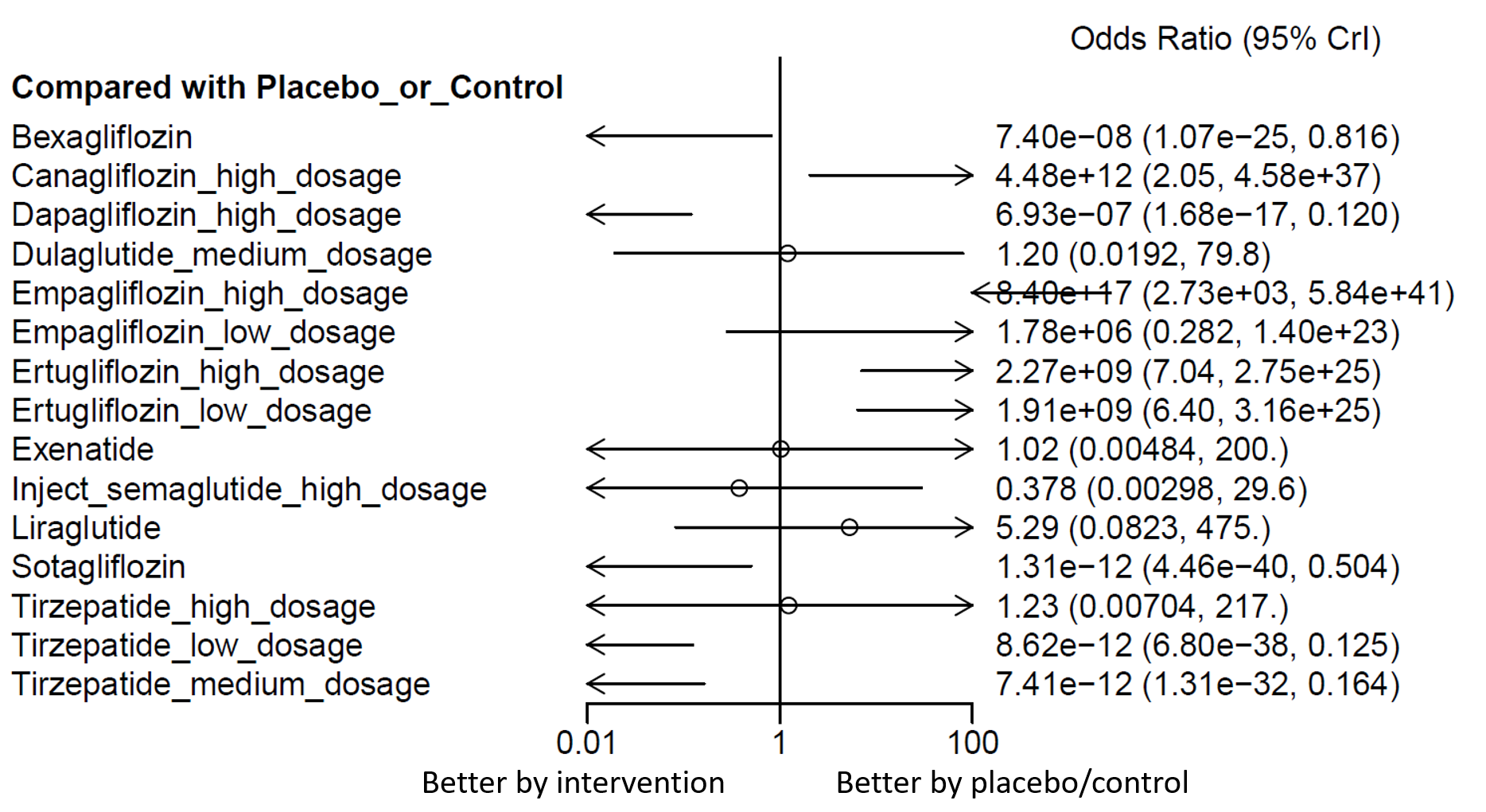
**

**eFigure 4D Bayesian-based forest plot of NMA of primary outcome: subgroup of ovarian tumor**

**
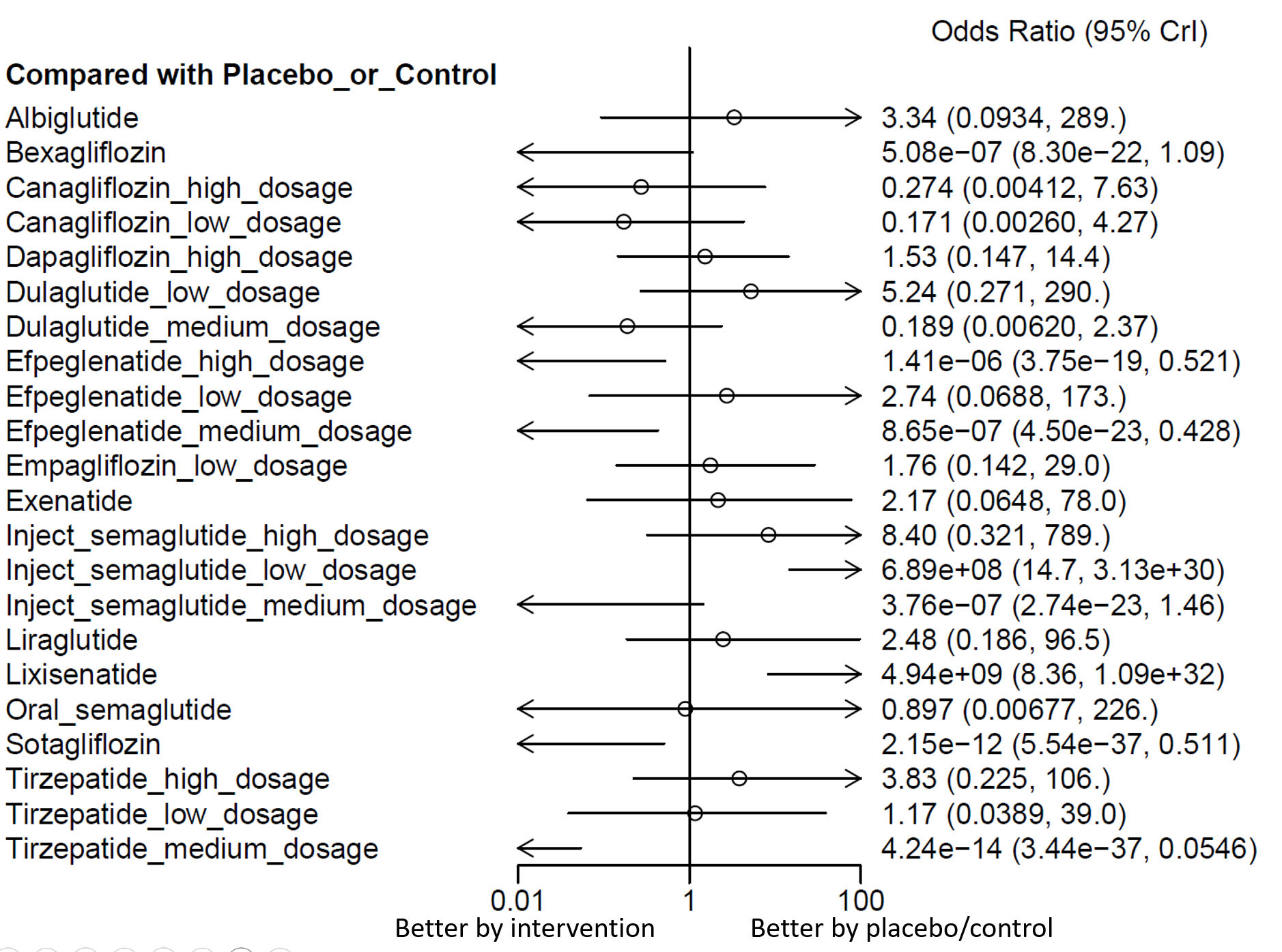
**

**eFigure 4E Bayesian-based forest plot of NMA of primary outcome: subgroup of breast tumor**

**
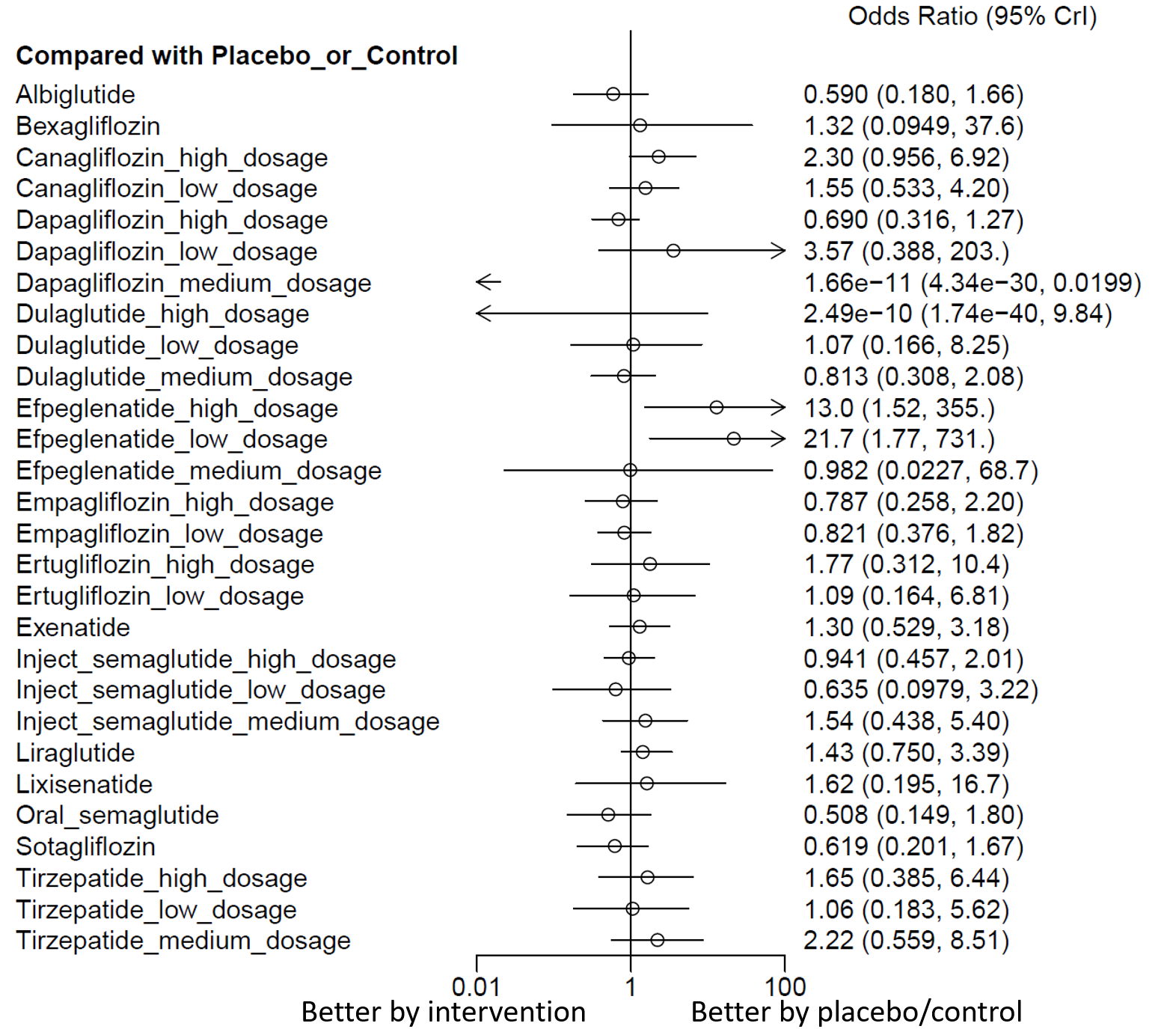
**

**eFigure 4F Bayesian-based forest plot of NMA of primary outcome: subgroup of vaginal tumor**

**
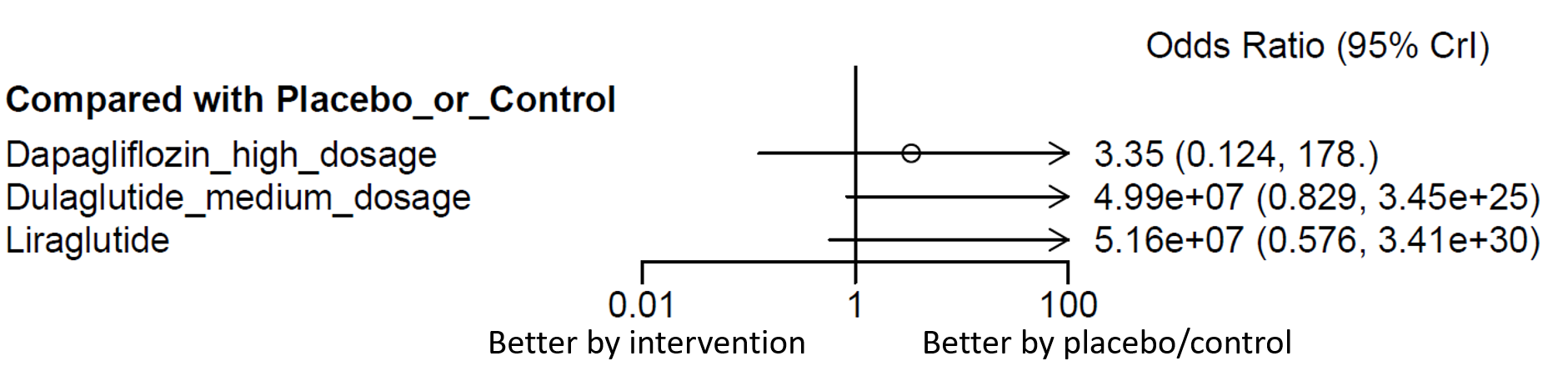
**

**eFigure 4G Bayesian-based forest plot of NMA of primary outcome: subgroup of vulvar tumor**

**
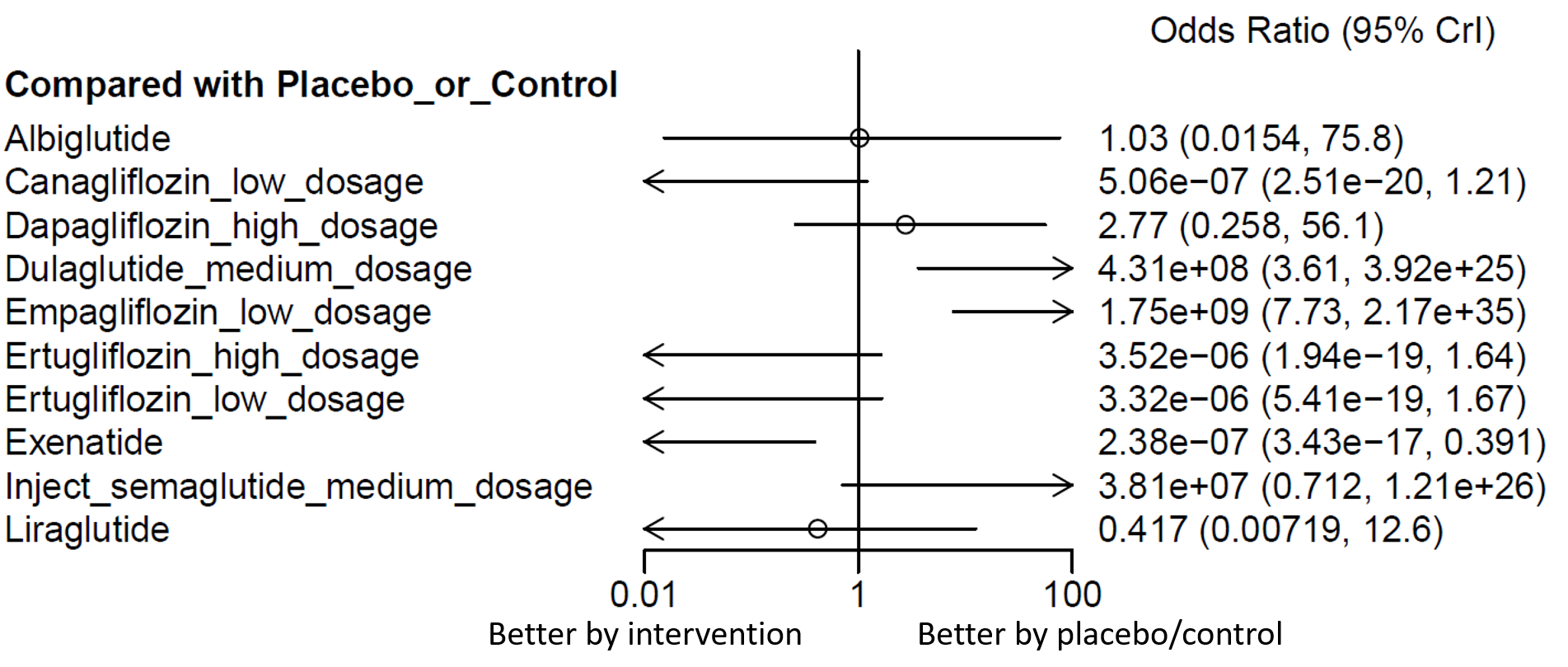
**

**eFigure 4H Bayesian-based forest plot of NMA of acceptability: drop-out rate***

**
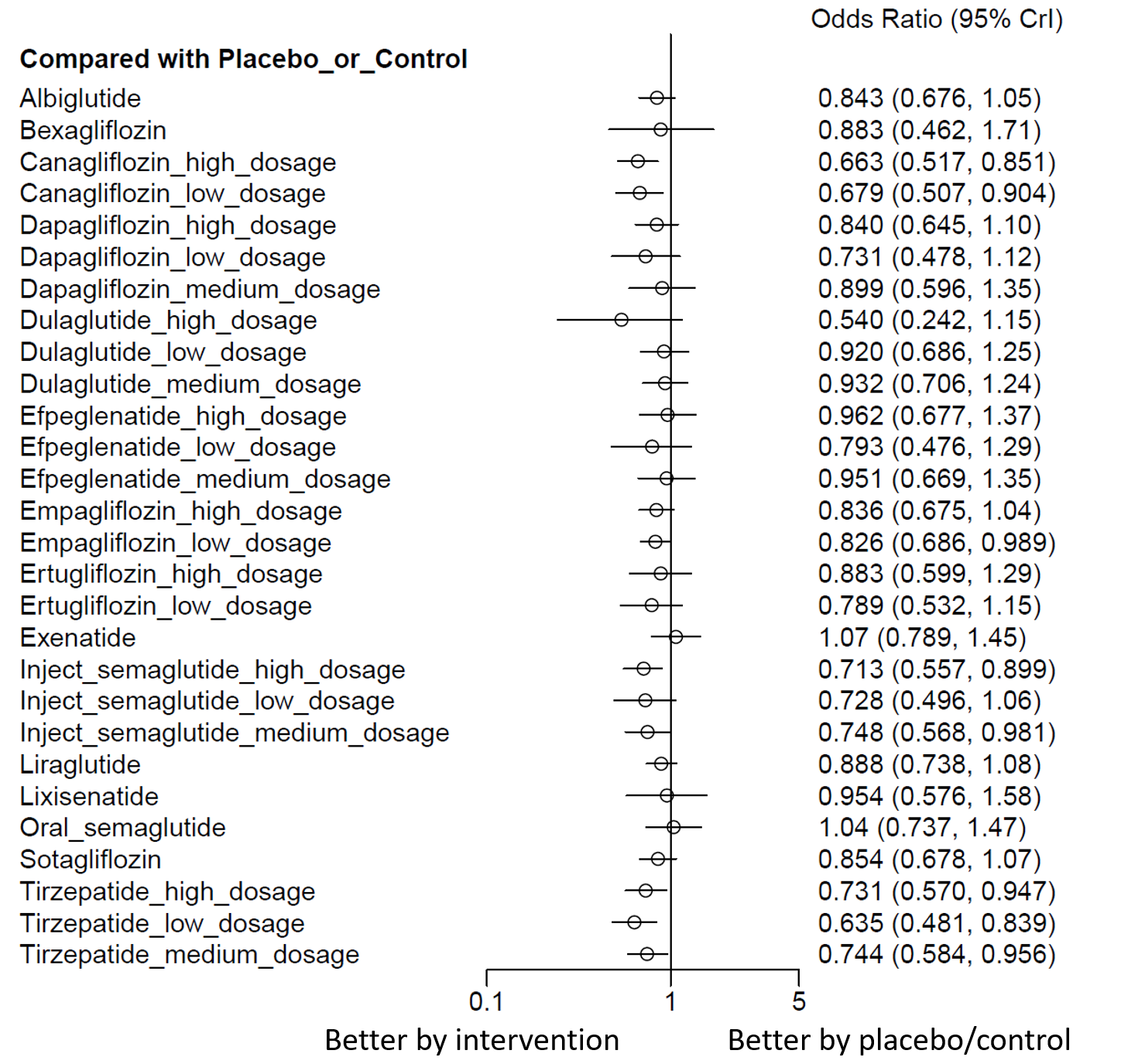
**

**Figure legend of eFigure 4H**

* The outcome of drop-out rate here was calculated according to drop-out rate data from the original composition of subjects from included studies because there were no any studies provided specific information regarding drop-out rate data in female subgroup.

***Abbreviation for eFigure 4A-4H:***

*95%CIs: 95% confidence intervals; GLP-1 agonist: glucagon-like peptide-1 agonist; NMA: network meta-analysis; OR: odds ratio; RCT: randomized controlled trial; SGLT2 inhibitor: sodium–glucose cotransporter 2 inhibitor*

**eFigure 5A Bayesian-based Litmus Rank-O-Gram rank plot of primary outcome: overall gynecologic tumor**

**
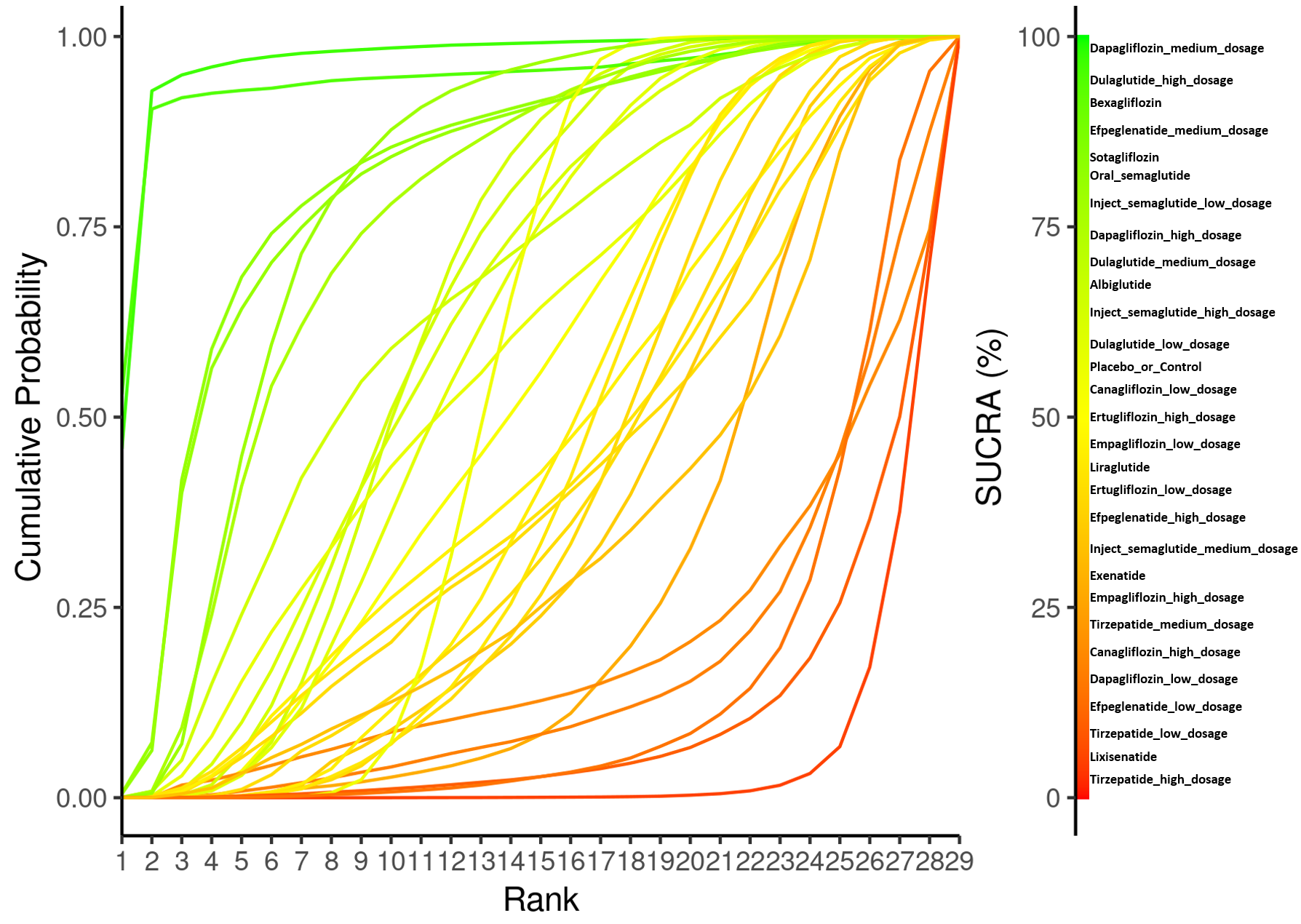
**

**eFigure 5B Bayesian-based radial surface under the cumulative ranking of primary outcome: overall gynecologic tumor**

**
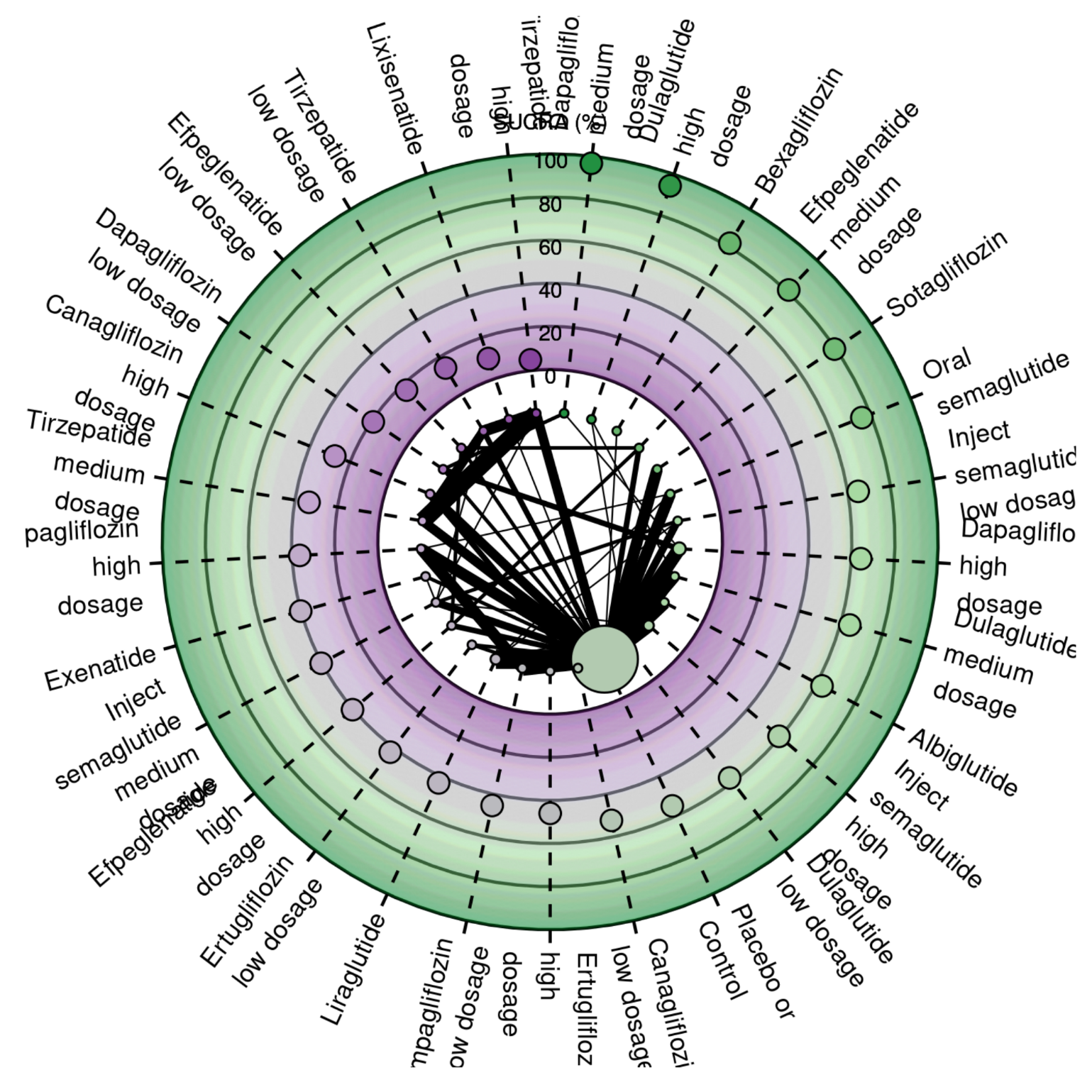
**

**eFigure 5C Bayesian-based Litmus Rank-O-Gram rank plot of primary outcome: subgroup of intra-uterus tumor**

**
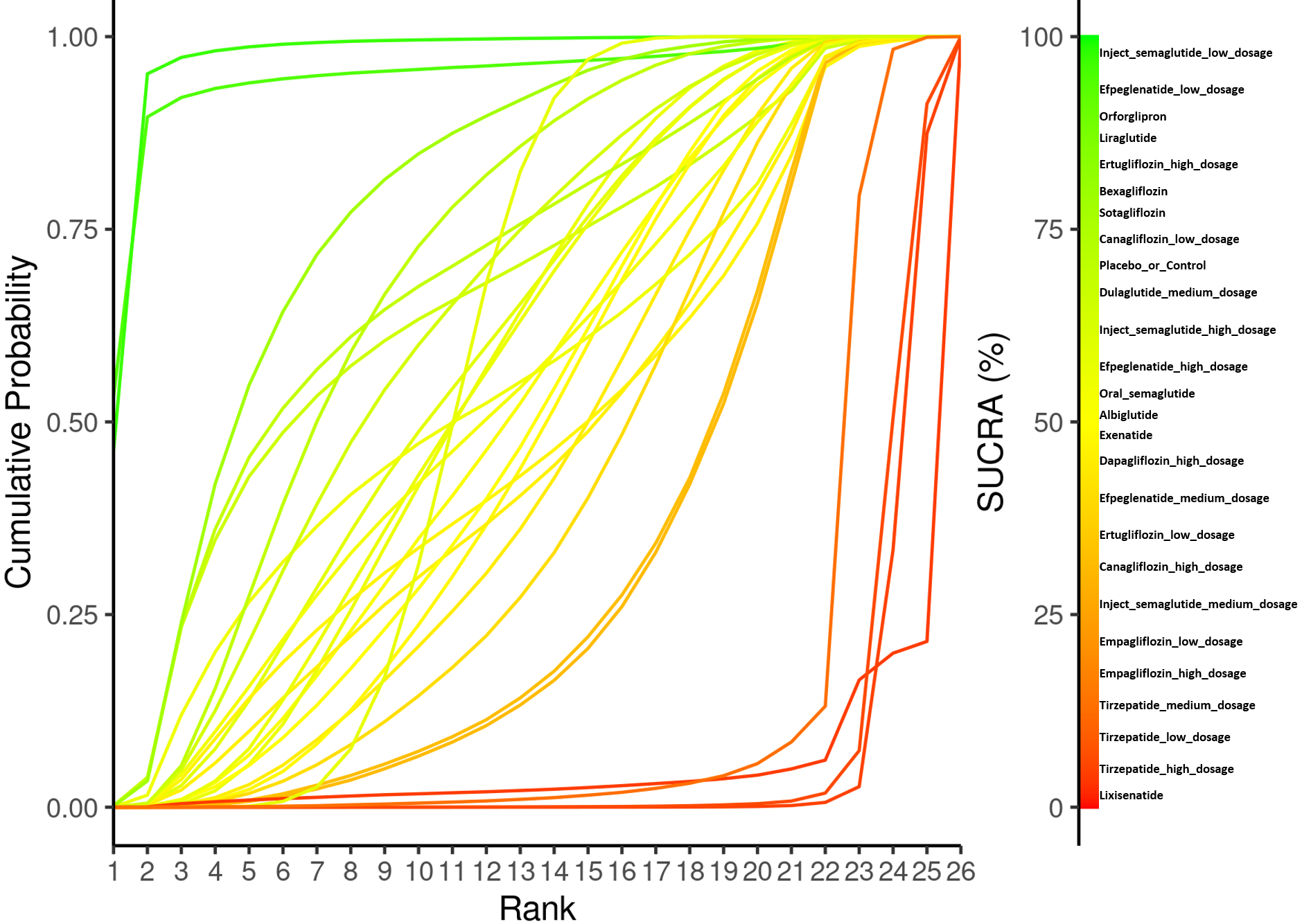
**

**eFigure 5D Bayesian-based radial surface under the cumulative ranking of primary outcome: subgroup of intra-uterus tumor**

**
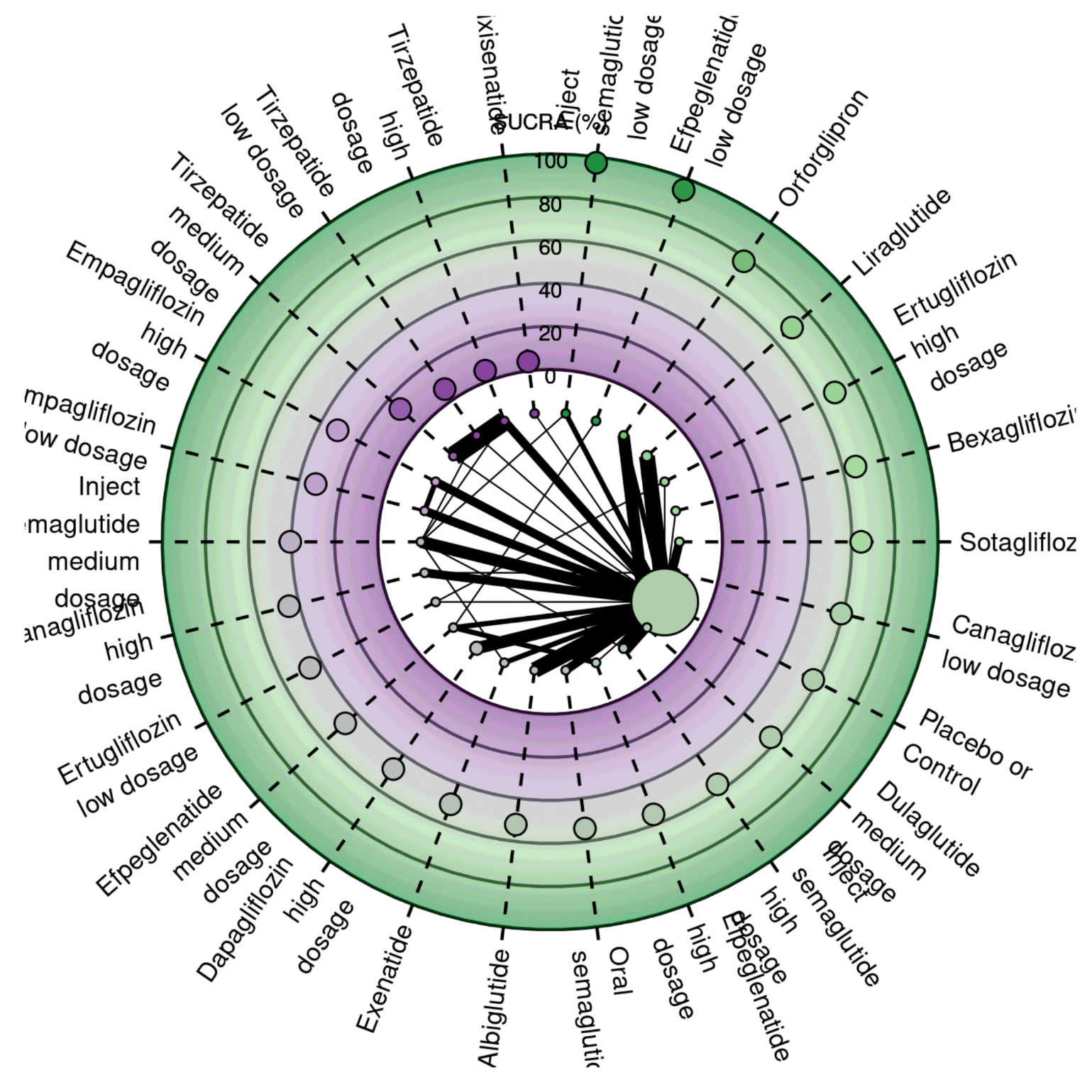
**

**eFigure 5E Bayesian-based Litmus Rank-O-Gram rank plot of primary outcome: subgroup of cervical tumor**

**
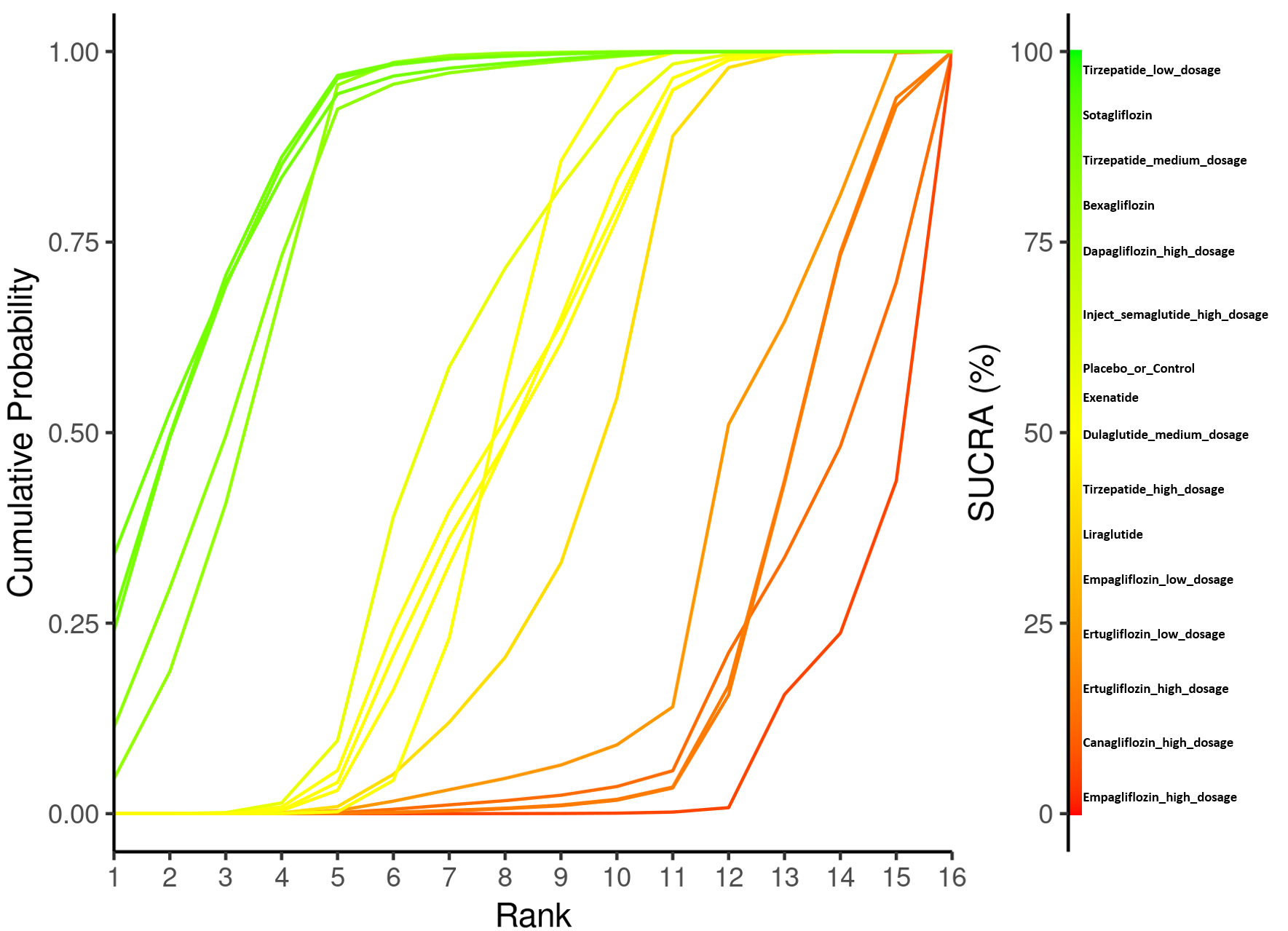
**

**eFigure 5F Bayesian-based radial surface under the cumulative ranking of primary outcome: subgroup of endometrial tumor**

**
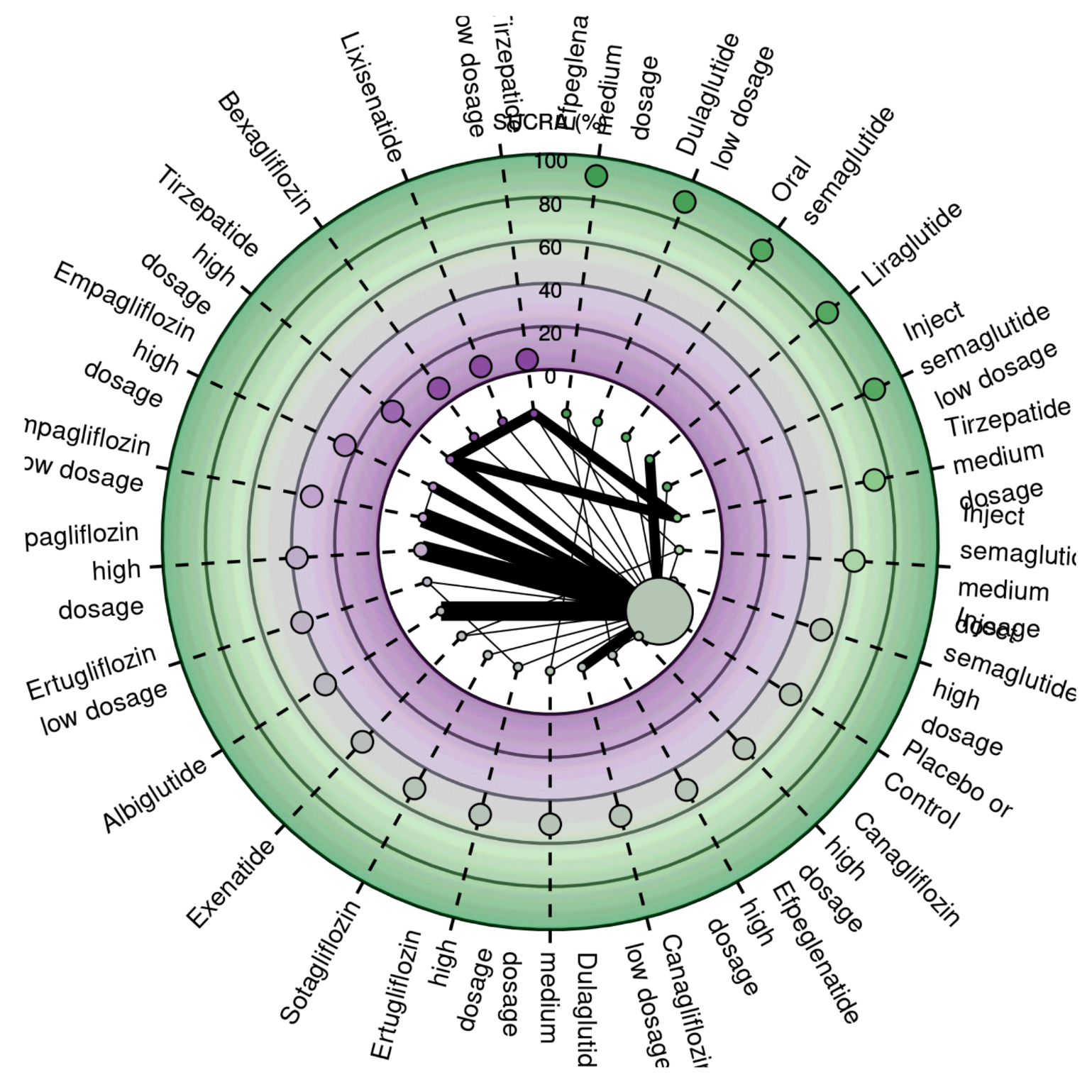
**

**eFigure 5G Bayesian-based Litmus Rank-O-Gram rank plot of primary outcome: subgroup of ovarian tumor**

**
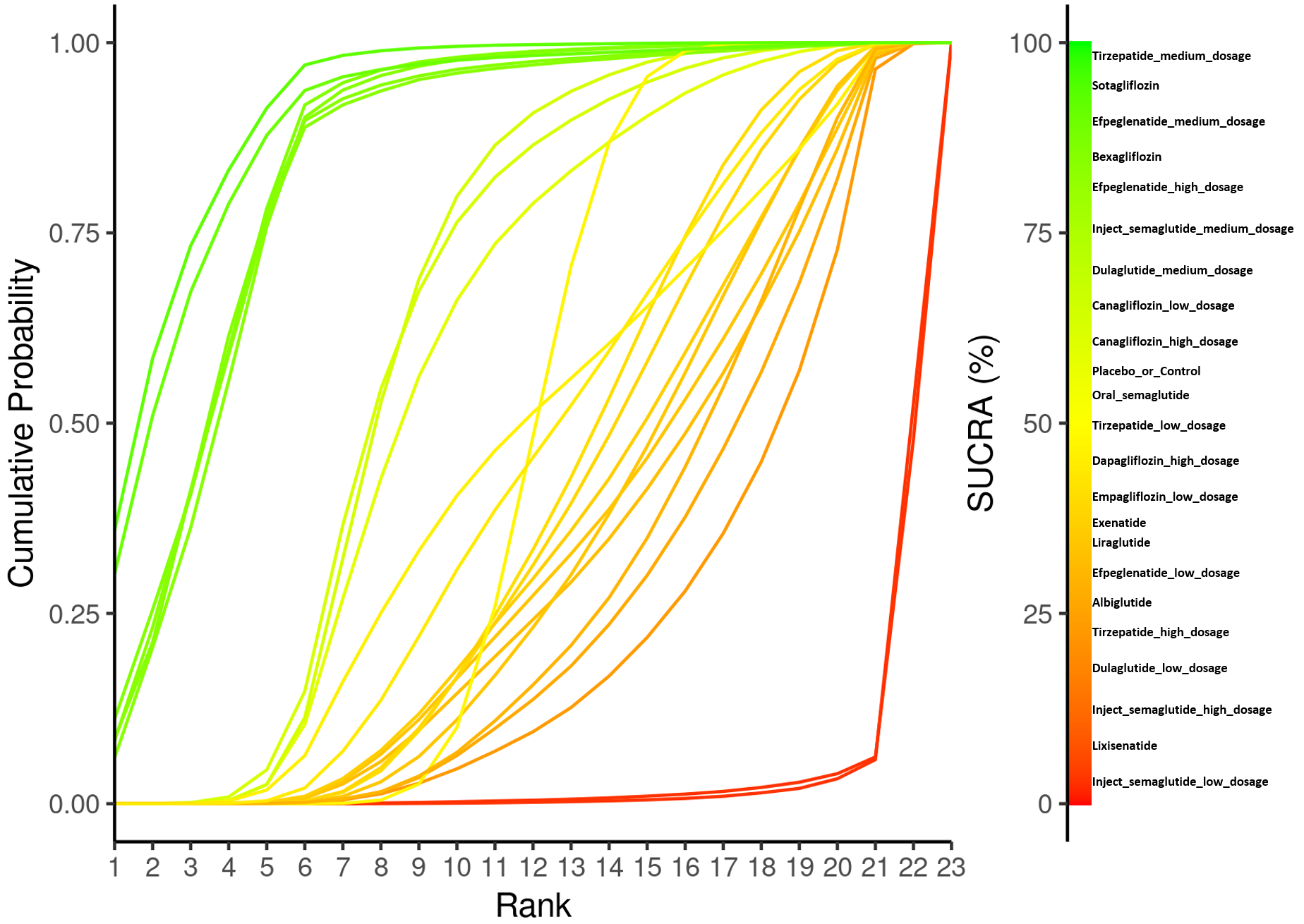
**

**eFigure 5H Bayesian-based radial surface under the cumulative ranking of primary outcome: subgroup of ovarian tumor**

**
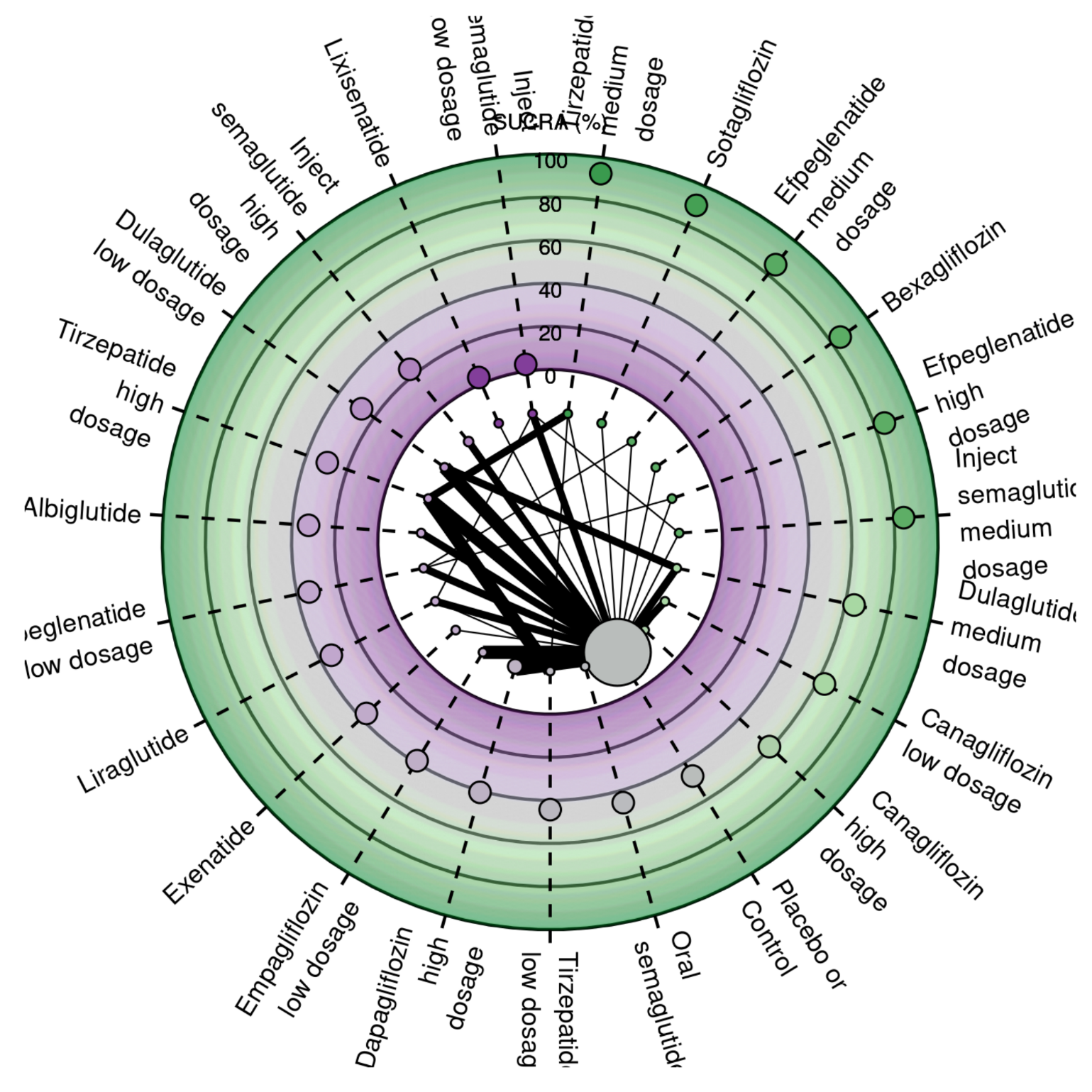
**

**eFigure 5I Bayesian-based Litmus Rank-O-Gram rank plot of primary outcome: subgroup of breast tumor**

**
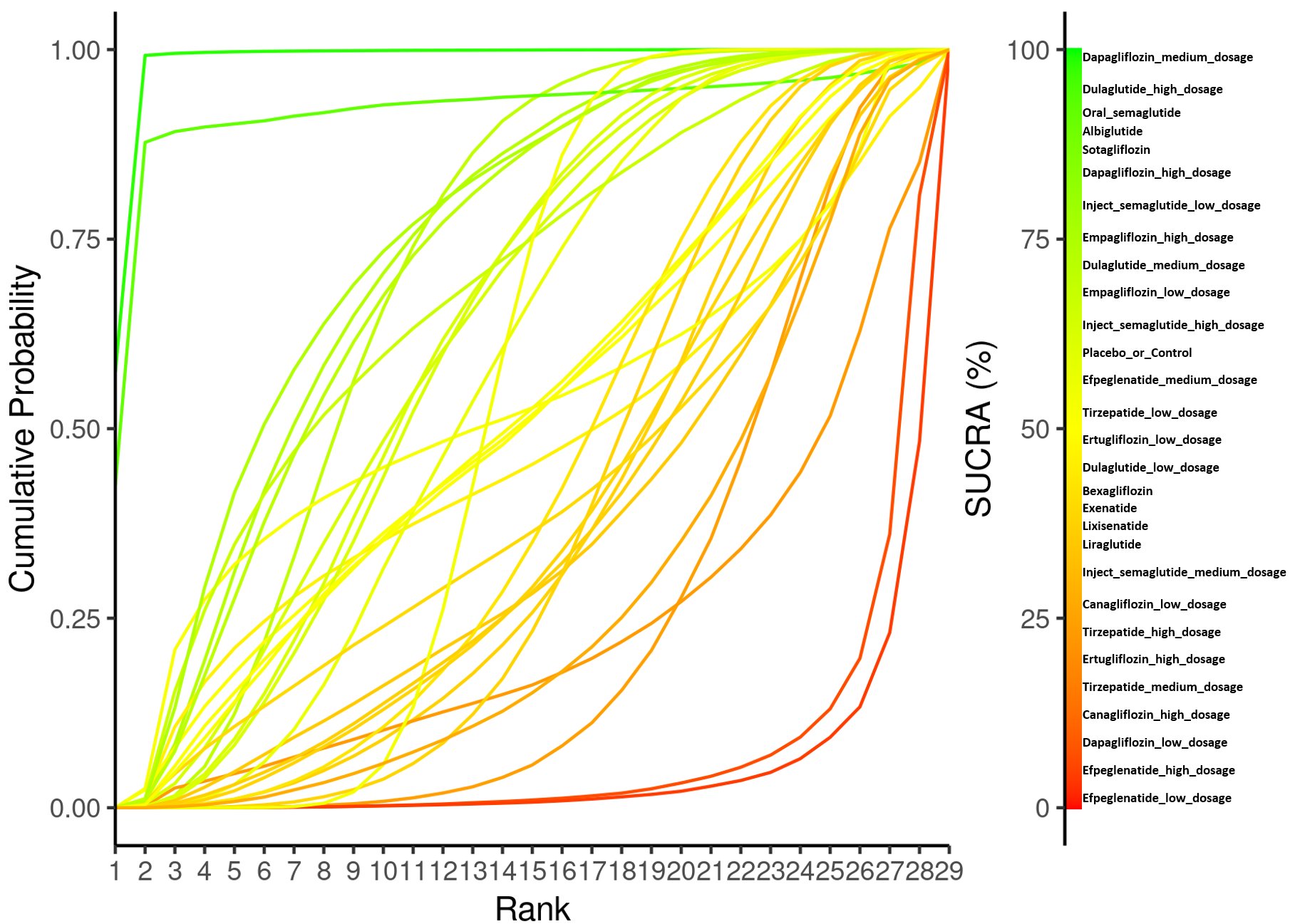
**

**eFigure 5J Bayesian-based radial surface under the cumulative ranking of primary outcome: subgroup of breast tumor**

**
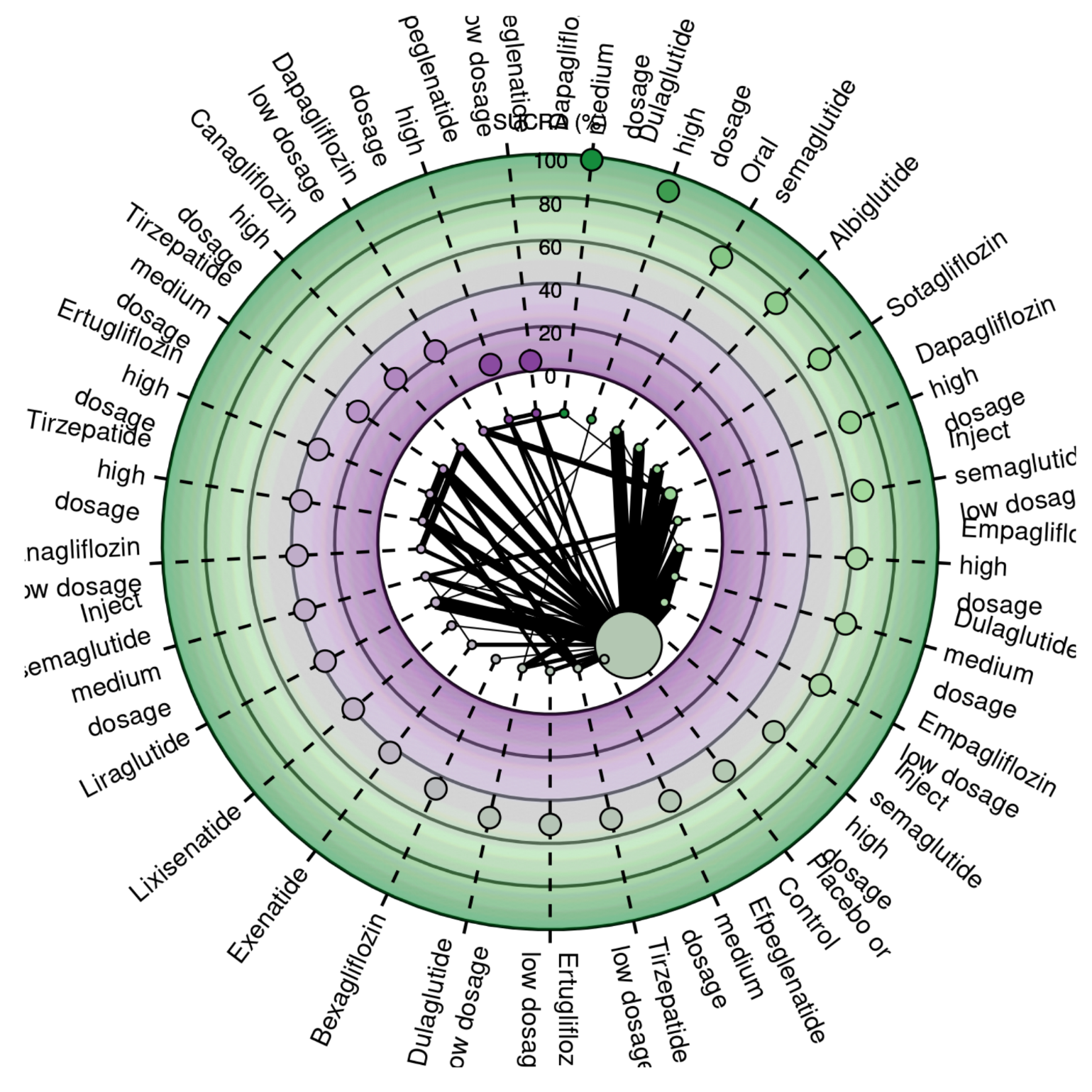
**

**eFigure 5K Bayesian-based Litmus Rank-O-Gram rank plot of primary outcome: subgroup of vaginal tumor**

**
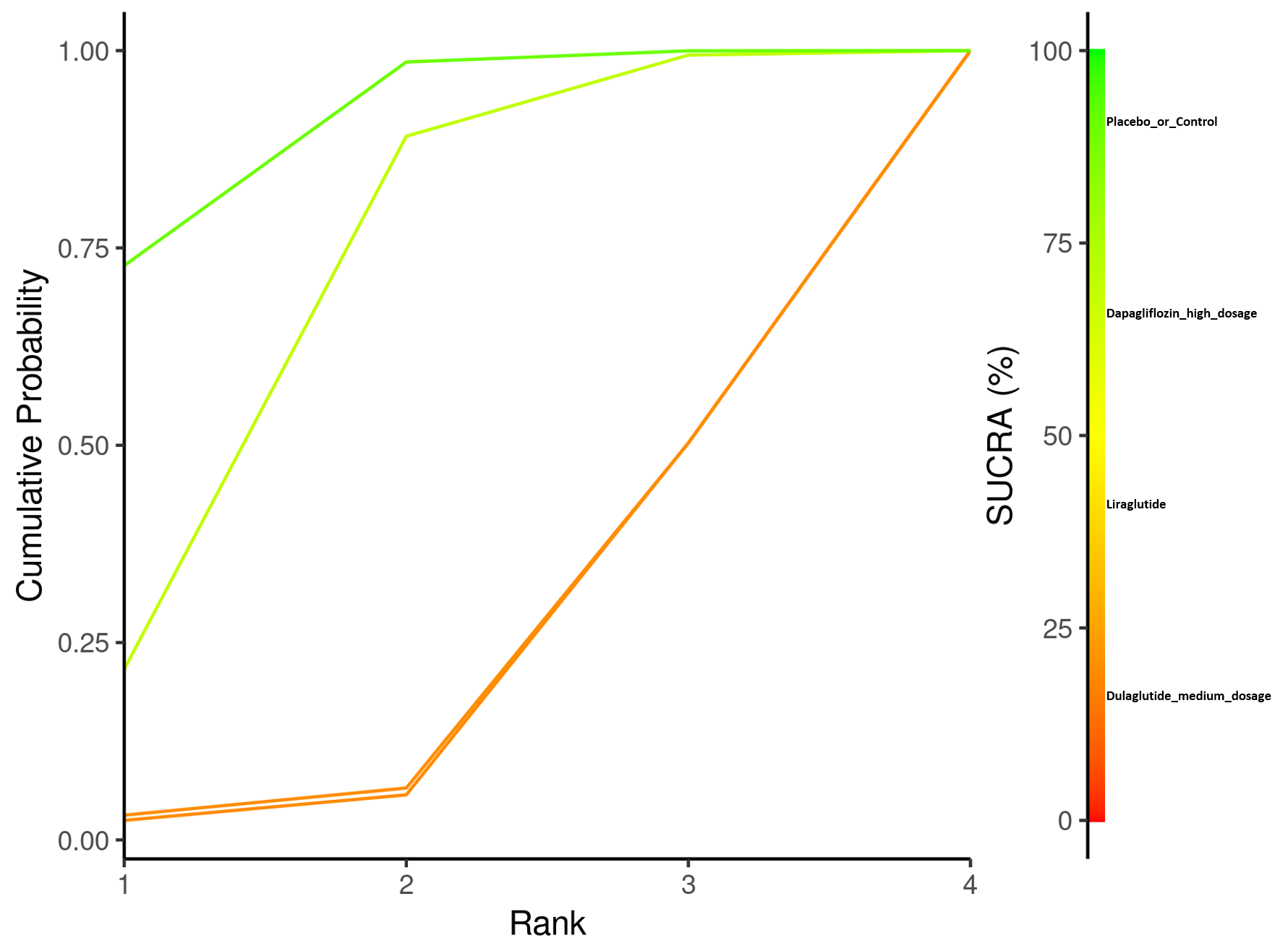
**

**eFigure 5L Bayesian-based radial surface under the cumulative ranking of primary outcome: subgroup of vaginal tumor**

**
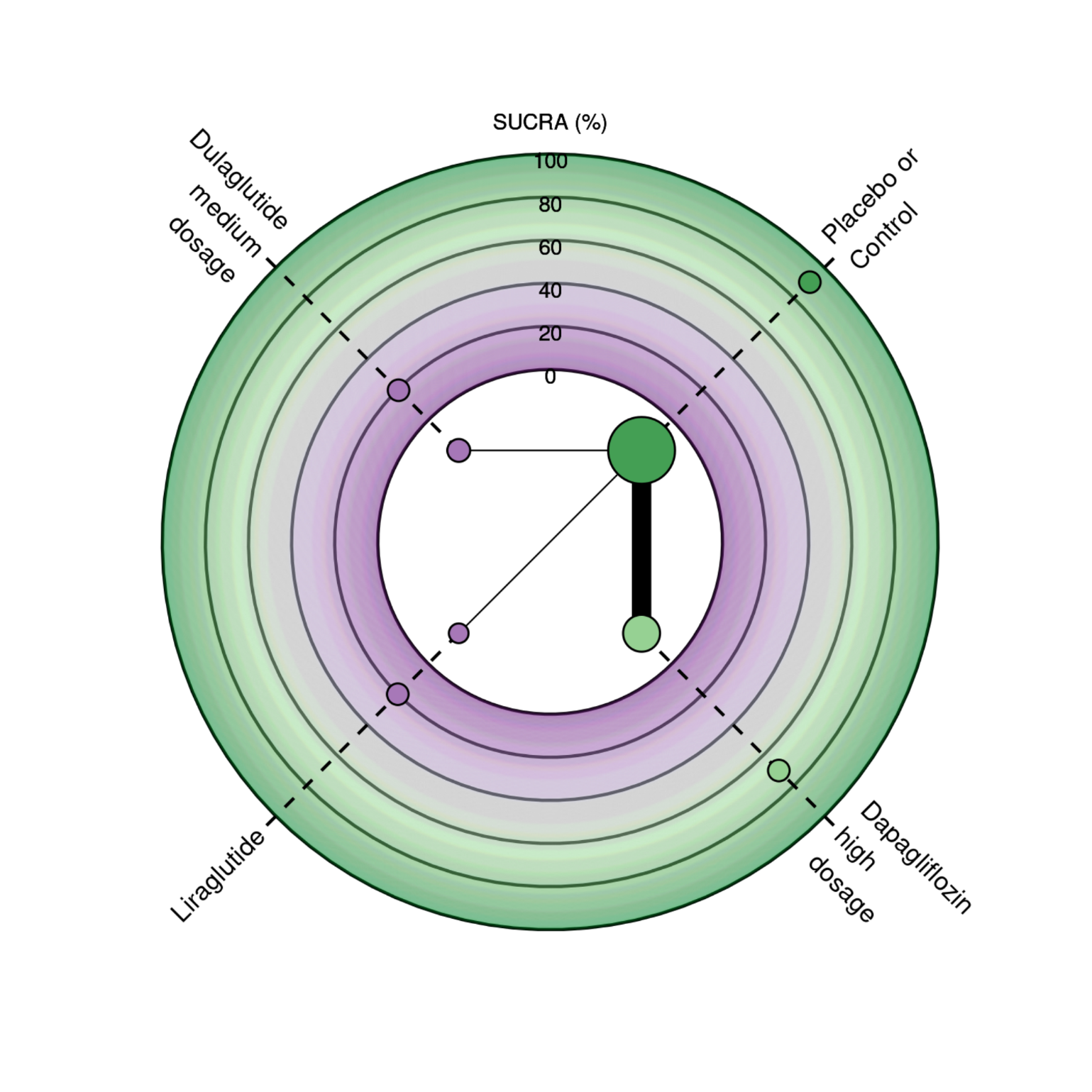
**

**eFigure 5M Bayesian-based Litmus Rank-O-Gram rank plot of primary outcome: subgroup of vulvar tumor**

**
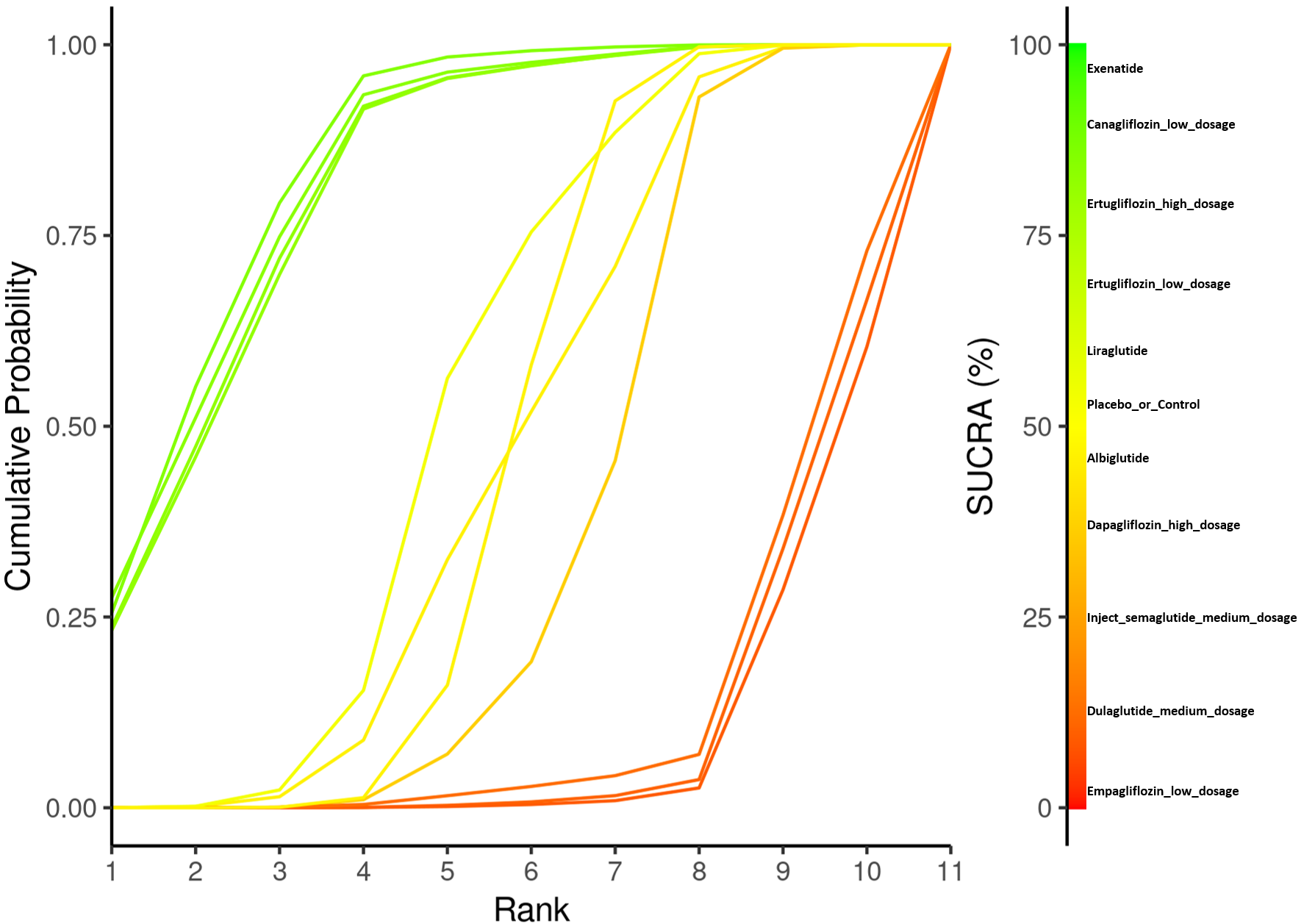
**

**eFigure 5N Bayesian-based radial surface under the cumulative ranking of primary outcome: subgroup of vulvar tumor**

**
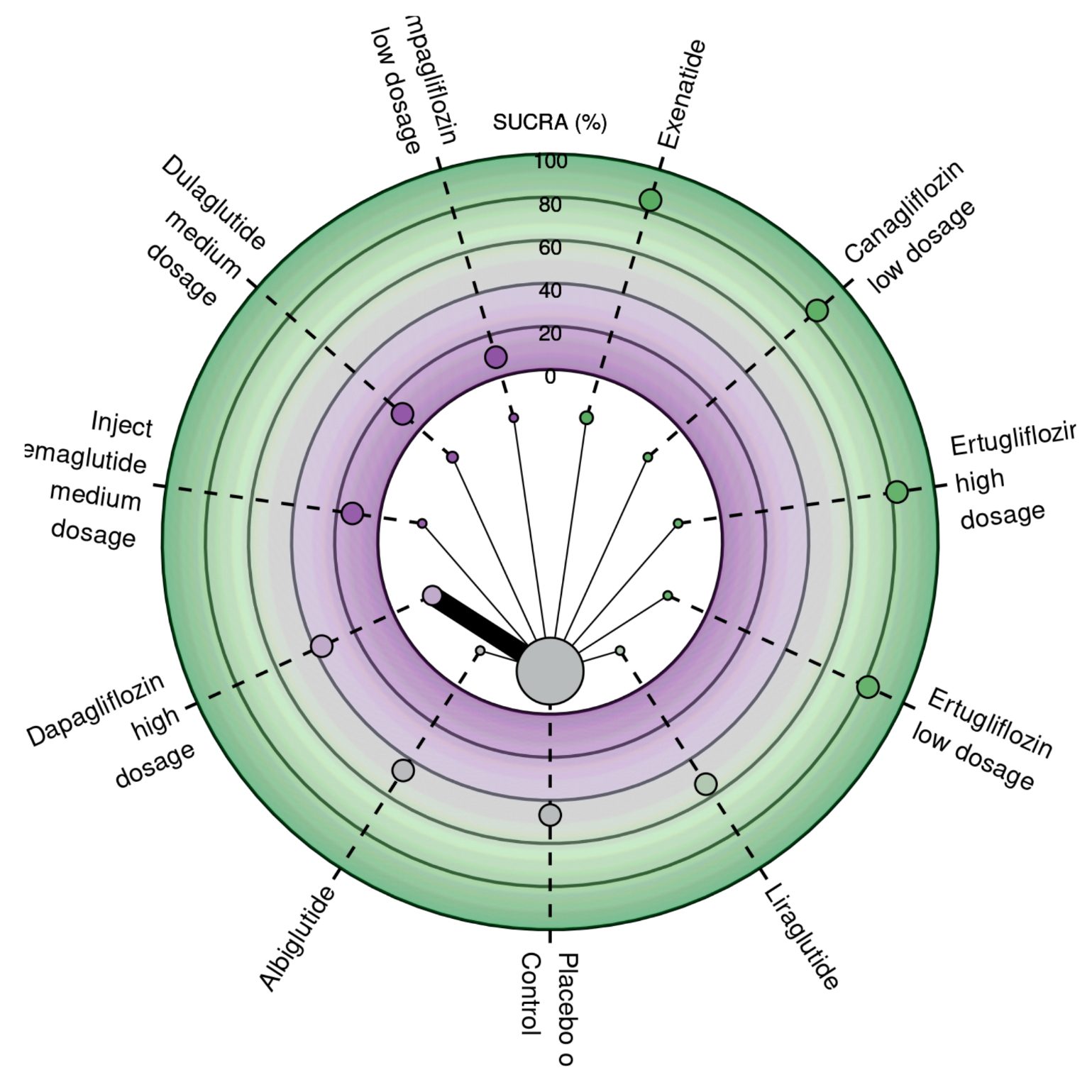
**

**eFigure 5O Bayesian-based Litmus Rank-O-Gram rank plot of acceptability: drop-out rate***

**
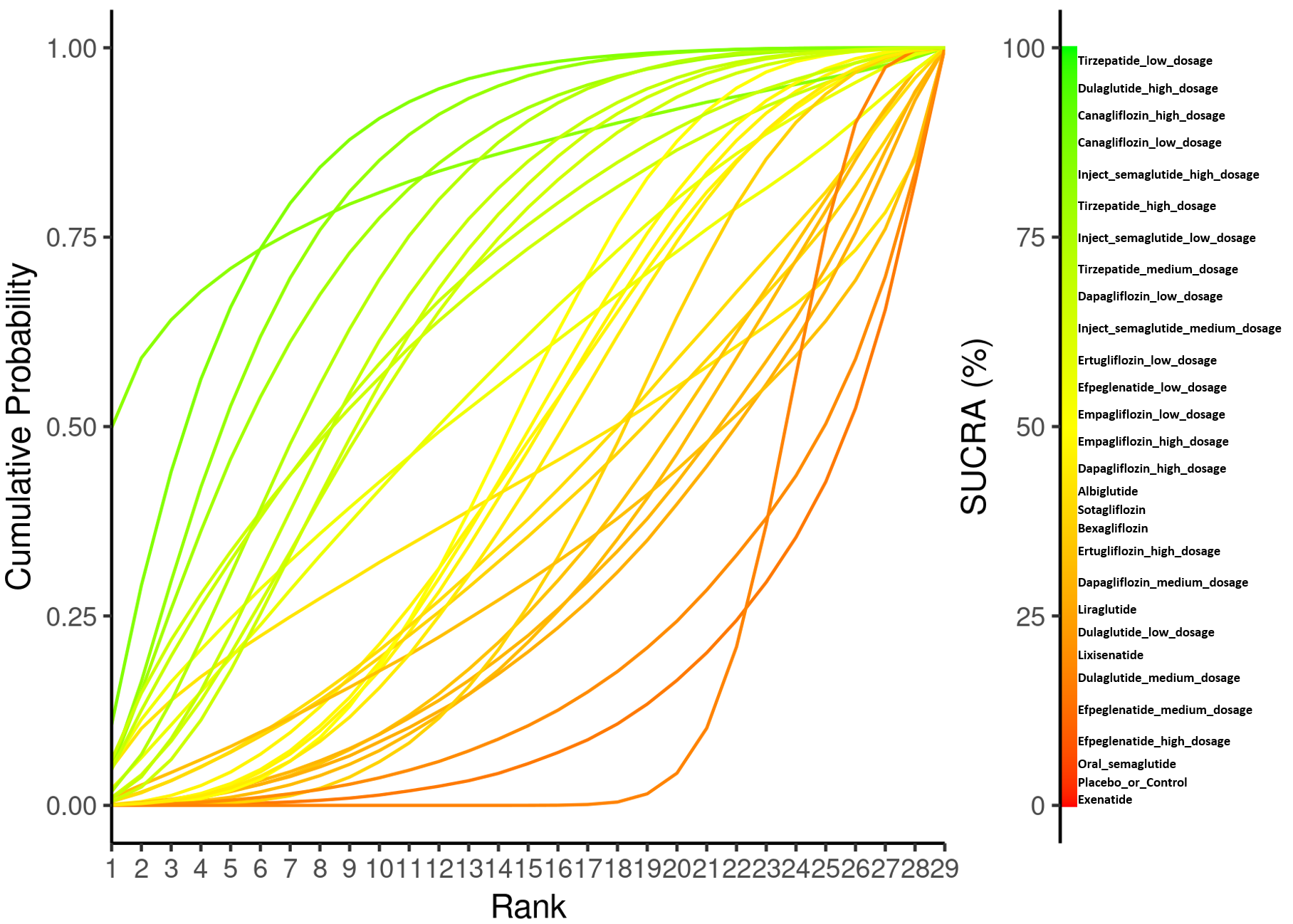
**

**eFigure 5P Bayesian-based radial surface under the cumulative ranking of acceptability: drop-out rate***

**
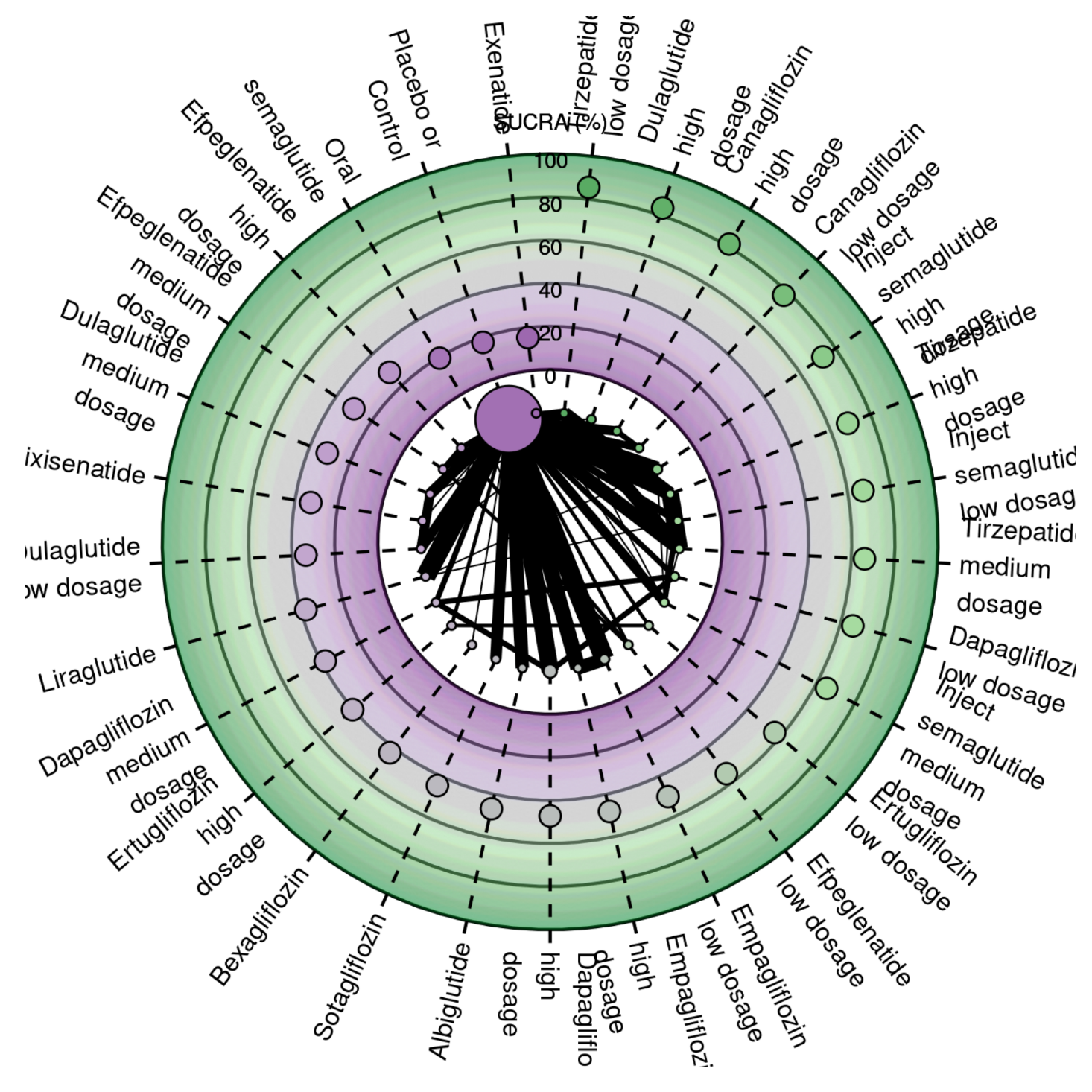
**

**Figure legend of eFigure 5O-5P**

* The outcome of drop-out rate here was calculated according to drop-out rate data from the original composition of subjects from included studies because there were no any studies provided specific information regarding drop-out rate data in female subgroup.

***Abbreviation for eFigure 5A-5P: 95%CIs:***

*95% confidence intervals; GLP-1 agonist: glucagon-like peptide-1 agonist; NMA: network meta-analysis; OR: odds ratio; RCT: randomized controlled trial; SGLT2 inhibitor: sodium–glucose cotransporter 2 inhibitor*

**eFigure 6A Bayesian-based residual deviance NMA/UME model of primary outcome: overall gynecologic tumor**

**
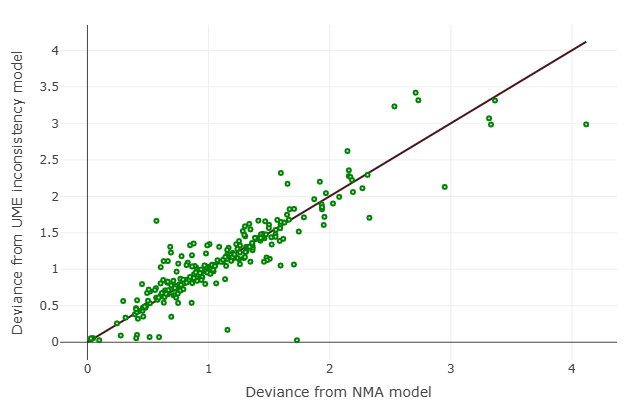
**

**eFigure 6B Bayesian-based per-arm residual deviance of primary outcome: overall gynecologic tumor**

**
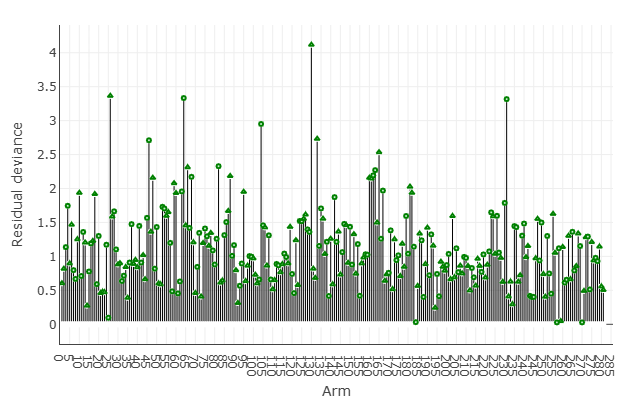
**

**eFigure 6C Bayesian-based leverage plot of primary outcome: overall gynecologic tumor**

**
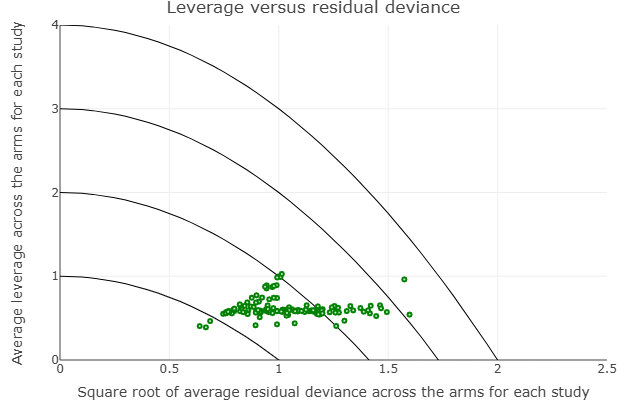
**

**eFigure 6D Bayesian-based residual deviance NMA/UME model of primary outcome: subgroup of intra-uterus tumor**

**
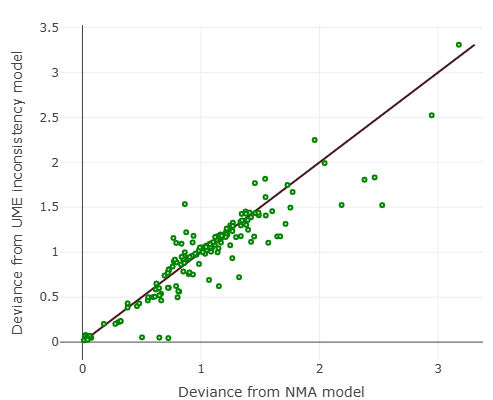
**

**eFigure 6E Bayesian-based per-arm residual deviance of primary outcome: subgroup of intra-uterus tumor**

**
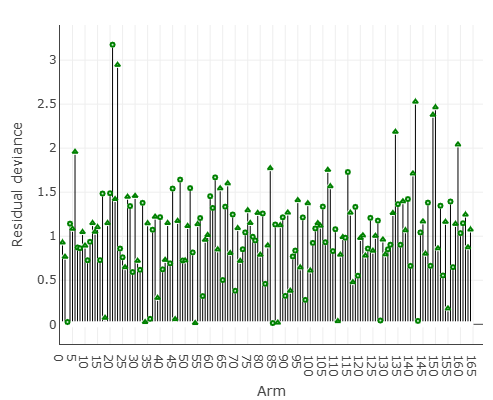
**

**eFigure 6F Bayesian-based leverage plot of primary outcome: subgroup of intra-uterus tumor**

**
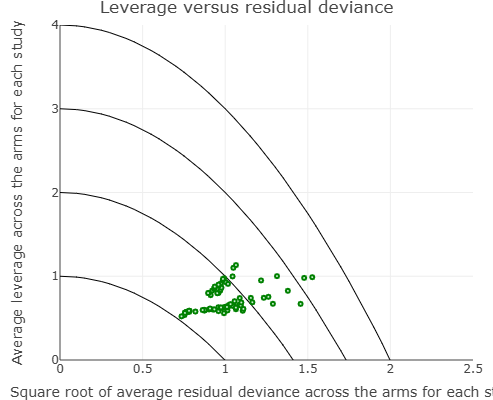
**

**eFigure 6G Bayesian-based residual deviance NMA/UME model of primary outcome: subgroup of cervical tumor**

**
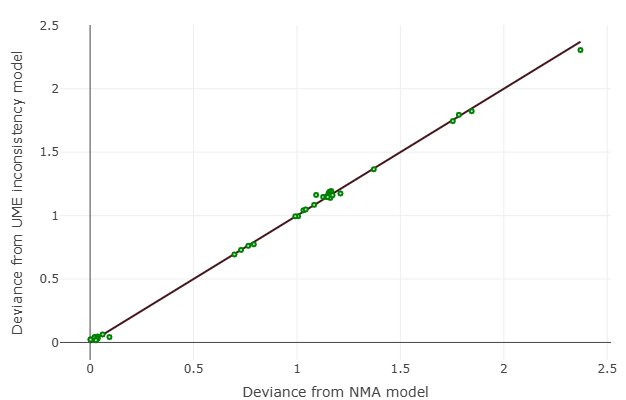
**

**eFigure 6H Bayesian-based per-arm residual deviance of primary outcome: subgroup of cervical tumor**

**
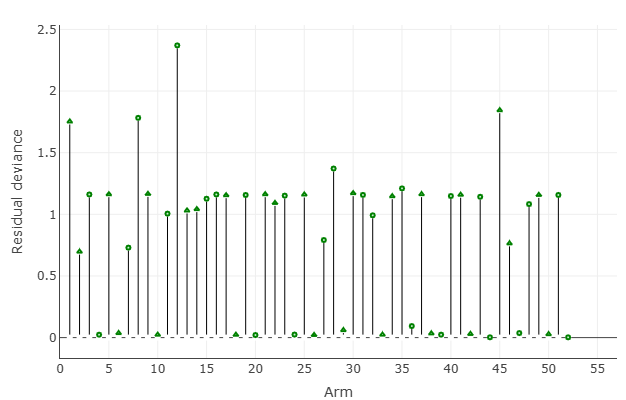
**

**eFigure 6I Bayesian-based leverage plot of primary outcome: subgroup of cervical tumor**

**
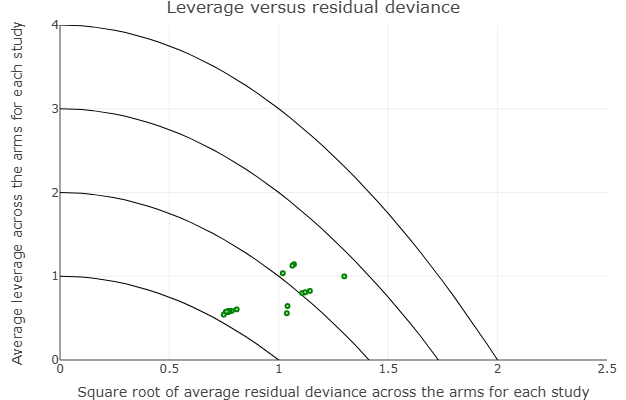
**

**eFigure 6J Bayesian-based residual deviance NMA/UME model of primary outcome: subgroup of ovarian tumor**

**
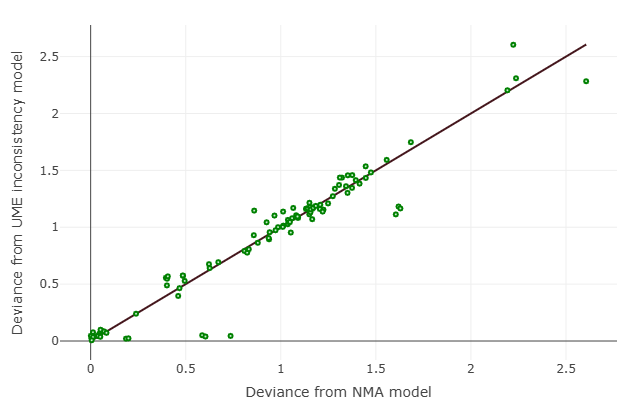
**

**eFigure 6K Bayesian-based per-arm residual deviance of primary outcome: subgroup of ovarian tumor**

**
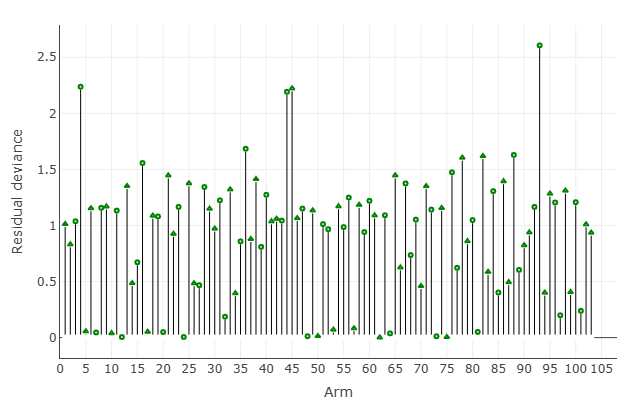
**

**eFigure 6L Bayesian-based leverage plot of primary outcome: subgroup of ovarian tumor**

**
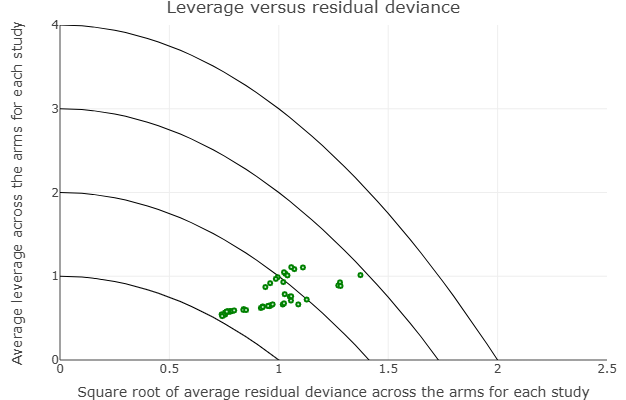
**

**eFigure 6M Bayesian-based residual deviance NMA/UME model of primary outcome: subgroup of breast tumor**

**
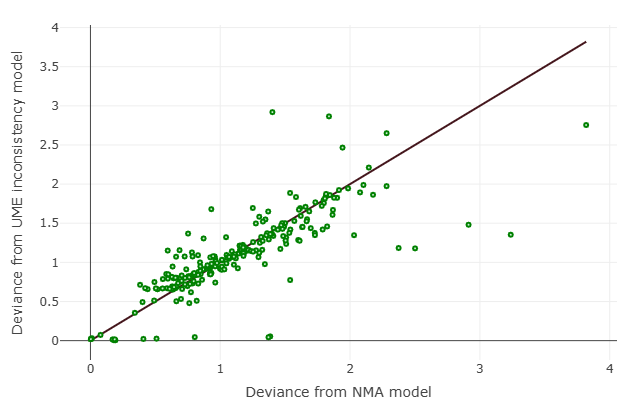
**

**eFigure 6N Bayesian-based per-arm residual deviance of primary outcome: subgroup of breast tumor**

**
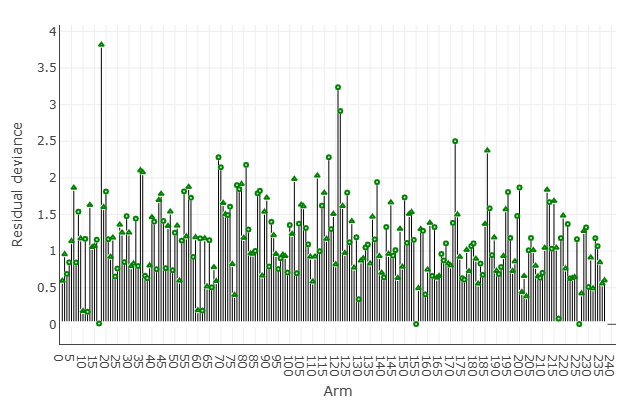
**

**eFigure 6O Bayesian-based leverage plot of primary outcome: subgroup of breast tumor**

**
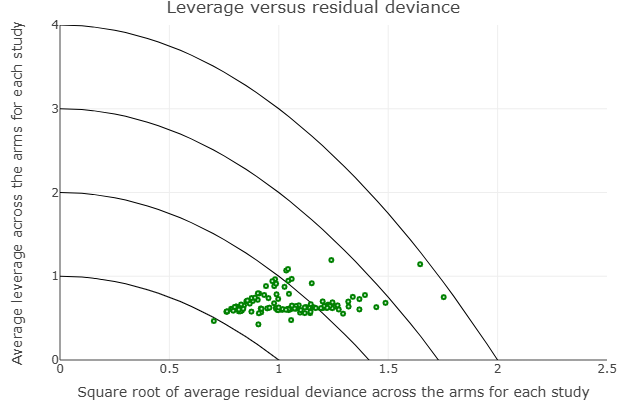
**

**eFigure 6P Bayesian-based residual deviance NMA/UME model of primary outcome: subgroup of vaginal tumor**

**
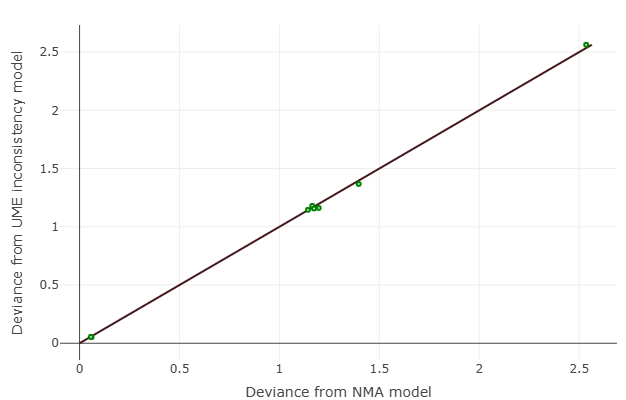
**

**eFigure 6Q Bayesian-based per-arm residual deviance of primary outcome: subgroup of vaginal tumor**

**
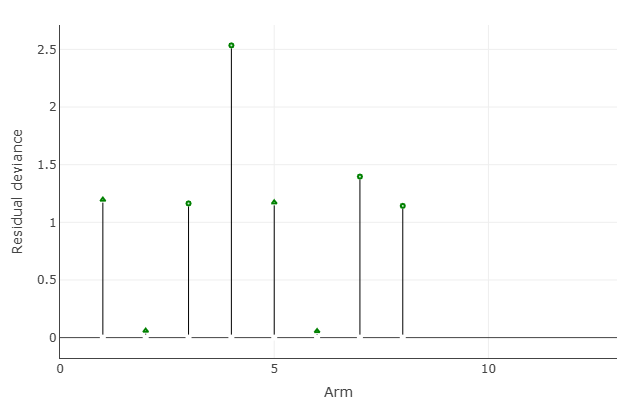
**

**eFigure 6R Bayesian-based leverage plot of primary outcome: subgroup of vaginal tumor**

**
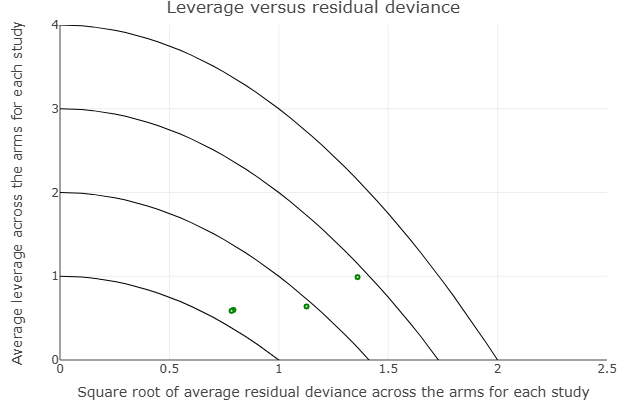
**

**eFigure 6S Bayesian-based residual deviance NMA/UME model of primary outcome: subgroup of vulvar tumor**

**
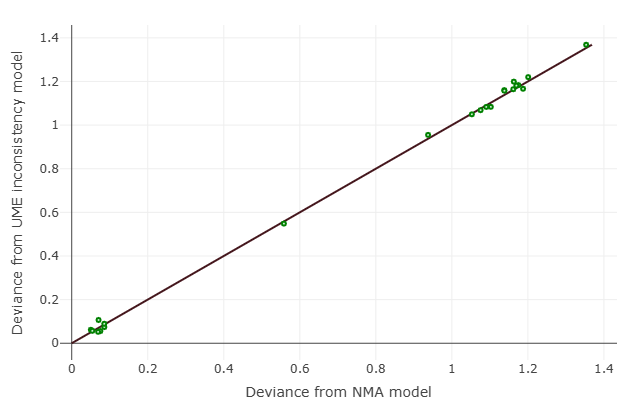
**

**eFigure 6T Bayesian-based per-arm residual deviance of primary outcome: subgroup of vulvar tumor**

**
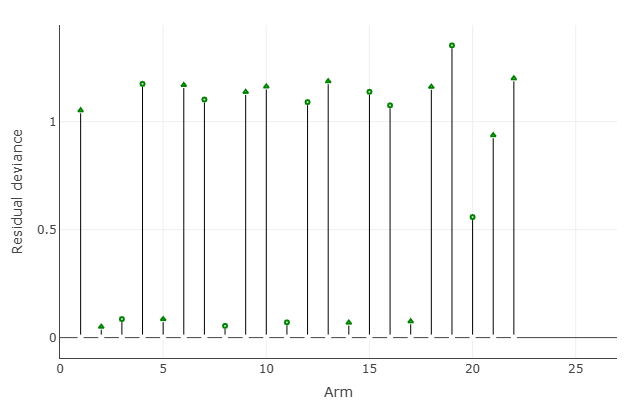
**

**eFigure 6U Bayesian-based leverage plot of primary outcome: subgroup of vulvar tumor**

**
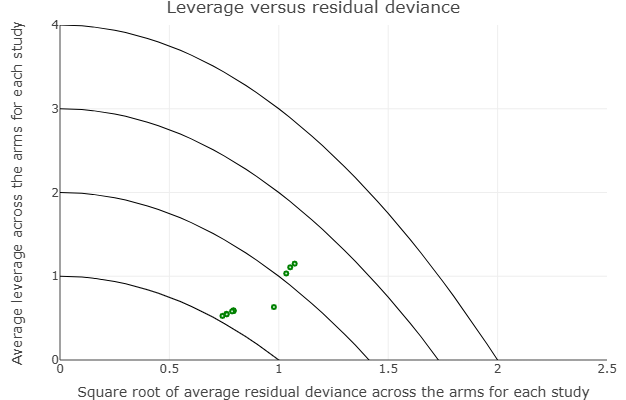
**

**eFigure 6V Bayesian-based residual deviance NMA/UME model of acceptability: drop-out rate***

**
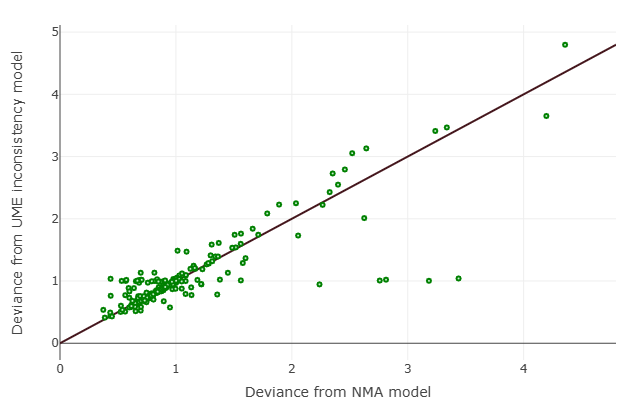
**

**eFigure 6W Bayesian-based per-arm residual deviance of acceptability: drop-out rate***

**
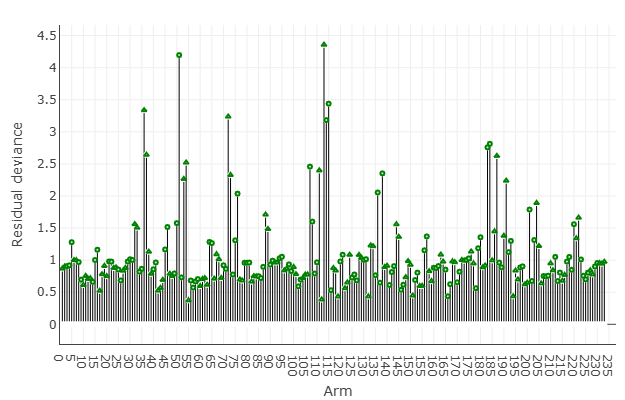
**

**eFigure 6X Bayesian-based leverage plot of acceptability: drop-out rate***

**
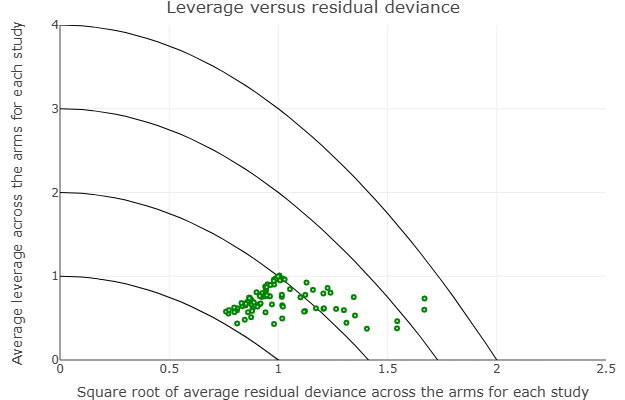
**

**Figure legend of eFigure 6A-6X**

* The outcome of drop-out rate here was calculated according to drop-out rate data from the original composition of subjects from included studies because there were no any studies provided specific information regarding drop-out rate data in female subgroup.

***Abbreviation for eFigure 6A-6X:***

*95%CIs: 95% confidence intervals; GLP-1 agonist: glucagon-like peptide-1 agonist; NMA: network meta-analysis; OR: odds ratio; RCT: randomized controlled trial; SGLT2 inhibitor: sodium–glucose cotransporter 2 inhibitor*

**eFigure 7A Funnel plot of current network meta-analysis of overall gynecologic tumor**

**
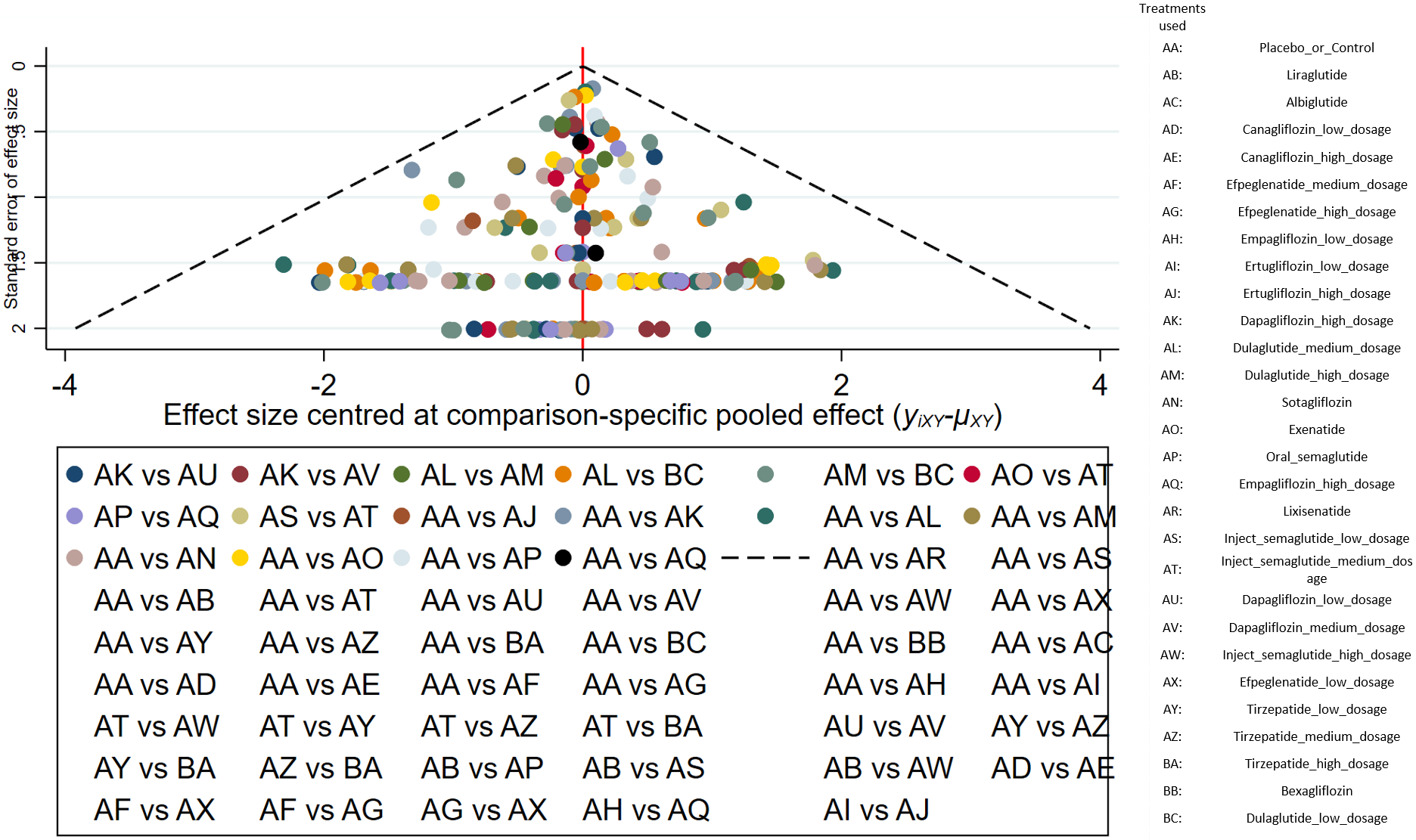
**

**eFigure 7B Egger test of current network meta-analysis of overall gynecologic tumor**

**
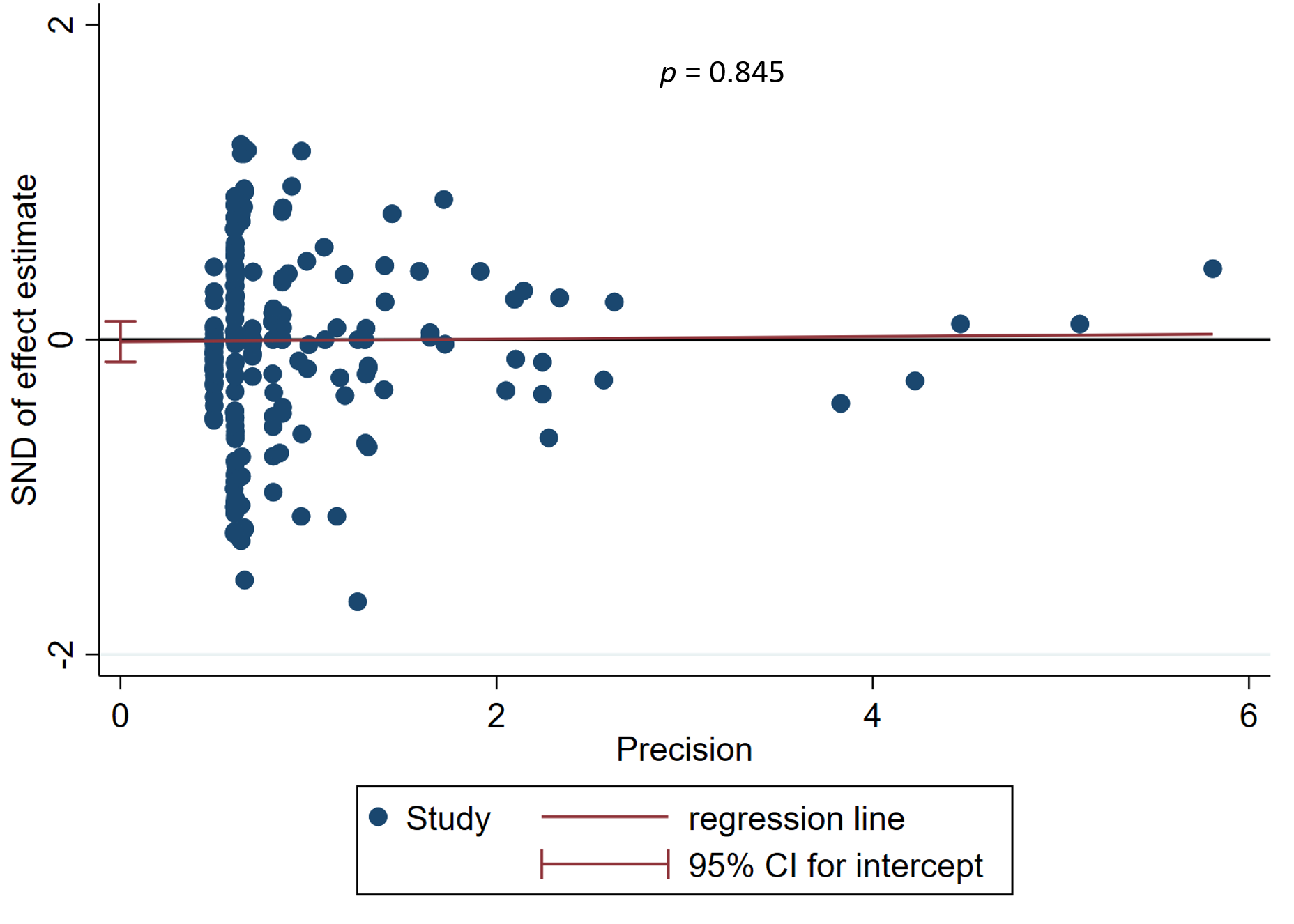
**

**eFigure 8A overview of risk of bias**

**
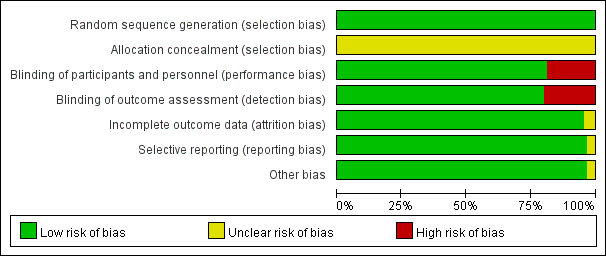
**

**eFigure 8B detailed risk of bias in each study**

**
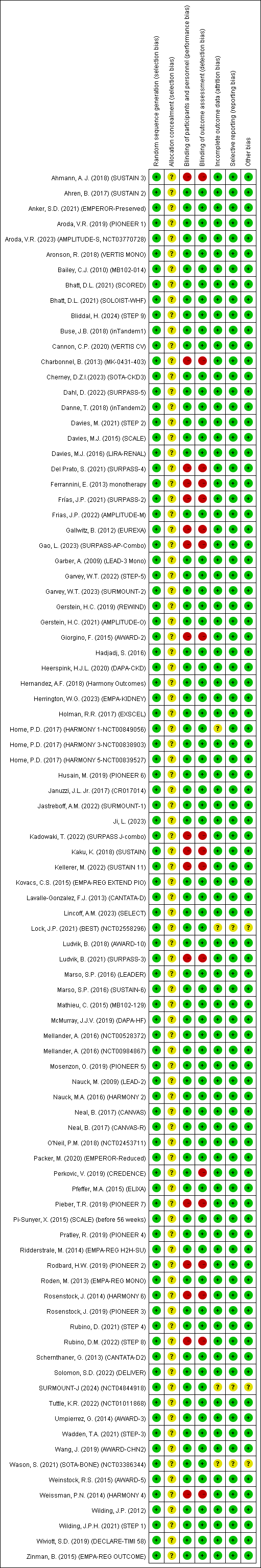
**

**
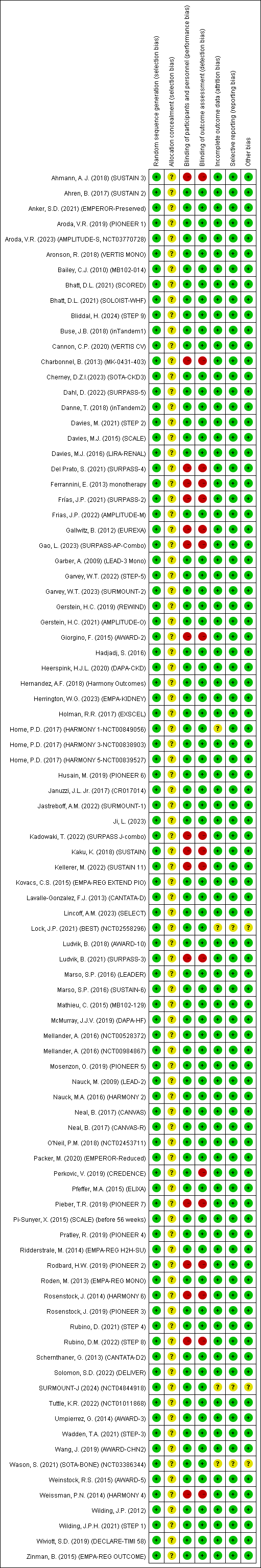
**

**
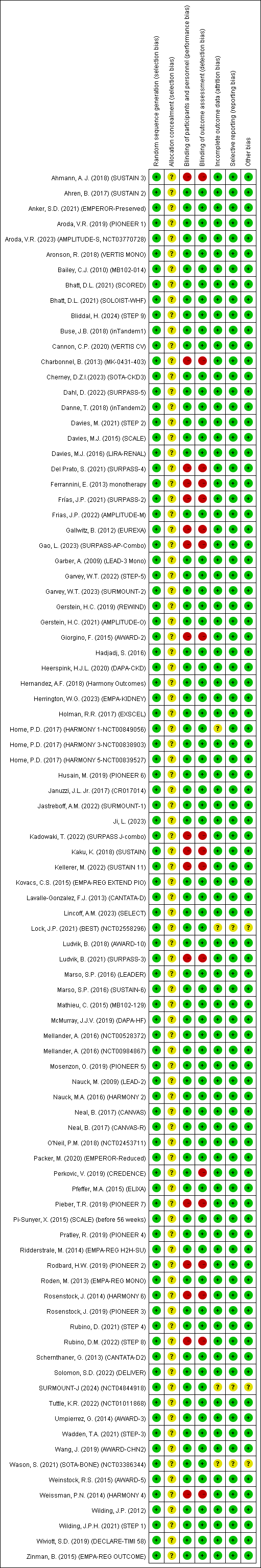
**

**eTable 1:** PRISMA 2020 checklist of the current network meta-analysis

| **Section and Topic** | **Item #** | **Checklist item** | **Page where item is reported** |
| --- | --- | --- | --- |
| **TITLE** | | |  |
| Title | 1 | Identify the report as a systematic review. | 1 |
| **ABSTRACT** | | |  |
| Abstract | 2 | See the PRISMA 2020 for Abstracts checklist. | 7-8 |
| **INTRODUCTION** | | |  |
| Rationale | 3 | Describe the rationale for the review in the context of existing knowledge. | 9-10 |
| Objectives | 4 | Provide an explicit statement of the objective(s) or question(s) the review addresses. | 9-10 |
| **METHODS** | | |  |
| Eligibility criteria | 5 | Specify the inclusion and exclusion criteria for the review and how studies were grouped for the syntheses. | 11-12 |
| Information sources | 6 | Specify all databases, registers, websites, organisations, reference lists and other sources searched or consulted to identify studies. Specify the date when each source was last searched or consulted. | 11-12 |
| Search strategy | 7 | Present the full search strategies for all databases, registers and websites, including any filters and limits used. | 11-12 |
| Selection process | 8 | Specify the methods used to decide whether a study met the inclusion criteria of the review, including how many reviewers screened each record and each report retrieved, whether they worked independently, and if applicable, details of automation tools used in the process. | 11-12 |
| Data collection process | 9 | Specify the methods used to collect data from reports, including how many reviewers collected data from each report, whether they worked independently, any processes for obtaining or confirming data from study investigators, and if applicable, details of automation tools used in the process. | 11-12 |
| Data items | 10a | List and define all outcomes for which data were sought. Specify whether all results that were compatible with each outcome domain in each study were sought (e.g. for all measures, time points, analyses), and if not, the methods used to decide which results to collect. | 12-13 |
|  | 10b | List and define all other variables for which data were sought (e.g. participant and intervention characteristics, funding sources). Describe any assumptions made about any missing or unclear information. | 12-13 |
| Study risk of bias assessment | 11 | Specify the methods used to assess risk of bias in the included studies, including details of the tool(s) used, how many reviewers assessed each study and whether they worked independently, and if applicable, details of automation tools used in the process. | 12-13 |
| Effect measures | 12 | Specify for each outcome the effect measure(s) (e.g. risk ratio, mean difference) used in the synthesis or presentation of results. | 12-13 |
| Synthesis methods | 13a | Describe the processes used to decide which studies were eligible for each synthesis (e.g. tabulating the study intervention characteristics and comparing against the planned groups for each synthesis (item #5)). | 12-13 |
|  | 13b | Describe any methods required to prepare the data for presentation or synthesis, such as handling of missing summary statistics, or data conversions. | 13-15 |
|  | 13c | Describe any methods used to tabulate or visually display results of individual studies and syntheses. | 13-15 |
|  | 13d | Describe any methods used to synthesize results and provide a rationale for the choice(s). If meta-analysis was performed, describe the model(s), method(s) to identify the presence and extent of statistical heterogeneity, and software package(s) used. | 13-15 |
|  | 13e | Describe any methods used to explore possible causes of heterogeneity among study results (e.g. subgroup analysis, meta-regression). | 13-15 |
|  | 13f | Describe any sensitivity analyses conducted to assess robustness of the synthesized results. | 13-15 |
| Reporting bias assessment | 14 | Describe any methods used to assess risk of bias due to missing results in a synthesis (arising from reporting biases). | 13-15 |
| Certainty assessment | 15 | Describe any methods used to assess certainty (or confidence) in the body of evidence for an outcome. | 13-15 |
| **RESULTS** | | |  |
| Study selection | 16a | Describe the results of the search and selection process, from the number of records identified in the search to the number of studies included in the review, ideally using a flow diagram. | 16-17, Fig 1, eTab 2 |
|  | 16b | Cite studies that might appear to meet the inclusion criteria, but which were excluded, and explain why they were excluded. | 16-17, eTab 3 |
| Study characteristics | 17 | Cite each included study and present its characteristics. | 16-17, eTab 4 |
| Risk of bias in studies | 18 | Present assessments of risk of bias for each included study. | 16-17, eFig 7 |
| Results of individual studies | 19 | For all outcomes, present, for each study: (a) summary statistics for each group (where appropriate) and (b) an effect estimate and its precision (e.g. confidence/credible interval), ideally using structured tables or plots. | 16-17, eFig 3 |
| Results of syntheses | 20a | For each synthesis, briefly summarise the characteristics and risk of bias among contributing studies. | 17-18, Fig 2 |
|  | 20b | Present results of all statistical syntheses conducted. If meta-analysis was done, present for each the summary estimate and its precision (e.g. confidence/credible interval) and measures of statistical heterogeneity. If comparing groups, describe the direction of the effect. | 17-18, Fig 3 |
|  | 20c | Present results of all investigations of possible causes of heterogeneity among study results. | 17-18, eTab 7 |
|  | 20d | Present results of all sensitivity analyses conducted to assess the robustness of the synthesized results. | 17-18 |
| Reporting biases | 21 | Present assessments of risk of bias due to missing results (arising from reporting biases) for each synthesis assessed. | 17-18, eFig 7 |
| Certainty of evidence | 22 | Present assessments of certainty (or confidence) in the body of evidence for each outcome assessed. | 17-18 |
| **DISCUSSION** | | |  |
| Discussion | 23a | Provide a general interpretation of the results in the context of other evidence. | 19-21 |
|  | 23b | Discuss any limitations of the evidence included in the review. | 21-22 |
|  | 23c | Discuss any limitations of the review processes used. | 21-22 |
|  | 23d | Discuss implications of the results for practice, policy, and future research. | 23 |
| **OTHER INFORMATION** | | |  |
| Registration and protocol | 24a | Provide registration information for the review, including register name and registration number, or state that the review was not registered. | 8 |
|  | 24b | Indicate where the review protocol can be accessed, or state that a protocol was not prepared. | 8 |
|  | 24c | Describe and explain any amendments to information provided at registration or in the protocol. | 8 |
| Support | 25 | Describe sources of financial or non-financial support for the review, and the role of the funders or sponsors in the review. | 24 |
| Competing interests | 26 | Declare any competing interests of review authors. | 24 |
| Availability of data, code and other materials | 27 | Report which of the following are publicly available and where they can be found: template data collection forms; data extracted from included studies; data used for all analyses; analytic code; any other materials used in the review. | 24 |

The current checklist followed the latest PRISMA 2020 guideline [1].

**eTable 2: Keyword used in each database and search results**

| Database | Keyword | Filter | Date | Result |
| --- | --- | --- | --- | --- |
| PubMed | (uterine OR cervical OR endometrial OR ovarian OR breast OR vaginal OR vulvar) AND (cancer OR tumor) AND (glucagon-like peptide-1 receptor agonist OR Sodium Glucose Cotransporter 2 Inhibitor OR lixisenatide OR orforglipron OR exenatide OR semaglutide OR liraglutide OR albiglutide OR dulaglutide OR tirzepatide OR bexagliflozin OR canagliflozin OR dapagliflozin OR empagliflozin OR ertugliflozin OR ipragliflozin OR luseogliflozin OR remogliflozin OR sergliflozin OR sotagliflozin OR tofogliflozin OR henagliflozin OR janagliflozin OR mizagliflozin OR velagliflozin OR enavogliflozin OR licogliflozin OR rongliflozin) AND (random OR randomized OR randomised) | N/A | 2025/03/03 | 37 |
| ClinicalKey | (uterine OR cervical OR endometrial OR ovarian OR breast OR vaginal OR vulvar) AND (glucagon-like peptide-1 receptor agonist OR Sodium Glucose Cotransporter 2 Inhibitor OR lixisenatide OR orforglipron OR exenatide OR semaglutide OR liraglutide OR albiglutide OR dulaglutide OR tirzepatide OR bexagliflozin OR canagliflozin OR dapagliflozin OR empagliflozin OR ertugliflozin OR ipragliflozin OR luseogliflozin OR remogliflozin OR sergliflozin OR sotagliflozin OR tofogliflozin OR henagliflozin OR janagliflozin OR mizagliflozin OR velagliflozin OR enavogliflozin OR licogliflozin OR rongliflozin) AND (random OR randomized OR randomised) | N/A | 2025/03/03 | 909 |
| Cochrane CENTRAL | (uterine OR cervical OR endometrial OR ovarian OR breast OR vaginal OR vulvar) AND (cancer OR tumor) AND (glucagon-like peptide-1 receptor agonist OR Sodium Glucose Cotransporter 2 Inhibitor OR lixisenatide OR orforglipron OR exenatide OR semaglutide OR liraglutide OR albiglutide OR dulaglutide OR tirzepatide OR bexagliflozin OR canagliflozin OR dapagliflozin OR empagliflozin OR ertugliflozin OR ipragliflozin OR luseogliflozin OR remogliflozin OR sergliflozin OR sotagliflozin OR tofogliflozin OR henagliflozin OR janagliflozin OR mizagliflozin OR velagliflozin OR enavogliflozin OR licogliflozin OR rongliflozin) AND (random OR randomized OR randomised) | N/A | 2025/03/03 | 25 |
| Embase | (uterine OR cervical OR endometrial OR ovarian OR breast OR vaginal OR vulvar) AND (cancer OR tumor) AND (glucagon-like peptide-1 receptor agonist OR Sodium Glucose Cotransporter 2 Inhibitor) AND (random OR randomized OR randomised) | N/A | 2025/03/03 | 159 |
| ProQuest | (uterine OR cervical OR endometrial OR ovarian OR breast OR vaginal OR vulvar) AND (cancer OR tumor) AND (glucagon-like peptide-1 receptor agonist OR Sodium Glucose Cotransporter 2 Inhibitor) AND (random OR randomized OR randomised) | N/A | 2025/03/03 | 3329 |
| ScienceDirect | (uterine OR cervical OR endometrial OR ovarian OR breast OR vaginal OR vulvar) AND (glucagon-like peptide-1 receptor agonist OR Sodium Glucose Cotransporter 2 Inhibitor) | N/A | 2025/03/03 | 7808 |
| Web of Science | (uterine OR cervical OR endometrial OR ovarian OR breast OR vaginal OR vulvar) AND (cancer OR tumor) AND (glucagon-like peptide-1 receptor agonist OR Sodium Glucose Cotransporter 2 Inhibitor) AND (random OR randomized OR randomised) | N/A | 2025/03/03 | 21 |
| ClinicalTrials.gov | (uterine OR cervical OR endometrial OR ovarian OR breast OR vaginal OR vulvar) AND (cancer OR tumor) AND (glucagon-like peptide-1 receptor agonist OR Sodium Glucose Cotransporter 2 Inhibitor) AND (random OR randomized OR randomised) | N/A | 2025/03/03 | 2 |

Abbreviation: N/A: not applied

**eTable 3: Excluded studies and reason**

| Reason | Numbers | References |
| --- | --- | --- |
| Animal study | 1 | [2] |
| Duplicate sample source with another included trial | 1 | [3] |
| Meta-analysis | 7 | [4-10] |
| Not providing specific information regarding dosage | 2 | [11,12] |
| Not randomized controlled trials | 3 | [13-15] |
| Not report target outcome | 50 | [16-65] |
| Review article | 3 | [66-68] |
| Significantly different demographic data at baseline, which would affect gynecologic tumor risk | 1 | [69] |
| Study protocol but not result of a study | 1 | [70] |

**eTable 4: Characteristics of the included studies***

* The demographic data was depicted as the original composition of subjects from the included randomized controlled trials because there were not any studies provided specific information regarding demographic data in female subgroup; however, the effect size of the main primary outcome of overall gynecologic tumor and subgroup analysis of site-specific gynecologic tumor in this study was calculated based on female-specific data.

| Study name | Baseline illness | Comparison | Subjects | Mean age (year) | Female (%) | Treatment duration | Route | Category | ClinicalTrials.gov | Country |
| --- | --- | --- | --- | --- | --- | --- | --- | --- | --- | --- |
| Bliddal, H. (2024) (STEP 9)[71] | patients with obesity and knee osteoarthritis | Inject semaglutide 2.4 mg Placebo | 271 136 | 56.0±10.0 56.0±10.0 | 84.1 76.5 | 68 weeks | injection | GLP-1 receptor agonist | NCT05064735 | Multiple countries |
| SURMOUNT-J (2024) (NCT04844918)[72] | patients with obesity | Tirzepatide 10mg Tirzepatide 15mg Placebo | 73 77 75 | 49.0±10.9 51.1±10.3 52.3±10.9 | 41.1 41.6 40.0 | 72 weeks | injection | GLP-1 receptor agonist | NCT04844918 | Japan |
| Aroda, V.R. (2023) (AMPLITUDE-S, NCT03770728)[64] | patients with type 2 diabetes mellitus | Efpeglenatide 2 mg Efpeglenatide 4 mg Efpeglenatide 6 mg Placebo | 78 77 78 79 | 60.1±10.9 57.9±10.5 58.8±11.5 58.9±10.6 | 43.6 42.9 50.0 43.0 | 30 weeks | injection | GLP-1 receptor agonist | NCT03770728 | Multiple countries |
| Cherney, D.Z.I.(2023) (SOTA-CKD3)[73] | patients with type 2 diabetes mellitus and chronic kidney disease | Sotagliflozin Placebo | 527 260 | 69.5±7.9 69.3±8.1 | 44.0 42.7 | 26 weeks | oral | SGLT2 inhibitor | NCT03242252 | Multiple countries |
| Gao, L. (2023) (SURPASS-AP-Combo)[74] | patients with type 2 diabetes mellitus | Tirzepatide 5mg Tirzepatide 10mg Tirzepatide 15mg Controls with insulin glargine | 230 228 229 220 | 53.1±11.2 53.5±11.1 54.3±11.6 55.6±11.4 | 41.7 44.7 43.7 46.4 | 40 weeks | injection | GLP-1 receptor agonist | NCT04093752 | Multiple countries |
| Garvey, W.T. (2023) (SURMOUNT-2)[75] | patients with obesity | Tirzepatide 10mg Tirzepatide 15mg Placebo | 312 311 315 | 54.3±10.7 53.6±10.6 54.7±10.5 | 50.6 51.1 50.5 | 72 weeks | injection | GLP-1 receptor agonist | NCT04657003 | Multiple countries |
| Herrington, W.G. (2023) (EMPA-KIDNEY)[76] | patients with renal failure | Empagliflozin 10mg Placebo | 3304 3305 | 63.9±13.9 63.8±13.9 | 33.2 33.1 | 104 weeks | oral | SGLT2 inhibitor | NCT03594110 | Multiple countries |
| Ji, L. (2023)[77] | patients with type 2 diabetes mellitus | Empagliflozin 10mg Empagliflozin 25mg Placebo | 73 73 73 | 59.9±7.7 60.7±9.1 60.1±8.0 | 41.1 45.2 50.7 | 24 weeks | oral | SGLT2 inhibitor | NCT04233801 | China |
| Lincoff, A.M. (2023) (SELECT)[78] | patients with obesity | Inject Semaglutide 2.4mg Placebo | 8803 8801 | 61.6±8.9 61.6±8.8 | 27.8 27.5 | 104 weeks | injection | GLP-1 receptor agonist | NCT03574597 | Multiple countries |
| Dahl, D. (2022) (SURPASS-5)[79] | patients with type 2 diabetes mellitus | Tirzepatide 5mg Tirzepatide 10mg Tirzepatide 15mg Placebo | 116 119 120 120 | 62.0±10.0 60.0±10.0 61.0±10.0 60.0±10.0 | 47.4 39.5 45.8 45.0 | 40 weeks | injection | GLP-1 receptor agonist | NCT04039503 | Multiple countries |
| Frias, J.P. (2022) (AMPLITUDE-M)[80] | patients with type 2 diabetes mellitus | Efpeglenatide 2 mg Efpeglenatide 4 mg Efpeglenatide 6 mg Placebo | 100 101 103 102 | 58.6±10.5 56.3±11.5 59.6±10.7 59.5±11.7 | 55.0 51.5 59.2 50.0 | 62 weeks | injection | GLP-1 receptor agonist | NCT03353350 | Multiple countries |
| Garvey, W.T. (2022) (STEP-5)[81] | patients with obesity | Inject semaglutide 2.4 mg Placebo | 152 152 | 47.3±11.7 47.4±10.3 | 80.9 74.3 | 104 weeks | injection | GLP-1 receptor agonist | NCT03693430 | Multiple countries |
| Jastreboff, A.M. (2022) (SURMOUNT-1)[82] | patients with obesity | Tirzepatide 5mg Tirzepatide 10mg Tirzepatide 15mg Placebo | 630 636 630 643 | 45.6±12.7 44.7±12.4 44.9±12.3 44.4±12.5 | 67.6 67.1 67.5 67.8 | 72 weeks | injection | GLP-1 receptor agonist | NCT04184622 | Multiple countries |
| Kadowaki, T. (2022) (SURPASS J-combo)[83] | patients with type 2 diabetes mellitus | Tirzepatide 5mg Tirzepatide 10mg Tirzepatide 15mg | 148 147 148 | 57.7±11.0 56.9±11.2 56.5±10.4 | 19.6 23.1 29.7 | 56 weeks | injection | GLP-1 receptor agonist | NCT03861039 | Japan |
| Kellerer, M. (2022) (SUSTAIN 11)[84] | patients with type 2 diabetes mellitus | Inject semaglutide 1.0 mg Control with insulin glargine and aspart | 874 874 | 60.8±9.4 61.5±9.5 | 49.1 48.6 | 52 weeks | injection | GLP-1 receptor agonist | NCT03689374 | Multiple countries |
| Rubino, D.M. (2022) (STEP 8)[85] | patients with obesity | Inject semaglutide 2.4 mg Liraglutide 3.0 mg Placebo | 126 127 85 | 48.0±14.0 49.0±13.0 51.0±12.0 | 81.0 76.4 77.6 | 68 weeks | injection | GLP-1 receptor agonist | NCT04074161 | Multiple countries |
| Solomon, S.D. (2022) (DELIVER)[86] | patients with stabilized heart failure | Dapagliflozin 10mg Placebo | 3131 3132 | 71.8±9.6 71.5±9.5 | 43.6 44.2 | 120 weeks | oral | SGLT2 inhibitor | NCT03619213 | Multiple countries |
| Tuttle, K.R. (2022) (NCT01011868)[48] | patients with type 2 diabetes mellitus and chronic kidney disease | Empagliflozin 10mg Empagliflozin 25mg Placebo | 169 155 170 | 58.6±9.8 59.9±10.5 58.1±9.4 | 45.0 40.0 47.1 | 78 weeks | oral | SGLT2 inhibitor | NCT01011868 | Multiple countries |
| Anker, S.D. (2021) (EMPEROR-Preserved)[87] | patients with heart failure with preserved ejection fraction | Empagliflozin 10mg Placebo | 2997 2991 | 71.8±9.3 71.9±9.6 | 44.6 44.7 | 156 weeks | oral | SGLT2 inhibitor | NCT03057951 | Multiple countries |
| Bhatt, D.L. (2021) (SCORED)[88] | patients with type 2 diabetes mellitus and chronic kidney disease | Sotagliflozin 200-400mg Placebo | 5292 5292 | 68.4±8.4 68.2±8.4 | 44.3 45.5 | 116 weeks | oral | SGLT2 inhibitor | NCT03315143 | Multiple countries |
| Bhatt, D.L. (2021) (SOLOIST-WHF)[89] | patients with type 2 diabetes mellitus and heart failure | Sotagliflozin 200mg Placebo | 608 614 | 68.6±9.5 69.3±8.8 | 32.6 34.9 | 36 weeks | oral | SGLT2 inhibitor | NCT03521934 | Multiple countries |
| Davies, M. (2021) (STEP 2)[90] | patients with type 2 diabetes mellitus and obesity | Inject semaglutide 1.0 mg Inject Semaglutide 2.4 mg Placebo | 403 404 403 | 56.0±10.0 55.0±11.0 55.0±11.0 | 50.4 55.2 47.1 | 68 weeks | injection | GLP-1 receptor agonist | NCT03552757 | Multiple countries |
| Del Prato, S. (2021) (SURPASS-4)[91] | patients with type 2 diabetes mellitus | Tirzepatide 5mg Tirzepatide 10mg Tirzepatide 15mg Controls with insulin glargine | 329 328 338 1000 | 62.9±8.6 63.7±8.7 63.7±8.6 63.8±8.5 | 39.8 36.3 39.9 36.4 | 108 weeks | injection | GLP-1 receptor agonist | NCT03730662 | Multiple countries |
| Frías, J.P. (2021) (SURPASS-2)[92] | patients with type 2 diabetes mellitus | Tirzepatide 5mg Tirzepatide 10mg Tirzepatide 15mg Inject semaglutide 1.0 mg | 470 469 470 469 | 56.3±10.0 57.2±10.5 55.9±10.4 56.9±10.8 | 56.4 49.3 54.5 52.0 | 40 weeks | injection | GLP-1 receptor agonist | NCT03987919 | Multiple countries |
| Gerstein, H.C. (2021) (AMPLITUDE-O)[93] | patients with type 2 diabetes mellitus | Efpeglenatide 4 mg Efpeglenatide 6 mg Placebo | 1359 1358 1359 | 64.6±8.2 64.7±8.2 64.4±8.3 | 32.5 35.6 30.8 | 104 weeks | injection | GLP-1 receptor agonist | NCT03496298 | Multiple countries |
| Lock, J.P. (2021) (BEST) (NCT02558296)[94] | patients with type 2 diabetes mellitus | Bexagliflozin 20mg Placebo | 1132 567 | 64.4±7.9 64.6±8.0 | 30.1 31.2 | 52 weeks | oral | SGLT2 inhibitor | NCT02558296 | Multiple countries |
| Ludvik, B. (2021) (SURPASS-3)[95] | patients with type 2 diabetes mellitus | Tirzepatide 5mg Tirzepatide 10mg Tirzepatide 15mg Controls with insulin degludec | 358 360 359 360 | 57.2±10.1 57.4±9.7 57.5±10.2 57.5±10.1 | 44.1 45.8 46.0 40.8 | 67 weeks | injection | GLP-1 receptor agonist | NCT03882970 | Multiple countries |
| Rubino, D. (2021) (STEP 4)[96] | patients with overweight or obesity | Inject semaglutide 2.4 mg Placebo | 535 268 | 47.0±12.0 46.0±12.0 | 80.2 76.5 | 68 weeks | injection | GLP-1 receptor agonist | NCT03548987 | Multiple countries |
| Wadden, T.A. (2021) (STEP-3)[97] | patients with obesity | Inject semaglutide 2.4 mg Placebo | 407 204 | 46.0±13.0 46.0±13.0 | 77.4 88.2 | 68 weeks | injection | GLP-1 receptor agonist | NCT03611582 | Multiple countries |
| Wason, S. (2021) (SOTA-BONE) (NCT03386344)[98] | patients with type 2 diabetes mellitus | Sotagliflozin 200-400mg Placebo | 250 126 | 66.3±6.8 66.3±5.7 | 44.4 44.4 | 110 weeks | oral | SGLT2 inhibitor | NCT03386344 | Multiple countries |
| Wilding, J.P.H. (2021) (STEP 1)[99] | patients with obesity | Inject semaglutide 2.4 mg Placebo | 1306 655 | 46.0±13.0 47.0±12.0 | 73.1 76.0 | 68 weeks | injection | GLP-1 receptor agonist | NCT03548935 | Multiple countries |
| Cannon, C.P. (2020) (VERTIS CV)[100] | patients with type 2 diabetes mellitus | Ertugliflozin 5 mg Ertugliflozin 15 mg Placebo | 2752 2747 2747 | 64.3±8.2 64.4±8.0 64.4±8.0 | 29.1 30.3 30.7 | 182 weeks | oral | SGLT2 inhibitor | NCT01986881 | Multiple countries |
| Heerspink, H.J.L. (2020) (DAPA-CKD)[101] | patients with renal failure | Dapagliflozin 10mg Placebo | 2152 2152 | 61.8±12.1 61.9±12.1 | 32.9 33.3 | 125 weeks | oral | SGLT2 inhibitor | NCT03036150 | Multiple countries |
| Packer, M. (2020) (EMPEROR-Reduced)[102] | patients with chronic heart failure | Empagliflozin 10mg Placebo | 1863 1867 | 67.2±10.8 66.5±11.2 | 23.5 24.4 | 64 weeks | oral | SGLT2 inhibitor | NCT03057977 | Multiple countries |
| Aroda, V.R. (2019) (PIONEER 1)[103] | patients with type 2 diabetes mellitus | Oral semaglutide 3-14mg Placebo | 525 178 | 55.0±11.0 54.0±11.0 | 49.0 50.0 | 26 weeks | oral | GLP-1 receptor agonist | NCT02906930 | Multiple countries |
| Gerstein, H.C. (2019) (REWIND)[104] | patients with type 2 diabetes mellitus | Dulaglutide 1.5 mg Placebo | 4949 4952 | 66.2±6.5 66.2±6.5 | 46.6 46.1 | 281 weeks | injection | GLP-1 receptor agonist | NCT01394952 | Multiple countries |
| Husain, M. (2019) (PIONEER 6)[105] | patients with cardiovascular disease or chronic kidney disease | Semaglutide 14mg Placebo | 1591 1592 | 66.0±7.0 66.0±7.0 | 31.9 31.4 | 64 weeks | oral | GLP-1 receptor agonist | NCT02692716 | Multiple countries |
| McMurray, J.J.V. (2019) (DAPA-HF)[106] | patients with stabilized heart failure | Dapagliflozin 10mg Placebo | 2373 2371 | 66.2±11.0 66.5±10.8 | 23.8 23.0 | 73 weeks | oral | SGLT2 inhibitor | NCT03036124 | Multiple countries |
| Mosenzon, O. (2019) (PIONEER 5)[107] | patients with type 2 diabetes mellitus and moderate renal impairment | Oral semaglutide 14mg Placebo | 163 161 | 71.0±8.0 70.0±8.0 | 49.1 54.7 | 31 weeks | oral | GLP-1 receptor agonist | NCT02827708 | Multiple countries |
| Perkovic, V. (2019) (CREDENCE)[108] | patients with type 2 diabetes mellitus and nephropathy | Canagliflozin 100 mg Placebo | 2202 2199 | 62.9±9.2 63.2±9.2 | 34.6 33.3 | 130 weeks | oral | SGLT2 inhibitor | NCT02065791 | Multiple countries |
| Pieber, T.R. (2019) (PIONEER 7)[109] | patients with type 2 diabetes mellitus | Oral semaglutide 3-14mg Control with sitagliptin | 253 251 | 56.9±9.7 57.9±10.1 | 42.7 44.2 | 52 weeks | oral | GLP-1 receptor agonist | NCT02849080 | Multiple countries |
| Pratley, R. (2019) (PIONEER 4)[110] | patients with type 2 diabetes mellitus | Oral semaglutide 14mg Liraglutide 1.8mg Placebo | 285 284 142 | 56.0±10.0 56.0±10.0 57.0±10.0 | 51.6 52.5 52.1 | 52 weeks | oral/injection | GLP-1 receptor agonist | NCT02863419 | Multiple countries |
| Rodbard, H.W. (2019) (PIONEER 2)[111] | patients with type 2 diabetes mellitus | Oral semaglutide 14mg Empagliflozin 25mg | 411 410 | 57.0±10.0 58.0±10.0 | 49.9 49.0 | 52 weeks | oral | GLP-1 receptor agonist/SGLT2 inhibitor | NCT02863328 | Multiple countries |
| Rosenstock, J. (2019) (PIONEER 3)[112] | patients with type 2 diabetes mellitus | Semaglutide 3-14mg Control with sitagliptin | 1396 466 | 57.7±10.0 58.0±10.0 | 46.6 49.0 | 78 weeks | oral | GLP-1 receptor agonist | NCT02607865 | Multiple countries |
| Wang, J. (2019) (AWARD-CHN2)[113] | patients with type 2 diabetes mellitus | Dulaglutide 0.75 mg Dulaglutide 1.5 mg Placebo with insulin glargine | 257 258 253 | 54.5±10.0 55.0±9.6 55.4±9.2 | 43.3 46.6 44.4 | 52 weeks | injection | GLP-1 receptor agonist | NCT01648582 | Multiple countries |
| Wiviott, S.D. (2019) (DECLARE-TIMI 58)[114] | patients with atherosclerotic vascular disease | Dapagliflozin 10mg Placebo | 8582 8578 | 63.9±6.8 64.0±6.8 | 36.9 37.9 | 206 weeks | oral | SGLT2 inhibitor | NCT01730534 | Multiple countries |
| Ahmann, A. J. (2018) (SUSTAIN 3)[115] | patients with type 2 diabetes mellitus | Inject semaglutide 1.0 mg Exenatide | 404 405 | 56.4 56.7 | 45.8 43.7 | 56 weeks | injection | GLP-1 receptor agonist | NCT01885208 | Multiple countries |
| Aronson, R. (2018) (VERTIS MONO)[116] | patients with type 2 diabetes mellitus | Ertugliflozin 5 mg Ertugliflozin 15 mg Placebo | 156 152 153 | 56.8±11.4 56.2±10.8 56.1±10.9 | 42.9 40.8 46.4 | 52 weeks | oral | SGLT2 inhibitor | NCT01958671 | Multiple countries |
| Buse, J.B. (2018) (inTandem1)[117] | patients with type 1 diabetes mellitus | Sotagliflozin Placebo | 525 268 | 46.5±13.3 45.2±12.72 | 53.1 48.9 | 52 weeks | oral | SGLT2 inhibitor | NCT02384941 | Multiple countries |
| Danne, T. (2018) (inTandem2)[118] | patients with type 1 diabetes mellitus | Sotagliflozin 200-400mg Placebo | 524 258 | 42.0±13.4 39.7±13.4 | 48.1 48.1 | 52 weeks | oral | SGLT2 inhibitor | NCT02421510 | Multiple countries |
| Hernandez, A.F. (2018) (Harmony Outcomes)[119] | patients with type 2 diabetes mellitus | Albiglutide 30-50 mg Placebo | 4731 4732 | 64.1±8.7 64.2±8.7 | 30.2 31.0 | 86 weeks | injection | GLP-1 receptor agonist | NCT02465515 | Multiple countries |
| Kaku, K. (2018) (SUSTAIN)[120] | patients with type 2 diabetes mellitus | Inject semaglutide 0.5 mg Inject semaglutide 1.0 mg Control with standard care | 239 241 121 | 58.0±10.6 58.7±10.2 59.2±10.1 | 30.5 27.8 25.8 | 61 weeks | injection | GLP-1 receptor agonist | NCT02207374 | Multiple countries |
| Ludvik, B. (2018) (AWARD-10)[121] | patients with type 2 diabetes mellitus | Dulaglutide 0.75 mg Dulaglutide 1.5 mg Placebo | 141 142 140 | 58.6±9.1 56.2±9.3 57.1±9.6 | 51.1 45.8 52.9 | 24 weeks | injection | GLP-1 receptor agonist | NCT02597049 | Multiple countries |
| O'Neil, P.M. (2018) (NCT02453711)[122] | patients with obesity | Inject semaglutide 0.05 to 0.4 mg Liraglutide 3.0mg Placebo | 718 103 136 | 46.4±12.6 48.5±11.2 46.4±12.8 | 64.6 65.0 64.7 | 52 weeks | injection | GLP-1 receptor agonist | NCT02453711 | Multiple countries |
| Ahren, B. (2017) (SUSTAIN 2)[123] | patients with type 2 diabetes mellitus | Inject semaglutide 1.0 mg Inject semaglutide 0.5 mg Placebo | 409 409 407 | 56.0±9.4 54.8±10.2 54.6±10.4 | 49.9 50.6 51.1 | 56 weeks | injection | GLP-1 receptor agonist | NCT01930188 | Multiple countries |
| Holman, R.R. (2017) (EXSCEL)[124] | patients with type 2 diabetes mellitus | Exenatide 2mg Placebo | 7356 7396 | 61.8±9.4 61.9±9.4 | 38.0 38.0 | 166 weeks | injection | GLP-1 receptor agonist | NCT01144338 | Multiple countries |
| Home, P.D. (2017) (HARMONY 1-NCT00849056)[125] | patients with type 2 diabetes mellitus | Albiglutide 30 mg Placebo | 150 151 | 55.2±10.0 54.9±9.4 | 38.7 41.7 | 52 weeks | injection | GLP-1 receptor agonist | NCT00849056 | Multiple countries |
| Home, P.D. (2017) (HARMONY 3-NCT00838903)[125] | patients with type 2 diabetes mellitus | Albiglutide 30 mg Placebo | 302 101 | 54.3±10.1 56.1±10.0 | 55.3 50.5 | 104 weeks | injection | GLP-1 receptor agonist | NCT00838903 | Multiple countries |
| Home, P.D. (2017) (HARMONY 5-NCT00839527)[125] | patients with type 2 diabetes mellitus | Albiglutide 30-50 mg Placebo | 271 115 | 54.5±9.5 55.7±9.6 | 50.2 39.1 | 156 weeks | injection | GLP-1 receptor agonist | NCT00839527 | Multiple countries |
| Januzzi, J.L. Jr. (2017) (CR017014)[126] | patients with type 2 diabetes mellitus | Canagliflozin 100 mg Canagliflozin 300 mg Placebo | 241 236 237 | 64.3±6.5 63.4±6.0 63.2±6.2 | 48.5 45.3 39.7 | 104 weeks | oral | SGLT2 inhibitor | NCT01106651 | Multiple countries |
| Neal, B. (2017) (CANVAS)[127] | patients with type 2 diabetes mellitus | Canagliflozin 100 mg Canagliflozin 300 mg Placebo | 1445 1443 1442 | 62.2±8.0 62.8±8.1 62.3±7.9 | 33.5 34.6 33.7 | 126 weeks | oral | SGLT2 inhibitor | NCT01032629 | Multiple countries |
| Neal, B. (2017) (CANVAS-R)[127] | patients with type 2 diabetes mellitus | Canagliflozin 300 mg Placebo | 2907 2905 | 63.9±8.4 64.0±8.3 | 36.2 38.2 | 126 weeks | oral | SGLT2 inhibitor | NCT01989754 | Multiple countries |
| Davies, M.J. (2016) (LIRA-RENAL)[128] | patients with type 2 diabetes mellitus | Liraglutide Placebo | 140 137 | 68.0±8.3 66.3±8.0 | 46.4 52.6 | 26 weeks | injection | GLP-1 receptor agonist | NCT01620489 | Multiple countries |
| Hadjadj, S. (2016)[129] | patients with type 2 diabetes mellitus | Empagliflozin 10mg Empagliflozin 25mg Placebo | 497 551 332 | 52.5±11.2 52.4±10.7 52.5±10.9 | 41.1 42.5 46.4 | 24 weeks | oral | SGLT2 inhibitor | NCT01719003 | Multiple countries |
| Marso, S.P. (2016) (LEADER)[130] | patients with type 2 diabetes mellitus | Liraglutide 1.8mg Placebo | 4668 4672 | 64.2±7.2 64.4±7.2 | 35.5 36.0 | 198 weeks | injection | GLP-1 receptor agonist | NCT01179048 | Multiple countries |
| Marso, S.P. (2016) (SUSTAIN-6)[131] | patients with type 2 diabetes mellitus | Inject semaglutide 0.5 mg Inject semaglutide 1.0 mg Placebo | 826 822 1649 | NA | 40.1 37.0 40.0 | 109 weeks | injection | GLP-1 receptor agonist | NCT01720446 | Multiple countries |
| Mellander, A. (2016) (NCT00528372)[132] | patients with type 2 diabetes mellitus | Dapagliflozin 2.5 mg Dapagliflozin 5.0 mg Dapagliflozin 10.0 mg Placebo | 132 132 146 75 | NA | 50.7 54.6 50.0 58.7 | 102 weeks | oral | SGLT2 inhibitor | NCT00528372 | Multiple countries |
| Mellander, A. (2016) (NCT00984867)[132] | patients with type 2 diabetes mellitus | Dapagliflozin 10mg Placebo | 223 224 | 54.8±10.4 55.0±10.2 | 43.0 47.3 | 48 weeks | oral | SGLT2 inhibitor | NCT00984867 | Multiple countries |
| Nauck, M.A. (2016) (HARMONY 2)[133] | patients with type 2 diabetes mellitus | Albiglutide 30-50 mg Placebo | 200 101 | 52.8±11.4 53.1±11.7 | 46.0 42.6 | 52 weeks | injection | GLP-1 receptor agonist | NCT00849017 | Multiple countries |
| Davies, M.J. (2015) (SCALE)[134] | patients with obesity | Liraglutide 1.8-3.0 mg Placebo | 634 212 | 55.0±10.8 54.7±9.8 | 48.3 54.2 | 56 weeks | injection | GLP-1 receptor agonist | NCT01272232 | Multiple countries |
| Giorgino, F. (2015) (AWARD-2)[135] | patients with type 2 diabetes mellitus | Dulaglutide 0.75 mg Dulaglutide 1.5 mg Control with insulin glargine | 272 273 262 | 56.6±9.3 56.2±9.8 57.0±9.0 | 50.0 47.3 48.9 | 78 weeks | injection | GLP-1 receptor agonist | NCT01075282 | Multiple countries |
| Kovacs, C.S. (2015) (EMPA-REG EXTEND PIO)[136] | patients with type 2 diabetes mellitus | Empagliflozin 10mg Empagliflozin 25mg Placebo | 165 168 165 | 54.7±9.9 54.2±8.9 54.6±10.5 | 49.7 49.4 55.8 | 24 weeks | oral | SGLT2 inhibitor | NCT01210001 | Multiple countries |
| Mathieu, C. (2015) (MB102-129)[137] | patients with type 2 diabetes mellitus | Dapagliflozin 10mg Placebo | 160 160 | 55.0±9.6 55.2±8.6 | 52.5 56.3 | 52 weeks | oral | SGLT2 inhibitor | NCT01646320 | Multiple countries |
| Pfeffer, M.A. (2015) (ELIXA)[138] | patients with type 2 diabetes mellitus and recent acute coronary syndrome | Lixisenatide 20ug Placebo | 3034 3034 | 59.9±9.7 60.6±9.6 | 30.4 30.9 | 100 weeks | injection | GLP-1 receptor agonist | NCT01147250 | Multiple countries |
| Pi-Sunyer, X. (2015) (SCALE) (before 56 weeks)[139] | patients with obesity | Liraglutide 3.0mg Placebo | 2487 1244 | 45.2±12.1 45.0±12.0 | 78.7 78.1 | 56 weeks | injection | GLP-1 receptor agonist | NCT01272219 | Multiple countries |
| Weinstock, R.S. (2015) (AWARD-5)[140] | patients with type 2 diabetes mellitus | Dulaglutide 0.25-1.0 mg Dulaglutide 1.5 mg Dulaglutide 2.0-3.0 mg Placebo or sitagliptin | 361 304 45 177 | 54.5±9.6 53.7±10.0 52.6±10.9 54.9±9.1 | 55.9 52.0 71.1 49.2 | 104 weeks | injection | GLP-1 receptor agonist | NCT00734474 | Multiple countries |
| Zinman, B. (2015) (EMPA-REG OUTCOME)[141] | patients with type 2 diabetes mellitus | Empagliflozin 10mg Empagliflozin 25mg Placebo | 2345 2342 2333 | 63.0±8.6 63.2±8.6 63.2±8.8 | 29.5 28.1 28.0 | 135 weeks | oral | SGLT2 inhibitor | NCT01131676 | Multiple countries |
| Ridderstrale, M. (2014) (EMPA-REG H2H-SU)[142] | patients with type 2 diabetes mellitus and moderate-to-severe chronic kidney disease | Empagliflozin 25mg Control with glimepiride | 765 780 | 56.2±10.3 55.7±10.4 | 43.5 46.0 | 104 weeks | oral | SGLT2 inhibitor | NCT01167881 | Multiple countries |
| Rosenstock, J. (2014) (HARMONY 6)[143] | patients with type 2 diabetes mellitus | Albiglutide 30-50 mg Control with Insulin glargine and lispro | 285 281 | 54.8±9.1 56.3±8.9 | 53.7 51.6 | 52 weeks | injection | GLP-1 receptor agonist | NCT00976391 | Multiple countries |
| Umpierrez, G. (2014) (AWARD-3)[144] | patients with type 2 diabetes mellitus | Dulaglutide 0.75 mg Dulaglutide 1.5 mg Control with insulin glargine | 270 269 268 | 55.9±10.7 55.5±10.4 55.0±10.0 | 56.3 57.6 54.9 | 52 weeks | injection | GLP-1 receptor agonist | NCT01126580 | Multiple countries |
| Weissman, P.N. (2014) (HARMONY 4)[145] | patients with type 2 diabetes mellitus | Albiglutide 30 mg Control with insulin glargine | 504 241 | 55.8±9.3 54.7±9.8 | 43.3 45.2 | 52 weeks | injection | GLP-1 receptor agonist | NCT00838916 | Multiple countries |
| Charbonnel, B. (2013) (MK-0431-403)[146] | patients with type 2 diabetes mellitus | Liraglutide 1.2 mg Controls with Sitagliptin +/- Glimepiride | 327 326 | 57.6±10.8 56.9±10.0 | 45.0 45.4 | 26 weeks | injection | GLP-1 receptor agonist | NCT01296412 | Multiple countries |
| Ferrannini, E. (2013) monotherapy[147] | patients with type 2 diabetes mellitus | Empagliflozin 10mg Empagliflozin 25mg Placebo | 106 109 56 | 59.0 59.0 58.0 | 53.8 47.7 50.0 | 78 weeks | oral | SGLT2 inhibitor | NCT00881530 | Multiple countries |
| Lavalle-Gonzalez, F.J. (2013) (data before week 26) (CANTATA-D)[148] | patients with type 2 diabetes mellitus | Canagliflozin 100 mg Canagliflozin 300 mg Placebo | 368 367 183 | 55.5±9.4 55.3±9.2 55.3±9.8 | 52.7 55.0 48.6 | 26 weeks | oral | SGLT2 inhibitor | NCT01106677 | Multiple countries |
| Roden, M. (2013) (EMPA-REG MONO)[149] | patients with type 2 diabetes mellitus | Empagliflozin 10mg Empagliflozin 25mg Placebo | 224 224 228 | 56.2±11.6 53.8±11.6 54.9±10.9 | 36.6 35.3 46.1 | 24 weeks | oral | SGLT2 inhibitor | NCT01177813 | Multiple countries |
| Schernthaner, G. (2013) (CANTATA-D2)[150] | patients with type 2 diabetes mellitus | Canagliflozin 300 mg Control with Sitagliptin | 377 378 | 56.6±9.6 56.7±9.3 | 45.1 43.1 | 52 weeks | oral | SGLT2 inhibitor | NCT01137812 | Multiple countries |
| Gallwitz, B. (2012) (EUREXA)[151] | patients with type 2 diabetes mellitus | Exenatide Control with glimepiride | 490 487 | 56.0±10.0 56.0±9.1 | 44.5 48.3 | 104 weeks | injection | GLP-1 receptor agonist | NCT00359762 | Multiple countries |
| Wilding, J.P. (2012)[152] | patients with type 2 diabetes mellitus and moderate-to-severe chronic kidney disease | Dapagliflozin 2.5 mg Dapagliflozin 5.0 mg Dapagliflozin 10.0 mg Placebo | 202 211 194 193 | 59.8±7.6 59.3±7.9 59.3±8.8 58.8±8.6 | 50.5 52.6 55.2 50.8 | 104 weeks | oral | SGLT2 inhibitor | NCT00673231 | Multiple countries |
| Bailey, C.J. (2010) (MB102-014)[153] | patients with type 2 diabetes mellitus | Dapagliflozin 2.5 mg Dapagliflozin 5.0 mg Dapagliflozin 10.0 mg Placebo | 137 137 135 137 | 55.0±9.3 54.3±9.4 52.7±9.9 53.7±10.3 | 48.9 49.6 43.0 44.5 | 24 weeks | oral | SGLT2 inhibitor | NCT00528879 | Multiple countries |
| Garber, A. (2009) (LEAD-3 Mono)[154] | patients with type 2 diabetes mellitus | Liraglutide 1.2-1.8 mg Placebo with glimepiride | 498 248 | 52.9±10.9 53.4±10.9 | 47.8 53.6 | 104 weeks | injection | GLP-1 receptor agonist | NCT00294723 | Multiple countries |
| Nauck, M. (2009) (LEAD-2)[155] | patients with type 2 diabetes mellitus | Liraglutide Placebo | 724 121 | 56.7±9.7 56.0±9.0 | 41.7 40.0 | 26 weeks | injection | GLP-1 receptor agonist | NCT00318461 | Multiple countries |

*Abbreviations: GLP-1 agonist: glucagon-like peptide-1 agonist; NA: not available; SGLT2 inhibitor: sodium–glucose cotransporter 2 inhibitor*

**eTable 5A: League table of NMA of the primary outcome: subgroup of uterine tumor**

| Bexagliflozin | . | . | . | . | . | . | . | . | . | . | 0.17 [0.01; 4.25] | . | . | . | . | . | . | . | . | . | . | . | . | . | . |
| --- | --- | --- | --- | --- | --- | --- | --- | --- | --- | --- | --- | --- | --- | --- | --- | --- | --- | --- | --- | --- | --- | --- | --- | --- | --- |
| 0.38 [0.01; 13.07] | Sotagliflozin | . | . | . | . | . | . | . | . | . | 0.46 [0.10; 2.08] | . | . | . | . | . | . | . | . | . | . | . | . | . | . |
| 0.43 [0.01; 21.13] | 1.15 [0.08; 16.64] | Inject_  semaglutide_  low_dosage | . | . | . | . | . | . | . | . | 0.46 [0.05; 4.47] | . | . | . | . | . | . | . | . | . | 0.18 [0.01; 3.82] | . | . | . | . |
| 0.32 [0.01; 9.13] | 0.85 [0.14; 5.15] | 0.74 [0.07; 8.24] | Dulaglutide_  medium_dosage | . | . | 0.77 [0.08; 7.05] | . | . | . | 0.62 [0.02; 15.54] | 0.48 [0.18; 1.31] | . | . | . | . | . | . | . | . | . | . | . | . | . | . |
| 0.51 [0.01; 47.36] | 1.35 [0.04; 46.82] | 1.18 [0.02; 57.56] | 1.59 [0.06; 45.23] | Ertugliflozin_  high_dosage | . | . | . | . | . | . | 0.34 [0.01; 8.30] | 0.32 [0.01; 7.88] | . | . | . | . | . | . | . | . | . | . | . | . | . |
| 0.27 [0.01; 7.97] | 0.72 [0.11; 4.63] | 0.63 [0.05; 7.29] | 0.84 [0.20; 3.62] | 0.53 [0.02; 15.58] | Liraglutide | . | . | . | . | . | 0.64 [0.22; 1.88] | . | . | . | . | . | . | . | . | . | . | . | . | . | . |
| 0.27 [0.01; 8.75] | 0.71 [0.09; 5.52] | 0.62 [0.05; 8.32] | 0.83 [0.18; 3.82] | 0.52 [0.02; 17.10] | 0.98 [0.17; 5.72] | Dulaglutide_  low_dosage | . | . | . | . | 0.77 [0.16; 3.64] | . | . | . | . | . | . | . | . | . | . | . | . | . | . |
| 0.24 [0.01; 7.74] | 0.63 [0.08; 4.83] | 0.55 [0.04; 7.32] | 0.75 [0.14; 3.93] | 0.47 [0.01; 15.12] | 0.88 [0.16; 4.98] | 0.90 [0.13; 6.22] | Albiglutide | . | . | . | 0.72 [0.19; 2.77] | . | . | . | . | . | . | . | . | . | . | . | . | . | . |
| 0.23 [0.01; 8.10] | 0.62 [0.07; 5.28] | 0.54 [0.04; 7.83] | 0.73 [0.12; 4.40] | 0.46 [0.01; 15.83] | 0.87 [0.14; 5.55] | 0.88 [0.11; 6.83] | 0.98 [0.13; 7.41] | Canagliflozin_  low_dosage | . | . | 0.98 [0.18; 5.37] | . | . | . | . | . | . | 0.51 [0.09; 2.82] | . | . | . | . | . | . | . |
| 0.19 [0.01; 5.56] | 0.51 [0.08; 3.18] | 0.44 [0.04; 5.05] | 0.60 [0.15; 2.46] | 0.38 [0.01; 10.86] | 0.71 [0.16; 3.15] | 0.72 [0.13; 4.05] | 0.80 [0.15; 4.37] | 0.82 [0.13; 5.06] | Exenatide | . | 0.90 [0.32; 2.50] | . | . | . | . | . | . | . | . | . | . | . | . | . | . |
| 0.20 [0.00; 20.71] | 0.53 [0.01; 21.13] | 0.46 [0.01; 25.65] | 0.62 [0.02; 15.54] | 0.39 [0.00; 40.50] | 0.73 [0.02; 25.18] | 0.75 [0.02; 26.34] | 0.83 [0.02; 31.17] | 0.85 [0.02; 33.78] | 1.03 [0.03; 34.86] | Dulaglutide_  high_dosage | . | . | . | . | . | . | . | . | . | . | . | . | . | . | . |
| 0.17 [0.01; 4.25] | 0.46 [0.10; 2.08] | 0.40 [0.04; 3.61] | 0.54 [0.20; 1.42] | 0.34 [0.01; 8.30] | 0.64 [0.22; 1.88] | 0.65 [0.16; 2.59] | 0.72 [0.19; 2.77] | 0.73 [0.16; 3.31] | 0.90 [0.32; 2.50] | 0.87 [0.03; 25.17] | Placebo_  or_Control | 0.95 [0.10; 9.14] | . | . | 0.86 [0.32; 2.32] | 0.76 [0.10; 5.92] | 0.69 [0.19; 2.55] | 0.64 [0.18; 2.28] | 0.28 [0.05; 1.74] | 0.90 [0.15; 5.31] | 0.46 [0.12; 1.82] | 0.31 [0.01; 7.89] | 0.28 [0.01; 7.15] | . | 0.25 [0.03; 2.32] |
| 0.16 [0.00; 8.28] | 0.43 [0.03; 6.63] | 0.38 [0.02; 8.92] | 0.51 [0.04; 6.01] | 0.32 [0.01; 7.88] | 0.60 [0.05; 7.45] | 0.61 [0.04; 8.75] | 0.68 [0.05; 9.55] | 0.70 [0.05; 10.58] | 0.85 [0.07; 10.23] | 0.82 [0.01; 47.69] | 0.95 [0.10; 9.14] | Ertugliflozin_  low_dosage | . | . | . | . | . | . | . | . | . | . | . | . | . |
| 0.15 [0.00; 40.15] | 0.40 [0.00; 49.43] | 0.35 [0.00; 55.82] | 0.48 [0.00; 50.39] | 0.30 [0.00; 78.54] | 0.56 [0.01; 61.15] | 0.57 [0.00; 67.28] | 0.64 [0.01; 74.06] | 0.65 [0.01; 79.10] | 0.79 [0.01; 85.01] | 0.77 [0.00; 222.42] | 0.88 [0.01; 84.56] | 0.93 [0.01; 151.60] | Efpeglenatide_  low_dosage | . | . | . | . | . | . | . | . | 0.36 [0.01; 8.95] | . | . | . |
| 0.14 [0.00; 37.52] | 0.38 [0.00; 46.20] | 0.33 [0.00; 52.17] | 0.44 [0.00; 47.10] | 0.28 [0.00; 73.40] | 0.53 [0.00; 57.15] | 0.53 [0.00; 62.88] | 0.59 [0.01; 69.22] | 0.61 [0.00; 73.93] | 0.74 [0.01; 79.46] | 0.72 [0.00; 207.86] | 0.83 [0.01; 79.03] | 0.87 [0.01; 141.69] | 0.93 [0.01; 89.55] | Efpeglenatide_  high_dosage | . | . | . | . | . | . | . | 0.38 [0.02; 9.59] | . | . | . |
| 0.15 [0.01; 4.25] | 0.39 [0.06; 2.41] | 0.34 [0.03; 3.84] | 0.46 [0.12; 1.86] | 0.29 [0.01; 8.29] | 0.55 [0.13; 2.38] | 0.56 [0.10; 3.06] | 0.62 [0.12; 3.30] | 0.63 [0.10; 3.83] | 0.77 [0.19; 3.21] | 0.75 [0.02; 24.98] | 0.86 [0.32; 2.32] | 0.91 [0.08; 10.74] | 0.97 [0.01; 103.62] | 1.04 [0.01; 111.01] | Dapagliflozin_  high_dosage | . | . | . | . | . | . | . | . | . | . |
| 0.13 [0.00; 5.89] | 0.35 [0.03; 4.46] | 0.30 [0.01; 6.15] | 0.41 [0.04; 3.96] | 0.26 [0.01; 11.51] | 0.48 [0.05; 4.93] | 0.49 [0.04; 5.86] | 0.55 [0.05; 6.38] | 0.56 [0.04; 7.12] | 0.68 [0.07; 6.76] | 0.66 [0.01; 34.03] | 0.76 [0.10; 5.92] | 0.80 [0.04; 17.02] | 0.86 [0.01; 127.82] | 0.92 [0.01; 136.93] | 0.88 [0.09; 8.63] | Oral_  semaglutide | . | . | . | . | . | . | . | . | . |
| 0.13 [0.00; 4.13] | 0.35 [0.05; 2.49] | 0.31 [0.03; 3.74] | 0.42 [0.09; 1.99] | 0.26 [0.01; 8.06] | 0.49 [0.10; 2.53] | 0.50 [0.08; 3.19] | 0.56 [0.09; 3.45] | 0.57 [0.08; 3.96] | 0.70 [0.14; 3.43] | 0.67 [0.02; 24.20] | 0.78 [0.23; 2.63] | 0.82 [0.06; 10.72] | 0.88 [0.01; 98.63] | 0.94 [0.01; 105.66] | 0.90 [0.19; 4.35] | 1.02 [0.09; 11.14] | Inject_  semaglutide_  high_dosage | . | . | . | 0.30 [0.01; 7.46] | . | . | . | . |
| 0.12 [0.00; 3.71] | 0.32 [0.05; 2.22] | 0.28 [0.02; 3.44] | 0.38 [0.08; 1.77] | 0.24 [0.01; 7.25] | 0.45 [0.09; 2.26] | 0.45 [0.07; 2.85] | 0.51 [0.08; 3.08] | 0.52 [0.12; 2.23] | 0.63 [0.13; 3.06] | 0.61 [0.02; 21.77] | 0.70 [0.21; 2.34] | 0.74 [0.06; 9.62] | 0.79 [0.01; 88.89] | 0.85 [0.01; 95.23] | 0.82 [0.17; 3.88] | 0.92 [0.09; 9.99] | 0.91 [0.16; 5.03] | Canagliflozin_  high_dosage | . | . | . | . | . | . | . |
| 0.10 [0.00; 3.64] | 0.27 [0.03; 2.43] | 0.24 [0.02; 3.56] | 0.32 [0.05; 2.04] | 0.20 [0.01; 7.12] | 0.38 [0.06; 2.57] | 0.38 [0.05; 3.15] | 0.43 [0.05; 3.42] | 0.43 [0.05; 3.87] | 0.53 [0.08; 3.51] | 0.51 [0.01; 21.24] | 0.59 [0.12; 2.89] | 0.62 [0.04; 9.90] | 0.67 [0.01; 83.80] | 0.72 [0.01; 89.77] | 0.69 [0.11; 4.46] | 0.78 [0.06; 10.43] | 0.76 [0.10; 5.65] | 0.84 [0.12; 6.16] | Empagliflozin_  low_dosage | 0.50 [0.09; 2.78] | . | . | . | . | . |
| 0.09 [0.00; 3.31] | 0.25 [0.03; 2.18] | 0.22 [0.01; 3.21] | 0.29 [0.05; 1.82] | 0.19 [0.01; 6.46] | 0.35 [0.05; 2.29] | 0.35 [0.04; 2.82] | 0.39 [0.05; 3.06] | 0.40 [0.05; 3.47] | 0.49 [0.08; 3.13] | 0.48 [0.01; 19.29] | 0.55 [0.12; 2.55] | 0.58 [0.04; 8.93] | 0.62 [0.01; 76.43] | 0.66 [0.01; 81.87] | 0.64 [0.10; 3.98] | 0.72 [0.06; 9.40] | 0.71 [0.10; 5.05] | 0.78 [0.11; 5.50] | 0.93 [0.21; 4.14] | Empagliflozin_  high_dosage | . | . | . | . | . |
| 0.08 [0.00; 2.44] | 0.21 [0.03; 1.44] | 0.19 [0.02; 1.86] | 0.25 [0.06; 1.14] | 0.16 [0.01; 4.77] | 0.30 [0.06; 1.46] | 0.30 [0.05; 1.85] | 0.34 [0.06; 1.99] | 0.34 [0.05; 2.30] | 0.42 [0.09; 1.97] | 0.41 [0.01; 14.32] | 0.47 [0.15; 1.49] | 0.49 [0.04; 6.28] | 0.53 [0.00; 58.69] | 0.57 [0.01; 62.87] | 0.55 [0.12; 2.50] | 0.62 [0.06; 6.52] | 0.61 [0.13; 2.89] | 0.67 [0.13; 3.54] | 0.79 [0.11; 5.63] | 0.86 [0.13; 5.87] | Inject_  semaglutide_  medium_dosage | . | . | 0.36 [0.01; 8.89] | 0.35 [0.01; 8.59] |
| 0.05 [0.00; 5.10] | 0.14 [0.00; 5.07] | 0.13 [0.00; 6.22] | 0.17 [0.01; 4.90] | 0.11 [0.00; 9.98] | 0.20 [0.01; 6.00] | 0.20 [0.01; 6.80] | 0.23 [0.01; 7.45] | 0.23 [0.01; 8.10] | 0.28 [0.01; 8.30] | 0.27 [0.00; 28.86] | 0.31 [0.01; 7.89] | 0.33 [0.01; 17.02] | 0.36 [0.01; 8.95] | 0.38 [0.02; 9.59] | 0.37 [0.01; 10.65] | 0.41 [0.01; 18.91] | 0.41 [0.01; 12.74] | 0.45 [0.01; 13.96] | 0.53 [0.01; 19.27] | 0.57 [0.02; 20.41] | 0.67 [0.02; 20.55] | Efpeglenatide_  medium_dosage | . | . | . |
| 0.05 [0.00; 2.09] | 0.14 [0.01; 1.46] | 0.13 [0.01; 1.97] | 0.17 [0.02; 1.25] | 0.11 [0.00; 4.08] | 0.20 [0.03; 1.57] | 0.20 [0.02; 1.90] | 0.23 [0.03; 2.06] | 0.23 [0.02; 2.32] | 0.28 [0.04; 2.14] | 0.27 [0.01; 12.14] | 0.32 [0.06; 1.80] | 0.33 [0.02; 5.80] | 0.36 [0.00; 47.14] | 0.38 [0.00; 50.50] | 0.37 [0.05; 2.73] | 0.42 [0.03; 6.14] | 0.41 [0.05; 3.32] | 0.45 [0.05; 3.74] | 0.53 [0.05; 5.62] | 0.58 [0.06; 5.90] | 0.67 [0.11; 4.28] | 1.00 [0.03; 39.28] | Tirzepatide_  medium_dosage | 1.16 [0.12; 11.27] | 0.51 [0.12; 2.10] |
| 0.05 [0.00; 2.06] | 0.14 [0.01; 1.48] | 0.12 [0.01; 1.94] | 0.16 [0.02; 1.29] | 0.10 [0.00; 4.03] | 0.19 [0.02; 1.61] | 0.19 [0.02; 1.93] | 0.21 [0.02; 2.10] | 0.22 [0.02; 2.36] | 0.27 [0.03; 2.20] | 0.26 [0.01; 11.98] | 0.30 [0.05; 1.88] | 0.31 [0.02; 5.81] | 0.34 [0.00; 45.99] | 0.36 [0.00; 49.27] | 0.34 [0.04; 2.81] | 0.39 [0.02; 6.18] | 0.38 [0.04; 3.37] | 0.42 [0.05; 3.83] | 0.50 [0.04; 5.71] | 0.54 [0.05; 5.99] | 0.63 [0.10; 4.09] | 0.94 [0.02; 38.80] | 0.94 [0.21; 4.28] | Tirzepatide_  low_dosage | 0.57 [0.10; 3.33] |
| 0.03 [0.00; 1.07] | ***0.08 [0.01; 0.70]** | ***0.07 [0.01; 0.97]** | ***0.10 [0.02; 0.59]** | 0.06 [0.00; 2.09] | ***0.11 [0.02; 0.74]** | ***0.11 [0.01; 0.91]** | ***0.13 [0.02; 0.99]** | 0.13 [0.02; 1.12] | 0.16 [0.02; 1.01] | 0.15 [0.00; 6.24] | ***0.18 [0.04; 0.83]** | 0.19 [0.01; 2.89] | 0.20 [0.00; 24.71] | 0.21 [0.00; 26.47] | 0.21 [0.03; 1.29] | 0.23 [0.02; 3.04] | 0.23 [0.03; 1.58] | 0.25 [0.04; 1.78] | 0.30 [0.03; 2.73] | 0.32 [0.04; 2.86] | 0.38 [0.07; 1.95] | 0.56 [0.02; 20.12] | 0.56 [0.17; 1.87] | 0.60 [0.15; 2.35] | Tirzepatide_  high_dosage |

Data presents as OR [95%CIs]. Pairwise (upper-right portion) and network (lower-left portion) meta-analysis results are presented as estimate effect sizes for the outcome of events of uterine tumor. Interventions are reported in order of mean ranking of beneficially prophylactic effect on events of uterine tumor, and outcomes are expressed as odds ratio (OR) (95% confidence intervals) (95%CIs). For the pairwise meta-analyses, OR of less than 1 indicates that the treatment specified in the row got more beneficial effect than that specified in the column. For the network meta-analysis (NMA), OR of less than 1 indicates that the treatment specified in the column got more beneficial effect than that specified in the row. Bold results marked with * indicate statistical significance.

**eTable 5B: League table of NMA of the primary outcome: subgroup of endometrial tumor**

| Liraglutide | . | . | . | . | . | . | . | . | . | . | . | 0.22 [0.02; 1.95] | . | . | . | . | . | . | . | . | . | . | . | . |
| --- | --- | --- | --- | --- | --- | --- | --- | --- | --- | --- | --- | --- | --- | --- | --- | --- | --- | --- | --- | --- | --- | --- | --- | --- |
| 1.27 [0.03; 60.12] | Dulaglutide_  low_dosage | . | . | . | . | . | . | . | . | 0.18 [0.01; 3.72] | . | . | . | . | . | . | . | . | . | . | . | . | . | . |
| 0.56 [0.05; 7.00] | 0.44 [0.01; 13.53] | Inject_  semaglutide_  high_dosage | . | . | . | . | . | . | . | . | . | 0.39 [0.11; 1.40] | . | . | . | . | . | . | . | . | . | . | . | . |
| 0.70 [0.01; 33.75] | 0.55 [0.01; 50.16] | 1.25 [0.04; 39.13] | Efpeglenatide_  medium_dosage | . | . | . | 0.36 [0.01; 8.95] | . | . | . | . | 0.32 [0.01; 7.76] | . | . | . | . | . | . | . | . | . | . | . | . |
| 0.67 [0.01; 32.42] | 0.53 [0.01; 48.19] | 1.20 [0.04; 37.58] | 0.96 [0.01; 89.13] | Oral_  semaglutide | . | . | . | . | . | . | . | 0.33 [0.01; 8.07] | . | . | . | . | . | . | . | . | . | . | . | . |
| 0.56 [0.01; 23.41] | 0.44 [0.01; 35.48] | 0.99 [0.04; 26.67] | 0.79 [0.01; 65.66] | 0.83 [0.01; 68.31] | Inject_  semaglutide_  low_dosage | . | . | . | . | . | . | 0.40 [0.02; 8.30] | . | . | . | . | . | . | . | . | . | . | . | . |
| 0.33 [0.03; 3.44] | 0.26 [0.01; 7.00] | 0.60 [0.13; 2.70] | 0.48 [0.02; 13.04] | 0.50 [0.02; 13.57] | 0.60 [0.03; 14.02] | Dapagliflozin_  high_dosage | . | . | . | . | . | 0.66 [0.29; 1.50] | . | . | . | . | . | . | . | . | . | . | . | . |
| 0.25 [0.01; 5.91] | 0.20 [0.00; 9.92] | 0.45 [0.03; 6.09] | 0.36 [0.01; 8.95] | 0.38 [0.01; 19.14] | 0.46 [0.01; 20.30] | 0.76 [0.07; 8.48] | Efpeglenatide_  high_dosage | . | . | . | . | 0.87 [0.09; 8.37] | . | . | . | . | . | . | . | . | . | . | . | . |
| 0.25 [0.01; 4.29] | 0.19 [0.00; 7.63] | 0.44 [0.05; 4.13] | 0.35 [0.01; 14.17] | 0.36 [0.01; 14.75] | 0.44 [0.01; 15.51] | 0.73 [0.10; 5.56] | 0.96 [0.05; 18.01] | Albiglutide | . | . | . | 0.90 [0.14; 5.75] | . | . | . | . | . | . | . | . | . | . | . | . |
| 0.23 [0.01; 6.80] | 0.18 [0.00; 10.90] | 0.41 [0.02; 7.34] | 0.33 [0.01; 20.21] | 0.34 [0.01; 21.02] | 0.41 [0.01; 22.44] | 0.69 [0.05; 10.42] | 0.90 [0.03; 28.25] | 0.93 [0.04; 22.69] | Canagliflozin_  high_dosage | . | 0.97 [0.06; 15.55] | 0.97 [0.06; 15.61] | . | . | . | . | . | . | . | . | . | . | . | . |
| 0.22 [0.02; 2.34] | 0.18 [0.01; 3.72] | 0.40 [0.08; 1.86] | 0.32 [0.01; 8.82] | 0.33 [0.01; 9.18] | 0.40 [0.02; 9.49] | 0.67 [0.20; 2.22] | 0.88 [0.08; 9.96] | 0.91 [0.12; 7.07] | 0.97 [0.06; 15.07] | Dulaglutide_  medium_dosage | . | 0.99 [0.41; 2.38] | . | . | . | . | . | . | . | . | . | . | . | . |
| 0.22 [0.01; 4.22] | 0.18 [0.00; 7.40] | 0.40 [0.04; 4.14] | 0.32 [0.01; 13.74] | 0.33 [0.01; 14.29] | 0.40 [0.01; 15.06] | 0.67 [0.08; 5.63] | 0.88 [0.04; 17.70] | 0.92 [0.06; 13.63] | 0.98 [0.07; 13.14] | 1.01 [0.12; 8.65] | Canagliflozin_  low_dosage | 0.98 [0.14; 6.98] | . | . | . | . | . | . | . | . | . | . | . | . |
| 0.22 [0.02; 1.95] | 0.17 [0.01; 4.17] | 0.39 [0.11; 1.40] | 0.32 [0.01; 7.76] | 0.33 [0.01; 8.07] | 0.40 [0.02; 8.30] | 0.66 [0.29; 1.50] | 0.87 [0.09; 8.37] | 0.90 [0.14; 5.75] | 0.96 [0.07; 12.91] | 0.99 [0.41; 2.38] | 0.98 [0.14; 6.98] | Placebo_  or_Control | 0.99 [0.06; 15.79] | . | 0.98 [0.14; 6.93] | 2.31 [0.11; 48.31] | 0.64 [0.03; 15.78] | 0.57 [0.09; 3.52] | 0.47 [0.04; 5.24] | 0.34 [0.03; 3.30] | 0.33 [0.01; 8.05] | 0.32 [0.03; 3.10] | 0.40 [0.12; 1.27] | 0.32 [0.01; 8.00] |
| 0.22 [0.01; 7.41] | 0.17 [0.00; 11.61] | 0.39 [0.02; 8.19] | 0.31 [0.00; 21.51] | 0.32 [0.00; 22.38] | 0.39 [0.01; 23.98] | 0.65 [0.04; 11.73] | 0.86 [0.02; 30.74] | 0.89 [0.03; 24.95] | 0.95 [0.02; 42.37] | 0.98 [0.05; 17.90] | 0.97 [0.03; 28.92] | 0.99 [0.06; 15.79] | Ertugliflozin_  high_dosage | . | . | . | . | . | 0.48 [0.04; 5.31] | . | . | . | . | . |
| 0.21 [0.01; 5.05] | 0.16 [0.00; 8.42] | 0.37 [0.03; 5.25] | 0.30 [0.01; 15.61] | 0.31 [0.01; 16.24] | 0.38 [0.01; 17.24] | 0.63 [0.05; 7.33] | 0.82 [0.03; 21.08] | 0.85 [0.04; 16.64] | 0.91 [0.03; 29.68] | 0.94 [0.08; 11.22] | 0.93 [0.04; 19.44] | 0.95 [0.09; 9.65] | 0.96 [0.03; 35.77] | Tirzepatide_  medium_dosage | . | . | . | . | . | 0.34 [0.03; 3.31] | . | . | . | 0.36 [0.04; 3.43] |
| 0.22 [0.01; 4.04] | 0.17 [0.00; 7.08] | 0.38 [0.04; 3.96] | 0.31 [0.01; 13.15] | 0.32 [0.01; 13.68] | 0.39 [0.01; 14.42] | 0.64 [0.08; 5.39] | 0.85 [0.04; 16.94] | 0.88 [0.06; 13.04] | 0.94 [0.04; 24.29] | 0.97 [0.11; 8.27] | 0.96 [0.06; 15.34] | 0.98 [0.14; 6.93] | 0.99 [0.03; 29.54] | 1.03 [0.05; 21.47] | Sotagliflozin | . | . | . | . | . | . | . | . | . |
| 0.19 [0.01; 3.43] | 0.15 [0.00; 6.08] | 0.34 [0.04; 3.31] | 0.28 [0.01; 11.30] | 0.29 [0.01; 11.75] | 0.35 [0.01; 12.37] | 0.58 [0.07; 4.47] | 0.76 [0.04; 14.39] | 0.79 [0.06; 11.01] | 0.84 [0.03; 20.72] | 0.87 [0.11; 6.87] | 0.86 [0.06; 12.97] | 0.88 [0.13; 5.70] | 0.89 [0.03; 25.26] | 0.93 [0.06; 13.36] | 0.90 [0.06; 13.53] | Inject_  semaglutide_  medium_dosage | . | . | . | . | . | . | 0.96 [0.06; 15.41] | 0.36 [0.01; 8.89] |
| 0.14 [0.00; 6.82] | 0.11 [0.00; 10.13] | 0.25 [0.01; 7.90] | 0.20 [0.00; 18.74] | 0.21 [0.00; 19.49] | 0.25 [0.00; 21.06] | 0.42 [0.02; 11.54] | 0.55 [0.01; 28.13] | 0.58 [0.01; 23.36] | 0.62 [0.01; 38.09] | 0.63 [0.02; 17.58] | 0.63 [0.01; 26.93] | 0.64 [0.03; 15.78] | 0.65 [0.01; 44.98] | 0.67 [0.01; 35.30] | 0.66 [0.02; 28.11] | 0.73 [0.02; 29.86] | Bexagliflozin | . | . | . | . | . | . | . |
| 0.12 [0.01; 1.89] | 0.09 [0.00; 3.44] | 0.21 [0.03; 1.78] | 0.17 [0.00; 6.39] | 0.18 [0.00; 6.64] | 0.21 [0.01; 6.97] | 0.35 [0.05; 2.36] | 0.47 [0.03; 7.96] | 0.48 [0.04; 6.02] | 0.52 [0.02; 11.56] | 0.53 [0.08; 3.63] | 0.53 [0.04; 7.11] | 0.54 [0.10; 2.97] | 0.54 [0.02; 14.15] | 0.57 [0.03; 10.11] | 0.55 [0.04; 7.42] | 0.61 [0.05; 7.72] | 0.84 [0.02; 31.75] | Empagliflozin_  low_dosage | . | . | . | 0.95 [0.10; 9.18] | . | . |
| 0.10 [0.00; 2.68] | 0.08 [0.00; 4.42] | 0.19 [0.01; 2.82] | 0.15 [0.00; 8.19] | 0.16 [0.00; 8.52] | 0.19 [0.00; 9.06] | 0.31 [0.02; 3.96] | 0.41 [0.02; 11.18] | 0.43 [0.02; 8.87] | 0.46 [0.01; 15.68] | 0.47 [0.04; 6.06] | 0.47 [0.02; 10.35] | 0.47 [0.04; 5.24] | 0.48 [0.04; 5.31] | 0.50 [0.02; 14.11] | 0.49 [0.02; 10.80] | 0.54 [0.03; 11.36] | 0.74 [0.01; 40.71] | 0.88 [0.05; 16.88] | Ertugliflozin_  low_dosage | . | . | . | . | . |
| 0.10 [0.01; 1.65] | 0.08 [0.00; 2.94] | 0.17 [0.02; 1.58] | 0.14 [0.00; 5.46] | 0.14 [0.00; 5.68] | 0.17 [0.00; 5.98] | 0.29 [0.04; 2.13] | 0.38 [0.02; 6.92] | 0.39 [0.03; 5.28] | 0.42 [0.02; 9.99] | 0.43 [0.06; 3.27] | 0.42 [0.03; 6.22] | 0.43 [0.07; 2.70] | 0.44 [0.02; 12.19] | 0.46 [0.08; 2.77] | 0.44 [0.03; 6.49] | 0.49 [0.05; 5.19] | 0.68 [0.02; 27.15] | 0.81 [0.07; 9.90] | 0.91 [0.04; 18.73] | Tirzepatide_  high_dosage | . | . | . | 0.34 [0.04; 3.26] |
| 0.07 [0.00; 3.48] | 0.06 [0.00; 5.18] | 0.13 [0.00; 4.04] | 0.10 [0.00; 9.57] | 0.11 [0.00; 9.96] | 0.13 [0.00; 10.76] | 0.22 [0.01; 5.89] | 0.28 [0.01; 14.37] | 0.30 [0.01; 11.93] | 0.32 [0.01; 19.46] | 0.32 [0.01; 8.97] | 0.32 [0.01; 13.75] | 0.33 [0.01; 8.05] | 0.33 [0.00; 22.98] | 0.35 [0.01; 18.03] | 0.34 [0.01; 14.35] | 0.37 [0.01; 15.25] | 0.51 [0.01; 47.57] | 0.61 [0.02; 23.05] | 0.69 [0.01; 37.86] | 0.76 [0.02; 30.32] | Lixisenatide | . | . | . |
| 0.09 [0.00; 1.71] | 0.07 [0.00; 2.99] | 0.16 [0.02; 1.68] | 0.13 [0.00; 5.55] | 0.13 [0.00; 5.77] | 0.16 [0.00; 6.08] | 0.27 [0.03; 2.29] | 0.35 [0.02; 7.16] | 0.37 [0.02; 5.52] | 0.39 [0.01; 10.26] | 0.40 [0.05; 3.52] | 0.40 [0.02; 6.49] | 0.41 [0.06; 2.95] | 0.41 [0.01; 12.47] | 0.43 [0.02; 9.08] | 0.42 [0.03; 6.78] | 0.46 [0.03; 7.08] | 0.64 [0.01; 27.57] | 0.76 [0.11; 5.33] | 0.86 [0.04; 19.33] | 0.94 [0.06; 13.98] | 1.24 [0.03; 53.62] | Empagliflozin_  high_dosage | . | . |
| 0.10 [0.01; 1.13] | 0.08 [0.00; 2.23] | ***0.17 [0.03; 0.94]** | 0.14 [0.00; 4.15] | 0.15 [0.00; 4.31] | 0.18 [0.01; 4.48] | 0.29 [0.07; 1.16] | 0.38 [0.03; 4.80] | 0.40 [0.05; 3.46] | 0.43 [0.03; 7.18] | 0.44 [0.11; 1.80] | 0.44 [0.05; 4.14] | 0.44 [0.15; 1.34] | 0.45 [0.02; 8.91] | 0.47 [0.04; 5.82] | 0.45 [0.05; 4.32] | 0.51 [0.07; 3.45] | 0.69 [0.02; 20.61] | 0.83 [0.11; 6.35] | 0.94 [0.07; 13.18] | 1.03 [0.13; 8.33] | 1.35 [0.05; 40.08] | 1.09 [0.11; 10.53] | Exenatide | . |
| ***0.06 [0.00; 0.99]** | 0.04 [0.00; 1.75] | ***0.10 [0.01; 0.96]** | 0.08 [0.00; 3.26] | 0.08 [0.00; 3.39] | 0.10 [0.00; 3.57] | 0.17 [0.02; 1.30] | 0.22 [0.01; 4.16] | 0.23 [0.02; 3.18] | 0.24 [0.01; 5.98] | 0.25 [0.03; 1.99] | 0.25 [0.02; 3.75] | 0.25 [0.04; 1.66] | 0.25 [0.01; 7.29] | 0.26 [0.04; 1.60] | 0.26 [0.02; 3.91] | 0.29 [0.03; 2.60] | 0.39 [0.01; 16.20] | 0.47 [0.04; 5.98] | 0.53 [0.02; 11.24] | 0.58 [0.11; 2.94] | 0.77 [0.02; 31.51] | 0.62 [0.04; 9.50] | 0.57 [0.07; 4.68] | Tirzepatide_  low_dosage |

Data presents as OR [95%CIs]. Pairwise (upper-right portion) and network (lower-left portion) meta-analysis results are presented as estimate effect sizes for the outcome of events of endometrial tumor. Interventions are reported in order of mean ranking of beneficially prophylactic effect on events of endometrial tumor, and outcomes are expressed as odds ratio (OR) (95% confidence intervals) (95%CIs). For the pairwise meta-analyses, OR of less than 1 indicates that the treatment specified in the row got more beneficial effect than that specified in the column. For the network meta-analysis (NMA), OR of less than 1 indicates that the treatment specified in the column got more beneficial effect than that specified in the row. Bold results marked with * indicate statistical significance.

**eTable 5C: League table of the acceptability: drop-out rate (the outcome of drop-out rate here was calculated according to drop-out rate data from the original composition of subjects from included randomized controlled trials because there were no any studies provided specific information regarding drop-out rate data in female subgroup)**

| Tirzepatide_  low_dosage | . | . | . | 0.94 [0.71; 1.25] | . | 0.83 [0.63; 1.10] | . | . | 0.72 [0.35; 1.46] | . | . | . | . | . | . | . | . | . | . | . | . | . | . | . | . | . | ***0.67 [0.50; 0.90]** | . |
| --- | --- | --- | --- | --- | --- | --- | --- | --- | --- | --- | --- | --- | --- | --- | --- | --- | --- | --- | --- | --- | --- | --- | --- | --- | --- | --- | --- | --- |
| 1.17 [0.52; 2.62] | Dulaglutide_  high_dosage | . | . | . | . | . | . | . | . | . | . | . | . | . | . | . | . | . | . | . | 0.69 [0.31; 1.51] | 0.62 [0.28; 1.38] | . | . | . | . | ***0.42 [0.19; 0.95]** | . |
| 0.96 [0.68; 1.37] | 0.82 [0.37; 1.82] | Canagliflozin_  high_dosage | 0.94 [0.69; 1.28] | . | . | . | . | . | . | . | . | . | . | . | . | . | . | . | . | . | . | . | . | . | . | . | ***0.67 [0.53; 0.85]** | . |
| 0.94 [0.64; 1.37] | 0.80 [0.36; 1.80] | 0.97 [0.74; 1.29] | Canagliflozin_  low_dosage | . | . | . | . | . | . | . | . | . | . | . | . | . | . | . | . | . | . | . | . | . | . | . | ***0.67 [0.50; 0.89]** | . |
| 0.88 [0.67; 1.16] | 0.75 [0.34; 1.68] | 0.92 [0.66; 1.28] | 0.94 [0.66; 1.35] | Tirzepatide_  high_dosage | . | 0.97 [0.76; 1.24] | . | . | 0.92 [0.46; 1.81] | . | . | . | . | . | . | . | . | . | . | . | . | . | . | . | . | . | ***0.67 [0.52; 0.87]** | . |
| 0.88 [0.62; 1.24] | 0.75 [0.34; 1.66] | 0.91 [0.66; 1.26] | 0.94 [0.66; 1.33] | 1.00 [0.72; 1.38] | Inject_  semaglutide_  high_dosage | . | . | . | 1.00 [0.42; 2.37] | . | . | . | . | . | . | . | . | . | . | 0.66 [0.21; 2.02] | . | . | . | . | . | . | ***0.73 [0.58; 0.92]** | . |
| 0.87 [0.66; 1.14] | 0.74 [0.33; 1.65] | 0.90 [0.65; 1.26] | 0.92 [0.64; 1.33] | 0.98 [0.77; 1.25] | 0.99 [0.71; 1.37] | Tirzepatide_  medium_dosage | . | . | 1.04 [0.53; 2.03] | . | . | . | . | . | . | . | . | . | . | . | . | . | . | . | . | . | ***0.73 [0.56; 0.94]** | . |
| 0.88 [0.56; 1.36] | 0.75 [0.32; 1.74] | 0.91 [0.59; 1.40] | 0.93 [0.59; 1.47] | 0.99 [0.65; 1.52] | 1.00 [0.65; 1.52] | 1.01 [0.66; 1.55] | Inject_  semaglutide_  low_dosage | . | 0.95 [0.58; 1.56] | . | . | . | . | . | . | . | . | . | . | 0.92 [0.37; 2.27] | . | . | . | . | . | . | 0.75 [0.50; 1.11] | . |
| 0.87 [0.53; 1.42] | 0.74 [0.31; 1.77] | 0.90 [0.56; 1.45] | 0.93 [0.57; 1.52] | 0.99 [0.61; 1.59] | 0.99 [0.62; 1.58] | 1.00 [0.62; 1.62] | 0.99 [0.57; 1.72] | Dapagliflozin_  low_dosage | . | . | . | . | . | 0.93 [0.60; 1.47] | . | . | . | . | 0.82 [0.53; 1.28] | . | . | . | . | . | . | . | 0.70 [0.44; 1.09] | . |
| 0.85 [0.60; 1.21] | 0.73 [0.33; 1.63] | 0.89 [0.62; 1.26] | 0.91 [0.62; 1.33] | 0.97 [0.69; 1.35] | 0.97 [0.69; 1.36] | 0.98 [0.71; 1.37] | 0.97 [0.66; 1.45] | 0.98 [0.60; 1.60] | Inject_  semaglutide_  medium_dosage | . | . | . | . | . | . | . | . | . | . | . | . | . | . | . | . | . | ***0.69 [0.49; 0.97]** | 0.83 [0.45; 1.54] |
| 0.80 [0.52; 1.24] | 0.68 [0.30; 1.58] | 0.83 [0.54; 1.27] | 0.85 [0.55; 1.33] | 0.91 [0.59; 1.39] | 0.91 [0.60; 1.38] | 0.92 [0.60; 1.41] | 0.91 [0.55; 1.51] | 0.92 [0.53; 1.58] | 0.94 [0.60; 1.46] | Ertugliflozin_  low_dosage | . | . | . | . | . | . | . | 0.89 [0.63; 1.28] | . | . | . | . | . | . | . | . | 0.80 [0.56; 1.13] | . |
| 0.81 [0.47; 1.39] | 0.69 [0.28; 1.69] | 0.84 [0.49; 1.42] | 0.86 [0.49; 1.49] | 0.91 [0.53; 1.56] | 0.92 [0.54; 1.55] | 0.93 [0.54; 1.58] | 0.92 [0.50; 1.67] | 0.92 [0.49; 1.74] | 0.94 [0.55; 1.63] | 1.01 [0.55; 1.82] | Efpeglenatide_  low_dosage | . | . | . | . | . | . | . | . | . | . | . | . | 0.84 [0.49; 1.43] | 0.84 [0.49; 1.43] | . | 0.77 [0.45; 1.32] | . |
| 0.78 [0.57; 1.06] | 0.66 [0.30; 1.44] | 0.80 [0.60; 1.07] | 0.83 [0.60; 1.13] | 0.88 [0.65; 1.17] | 0.88 [0.67; 1.16] | 0.89 [0.67; 1.19] | 0.88 [0.59; 1.32] | 0.89 [0.57; 1.39] | 0.91 [0.66; 1.24] | 0.97 [0.66; 1.43] | 0.96 [0.58; 1.60] | Empagliflozin_  low_dosage | 0.96 [0.74; 1.24] | . | . | . | . | . | . | . | . | . | . | . | . | . | ***0.81 [0.68; 0.96]** | . |
| 0.77 [0.55; 1.07] | 0.66 [0.30; 1.44] | 0.80 [0.59; 1.09] | 0.82 [0.58; 1.15] | 0.87 [0.64; 1.19] | 0.87 [0.65; 1.18] | 0.89 [0.65; 1.21] | 0.88 [0.58; 1.33] | 0.88 [0.56; 1.40] | 0.90 [0.65; 1.26] | 0.96 [0.64; 1.44] | 0.96 [0.57; 1.61] | 0.99 [0.80; 1.24] | Empagliflozin_  high_dosage | . | . | . | . | . | . | . | . | . | . | . | . | 2.16 [0.95; 4.91] | ***0.73 [0.59; 0.91]** | . |
| 0.76 [0.53; 1.10] | 0.65 [0.29; 1.46] | 0.79 [0.56; 1.12] | 0.81 [0.56; 1.18] | 0.86 [0.61; 1.23] | 0.87 [0.62; 1.22] | 0.88 [0.62; 1.25] | 0.87 [0.56; 1.36] | 0.88 [0.58; 1.32] | 0.90 [0.62; 1.29] | 0.95 [0.62; 1.47] | 0.95 [0.55; 1.63] | 0.99 [0.73; 1.34] | 0.99 [0.72; 1.38] | Dapagliflozin_  high_dosage | . | . | . | . | 0.87 [0.57; 1.34] | . | . | . | . | . | . | . | 0.83 [0.64; 1.08] | . |
| 0.75 [0.54; 1.06] | 0.64 [0.29; 1.42] | 0.78 [0.57; 1.07] | 0.80 [0.57; 1.13] | 0.85 [0.62; 1.17] | 0.86 [0.63; 1.16] | 0.87 [0.63; 1.19] | 0.86 [0.57; 1.30] | 0.87 [0.55; 1.37] | 0.88 [0.63; 1.24] | 0.94 [0.63; 1.42] | 0.94 [0.56; 1.58] | 0.97 [0.75; 1.27] | 0.98 [0.73; 1.31] | 0.99 [0.71; 1.37] | Albiglutide | . | . | . | . | . | . | . | . | . | . | . | 0.85 [0.69; 1.04] | . |
| 0.75 [0.53; 1.05] | 0.64 [0.29; 1.41] | 0.78 [0.57; 1.06] | 0.80 [0.56; 1.12] | 0.85 [0.61; 1.16] | 0.85 [0.62; 1.16] | 0.86 [0.63; 1.18] | 0.85 [0.56; 1.30] | 0.86 [0.54; 1.36] | 0.88 [0.62; 1.23] | 0.93 [0.62; 1.41] | 0.93 [0.55; 1.57] | 0.96 [0.74; 1.26] | 0.97 [0.73; 1.30] | 0.98 [0.70; 1.36] | 0.99 [0.74; 1.33] | Sotagliflozin | . | . | . | . | . | . | . | . | . | . | 0.85 [0.69; 1.05] | . |
| 0.73 [0.37; 1.43] | 0.62 [0.23; 1.66] | 0.76 [0.39; 1.47] | 0.78 [0.39; 1.53] | 0.82 [0.42; 1.61] | 0.83 [0.43; 1.61] | 0.84 [0.43; 1.64] | 0.83 [0.40; 1.71] | 0.83 [0.40; 1.76] | 0.85 [0.43; 1.68] | 0.91 [0.44; 1.86] | 0.90 [0.41; 1.99] | 0.94 [0.49; 1.79] | 0.95 [0.49; 1.82] | 0.95 [0.49; 1.87] | 0.97 [0.50; 1.86] | 0.97 [0.50; 1.88] | Bexagliflozin | . | . | . | . | . | . | . | . | . | 0.88 [0.47; 1.64] | . |
| 0.72 [0.46; 1.11] | 0.61 [0.27; 1.42] | 0.75 [0.49; 1.14] | 0.77 [0.49; 1.19] | 0.81 [0.53; 1.24] | 0.82 [0.54; 1.24] | 0.83 [0.54; 1.26] | 0.82 [0.50; 1.35] | 0.82 [0.48; 1.41] | 0.84 [0.54; 1.31] | 0.90 [0.63; 1.28] | 0.89 [0.49; 1.61] | 0.93 [0.63; 1.36] | 0.93 [0.62; 1.40] | 0.94 [0.61; 1.45] | 0.95 [0.64; 1.43] | 0.96 [0.64; 1.45] | 0.99 [0.48; 2.02] | Ertugliflozin_  high_dosage | . | . | . | . | . | . | . | . | 0.89 [0.63; 1.26] | . |
| 0.71 [0.45; 1.15] | 0.61 [0.26; 1.44] | 0.74 [0.47; 1.17] | 0.76 [0.47; 1.22] | 0.81 [0.51; 1.28] | 0.81 [0.52; 1.28] | 0.82 [0.52; 1.30] | 0.81 [0.48; 1.39] | 0.82 [0.53; 1.27] | 0.84 [0.52; 1.34] | 0.89 [0.53; 1.51] | 0.89 [0.48; 1.65] | 0.92 [0.60; 1.41] | 0.93 [0.60; 1.44] | 0.93 [0.63; 1.38] | 0.95 [0.61; 1.48] | 0.96 [0.61; 1.49] | 0.98 [0.47; 2.05] | 0.99 [0.59; 1.68] | Dapagliflozin_  medium_dosage | . | . | . | . | . | . | . | 0.83 [0.53; 1.28] | . |
| ***0.72 [0.53; 0.99]** | 0.62 [0.28; 1.35] | 0.75 [0.56; 1.01] | 0.77 [0.56; 1.06] | 0.82 [0.61; 1.10] | 0.82 [0.62; 1.09] | 0.83 [0.62; 1.12] | 0.83 [0.56; 1.22] | 0.83 [0.53; 1.30] | 0.85 [0.62; 1.16] | 0.90 [0.61; 1.34] | 0.90 [0.54; 1.50] | 0.93 [0.73; 1.19] | 0.94 [0.72; 1.23] | 0.95 [0.69; 1.29] | 0.96 [0.73; 1.26] | 0.97 [0.74; 1.28] | 0.99 [0.52; 1.90] | 1.01 [0.68; 1.49] | 1.01 [0.66; 1.56] | Liraglutide | . | . | . | . | . | 1.26 [0.46; 3.49] | 0.87 [0.73; 1.04] | . |
| 0.70 [0.48; 1.03] | 0.60 [0.28; 1.27] | 0.73 [0.50; 1.05] | 0.75 [0.50; 1.10] | 0.79 [0.55; 1.15] | 0.80 [0.56; 1.14] | 0.81 [0.56; 1.17] | 0.80 [0.50; 1.26] | 0.80 [0.49; 1.32] | 0.82 [0.56; 1.21] | 0.87 [0.56; 1.37] | 0.87 [0.50; 1.52] | 0.90 [0.65; 1.25] | 0.91 [0.64; 1.29] | 0.92 [0.63; 1.34] | 0.93 [0.65; 1.32] | 0.94 [0.66; 1.33] | 0.96 [0.48; 1.91] | 0.97 [0.62; 1.53] | 0.98 [0.60; 1.59] | 0.97 [0.69; 1.35] | Dulaglutide_  low_dosage | 0.99 [0.75; 1.31] | . | . | . | . | 0.91 [0.68; 1.21] | . |
| 0.69 [0.47; 1.01] | 0.59 [0.28; 1.26] | 0.72 [0.50; 1.02] | 0.73 [0.50; 1.08] | 0.78 [0.54; 1.12] | 0.78 [0.55; 1.11] | 0.79 [0.55; 1.14] | 0.79 [0.50; 1.24] | 0.79 [0.48; 1.30] | 0.81 [0.55; 1.18] | 0.86 [0.55; 1.34] | 0.86 [0.49; 1.49] | 0.89 [0.65; 1.22] | 0.90 [0.64; 1.26] | 0.90 [0.62; 1.31] | 0.91 [0.65; 1.29] | 0.92 [0.65; 1.30] | 0.95 [0.48; 1.87] | 0.96 [0.62; 1.49] | 0.97 [0.60; 1.56] | 0.95 [0.69; 1.32] | 0.99 [0.75; 1.30] | Dulaglutide_  medium_dosage | . | . | . | . | 0.92 [0.70; 1.21] | . |
| 0.67 [0.39; 1.14] | 0.57 [0.23; 1.39] | 0.69 [0.41; 1.16] | 0.71 [0.42; 1.22] | 0.76 [0.45; 1.27] | 0.76 [0.46; 1.27] | 0.77 [0.46; 1.29] | 0.76 [0.42; 1.37] | 0.77 [0.41; 1.42] | 0.78 [0.46; 1.33] | 0.84 [0.47; 1.49] | 0.83 [0.43; 1.62] | 0.86 [0.53; 1.41] | 0.87 [0.53; 1.44] | 0.88 [0.52; 1.48] | 0.89 [0.54; 1.47] | 0.90 [0.54; 1.49] | 0.92 [0.42; 2.00] | 0.93 [0.52; 1.66] | 0.94 [0.51; 1.72] | 0.92 [0.56; 1.51] | 0.96 [0.56; 1.64] | 0.97 [0.57; 1.65] | Lixisenatide | . | . | . | 0.95 [0.60; 1.51] | . |
| 0.67 [0.44; 1.02] | 0.57 [0.25; 1.31] | 0.70 [0.47; 1.04] | 0.71 [0.47; 1.09] | 0.76 [0.51; 1.14] | 0.76 [0.51; 1.13] | 0.77 [0.51; 1.16] | 0.76 [0.47; 1.24] | 0.77 [0.45; 1.30] | 0.78 [0.52; 1.20] | 0.84 [0.52; 1.35] | 0.83 [0.52; 1.34] | 0.86 [0.60; 1.25] | 0.87 [0.59; 1.28] | 0.88 [0.58; 1.33] | 0.89 [0.60; 1.31] | 0.90 [0.61; 1.32] | 0.92 [0.45; 1.86] | 0.93 [0.58; 1.50] | 0.94 [0.56; 1.56] | 0.93 [0.64; 1.34] | 0.96 [0.62; 1.47] | 0.97 [0.63; 1.48] | 1.00 [0.57; 1.76] | Efpeglenatide_  medium_dosage | 0.99 [0.71; 1.37] | . | 0.95 [0.69; 1.32] | . |
| 0.66 [0.43; 1.01] | 0.57 [0.25; 1.29] | 0.69 [0.46; 1.03] | 0.70 [0.46; 1.08] | 0.75 [0.50; 1.12] | 0.75 [0.51; 1.12] | 0.76 [0.51; 1.14] | 0.75 [0.46; 1.23] | 0.76 [0.45; 1.28] | 0.78 [0.51; 1.18] | 0.83 [0.51; 1.34] | 0.82 [0.51; 1.33] | 0.85 [0.59; 1.23] | 0.86 [0.59; 1.26] | 0.87 [0.57; 1.31] | 0.88 [0.60; 1.29] | 0.89 [0.60; 1.31] | 0.91 [0.45; 1.84] | 0.92 [0.57; 1.48] | 0.93 [0.56; 1.54] | 0.91 [0.63; 1.33] | 0.94 [0.61; 1.46] | 0.96 [0.63; 1.47] | 0.99 [0.56; 1.74] | 0.99 [0.71; 1.37] | Efpeglenatide_  high_dosage | . | 0.96 [0.70; 1.34] | . |
| ***0.62 [0.40; 0.95]** | 0.53 [0.23; 1.21] | ***0.64 [0.42; 0.97]** | 0.66 [0.42; 1.02] | 0.70 [0.46; 1.06] | 0.70 [0.46; 1.06] | 0.71 [0.47; 1.08] | 0.70 [0.43; 1.16] | 0.71 [0.41; 1.21] | 0.72 [0.47; 1.11] | 0.77 [0.47; 1.26] | 0.76 [0.42; 1.38] | 0.79 [0.54; 1.16] | 0.80 [0.55; 1.17] | 0.81 [0.52; 1.24] | 0.82 [0.55; 1.22] | 0.82 [0.55; 1.23] | 0.85 [0.41; 1.73] | 0.86 [0.52; 1.40] | 0.86 [0.51; 1.45] | 0.85 [0.58; 1.25] | 0.88 [0.56; 1.37] | 0.89 [0.58; 1.38] | 0.92 [0.52; 1.64] | 0.92 [0.57; 1.48] | 0.93 [0.58; 1.50] | Oral_  semaglutide | 1.29 [0.88; 1.91] | . |
| ***0.64 [0.49; 0.83]** | 0.54 [0.25; 1.17] | ***0.66 [0.52; 0.84]** | ***0.68 [0.52; 0.89]** | ***0.72 [0.57; 0.92]** | ***0.73 [0.58; 0.91]** | ***0.73 [0.58; 0.93]** | 0.73 [0.51; 1.04] | 0.73 [0.48; 1.10] | ***0.75 [0.57; 0.97]** | 0.80 [0.56; 1.13] | 0.79 [0.49; 1.28] | ***0.82 [0.70; 0.97]** | 0.83 [0.68; 1.01] | 0.83 [0.65; 1.08] | 0.85 [0.69; 1.04] | 0.85 [0.69; 1.05] | 0.88 [0.47; 1.64] | 0.89 [0.63; 1.26] | 0.89 [0.60; 1.32] | 0.88 [0.74; 1.05] | 0.91 [0.69; 1.21] | 0.92 [0.70; 1.21] | 0.95 [0.60; 1.51] | 0.95 [0.69; 1.32] | 0.96 [0.70; 1.34] | 1.04 [0.73; 1.46] | Placebo_  or_Control | 0.90 [0.66; 1.22] |
| ***0.60 [0.41; 0.87]** | 0.51 [0.23; 1.15] | ***0.62 [0.43; 0.89]** | ***0.64 [0.43; 0.94]** | ***0.68 [0.47; 0.97]** | ***0.68 [0.48; 0.97]** | ***0.69 [0.48; 0.99]** | 0.68 [0.44; 1.06] | 0.68 [0.42; 1.13] | ***0.70 [0.49; 0.99]** | 0.75 [0.48; 1.17] | 0.74 [0.43; 1.29] | 0.77 [0.56; 1.06] | 0.78 [0.55; 1.09] | 0.78 [0.54; 1.14] | 0.79 [0.56; 1.12] | 0.80 [0.56; 1.13] | 0.82 [0.41; 1.62] | 0.83 [0.53; 1.30] | 0.84 [0.52; 1.35] | 0.82 [0.59; 1.15] | 0.85 [0.57; 1.27] | 0.87 [0.59; 1.28] | 0.89 [0.52; 1.53] | 0.89 [0.58; 1.37] | 0.90 [0.59; 1.39] | 0.97 [0.62; 1.51] | 0.94 [0.71; 1.24] | Exenatide |

Data presents as OR [95%CIs]. Pairwise (upper-right portion) and network (lower-left portion) meta-analysis results are presented as estimate effect sizes for the outcome of acceptability (drop-out rate). Interventions are reported in order of mean ranking of acceptability, and outcomes are expressed as odds ratio (OR) (95% confidence intervals) (95%CIs). For the pairwise meta-analyses, OR of less than 1 indicates that the treatment specified in the row got more acceptability than that specified in the column. For the network meta-analysis (NMA), OR of less than 1 indicates that the treatment specified in the column got more acceptability than that specified in the row. Bold results marked with * indicate statistical significance.

*Abbreviation: 95%CIs: 95% confidence intervals; GLP-1 agonist: glucagon-like peptide-1 agonist; NMA: network meta-analysis; OR: odds ratio; RCT: randomized controlled trial; SGLT2 inhibitor: sodium–glucose cotransporter 2 inhibitor*

**eTable 6A: SUCRA (Surface under the cumulative ranking) of primary outcome: overall gynecologic tumor**

| Treatment | Rank 1 | Rank 2 | Rank 3 | Rank 4 | Rank 5 | Rank 6 | Rank 7 | Rank 8 | Rank 9 | Rank 10 | Rank 11 | Rank 12 | Rank 13 | Rank 14 | Rank 15 | Rank 16 | Rank 17 | Rank 18 | Rank 19 | Rank 20 | Rank 21 | Rank 22 | Rank 23 | Rank 24 | Rank 25 | Rank 26 | Rank 27 | Rank 28 | Rank 29 | SUCRA |
| --- | --- | --- | --- | --- | --- | --- | --- | --- | --- | --- | --- | --- | --- | --- | --- | --- | --- | --- | --- | --- | --- | --- | --- | --- | --- | --- | --- | --- | --- | --- |
| Dapagliflozin_medium_dosage | 0.4582875 | 0.4702625 | 0.0208375 | 0.010575 | 0.0085625 | 0.00525 | 0.0041 | 0.0026 | 0.002275 | 0.002175 | 0.0019 | 0.001825 | 0.0010625 | 0.0011125 | 0.00105 | 0.001275 | 0.0008875 | 0.000875 | 0.000775 | 0.0007125 | 0.00095 | 0.0007375 | 0.0007875 | 0.0009125 | 0.000175 | 3.75E-05 | 0 | 0 | 0 | 96.71660714 |
| Dulaglutide_high_dosage | 0.52975 | 0.3748875 | 0.0148875 | 0.0058875 | 0.0037375 | 0.0028625 | 0.00515 | 0.0047125 | 0.0025875 | 0.0018875 | 0.00175 | 0.00205 | 0.0017 | 0.0021625 | 0.0015875 | 0.0021375 | 0.0020125 | 0.0043625 | 0.0039125 | 0.0034375 | 0.0046375 | 0.005225 | 0.0085125 | 0.00325 | 0.001725 | 0.00145 | 0.0012625 | 0.0019375 | 0.0005375 | 94.35553571 |
| Bexagliflozin | 0.0049875 | 0.0569625 | 0.3556 | 0.1718 | 0.0945625 | 0.0573125 | 0.03635 | 0.029975 | 0.0268 | 0.020325 | 0.0156375 | 0.0131125 | 0.0110125 | 0.0107375 | 0.010325 | 0.009675 | 0.009825 | 0.0094 | 0.009525 | 0.0092375 | 0.007925 | 0.0086 | 0.0063 | 0.005325 | 0.0043375 | 0.0020875 | 0.0012625 | 0.000625 | 0.000375 | 81.72758929 |
| Efpeglenatide_medium_dosage | 0.0057 | 0.066725 | 0.32795 | 0.1639125 | 0.07775 | 0.0610125 | 0.04595 | 0.0388625 | 0.031475 | 0.02265 | 0.01885 | 0.0145875 | 0.01245 | 0.011025 | 0.01095 | 0.011075 | 0.01355 | 0.0125125 | 0.010325 | 0.0081 | 0.00755 | 0.0087625 | 0.008075 | 0.0044125 | 0.0029625 | 0.001725 | 0.000575 | 0.0004875 | 3.75E-05 | 80.99383929 |
| Sotagliflozin | 2.00E-04 | 0.00565 | 0.06485 | 0.1918375 | 0.1862875 | 0.1467 | 0.1193125 | 0.0702875 | 0.05165 | 0.040725 | 0.029425 | 0.02165 | 0.015325 | 0.0123 | 0.00995 | 0.0085375 | 0.0080875 | 0.0056875 | 0.0047125 | 0.0029625 | 0.001925 | 0.00095 | 5.00E-04 | 0.00025 | 0.00015 | 7.50E-05 | 1.25E-05 | 0 | 0 | 79.39683036 |
| Oral_semaglutide | 0.00015 | 0.0083375 | 0.0831375 | 0.1483875 | 0.1693875 | 0.1315125 | 0.0787625 | 0.0691125 | 0.0523 | 0.0390875 | 0.032875 | 0.02805 | 0.0243375 | 0.02365 | 0.0215625 | 0.0192 | 0.0157875 | 0.0134625 | 0.0125375 | 0.008925 | 0.0067125 | 0.0055 | 0.0028125 | 0.001775 | 0.001375 | 0.0006375 | 0.0005125 | 0.0001125 | 0 | 75.70508929 |
| Inject_semaglutide_low_dosage | 0.0007125 | 0.007525 | 0.0409375 | 0.1002125 | 0.0912875 | 0.0861875 | 0.0931 | 0.06535 | 0.0612625 | 0.0435125 | 0.0338875 | 0.030975 | 0.0278 | 0.0308625 | 0.0292125 | 0.0297 | 0.0310625 | 0.0291 | 0.028025 | 0.0232875 | 0.034825 | 0.02175 | 0.0185125 | 0.015225 | 0.0119875 | 0.0077 | 0.004425 | 0.001475 | 1.00E-04 | 64.85727679 |
| Dapagliflozin_high_dosage | 1.25E-05 | 0.0003125 | 0.0018 | 0.007575 | 0.0196 | 0.043675 | 0.0775625 | 0.100225 | 0.1191 | 0.125875 | 0.1033375 | 0.1023625 | 0.0835 | 0.059875 | 0.04625 | 0.0350125 | 0.0248125 | 0.018075 | 0.0127875 | 0.0107125 | 0.004375 | 0.0019375 | 0.000925 | 0.000225 | 5.00E-05 | 2.50E-05 | 0 | 0 | 0 | 64.35004464 |
| Dulaglutide_medium_dosage | 0 | 2.00E-04 | 0.0031 | 0.0136875 | 0.04115 | 0.0629875 | 0.0873625 | 0.0957625 | 0.1046625 | 0.0997875 | 0.084175 | 0.078675 | 0.070475 | 0.0542375 | 0.04565 | 0.0438375 | 0.0445 | 0.0310125 | 0.0151875 | 0.0098125 | 0.006475 | 0.00355 | 0.002175 | 0.0010125 | 0.000375 | 0.000125 | 2.50E-05 | 0 | 0 | 64.29102679 |
| Albiglutide | 0 | 0.0003625 | 0.009225 | 0.0342625 | 0.0549375 | 0.06865 | 0.081025 | 0.0822625 | 0.0767 | 0.0719125 | 0.0693875 | 0.07305 | 0.0613875 | 0.0531 | 0.0487 | 0.0441375 | 0.036075 | 0.03315 | 0.0304125 | 0.0238 | 0.018475 | 0.0149125 | 0.0082375 | 0.0030875 | 0.0018125 | 0.0007125 | 0.000125 | 1.00E-04 | 0 | 62.79223214 |
| Inject_semaglutide_high_dosage | 0 | 0.0004875 | 0.0016125 | 0.006775 | 0.0206125 | 0.036325 | 0.051775 | 0.08275 | 0.0825125 | 0.0902875 | 0.0905625 | 0.08105 | 0.0753375 | 0.0724375 | 0.0637125 | 0.059025 | 0.0501375 | 0.044025 | 0.0354375 | 0.0225625 | 0.0158125 | 0.008125 | 0.0049875 | 0.00205 | 0.0009375 | 0.000425 | 0.00015 | 8.75E-05 | 0 | 59.38825893 |
| Dulaglutide_low_dosage | 0 | 0.0023375 | 0.0263625 | 0.0518625 | 0.0720375 | 0.065075 | 0.05645 | 0.0549625 | 0.0541625 | 0.0515125 | 0.043825 | 0.0406875 | 0.0383625 | 0.0464375 | 0.0399125 | 0.0355125 | 0.033 | 0.0358625 | 0.0376625 | 0.042225 | 0.0437375 | 0.039275 | 0.0337625 | 0.0248375 | 0.0149375 | 0.0088875 | 0.0046375 | 0.0015625 | 0.0001125 | 57.6290625 |
| Placebo_or_Control | 0 | 0 | 0 | 0 | 0 | 5.00E-05 | 0.00125 | 0.0052 | 0.0170375 | 0.049925 | 0.0998875 | 0.143425 | 0.1705375 | 0.168 | 0.1435125 | 0.1145625 | 0.0566125 | 0.02095 | 0.00655 | 0.0019625 | 0.000475 | 5.00E-05 | 1.25E-05 | 0 | 0 | 0 | 0 | 0 | 0 | 55.02732143 |
| Canagliflozin_low_dosage | 0.0001625 | 0.000625 | 0.00275 | 0.0086 | 0.0227875 | 0.0435 | 0.0506125 | 0.0486125 | 0.052025 | 0.0586 | 0.0587 | 0.0526125 | 0.0509625 | 0.0535125 | 0.0548375 | 0.0597875 | 0.0604625 | 0.056575 | 0.06215 | 0.0517625 | 0.0444125 | 0.0425 | 0.0273 | 0.0179625 | 0.0117875 | 0.0049875 | 0.0011375 | 0.000225 | 5.00E-05 | 52.37102679 |
| Ertugliflozin_high_dosage | 1.25E-05 | 0.001075 | 0.0071875 | 0.02065 | 0.029525 | 0.049025 | 0.0384625 | 0.04065 | 0.0393125 | 0.0363875 | 0.0320375 | 0.0323875 | 0.0309125 | 0.0338625 | 0.0355625 | 0.045575 | 0.0462125 | 0.0543 | 0.0492375 | 0.0702125 | 0.052 | 0.0542125 | 0.0507 | 0.045775 | 0.0405375 | 0.03555 | 0.0171125 | 0.009575 | 0.00195 | 46.01272321 |
| Empagliflozin_low_dosage | 0 | 0 | 0.0001375 | 0.000625 | 0.0018375 | 0.004775 | 0.01085 | 0.0195375 | 0.0411875 | 0.0363875 | 0.041125 | 0.0447375 | 0.0600625 | 0.0749875 | 0.0727875 | 0.0759375 | 0.0844875 | 0.0879375 | 0.087725 | 0.0800375 | 0.0634375 | 0.0468125 | 0.0314375 | 0.0204375 | 0.0090375 | 0.00275 | 5.00E-04 | 0.0002625 | 0.0001625 | 45.28732143 |
| Liraglutide | 0 | 0.0001375 | 0.0001375 | 0.00035 | 0.00125 | 0.003175 | 0.006525 | 0.0162125 | 0.0182 | 0.02445 | 0.0385125 | 0.0361 | 0.0502 | 0.0595375 | 0.078875 | 0.0872125 | 0.10415 | 0.100525 | 0.1012125 | 0.0903125 | 0.0802375 | 0.0465875 | 0.0276375 | 0.0170125 | 0.0078375 | 0.00275 | 0.000725 | 0.0001375 | 0 | 43.26223214 |
| Ertugliflozin_low_dosage | 2.50E-05 | 0.002475 | 0.0103125 | 0.0216125 | 0.0308 | 0.034325 | 0.03465 | 0.032275 | 0.0301125 | 0.029875 | 0.0304 | 0.030225 | 0.0283 | 0.0283875 | 0.032925 | 0.0342625 | 0.04095 | 0.045425 | 0.049125 | 0.0569625 | 0.0655125 | 0.060625 | 0.06835 | 0.052925 | 0.0634375 | 0.0462875 | 0.0281875 | 0.0084625 | 0.0027875 | 42.629375 |
| Efpeglenatide_high_dosage | 0 | 0.000275 | 0.006075 | 0.0210125 | 0.02535 | 0.0276125 | 0.030725 | 0.0350625 | 0.0302125 | 0.0278875 | 0.042275 | 0.0298125 | 0.0262375 | 0.0293 | 0.0349625 | 0.0355125 | 0.0349125 | 0.038675 | 0.0374625 | 0.041575 | 0.0497625 | 0.048625 | 0.0613 | 0.095525 | 0.0704625 | 0.061275 | 0.0364125 | 0.0170625 | 0.0046375 | 40.32638393 |
| Inject_semaglutide_medium_dosage | 0 | 6.25E-05 | 0.0008125 | 0.0016125 | 0.0084375 | 0.019225 | 0.0312625 | 0.0199875 | 0.0238375 | 0.027125 | 0.0288875 | 0.032875 | 0.0330375 | 0.0398375 | 0.046025 | 0.046425 | 0.0564625 | 0.065775 | 0.0706125 | 0.0732625 | 0.07925 | 0.0879125 | 0.072675 | 0.0627 | 0.045175 | 0.01905 | 0.0069125 | 0.0007625 | 0 | 40.27950893 |
| Exenatide | 0 | 1.25E-05 | 0.0001625 | 0.0004375 | 0.0016625 | 0.0032375 | 0.0075875 | 0.01045 | 0.0182375 | 0.0304875 | 0.0271875 | 0.0300125 | 0.0402625 | 0.0429125 | 0.0537375 | 0.0667625 | 0.081225 | 0.0994875 | 0.1011625 | 0.1014125 | 0.094425 | 0.07635 | 0.0613875 | 0.028525 | 0.0157875 | 0.0050125 | 0.0016625 | 0.0003875 | 2.50E-05 | 40.15736607 |
| Empagliflozin_high_dosage | 0 | 1.25E-05 | 1.00E-04 | 0.0007625 | 0.002 | 0.0049625 | 0.0082875 | 0.031225 | 0.019125 | 0.022575 | 0.0260875 | 0.0280125 | 0.0280875 | 0.030375 | 0.0371125 | 0.042575 | 0.0515125 | 0.0653875 | 0.079925 | 0.0826 | 0.084575 | 0.0912 | 0.083275 | 0.0883625 | 0.0479875 | 0.0226625 | 0.0141375 | 0.006375 | 7.00E-04 | 36.3878125 |
| Tirzepatide_medium_dosage | 0 | 0.000375 | 0.0047375 | 0.0075625 | 0.01935 | 0.0206 | 0.017425 | 0.0203125 | 0.018125 | 0.0172625 | 0.0201875 | 0.0213375 | 0.0248625 | 0.0249375 | 0.033675 | 0.033325 | 0.0301875 | 0.0368125 | 0.041425 | 0.039725 | 0.0442875 | 0.0560375 | 0.0743875 | 0.099675 | 0.14165 | 0.101175 | 0.0406 | 0.0083 | 0.0016625 | 33.40522321 |
| Canagliflozin_high_dosage | 0 | 0 | 2.50E-05 | 0.000275 | 0.0015 | 0.004625 | 0.0057375 | 0.0035875 | 0.0049625 | 0.006225 | 0.007 | 0.0078625 | 0.010175 | 0.0125875 | 0.0183875 | 0.0278375 | 0.0438 | 0.044525 | 0.056325 | 0.0717875 | 0.08945 | 0.1314125 | 0.1461 | 0.1173625 | 0.0837125 | 0.06275 | 0.0338125 | 0.0074625 | 0.0007125 | 27.57633929 |
| Dapagliflozin_low_dosage | 0 | 0.0004875 | 0.01605 | 0.0064625 | 0.0093 | 0.0101125 | 0.011225 | 0.009875 | 0.0111125 | 0.0115625 | 0.0084375 | 0.0079 | 0.00845 | 0.007675 | 0.00885 | 0.0099375 | 0.012625 | 0.0148375 | 0.01635 | 0.023775 | 0.0278625 | 0.0395375 | 0.058025 | 0.053125 | 0.0676125 | 0.091075 | 0.085225 | 0.1188375 | 0.253675 | 19.18660714 |
| Efpeglenatide_low_dosage | 0 | 0.00035 | 9.00E-04 | 0.0025375 | 0.0048375 | 0.0050375 | 0.006 | 0.0062625 | 0.007475 | 0.0069625 | 0.00875 | 0.0088875 | 0.0080125 | 0.0075875 | 0.00975 | 0.009925 | 0.0130625 | 0.01305 | 0.0147 | 0.0187875 | 0.026275 | 0.0400625 | 0.05145 | 0.0841875 | 0.101075 | 0.1239625 | 0.1576125 | 0.137725 | 0.124775 | 16.98383929 |
| Tirzepatide_low_dosage | 0 | 0 | 1.25E-05 | 3.75E-05 | 0.0003125 | 0.0008375 | 0.0009875 | 0.001775 | 0.0015125 | 0.0021 | 0.002325 | 0.0028125 | 0.0038875 | 0.005275 | 0.0057 | 0.00605 | 0.0079 | 0.0105875 | 0.01525 | 0.016975 | 0.0255 | 0.03385 | 0.0531625 | 0.089275 | 0.145375 | 0.182175 | 0.2241875 | 0.116525 | 0.0456125 | 14.15133929 |
| Lixisenatide | 0 | 6.25E-05 | 3.00E-04 | 0.0006875 | 0.0011375 | 0.00135 | 0.0015125 | 0.0021125 | 0.0020375 | 0.0024375 | 0.0025125 | 0.0028625 | 0.0032125 | 0.0031625 | 0.0042625 | 0.005 | 0.005475 | 0.0072125 | 0.0088625 | 0.0117375 | 0.01715 | 0.0211625 | 0.0298875 | 0.04935 | 0.0723875 | 0.1101875 | 0.13455 | 0.233525 | 0.2658625 | 9.787767857 |
| Tirzepatide_high_dosage | 0 | 0 | 0 | 0 | 0 | 0 | 0 | 0 | 0 | 1.25E-05 | 7.50E-05 | 6.25E-05 | 5.00E-05 | 0.000125 | 0.000175 | 0.0001875 | 0.000225 | 0.0004125 | 0.000625 | 0.0013375 | 0.0019875 | 0.0037375 | 0.007325 | 0.0154375 | 0.0353125 | 0.1044625 | 0.2042375 | 0.3279875 | 0.296225 | 4.964419643 |

**eTable 6B: SUCRA (Surface under the cumulative ranking) of primary outcome: subgroup of intra-uterus tumor**

| Treatment | Rank 1 | Rank 2 | Rank 3 | Rank 4 | Rank 5 | Rank 6 | Rank 7 | Rank 8 | Rank 9 | Rank 10 | Rank 11 | Rank 12 | Rank 13 | Rank 14 | Rank 15 | Rank 16 | Rank 17 | Rank 18 | Rank 19 | Rank 20 | Rank 21 | Rank 22 | Rank 23 | Rank 24 | Rank 25 | Rank 26 | SUCRA |
| --- | --- | --- | --- | --- | --- | --- | --- | --- | --- | --- | --- | --- | --- | --- | --- | --- | --- | --- | --- | --- | --- | --- | --- | --- | --- | --- | --- |
| Inject_semaglutide_low_dosage | 0.461925 | 0.489825 | 0.0213 | 0.008525 | 0.0051625 | 0.003425 | 0.00215 | 0.0017 | 0.00095 | 0.000775 | 0.00065 | 0.0006125 | 0.000625 | 0.0004375 | 5.00E-04 | 0.0003625 | 0.00035 | 0.0002625 | 0.0001875 | 0.000225 | 1.25E-05 | 3.75E-05 | 0 | 0 | 0 | 0 | 97.22895 |
| Efpeglenatide_low_dosage | 0.5320875 | 0.3637125 | 0.0252 | 0.01175 | 0.0072125 | 0.005325 | 0.004 | 0.00335 | 0.002475 | 0.002325 | 0.0024625 | 0.0020375 | 0.0025125 | 0.00235 | 0.0024 | 0.00245 | 0.0027375 | 0.0033125 | 0.0030625 | 0.004175 | 0.0052375 | 0.006425 | 0.001825 | 6.00E-04 | 0.0009625 | 1.25E-05 | 94.7092 |
| Orforglipron | 0.0014125 | 0.0326125 | 0.2054625 | 0.180175 | 0.1280625 | 0.095475 | 0.073975 | 0.0550625 | 0.0422875 | 0.033525 | 0.026625 | 0.0223 | 0.020725 | 0.0202625 | 0.0184875 | 0.0139625 | 0.01055 | 0.006775 | 0.0055625 | 0.0033375 | 0.0022 | 0.0010625 | 8.75E-05 | 0 | 1.25E-05 | 0 | 78.1961 |
| Liraglutide | 1.00E-04 | 0.0041625 | 0.04965 | 0.1000125 | 0.120325 | 0.11925 | 0.10685 | 0.0904125 | 0.074775 | 0.0624625 | 0.0508375 | 0.0416625 | 0.037125 | 0.0332375 | 0.0285125 | 0.0240125 | 0.0196 | 0.014825 | 0.009525 | 0.006225 | 0.004125 | 0.00195 | 0.000325 | 2.50E-05 | 1.25E-05 | 0 | 69.97665 |
| Ertugliflozin_high_dosage | 0.001925 | 0.036825 | 0.1956125 | 0.1265 | 0.0932875 | 0.063675 | 0.0501875 | 0.0425 | 0.0353 | 0.0300875 | 0.02635 | 0.026975 | 0.027425 | 0.0260625 | 0.026475 | 0.025325 | 0.025975 | 0.027575 | 0.0283375 | 0.028525 | 0.028875 | 0.0209 | 0.0039125 | 0.0009125 | 0.00045 | 2.50E-05 | 69.19655 |
| Bexagliflozin | 0.0014375 | 0.034725 | 0.197925 | 0.112975 | 0.0821875 | 0.0570375 | 0.0473375 | 0.039 | 0.0327875 | 0.0277875 | 0.0241 | 0.0232375 | 0.023575 | 0.0248875 | 0.0249375 | 0.02525 | 0.0258875 | 0.0285875 | 0.03045 | 0.03245 | 0.033475 | 0.055125 | 0.009525 | 0.002625 | 0.0024625 | 0.000225 | 65.95845 |
| Sotagliflozin | 0.0001375 | 0.0051625 | 0.0433125 | 0.0767 | 0.08945 | 0.09215 | 0.0862 | 0.080225 | 0.0683875 | 0.0583875 | 0.052425 | 0.050175 | 0.0466 | 0.0429 | 0.041425 | 0.0389125 | 0.0336375 | 0.0292875 | 0.0235125 | 0.0183 | 0.0130125 | 0.0083375 | 0.0010875 | 2.00E-04 | 6.25E-05 | 1.25E-05 | 64.3152 |
| Canagliflozin_low_dosage | 0.0001375 | 0.0029875 | 0.0250375 | 0.0475 | 0.0629875 | 0.07315 | 0.0731625 | 0.07285 | 0.068425 | 0.0609875 | 0.0560625 | 0.0551 | 0.054775 | 0.056775 | 0.0563375 | 0.0531625 | 0.0481125 | 0.0413375 | 0.03595 | 0.0259375 | 0.0173375 | 0.0099625 | 0.0016125 | 0.000225 | 8.75E-05 | 0 | 59.1284 |
| Placebo_or_Control | 0 | 0 | 1.25E-05 | 0.000175 | 0.0011375 | 0.0062625 | 0.0186375 | 0.049425 | 0.0935 | 0.145725 | 0.1813375 | 0.1840375 | 0.1446125 | 0.095425 | 0.050325 | 0.0209125 | 0.0066375 | 0.001575 | 0.0002625 | 0 | 0 | 0 | 0 | 0 | 0 | 0 | 57.90665 |
| Dulaglutide_medium_dosage | 0 | 0.0001875 | 0.0041875 | 0.01695 | 0.0342 | 0.05235 | 0.0700625 | 0.0776625 | 0.0817 | 0.079675 | 0.0785125 | 0.0746 | 0.072625 | 0.0718375 | 0.06815 | 0.06185 | 0.0494375 | 0.0393375 | 0.0283875 | 0.020225 | 0.0112875 | 0.006 | 0.000625 | 0.0001125 | 3.75E-05 | 0 | 56.76165 |
| Inject_semaglutide_high_dosage | 1.25E-05 | 0.0008125 | 0.0081875 | 0.0249125 | 0.0420625 | 0.063125 | 0.0713375 | 0.0749375 | 0.0737875 | 0.071875 | 0.0685875 | 0.0654375 | 0.0658625 | 0.06505 | 0.0608875 | 0.0566875 | 0.0511 | 0.04315 | 0.0359 | 0.027575 | 0.0171375 | 0.0098 | 0.001425 | 0.000225 | 0.0001125 | 1.25E-05 | 56.7226 |
| Efpeglenatide_high_dosage | 0.0003875 | 0.01525 | 0.104125 | 0.0816625 | 0.06495 | 0.0519 | 0.0462375 | 0.0412375 | 0.0342 | 0.032 | 0.0273625 | 0.025075 | 0.0275125 | 0.027375 | 0.03075 | 0.032275 | 0.036425 | 0.0386 | 0.04245 | 0.049725 | 0.07705 | 0.085475 | 0.0184625 | 0.00575 | 0.003525 | 0.0002375 | 55.28645 |
| Oral_semaglutide | 0.0001125 | 0.0044125 | 0.0378625 | 0.0558 | 0.058325 | 0.0612875 | 0.0574875 | 0.0541875 | 0.04745 | 0.043675 | 0.0412375 | 0.0418125 | 0.0408625 | 0.0451375 | 0.0466125 | 0.0456375 | 0.0494625 | 0.0509625 | 0.049875 | 0.0572625 | 0.0471375 | 0.0532 | 0.0083375 | 0.0012125 | 0.0005875 | 6.25E-05 | 53.9952 |
| Albiglutide | 2.50E-05 | 0.001225 | 0.0089125 | 0.022075 | 0.03545 | 0.0476625 | 0.0553625 | 0.05805 | 0.060125 | 0.0597125 | 0.0556125 | 0.0592 | 0.060675 | 0.06545 | 0.065325 | 0.0656625 | 0.061725 | 0.0599125 | 0.0519875 | 0.043425 | 0.0332 | 0.024725 | 0.0036625 | 6.00E-04 | 0.0002375 | 0 | 52.1666 |
| Exenatide | 0 | 0.0010875 | 0.0079 | 0.0176875 | 0.0275375 | 0.0359625 | 0.0422125 | 0.047475 | 0.05115 | 0.0528 | 0.0563625 | 0.0601875 | 0.06695 | 0.076075 | 0.0782375 | 0.080825 | 0.0777125 | 0.0703375 | 0.062925 | 0.041825 | 0.027225 | 0.0156375 | 0.0015875 | 0.0002375 | 6.25E-05 | 0 | 50.25235 |
| Dapagliflozin_high_dosage | 0 | 0.00015 | 0.002325 | 0.006575 | 0.013975 | 0.0234625 | 0.0354875 | 0.043775 | 0.05345 | 0.057425 | 0.0627875 | 0.0667625 | 0.0729875 | 0.078625 | 0.08325 | 0.083 | 0.0794 | 0.0714625 | 0.0627375 | 0.0480125 | 0.031975 | 0.0190375 | 0.0028375 | 0.0004375 | 6.25E-05 | 0 | 48.1108 |
| Efpeglenatide_medium_dosage | 0.00015 | 0.0025625 | 0.0331625 | 0.0528875 | 0.0526125 | 0.0466625 | 0.0418625 | 0.0385 | 0.035625 | 0.0324375 | 0.0311 | 0.03035 | 0.032025 | 0.03395 | 0.0370875 | 0.04065 | 0.0445 | 0.049025 | 0.05455 | 0.0679375 | 0.0881125 | 0.115 | 0.0265125 | 0.0069125 | 0.0054125 | 0.0004125 | 47.01495 |
| Ertugliflozin_low_dosage | 0.0001375 | 0.002875 | 0.0193125 | 0.035025 | 0.04125 | 0.043125 | 0.040425 | 0.040125 | 0.0405 | 0.035875 | 0.0350375 | 0.0337625 | 0.0367875 | 0.0393 | 0.0440875 | 0.0509375 | 0.05545 | 0.0601375 | 0.068525 | 0.0743625 | 0.0778625 | 0.099125 | 0.0187375 | 0.00455 | 0.0024625 | 0.000225 | 45.883 |
| Canagliflozin_high_dosage | 0 | 0.0004625 | 0.002975 | 0.00905 | 0.0168375 | 0.0252625 | 0.0329625 | 0.0364125 | 0.0411625 | 0.0434875 | 0.045825 | 0.049175 | 0.0573875 | 0.0659125 | 0.0732 | 0.0815125 | 0.0839125 | 0.0831 | 0.077925 | 0.07255 | 0.059825 | 0.035075 | 0.005 | 0.0007625 | 2.00E-04 | 2.50E-05 | 44.81925 |
| Inject_semaglutide_medium_dosage | 0 | 0.00025 | 0.0017 | 0.0052 | 0.0106875 | 0.015925 | 0.021275 | 0.0261375 | 0.029825 | 0.033525 | 0.036875 | 0.04115 | 0.0496625 | 0.05805 | 0.0712875 | 0.081975 | 0.0909375 | 0.097325 | 0.099275 | 0.0916375 | 0.07475 | 0.055375 | 0.00635 | 0.0007125 | 1.00E-04 | 1.25E-05 | 40.6141 |
| Empagliflozin_low_dosage | 0 | 1.25E-05 | 0.000875 | 0.002825 | 0.0054875 | 0.00825 | 0.011125 | 0.012575 | 0.01485 | 0.0165875 | 0.0189 | 0.022075 | 0.0282625 | 0.0347 | 0.04475 | 0.0541125 | 0.0674 | 0.084725 | 0.1094 | 0.1336 | 0.154875 | 0.1480875 | 0.02005 | 0.004575 | 0.0017625 | 0.0001375 | 32.07085 |
| Empagliflozin_high_dosage | 0 | 6.25E-05 | 0.0009125 | 0.002325 | 0.0043375 | 0.00665 | 0.0094625 | 0.0115125 | 0.01465 | 0.01635 | 0.0182875 | 0.0213625 | 0.026675 | 0.03205 | 0.0415 | 0.054125 | 0.0706875 | 0.0882 | 0.1045625 | 0.130325 | 0.1545625 | 0.1573 | 0.0261125 | 0.0051875 | 0.0025125 | 0.0002875 | 31.38725 |
| Tirzepatide_medium_dosage | 0 | 0 | 2.50E-05 | 0.00025 | 0.000375 | 0.00065 | 0.000625 | 0.0011375 | 0.0010125 | 0.0012625 | 0.0012875 | 0.0015 | 0.002025 | 0.0022625 | 0.003175 | 0.0037125 | 0.00515 | 0.0066125 | 0.0098125 | 0.015725 | 0.028025 | 0.0464875 | 0.6623375 | 0.1901375 | 0.0156 | 0.0008125 | 12.9352 |
| Tirzepatide_low_dosage | 0 | 0 | 0 | 0 | 0 | 0 | 0 | 2.50E-05 | 1.25E-05 | 1.25E-05 | 3.75E-05 | 7.50E-05 | 0.0001125 | 0.000175 | 0.00015 | 0.0002125 | 0.0004625 | 0.00065 | 0.00105 | 0.001575 | 0.0034125 | 0.01035 | 0.055125 | 0.4317625 | 0.4077625 | 0.0870375 | 6.1244 |
| Tirzepatide_high_dosage | 0 | 0 | 0 | 0 | 0 | 0 | 0 | 0 | 0 | 0 | 0 | 1.25E-05 | 0 | 0 | 1.25E-05 | 5.00E-05 | 2.50E-05 | 0.00015 | 0.0002125 | 0.0004625 | 0.0012 | 0.0040875 | 0.0204625 | 0.30745 | 0.5403 | 0.125575 | 4.98175 |
| Lixisenatide | 1.25E-05 | 0.0006375 | 0.004025 | 0.0024625 | 0.0021 | 0.001975 | 0.001575 | 0.001725 | 0.0016125 | 0.0012375 | 0.0013375 | 0.001325 | 0.0016125 | 0.0017125 | 0.0021375 | 0.002425 | 0.002725 | 0.002775 | 0.003575 | 0.0046 | 0.0080875 | 0.0114375 | 0.104 | 0.0347875 | 0.0152125 | 0.7848875 | 4.25745 |

**eTable 6C: SUCRA (Surface under the cumulative ranking) of primary outcome: subgroup of cervical tumor**

| Treatment | Rank 1 | Rank 2 | Rank 3 | Rank 4 | Rank 5 | Rank 6 | Rank 7 | Rank 8 | Rank 9 | Rank 10 | Rank 11 | Rank 12 | Rank 13 | Rank 14 | Rank 15 | Rank 16 | SUCRA |
| --- | --- | --- | --- | --- | --- | --- | --- | --- | --- | --- | --- | --- | --- | --- | --- | --- | --- |
| Bexagliflozin | 0.1135 | 0.1827 | 0.199275 | 0.2362 | 0.1927375 | 0.0327625 | 0.0148625 | 0.0085 | 0.0068625 | 0.006325 | 0.0049375 | 0.00115 | 0.000175 | 1.25E-05 | 0 | 0 | 83.004 |
| Canagliflozin_high_dosage | 0 | 0 | 1.25E-05 | 0.0001375 | 0.001075 | 0.00455 | 0.005775 | 0.0055625 | 0.0071375 | 0.0114125 | 0.0206875 | 0.154975 | 0.124825 | 0.1458625 | 0.2151375 | 0.30285 | 12.52483333 |
| Dapagliflozin_high_dosage | 0.0458625 | 0.140775 | 0.220325 | 0.2795625 | 0.2695625 | 0.0296125 | 0.0090875 | 0.00305 | 0.0010875 | 0.0007625 | 0.00025 | 5.00E-05 | 1.25E-05 | 0 | 0 | 0 | 81.72625 |
| Dulaglutide_medium_dosage | 0 | 0 | 0.0004125 | 0.003625 | 0.0265625 | 0.1318125 | 0.164625 | 0.15585 | 0.1678875 | 0.1805625 | 0.133975 | 0.02795 | 0.0059 | 0.00075 | 8.75E-05 | 0 | 49.64766667 |
| Empagliflozin_high_dosage | 0 | 0 | 0 | 0 | 0 | 0 | 5.00E-05 | 3.75E-05 | 0.0001375 | 0.0004125 | 0.001425 | 0.005825 | 0.1485125 | 0.080475 | 0.19975 | 0.563375 | 5.605666667 |
| Empagliflozin_low_dosage | 0 | 1.25E-05 | 3.75E-05 | 0.000575 | 0.003275 | 0.0125875 | 0.015025 | 0.014825 | 0.0175125 | 0.0264125 | 0.0498875 | 0.37065 | 0.1347625 | 0.1664375 | 0.1863625 | 0.0016375 | 22.39941667 |
| Ertugliflozin_high_dosage | 0 | 0 | 0 | 1.25E-05 | 8.75E-05 | 0.0012375 | 0.0022625 | 0.0029875 | 0.0038125 | 0.007275 | 0.016075 | 0.12185 | 0.2786375 | 0.2986625 | 0.19575 | 0.07135 | 15.499 |
| Ertugliflozin_low_dosage | 0 | 0 | 0 | 5.00E-05 | 0.000325 | 0.0013 | 0.002525 | 0.003025 | 0.004125 | 0.0074875 | 0.016125 | 0.132975 | 0.2693375 | 0.2994875 | 0.20245 | 0.0607875 | 15.73241667 |
| Exenatide | 1.25E-05 | 6.25E-05 | 0.0007625 | 0.0080625 | 0.047825 | 0.1848875 | 0.1553125 | 0.1204375 | 0.1252125 | 0.1538625 | 0.1530625 | 0.0393625 | 0.009125 | 0.00185 | 0.0001625 | 0 | 50.651 |
| Inject_semaglutide_high_dosage | 0 | 2.50E-05 | 0.0013125 | 0.0128125 | 0.081775 | 0.293525 | 0.1966875 | 0.1296 | 0.10685 | 0.0962875 | 0.0647625 | 0.0129375 | 0.003 | 0.000425 | 0 | 0 | 56.82675 |
| Liraglutide | 0 | 0 | 2.50E-05 | 0.001025 | 0.0082875 | 0.0423375 | 0.0682 | 0.08525 | 0.1237375 | 0.2168375 | 0.3433875 | 0.08995 | 0.0175125 | 0.003225 | 0.000225 | 0 | 40.84066667 |
| Placebo_or_Control | 0 | 0 | 0 | 8.75E-05 | 0.002425 | 0.0411625 | 0.1869625 | 0.3345375 | 0.2912 | 0.1208875 | 0.0211625 | 0.00155 | 2.50E-05 | 0 | 0 | 0 | 51.16083333 |
| Sotagliflozin | 0.33965 | 0.1879 | 0.1685375 | 0.1387125 | 0.1096375 | 0.0233625 | 0.010425 | 0.00645 | 0.0058625 | 0.004725 | 0.003525 | 0.001025 | 0.0001125 | 7.50E-05 | 0 | 0 | 88.38366667 |
| Tirzepatide_high_dosage | 0 | 0 | 0.0002875 | 0.00445 | 0.0364 | 0.1667375 | 0.1544125 | 0.1229625 | 0.1327625 | 0.162825 | 0.168975 | 0.039325 | 0.00805 | 0.0027375 | 7.50E-05 | 0 | 49.57658333 |
| Tirzepatide_low_dosage | 0.2607875 | 0.2353625 | 0.2103 | 0.1551 | 0.10705 | 0.01595 | 0.006175 | 0.003075 | 0.002975 | 0.0020625 | 0.0009125 | 0.0002375 | 1.25E-05 | 0 | 0 | 0 | 88.38641667 |
| Tirzepatide_medium_dosage | 0.2401875 | 0.2531625 | 0.1987125 | 0.1595875 | 0.112975 | 0.018175 | 0.0076125 | 0.00385 | 0.0028375 | 0.0018625 | 0.00085 | 0.0001875 | 0 | 0 | 0 | 0 | 88.03483333 |

**eTable 6D: SUCRA (Surface under the cumulative ranking) of primary outcome: subgroup of ovarian tumor**

| Treatment | Rank 1 | Rank 2 | Rank 3 | Rank 4 | Rank 5 | Rank 6 | Rank 7 | Rank 8 | Rank 9 | Rank 10 | Rank 11 | Rank 12 | Rank 13 | Rank 14 | Rank 15 | Rank 16 | Rank 17 | Rank 18 | Rank 19 | Rank 20 | Rank 21 | Rank 22 | Rank 23 | SUCRA |
| --- | --- | --- | --- | --- | --- | --- | --- | --- | --- | --- | --- | --- | --- | --- | --- | --- | --- | --- | --- | --- | --- | --- | --- | --- |
| Albiglutide | 0 | 0 | 3.75E-05 | 1.00E-04 | 0.001425 | 0.005675 | 0.0185375 | 0.029625 | 0.041225 | 0.0481875 | 0.04855 | 0.0487 | 0.049075 | 0.0571375 | 0.0654875 | 0.07275 | 0.0795625 | 0.0891375 | 0.0968625 | 0.1083125 | 0.12385 | 0.015325 | 0.0004375 | 32.38295455 |
| Bexagliflozin | 0.08465 | 0.1275625 | 0.1985375 | 0.204275 | 0.1618375 | 0.12075 | 0.02865 | 0.017975 | 0.012075 | 0.0085875 | 0.005625 | 0.0047625 | 0.0037375 | 0.003325 | 0.0036875 | 0.0028125 | 0.002725 | 0.0025125 | 0.00205 | 0.0019625 | 0.001725 | 0.00015 | 2.50E-05 | 84.77545455 |
| Canagliflozin_high_dosage | 0 | 0 | 4.00E-04 | 0.0035125 | 0.0213375 | 0.07875 | 0.1654875 | 0.1576875 | 0.134175 | 0.1003125 | 0.0739625 | 0.053225 | 0.0428375 | 0.0378125 | 0.033975 | 0.0303125 | 0.0238875 | 0.017575 | 0.0128875 | 0.0081125 | 0.0035875 | 0.0001625 | 0 | 59.24232955 |
| Canagliflozin_low_dosage | 0 | 3.00E-04 | 0.001125 | 0.007425 | 0.03565 | 0.103225 | 0.2185 | 0.1787 | 0.128675 | 0.09055 | 0.05915 | 0.0422375 | 0.032725 | 0.0274625 | 0.022425 | 0.018225 | 0.0135875 | 0.0094 | 0.006325 | 0.00295 | 0.0012375 | 0.000125 | 0 | 63.37534091 |
| Dapagliflozin_high_dosage | 0 | 0 | 0 | 2.50E-05 | 0.000425 | 0.0029375 | 0.0115125 | 0.028075 | 0.0526875 | 0.07095 | 0.080975 | 0.0863625 | 0.0949375 | 0.103975 | 0.10865 | 0.104225 | 0.0937875 | 0.0720625 | 0.05015 | 0.02775 | 0.010025 | 0.000475 | 1.25E-05 | 40.71085227 |
| Dulaglutide_low_dosage | 0 | 0 | 0 | 1.25E-05 | 0.0001125 | 0.0008875 | 0.0036875 | 0.0105625 | 0.019075 | 0.0289375 | 0.035625 | 0.0379875 | 0.0440375 | 0.05465 | 0.064425 | 0.0766875 | 0.0897125 | 0.1008 | 0.117025 | 0.136775 | 0.1582125 | 0.02 | 0.0007875 | 27.11340909 |
| Dulaglutide_medium_dosage | 0 | 7.50E-05 | 0.00045 | 0.00375 | 0.0211 | 0.0878875 | 0.208875 | 0.2047625 | 0.1624375 | 0.1088125 | 0.067125 | 0.042275 | 0.028575 | 0.021575 | 0.016675 | 0.011325 | 0.007375 | 0.0040875 | 0.0018625 | 0.0007375 | 0.0002125 | 2.50E-05 | 0 | 64.07164773 |
| Efpeglenatide_high_dosage | 0.06045 | 0.1447375 | 0.1574375 | 0.193075 | 0.202325 | 0.144 | 0.035675 | 0.0190125 | 0.012425 | 0.0082375 | 0.0057375 | 0.0038875 | 0.00265 | 0.00255 | 0.0021 | 0.001475 | 0.0014375 | 0.0010875 | 0.000775 | 0.0006375 | 0.0002625 | 2.50E-05 | 0 | 84.64204545 |
| Efpeglenatide_low_dosage | 0 | 0 | 0 | 7.50E-05 | 0.0008375 | 0.0062 | 0.022125 | 0.035725 | 0.045675 | 0.0541625 | 0.05425 | 0.0545875 | 0.0547375 | 0.0586125 | 0.067375 | 0.076775 | 0.0798 | 0.085325 | 0.09265 | 0.0987875 | 0.0985125 | 0.013175 | 0.0006125 | 34.27590909 |
| Efpeglenatide_medium_dosage | 0.111225 | 0.1438375 | 0.1509875 | 0.190125 | 0.18605 | 0.1359125 | 0.0291 | 0.01675 | 0.010675 | 0.0062125 | 0.0043875 | 0.003375 | 0.0024875 | 0.0023625 | 0.0017375 | 0.0014625 | 0.001425 | 0.000875 | 0.0006125 | 0.0002375 | 0.00015 | 1.25E-05 | 0 | 85.82994318 |
| Empagliflozin_low_dosage | 0 | 0 | 0 | 8.75E-05 | 0.00035 | 0.0031 | 0.0125 | 0.03135 | 0.0517625 | 0.0665125 | 0.0722125 | 0.0756125 | 0.0811125 | 0.0894875 | 0.0966625 | 0.0983 | 0.094775 | 0.0852875 | 0.0664 | 0.0487 | 0.02385 | 0.0018625 | 7.50E-05 | 38.87636364 |
| Exenatide | 0 | 0 | 5.00E-05 | 0.0001875 | 0.0015625 | 0.007975 | 0.02325 | 0.036975 | 0.04815 | 0.0581625 | 0.0607 | 0.0587125 | 0.0632125 | 0.0683125 | 0.0793 | 0.086275 | 0.088775 | 0.08915 | 0.0881125 | 0.07865 | 0.056675 | 0.0056375 | 0.000175 | 36.68357955 |
| Inject_semaglutide_high_dosage | 0 | 0 | 0 | 3.75E-05 | 0.0001625 | 0.0011125 | 0.003825 | 0.007775 | 0.014 | 0.0189625 | 0.02315 | 0.0255625 | 0.031775 | 0.0414125 | 0.0507125 | 0.06095 | 0.075825 | 0.0939875 | 0.1198125 | 0.159 | 0.2372625 | 0.0334 | 0.001275 | 23.24431818 |
| Inject_semaglutide_low_dosage | 0 | 0 | 0 | 0 | 0 | 0 | 2.50E-05 | 0.000125 | 0.0002625 | 3.00E-04 | 0.0004875 | 0.0006375 | 0.0008875 | 0.001025 | 0.00125 | 0.0019375 | 0.0027875 | 0.00455 | 0.0058625 | 0.01265 | 0.02515 | 0.4675 | 0.4745625 | 3.104772727 |
| Inject_semaglutide_medium_dosage | 0.082925 | 0.149 | 0.1799875 | 0.178275 | 0.177275 | 0.1212875 | 0.029475 | 0.018325 | 0.0148875 | 0.008575 | 0.00605 | 0.0047 | 0.0043125 | 0.0036875 | 0.0038 | 0.00365 | 0.00285 | 0.0032875 | 0.0024125 | 0.00235 | 0.0027 | 0.0001875 | 0 | 84.41784091 |
| Liraglutide | 0 | 0 | 0 | 3.75E-05 | 0.0003125 | 0.0016375 | 0.0075625 | 0.019125 | 0.0332625 | 0.0484125 | 0.05825 | 0.06305 | 0.06925 | 0.080075 | 0.0886625 | 0.0966 | 0.1013875 | 0.098575 | 0.09505 | 0.0818375 | 0.052125 | 0.004675 | 0.0001125 | 34.3825 |
| Lixisenatide | 0 | 0 | 0 | 0 | 0 | 7.50E-05 | 0.0002125 | 0.0005875 | 0.0006375 | 0.0010625 | 0.001025 | 0.000875 | 0.00145 | 0.0017125 | 0.0022375 | 0.0026875 | 0.0036 | 0.005225 | 0.0068 | 0.0112625 | 0.0219375 | 0.417425 | 0.5211875 | 3.158125 |
| Oral_semaglutide | 0 | 2.50E-05 | 0.0004125 | 0.003075 | 0.0145 | 0.0450125 | 0.0972375 | 0.0903 | 0.081775 | 0.07245 | 0.0592 | 0.0499 | 0.0452375 | 0.04575 | 0.0481125 | 0.0502 | 0.0503625 | 0.053125 | 0.05375 | 0.05875 | 0.0713125 | 0.0091375 | 0.000375 | 45.73159091 |
| Placebo_or_Control | 0 | 0 | 0 | 0 | 0 | 2.50E-05 | 0.0004625 | 0.0042 | 0.021275 | 0.0741625 | 0.1588 | 0.2235875 | 0.2239 | 0.1622 | 0.0867 | 0.0336375 | 0.0090875 | 0.0017625 | 0.0001875 | 1.25E-05 | 0 | 0 | 0 | 47.22653409 |
| Sotagliflozin | 0.301875 | 0.208075 | 0.1626375 | 0.11575 | 0.08995 | 0.0588625 | 0.01795 | 0.0096 | 0.00685 | 0.0052875 | 0.0036375 | 0.0026 | 0.0021 | 0.002125 | 0.002025 | 0.0019875 | 0.0019625 | 0.0015875 | 0.001675 | 0.00145 | 0.00175 | 0.00025 | 1.25E-05 | 90.25193182 |
| Tirzepatide_high_dosage | 0 | 0 | 1.25E-05 | 7.50E-05 | 0.000175 | 0.0013 | 0.004525 | 0.0097 | 0.02045 | 0.0312625 | 0.0414 | 0.047225 | 0.0519 | 0.0627625 | 0.0782625 | 0.0935 | 0.1046 | 0.113325 | 0.121925 | 0.11895 | 0.089775 | 0.0085625 | 0.0003125 | 29.75073864 |
| Tirzepatide_low_dosage | 0 | 0 | 2.50E-05 | 0.0005625 | 0.0032375 | 0.0167625 | 0.0482375 | 0.0670125 | 0.0839125 | 0.0878 | 0.0782 | 0.06935 | 0.0684625 | 0.0714375 | 0.075125 | 0.0738625 | 0.0705 | 0.0670125 | 0.056775 | 0.0401125 | 0.0196875 | 0.0018875 | 3.75E-05 | 44.27818182 |
| Tirzepatide_medium_dosage | 0.358875 | 0.2263875 | 0.1479 | 0.0995375 | 0.081375 | 0.056625 | 0.0125875 | 0.00605 | 0.00365 | 0.0021 | 0.0015 | 0.0007875 | 6.00E-04 | 0.00055 | 0.0006125 | 0.0003625 | 0.0001875 | 0.0002625 | 3.75E-05 | 1.25E-05 | 0 | 0 | 0 | 92.47363636 |

**eTable 6E: SUCRA (Surface under the cumulative ranking) of primary outcome: subgroup of breast tumor**

| Treatment | Rank 1 | Rank 2 | Rank 3 | Rank 4 | Rank 5 | Rank 6 | Rank 7 | Rank 8 | Rank 9 | Rank 10 | Rank 11 | Rank 12 | Rank 13 | Rank 14 | Rank 15 | Rank 16 | Rank 17 | Rank 18 | Rank 19 | Rank 20 | Rank 21 | Rank 22 | Rank 23 | Rank 24 | Rank 25 | Rank 26 | Rank 27 | Rank 28 | Rank 29 | SUCRA |
| --- | --- | --- | --- | --- | --- | --- | --- | --- | --- | --- | --- | --- | --- | --- | --- | --- | --- | --- | --- | --- | --- | --- | --- | --- | --- | --- | --- | --- | --- | --- |
| Albiglutide | 0 | 0.00805 | 0.0759625 | 0.11035 | 0.1166 | 0.1073875 | 0.0872625 | 0.077775 | 0.0656375 | 0.0559875 | 0.0499875 | 0.0432125 | 0.0357875 | 0.02905 | 0.0250375 | 0.0255 | 0.020475 | 0.0182375 | 0.013875 | 0.010975 | 0.00865 | 0.0057 | 0.0037875 | 0.002525 | 0.0012375 | 6.00E-04 | 0.0002625 | 2.50E-05 | 6.25E-05 | 72.53339286 |
| Bexagliflozin | 0 | 0.0114625 | 0.0952625 | 0.05915 | 0.04465 | 0.0353375 | 0.03255 | 0.0274625 | 0.0234875 | 0.02345 | 0.0213375 | 0.0199875 | 0.019125 | 0.0194375 | 0.020175 | 0.0224625 | 0.0229375 | 0.025125 | 0.0276875 | 0.0339 | 0.03625 | 0.0421375 | 0.0401 | 0.0457375 | 0.0491 | 0.0544625 | 0.0593375 | 0.0379375 | 0.04995 | 46.27741071 |
| Canagliflozin_high_dosage | 0 | 0 | 2.50E-05 | 0.0001125 | 0.00025 | 0.00055 | 0.0007625 | 0.0016875 | 0.00195 | 0.003175 | 0.004575 | 0.0063375 | 0.008175 | 0.0124125 | 0.0164375 | 0.0252125 | 0.0303375 | 0.0428625 | 0.0527375 | 0.0696875 | 0.0779625 | 0.102675 | 0.113375 | 0.1263 | 0.123 | 0.102675 | 0.0541375 | 0.01725 | 0.0053375 | 24.31584821 |
| Canagliflozin_low_dosage | 0 | 8.75E-05 | 0.001725 | 0.0035125 | 0.005925 | 0.009375 | 0.0121125 | 0.0163 | 0.0189875 | 0.0223125 | 0.0257 | 0.028175 | 0.032625 | 0.037925 | 0.0425625 | 0.050175 | 0.0590625 | 0.0671375 | 0.075175 | 0.079925 | 0.096175 | 0.0859125 | 0.0803125 | 0.0592625 | 0.0424 | 0.03265 | 0.01075 | 0.002975 | 0.0007625 | 37.66160714 |
| Dapagliflozin_high_dosage | 0 | 0.001225 | 0.01475 | 0.0383375 | 0.0693625 | 0.094925 | 0.11205 | 0.11845 | 0.1136125 | 0.09605 | 0.0818125 | 0.0678125 | 0.055425 | 0.04215 | 0.0285 | 0.021325 | 0.0161125 | 0.0106375 | 0.0072125 | 0.0044625 | 0.0027875 | 0.0016 | 0.0007875 | 0.0004125 | 0.0001125 | 7.50E-05 | 1.25E-05 | 0 | 0 | 69.84674107 |
| Dapagliflozin_low_dosage | 0 | 0.0023625 | 0.0236375 | 0.0089625 | 0.0101125 | 0.0097 | 0.012075 | 0.011725 | 0.0116 | 0.0122 | 0.012175 | 0.0118625 | 0.0112375 | 0.0116625 | 0.01265 | 0.01655 | 0.01845 | 0.0220125 | 0.0242375 | 0.0287 | 0.0322 | 0.03735 | 0.0445875 | 0.0563875 | 0.07435 | 0.111975 | 0.1354875 | 0.0870875 | 0.1486625 | 23.3421875 |
| Dapagliflozin_medium_dosage | 0.5746125 | 0.41775 | 0.002675 | 0.001225 | 0.0008625 | 0.00055 | 0.00035 | 3.00E-04 | 0.0002375 | 0.00025 | 0.0001875 | 8.75E-05 | 8.75E-05 | 0.00015 | 0.000125 | 1.00E-04 | 8.75E-05 | 7.50E-05 | 6.25E-05 | 8.75E-05 | 3.75E-05 | 5.00E-05 | 1.25E-05 | 0 | 0 | 1.25E-05 | 0 | 2.50E-05 | 0 | 98.3609375 |
| Dulaglutide_high_dosage | 0.4253875 | 0.4522 | 0.0142625 | 0.0060625 | 0.0040875 | 0.004075 | 0.0062 | 0.004525 | 0.0056 | 0.0046625 | 0.0027625 | 0.002575 | 0.001975 | 0.0028125 | 0.0019625 | 0.00165 | 0.002125 | 0.0016875 | 0.0021875 | 0.002075 | 0.0021875 | 0.00245 | 0.0027875 | 0.003125 | 0.0035125 | 0.00545 | 0.00685 | 0.007 | 0.0177625 | 91.73357143 |
| Dulaglutide_low_dosage | 0 | 0.0236125 | 0.055425 | 0.0545875 | 0.04415 | 0.040975 | 0.034875 | 0.035925 | 0.0375375 | 0.03535 | 0.03225 | 0.031175 | 0.029875 | 0.029825 | 0.033025 | 0.0336625 | 0.034225 | 0.0338875 | 0.0389125 | 0.0375375 | 0.04 | 0.041125 | 0.0439 | 0.0434875 | 0.0447 | 0.0387875 | 0.0288375 | 0.0141375 | 0.0082125 | 51.04035714 |
| Dulaglutide_medium_dosage | 0 | 0.0018 | 0.0136125 | 0.028975 | 0.04635 | 0.05945 | 0.0701625 | 0.0753375 | 0.0865875 | 0.0805375 | 0.08295 | 0.071775 | 0.0607875 | 0.05415 | 0.050575 | 0.04475 | 0.038625 | 0.0331125 | 0.0294625 | 0.0219875 | 0.0164125 | 0.0119 | 0.0089 | 0.00565 | 0.0035375 | 0.0016375 | 0.0006875 | 0.000225 | 6.25E-05 | 62.21200893 |
| Efpeglenatide_high_dosage | 0 | 0 | 0 | 0 | 8.75E-05 | 0.0001625 | 0.000375 | 0.0004875 | 0.0007125 | 0.001 | 0.0009375 | 0.0012 | 0.0017125 | 0.0015375 | 0.00205 | 0.0023375 | 0.0031125 | 0.003575 | 0.0056875 | 0.007725 | 0.0088375 | 0.0119 | 0.0159875 | 0.023775 | 0.03725 | 0.06665 | 0.16375 | 0.4470875 | 0.1920625 | 6.785044643 |
| Efpeglenatide_low_dosage | 0 | 0 | 0 | 7.50E-05 | 0.0002125 | 0.00025 | 0.000325 | 4.00E-04 | 5.00E-04 | 6.00E-04 | 0.0007375 | 0.000875 | 0.0009875 | 0.0011 | 0.001275 | 0.0018375 | 0.002325 | 0.0028875 | 0.0032125 | 0.0042 | 0.00665 | 0.0074875 | 0.010725 | 0.0181125 | 0.0281625 | 0.04015 | 0.097825 | 0.2519875 | 0.5171 | 4.366830357 |
| Efpeglenatide_medium_dosage | 0 | 0.025425 | 0.183025 | 0.0662 | 0.046275 | 0.0335 | 0.0293125 | 0.024725 | 0.0211 | 0.01935 | 0.0174 | 0.0167 | 0.015475 | 0.0137625 | 0.0152375 | 0.0162125 | 0.018075 | 0.019225 | 0.0219875 | 0.0214625 | 0.0251125 | 0.0292625 | 0.0321875 | 0.0384125 | 0.0464875 | 0.0731625 | 0.1192 | 0.0104625 | 0.0012625 | 52.48870536 |
| Empagliflozin_high_dosage | 0 | 0.0054625 | 0.0264875 | 0.0471375 | 0.0585125 | 0.0704125 | 0.070775 | 0.0704625 | 0.06765 | 0.0671125 | 0.0627875 | 0.056525 | 0.0523625 | 0.053825 | 0.045775 | 0.043075 | 0.0412625 | 0.036975 | 0.0325 | 0.027025 | 0.02145 | 0.015 | 0.0111625 | 0.007925 | 0.0048375 | 0.0024 | 0.000975 | 8.75E-05 | 3.75E-05 | 62.57955357 |
| Empagliflozin_low_dosage | 0 | 0.0008125 | 0.0113625 | 0.0269375 | 0.043525 | 0.0564875 | 0.0645625 | 0.070375 | 0.0774875 | 0.0846625 | 0.086325 | 0.0772375 | 0.0698625 | 0.063475 | 0.0549125 | 0.0486125 | 0.0417375 | 0.0357 | 0.026925 | 0.0209125 | 0.013375 | 0.010075 | 0.0064875 | 0.0042875 | 0.0023375 | 0.00125 | 0.000225 | 3.75E-05 | 1.25E-05 | 61.89727679 |
| Ertugliflozin_high_dosage | 0 | 0.0007125 | 0.0088375 | 0.0175 | 0.0204625 | 0.0226125 | 0.0228375 | 0.0214625 | 0.0227125 | 0.0239625 | 0.023775 | 0.024575 | 0.023225 | 0.0235875 | 0.0263375 | 0.02985 | 0.0348625 | 0.0425875 | 0.0436 | 0.0477 | 0.0571625 | 0.05965 | 0.0645375 | 0.0730375 | 0.0966625 | 0.0811375 | 0.0504625 | 0.0233875 | 0.0127625 | 35.79299107 |
| Ertugliflozin_low_dosage | 0 | 0.00555 | 0.0495625 | 0.052675 | 0.044025 | 0.04325 | 0.040525 | 0.0461875 | 0.037 | 0.0340375 | 0.03225 | 0.0338 | 0.0304875 | 0.0292625 | 0.0356875 | 0.03815 | 0.0395375 | 0.03825 | 0.0452625 | 0.044525 | 0.0449625 | 0.044875 | 0.0423625 | 0.0429875 | 0.04255 | 0.0318625 | 0.0182125 | 0.0084875 | 0.003675 | 51.19464286 |
| Exenatide | 0 | 1.00E-04 | 0.001625 | 0.00375 | 0.005975 | 0.009525 | 0.0142125 | 0.018625 | 0.0241625 | 0.0285875 | 0.033725 | 0.0413375 | 0.04655 | 0.056775 | 0.065325 | 0.0712125 | 0.079825 | 0.086125 | 0.0833125 | 0.0793125 | 0.0704625 | 0.0578125 | 0.047375 | 0.034625 | 0.0217 | 0.01195 | 0.004475 | 0.0013125 | 0.000225 | 42.81714286 |
| Inject_semaglutide_high_dosage | 0 | 2.00E-04 | 0.002475 | 0.0096125 | 0.018275 | 0.02975 | 0.04305 | 0.0586875 | 0.0705625 | 0.08305 | 0.0753375 | 0.0726375 | 0.0721125 | 0.0697 | 0.068125 | 0.065075 | 0.0598875 | 0.0540625 | 0.0430625 | 0.03985 | 0.025775 | 0.0174125 | 0.0107 | 0.006475 | 0.0025875 | 0.0011625 | 3.00E-04 | 7.50E-05 | 0 | 56.190625 |
| Inject_semaglutide_low_dosage | 0 | 0.012125 | 0.1416125 | 0.1067 | 0.086675 | 0.06655 | 0.0560375 | 0.0483 | 0.0404375 | 0.03815 | 0.0358625 | 0.031225 | 0.02985 | 0.0294875 | 0.0284 | 0.030525 | 0.029025 | 0.0272 | 0.02585 | 0.0266125 | 0.021225 | 0.0221375 | 0.0188625 | 0.01725 | 0.0145125 | 0.0084875 | 0.0052375 | 0.00145 | 0.0002125 | 66.87214286 |
| Inject_semaglutide_medium_dosage | 0 | 0.0004625 | 0.0024875 | 0.0076 | 0.0117375 | 0.0173875 | 0.01945 | 0.021475 | 0.02315 | 0.0258 | 0.0265375 | 0.0279 | 0.0305375 | 0.0354 | 0.04095 | 0.0479 | 0.0529375 | 0.06 | 0.0656875 | 0.068025 | 0.072175 | 0.0677375 | 0.066275 | 0.057875 | 0.055325 | 0.042175 | 0.0373 | 0.0135375 | 0.002175 | 38.16901786 |
| Liraglutide | 0 | 0 | 0.0001125 | 0.000375 | 0.0011375 | 0.0023125 | 0.0037875 | 0.0067875 | 0.00935 | 0.013875 | 0.0206 | 0.0269 | 0.037825 | 0.0472625 | 0.06255 | 0.075675 | 0.090875 | 0.09865 | 0.0946125 | 0.0953375 | 0.0859125 | 0.0737375 | 0.0592625 | 0.0442125 | 0.027175 | 0.014075 | 0.006175 | 0.0012125 | 0.0002125 | 38.1978125 |
| Lixisenatide | 0 | 0.0081 | 0.0366875 | 0.0331875 | 0.0292 | 0.0266 | 0.026875 | 0.0267875 | 0.02705 | 0.0246875 | 0.0251125 | 0.0253625 | 0.024825 | 0.0243625 | 0.025275 | 0.0262375 | 0.029525 | 0.0326 | 0.03615 | 0.0394375 | 0.0415 | 0.0431625 | 0.04875 | 0.0581 | 0.0661375 | 0.079825 | 0.0814875 | 0.03215 | 0.020825 | 39.87535714 |
| Oral_semaglutide | 0 | 0.009275 | 0.1225625 | 0.1589625 | 0.1247125 | 0.090225 | 0.0719625 | 0.0603625 | 0.05185 | 0.0439375 | 0.0359125 | 0.030975 | 0.02755 | 0.025075 | 0.022625 | 0.0218625 | 0.02235 | 0.021925 | 0.0191125 | 0.011075 | 0.0080875 | 0.006925 | 0.00525 | 0.0036 | 0.0022875 | 0.0011 | 0.0003375 | 8.75E-05 | 1.25E-05 | 74.17477679 |
| Placebo_or_Control | 0 | 0 | 0 | 0 | 8.75E-05 | 0.0001875 | 0.0012625 | 0.004425 | 0.01475 | 0.0361625 | 0.0758625 | 0.12855 | 0.1640125 | 0.1697 | 0.153525 | 0.11245 | 0.0730625 | 0.0392375 | 0.0177125 | 0.0063375 | 0.0019875 | 0.0005625 | 0.0001125 | 1.25E-05 | 0 | 0 | 0 | 0 | 0 | 53.58598214 |
| Sotagliflozin | 0 | 0.0091875 | 0.0655 | 0.0999 | 0.1011875 | 0.1010375 | 0.0887 | 0.0784875 | 0.0701625 | 0.0610125 | 0.053225 | 0.0437375 | 0.03745 | 0.0330375 | 0.0299375 | 0.0259 | 0.023725 | 0.0197375 | 0.0162125 | 0.0123375 | 0.01105 | 0.00755 | 0.005525 | 0.0028375 | 0.001775 | 0.0006375 | 0.00015 | 0 | 0 | 71.02892857 |
| Tirzepatide_high_dosage | 0 | 0.0001875 | 0.0057375 | 0.010775 | 0.0138125 | 0.01635 | 0.01815 | 0.021475 | 0.0243 | 0.026675 | 0.026675 | 0.0273625 | 0.028075 | 0.031475 | 0.033625 | 0.03815 | 0.04375 | 0.0487125 | 0.0580875 | 0.0621625 | 0.0685125 | 0.073575 | 0.083325 | 0.0751125 | 0.065725 | 0.051575 | 0.031425 | 0.0117875 | 0.003425 | 37.28455357 |
| Tirzepatide_low_dosage | 0 | 0.003825 | 0.0432 | 0.0448375 | 0.0474375 | 0.0452 | 0.0499375 | 0.041475 | 0.0398875 | 0.03985 | 0.0383 | 0.03415 | 0.0338375 | 0.0318125 | 0.0331125 | 0.035275 | 0.0394125 | 0.0389875 | 0.043175 | 0.041925 | 0.0434125 | 0.0467375 | 0.04675 | 0.0495375 | 0.0378 | 0.0298625 | 0.0143625 | 0.004725 | 0.001175 | 51.60169643 |
| Tirzepatide_medium_dosage | 0 | 2.50E-05 | 0.0013875 | 0.0025 | 0.0043125 | 0.005875 | 0.0094625 | 0.009525 | 0.0119375 | 0.0135125 | 0.0149 | 0.01595 | 0.0181625 | 0.0197875 | 0.024225 | 0.028275 | 0.032275 | 0.0387875 | 0.0463 | 0.0547 | 0.0596875 | 0.0735 | 0.0858125 | 0.0989375 | 0.1047375 | 0.1142125 | 0.0717375 | 0.0254625 | 0.0140125 | 27.77285714 |

**eTable 6F: SUCRA (Surface under the cumulative ranking) of primary outcome: subgroup of vaginal tumor**

| Treatment | Rank 1 | Rank 2 | Rank 3 | Rank 4 | SUCRA |
| --- | --- | --- | --- | --- | --- |
| Dapagliflozin_high_dosage | 0.21625 | 0.675275 | 0.1028125 | 0.0056625 | 70.07041667 |
| Dulaglutide_medium_dosage | 0.02475 | 0.0324 | 0.4459125 | 0.4969375 | 19.49875 |
| Liraglutide | 0.03135 | 0.0344125 | 0.4371 | 0.4971375 | 19.99916667 |
| Placebo_or_Control | 0.72765 | 0.2579125 | 0.014175 | 0.0002625 | 90.43166667 |

**eTable 6G: SUCRA (Surface under the cumulative ranking) of primary outcome: subgroup of vulvar tumor**

| Treatment | Rank 1 | Rank 2 | Rank 3 | Rank 4 | Rank 5 | Rank 6 | Rank 7 | Rank 8 | Rank 9 | Rank 10 | Rank 11 | SUCRA |
| --- | --- | --- | --- | --- | --- | --- | --- | --- | --- | --- | --- | --- |
| Albiglutide | 0.0001125 | 0.0015125 | 0.0128375 | 0.074175 | 0.236475 | 0.19405 | 0.190225 | 0.248475 | 0.038475 | 0.003625 | 3.75E-05 | 46.126625 |
| Canagliflozin_low_dosage | 0.2753875 | 0.235825 | 0.2371375 | 0.18605 | 0.029675 | 0.012825 | 0.0108125 | 0.010125 | 0.0021125 | 3.75E-05 | 1.25E-05 | 83.958125 |
| Dapagliflozin_high_dosage | 0 | 5.00E-05 | 6.00E-04 | 0.009875 | 0.0598625 | 0.120975 | 0.2635125 | 0.47665 | 0.063975 | 0.004375 | 0.000125 | 36.5475 |
| Dulaglutide_medium_dosage | 0 | 2.50E-05 | 1.25E-05 | 0.00045 | 0.0026375 | 0.004525 | 0.0081875 | 0.0211 | 0.302775 | 0.3261375 | 0.33415 | 10.696625 |
| Empagliflozin_low_dosage | 0 | 0 | 5.00E-05 | 0.0002875 | 0.001225 | 0.0026625 | 0.00505 | 0.0166875 | 0.259775 | 0.3185875 | 0.395675 | 9.31475 |
| Ertugliflozin_high_dosage | 0.236575 | 0.2367875 | 0.246875 | 0.1992375 | 0.0372875 | 0.016375 | 0.012675 | 0.0111375 | 0.0030125 | 3.75E-05 | 0 | 82.62275 |
| Ertugliflozin_low_dosage | 0.23245 | 0.2273 | 0.239175 | 0.2167375 | 0.0401125 | 0.0166 | 0.01405 | 0.0112625 | 0.002225 | 8.75E-05 | 0 | 82.189625 |
| Exenatide | 0.2554125 | 0.2965375 | 0.2407375 | 0.166475 | 0.0247375 | 0.0082875 | 0.0048875 | 0.00265 | 0.0002375 | 3.75E-05 | 0 | 85.320625 |
| Inject_semaglutide_medium_dosage | 0 | 1.00E-04 | 0.0005875 | 0.0036 | 0.0115 | 0.01215 | 0.014125 | 0.027875 | 0.313525 | 0.3465375 | 0.27 | 12.742625 |
| Liraglutide | 6.25E-05 | 0.0018625 | 0.02135 | 0.130575 | 0.4089625 | 0.1918125 | 0.1305 | 0.1031375 | 0.0112375 | 5.00E-04 | 0 | 53.694375 |
| Placebo_or_Control | 0 | 0 | 0.0006375 | 0.0125375 | 0.147525 | 0.4197375 | 0.345975 | 0.0709 | 0.00265 | 3.75E-05 | 0 | 46.786375 |

**eTable 6H: SUCRA (Surface under the cumulative ranking) of acceptability: drop-out rate (the outcome of drop-out rate here was calculated according to drop-out rate data from the original composition of subjects from included randomized controlled trials because there were no any studies provided specific information regarding drop-out rate data in female subgroup)**

| Treatment | Rank 1 | Rank 2 | Rank 3 | Rank 4 | Rank 5 | Rank 6 | Rank 7 | Rank 8 | Rank 9 | Rank 10 | Rank 11 | Rank 12 | Rank 13 | Rank 14 | Rank 15 | Rank 16 | Rank 17 | Rank 18 | Rank 19 | Rank 20 | Rank 21 | Rank 22 | Rank 23 | Rank 24 | Rank 25 | Rank 26 | Rank 27 | Rank 28 | Rank 29 | SUCRA |
| --- | --- | --- | --- | --- | --- | --- | --- | --- | --- | --- | --- | --- | --- | --- | --- | --- | --- | --- | --- | --- | --- | --- | --- | --- | --- | --- | --- | --- | --- | --- |
| Tirzepatide_low_dosage | 0.1067 | 0.1836625 | 0.1500375 | 0.1220875 | 0.0950625 | 0.077075 | 0.06015 | 0.0474 | 0.03665 | 0.028 | 0.021775 | 0.0172625 | 0.0133375 | 0.0094375 | 0.007575 | 0.0058125 | 0.0045875 | 0.0034375 | 0.002875 | 0.0021 | 0.0015375 | 0.001375 | 0.000975 | 0.0004375 | 0.00035 | 0.000125 | 0.00015 | 1.25E-05 | 1.25E-05 | 85.471875 |
| Dulaglutide_high_dosage | 0.49855 | 0.09195 | 0.0503125 | 0.03755 | 0.0303375 | 0.0253875 | 0.0215875 | 0.0193125 | 0.0186625 | 0.0152375 | 0.014625 | 0.0136 | 0.012025 | 0.0108625 | 0.010675 | 0.0106625 | 0.0099 | 0.00945 | 0.00945 | 0.0089 | 0.0091125 | 0.00755 | 0.0078625 | 0.008025 | 0.00795 | 0.0079625 | 0.0092875 | 0.0101625 | 0.01305 | 83.48879464 |
| Canagliflozin_high_dosage | 0.0490625 | 0.1142625 | 0.132425 | 0.1243 | 0.1072625 | 0.0913625 | 0.07745 | 0.0633125 | 0.0509375 | 0.0411375 | 0.033825 | 0.0267375 | 0.021075 | 0.0164875 | 0.013525 | 0.00995 | 0.007525 | 0.0059125 | 0.0040125 | 0.00315 | 0.0024 | 0.0016375 | 9.00E-04 | 0.000725 | 0.000275 | 2.00E-04 | 1.00E-04 | 5.00E-05 | 0 | 81.25321429 |
| Canagliflozin_low_dosage | 0.048475 | 0.1022375 | 0.1084375 | 0.1027625 | 0.0953 | 0.081775 | 0.07265 | 0.0625625 | 0.0554 | 0.0458375 | 0.039 | 0.0331875 | 0.0287125 | 0.02485 | 0.0197875 | 0.0163875 | 0.0136125 | 0.0112875 | 0.009575 | 0.007725 | 0.0063875 | 0.00445 | 0.003075 | 0.002675 | 0.0015125 | 0.001025 | 0.0007875 | 0.00045 | 7.50E-05 | 77.61290179 |
| Inject_semaglutide_high_dosage | 0.018175 | 0.04905 | 0.0699 | 0.0814125 | 0.0860375 | 0.086375 | 0.086 | 0.078375 | 0.073525 | 0.065675 | 0.0572375 | 0.0479125 | 0.0402375 | 0.035275 | 0.0284625 | 0.02355 | 0.0189625 | 0.01475 | 0.011575 | 0.00925 | 0.006325 | 0.0048125 | 0.0029625 | 0.0017625 | 0.0011875 | 0.000675 | 0.0003125 | 0.0001625 | 6.25E-05 | 72.94022321 |
| Tirzepatide_high_dosage | 0.0087125 | 0.0288375 | 0.0503625 | 0.0633 | 0.075075 | 0.0815125 | 0.0807875 | 0.079775 | 0.075875 | 0.0692375 | 0.059975 | 0.0528875 | 0.047725 | 0.04065 | 0.0355375 | 0.03025 | 0.025625 | 0.0216125 | 0.01815 | 0.0146125 | 0.0118125 | 0.008575 | 0.0066875 | 0.004475 | 0.0033 | 0.0020875 | 0.0015 | 0.000875 | 0.0001875 | 68.6359375 |
| Inject_semaglutide_low_dosage | 0.0474375 | 0.0779375 | 0.0716 | 0.066025 | 0.060075 | 0.0572125 | 0.0559375 | 0.0522625 | 0.04935 | 0.04525 | 0.0426125 | 0.0401625 | 0.035875 | 0.0341 | 0.0304625 | 0.02895 | 0.027825 | 0.025675 | 0.023375 | 0.0215375 | 0.019825 | 0.0171125 | 0.0150625 | 0.0127 | 0.0111 | 0.0104 | 0.0095625 | 0.00675 | 0.003825 | 67.11830357 |
| Tirzepatide_medium_dosage | 0.0048125 | 0.0194125 | 0.0361625 | 0.0520125 | 0.0658 | 0.074425 | 0.0780125 | 0.0782875 | 0.0755375 | 0.071625 | 0.0659375 | 0.06035 | 0.0522625 | 0.0456875 | 0.040025 | 0.036075 | 0.0312 | 0.025475 | 0.0215125 | 0.017325 | 0.0145375 | 0.010725 | 0.0081375 | 0.00545 | 0.0038625 | 0.0027125 | 0.0016125 | 8.00E-04 | 0.000225 | 66.09549107 |
| Dapagliflozin_low_dosage | 0.0604625 | 0.087575 | 0.070325 | 0.06075 | 0.0559 | 0.052075 | 0.0488125 | 0.0458625 | 0.0426125 | 0.0400125 | 0.0398625 | 0.0353 | 0.0337 | 0.0316375 | 0.0303125 | 0.0283125 | 0.0277875 | 0.0251875 | 0.024475 | 0.0240375 | 0.0201625 | 0.0197625 | 0.0178875 | 0.0158 | 0.0150125 | 0.014575 | 0.0135125 | 0.011725 | 0.0065625 | 65.77638393 |
| Inject_semaglutide_medium_dosage | 0.010325 | 0.030025 | 0.0441625 | 0.053775 | 0.061925 | 0.0672375 | 0.067225 | 0.0692875 | 0.06735 | 0.0632375 | 0.060875 | 0.056425 | 0.0512 | 0.0455625 | 0.0407375 | 0.0363375 | 0.0327125 | 0.0298625 | 0.0246375 | 0.022025 | 0.017425 | 0.0141 | 0.0109125 | 0.008175 | 0.0058125 | 0.003925 | 0.0027375 | 0.0016 | 0.0003875 | 65.21446429 |
| Ertugliflozin_low_dosage | 0.0221125 | 0.0408125 | 0.0432375 | 0.0437125 | 0.0451375 | 0.0429375 | 0.0463 | 0.0453 | 0.043575 | 0.043175 | 0.0428375 | 0.04255 | 0.04075 | 0.03985 | 0.0387875 | 0.0385125 | 0.036425 | 0.0365 | 0.03495 | 0.0332875 | 0.0308625 | 0.0285625 | 0.0260625 | 0.023475 | 0.022925 | 0.021525 | 0.0204125 | 0.0163375 | 0.0090875 | 56.71513393 |
| Efpeglenatide_low_dosage | 0.0508625 | 0.062425 | 0.049625 | 0.042725 | 0.040875 | 0.0391875 | 0.0367875 | 0.0360375 | 0.034575 | 0.0338375 | 0.0329125 | 0.0332375 | 0.0304375 | 0.0306375 | 0.03085 | 0.029475 | 0.0298375 | 0.029275 | 0.029 | 0.0283875 | 0.02955 | 0.028175 | 0.025825 | 0.0274625 | 0.0296125 | 0.0321875 | 0.0335375 | 0.0331 | 0.0295625 | 55.130625 |
| Empagliflozin_low_dosage | 0.0002125 | 0.0009625 | 0.0025375 | 0.0052125 | 0.0106125 | 0.01515 | 0.0236 | 0.03105 | 0.0412875 | 0.0526125 | 0.06075 | 0.0683875 | 0.07545 | 0.079475 | 0.0810125 | 0.0775125 | 0.072925 | 0.0691875 | 0.0598625 | 0.048825 | 0.040125 | 0.0300125 | 0.0212125 | 0.0139125 | 0.0081875 | 0.0055875 | 0.003 | 0.00105 | 0.0002875 | 50.29986607 |
| Empagliflozin_high_dosage | 0.000375 | 0.0019875 | 0.0045875 | 0.008675 | 0.0131375 | 0.018475 | 0.024925 | 0.032575 | 0.0378625 | 0.0463875 | 0.0522375 | 0.05835 | 0.0622625 | 0.06515 | 0.0677 | 0.0679625 | 0.0675 | 0.063875 | 0.0610875 | 0.054825 | 0.0474 | 0.0410125 | 0.0318 | 0.023725 | 0.0184 | 0.013425 | 0.0084875 | 0.0045375 | 0.001275 | 48.13066964 |
| Dapagliflozin_high_dosage | 0.0011 | 0.0037625 | 0.0081125 | 0.0131625 | 0.0178 | 0.0235625 | 0.028525 | 0.0338875 | 0.0392625 | 0.04375 | 0.048325 | 0.051425 | 0.0553375 | 0.0551375 | 0.0577125 | 0.056125 | 0.0571875 | 0.0570375 | 0.0560125 | 0.05345 | 0.0486875 | 0.04185 | 0.0367125 | 0.031875 | 0.026475 | 0.02155 | 0.0173 | 0.0108 | 0.004075 | 47.229375 |
| Albiglutide | 0.0003375 | 0.001875 | 0.0045 | 0.00785 | 0.0117125 | 0.0177625 | 0.02375 | 0.0305375 | 0.0367125 | 0.0427375 | 0.05065 | 0.0535625 | 0.06005 | 0.0632125 | 0.0634375 | 0.0662 | 0.0667 | 0.063075 | 0.060375 | 0.057475 | 0.052175 | 0.0429 | 0.03615 | 0.028725 | 0.0211125 | 0.0165625 | 0.0114625 | 0.0062625 | 0.0021375 | 46.82066964 |
| Sotagliflozin | 0.00045 | 0.0018875 | 0.003625 | 0.0066875 | 0.0103875 | 0.0153 | 0.0202 | 0.0257875 | 0.0321625 | 0.03875 | 0.0437125 | 0.0508625 | 0.0535 | 0.0577875 | 0.0610875 | 0.06545 | 0.0659 | 0.066325 | 0.0638875 | 0.060425 | 0.0554875 | 0.0491125 | 0.0419875 | 0.03365 | 0.0275125 | 0.0206375 | 0.015425 | 0.008875 | 0.0031375 | 44.62370536 |
| Bexagliflozin | 0.0493625 | 0.052075 | 0.03715 | 0.03115 | 0.0280625 | 0.0258 | 0.0252625 | 0.0243125 | 0.0234125 | 0.0241375 | 0.022675 | 0.0226 | 0.022625 | 0.0224 | 0.0221625 | 0.02255 | 0.022975 | 0.0234625 | 0.024875 | 0.0252625 | 0.0265875 | 0.0269875 | 0.0272875 | 0.028575 | 0.032825 | 0.0385375 | 0.04955 | 0.069375 | 0.1479625 | 42.92928571 |
| Ertugliflozin_high_dosage | 0.004975 | 0.0116 | 0.016225 | 0.0176875 | 0.0204125 | 0.0232875 | 0.0255125 | 0.02675 | 0.0284 | 0.03005 | 0.0319 | 0.0331125 | 0.035175 | 0.0360375 | 0.0368125 | 0.039 | 0.0407625 | 0.04195 | 0.0438375 | 0.04515 | 0.04385 | 0.045175 | 0.043825 | 0.0437375 | 0.044325 | 0.04995 | 0.05135 | 0.049125 | 0.040025 | 40.32084821 |
| Dapagliflozin_medium_dosage | 0.005225 | 0.0124 | 0.0148875 | 0.0186125 | 0.0189375 | 0.021275 | 0.0224375 | 0.02505 | 0.0271875 | 0.029075 | 0.0292625 | 0.030975 | 0.0325625 | 0.03375 | 0.0333375 | 0.0355625 | 0.036 | 0.0383625 | 0.040175 | 0.040975 | 0.0425625 | 0.0433875 | 0.04325 | 0.0432625 | 0.0466625 | 0.0529625 | 0.059175 | 0.06255 | 0.0601375 | 38.12044643 |
| Liraglutide | 1.25E-05 | 0.000125 | 3.00E-04 | 0.0009125 | 0.001825 | 0.0034125 | 0.0061375 | 0.009725 | 0.014925 | 0.020225 | 0.0247625 | 0.0328125 | 0.041575 | 0.04885 | 0.0578875 | 0.0638625 | 0.0729875 | 0.07835 | 0.083625 | 0.0825875 | 0.0765875 | 0.0712375 | 0.0604 | 0.0483875 | 0.0373375 | 0.0288625 | 0.0190875 | 0.0101375 | 0.0030625 | 37.63558036 |
| Dulaglutide_low_dosage | 2.00E-04 | 0.0013125 | 0.0036375 | 0.0051125 | 0.007825 | 0.0095875 | 0.0117625 | 0.0156375 | 0.0184875 | 0.02065 | 0.0246 | 0.0285375 | 0.0322875 | 0.035425 | 0.0391 | 0.042675 | 0.0459375 | 0.0507625 | 0.0543 | 0.0575375 | 0.0594 | 0.0592875 | 0.057925 | 0.0594625 | 0.05905 | 0.0610625 | 0.058275 | 0.0502125 | 0.02995 | 33.53852679 |
| Lixisenatide | 0.0099 | 0.0170625 | 0.0164375 | 0.0169625 | 0.0174 | 0.0189125 | 0.0185875 | 0.020075 | 0.02055 | 0.0215875 | 0.0214 | 0.023225 | 0.0243 | 0.0246125 | 0.025075 | 0.0262125 | 0.0275 | 0.02895 | 0.031375 | 0.0329375 | 0.0360875 | 0.03615 | 0.0379125 | 0.0401375 | 0.0460625 | 0.05375 | 0.0677125 | 0.096 | 0.143125 | 32.603125 |
| Dulaglutide_medium_dosage | 0.00015 | 0.00095 | 0.001925 | 0.0030125 | 0.0046 | 0.007225 | 0.009375 | 0.012 | 0.0149375 | 0.018175 | 0.0220125 | 0.024275 | 0.027875 | 0.032 | 0.0372 | 0.041025 | 0.044225 | 0.050625 | 0.053925 | 0.058425 | 0.063025 | 0.0635125 | 0.0642875 | 0.0647875 | 0.0653125 | 0.0673875 | 0.0644 | 0.0533875 | 0.0299625 | 31.3565625 |
| Efpeglenatide_medium_dosage | 0.001 | 0.0026625 | 0.0043 | 0.0062875 | 0.0081125 | 0.010275 | 0.0117375 | 0.014475 | 0.0161875 | 0.0185625 | 0.0218375 | 0.0231125 | 0.0261875 | 0.028825 | 0.0307375 | 0.034625 | 0.0369625 | 0.0396625 | 0.0428 | 0.0481 | 0.05145 | 0.0532625 | 0.0552125 | 0.057325 | 0.0649875 | 0.07315 | 0.082175 | 0.078025 | 0.0579625 | 30.07571429 |
| Efpeglenatide_high_dosage | 0.0007375 | 0.0021625 | 0.0038875 | 0.005675 | 0.0068875 | 0.0084875 | 0.0102125 | 0.0127 | 0.0146625 | 0.0184875 | 0.0188375 | 0.0215125 | 0.0222125 | 0.027025 | 0.0292875 | 0.031725 | 0.034675 | 0.0387875 | 0.0419125 | 0.0478875 | 0.0497375 | 0.053375 | 0.05495 | 0.0584375 | 0.0657875 | 0.07575 | 0.0868 | 0.0882625 | 0.0691375 | 28.37482143 |
| Oral_semaglutide | 0.00025 | 0.0008875 | 0.001 | 0.0019625 | 0.0027625 | 0.0037625 | 0.004675 | 0.0055875 | 0.007 | 0.0085 | 0.01005 | 0.0114625 | 0.0139625 | 0.0156125 | 0.01775 | 0.0204 | 0.0241125 | 0.0269375 | 0.0313875 | 0.0351125 | 0.0411125 | 0.0458875 | 0.0489 | 0.056025 | 0.068575 | 0.0853625 | 0.1084875 | 0.1384125 | 0.1640625 | 19.4734375 |
| Placebo_or_Control | 0 | 0 | 0 | 0 | 0 | 0 | 0 | 0 | 0 | 0 | 0 | 0 | 0 | 0 | 3.75E-05 | 0.000175 | 0.00095 | 0.0032 | 0.01085 | 0.0273 | 0.059425 | 0.107275 | 0.1614625 | 0.197475 | 0.191325 | 0.140975 | 0.07375 | 0.0227125 | 0.0030875 | 17.65897321 |
| Exenatide | 2.50E-05 | 1.00E-04 | 3.00E-04 | 0.000625 | 0.0007375 | 0.0011625 | 0.0016 | 0.002075 | 0.0029125 | 0.00405 | 0.0055125 | 0.006175 | 0.0073 | 0.0096625 | 0.012925 | 0.0146625 | 0.0167 | 0.021025 | 0.026125 | 0.0313875 | 0.0363625 | 0.0427375 | 0.050375 | 0.0593375 | 0.0731625 | 0.0970875 | 0.13005 | 0.16825 | 0.177575 | 15.35504464 |

**eTable 7A: Side-splitting model inconsistency of primary outcome: overall gynecologic tumor**

|  | Comparison | No.Studies | NMA | Direct | Indirect | Difference | Diff_95CI_lower | Diff_95CI_upper | *p* value |
| --- | --- | --- | --- | --- | --- | --- | --- | --- | --- |
| 1 | Albiglutide:Bexagliflozin | 0 | 0.749671276 | NA | 0.749671276 | NA | NA | NA | NA |
| 2 | Albiglutide:Canagliflozin_high_dosage | 0 | -0.606237053 | NA | -0.606237053 | NA | NA | NA | NA |
| 3 | Albiglutide:Canagliflozin_low_dosage | 0 | -0.252562453 | NA | -0.252562453 | NA | NA | NA | NA |
| 4 | Albiglutide:Dapagliflozin_high_dosage | 0 | -0.106259785 | NA | -0.106259785 | NA | NA | NA | NA |
| 5 | Albiglutide:Dapagliflozin_low_dosage | 0 | -0.680518547 | NA | -0.680518547 | NA | NA | NA | NA |
| 6 | Albiglutide:Dapagliflozin_medium_dosage | 0 | 0.770545977 | NA | 0.770545977 | NA | NA | NA | NA |
| 7 | Albiglutide:Dulaglutide_high_dosage | 0 | -0.665927094 | NA | -0.665927094 | NA | NA | NA | NA |
| 8 | Albiglutide:Dulaglutide_low_dosage | 0 | -0.064466221 | NA | -0.064466221 | NA | NA | NA | NA |
| 9 | Albiglutide:Dulaglutide_medium_dosage | 0 | -0.061814022 | NA | -0.061814022 | NA | NA | NA | NA |
| 10 | Albiglutide:Efpeglenatide_high_dosage | 0 | -0.455753354 | NA | -0.455753354 | NA | NA | NA | NA |
| 11 | Albiglutide:Efpeglenatide_low_dosage | 0 | -0.846793051 | NA | -0.846793051 | NA | NA | NA | NA |
| 12 | Albiglutide:Efpeglenatide_medium_dosage | 0 | 0.403745457 | NA | 0.403745457 | NA | NA | NA | NA |
| 13 | Albiglutide:Empagliflozin_high_dosage | 0 | -0.503526392 | NA | -0.503526392 | NA | NA | NA | NA |
| 14 | Albiglutide:Empagliflozin_low_dosage | 0 | -0.343433514 | NA | -0.343433514 | NA | NA | NA | NA |
| 15 | Albiglutide:Ertugliflozin_high_dosage | 0 | -0.395252349 | NA | -0.395252349 | NA | NA | NA | NA |
| 16 | Albiglutide:Ertugliflozin_low_dosage | 0 | -0.416896987 | NA | -0.416896987 | NA | NA | NA | NA |
| 17 | Albiglutide:Exenatide | 0 | -0.489505066 | NA | -0.489505066 | NA | NA | NA | NA |
| 18 | Albiglutide:Inject_semaglutide_high_dosage | 0 | -0.062432768 | NA | -0.062432768 | NA | NA | NA | NA |
| 19 | Albiglutide:Inject_semaglutide_low_dosage | 0 | 0.200755665 | NA | 0.200755665 | NA | NA | NA | NA |
| 20 | Albiglutide:Inject_semaglutide_medium_dosage | 0 | -0.450757971 | NA | -0.450757971 | NA | NA | NA | NA |
| 21 | Albiglutide:Liraglutide | 0 | -0.334010289 | NA | -0.334010289 | NA | NA | NA | NA |
| 22 | Albiglutide:Lixisenatide | 0 | -1.616750869 | NA | -1.616750869 | NA | NA | NA | NA |
| 23 | Albiglutide:Oral_semaglutide | 0 | 0.144388872 | NA | 0.144388872 | NA | NA | NA | NA |
| 24 | Albiglutide:Placebo_or_Control | 7 | -0.207765097 | -0.207765097 | NA | NA | NA | NA | NA |
| 25 | Albiglutide:Sotagliflozin | 0 | 0.324778788 | NA | 0.324778788 | NA | NA | NA | NA |
| 26 | Albiglutide:Tirzepatide_high_dosage | 0 | -1.068762555 | NA | -1.068762555 | NA | NA | NA | NA |
| 27 | Albiglutide:Tirzepatide_low_dosage | 0 | -0.866479499 | NA | -0.866479499 | NA | NA | NA | NA |
| 28 | Albiglutide:Tirzepatide_medium_dosage | 0 | -0.727819392 | NA | -0.727819392 | NA | NA | NA | NA |
| 29 | Bexagliflozin:Canagliflozin_high_dosage | 0 | -1.355908329 | NA | -1.355908329 | NA | NA | NA | NA |
| 30 | Bexagliflozin:Canagliflozin_low_dosage | 0 | -1.002233729 | NA | -1.002233729 | NA | NA | NA | NA |
| 31 | Bexagliflozin:Dapagliflozin_high_dosage | 0 | -0.855931061 | NA | -0.855931061 | NA | NA | NA | NA |
| 32 | Bexagliflozin:Dapagliflozin_low_dosage | 0 | -1.430189823 | NA | -1.430189823 | NA | NA | NA | NA |
| 33 | Bexagliflozin:Dapagliflozin_medium_dosage | 0 | 0.0208747 | NA | 0.0208747 | NA | NA | NA | NA |
| 34 | Bexagliflozin:Dulaglutide_high_dosage | 0 | -1.41559837 | NA | -1.41559837 | NA | NA | NA | NA |
| 35 | Bexagliflozin:Dulaglutide_low_dosage | 0 | -0.814137498 | NA | -0.814137498 | NA | NA | NA | NA |
| 36 | Bexagliflozin:Dulaglutide_medium_dosage | 0 | -0.811485298 | NA | -0.811485298 | NA | NA | NA | NA |
| 37 | Bexagliflozin:Efpeglenatide_high_dosage | 0 | -1.205424631 | NA | -1.205424631 | NA | NA | NA | NA |
| 38 | Bexagliflozin:Efpeglenatide_low_dosage | 0 | -1.596464327 | NA | -1.596464327 | NA | NA | NA | NA |
| 39 | Bexagliflozin:Efpeglenatide_medium_dosage | 0 | -0.345925819 | NA | -0.345925819 | NA | NA | NA | NA |
| 40 | Bexagliflozin:Empagliflozin_high_dosage | 0 | -1.253197668 | NA | -1.253197668 | NA | NA | NA | NA |
| 41 | Bexagliflozin:Empagliflozin_low_dosage | 0 | -1.09310479 | NA | -1.09310479 | NA | NA | NA | NA |
| 42 | Bexagliflozin:Ertugliflozin_high_dosage | 0 | -1.144923626 | NA | -1.144923626 | NA | NA | NA | NA |
| 43 | Bexagliflozin:Ertugliflozin_low_dosage | 0 | -1.166568264 | NA | -1.166568264 | NA | NA | NA | NA |
| 44 | Bexagliflozin:Exenatide | 0 | -1.239176342 | NA | -1.239176342 | NA | NA | NA | NA |
| 45 | Bexagliflozin:Inject_semaglutide_high_dosage | 0 | -0.812104045 | NA | -0.812104045 | NA | NA | NA | NA |
| 46 | Bexagliflozin:Inject_semaglutide_low_dosage | 0 | -0.548915611 | NA | -0.548915611 | NA | NA | NA | NA |
| 47 | Bexagliflozin:Inject_semaglutide_medium_dosage | 0 | -1.200429247 | NA | -1.200429247 | NA | NA | NA | NA |
| 48 | Bexagliflozin:Liraglutide | 0 | -1.083681565 | NA | -1.083681565 | NA | NA | NA | NA |
| 49 | Bexagliflozin:Lixisenatide | 0 | -2.366422146 | NA | -2.366422146 | NA | NA | NA | NA |
| 50 | Bexagliflozin:Oral_semaglutide | 0 | -0.605282404 | NA | -0.605282404 | NA | NA | NA | NA |
| 51 | Bexagliflozin:Placebo_or_Control | 1 | -0.957436373 | -0.957436373 | NA | NA | NA | NA | NA |
| 52 | Bexagliflozin:Sotagliflozin | 0 | -0.424892488 | NA | -0.424892488 | NA | NA | NA | NA |
| 53 | Bexagliflozin:Tirzepatide_high_dosage | 0 | -1.818433832 | NA | -1.818433832 | NA | NA | NA | NA |
| 54 | Bexagliflozin:Tirzepatide_low_dosage | 0 | -1.616150775 | NA | -1.616150775 | NA | NA | NA | NA |
| 55 | Bexagliflozin:Tirzepatide_medium_dosage | 0 | -1.477490668 | NA | -1.477490668 | NA | NA | NA | NA |
| 56 | Canagliflozin_high_dosage:Canagliflozin_low_dosage | 3 | 0.3536746 | 0.41740782 | 0.200574286 | 0.216833534 | -1.28140295 | 1.715070019 | 0.776672917 |
| 57 | Canagliflozin_high_dosage:Dapagliflozin_high_dosage | 0 | 0.499977268 | NA | 0.499977268 | NA | NA | NA | NA |
| 58 | Canagliflozin_high_dosage:Dapagliflozin_low_dosage | 0 | -0.074281494 | NA | -0.074281494 | NA | NA | NA | NA |
| 59 | Canagliflozin_high_dosage:Dapagliflozin_medium_dosage | 0 | 1.376783029 | NA | 1.376783029 | NA | NA | NA | NA |
| 60 | Canagliflozin_high_dosage:Dulaglutide_high_dosage | 0 | -0.059690041 | NA | -0.059690041 | NA | NA | NA | NA |
| 61 | Canagliflozin_high_dosage:Dulaglutide_low_dosage | 0 | 0.541770831 | NA | 0.541770831 | NA | NA | NA | NA |
| 62 | Canagliflozin_high_dosage:Dulaglutide_medium_dosage | 0 | 0.544423031 | NA | 0.544423031 | NA | NA | NA | NA |
| 63 | Canagliflozin_high_dosage:Efpeglenatide_high_dosage | 0 | 0.150483698 | NA | 0.150483698 | NA | NA | NA | NA |
| 64 | Canagliflozin_high_dosage:Efpeglenatide_low_dosage | 0 | -0.240555998 | NA | -0.240555998 | NA | NA | NA | NA |
| 65 | Canagliflozin_high_dosage:Efpeglenatide_medium_dosage | 0 | 1.00998251 | NA | 1.00998251 | NA | NA | NA | NA |
| 66 | Canagliflozin_high_dosage:Empagliflozin_high_dosage | 0 | 0.102710661 | NA | 0.102710661 | NA | NA | NA | NA |
| 67 | Canagliflozin_high_dosage:Empagliflozin_low_dosage | 0 | 0.262803539 | NA | 0.262803539 | NA | NA | NA | NA |
| 68 | Canagliflozin_high_dosage:Ertugliflozin_high_dosage | 0 | 0.210984703 | NA | 0.210984703 | NA | NA | NA | NA |
| 69 | Canagliflozin_high_dosage:Ertugliflozin_low_dosage | 0 | 0.189340066 | NA | 0.189340066 | NA | NA | NA | NA |
| 70 | Canagliflozin_high_dosage:Exenatide | 0 | 0.116731987 | NA | 0.116731987 | NA | NA | NA | NA |
| 71 | Canagliflozin_high_dosage:Inject_semaglutide_high_dosage | 0 | 0.543804284 | NA | 0.543804284 | NA | NA | NA | NA |
| 72 | Canagliflozin_high_dosage:Inject_semaglutide_low_dosage | 0 | 0.806992718 | NA | 0.806992718 | NA | NA | NA | NA |
| 73 | Canagliflozin_high_dosage:Inject_semaglutide_medium_dosage | 0 | 0.155479082 | NA | 0.155479082 | NA | NA | NA | NA |
| 74 | Canagliflozin_high_dosage:Liraglutide | 0 | 0.272226764 | NA | 0.272226764 | NA | NA | NA | NA |
| 75 | Canagliflozin_high_dosage:Lixisenatide | 0 | -1.010513817 | NA | -1.010513817 | NA | NA | NA | NA |
| 76 | Canagliflozin_high_dosage:Oral_semaglutide | 0 | 0.750625925 | NA | 0.750625925 | NA | NA | NA | NA |
| 77 | Canagliflozin_high_dosage:Placebo_or_Control | 5 | 0.398471956 | 0.330652581 | 1.008832996 | -0.678180415 | -2.596558565 | 1.240197736 | 0.488383771 |
| 78 | Canagliflozin_high_dosage:Sotagliflozin | 0 | 0.931015841 | NA | 0.931015841 | NA | NA | NA | NA |
| 79 | Canagliflozin_high_dosage:Tirzepatide_high_dosage | 0 | -0.462525503 | NA | -0.462525503 | NA | NA | NA | NA |
| 80 | Canagliflozin_high_dosage:Tirzepatide_low_dosage | 0 | -0.260242446 | NA | -0.260242446 | NA | NA | NA | NA |
| 81 | Canagliflozin_high_dosage:Tirzepatide_medium_dosage | 0 | -0.121582339 | NA | -0.121582339 | NA | NA | NA | NA |
| 82 | Canagliflozin_low_dosage:Dapagliflozin_high_dosage | 0 | 0.146302668 | NA | 0.146302668 | NA | NA | NA | NA |
| 83 | Canagliflozin_low_dosage:Dapagliflozin_low_dosage | 0 | -0.427956094 | NA | -0.427956094 | NA | NA | NA | NA |
| 84 | Canagliflozin_low_dosage:Dapagliflozin_medium_dosage | 0 | 1.02310843 | NA | 1.02310843 | NA | NA | NA | NA |
| 85 | Canagliflozin_low_dosage:Dulaglutide_high_dosage | 0 | -0.413364641 | NA | -0.413364641 | NA | NA | NA | NA |
| 86 | Canagliflozin_low_dosage:Dulaglutide_low_dosage | 0 | 0.188096231 | NA | 0.188096231 | NA | NA | NA | NA |
| 87 | Canagliflozin_low_dosage:Dulaglutide_medium_dosage | 0 | 0.190748431 | NA | 0.190748431 | NA | NA | NA | NA |
| 88 | Canagliflozin_low_dosage:Efpeglenatide_high_dosage | 0 | -0.203190902 | NA | -0.203190902 | NA | NA | NA | NA |
| 89 | Canagliflozin_low_dosage:Efpeglenatide_low_dosage | 0 | -0.594230598 | NA | -0.594230598 | NA | NA | NA | NA |
| 90 | Canagliflozin_low_dosage:Efpeglenatide_medium_dosage | 0 | 0.65630791 | NA | 0.65630791 | NA | NA | NA | NA |
| 91 | Canagliflozin_low_dosage:Empagliflozin_high_dosage | 0 | -0.250963939 | NA | -0.250963939 | NA | NA | NA | NA |
| 92 | Canagliflozin_low_dosage:Empagliflozin_low_dosage | 0 | -0.090871061 | NA | -0.090871061 | NA | NA | NA | NA |
| 93 | Canagliflozin_low_dosage:Ertugliflozin_high_dosage | 0 | -0.142689897 | NA | -0.142689897 | NA | NA | NA | NA |
| 94 | Canagliflozin_low_dosage:Ertugliflozin_low_dosage | 0 | -0.164334534 | NA | -0.164334534 | NA | NA | NA | NA |
| 95 | Canagliflozin_low_dosage:Exenatide | 0 | -0.236942613 | NA | -0.236942613 | NA | NA | NA | NA |
| 96 | Canagliflozin_low_dosage:Inject_semaglutide_high_dosage | 0 | 0.190129684 | NA | 0.190129684 | NA | NA | NA | NA |
| 97 | Canagliflozin_low_dosage:Inject_semaglutide_low_dosage | 0 | 0.453318118 | NA | 0.453318118 | NA | NA | NA | NA |
| 98 | Canagliflozin_low_dosage:Inject_semaglutide_medium_dosage | 0 | -0.198195518 | NA | -0.198195518 | NA | NA | NA | NA |
| 99 | Canagliflozin_low_dosage:Liraglutide | 0 | -0.081447836 | NA | -0.081447836 | NA | NA | NA | NA |
| 100 | Canagliflozin_low_dosage:Lixisenatide | 0 | -1.364188417 | NA | -1.364188417 | NA | NA | NA | NA |
| 101 | Canagliflozin_low_dosage:Oral_semaglutide | 0 | 0.396951325 | NA | 0.396951325 | NA | NA | NA | NA |
| 102 | Canagliflozin_low_dosage:Placebo_or_Control | 2 | 0.044797356 | 0.094443668 | -0.337135638 | 0.431579306 | -1.515553663 | 2.378712275 | 0.663981094 |
| 103 | Canagliflozin_low_dosage:Sotagliflozin | 0 | 0.577341241 | NA | 0.577341241 | NA | NA | NA | NA |
| 104 | Canagliflozin_low_dosage:Tirzepatide_high_dosage | 0 | -0.816200103 | NA | -0.816200103 | NA | NA | NA | NA |
| 105 | Canagliflozin_low_dosage:Tirzepatide_low_dosage | 0 | -0.613917046 | NA | -0.613917046 | NA | NA | NA | NA |
| 106 | Canagliflozin_low_dosage:Tirzepatide_medium_dosage | 0 | -0.475256939 | NA | -0.475256939 | NA | NA | NA | NA |
| 107 | Dapagliflozin_high_dosage:Dapagliflozin_low_dosage | 3 | -0.574258762 | -0.432798864 | -0.735548697 | 0.302749833 | -2.41363735 | 3.019137016 | 0.827083104 |
| 108 | Dapagliflozin_high_dosage:Dapagliflozin_medium_dosage | 1 | 0.876805762 | 1.273661086 | 0.599296054 | 0.674365032 | -3.522773576 | 4.87150364 | 0.752828133 |
| 109 | Dapagliflozin_high_dosage:Dulaglutide_high_dosage | 0 | -0.559667309 | NA | -0.559667309 | NA | NA | NA | NA |
| 110 | Dapagliflozin_high_dosage:Dulaglutide_low_dosage | 0 | 0.041793563 | NA | 0.041793563 | NA | NA | NA | NA |
| 111 | Dapagliflozin_high_dosage:Dulaglutide_medium_dosage | 0 | 0.044445763 | NA | 0.044445763 | NA | NA | NA | NA |
| 112 | Dapagliflozin_high_dosage:Efpeglenatide_high_dosage | 0 | -0.34949357 | NA | -0.34949357 | NA | NA | NA | NA |
| 113 | Dapagliflozin_high_dosage:Efpeglenatide_low_dosage | 0 | -0.740533266 | NA | -0.740533266 | NA | NA | NA | NA |
| 114 | Dapagliflozin_high_dosage:Efpeglenatide_medium_dosage | 0 | 0.510005242 | NA | 0.510005242 | NA | NA | NA | NA |
| 115 | Dapagliflozin_high_dosage:Empagliflozin_high_dosage | 0 | -0.397266607 | NA | -0.397266607 | NA | NA | NA | NA |
| 116 | Dapagliflozin_high_dosage:Empagliflozin_low_dosage | 0 | -0.237173729 | NA | -0.237173729 | NA | NA | NA | NA |
| 117 | Dapagliflozin_high_dosage:Ertugliflozin_high_dosage | 0 | -0.288992565 | NA | -0.288992565 | NA | NA | NA | NA |
| 118 | Dapagliflozin_high_dosage:Ertugliflozin_low_dosage | 0 | -0.310637202 | NA | -0.310637202 | NA | NA | NA | NA |
| 119 | Dapagliflozin_high_dosage:Exenatide | 0 | -0.383245281 | NA | -0.383245281 | NA | NA | NA | NA |
| 120 | Dapagliflozin_high_dosage:Inject_semaglutide_high_dosage | 0 | 0.043827016 | NA | 0.043827016 | NA | NA | NA | NA |
| 121 | Dapagliflozin_high_dosage:Inject_semaglutide_low_dosage | 0 | 0.30701545 | NA | 0.30701545 | NA | NA | NA | NA |
| 122 | Dapagliflozin_high_dosage:Inject_semaglutide_medium_dosage | 0 | -0.344498186 | NA | -0.344498186 | NA | NA | NA | NA |
| 123 | Dapagliflozin_high_dosage:Liraglutide | 0 | -0.227750504 | NA | -0.227750504 | NA | NA | NA | NA |
| 124 | Dapagliflozin_high_dosage:Lixisenatide | 0 | -1.510491085 | NA | -1.510491085 | NA | NA | NA | NA |
| 125 | Dapagliflozin_high_dosage:Oral_semaglutide | 0 | 0.250648657 | NA | 0.250648657 | NA | NA | NA | NA |
| 126 | Dapagliflozin_high_dosage:Placebo_or_Control | 7 | -0.101505312 | -0.108314548 | 0.532873739 | -0.641188286 | -3.484503974 | 2.202127401 | 0.65849928 |
| 127 | Dapagliflozin_high_dosage:Sotagliflozin | 0 | 0.431038573 | NA | 0.431038573 | NA | NA | NA | NA |
| 128 | Dapagliflozin_high_dosage:Tirzepatide_high_dosage | 0 | -0.962502771 | NA | -0.962502771 | NA | NA | NA | NA |
| 129 | Dapagliflozin_high_dosage:Tirzepatide_low_dosage | 0 | -0.760219714 | NA | -0.760219714 | NA | NA | NA | NA |
| 130 | Dapagliflozin_high_dosage:Tirzepatide_medium_dosage | 0 | -0.621559607 | NA | -0.621559607 | NA | NA | NA | NA |
| 131 | Dapagliflozin_low_dosage:Dapagliflozin_medium_dosage | 2 | 1.451064524 | 1.253299094 | 1.927664126 | -0.674365032 | -4.87150364 | 3.522773576 | 0.752828133 |
| 132 | Dapagliflozin_low_dosage:Dulaglutide_high_dosage | 0 | 0.014591453 | NA | 0.014591453 | NA | NA | NA | NA |
| 133 | Dapagliflozin_low_dosage:Dulaglutide_low_dosage | 0 | 0.616052326 | NA | 0.616052326 | NA | NA | NA | NA |
| 134 | Dapagliflozin_low_dosage:Dulaglutide_medium_dosage | 0 | 0.618704525 | NA | 0.618704525 | NA | NA | NA | NA |
| 135 | Dapagliflozin_low_dosage:Efpeglenatide_high_dosage | 0 | 0.224765193 | NA | 0.224765193 | NA | NA | NA | NA |
| 136 | Dapagliflozin_low_dosage:Efpeglenatide_low_dosage | 0 | -0.166274504 | NA | -0.166274504 | NA | NA | NA | NA |
| 137 | Dapagliflozin_low_dosage:Efpeglenatide_medium_dosage | 0 | 1.084264004 | NA | 1.084264004 | NA | NA | NA | NA |
| 138 | Dapagliflozin_low_dosage:Empagliflozin_high_dosage | 0 | 0.176992155 | NA | 0.176992155 | NA | NA | NA | NA |
| 139 | Dapagliflozin_low_dosage:Empagliflozin_low_dosage | 0 | 0.337085033 | NA | 0.337085033 | NA | NA | NA | NA |
| 140 | Dapagliflozin_low_dosage:Ertugliflozin_high_dosage | 0 | 0.285266198 | NA | 0.285266198 | NA | NA | NA | NA |
| 141 | Dapagliflozin_low_dosage:Ertugliflozin_low_dosage | 0 | 0.26362156 | NA | 0.26362156 | NA | NA | NA | NA |
| 142 | Dapagliflozin_low_dosage:Exenatide | 0 | 0.191013481 | NA | 0.191013481 | NA | NA | NA | NA |
| 143 | Dapagliflozin_low_dosage:Inject_semaglutide_high_dosage | 0 | 0.618085779 | NA | 0.618085779 | NA | NA | NA | NA |
| 144 | Dapagliflozin_low_dosage:Inject_semaglutide_low_dosage | 0 | 0.881274212 | NA | 0.881274212 | NA | NA | NA | NA |
| 145 | Dapagliflozin_low_dosage:Inject_semaglutide_medium_dosage | 0 | 0.229760576 | NA | 0.229760576 | NA | NA | NA | NA |
| 146 | Dapagliflozin_low_dosage:Liraglutide | 0 | 0.346508258 | NA | 0.346508258 | NA | NA | NA | NA |
| 147 | Dapagliflozin_low_dosage:Lixisenatide | 0 | -0.936232322 | NA | -0.936232322 | NA | NA | NA | NA |
| 148 | Dapagliflozin_low_dosage:Oral_semaglutide | 0 | 0.824907419 | NA | 0.824907419 | NA | NA | NA | NA |
| 149 | Dapagliflozin_low_dosage:Placebo_or_Control | 2 | 0.47275345 | 0.883358288 | 0.242170002 | 0.641188286 | -2.202127401 | 3.484503974 | 0.65849928 |
| 150 | Dapagliflozin_low_dosage:Sotagliflozin | 0 | 1.005297335 | NA | 1.005297335 | NA | NA | NA | NA |
| 151 | Dapagliflozin_low_dosage:Tirzepatide_high_dosage | 0 | -0.388244008 | NA | -0.388244008 | NA | NA | NA | NA |
| 152 | Dapagliflozin_low_dosage:Tirzepatide_low_dosage | 0 | -0.185960952 | NA | -0.185960952 | NA | NA | NA | NA |
| 153 | Dapagliflozin_low_dosage:Tirzepatide_medium_dosage | 0 | -0.047300845 | NA | -0.047300845 | NA | NA | NA | NA |
| 154 | Dapagliflozin_medium_dosage:Dulaglutide_high_dosage | 0 | -1.436473071 | NA | -1.436473071 | NA | NA | NA | NA |
| 155 | Dapagliflozin_medium_dosage:Dulaglutide_low_dosage | 0 | -0.835012198 | NA | -0.835012198 | NA | NA | NA | NA |
| 156 | Dapagliflozin_medium_dosage:Dulaglutide_medium_dosage | 0 | -0.832359999 | NA | -0.832359999 | NA | NA | NA | NA |
| 157 | Dapagliflozin_medium_dosage:Efpeglenatide_high_dosage | 0 | -1.226299331 | NA | -1.226299331 | NA | NA | NA | NA |
| 158 | Dapagliflozin_medium_dosage:Efpeglenatide_low_dosage | 0 | -1.617339028 | NA | -1.617339028 | NA | NA | NA | NA |
| 159 | Dapagliflozin_medium_dosage:Efpeglenatide_medium_dosage | 0 | -0.366800519 | NA | -0.366800519 | NA | NA | NA | NA |
| 160 | Dapagliflozin_medium_dosage:Empagliflozin_high_dosage | 0 | -1.274072369 | NA | -1.274072369 | NA | NA | NA | NA |
| 161 | Dapagliflozin_medium_dosage:Empagliflozin_low_dosage | 0 | -1.113979491 | NA | -1.113979491 | NA | NA | NA | NA |
| 162 | Dapagliflozin_medium_dosage:Ertugliflozin_high_dosage | 0 | -1.165798326 | NA | -1.165798326 | NA | NA | NA | NA |
| 163 | Dapagliflozin_medium_dosage:Ertugliflozin_low_dosage | 0 | -1.187442964 | NA | -1.187442964 | NA | NA | NA | NA |
| 164 | Dapagliflozin_medium_dosage:Exenatide | 0 | -1.260051043 | NA | -1.260051043 | NA | NA | NA | NA |
| 165 | Dapagliflozin_medium_dosage:Inject_semaglutide_high_dosage | 0 | -0.832978745 | NA | -0.832978745 | NA | NA | NA | NA |
| 166 | Dapagliflozin_medium_dosage:Inject_semaglutide_low_dosage | 0 | -0.569790312 | NA | -0.569790312 | NA | NA | NA | NA |
| 167 | Dapagliflozin_medium_dosage:Inject_semaglutide_medium_dosage | 0 | -1.221303948 | NA | -1.221303948 | NA | NA | NA | NA |
| 168 | Dapagliflozin_medium_dosage:Liraglutide | 0 | -1.104556266 | NA | -1.104556266 | NA | NA | NA | NA |
| 169 | Dapagliflozin_medium_dosage:Lixisenatide | 0 | -2.387296846 | NA | -2.387296846 | NA | NA | NA | NA |
| 170 | Dapagliflozin_medium_dosage:Oral_semaglutide | 0 | -0.626157105 | NA | -0.626157105 | NA | NA | NA | NA |
| 171 | Dapagliflozin_medium_dosage:Placebo_or_Control | 0 | -0.978311074 | NA | -0.978311074 | NA | NA | NA | NA |
| 172 | Dapagliflozin_medium_dosage:Sotagliflozin | 0 | -0.445767189 | NA | -0.445767189 | NA | NA | NA | NA |
| 173 | Dapagliflozin_medium_dosage:Tirzepatide_high_dosage | 0 | -1.839308532 | NA | -1.839308532 | NA | NA | NA | NA |
| 174 | Dapagliflozin_medium_dosage:Tirzepatide_low_dosage | 0 | -1.637025476 | NA | -1.637025476 | NA | NA | NA | NA |
| 175 | Dapagliflozin_medium_dosage:Tirzepatide_medium_dosage | 0 | -1.498365369 | NA | -1.498365369 | NA | NA | NA | NA |
| 176 | Dulaglutide_high_dosage:Dulaglutide_low_dosage | 1 | 0.601460872 | 0.725937003 | 0.46628425 | 0.259652754 | -4.393809835 | 4.913115342 | 0.912915701 |
| 177 | Dulaglutide_high_dosage:Dulaglutide_medium_dosage | 1 | 0.604113072 | 0.47957308 | 0.739225834 | -0.259652754 | -4.913115342 | 4.393809835 | 0.912915701 |
| 178 | Dulaglutide_high_dosage:Efpeglenatide_high_dosage | 0 | 0.210173739 | NA | 0.210173739 | NA | NA | NA | NA |
| 179 | Dulaglutide_high_dosage:Efpeglenatide_low_dosage | 0 | -0.180865957 | NA | -0.180865957 | NA | NA | NA | NA |
| 180 | Dulaglutide_high_dosage:Efpeglenatide_medium_dosage | 0 | 1.069672551 | NA | 1.069672551 | NA | NA | NA | NA |
| 181 | Dulaglutide_high_dosage:Empagliflozin_high_dosage | 0 | 0.162400702 | NA | 0.162400702 | NA | NA | NA | NA |
| 182 | Dulaglutide_high_dosage:Empagliflozin_low_dosage | 0 | 0.32249358 | NA | 0.32249358 | NA | NA | NA | NA |
| 183 | Dulaglutide_high_dosage:Ertugliflozin_high_dosage | 0 | 0.270674744 | NA | 0.270674744 | NA | NA | NA | NA |
| 184 | Dulaglutide_high_dosage:Ertugliflozin_low_dosage | 0 | 0.249030107 | NA | 0.249030107 | NA | NA | NA | NA |
| 185 | Dulaglutide_high_dosage:Exenatide | 0 | 0.176422028 | NA | 0.176422028 | NA | NA | NA | NA |
| 186 | Dulaglutide_high_dosage:Inject_semaglutide_high_dosage | 0 | 0.603494325 | NA | 0.603494325 | NA | NA | NA | NA |
| 187 | Dulaglutide_high_dosage:Inject_semaglutide_low_dosage | 0 | 0.866682759 | NA | 0.866682759 | NA | NA | NA | NA |
| 188 | Dulaglutide_high_dosage:Inject_semaglutide_medium_dosage | 0 | 0.215169123 | NA | 0.215169123 | NA | NA | NA | NA |
| 189 | Dulaglutide_high_dosage:Liraglutide | 0 | 0.331916805 | NA | 0.331916805 | NA | NA | NA | NA |
| 190 | Dulaglutide_high_dosage:Lixisenatide | 0 | -0.950823776 | NA | -0.950823776 | NA | NA | NA | NA |
| 191 | Dulaglutide_high_dosage:Oral_semaglutide | 0 | 0.810315966 | NA | 0.810315966 | NA | NA | NA | NA |
| 192 | Dulaglutide_high_dosage:Placebo_or_Control | 0 | 0.458161997 | NA | 0.458161997 | NA | NA | NA | NA |
| 193 | Dulaglutide_high_dosage:Sotagliflozin | 0 | 0.990705882 | NA | 0.990705882 | NA | NA | NA | NA |
| 194 | Dulaglutide_high_dosage:Tirzepatide_high_dosage | 0 | -0.402835462 | NA | -0.402835462 | NA | NA | NA | NA |
| 195 | Dulaglutide_high_dosage:Tirzepatide_low_dosage | 0 | -0.200552405 | NA | -0.200552405 | NA | NA | NA | NA |
| 196 | Dulaglutide_high_dosage:Tirzepatide_medium_dosage | 0 | -0.061892298 | NA | -0.061892298 | NA | NA | NA | NA |
| 197 | Dulaglutide_low_dosage:Dulaglutide_medium_dosage | 4 | 0.0026522 | 0.337848643 | -0.279281338 | 0.617129981 | -1.23449337 | 2.468753332 | 0.513602229 |
| 198 | Dulaglutide_low_dosage:Efpeglenatide_high_dosage | 0 | -0.391287133 | NA | -0.391287133 | NA | NA | NA | NA |
| 199 | Dulaglutide_low_dosage:Efpeglenatide_low_dosage | 0 | -0.782326829 | NA | -0.782326829 | NA | NA | NA | NA |
| 200 | Dulaglutide_low_dosage:Efpeglenatide_medium_dosage | 0 | 0.468211679 | NA | 0.468211679 | NA | NA | NA | NA |
| 201 | Dulaglutide_low_dosage:Empagliflozin_high_dosage | 0 | -0.43906017 | NA | -0.43906017 | NA | NA | NA | NA |
| 202 | Dulaglutide_low_dosage:Empagliflozin_low_dosage | 0 | -0.278967292 | NA | -0.278967292 | NA | NA | NA | NA |
| 203 | Dulaglutide_low_dosage:Ertugliflozin_high_dosage | 0 | -0.330786128 | NA | -0.330786128 | NA | NA | NA | NA |
| 204 | Dulaglutide_low_dosage:Ertugliflozin_low_dosage | 0 | -0.352430766 | NA | -0.352430766 | NA | NA | NA | NA |
| 205 | Dulaglutide_low_dosage:Exenatide | 0 | -0.425038845 | NA | -0.425038845 | NA | NA | NA | NA |
| 206 | Dulaglutide_low_dosage:Inject_semaglutide_high_dosage | 0 | 0.002033453 | NA | 0.002033453 | NA | NA | NA | NA |
| 207 | Dulaglutide_low_dosage:Inject_semaglutide_low_dosage | 0 | 0.265221887 | NA | 0.265221887 | NA | NA | NA | NA |
| 208 | Dulaglutide_low_dosage:Inject_semaglutide_medium_dosage | 0 | -0.38629175 | NA | -0.38629175 | NA | NA | NA | NA |
| 209 | Dulaglutide_low_dosage:Liraglutide | 0 | -0.269544067 | NA | -0.269544067 | NA | NA | NA | NA |
| 210 | Dulaglutide_low_dosage:Lixisenatide | 0 | -1.552284648 | NA | -1.552284648 | NA | NA | NA | NA |
| 211 | Dulaglutide_low_dosage:Oral_semaglutide | 0 | 0.208855094 | NA | 0.208855094 | NA | NA | NA | NA |
| 212 | Dulaglutide_low_dosage:Placebo_or_Control | 4 | -0.143298876 | -0.118452881 | -0.204352681 | 0.0858998 | -1.90641594 | 2.07821554 | 0.932654978 |
| 213 | Dulaglutide_low_dosage:Sotagliflozin | 0 | 0.389245009 | NA | 0.389245009 | NA | NA | NA | NA |
| 214 | Dulaglutide_low_dosage:Tirzepatide_high_dosage | 0 | -1.004296334 | NA | -1.004296334 | NA | NA | NA | NA |
| 215 | Dulaglutide_low_dosage:Tirzepatide_low_dosage | 0 | -0.802013277 | NA | -0.802013277 | NA | NA | NA | NA |
| 216 | Dulaglutide_low_dosage:Tirzepatide_medium_dosage | 0 | -0.663353171 | NA | -0.663353171 | NA | NA | NA | NA |
| 217 | Dulaglutide_medium_dosage:Efpeglenatide_high_dosage | 0 | -0.393939333 | NA | -0.393939333 | NA | NA | NA | NA |
| 218 | Dulaglutide_medium_dosage:Efpeglenatide_low_dosage | 0 | -0.784979029 | NA | -0.784979029 | NA | NA | NA | NA |
| 219 | Dulaglutide_medium_dosage:Efpeglenatide_medium_dosage | 0 | 0.465559479 | NA | 0.465559479 | NA | NA | NA | NA |
| 220 | Dulaglutide_medium_dosage:Empagliflozin_high_dosage | 0 | -0.44171237 | NA | -0.44171237 | NA | NA | NA | NA |
| 221 | Dulaglutide_medium_dosage:Empagliflozin_low_dosage | 0 | -0.281619492 | NA | -0.281619492 | NA | NA | NA | NA |
| 222 | Dulaglutide_medium_dosage:Ertugliflozin_high_dosage | 0 | -0.333438328 | NA | -0.333438328 | NA | NA | NA | NA |
| 223 | Dulaglutide_medium_dosage:Ertugliflozin_low_dosage | 0 | -0.355082965 | NA | -0.355082965 | NA | NA | NA | NA |
| 224 | Dulaglutide_medium_dosage:Exenatide | 0 | -0.427691044 | NA | -0.427691044 | NA | NA | NA | NA |
| 225 | Dulaglutide_medium_dosage:Inject_semaglutide_high_dosage | 0 | -0.000618747 | NA | -0.000618747 | NA | NA | NA | NA |
| 226 | Dulaglutide_medium_dosage:Inject_semaglutide_low_dosage | 0 | 0.262569687 | NA | 0.262569687 | NA | NA | NA | NA |
| 227 | Dulaglutide_medium_dosage:Inject_semaglutide_medium_dosage | 0 | -0.388943949 | NA | -0.388943949 | NA | NA | NA | NA |
| 228 | Dulaglutide_medium_dosage:Liraglutide | 0 | -0.272196267 | NA | -0.272196267 | NA | NA | NA | NA |
| 229 | Dulaglutide_medium_dosage:Lixisenatide | 0 | -1.554936848 | NA | -1.554936848 | NA | NA | NA | NA |
| 230 | Dulaglutide_medium_dosage:Oral_semaglutide | 0 | 0.206202894 | NA | 0.206202894 | NA | NA | NA | NA |
| 231 | Dulaglutide_medium_dosage:Placebo_or_Control | 6 | -0.145951075 | -0.163910295 | 0.664378615 | -0.82828891 | -3.331800463 | 1.675222642 | 0.51669023 |
| 232 | Dulaglutide_medium_dosage:Sotagliflozin | 0 | 0.38659281 | NA | 0.38659281 | NA | NA | NA | NA |
| 233 | Dulaglutide_medium_dosage:Tirzepatide_high_dosage | 0 | -1.006948534 | NA | -1.006948534 | NA | NA | NA | NA |
| 234 | Dulaglutide_medium_dosage:Tirzepatide_low_dosage | 0 | -0.804665477 | NA | -0.804665477 | NA | NA | NA | NA |
| 235 | Dulaglutide_medium_dosage:Tirzepatide_medium_dosage | 0 | -0.66600537 | NA | -0.66600537 | NA | NA | NA | NA |
| 236 | Efpeglenatide_high_dosage:Efpeglenatide_low_dosage | 2 | -0.391039696 | -0.495360994 | -0.117172401 | -0.378188593 | -3.215474425 | 2.459097238 | 0.793901054 |
| 237 | Efpeglenatide_high_dosage:Efpeglenatide_medium_dosage | 2 | 0.859498812 | 0.978934895 | 0.381337651 | 0.597597244 | -2.768331217 | 3.963525705 | 0.727856701 |
| 238 | Efpeglenatide_high_dosage:Empagliflozin_high_dosage | 0 | -0.047773037 | NA | -0.047773037 | NA | NA | NA | NA |
| 239 | Efpeglenatide_high_dosage:Empagliflozin_low_dosage | 0 | 0.112319841 | NA | 0.112319841 | NA | NA | NA | NA |
| 240 | Efpeglenatide_high_dosage:Ertugliflozin_high_dosage | 0 | 0.060501005 | NA | 0.060501005 | NA | NA | NA | NA |
| 241 | Efpeglenatide_high_dosage:Ertugliflozin_low_dosage | 0 | 0.038856367 | NA | 0.038856367 | NA | NA | NA | NA |
| 242 | Efpeglenatide_high_dosage:Exenatide | 0 | -0.033751712 | NA | -0.033751712 | NA | NA | NA | NA |
| 243 | Efpeglenatide_high_dosage:Inject_semaglutide_high_dosage | 0 | 0.393320586 | NA | 0.393320586 | NA | NA | NA | NA |
| 244 | Efpeglenatide_high_dosage:Inject_semaglutide_low_dosage | 0 | 0.65650902 | NA | 0.65650902 | NA | NA | NA | NA |
| 245 | Efpeglenatide_high_dosage:Inject_semaglutide_medium_dosage | 0 | 0.004995383 | NA | 0.004995383 | NA | NA | NA | NA |
| 246 | Efpeglenatide_high_dosage:Liraglutide | 0 | 0.121743065 | NA | 0.121743065 | NA | NA | NA | NA |
| 247 | Efpeglenatide_high_dosage:Lixisenatide | 0 | -1.160997515 | NA | -1.160997515 | NA | NA | NA | NA |
| 248 | Efpeglenatide_high_dosage:Oral_semaglutide | 0 | 0.600142227 | NA | 0.600142227 | NA | NA | NA | NA |
| 249 | Efpeglenatide_high_dosage:Placebo_or_Control | 3 | 0.247988257 | 0.488891806 | -0.866749143 | 1.355640948 | -1.851273572 | 4.562555469 | 0.407373493 |
| 250 | Efpeglenatide_high_dosage:Sotagliflozin | 0 | 0.780532142 | NA | 0.780532142 | NA | NA | NA | NA |
| 251 | Efpeglenatide_high_dosage:Tirzepatide_high_dosage | 0 | -0.613009201 | NA | -0.613009201 | NA | NA | NA | NA |
| 252 | Efpeglenatide_high_dosage:Tirzepatide_low_dosage | 0 | -0.410726144 | NA | -0.410726144 | NA | NA | NA | NA |
| 253 | Efpeglenatide_high_dosage:Tirzepatide_medium_dosage | 0 | -0.272066038 | NA | -0.272066038 | NA | NA | NA | NA |
| 254 | Efpeglenatide_low_dosage:Efpeglenatide_medium_dosage | 2 | 1.250538508 | 1.011279164 | 1.822043726 | -0.810764561 | -3.892942481 | 2.271413358 | 0.606156837 |
| 255 | Efpeglenatide_low_dosage:Empagliflozin_high_dosage | 0 | 0.343266659 | NA | 0.343266659 | NA | NA | NA | NA |
| 256 | Efpeglenatide_low_dosage:Empagliflozin_low_dosage | 0 | 0.503359537 | NA | 0.503359537 | NA | NA | NA | NA |
| 257 | Efpeglenatide_low_dosage:Ertugliflozin_high_dosage | 0 | 0.451540701 | NA | 0.451540701 | NA | NA | NA | NA |
| 258 | Efpeglenatide_low_dosage:Ertugliflozin_low_dosage | 0 | 0.429896064 | NA | 0.429896064 | NA | NA | NA | NA |
| 259 | Efpeglenatide_low_dosage:Exenatide | 0 | 0.357287985 | NA | 0.357287985 | NA | NA | NA | NA |
| 260 | Efpeglenatide_low_dosage:Inject_semaglutide_high_dosage | 0 | 0.784360282 | NA | 0.784360282 | NA | NA | NA | NA |
| 261 | Efpeglenatide_low_dosage:Inject_semaglutide_low_dosage | 0 | 1.047548716 | NA | 1.047548716 | NA | NA | NA | NA |
| 262 | Efpeglenatide_low_dosage:Inject_semaglutide_medium_dosage | 0 | 0.39603508 | NA | 0.39603508 | NA | NA | NA | NA |
| 263 | Efpeglenatide_low_dosage:Liraglutide | 0 | 0.512782762 | NA | 0.512782762 | NA | NA | NA | NA |
| 264 | Efpeglenatide_low_dosage:Lixisenatide | 0 | -0.769957819 | NA | -0.769957819 | NA | NA | NA | NA |
| 265 | Efpeglenatide_low_dosage:Oral_semaglutide | 0 | 0.991181923 | NA | 0.991181923 | NA | NA | NA | NA |
| 266 | Efpeglenatide_low_dosage:Placebo_or_Control | 2 | 0.639027954 | 1.003709856 | 0.294626834 | 0.709083023 | -2.14846906 | 3.566635105 | 0.626717365 |
| 267 | Efpeglenatide_low_dosage:Sotagliflozin | 0 | 1.171571839 | NA | 1.171571839 | NA | NA | NA | NA |
| 268 | Efpeglenatide_low_dosage:Tirzepatide_high_dosage | 0 | -0.221969505 | NA | -0.221969505 | NA | NA | NA | NA |
| 269 | Efpeglenatide_low_dosage:Tirzepatide_low_dosage | 0 | -0.019686448 | NA | -0.019686448 | NA | NA | NA | NA |
| 270 | Efpeglenatide_low_dosage:Tirzepatide_medium_dosage | 0 | 0.118973659 | NA | 0.118973659 | NA | NA | NA | NA |
| 271 | Efpeglenatide_medium_dosage:Empagliflozin_high_dosage | 0 | -0.907271849 | NA | -0.907271849 | NA | NA | NA | NA |
| 272 | Efpeglenatide_medium_dosage:Empagliflozin_low_dosage | 0 | -0.747178971 | NA | -0.747178971 | NA | NA | NA | NA |
| 273 | Efpeglenatide_medium_dosage:Ertugliflozin_high_dosage | 0 | -0.798997807 | NA | -0.798997807 | NA | NA | NA | NA |
| 274 | Efpeglenatide_medium_dosage:Ertugliflozin_low_dosage | 0 | -0.820642445 | NA | -0.820642445 | NA | NA | NA | NA |
| 275 | Efpeglenatide_medium_dosage:Exenatide | 0 | -0.893250523 | NA | -0.893250523 | NA | NA | NA | NA |
| 276 | Efpeglenatide_medium_dosage:Inject_semaglutide_high_dosage | 0 | -0.466178226 | NA | -0.466178226 | NA | NA | NA | NA |
| 277 | Efpeglenatide_medium_dosage:Inject_semaglutide_low_dosage | 0 | -0.202989792 | NA | -0.202989792 | NA | NA | NA | NA |
| 278 | Efpeglenatide_medium_dosage:Inject_semaglutide_medium_dosage | 0 | -0.854503428 | NA | -0.854503428 | NA | NA | NA | NA |
| 279 | Efpeglenatide_medium_dosage:Liraglutide | 0 | -0.737755746 | NA | -0.737755746 | NA | NA | NA | NA |
| 280 | Efpeglenatide_medium_dosage:Lixisenatide | 0 | -2.020496327 | NA | -2.020496327 | NA | NA | NA | NA |
| 281 | Efpeglenatide_medium_dosage:Oral_semaglutide | 0 | -0.259356585 | NA | -0.259356585 | NA | NA | NA | NA |
| 282 | Efpeglenatide_medium_dosage:Placebo_or_Control | 3 | -0.611510554 | -0.337478568 | -1.538506482 | 1.201027913 | -2.265778488 | 4.667834315 | 0.497136051 |
| 283 | Efpeglenatide_medium_dosage:Sotagliflozin | 0 | -0.07896667 | NA | -0.07896667 | NA | NA | NA | NA |
| 284 | Efpeglenatide_medium_dosage:Tirzepatide_high_dosage | 0 | -1.472508013 | NA | -1.472508013 | NA | NA | NA | NA |
| 285 | Efpeglenatide_medium_dosage:Tirzepatide_low_dosage | 0 | -1.270224956 | NA | -1.270224956 | NA | NA | NA | NA |
| 286 | Efpeglenatide_medium_dosage:Tirzepatide_medium_dosage | 0 | -1.131564849 | NA | -1.131564849 | NA | NA | NA | NA |
| 287 | Empagliflozin_high_dosage:Empagliflozin_low_dosage | 6 | 0.160092878 | 0.353703468 | -0.137575038 | 0.491278506 | -0.980167232 | 1.962724245 | 0.512865477 |
| 288 | Empagliflozin_high_dosage:Ertugliflozin_high_dosage | 0 | 0.108274043 | NA | 0.108274043 | NA | NA | NA | NA |
| 289 | Empagliflozin_high_dosage:Ertugliflozin_low_dosage | 0 | 0.086629405 | NA | 0.086629405 | NA | NA | NA | NA |
| 290 | Empagliflozin_high_dosage:Exenatide | 0 | 0.014021326 | NA | 0.014021326 | NA | NA | NA | NA |
| 291 | Empagliflozin_high_dosage:Inject_semaglutide_high_dosage | 0 | 0.441093623 | NA | 0.441093623 | NA | NA | NA | NA |
| 292 | Empagliflozin_high_dosage:Inject_semaglutide_low_dosage | 0 | 0.704282057 | NA | 0.704282057 | NA | NA | NA | NA |
| 293 | Empagliflozin_high_dosage:Inject_semaglutide_medium_dosage | 0 | 0.052768421 | NA | 0.052768421 | NA | NA | NA | NA |
| 294 | Empagliflozin_high_dosage:Liraglutide | 0 | 0.169516103 | NA | 0.169516103 | NA | NA | NA | NA |
| 295 | Empagliflozin_high_dosage:Lixisenatide | 0 | -1.113224477 | NA | -1.113224477 | NA | NA | NA | NA |
| 296 | Empagliflozin_high_dosage:Oral_semaglutide | 1 | 0.647915264 | 0.019802627 | 0.760437417 | -0.74063479 | -3.757937041 | 2.276667461 | 0.630446982 |
| 297 | Empagliflozin_high_dosage:Placebo_or_Control | 6 | 0.295761295 | 0.298784539 | 0.289526271 | 0.009258268 | -1.468185236 | 1.486701771 | 0.990200677 |
| 298 | Empagliflozin_high_dosage:Sotagliflozin | 0 | 0.82830518 | NA | 0.82830518 | NA | NA | NA | NA |
| 299 | Empagliflozin_high_dosage:Tirzepatide_high_dosage | 0 | -0.565236164 | NA | -0.565236164 | NA | NA | NA | NA |
| 300 | Empagliflozin_high_dosage:Tirzepatide_low_dosage | 0 | -0.362953107 | NA | -0.362953107 | NA | NA | NA | NA |
| 301 | Empagliflozin_high_dosage:Tirzepatide_medium_dosage | 0 | -0.224293 | NA | -0.224293 | NA | NA | NA | NA |
| 302 | Empagliflozin_low_dosage:Ertugliflozin_high_dosage | 0 | -0.051818836 | NA | -0.051818836 | NA | NA | NA | NA |
| 303 | Empagliflozin_low_dosage:Ertugliflozin_low_dosage | 0 | -0.073463473 | NA | -0.073463473 | NA | NA | NA | NA |
| 304 | Empagliflozin_low_dosage:Exenatide | 0 | -0.146071552 | NA | -0.146071552 | NA | NA | NA | NA |
| 305 | Empagliflozin_low_dosage:Inject_semaglutide_high_dosage | 0 | 0.281000745 | NA | 0.281000745 | NA | NA | NA | NA |
| 306 | Empagliflozin_low_dosage:Inject_semaglutide_low_dosage | 0 | 0.544189179 | NA | 0.544189179 | NA | NA | NA | NA |
| 307 | Empagliflozin_low_dosage:Inject_semaglutide_medium_dosage | 0 | -0.107324457 | NA | -0.107324457 | NA | NA | NA | NA |
| 308 | Empagliflozin_low_dosage:Liraglutide | 0 | 0.009423225 | NA | 0.009423225 | NA | NA | NA | NA |
| 309 | Empagliflozin_low_dosage:Lixisenatide | 0 | -1.273317356 | NA | -1.273317356 | NA | NA | NA | NA |
| 310 | Empagliflozin_low_dosage:Oral_semaglutide | 0 | 0.487822386 | NA | 0.487822386 | NA | NA | NA | NA |
| 311 | Empagliflozin_low_dosage:Placebo_or_Control | 8 | 0.135668417 | 0.185161768 | -0.456711074 | 0.641872841 | -1.255381004 | 2.539126687 | 0.507273725 |
| 312 | Empagliflozin_low_dosage:Sotagliflozin | 0 | 0.668212302 | NA | 0.668212302 | NA | NA | NA | NA |
| 313 | Empagliflozin_low_dosage:Tirzepatide_high_dosage | 0 | -0.725329042 | NA | -0.725329042 | NA | NA | NA | NA |
| 314 | Empagliflozin_low_dosage:Tirzepatide_low_dosage | 0 | -0.523045985 | NA | -0.523045985 | NA | NA | NA | NA |
| 315 | Empagliflozin_low_dosage:Tirzepatide_medium_dosage | 0 | -0.384385878 | NA | -0.384385878 | NA | NA | NA | NA |
| 316 | Ertugliflozin_high_dosage:Ertugliflozin_low_dosage | 2 | -0.021644638 | -0.021651763 | NA | NA | NA | NA | NA |
| 317 | Ertugliflozin_high_dosage:Exenatide | 0 | -0.094252717 | NA | -0.094252717 | NA | NA | NA | NA |
| 318 | Ertugliflozin_high_dosage:Inject_semaglutide_high_dosage | 0 | 0.332819581 | NA | 0.332819581 | NA | NA | NA | NA |
| 319 | Ertugliflozin_high_dosage:Inject_semaglutide_low_dosage | 0 | 0.596008015 | NA | 0.596008015 | NA | NA | NA | NA |
| 320 | Ertugliflozin_high_dosage:Inject_semaglutide_medium_dosage | 0 | -0.055505622 | NA | -0.055505622 | NA | NA | NA | NA |
| 321 | Ertugliflozin_high_dosage:Liraglutide | 0 | 0.06124206 | NA | 0.06124206 | NA | NA | NA | NA |
| 322 | Ertugliflozin_high_dosage:Lixisenatide | 0 | -1.22149852 | NA | -1.22149852 | NA | NA | NA | NA |
| 323 | Ertugliflozin_high_dosage:Oral_semaglutide | 0 | 0.539641221 | NA | 0.539641221 | NA | NA | NA | NA |
| 324 | Ertugliflozin_high_dosage:Placebo_or_Control | 2 | 0.187487252 | 0.188661175 | NA | NA | NA | NA | NA |
| 325 | Ertugliflozin_high_dosage:Sotagliflozin | 0 | 0.720031137 | NA | 0.720031137 | NA | NA | NA | NA |
| 326 | Ertugliflozin_high_dosage:Tirzepatide_high_dosage | 0 | -0.673510206 | NA | -0.673510206 | NA | NA | NA | NA |
| 327 | Ertugliflozin_high_dosage:Tirzepatide_low_dosage | 0 | -0.47122715 | NA | -0.47122715 | NA | NA | NA | NA |
| 328 | Ertugliflozin_high_dosage:Tirzepatide_medium_dosage | 0 | -0.332567043 | NA | -0.332567043 | NA | NA | NA | NA |
| 329 | Ertugliflozin_low_dosage:Exenatide | 0 | -0.072608079 | NA | -0.072608079 | NA | NA | NA | NA |
| 330 | Ertugliflozin_low_dosage:Inject_semaglutide_high_dosage | 0 | 0.354464219 | NA | 0.354464219 | NA | NA | NA | NA |
| 331 | Ertugliflozin_low_dosage:Inject_semaglutide_low_dosage | 0 | 0.617652652 | NA | 0.617652652 | NA | NA | NA | NA |
| 332 | Ertugliflozin_low_dosage:Inject_semaglutide_medium_dosage | 0 | -0.033860984 | NA | -0.033860984 | NA | NA | NA | NA |
| 333 | Ertugliflozin_low_dosage:Liraglutide | 0 | 0.082886698 | NA | 0.082886698 | NA | NA | NA | NA |
| 334 | Ertugliflozin_low_dosage:Lixisenatide | 0 | -1.199853882 | NA | -1.199853882 | NA | NA | NA | NA |
| 335 | Ertugliflozin_low_dosage:Oral_semaglutide | 0 | 0.561285859 | NA | 0.561285859 | NA | NA | NA | NA |
| 336 | Ertugliflozin_low_dosage:Placebo_or_Control | 2 | 0.20913189 | 0.208897589 | NA | NA | NA | NA | NA |
| 337 | Ertugliflozin_low_dosage:Sotagliflozin | 0 | 0.741675775 | NA | 0.741675775 | NA | NA | NA | NA |
| 338 | Ertugliflozin_low_dosage:Tirzepatide_high_dosage | 0 | -0.651865568 | NA | -0.651865568 | NA | NA | NA | NA |
| 339 | Ertugliflozin_low_dosage:Tirzepatide_low_dosage | 0 | -0.449582512 | NA | -0.449582512 | NA | NA | NA | NA |
| 340 | Ertugliflozin_low_dosage:Tirzepatide_medium_dosage | 0 | -0.310922405 | NA | -0.310922405 | NA | NA | NA | NA |
| 341 | Exenatide:Inject_semaglutide_high_dosage | 0 | 0.427072298 | NA | 0.427072298 | NA | NA | NA | NA |
| 342 | Exenatide:Inject_semaglutide_low_dosage | 0 | 0.690260731 | NA | 0.690260731 | NA | NA | NA | NA |
| 343 | Exenatide:Inject_semaglutide_medium_dosage | 1 | 0.038747095 | -0.366244395 | 0.133623747 | -0.499868142 | -2.500943896 | 1.501207612 | 0.624418085 |
| 344 | Exenatide:Liraglutide | 0 | 0.155494777 | NA | 0.155494777 | NA | NA | NA | NA |
| 345 | Exenatide:Lixisenatide | 0 | -1.127245803 | NA | -1.127245803 | NA | NA | NA | NA |
| 346 | Exenatide:Oral_semaglutide | 0 | 0.633893938 | NA | 0.633893938 | NA | NA | NA | NA |
| 347 | Exenatide:Placebo_or_Control | 2 | 0.281739969 | 0.303623774 | -0.196244368 | 0.499868142 | -1.501207612 | 2.500943896 | 0.624418085 |
| 348 | Exenatide:Sotagliflozin | 0 | 0.814283854 | NA | 0.814283854 | NA | NA | NA | NA |
| 349 | Exenatide:Tirzepatide_high_dosage | 0 | -0.579257489 | NA | -0.579257489 | NA | NA | NA | NA |
| 350 | Exenatide:Tirzepatide_low_dosage | 0 | -0.376974433 | NA | -0.376974433 | NA | NA | NA | NA |
| 351 | Exenatide:Tirzepatide_medium_dosage | 0 | -0.238314326 | NA | -0.238314326 | NA | NA | NA | NA |
| 352 | Inject_semaglutide_high_dosage:Inject_semaglutide_low_dosage | 0 | 0.263188434 | NA | 0.263188434 | NA | NA | NA | NA |
| 353 | Inject_semaglutide_high_dosage:Inject_semaglutide_medium_dosage | 1 | -0.388325203 | -1.713059945 | -0.283591401 | -1.429468544 | -4.589771532 | 1.730834443 | 0.375331407 |
| 354 | Inject_semaglutide_high_dosage:Liraglutide | 1 | -0.271577521 | -0.754390806 | -0.241809268 | -0.512581538 | -3.002715979 | 1.977552904 | 0.686618211 |
| 355 | Inject_semaglutide_high_dosage:Lixisenatide | 0 | -1.554318101 | NA | -1.554318101 | NA | NA | NA | NA |
| 356 | Inject_semaglutide_high_dosage:Oral_semaglutide | 0 | 0.206821641 | NA | 0.206821641 | NA | NA | NA | NA |
| 357 | Inject_semaglutide_high_dosage:Placebo_or_Control | 7 | -0.145332329 | -0.106701716 | -1.280351054 | 1.173649338 | -1.337039368 | 3.684338044 | 0.359558363 |
| 358 | Inject_semaglutide_high_dosage:Sotagliflozin | 0 | 0.387211556 | NA | 0.387211556 | NA | NA | NA | NA |
| 359 | Inject_semaglutide_high_dosage:Tirzepatide_high_dosage | 0 | -1.006329787 | NA | -1.006329787 | NA | NA | NA | NA |
| 360 | Inject_semaglutide_high_dosage:Tirzepatide_low_dosage | 0 | -0.80404673 | NA | -0.80404673 | NA | NA | NA | NA |
| 361 | Inject_semaglutide_high_dosage:Tirzepatide_medium_dosage | 0 | -0.665386624 | NA | -0.665386624 | NA | NA | NA | NA |
| 362 | Inject_semaglutide_low_dosage:Inject_semaglutide_medium_dosage | 2 | -0.651513636 | -0.501798575 | -0.952560598 | 0.450762023 | -1.769735976 | 2.671260022 | 0.690723428 |
| 363 | Inject_semaglutide_low_dosage:Liraglutide | 1 | -0.534765954 | -1.941604437 | -0.17634598 | -1.765258457 | -4.316678637 | 0.786161723 | 0.175084549 |
| 364 | Inject_semaglutide_low_dosage:Lixisenatide | 0 | -1.817506535 | NA | -1.817506535 | NA | NA | NA | NA |
| 365 | Inject_semaglutide_low_dosage:Oral_semaglutide | 0 | -0.056366793 | NA | -0.056366793 | NA | NA | NA | NA |
| 366 | Inject_semaglutide_low_dosage:Placebo_or_Control | 4 | -0.408520762 | -0.173640816 | -1.188090868 | 1.014450052 | -1.300011122 | 3.328911226 | 0.390301562 |
| 367 | Inject_semaglutide_low_dosage:Sotagliflozin | 0 | 0.124023123 | NA | 0.124023123 | NA | NA | NA | NA |
| 368 | Inject_semaglutide_low_dosage:Tirzepatide_high_dosage | 0 | -1.269518221 | NA | -1.269518221 | NA | NA | NA | NA |
| 369 | Inject_semaglutide_low_dosage:Tirzepatide_low_dosage | 0 | -1.067235164 | NA | -1.067235164 | NA | NA | NA | NA |
| 370 | Inject_semaglutide_low_dosage:Tirzepatide_medium_dosage | 0 | -0.928575057 | NA | -0.928575057 | NA | NA | NA | NA |
| 371 | Inject_semaglutide_medium_dosage:Liraglutide | 0 | 0.116747682 | NA | 0.116747682 | NA | NA | NA | NA |
| 372 | Inject_semaglutide_medium_dosage:Lixisenatide | 0 | -1.165992898 | NA | -1.165992898 | NA | NA | NA | NA |
| 373 | Inject_semaglutide_medium_dosage:Oral_semaglutide | 0 | 0.595146843 | NA | 0.595146843 | NA | NA | NA | NA |
| 374 | Inject_semaglutide_medium_dosage:Placebo_or_Control | 4 | 0.242992874 | 0.318210667 | 0.097592492 | 0.220618175 | -1.270104835 | 1.711341185 | 0.771767996 |
| 375 | Inject_semaglutide_medium_dosage:Sotagliflozin | 0 | 0.775536759 | NA | 0.775536759 | NA | NA | NA | NA |
| 376 | Inject_semaglutide_medium_dosage:Tirzepatide_high_dosage | 1 | -0.618004584 | -1.054605188 | -0.567331358 | -0.48727383 | -3.873590869 | 2.899043209 | 0.777921376 |
| 377 | Inject_semaglutide_medium_dosage:Tirzepatide_low_dosage | 1 | -0.415721528 | -1.534599853 | -0.243176586 | -1.291423268 | -4.558872427 | 1.976025891 | 0.438544043 |
| 378 | Inject_semaglutide_medium_dosage:Tirzepatide_medium_dosage | 0 | -0.277061421 | NA | -0.277061421 | NA | NA | NA | NA |
| 379 | Liraglutide:Lixisenatide | 0 | -1.28274058 | NA | -1.28274058 | NA | NA | NA | NA |
| 380 | Liraglutide:Oral_semaglutide | 1 | 0.478399161 | 0.022059718 | 0.574238611 | -0.552178893 | -3.055378832 | 1.951021045 | 0.665489302 |
| 381 | Liraglutide:Placebo_or_Control | 10 | 0.126245192 | 0.104017186 | 0.643453443 | -0.539436256 | -2.463511797 | 1.384639285 | 0.582663746 |
| 382 | Liraglutide:Sotagliflozin | 0 | 0.658789077 | NA | 0.658789077 | NA | NA | NA | NA |
| 383 | Liraglutide:Tirzepatide_high_dosage | 0 | -0.734752266 | NA | -0.734752266 | NA | NA | NA | NA |
| 384 | Liraglutide:Tirzepatide_low_dosage | 0 | -0.53246921 | NA | -0.53246921 | NA | NA | NA | NA |
| 385 | Liraglutide:Tirzepatide_medium_dosage | 0 | -0.393809103 | NA | -0.393809103 | NA | NA | NA | NA |
| 386 | Lixisenatide:Oral_semaglutide | 0 | 1.761139741 | NA | 1.761139741 | NA | NA | NA | NA |
| 387 | Lixisenatide:Placebo_or_Control | 1 | 1.408985772 | 1.408985772 | NA | NA | NA | NA | NA |
| 388 | Lixisenatide:Sotagliflozin | 0 | 1.941529657 | NA | 1.941529657 | NA | NA | NA | NA |
| 389 | Lixisenatide:Tirzepatide_high_dosage | 0 | 0.547988314 | NA | 0.547988314 | NA | NA | NA | NA |
| 390 | Lixisenatide:Tirzepatide_low_dosage | 0 | 0.75027137 | NA | 0.75027137 | NA | NA | NA | NA |
| 391 | Lixisenatide:Tirzepatide_medium_dosage | 0 | 0.888931477 | NA | 0.888931477 | NA | NA | NA | NA |
| 392 | Oral_semaglutide:Placebo_or_Control | 6 | -0.352153969 | -0.474880529 | 0.167862493 | -0.642743022 | -2.905098526 | 1.619612481 | 0.577641845 |
| 393 | Oral_semaglutide:Sotagliflozin | 0 | 0.180389916 | NA | 0.180389916 | NA | NA | NA | NA |
| 394 | Oral_semaglutide:Tirzepatide_high_dosage | 0 | -1.213151428 | NA | -1.213151428 | NA | NA | NA | NA |
| 395 | Oral_semaglutide:Tirzepatide_low_dosage | 0 | -1.010868371 | NA | -1.010868371 | NA | NA | NA | NA |
| 396 | Oral_semaglutide:Tirzepatide_medium_dosage | 0 | -0.872208264 | NA | -0.872208264 | NA | NA | NA | NA |
| 397 | Sotagliflozin:Placebo_or_Control | 6 | -0.532543885 | -0.532543885 | NA | NA | NA | NA | NA |
| 398 | Tirzepatide_high_dosage:Placebo_or_Control | 5 | 0.860997458 | 0.610411913 | 1.510802752 | -0.900390839 | -2.739278386 | 0.938496709 | 0.337219026 |
| 399 | Tirzepatide_low_dosage:Placebo_or_Control | 3 | 0.658714402 | 0.176629937 | 1.277270937 | -1.100641 | -2.980186974 | 0.778904974 | 0.251078852 |
| 400 | Tirzepatide_medium_dosage:Placebo_or_Control | 5 | 0.520054295 | 0.455365881 | 0.608569303 | -0.153203422 | -2.068355793 | 1.76194895 | 0.875411816 |
| 401 | Sotagliflozin:Tirzepatide_high_dosage | 0 | -1.393541343 | NA | -1.393541343 | NA | NA | NA | NA |
| 402 | Sotagliflozin:Tirzepatide_low_dosage | 0 | -1.191258287 | NA | -1.191258287 | NA | NA | NA | NA |
| 403 | Sotagliflozin:Tirzepatide_medium_dosage | 0 | -1.05259818 | NA | -1.05259818 | NA | NA | NA | NA |
| 404 | Tirzepatide_high_dosage:Tirzepatide_low_dosage | 6 | 0.202283057 | 0.161584709 | 0.310708158 | -0.14912345 | -2.037830721 | 1.739583821 | 0.877018769 |
| 405 | Tirzepatide_high_dosage:Tirzepatide_medium_dosage | 9 | 0.340943163 | 0.539515621 | -0.21254938 | 0.752065002 | -1.137006883 | 2.641136886 | 0.435221314 |
| 406 | Tirzepatide_low_dosage:Tirzepatide_medium_dosage | 5 | 0.138660107 | 0.146715097 | 0.130251507 | 0.01646359 | -1.902486309 | 1.935413489 | 0.986583829 |

**eTable 7B: Side-splitting model inconsistency of primary outcome: subgroup of intra-uterus tumor**

|  | Comparison | No.Studies | NMA | Direct | Indirect | Difference | Diff_95CI_lower | Diff_95CI_upper | pValue |
| --- | --- | --- | --- | --- | --- | --- | --- | --- | --- |
| 1 | Albiglutide:Bexagliflozin | 0 | 0.414300815 | NA | 0.414300815 | NA | NA | NA | NA |
| 2 | Albiglutide:Canagliflozin_high_dosage | 0 | -0.577999192 | NA | -0.577999192 | NA | NA | NA | NA |
| 3 | Albiglutide:Canagliflozin_low_dosage | 0 | -0.088148249 | NA | -0.088148249 | NA | NA | NA | NA |
| 4 | Albiglutide:Dapagliflozin_high_dosage | 0 | -0.02073425 | NA | -0.02073425 | NA | NA | NA | NA |
| 5 | Albiglutide:Dulaglutide_medium_dosage | 0 | -0.063103725 | NA | -0.063103725 | NA | NA | NA | NA |
| 6 | Albiglutide:Efpeglenatide_high_dosage | 0 | -0.168387014 | NA | -0.168387014 | NA | NA | NA | NA |
| 7 | Albiglutide:Efpeglenatide_low_dosage | 0 | 0.553196826 | NA | 0.553196826 | NA | NA | NA | NA |
| 8 | Albiglutide:Efpeglenatide_medium_dosage | 0 | -0.48156399 | NA | -0.48156399 | NA | NA | NA | NA |
| 9 | Albiglutide:Empagliflozin_high_dosage | 0 | -0.871623595 | NA | -0.871623595 | NA | NA | NA | NA |
| 10 | Albiglutide:Empagliflozin_low_dosage | 0 | -0.83840292 | NA | -0.83840292 | NA | NA | NA | NA |
| 11 | Albiglutide:Ertugliflozin_high_dosage | 0 | 0.435836154 | NA | 0.435836154 | NA | NA | NA | NA |
| 12 | Albiglutide:Ertugliflozin_low_dosage | 0 | -0.703297332 | NA | -0.703297332 | NA | NA | NA | NA |
| 13 | Albiglutide:Exenatide | 0 | -0.619113531 | NA | -0.619113531 | NA | NA | NA | NA |
| 14 | Albiglutide:Inject_semaglutide_high_dosage | 0 | 0.14558519 | NA | 0.14558519 | NA | NA | NA | NA |
| 15 | Albiglutide:Inject_semaglutide_low_dosage | 0 | 0.967747068 | NA | 0.967747068 | NA | NA | NA | NA |
| 16 | Albiglutide:Inject_semaglutide_medium_dosage | 0 | -0.639459707 | NA | -0.639459707 | NA | NA | NA | NA |
| 17 | Albiglutide:Liraglutide | 0 | 0.499155419 | NA | 0.499155419 | NA | NA | NA | NA |
| 18 | Albiglutide:Lixisenatide | 0 | -1.359968576 | NA | -1.359968576 | NA | NA | NA | NA |
| 19 | Albiglutide:Oral_semaglutide | 0 | -0.11470578 | NA | -0.11470578 | NA | NA | NA | NA |
| 20 | Albiglutide:Orforglipron | 0 | 0.217950958 | NA | 0.217950958 | NA | NA | NA | NA |
| 21 | Albiglutide:Placebo_or_Control | 5 | -0.244160807 | -0.244160807 | NA | NA | NA | NA | NA |
| 22 | Albiglutide:Sotagliflozin | 0 | 0.297613497 | NA | 0.297613497 | NA | NA | NA | NA |
| 23 | Albiglutide:Tirzepatide_high_dosage | 0 | -1.78189548 | NA | -1.78189548 | NA | NA | NA | NA |
| 24 | Albiglutide:Tirzepatide_low_dosage | 0 | -1.771450366 | NA | -1.771450366 | NA | NA | NA | NA |
| 25 | Albiglutide:Tirzepatide_medium_dosage | 0 | -1.110789121 | NA | -1.110789121 | NA | NA | NA | NA |
| 26 | Bexagliflozin:Canagliflozin_high_dosage | 0 | -0.992300007 | NA | -0.992300007 | NA | NA | NA | NA |
| 27 | Bexagliflozin:Canagliflozin_low_dosage | 0 | -0.502449064 | NA | -0.502449064 | NA | NA | NA | NA |
| 28 | Bexagliflozin:Dapagliflozin_high_dosage | 0 | -0.435035065 | NA | -0.435035065 | NA | NA | NA | NA |
| 29 | Bexagliflozin:Dulaglutide_medium_dosage | 0 | -0.477404541 | NA | -0.477404541 | NA | NA | NA | NA |
| 30 | Bexagliflozin:Efpeglenatide_high_dosage | 0 | -0.582687829 | NA | -0.582687829 | NA | NA | NA | NA |
| 31 | Bexagliflozin:Efpeglenatide_low_dosage | 0 | 0.138896011 | NA | 0.138896011 | NA | NA | NA | NA |
| 32 | Bexagliflozin:Efpeglenatide_medium_dosage | 0 | -0.895864806 | NA | -0.895864806 | NA | NA | NA | NA |
| 33 | Bexagliflozin:Empagliflozin_high_dosage | 0 | -1.28592441 | NA | -1.28592441 | NA | NA | NA | NA |
| 34 | Bexagliflozin:Empagliflozin_low_dosage | 0 | -1.252703736 | NA | -1.252703736 | NA | NA | NA | NA |
| 35 | Bexagliflozin:Ertugliflozin_high_dosage | 0 | 0.021535339 | NA | 0.021535339 | NA | NA | NA | NA |
| 36 | Bexagliflozin:Ertugliflozin_low_dosage | 0 | -1.117598147 | NA | -1.117598147 | NA | NA | NA | NA |
| 37 | Bexagliflozin:Exenatide | 0 | -1.033414346 | NA | -1.033414346 | NA | NA | NA | NA |
| 38 | Bexagliflozin:Inject_semaglutide_high_dosage | 0 | -0.268715625 | NA | -0.268715625 | NA | NA | NA | NA |
| 39 | Bexagliflozin:Inject_semaglutide_low_dosage | 0 | 0.553446253 | NA | 0.553446253 | NA | NA | NA | NA |
| 40 | Bexagliflozin:Inject_semaglutide_medium_dosage | 0 | -1.053760522 | NA | -1.053760522 | NA | NA | NA | NA |
| 41 | Bexagliflozin:Liraglutide | 0 | 0.084854604 | NA | 0.084854604 | NA | NA | NA | NA |
| 42 | Bexagliflozin:Lixisenatide | 0 | -1.774269391 | NA | -1.774269391 | NA | NA | NA | NA |
| 43 | Bexagliflozin:Oral_semaglutide | 0 | -0.529006596 | NA | -0.529006596 | NA | NA | NA | NA |
| 44 | Bexagliflozin:Orforglipron | 0 | -0.196349858 | NA | -0.196349858 | NA | NA | NA | NA |
| 45 | Bexagliflozin:Placebo_or_Control | 1 | -0.658461623 | -0.658461623 | NA | NA | NA | NA | NA |
| 46 | Bexagliflozin:Sotagliflozin | 0 | -0.116687318 | NA | -0.116687318 | NA | NA | NA | NA |
| 47 | Bexagliflozin:Tirzepatide_high_dosage | 0 | -2.196196295 | NA | -2.196196295 | NA | NA | NA | NA |
| 48 | Bexagliflozin:Tirzepatide_low_dosage | 0 | -2.185751181 | NA | -2.185751181 | NA | NA | NA | NA |
| 49 | Bexagliflozin:Tirzepatide_medium_dosage | 0 | -1.525089936 | NA | -1.525089936 | NA | NA | NA | NA |
| 50 | Canagliflozin_high_dosage:Canagliflozin_low_dosage | 1 | 0.489850943 | 0.484157377 | 0.505379053 | -0.021221676 | -2.79524035 | 2.752796998 | 0.988036943 |
| 51 | Canagliflozin_high_dosage:Dapagliflozin_high_dosage | 0 | 0.557264942 | NA | 0.557264942 | NA | NA | NA | NA |
| 52 | Canagliflozin_high_dosage:Dulaglutide_medium_dosage | 0 | 0.514895466 | NA | 0.514895466 | NA | NA | NA | NA |
| 53 | Canagliflozin_high_dosage:Efpeglenatide_high_dosage | 0 | 0.409612178 | NA | 0.409612178 | NA | NA | NA | NA |
| 54 | Canagliflozin_high_dosage:Efpeglenatide_low_dosage | 0 | 1.131196018 | NA | 1.131196018 | NA | NA | NA | NA |
| 55 | Canagliflozin_high_dosage:Efpeglenatide_medium_dosage | 0 | 0.096435201 | NA | 0.096435201 | NA | NA | NA | NA |
| 56 | Canagliflozin_high_dosage:Empagliflozin_high_dosage | 0 | -0.293624403 | NA | -0.293624403 | NA | NA | NA | NA |
| 57 | Canagliflozin_high_dosage:Empagliflozin_low_dosage | 0 | -0.260403729 | NA | -0.260403729 | NA | NA | NA | NA |
| 58 | Canagliflozin_high_dosage:Ertugliflozin_high_dosage | 0 | 1.013835346 | NA | 1.013835346 | NA | NA | NA | NA |
| 59 | Canagliflozin_high_dosage:Ertugliflozin_low_dosage | 0 | -0.12529814 | NA | -0.12529814 | NA | NA | NA | NA |
| 60 | Canagliflozin_high_dosage:Exenatide | 0 | -0.041114339 | NA | -0.041114339 | NA | NA | NA | NA |
| 61 | Canagliflozin_high_dosage:Inject_semaglutide_high_dosage | 0 | 0.723584382 | NA | 0.723584382 | NA | NA | NA | NA |
| 62 | Canagliflozin_high_dosage:Inject_semaglutide_low_dosage | 0 | 1.54574626 | NA | 1.54574626 | NA | NA | NA | NA |
| 63 | Canagliflozin_high_dosage:Inject_semaglutide_medium_dosage | 0 | -0.061460515 | NA | -0.061460515 | NA | NA | NA | NA |
| 64 | Canagliflozin_high_dosage:Liraglutide | 0 | 1.077154611 | NA | 1.077154611 | NA | NA | NA | NA |
| 65 | Canagliflozin_high_dosage:Lixisenatide | 0 | -0.781969384 | NA | -0.781969384 | NA | NA | NA | NA |
| 66 | Canagliflozin_high_dosage:Oral_semaglutide | 0 | 0.463293411 | NA | 0.463293411 | NA | NA | NA | NA |
| 67 | Canagliflozin_high_dosage:Orforglipron | 0 | 0.795950149 | NA | 0.795950149 | NA | NA | NA | NA |
| 68 | Canagliflozin_high_dosage:Placebo_or_Control | 3 | 0.333838384 | 0.416851704 | -0.308518389 | 0.725370093 | -2.624679048 | 4.075419233 | 0.671287528 |
| 69 | Canagliflozin_high_dosage:Sotagliflozin | 0 | 0.875612689 | NA | 0.875612689 | NA | NA | NA | NA |
| 70 | Canagliflozin_high_dosage:Tirzepatide_high_dosage | 0 | -1.203896288 | NA | -1.203896288 | NA | NA | NA | NA |
| 71 | Canagliflozin_high_dosage:Tirzepatide_low_dosage | 0 | -1.193451174 | NA | -1.193451174 | NA | NA | NA | NA |
| 72 | Canagliflozin_high_dosage:Tirzepatide_medium_dosage | 0 | -0.532789929 | NA | -0.532789929 | NA | NA | NA | NA |
| 73 | Canagliflozin_low_dosage:Dapagliflozin_high_dosage | 0 | 0.067413999 | NA | 0.067413999 | NA | NA | NA | NA |
| 74 | Canagliflozin_low_dosage:Dulaglutide_medium_dosage | 0 | 0.025044524 | NA | 0.025044524 | NA | NA | NA | NA |
| 75 | Canagliflozin_low_dosage:Efpeglenatide_high_dosage | 0 | -0.080238764 | NA | -0.080238764 | NA | NA | NA | NA |
| 76 | Canagliflozin_low_dosage:Efpeglenatide_low_dosage | 0 | 0.641345075 | NA | 0.641345075 | NA | NA | NA | NA |
| 77 | Canagliflozin_low_dosage:Efpeglenatide_medium_dosage | 0 | -0.393415741 | NA | -0.393415741 | NA | NA | NA | NA |
| 78 | Canagliflozin_low_dosage:Empagliflozin_high_dosage | 0 | -0.783475346 | NA | -0.783475346 | NA | NA | NA | NA |
| 79 | Canagliflozin_low_dosage:Empagliflozin_low_dosage | 0 | -0.750254671 | NA | -0.750254671 | NA | NA | NA | NA |
| 80 | Canagliflozin_low_dosage:Ertugliflozin_high_dosage | 0 | 0.523984403 | NA | 0.523984403 | NA | NA | NA | NA |
| 81 | Canagliflozin_low_dosage:Ertugliflozin_low_dosage | 0 | -0.615149083 | NA | -0.615149083 | NA | NA | NA | NA |
| 82 | Canagliflozin_low_dosage:Exenatide | 0 | -0.530965282 | NA | -0.530965282 | NA | NA | NA | NA |
| 83 | Canagliflozin_low_dosage:Inject_semaglutide_high_dosage | 0 | 0.233733439 | NA | 0.233733439 | NA | NA | NA | NA |
| 84 | Canagliflozin_low_dosage:Inject_semaglutide_low_dosage | 0 | 1.055895317 | NA | 1.055895317 | NA | NA | NA | NA |
| 85 | Canagliflozin_low_dosage:Inject_semaglutide_medium_dosage | 0 | -0.551311458 | NA | -0.551311458 | NA | NA | NA | NA |
| 86 | Canagliflozin_low_dosage:Liraglutide | 0 | 0.587303668 | NA | 0.587303668 | NA | NA | NA | NA |
| 87 | Canagliflozin_low_dosage:Lixisenatide | 0 | -1.271820327 | NA | -1.271820327 | NA | NA | NA | NA |
| 88 | Canagliflozin_low_dosage:Oral_semaglutide | 0 | -0.026557531 | NA | -0.026557531 | NA | NA | NA | NA |
| 89 | Canagliflozin_low_dosage:Orforglipron | 0 | 0.306099207 | NA | 0.306099207 | NA | NA | NA | NA |
| 90 | Canagliflozin_low_dosage:Placebo_or_Control | 2 | -0.156012558 | -0.018087528 | -1.109785548 | 1.091698019 | -2.47495136 | 4.658347399 | 0.548562448 |
| 91 | Canagliflozin_low_dosage:Sotagliflozin | 0 | 0.385761746 | NA | 0.385761746 | NA | NA | NA | NA |
| 92 | Canagliflozin_low_dosage:Tirzepatide_high_dosage | 0 | -1.693747231 | NA | -1.693747231 | NA | NA | NA | NA |
| 93 | Canagliflozin_low_dosage:Tirzepatide_low_dosage | 0 | -1.683302117 | NA | -1.683302117 | NA | NA | NA | NA |
| 94 | Canagliflozin_low_dosage:Tirzepatide_medium_dosage | 0 | -1.022640872 | NA | -1.022640872 | NA | NA | NA | NA |
| 95 | Dapagliflozin_high_dosage:Dulaglutide_medium_dosage | 0 | -0.042369476 | NA | -0.042369476 | NA | NA | NA | NA |
| 96 | Dapagliflozin_high_dosage:Efpeglenatide_high_dosage | 0 | -0.147652764 | NA | -0.147652764 | NA | NA | NA | NA |
| 97 | Dapagliflozin_high_dosage:Efpeglenatide_low_dosage | 0 | 0.573931076 | NA | 0.573931076 | NA | NA | NA | NA |
| 98 | Dapagliflozin_high_dosage:Efpeglenatide_medium_dosage | 0 | -0.460829741 | NA | -0.460829741 | NA | NA | NA | NA |
| 99 | Dapagliflozin_high_dosage:Empagliflozin_high_dosage | 0 | -0.850889345 | NA | -0.850889345 | NA | NA | NA | NA |
| 100 | Dapagliflozin_high_dosage:Empagliflozin_low_dosage | 0 | -0.817668671 | NA | -0.817668671 | NA | NA | NA | NA |
| 101 | Dapagliflozin_high_dosage:Ertugliflozin_high_dosage | 0 | 0.456570403 | NA | 0.456570403 | NA | NA | NA | NA |
| 102 | Dapagliflozin_high_dosage:Ertugliflozin_low_dosage | 0 | -0.682563083 | NA | -0.682563083 | NA | NA | NA | NA |
| 103 | Dapagliflozin_high_dosage:Exenatide | 0 | -0.598379281 | NA | -0.598379281 | NA | NA | NA | NA |
| 104 | Dapagliflozin_high_dosage:Inject_semaglutide_high_dosage | 0 | 0.16631944 | NA | 0.16631944 | NA | NA | NA | NA |
| 105 | Dapagliflozin_high_dosage:Inject_semaglutide_low_dosage | 0 | 0.988481318 | NA | 0.988481318 | NA | NA | NA | NA |
| 106 | Dapagliflozin_high_dosage:Inject_semaglutide_medium_dosage | 0 | -0.618725457 | NA | -0.618725457 | NA | NA | NA | NA |
| 107 | Dapagliflozin_high_dosage:Liraglutide | 0 | 0.519889668 | NA | 0.519889668 | NA | NA | NA | NA |
| 108 | Dapagliflozin_high_dosage:Lixisenatide | 0 | -1.339234327 | NA | -1.339234327 | NA | NA | NA | NA |
| 109 | Dapagliflozin_high_dosage:Oral_semaglutide | 0 | -0.093971531 | NA | -0.093971531 | NA | NA | NA | NA |
| 110 | Dapagliflozin_high_dosage:Orforglipron | 0 | 0.238685207 | NA | 0.238685207 | NA | NA | NA | NA |
| 111 | Dapagliflozin_high_dosage:Placebo_or_Control | 4 | -0.223426558 | -0.223426558 | NA | NA | NA | NA | NA |
| 112 | Dapagliflozin_high_dosage:Sotagliflozin | 0 | 0.318347747 | NA | 0.318347747 | NA | NA | NA | NA |
| 113 | Dapagliflozin_high_dosage:Tirzepatide_high_dosage | 0 | -1.76116123 | NA | -1.76116123 | NA | NA | NA | NA |
| 114 | Dapagliflozin_high_dosage:Tirzepatide_low_dosage | 0 | -1.750716116 | NA | -1.750716116 | NA | NA | NA | NA |
| 115 | Dapagliflozin_high_dosage:Tirzepatide_medium_dosage | 0 | -1.090054871 | NA | -1.090054871 | NA | NA | NA | NA |
| 116 | Dulaglutide_medium_dosage:Efpeglenatide_high_dosage | 0 | -0.105283288 | NA | -0.105283288 | NA | NA | NA | NA |
| 117 | Dulaglutide_medium_dosage:Efpeglenatide_low_dosage | 0 | 0.616300552 | NA | 0.616300552 | NA | NA | NA | NA |
| 118 | Dulaglutide_medium_dosage:Efpeglenatide_medium_dosage | 0 | -0.418460265 | NA | -0.418460265 | NA | NA | NA | NA |
| 119 | Dulaglutide_medium_dosage:Empagliflozin_high_dosage | 0 | -0.808519869 | NA | -0.808519869 | NA | NA | NA | NA |
| 120 | Dulaglutide_medium_dosage:Empagliflozin_low_dosage | 0 | -0.775299195 | NA | -0.775299195 | NA | NA | NA | NA |
| 121 | Dulaglutide_medium_dosage:Ertugliflozin_high_dosage | 0 | 0.498939879 | NA | 0.498939879 | NA | NA | NA | NA |
| 122 | Dulaglutide_medium_dosage:Ertugliflozin_low_dosage | 0 | -0.640193607 | NA | -0.640193607 | NA | NA | NA | NA |
| 123 | Dulaglutide_medium_dosage:Exenatide | 0 | -0.556009805 | NA | -0.556009805 | NA | NA | NA | NA |
| 124 | Dulaglutide_medium_dosage:Inject_semaglutide_high_dosage | 0 | 0.208688916 | NA | 0.208688916 | NA | NA | NA | NA |
| 125 | Dulaglutide_medium_dosage:Inject_semaglutide_low_dosage | 0 | 1.030850794 | NA | 1.030850794 | NA | NA | NA | NA |
| 126 | Dulaglutide_medium_dosage:Inject_semaglutide_medium_dosage | 0 | -0.576355981 | NA | -0.576355981 | NA | NA | NA | NA |
| 127 | Dulaglutide_medium_dosage:Liraglutide | 0 | 0.562259144 | NA | 0.562259144 | NA | NA | NA | NA |
| 128 | Dulaglutide_medium_dosage:Lixisenatide | 0 | -1.296864851 | NA | -1.296864851 | NA | NA | NA | NA |
| 129 | Dulaglutide_medium_dosage:Oral_semaglutide | 0 | -0.051602055 | NA | -0.051602055 | NA | NA | NA | NA |
| 130 | Dulaglutide_medium_dosage:Orforglipron | 4 | 0.281054683 | 0.215333363 | 0.375750831 | -0.160417467 | -2.605430468 | 2.284595534 | 0.897679414 |
| 131 | Dulaglutide_medium_dosage:Placebo_or_Control | 5 | -0.181057082 | -0.239936014 | 0.86805687 | -1.107992884 | -3.882381713 | 1.666395944 | 0.433779588 |
| 132 | Dulaglutide_medium_dosage:Sotagliflozin | 0 | 0.360717222 | NA | 0.360717222 | NA | NA | NA | NA |
| 133 | Dulaglutide_medium_dosage:Tirzepatide_high_dosage | 0 | -1.718791755 | NA | -1.718791755 | NA | NA | NA | NA |
| 134 | Dulaglutide_medium_dosage:Tirzepatide_low_dosage | 0 | -1.708346641 | NA | -1.708346641 | NA | NA | NA | NA |
| 135 | Dulaglutide_medium_dosage:Tirzepatide_medium_dosage | 0 | -1.047685395 | NA | -1.047685395 | NA | NA | NA | NA |
| 136 | Efpeglenatide_high_dosage:Efpeglenatide_low_dosage | 0 | 0.72158384 | NA | 0.72158384 | NA | NA | NA | NA |
| 137 | Efpeglenatide_high_dosage:Efpeglenatide_medium_dosage | 2 | -0.313176977 | 0.030078727 | -2.07346037 | 2.103539097 | -3.524238856 | 7.73131705 | 0.463807771 |
| 138 | Efpeglenatide_high_dosage:Empagliflozin_high_dosage | 0 | -0.703236581 | NA | -0.703236581 | NA | NA | NA | NA |
| 139 | Efpeglenatide_high_dosage:Empagliflozin_low_dosage | 0 | -0.670015907 | NA | -0.670015907 | NA | NA | NA | NA |
| 140 | Efpeglenatide_high_dosage:Ertugliflozin_high_dosage | 0 | 0.604223167 | NA | 0.604223167 | NA | NA | NA | NA |
| 141 | Efpeglenatide_high_dosage:Ertugliflozin_low_dosage | 0 | -0.534910319 | NA | -0.534910319 | NA | NA | NA | NA |
| 142 | Efpeglenatide_high_dosage:Exenatide | 0 | -0.450726517 | NA | -0.450726517 | NA | NA | NA | NA |
| 143 | Efpeglenatide_high_dosage:Inject_semaglutide_high_dosage | 0 | 0.313972204 | NA | 0.313972204 | NA | NA | NA | NA |
| 144 | Efpeglenatide_high_dosage:Inject_semaglutide_low_dosage | 0 | 1.136134082 | NA | 1.136134082 | NA | NA | NA | NA |
| 145 | Efpeglenatide_high_dosage:Inject_semaglutide_medium_dosage | 0 | -0.471072693 | NA | -0.471072693 | NA | NA | NA | NA |
| 146 | Efpeglenatide_high_dosage:Liraglutide | 0 | 0.667542432 | NA | 0.667542432 | NA | NA | NA | NA |
| 147 | Efpeglenatide_high_dosage:Lixisenatide | 0 | -1.191581563 | NA | -1.191581563 | NA | NA | NA | NA |
| 148 | Efpeglenatide_high_dosage:Oral_semaglutide | 0 | 0.053681233 | NA | 0.053681233 | NA | NA | NA | NA |
| 149 | Efpeglenatide_high_dosage:Orforglipron | 0 | 0.386337971 | NA | 0.386337971 | NA | NA | NA | NA |
| 150 | Efpeglenatide_high_dosage:Placebo_or_Control | 1 | -0.075773794 | -0.142304031 | 0.193646024 | -0.335950054 | -5.430088643 | 4.758188534 | 0.897154771 |
| 151 | Efpeglenatide_high_dosage:Sotagliflozin | 0 | 0.46600051 | NA | 0.46600051 | NA | NA | NA | NA |
| 152 | Efpeglenatide_high_dosage:Tirzepatide_high_dosage | 0 | -1.613508466 | NA | -1.613508466 | NA | NA | NA | NA |
| 153 | Efpeglenatide_high_dosage:Tirzepatide_low_dosage | 0 | -1.603063353 | NA | -1.603063353 | NA | NA | NA | NA |
| 154 | Efpeglenatide_high_dosage:Tirzepatide_medium_dosage | 0 | -0.942402107 | NA | -0.942402107 | NA | NA | NA | NA |
| 155 | Efpeglenatide_low_dosage:Efpeglenatide_medium_dosage | 1 | -1.034760817 | -1.034760817 | NA | NA | NA | NA | NA |
| 156 | Efpeglenatide_low_dosage:Empagliflozin_high_dosage | 0 | -1.424820421 | NA | -1.424820421 | NA | NA | NA | NA |
| 157 | Efpeglenatide_low_dosage:Empagliflozin_low_dosage | 0 | -1.391599747 | NA | -1.391599747 | NA | NA | NA | NA |
| 158 | Efpeglenatide_low_dosage:Ertugliflozin_high_dosage | 0 | -0.117360672 | NA | -0.117360672 | NA | NA | NA | NA |
| 159 | Efpeglenatide_low_dosage:Ertugliflozin_low_dosage | 0 | -1.256494159 | NA | -1.256494159 | NA | NA | NA | NA |
| 160 | Efpeglenatide_low_dosage:Exenatide | 0 | -1.172310357 | NA | -1.172310357 | NA | NA | NA | NA |
| 161 | Efpeglenatide_low_dosage:Inject_semaglutide_high_dosage | 0 | -0.407611636 | NA | -0.407611636 | NA | NA | NA | NA |
| 162 | Efpeglenatide_low_dosage:Inject_semaglutide_low_dosage | 0 | 0.414550242 | NA | 0.414550242 | NA | NA | NA | NA |
| 163 | Efpeglenatide_low_dosage:Inject_semaglutide_medium_dosage | 0 | -1.192656533 | NA | -1.192656533 | NA | NA | NA | NA |
| 164 | Efpeglenatide_low_dosage:Liraglutide | 0 | -0.054041407 | NA | -0.054041407 | NA | NA | NA | NA |
| 165 | Efpeglenatide_low_dosage:Lixisenatide | 0 | -1.913165402 | NA | -1.913165402 | NA | NA | NA | NA |
| 166 | Efpeglenatide_low_dosage:Oral_semaglutide | 0 | -0.667902607 | NA | -0.667902607 | NA | NA | NA | NA |
| 167 | Efpeglenatide_low_dosage:Orforglipron | 0 | -0.335245869 | NA | -0.335245869 | NA | NA | NA | NA |
| 168 | Efpeglenatide_low_dosage:Placebo_or_Control | 0 | -0.797357634 | NA | -0.797357634 | NA | NA | NA | NA |
| 169 | Efpeglenatide_low_dosage:Sotagliflozin | 0 | -0.255583329 | NA | -0.255583329 | NA | NA | NA | NA |
| 170 | Efpeglenatide_low_dosage:Tirzepatide_high_dosage | 0 | -2.335092306 | NA | -2.335092306 | NA | NA | NA | NA |
| 171 | Efpeglenatide_low_dosage:Tirzepatide_low_dosage | 0 | -2.324647192 | NA | -2.324647192 | NA | NA | NA | NA |
| 172 | Efpeglenatide_low_dosage:Tirzepatide_medium_dosage | 0 | -1.663985947 | NA | -1.663985947 | NA | NA | NA | NA |
| 173 | Efpeglenatide_medium_dosage:Empagliflozin_high_dosage | 0 | -0.390059604 | NA | -0.390059604 | NA | NA | NA | NA |
| 174 | Efpeglenatide_medium_dosage:Empagliflozin_low_dosage | 0 | -0.35683893 | NA | -0.35683893 | NA | NA | NA | NA |
| 175 | Efpeglenatide_medium_dosage:Ertugliflozin_high_dosage | 0 | 0.917400144 | NA | 0.917400144 | NA | NA | NA | NA |
| 176 | Efpeglenatide_medium_dosage:Ertugliflozin_low_dosage | 0 | -0.221733342 | NA | -0.221733342 | NA | NA | NA | NA |
| 177 | Efpeglenatide_medium_dosage:Exenatide | 0 | -0.13754954 | NA | -0.13754954 | NA | NA | NA | NA |
| 178 | Efpeglenatide_medium_dosage:Inject_semaglutide_high_dosage | 0 | 0.627149181 | NA | 0.627149181 | NA | NA | NA | NA |
| 179 | Efpeglenatide_medium_dosage:Inject_semaglutide_low_dosage | 0 | 1.449311059 | NA | 1.449311059 | NA | NA | NA | NA |
| 180 | Efpeglenatide_medium_dosage:Inject_semaglutide_medium_dosage | 0 | -0.157895716 | NA | -0.157895716 | NA | NA | NA | NA |
| 181 | Efpeglenatide_medium_dosage:Liraglutide | 0 | 0.980719409 | NA | 0.980719409 | NA | NA | NA | NA |
| 182 | Efpeglenatide_medium_dosage:Lixisenatide | 0 | -0.878404586 | NA | -0.878404586 | NA | NA | NA | NA |
| 183 | Efpeglenatide_medium_dosage:Oral_semaglutide | 0 | 0.36685821 | NA | 0.36685821 | NA | NA | NA | NA |
| 184 | Efpeglenatide_medium_dosage:Orforglipron | 0 | 0.699514948 | NA | 0.699514948 | NA | NA | NA | NA |
| 185 | Efpeglenatide_medium_dosage:Placebo_or_Control | 2 | 0.237403183 | -0.005482421 | 1.485406878 | -1.4908893 | -7.12132056 | 4.13954196 | 0.603773683 |
| 186 | Efpeglenatide_medium_dosage:Sotagliflozin | 0 | 0.779177487 | NA | 0.779177487 | NA | NA | NA | NA |
| 187 | Efpeglenatide_medium_dosage:Tirzepatide_high_dosage | 0 | -1.300331489 | NA | -1.300331489 | NA | NA | NA | NA |
| 188 | Efpeglenatide_medium_dosage:Tirzepatide_low_dosage | 0 | -1.289886376 | NA | -1.289886376 | NA | NA | NA | NA |
| 189 | Efpeglenatide_medium_dosage:Tirzepatide_medium_dosage | 0 | -0.62922513 | NA | -0.62922513 | NA | NA | NA | NA |
| 190 | Empagliflozin_high_dosage:Empagliflozin_low_dosage | 2 | 0.033220674 | 0.516278344 | -1.546748431 | 2.063026775 | -0.93464144 | 5.060694989 | 0.177379427 |
| 191 | Empagliflozin_high_dosage:Ertugliflozin_high_dosage | 0 | 1.307459748 | NA | 1.307459748 | NA | NA | NA | NA |
| 192 | Empagliflozin_high_dosage:Ertugliflozin_low_dosage | 0 | 0.168326262 | NA | 0.168326262 | NA | NA | NA | NA |
| 193 | Empagliflozin_high_dosage:Exenatide | 0 | 0.252510064 | NA | 0.252510064 | NA | NA | NA | NA |
| 194 | Empagliflozin_high_dosage:Inject_semaglutide_high_dosage | 0 | 1.017208785 | NA | 1.017208785 | NA | NA | NA | NA |
| 195 | Empagliflozin_high_dosage:Inject_semaglutide_low_dosage | 0 | 1.839370663 | NA | 1.839370663 | NA | NA | NA | NA |
| 196 | Empagliflozin_high_dosage:Inject_semaglutide_medium_dosage | 0 | 0.232163888 | NA | 0.232163888 | NA | NA | NA | NA |
| 197 | Empagliflozin_high_dosage:Liraglutide | 0 | 1.370779013 | NA | 1.370779013 | NA | NA | NA | NA |
| 198 | Empagliflozin_high_dosage:Lixisenatide | 0 | -0.488344981 | NA | -0.488344981 | NA | NA | NA | NA |
| 199 | Empagliflozin_high_dosage:Oral_semaglutide | 0 | 0.756917814 | NA | 0.756917814 | NA | NA | NA | NA |
| 200 | Empagliflozin_high_dosage:Orforglipron | 0 | 1.089574552 | NA | 1.089574552 | NA | NA | NA | NA |
| 201 | Empagliflozin_high_dosage:Placebo_or_Control | 3 | 0.627462787 | 0.22843374 | 1.71833092 | -1.48989718 | -4.524929301 | 1.545134941 | 0.335976138 |
| 202 | Empagliflozin_high_dosage:Sotagliflozin | 0 | 1.169237092 | NA | 1.169237092 | NA | NA | NA | NA |
| 203 | Empagliflozin_high_dosage:Tirzepatide_high_dosage | 0 | -0.910271885 | NA | -0.910271885 | NA | NA | NA | NA |
| 204 | Empagliflozin_high_dosage:Tirzepatide_low_dosage | 0 | -0.899826771 | NA | -0.899826771 | NA | NA | NA | NA |
| 205 | Empagliflozin_high_dosage:Tirzepatide_medium_dosage | 0 | -0.239165526 | NA | -0.239165526 | NA | NA | NA | NA |
| 206 | Empagliflozin_low_dosage:Ertugliflozin_high_dosage | 0 | 1.274239074 | NA | 1.274239074 | NA | NA | NA | NA |
| 207 | Empagliflozin_low_dosage:Ertugliflozin_low_dosage | 0 | 0.135105588 | NA | 0.135105588 | NA | NA | NA | NA |
| 208 | Empagliflozin_low_dosage:Exenatide | 0 | 0.21928939 | NA | 0.21928939 | NA | NA | NA | NA |
| 209 | Empagliflozin_low_dosage:Inject_semaglutide_high_dosage | 0 | 0.983988111 | NA | 0.983988111 | NA | NA | NA | NA |
| 210 | Empagliflozin_low_dosage:Inject_semaglutide_low_dosage | 0 | 1.806149989 | NA | 1.806149989 | NA | NA | NA | NA |
| 211 | Empagliflozin_low_dosage:Inject_semaglutide_medium_dosage | 0 | 0.198943214 | NA | 0.198943214 | NA | NA | NA | NA |
| 212 | Empagliflozin_low_dosage:Liraglutide | 0 | 1.337558339 | NA | 1.337558339 | NA | NA | NA | NA |
| 213 | Empagliflozin_low_dosage:Lixisenatide | 0 | -0.521565656 | NA | -0.521565656 | NA | NA | NA | NA |
| 214 | Empagliflozin_low_dosage:Oral_semaglutide | 0 | 0.72369714 | NA | 0.72369714 | NA | NA | NA | NA |
| 215 | Empagliflozin_low_dosage:Orforglipron | 0 | 1.056353878 | NA | 1.056353878 | NA | NA | NA | NA |
| 216 | Empagliflozin_low_dosage:Placebo_or_Control | 3 | 0.594242113 | 1.290091572 | -1.351666324 | 2.641757896 | -0.456479802 | 5.739995594 | 0.094683742 |
| 217 | Empagliflozin_low_dosage:Sotagliflozin | 0 | 1.136016417 | NA | 1.136016417 | NA | NA | NA | NA |
| 218 | Empagliflozin_low_dosage:Tirzepatide_high_dosage | 0 | -0.943492559 | NA | -0.943492559 | NA | NA | NA | NA |
| 219 | Empagliflozin_low_dosage:Tirzepatide_low_dosage | 0 | -0.933047446 | NA | -0.933047446 | NA | NA | NA | NA |
| 220 | Empagliflozin_low_dosage:Tirzepatide_medium_dosage | 0 | -0.2723862 | NA | -0.2723862 | NA | NA | NA | NA |
| 221 | Ertugliflozin_high_dosage:Ertugliflozin_low_dosage | 1 | -1.139133486 | -1.139133486 | NA | NA | NA | NA | NA |
| 222 | Ertugliflozin_high_dosage:Exenatide | 0 | -1.054949684 | NA | -1.054949684 | NA | NA | NA | NA |
| 223 | Ertugliflozin_high_dosage:Inject_semaglutide_high_dosage | 0 | -0.290250964 | NA | -0.290250964 | NA | NA | NA | NA |
| 224 | Ertugliflozin_high_dosage:Inject_semaglutide_low_dosage | 0 | 0.531910914 | NA | 0.531910914 | NA | NA | NA | NA |
| 225 | Ertugliflozin_high_dosage:Inject_semaglutide_medium_dosage | 0 | -1.07529586 | NA | -1.07529586 | NA | NA | NA | NA |
| 226 | Ertugliflozin_high_dosage:Liraglutide | 0 | 0.063319265 | NA | 0.063319265 | NA | NA | NA | NA |
| 227 | Ertugliflozin_high_dosage:Lixisenatide | 0 | -1.79580473 | NA | -1.79580473 | NA | NA | NA | NA |
| 228 | Ertugliflozin_high_dosage:Oral_semaglutide | 0 | -0.550541934 | NA | -0.550541934 | NA | NA | NA | NA |
| 229 | Ertugliflozin_high_dosage:Orforglipron | 0 | -0.217885196 | NA | -0.217885196 | NA | NA | NA | NA |
| 230 | Ertugliflozin_high_dosage:Placebo_or_Control | 1 | -0.679996961 | -0.679996961 | NA | NA | NA | NA | NA |
| 231 | Ertugliflozin_high_dosage:Sotagliflozin | 0 | -0.138222657 | NA | -0.138222657 | NA | NA | NA | NA |
| 232 | Ertugliflozin_high_dosage:Tirzepatide_high_dosage | 0 | -2.217731634 | NA | -2.217731634 | NA | NA | NA | NA |
| 233 | Ertugliflozin_high_dosage:Tirzepatide_low_dosage | 0 | -2.20728652 | NA | -2.20728652 | NA | NA | NA | NA |
| 234 | Ertugliflozin_high_dosage:Tirzepatide_medium_dosage | 0 | -1.546625275 | NA | -1.546625275 | NA | NA | NA | NA |
| 235 | Ertugliflozin_low_dosage:Exenatide | 0 | 0.084183802 | NA | 0.084183802 | NA | NA | NA | NA |
| 236 | Ertugliflozin_low_dosage:Inject_semaglutide_high_dosage | 0 | 0.848882523 | NA | 0.848882523 | NA | NA | NA | NA |
| 237 | Ertugliflozin_low_dosage:Inject_semaglutide_low_dosage | 0 | 1.6710444 | NA | 1.6710444 | NA | NA | NA | NA |
| 238 | Ertugliflozin_low_dosage:Inject_semaglutide_medium_dosage | 0 | 0.063837626 | NA | 0.063837626 | NA | NA | NA | NA |
| 239 | Ertugliflozin_low_dosage:Liraglutide | 0 | 1.202452751 | NA | 1.202452751 | NA | NA | NA | NA |
| 240 | Ertugliflozin_low_dosage:Lixisenatide | 0 | -0.656671244 | NA | -0.656671244 | NA | NA | NA | NA |
| 241 | Ertugliflozin_low_dosage:Oral_semaglutide | 0 | 0.588591552 | NA | 0.588591552 | NA | NA | NA | NA |
| 242 | Ertugliflozin_low_dosage:Orforglipron | 0 | 0.92124829 | NA | 0.92124829 | NA | NA | NA | NA |
| 243 | Ertugliflozin_low_dosage:Placebo_or_Control | 1 | 0.459136525 | 0.459136525 | NA | NA | NA | NA | NA |
| 244 | Ertugliflozin_low_dosage:Sotagliflozin | 0 | 1.000910829 | NA | 1.000910829 | NA | NA | NA | NA |
| 245 | Ertugliflozin_low_dosage:Tirzepatide_high_dosage | 0 | -1.078598148 | NA | -1.078598148 | NA | NA | NA | NA |
| 246 | Ertugliflozin_low_dosage:Tirzepatide_low_dosage | 0 | -1.068153034 | NA | -1.068153034 | NA | NA | NA | NA |
| 247 | Ertugliflozin_low_dosage:Tirzepatide_medium_dosage | 0 | -0.407491788 | NA | -0.407491788 | NA | NA | NA | NA |
| 248 | Exenatide:Inject_semaglutide_high_dosage | 0 | 0.764698721 | NA | 0.764698721 | NA | NA | NA | NA |
| 249 | Exenatide:Inject_semaglutide_low_dosage | 0 | 1.586860599 | NA | 1.586860599 | NA | NA | NA | NA |
| 250 | Exenatide:Inject_semaglutide_medium_dosage | 1 | -0.020346176 | 0.044451763 | -0.034294656 | 0.078746418 | -2.985343645 | 3.142836482 | 0.959827041 |
| 251 | Exenatide:Liraglutide | 0 | 1.118268949 | NA | 1.118268949 | NA | NA | NA | NA |
| 252 | Exenatide:Lixisenatide | 0 | -0.740855045 | NA | -0.740855045 | NA | NA | NA | NA |
| 253 | Exenatide:Oral_semaglutide | 0 | 0.50440775 | NA | 0.50440775 | NA | NA | NA | NA |
| 254 | Exenatide:Orforglipron | 0 | 0.837064488 | NA | 0.837064488 | NA | NA | NA | NA |
| 255 | Exenatide:Placebo_or_Control | 2 | 0.374952723 | 0.370207204 | 0.448953623 | -0.078746418 | -3.142836482 | 2.985343645 | 0.959827041 |
| 256 | Exenatide:Sotagliflozin | 0 | 0.916727028 | NA | 0.916727028 | NA | NA | NA | NA |
| 257 | Exenatide:Tirzepatide_high_dosage | 0 | -1.162781949 | NA | -1.162781949 | NA | NA | NA | NA |
| 258 | Exenatide:Tirzepatide_low_dosage | 0 | -1.152336835 | NA | -1.152336835 | NA | NA | NA | NA |
| 259 | Exenatide:Tirzepatide_medium_dosage | 0 | -0.49167559 | NA | -0.49167559 | NA | NA | NA | NA |
| 260 | Inject_semaglutide_high_dosage:Inject_semaglutide_low_dosage | 0 | 0.822161878 | NA | 0.822161878 | NA | NA | NA | NA |
| 261 | Inject_semaglutide_high_dosage:Inject_semaglutide_medium_dosage | 1 | -0.785044897 | -1.197283816 | -0.711535106 | -0.48574871 | -3.966146671 | 2.994649251 | 0.784433308 |
| 262 | Inject_semaglutide_high_dosage:Liraglutide | 0 | 0.353570229 | NA | 0.353570229 | NA | NA | NA | NA |
| 263 | Inject_semaglutide_high_dosage:Lixisenatide | 0 | -1.505553766 | NA | -1.505553766 | NA | NA | NA | NA |
| 264 | Inject_semaglutide_high_dosage:Oral_semaglutide | 0 | -0.260290971 | NA | -0.260290971 | NA | NA | NA | NA |
| 265 | Inject_semaglutide_high_dosage:Orforglipron | 0 | 0.072365767 | NA | 0.072365767 | NA | NA | NA | NA |
| 266 | Inject_semaglutide_high_dosage:Placebo_or_Control | 5 | -0.389745998 | -0.358828796 | -0.844577505 | 0.48574871 | -2.994649251 | 3.966146671 | 0.784433308 |
| 267 | Inject_semaglutide_high_dosage:Sotagliflozin | 0 | 0.152028307 | NA | 0.152028307 | NA | NA | NA | NA |
| 268 | Inject_semaglutide_high_dosage:Tirzepatide_high_dosage | 0 | -1.92748067 | NA | -1.92748067 | NA | NA | NA | NA |
| 269 | Inject_semaglutide_high_dosage:Tirzepatide_low_dosage | 0 | -1.917035556 | NA | -1.917035556 | NA | NA | NA | NA |
| 270 | Inject_semaglutide_high_dosage:Tirzepatide_medium_dosage | 0 | -1.256374311 | NA | -1.256374311 | NA | NA | NA | NA |
| 271 | Inject_semaglutide_low_dosage:Inject_semaglutide_medium_dosage | 1 | -1.607206775 | -1.700984445 | -1.487694536 | -0.213289908 | -4.79851258 | 4.371932763 | 0.927356509 |
| 272 | Inject_semaglutide_low_dosage:Liraglutide | 0 | -0.468591649 | NA | -0.468591649 | NA | NA | NA | NA |
| 273 | Inject_semaglutide_low_dosage:Lixisenatide | 0 | -2.327715644 | NA | -2.327715644 | NA | NA | NA | NA |
| 274 | Inject_semaglutide_low_dosage:Oral_semaglutide | 0 | -1.082452849 | NA | -1.082452849 | NA | NA | NA | NA |
| 275 | Inject_semaglutide_low_dosage:Orforglipron | 0 | -0.749796111 | NA | -0.749796111 | NA | NA | NA | NA |
| 276 | Inject_semaglutide_low_dosage:Placebo_or_Control | 2 | -1.211907876 | -1.199903246 | -1.865943006 | 0.66603976 | -15.55267129 | 16.88475081 | 0.935849026 |
| 277 | Inject_semaglutide_low_dosage:Sotagliflozin | 0 | -0.670133571 | NA | -0.670133571 | NA | NA | NA | NA |
| 278 | Inject_semaglutide_low_dosage:Tirzepatide_high_dosage | 0 | -2.749642548 | NA | -2.749642548 | NA | NA | NA | NA |
| 279 | Inject_semaglutide_low_dosage:Tirzepatide_low_dosage | 0 | -2.739197434 | NA | -2.739197434 | NA | NA | NA | NA |
| 280 | Inject_semaglutide_low_dosage:Tirzepatide_medium_dosage | 0 | -2.078536189 | NA | -2.078536189 | NA | NA | NA | NA |
| 281 | Inject_semaglutide_medium_dosage:Liraglutide | 0 | 1.138615125 | NA | 1.138615125 | NA | NA | NA | NA |
| 282 | Inject_semaglutide_medium_dosage:Lixisenatide | 0 | -0.720508869 | NA | -0.720508869 | NA | NA | NA | NA |
| 283 | Inject_semaglutide_medium_dosage:Oral_semaglutide | 0 | 0.524753926 | NA | 0.524753926 | NA | NA | NA | NA |
| 284 | Inject_semaglutide_medium_dosage:Orforglipron | 0 | 0.857410664 | NA | 0.857410664 | NA | NA | NA | NA |
| 285 | Inject_semaglutide_medium_dosage:Placebo_or_Control | 4 | 0.395298899 | 0.395128321 | 0.395613184 | -0.000484863 | -2.061865771 | 2.060896045 | 0.999632168 |
| 286 | Inject_semaglutide_medium_dosage:Sotagliflozin | 0 | 0.937073204 | NA | 0.937073204 | NA | NA | NA | NA |
| 287 | Inject_semaglutide_medium_dosage:Tirzepatide_high_dosage | 1 | -1.142435773 | -1.054605188 | -1.164994349 | 0.110389161 | -3.483159791 | 3.703938112 | 0.951990332 |
| 288 | Inject_semaglutide_medium_dosage:Tirzepatide_low_dosage | 1 | -1.131990659 | -1.534599853 | -0.985292242 | -0.549307611 | -4.101789971 | 3.003174749 | 0.761842457 |
| 289 | Inject_semaglutide_medium_dosage:Tirzepatide_medium_dosage | 0 | -0.471329414 | NA | -0.471329414 | NA | NA | NA | NA |
| 290 | Liraglutide:Lixisenatide | 0 | -1.859123995 | NA | -1.859123995 | NA | NA | NA | NA |
| 291 | Liraglutide:Oral_semaglutide | 0 | -0.613861199 | NA | -0.613861199 | NA | NA | NA | NA |
| 292 | Liraglutide:Orforglipron | 0 | -0.281204461 | NA | -0.281204461 | NA | NA | NA | NA |
| 293 | Liraglutide:Placebo_or_Control | 5 | -0.743316226 | -0.743316226 | NA | NA | NA | NA | NA |
| 294 | Liraglutide:Sotagliflozin | 0 | -0.201541922 | NA | -0.201541922 | NA | NA | NA | NA |
| 295 | Liraglutide:Tirzepatide_high_dosage | 0 | -2.281050899 | NA | -2.281050899 | NA | NA | NA | NA |
| 296 | Liraglutide:Tirzepatide_low_dosage | 0 | -2.270605785 | NA | -2.270605785 | NA | NA | NA | NA |
| 297 | Liraglutide:Tirzepatide_medium_dosage | 0 | -1.609944539 | NA | -1.609944539 | NA | NA | NA | NA |
| 298 | Lixisenatide:Oral_semaglutide | 0 | 1.245262796 | NA | 1.245262796 | NA | NA | NA | NA |
| 299 | Lixisenatide:Orforglipron | 0 | 1.577919534 | NA | 1.577919534 | NA | NA | NA | NA |
| 300 | Lixisenatide:Placebo_or_Control | 1 | 1.115807769 | 1.115807769 | NA | NA | NA | NA | NA |
| 301 | Lixisenatide:Sotagliflozin | 0 | 1.657582073 | NA | 1.657582073 | NA | NA | NA | NA |
| 302 | Lixisenatide:Tirzepatide_high_dosage | 0 | -0.421926904 | NA | -0.421926904 | NA | NA | NA | NA |
| 303 | Lixisenatide:Tirzepatide_low_dosage | 0 | -0.41148179 | NA | -0.41148179 | NA | NA | NA | NA |
| 304 | Lixisenatide:Tirzepatide_medium_dosage | 0 | 0.249179455 | NA | 0.249179455 | NA | NA | NA | NA |
| 305 | Oral_semaglutide:Orforglipron | 0 | 0.332656738 | NA | 0.332656738 | NA | NA | NA | NA |
| 306 | Oral_semaglutide:Placebo_or_Control | 3 | -0.129455027 | -0.129455027 | NA | NA | NA | NA | NA |
| 307 | Oral_semaglutide:Sotagliflozin | 0 | 0.412319277 | NA | 0.412319277 | NA | NA | NA | NA |
| 308 | Oral_semaglutide:Tirzepatide_high_dosage | 0 | -1.6671897 | NA | -1.6671897 | NA | NA | NA | NA |
| 309 | Oral_semaglutide:Tirzepatide_low_dosage | 0 | -1.656744586 | NA | -1.656744586 | NA | NA | NA | NA |
| 310 | Oral_semaglutide:Tirzepatide_medium_dosage | 0 | -0.99608334 | NA | -0.99608334 | NA | NA | NA | NA |
| 311 | Orforglipron:Placebo_or_Control | 2 | -0.462111765 | -0.258702947 | -0.760345499 | 0.501642552 | -1.935706351 | 2.938991454 | 0.686661596 |
| 312 | Orforglipron:Sotagliflozin | 0 | 0.079662539 | NA | 0.079662539 | NA | NA | NA | NA |
| 313 | Orforglipron:Tirzepatide_high_dosage | 0 | -1.999846437 | NA | -1.999846437 | NA | NA | NA | NA |
| 314 | Orforglipron:Tirzepatide_low_dosage | 0 | -1.989401324 | NA | -1.989401324 | NA | NA | NA | NA |
| 315 | Orforglipron:Tirzepatide_medium_dosage | 0 | -1.328740078 | NA | -1.328740078 | NA | NA | NA | NA |
| 316 | Sotagliflozin:Placebo_or_Control | 3 | -0.541774304 | -0.541774304 | NA | NA | NA | NA | NA |
| 317 | Tirzepatide_high_dosage:Placebo_or_Control | 3 | 1.537734673 | 1.428742408 | 1.650948346 | -0.222205938 | -2.806473052 | 2.362061177 | 0.866169663 |
| 318 | Tirzepatide_low_dosage:Placebo_or_Control | 1 | 1.527289559 | 1.124135716 | 1.633626132 | -0.509490416 | -4.110652461 | 3.091671629 | 0.781553889 |
| 319 | Tirzepatide_medium_dosage:Placebo_or_Control | 1 | 0.866628313 | 1.257360678 | 0.758713358 | 0.498647319 | -3.143947089 | 4.141241727 | 0.788463608 |
| 320 | Sotagliflozin:Tirzepatide_high_dosage | 0 | -2.079508977 | NA | -2.079508977 | NA | NA | NA | NA |
| 321 | Sotagliflozin:Tirzepatide_low_dosage | 0 | -2.069063863 | NA | -2.069063863 | NA | NA | NA | NA |
| 322 | Sotagliflozin:Tirzepatide_medium_dosage | 0 | -1.408402618 | NA | -1.408402618 | NA | NA | NA | NA |
| 323 | Tirzepatide_high_dosage:Tirzepatide_low_dosage | 3 | 0.010445114 | -0.11593316 | 0.16071897 | -0.276652129 | -2.621639659 | 2.068335401 | 0.817137204 |
| 324 | Tirzepatide_high_dosage:Tirzepatide_medium_dosage | 6 | 0.671106359 | 0.826068897 | 0.311275316 | 0.514793581 | -1.849031646 | 2.878618808 | 0.66949534 |
| 325 | Tirzepatide_low_dosage:Tirzepatide_medium_dosage | 3 | 0.660661245 | 0.479880751 | 0.83438069 | -0.354499939 | -2.903763747 | 2.194763869 | 0.785197564 |

**eTable 7C: Side-splitting model inconsistency of primary outcome: subgroup of cervical tumor**

|  | Comparison | No.Studies | NMA | Direct | Indirect | Difference | Diff_95CI_lower | Diff_95CI_upper | *p* value |
| --- | --- | --- | --- | --- | --- | --- | --- | --- | --- |
| 1 | Bexagliflozin:Canagliflozin_high_dosage | 0 | -2.821211443 | NA | -2.821211443 | NA | NA | NA | NA |
| 2 | Bexagliflozin:Dapagliflozin_high_dosage | 0 | -0.478815304 | NA | -0.478815304 | NA | NA | NA | NA |
| 3 | Bexagliflozin:Dulaglutide_medium_dosage | 0 | -1.93134939 | NA | -1.93134939 | NA | NA | NA | NA |
| 4 | Bexagliflozin:Empagliflozin_high_dosage | 0 | -3.274778379 | NA | -3.274778379 | NA | NA | NA | NA |
| 5 | Bexagliflozin:Empagliflozin_low_dosage | 0 | -2.32969919 | NA | -2.32969919 | NA | NA | NA | NA |
| 6 | Bexagliflozin:Ertugliflozin_high_dosage | 0 | -2.977187008 | NA | -2.977187008 | NA | NA | NA | NA |
| 7 | Bexagliflozin:Ertugliflozin_low_dosage | 0 | -2.941167373 | NA | -2.941167373 | NA | NA | NA | NA |
| 8 | Bexagliflozin:Exenatide | 0 | -1.763995291 | NA | -1.763995291 | NA | NA | NA | NA |
| 9 | Bexagliflozin:Inject_semaglutide_high_dosage | 0 | -1.118231499 | NA | -1.118231499 | NA | NA | NA | NA |
| 10 | Bexagliflozin:Liraglutide | 0 | -2.744659096 | NA | -2.744659096 | NA | NA | NA | NA |
| 11 | Bexagliflozin:Placebo_or_Control | 1 | -1.758639091 | -1.758639091 | NA | NA | NA | NA | NA |
| 12 | Bexagliflozin:Sotagliflozin | 0 | -0.68484928 | NA | -0.68484928 | NA | NA | NA | NA |
| 13 | Bexagliflozin:Tirzepatide_high_dosage | 0 | -1.784238898 | NA | -1.784238898 | NA | NA | NA | NA |
| 14 | Bexagliflozin:Tirzepatide_low_dosage | 0 | -0.68091774 | NA | -0.68091774 | NA | NA | NA | NA |
| 15 | Bexagliflozin:Tirzepatide_medium_dosage | 0 | -0.678575818 | NA | -0.678575818 | NA | NA | NA | NA |
| 16 | Canagliflozin_high_dosage:Dapagliflozin_high_dosage | 0 | 2.342396139 | NA | 2.342396139 | NA | NA | NA | NA |
| 17 | Canagliflozin_high_dosage:Dulaglutide_medium_dosage | 0 | 0.889862053 | NA | 0.889862053 | NA | NA | NA | NA |
| 18 | Canagliflozin_high_dosage:Empagliflozin_high_dosage | 0 | -0.453566935 | NA | -0.453566935 | NA | NA | NA | NA |
| 19 | Canagliflozin_high_dosage:Empagliflozin_low_dosage | 0 | 0.491512253 | NA | 0.491512253 | NA | NA | NA | NA |
| 20 | Canagliflozin_high_dosage:Ertugliflozin_high_dosage | 0 | -0.155975564 | NA | -0.155975564 | NA | NA | NA | NA |
| 21 | Canagliflozin_high_dosage:Ertugliflozin_low_dosage | 0 | -0.119955929 | NA | -0.119955929 | NA | NA | NA | NA |
| 22 | Canagliflozin_high_dosage:Exenatide | 0 | 1.057216153 | NA | 1.057216153 | NA | NA | NA | NA |
| 23 | Canagliflozin_high_dosage:Inject_semaglutide_high_dosage | 0 | 1.702979945 | NA | 1.702979945 | NA | NA | NA | NA |
| 24 | Canagliflozin_high_dosage:Liraglutide | 0 | 0.076552347 | NA | 0.076552347 | NA | NA | NA | NA |
| 25 | Canagliflozin_high_dosage:Placebo_or_Control | 1 | 1.062572352 | 1.062572352 | NA | NA | NA | NA | NA |
| 26 | Canagliflozin_high_dosage:Sotagliflozin | 0 | 2.136362163 | NA | 2.136362163 | NA | NA | NA | NA |
| 27 | Canagliflozin_high_dosage:Tirzepatide_high_dosage | 0 | 1.036972546 | NA | 1.036972546 | NA | NA | NA | NA |
| 28 | Canagliflozin_high_dosage:Tirzepatide_low_dosage | 0 | 2.140293704 | NA | 2.140293704 | NA | NA | NA | NA |
| 29 | Canagliflozin_high_dosage:Tirzepatide_medium_dosage | 0 | 2.142635625 | NA | 2.142635625 | NA | NA | NA | NA |
| 30 | Dapagliflozin_high_dosage:Dulaglutide_medium_dosage | 0 | -1.452534086 | NA | -1.452534086 | NA | NA | NA | NA |
| 31 | Dapagliflozin_high_dosage:Empagliflozin_high_dosage | 0 | -2.795963075 | NA | -2.795963075 | NA | NA | NA | NA |
| 32 | Dapagliflozin_high_dosage:Empagliflozin_low_dosage | 0 | -1.850883886 | NA | -1.850883886 | NA | NA | NA | NA |
| 33 | Dapagliflozin_high_dosage:Ertugliflozin_high_dosage | 0 | -2.498371704 | NA | -2.498371704 | NA | NA | NA | NA |
| 34 | Dapagliflozin_high_dosage:Ertugliflozin_low_dosage | 0 | -2.462352069 | NA | -2.462352069 | NA | NA | NA | NA |
| 35 | Dapagliflozin_high_dosage:Exenatide | 0 | -1.285179987 | NA | -1.285179987 | NA | NA | NA | NA |
| 36 | Dapagliflozin_high_dosage:Inject_semaglutide_high_dosage | 0 | -0.639416195 | NA | -0.639416195 | NA | NA | NA | NA |
| 37 | Dapagliflozin_high_dosage:Liraglutide | 0 | -2.265843792 | NA | -2.265843792 | NA | NA | NA | NA |
| 38 | Dapagliflozin_high_dosage:Placebo_or_Control | 3 | -1.279823787 | -1.279823787 | NA | NA | NA | NA | NA |
| 39 | Dapagliflozin_high_dosage:Sotagliflozin | 0 | -0.206033976 | NA | -0.206033976 | NA | NA | NA | NA |
| 40 | Dapagliflozin_high_dosage:Tirzepatide_high_dosage | 0 | -1.305423594 | NA | -1.305423594 | NA | NA | NA | NA |
| 41 | Dapagliflozin_high_dosage:Tirzepatide_low_dosage | 0 | -0.202102436 | NA | -0.202102436 | NA | NA | NA | NA |
| 42 | Dapagliflozin_high_dosage:Tirzepatide_medium_dosage | 0 | -0.199760514 | NA | -0.199760514 | NA | NA | NA | NA |
| 43 | Dulaglutide_medium_dosage:Empagliflozin_high_dosage | 0 | -1.343428988 | NA | -1.343428988 | NA | NA | NA | NA |
| 44 | Dulaglutide_medium_dosage:Empagliflozin_low_dosage | 0 | -0.3983498 | NA | -0.3983498 | NA | NA | NA | NA |
| 45 | Dulaglutide_medium_dosage:Ertugliflozin_high_dosage | 0 | -1.045837617 | NA | -1.045837617 | NA | NA | NA | NA |
| 46 | Dulaglutide_medium_dosage:Ertugliflozin_low_dosage | 0 | -1.009817983 | NA | -1.009817983 | NA | NA | NA | NA |
| 47 | Dulaglutide_medium_dosage:Exenatide | 0 | 0.1673541 | NA | 0.1673541 | NA | NA | NA | NA |
| 48 | Dulaglutide_medium_dosage:Inject_semaglutide_high_dosage | 0 | 0.813117891 | NA | 0.813117891 | NA | NA | NA | NA |
| 49 | Dulaglutide_medium_dosage:Liraglutide | 0 | -0.813309706 | NA | -0.813309706 | NA | NA | NA | NA |
| 50 | Dulaglutide_medium_dosage:Placebo_or_Control | 1 | 0.172710299 | 0.172710299 | NA | NA | NA | NA | NA |
| 51 | Dulaglutide_medium_dosage:Sotagliflozin | 0 | 1.24650011 | NA | 1.24650011 | NA | NA | NA | NA |
| 52 | Dulaglutide_medium_dosage:Tirzepatide_high_dosage | 0 | 0.147110492 | NA | 0.147110492 | NA | NA | NA | NA |
| 53 | Dulaglutide_medium_dosage:Tirzepatide_low_dosage | 0 | 1.25043165 | NA | 1.25043165 | NA | NA | NA | NA |
| 54 | Dulaglutide_medium_dosage:Tirzepatide_medium_dosage | 0 | 1.252773572 | NA | 1.252773572 | NA | NA | NA | NA |
| 55 | Empagliflozin_high_dosage:Empagliflozin_low_dosage | 2 | 0.945079188 | 1.232498333 | 0.085337325 | 1.147161008 | -3.387840897 | 5.682162913 | 0.620044811 |
| 56 | Empagliflozin_high_dosage:Ertugliflozin_high_dosage | 0 | 0.297591371 | NA | 0.297591371 | NA | NA | NA | NA |
| 57 | Empagliflozin_high_dosage:Ertugliflozin_low_dosage | 0 | 0.333611006 | NA | 0.333611006 | NA | NA | NA | NA |
| 58 | Empagliflozin_high_dosage:Exenatide | 0 | 1.510783088 | NA | 1.510783088 | NA | NA | NA | NA |
| 59 | Empagliflozin_high_dosage:Inject_semaglutide_high_dosage | 0 | 2.15654688 | NA | 2.15654688 | NA | NA | NA | NA |
| 60 | Empagliflozin_high_dosage:Liraglutide | 0 | 0.530119282 | NA | 0.530119282 | NA | NA | NA | NA |
| 61 | Empagliflozin_high_dosage:Placebo_or_Control | 2 | 1.516139287 | 1.228749939 | 2.375910947 | -1.147161008 | -5.682162913 | 3.387840897 | 0.620044811 |
| 62 | Empagliflozin_high_dosage:Sotagliflozin | 0 | 2.589929098 | NA | 2.589929098 | NA | NA | NA | NA |
| 63 | Empagliflozin_high_dosage:Tirzepatide_high_dosage | 0 | 1.490539481 | NA | 1.490539481 | NA | NA | NA | NA |
| 64 | Empagliflozin_high_dosage:Tirzepatide_low_dosage | 0 | 2.593860639 | NA | 2.593860639 | NA | NA | NA | NA |
| 65 | Empagliflozin_high_dosage:Tirzepatide_medium_dosage | 0 | 2.59620256 | NA | 2.59620256 | NA | NA | NA | NA |
| 66 | Empagliflozin_low_dosage:Ertugliflozin_high_dosage | 0 | -0.647487817 | NA | -0.647487817 | NA | NA | NA | NA |
| 67 | Empagliflozin_low_dosage:Ertugliflozin_low_dosage | 0 | -0.611468182 | NA | -0.611468182 | NA | NA | NA | NA |
| 68 | Empagliflozin_low_dosage:Exenatide | 0 | 0.5657039 | NA | 0.5657039 | NA | NA | NA | NA |
| 69 | Empagliflozin_low_dosage:Inject_semaglutide_high_dosage | 0 | 1.211467692 | NA | 1.211467692 | NA | NA | NA | NA |
| 70 | Empagliflozin_low_dosage:Liraglutide | 0 | -0.414959906 | NA | -0.414959906 | NA | NA | NA | NA |
| 71 | Empagliflozin_low_dosage:Placebo_or_Control | 1 | 0.571060099 | 1.143412613 | -0.003748394 | 1.147161008 | -3.387840897 | 5.682162913 | 0.620044811 |
| 72 | Empagliflozin_low_dosage:Sotagliflozin | 0 | 1.64484991 | NA | 1.64484991 | NA | NA | NA | NA |
| 73 | Empagliflozin_low_dosage:Tirzepatide_high_dosage | 0 | 0.545460293 | NA | 0.545460293 | NA | NA | NA | NA |
| 74 | Empagliflozin_low_dosage:Tirzepatide_low_dosage | 0 | 1.648781451 | NA | 1.648781451 | NA | NA | NA | NA |
| 75 | Empagliflozin_low_dosage:Tirzepatide_medium_dosage | 0 | 1.651123372 | NA | 1.651123372 | NA | NA | NA | NA |
| 76 | Ertugliflozin_high_dosage:Ertugliflozin_low_dosage | 2 | 0.036019635 | 0.020734551 | 0.097152071 | -0.07641752 | -5.152568869 | 4.99973383 | 0.976461243 |
| 77 | Ertugliflozin_high_dosage:Exenatide | 0 | 1.213191717 | NA | 1.213191717 | NA | NA | NA | NA |
| 78 | Ertugliflozin_high_dosage:Inject_semaglutide_high_dosage | 0 | 1.858955509 | NA | 1.858955509 | NA | NA | NA | NA |
| 79 | Ertugliflozin_high_dosage:Liraglutide | 0 | 0.232527911 | NA | 0.232527911 | NA | NA | NA | NA |
| 80 | Ertugliflozin_high_dosage:Placebo_or_Control | 1 | 1.218547916 | 1.249272564 | 1.172855044 | 0.07641752 | -4.99973383 | 5.152568869 | 0.976461243 |
| 81 | Ertugliflozin_high_dosage:Sotagliflozin | 0 | 2.292337727 | NA | 2.292337727 | NA | NA | NA | NA |
| 82 | Ertugliflozin_high_dosage:Tirzepatide_high_dosage | 0 | 1.19294811 | NA | 1.19294811 | NA | NA | NA | NA |
| 83 | Ertugliflozin_high_dosage:Tirzepatide_low_dosage | 0 | 2.296269268 | NA | 2.296269268 | NA | NA | NA | NA |
| 84 | Ertugliflozin_high_dosage:Tirzepatide_medium_dosage | 0 | 2.298611189 | NA | 2.298611189 | NA | NA | NA | NA |
| 85 | Ertugliflozin_low_dosage:Exenatide | 0 | 1.177172082 | NA | 1.177172082 | NA | NA | NA | NA |
| 86 | Ertugliflozin_low_dosage:Inject_semaglutide_high_dosage | 0 | 1.822935874 | NA | 1.822935874 | NA | NA | NA | NA |
| 87 | Ertugliflozin_low_dosage:Liraglutide | 0 | 0.196508277 | NA | 0.196508277 | NA | NA | NA | NA |
| 88 | Ertugliflozin_low_dosage:Placebo_or_Control | 1 | 1.182528282 | 1.152120492 | 1.228538012 | -0.07641752 | -5.152568869 | 4.99973383 | 0.976461243 |
| 89 | Ertugliflozin_low_dosage:Sotagliflozin | 0 | 2.256318093 | NA | 2.256318093 | NA | NA | NA | NA |
| 90 | Ertugliflozin_low_dosage:Tirzepatide_high_dosage | 0 | 1.156928475 | NA | 1.156928475 | NA | NA | NA | NA |
| 91 | Ertugliflozin_low_dosage:Tirzepatide_low_dosage | 0 | 2.260249633 | NA | 2.260249633 | NA | NA | NA | NA |
| 92 | Ertugliflozin_low_dosage:Tirzepatide_medium_dosage | 0 | 2.262591554 | NA | 2.262591554 | NA | NA | NA | NA |
| 93 | Exenatide:Inject_semaglutide_high_dosage | 0 | 0.645763792 | NA | 0.645763792 | NA | NA | NA | NA |
| 94 | Exenatide:Liraglutide | 0 | -0.980663806 | NA | -0.980663806 | NA | NA | NA | NA |
| 95 | Exenatide:Placebo_or_Control | 1 | 0.005356199 | 0.005356199 | NA | NA | NA | NA | NA |
| 96 | Exenatide:Sotagliflozin | 0 | 1.07914601 | NA | 1.07914601 | NA | NA | NA | NA |
| 97 | Exenatide:Tirzepatide_high_dosage | 0 | -0.020243607 | NA | -0.020243607 | NA | NA | NA | NA |
| 98 | Exenatide:Tirzepatide_low_dosage | 0 | 1.083077551 | NA | 1.083077551 | NA | NA | NA | NA |
| 99 | Exenatide:Tirzepatide_medium_dosage | 0 | 1.085419472 | NA | 1.085419472 | NA | NA | NA | NA |
| 100 | Inject_semaglutide_high_dosage:Liraglutide | 0 | -1.626427597 | NA | -1.626427597 | NA | NA | NA | NA |
| 101 | Inject_semaglutide_high_dosage:Placebo_or_Control | 2 | -0.640407592 | -0.640407592 | NA | NA | NA | NA | NA |
| 102 | Inject_semaglutide_high_dosage:Sotagliflozin | 0 | 0.433382219 | NA | 0.433382219 | NA | NA | NA | NA |
| 103 | Inject_semaglutide_high_dosage:Tirzepatide_high_dosage | 0 | -0.666007399 | NA | -0.666007399 | NA | NA | NA | NA |
| 104 | Inject_semaglutide_high_dosage:Tirzepatide_low_dosage | 0 | 0.437313759 | NA | 0.437313759 | NA | NA | NA | NA |
| 105 | Inject_semaglutide_high_dosage:Tirzepatide_medium_dosage | 0 | 0.43965568 | NA | 0.43965568 | NA | NA | NA | NA |
| 106 | Liraglutide:Placebo_or_Control | 2 | 0.986020005 | 0.986020005 | NA | NA | NA | NA | NA |
| 107 | Liraglutide:Sotagliflozin | 0 | 2.059809816 | NA | 2.059809816 | NA | NA | NA | NA |
| 108 | Liraglutide:Tirzepatide_high_dosage | 0 | 0.960420198 | NA | 0.960420198 | NA | NA | NA | NA |
| 109 | Liraglutide:Tirzepatide_low_dosage | 0 | 2.063741356 | NA | 2.063741356 | NA | NA | NA | NA |
| 110 | Liraglutide:Tirzepatide_medium_dosage | 0 | 2.066083278 | NA | 2.066083278 | NA | NA | NA | NA |
| 111 | Sotagliflozin:Placebo_or_Control | 1 | -1.073789811 | -1.073789811 | NA | NA | NA | NA | NA |
| 112 | Tirzepatide_high_dosage:Placebo_or_Control | 1 | 0.025599807 | 0.025612576 | 0.025582791 | 2.98E-05 | -4.238204553 | 4.238264123 | 0.99998901 |
| 113 | Tirzepatide_low_dosage:Placebo_or_Control | 1 | -1.077721351 | -1.077729859 | -1.077708201 | -2.17E-05 | -5.111124225 | 5.111080908 | 0.999993373 |
| 114 | Tirzepatide_medium_dosage:Placebo_or_Control | 1 | -1.080063273 | -1.080071781 | -1.080050122 | -2.17E-05 | -5.11112041 | 5.111077093 | 0.999993373 |
| 115 | Sotagliflozin:Tirzepatide_high_dosage | 0 | -1.099389618 | NA | -1.099389618 | NA | NA | NA | NA |
| 116 | Sotagliflozin:Tirzepatide_low_dosage | 0 | 0.00393154 | NA | 0.00393154 | NA | NA | NA | NA |
| 117 | Sotagliflozin:Tirzepatide_medium_dosage | 0 | 0.006273462 | NA | 0.006273462 | NA | NA | NA | NA |
| 118 | Tirzepatide_high_dosage:Tirzepatide_low_dosage | 1 | 1.103321158 | 1.10331265 | 1.103334308 | -2.17E-05 | -5.111124225 | 5.111080908 | 0.999993373 |
| 119 | Tirzepatide_high_dosage:Tirzepatide_medium_dosage | 1 | 1.105663079 | 1.105654571 | 1.10567623 | -2.17E-05 | -5.11112041 | 5.111077093 | 0.999993373 |
| 120 | Tirzepatide_low_dosage:Tirzepatide_medium_dosage | 0 | 0.002341921 | NA | 0.002341921 | NA | NA | NA | NA |

**eTable 7D: Side-splitting model inconsistency of primary outcome: subgroup of ovarian tumor**

|  | Comparison | No.Studies | NMA | Direct | Indirect | Difference | Diff_95CI_lower | Diff_95CI_upper | *p* value |
| --- | --- | --- | --- | --- | --- | --- | --- | --- | --- |
| 1 | Albiglutide:Bexagliflozin | 0 | 2.224057119 | NA | 2.224057119 | NA | NA | NA | NA |
| 2 | Albiglutide:Canagliflozin_high_dosage | 0 | 1.043029307 | NA | 1.043029307 | NA | NA | NA | NA |
| 3 | Albiglutide:Canagliflozin_low_dosage | 0 | 1.250837653 | NA | 1.250837653 | NA | NA | NA | NA |
| 4 | Albiglutide:Dapagliflozin_high_dosage | 0 | -0.023690736 | NA | -0.023690736 | NA | NA | NA | NA |
| 5 | Albiglutide:Dulaglutide_low_dosage | 0 | 0.121651921 | NA | 0.121651921 | NA | NA | NA | NA |
| 6 | Albiglutide:Dulaglutide_medium_dosage | 0 | 1.241439292 | NA | 1.241439292 | NA | NA | NA | NA |
| 7 | Albiglutide:Efpeglenatide_high_dosage | 0 | 1.672217827 | NA | 1.672217827 | NA | NA | NA | NA |
| 8 | Albiglutide:Efpeglenatide_low_dosage | 0 | 0.035190931 | NA | 0.035190931 | NA | NA | NA | NA |
| 9 | Albiglutide:Efpeglenatide_medium_dosage | 0 | 1.67468193 | NA | 1.67468193 | NA | NA | NA | NA |
| 10 | Albiglutide:Empagliflozin_low_dosage | 0 | 0.215255024 | NA | 0.215255024 | NA | NA | NA | NA |
| 11 | Albiglutide:Exenatide | 0 | -0.234164642 | NA | -0.234164642 | NA | NA | NA | NA |
| 12 | Albiglutide:Inject_semaglutide_high_dosage | 0 | -0.615971675 | NA | -0.615971675 | NA | NA | NA | NA |
| 13 | Albiglutide:Inject_semaglutide_low_dosage | 0 | 0.039987784 | NA | 0.039987784 | NA | NA | NA | NA |
| 14 | Albiglutide:Inject_semaglutide_medium_dosage | 0 | 1.57299402 | NA | 1.57299402 | NA | NA | NA | NA |
| 15 | Albiglutide:Liraglutide | 0 | 0.322821051 | NA | 0.322821051 | NA | NA | NA | NA |
| 16 | Albiglutide:Lixisenatide | 0 | -1.752259362 | NA | -1.752259362 | NA | NA | NA | NA |
| 17 | Albiglutide:Oral_semaglutide | 0 | 0.928068885 | NA | 0.928068885 | NA | NA | NA | NA |
| 18 | Albiglutide:Placebo_or_Control | 2 | 0.465418027 | 0.465418027 | NA | NA | NA | NA | NA |
| 19 | Albiglutide:Sotagliflozin | 0 | 1.539207838 | NA | 1.539207838 | NA | NA | NA | NA |
| 20 | Albiglutide:Tirzepatide_high_dosage | 0 | -0.074120992 | NA | -0.074120992 | NA | NA | NA | NA |
| 21 | Albiglutide:Tirzepatide_low_dosage | 0 | 0.287082402 | NA | 0.287082402 | NA | NA | NA | NA |
| 22 | Albiglutide:Tirzepatide_medium_dosage | 0 | 1.228788104 | NA | 1.228788104 | NA | NA | NA | NA |
| 23 | Bexagliflozin:Canagliflozin_high_dosage | 0 | -1.181027811 | NA | -1.181027811 | NA | NA | NA | NA |
| 24 | Bexagliflozin:Canagliflozin_low_dosage | 0 | -0.973219466 | NA | -0.973219466 | NA | NA | NA | NA |
| 25 | Bexagliflozin:Dapagliflozin_high_dosage | 0 | -2.247747855 | NA | -2.247747855 | NA | NA | NA | NA |
| 26 | Bexagliflozin:Dulaglutide_low_dosage | 0 | -2.102405197 | NA | -2.102405197 | NA | NA | NA | NA |
| 27 | Bexagliflozin:Dulaglutide_medium_dosage | 0 | -0.982617826 | NA | -0.982617826 | NA | NA | NA | NA |
| 28 | Bexagliflozin:Efpeglenatide_high_dosage | 0 | -0.551839291 | NA | -0.551839291 | NA | NA | NA | NA |
| 29 | Bexagliflozin:Efpeglenatide_low_dosage | 0 | -2.188866187 | NA | -2.188866187 | NA | NA | NA | NA |
| 30 | Bexagliflozin:Efpeglenatide_medium_dosage | 0 | -0.549375189 | NA | -0.549375189 | NA | NA | NA | NA |
| 31 | Bexagliflozin:Empagliflozin_low_dosage | 0 | -2.008802095 | NA | -2.008802095 | NA | NA | NA | NA |
| 32 | Bexagliflozin:Exenatide | 0 | -2.458221761 | NA | -2.458221761 | NA | NA | NA | NA |
| 33 | Bexagliflozin:Inject_semaglutide_high_dosage | 0 | -2.840028793 | NA | -2.840028793 | NA | NA | NA | NA |
| 34 | Bexagliflozin:Inject_semaglutide_low_dosage | 0 | -2.184069335 | NA | -2.184069335 | NA | NA | NA | NA |
| 35 | Bexagliflozin:Inject_semaglutide_medium_dosage | 0 | -0.651063099 | NA | -0.651063099 | NA | NA | NA | NA |
| 36 | Bexagliflozin:Liraglutide | 0 | -1.901236068 | NA | -1.901236068 | NA | NA | NA | NA |
| 37 | Bexagliflozin:Lixisenatide | 0 | -3.976316481 | NA | -3.976316481 | NA | NA | NA | NA |
| 38 | Bexagliflozin:Oral_semaglutide | 0 | -1.295988233 | NA | -1.295988233 | NA | NA | NA | NA |
| 39 | Bexagliflozin:Placebo_or_Control | 1 | -1.758639091 | -1.758639091 | NA | NA | NA | NA | NA |
| 40 | Bexagliflozin:Sotagliflozin | 0 | -0.68484928 | NA | -0.68484928 | NA | NA | NA | NA |
| 41 | Bexagliflozin:Tirzepatide_high_dosage | 0 | -2.298178111 | NA | -2.298178111 | NA | NA | NA | NA |
| 42 | Bexagliflozin:Tirzepatide_low_dosage | 0 | -1.936974717 | NA | -1.936974717 | NA | NA | NA | NA |
| 43 | Bexagliflozin:Tirzepatide_medium_dosage | 0 | -0.995269015 | NA | -0.995269015 | NA | NA | NA | NA |
| 44 | Canagliflozin_high_dosage:Canagliflozin_low_dosage | 1 | 0.207808346 | 1.070126131 | -1.013476325 | 2.083602456 | -2.895343179 | 7.06254809 | 0.412095856 |
| 45 | Canagliflozin_high_dosage:Dapagliflozin_high_dosage | 0 | -1.066720043 | NA | -1.066720043 | NA | NA | NA | NA |
| 46 | Canagliflozin_high_dosage:Dulaglutide_low_dosage | 0 | -0.921377386 | NA | -0.921377386 | NA | NA | NA | NA |
| 47 | Canagliflozin_high_dosage:Dulaglutide_medium_dosage | 0 | 0.198409985 | NA | 0.198409985 | NA | NA | NA | NA |
| 48 | Canagliflozin_high_dosage:Efpeglenatide_high_dosage | 0 | 0.62918852 | NA | 0.62918852 | NA | NA | NA | NA |
| 49 | Canagliflozin_high_dosage:Efpeglenatide_low_dosage | 0 | -1.007838376 | NA | -1.007838376 | NA | NA | NA | NA |
| 50 | Canagliflozin_high_dosage:Efpeglenatide_medium_dosage | 0 | 0.631652622 | NA | 0.631652622 | NA | NA | NA | NA |
| 51 | Canagliflozin_high_dosage:Empagliflozin_low_dosage | 0 | -0.827774284 | NA | -0.827774284 | NA | NA | NA | NA |
| 52 | Canagliflozin_high_dosage:Exenatide | 0 | -1.277193949 | NA | -1.277193949 | NA | NA | NA | NA |
| 53 | Canagliflozin_high_dosage:Inject_semaglutide_high_dosage | 0 | -1.659000982 | NA | -1.659000982 | NA | NA | NA | NA |
| 54 | Canagliflozin_high_dosage:Inject_semaglutide_low_dosage | 0 | -1.003041524 | NA | -1.003041524 | NA | NA | NA | NA |
| 55 | Canagliflozin_high_dosage:Inject_semaglutide_medium_dosage | 0 | 0.529964713 | NA | 0.529964713 | NA | NA | NA | NA |
| 56 | Canagliflozin_high_dosage:Liraglutide | 0 | -0.720208256 | NA | -0.720208256 | NA | NA | NA | NA |
| 57 | Canagliflozin_high_dosage:Lixisenatide | 0 | -2.795288669 | NA | -2.795288669 | NA | NA | NA | NA |
| 58 | Canagliflozin_high_dosage:Oral_semaglutide | 0 | -0.114960422 | NA | -0.114960422 | NA | NA | NA | NA |
| 59 | Canagliflozin_high_dosage:Placebo_or_Control | 2 | -0.57761128 | -0.684379221 | 3.639673 | -4.324052222 | -15.22732726 | 6.579222814 | 0.436988777 |
| 60 | Canagliflozin_high_dosage:Sotagliflozin | 0 | 0.496178531 | NA | 0.496178531 | NA | NA | NA | NA |
| 61 | Canagliflozin_high_dosage:Tirzepatide_high_dosage | 0 | -1.117150299 | NA | -1.117150299 | NA | NA | NA | NA |
| 62 | Canagliflozin_high_dosage:Tirzepatide_low_dosage | 0 | -0.755946905 | NA | -0.755946905 | NA | NA | NA | NA |
| 63 | Canagliflozin_high_dosage:Tirzepatide_medium_dosage | 0 | 0.185758797 | NA | 0.185758797 | NA | NA | NA | NA |
| 64 | Canagliflozin_low_dosage:Dapagliflozin_high_dosage | 0 | -1.274528389 | NA | -1.274528389 | NA | NA | NA | NA |
| 65 | Canagliflozin_low_dosage:Dulaglutide_low_dosage | 0 | -1.129185731 | NA | -1.129185731 | NA | NA | NA | NA |
| 66 | Canagliflozin_low_dosage:Dulaglutide_medium_dosage | 0 | -0.009398361 | NA | -0.009398361 | NA | NA | NA | NA |
| 67 | Canagliflozin_low_dosage:Efpeglenatide_high_dosage | 0 | 0.421380174 | NA | 0.421380174 | NA | NA | NA | NA |
| 68 | Canagliflozin_low_dosage:Efpeglenatide_low_dosage | 0 | -1.215646721 | NA | -1.215646721 | NA | NA | NA | NA |
| 69 | Canagliflozin_low_dosage:Efpeglenatide_medium_dosage | 0 | 0.423844277 | NA | 0.423844277 | NA | NA | NA | NA |
| 70 | Canagliflozin_low_dosage:Empagliflozin_low_dosage | 0 | -1.035582629 | NA | -1.035582629 | NA | NA | NA | NA |
| 71 | Canagliflozin_low_dosage:Exenatide | 0 | -1.485002295 | NA | -1.485002295 | NA | NA | NA | NA |
| 72 | Canagliflozin_low_dosage:Inject_semaglutide_high_dosage | 0 | -1.866809327 | NA | -1.866809327 | NA | NA | NA | NA |
| 73 | Canagliflozin_low_dosage:Inject_semaglutide_low_dosage | 0 | -1.210849869 | NA | -1.210849869 | NA | NA | NA | NA |
| 74 | Canagliflozin_low_dosage:Inject_semaglutide_medium_dosage | 0 | 0.322156367 | NA | 0.322156367 | NA | NA | NA | NA |
| 75 | Canagliflozin_low_dosage:Liraglutide | 0 | -0.928016602 | NA | -0.928016602 | NA | NA | NA | NA |
| 76 | Canagliflozin_low_dosage:Lixisenatide | 0 | -3.003097015 | NA | -3.003097015 | NA | NA | NA | NA |
| 77 | Canagliflozin_low_dosage:Oral_semaglutide | 0 | -0.322768768 | NA | -0.322768768 | NA | NA | NA | NA |
| 78 | Canagliflozin_low_dosage:Placebo_or_Control | 2 | -0.785419626 | -0.753353602 | -4.638683863 | 3.885330261 | -18.66547925 | 26.43613977 | 0.735599334 |
| 79 | Canagliflozin_low_dosage:Sotagliflozin | 0 | 0.288370185 | NA | 0.288370185 | NA | NA | NA | NA |
| 80 | Canagliflozin_low_dosage:Tirzepatide_high_dosage | 0 | -1.324958645 | NA | -1.324958645 | NA | NA | NA | NA |
| 81 | Canagliflozin_low_dosage:Tirzepatide_low_dosage | 0 | -0.963755251 | NA | -0.963755251 | NA | NA | NA | NA |
| 82 | Canagliflozin_low_dosage:Tirzepatide_medium_dosage | 0 | -0.022049549 | NA | -0.022049549 | NA | NA | NA | NA |
| 83 | Dapagliflozin_high_dosage:Dulaglutide_low_dosage | 0 | 0.145342658 | NA | 0.145342658 | NA | NA | NA | NA |
| 84 | Dapagliflozin_high_dosage:Dulaglutide_medium_dosage | 0 | 1.265130028 | NA | 1.265130028 | NA | NA | NA | NA |
| 85 | Dapagliflozin_high_dosage:Efpeglenatide_high_dosage | 0 | 1.695908564 | NA | 1.695908564 | NA | NA | NA | NA |
| 86 | Dapagliflozin_high_dosage:Efpeglenatide_low_dosage | 0 | 0.058881668 | NA | 0.058881668 | NA | NA | NA | NA |
| 87 | Dapagliflozin_high_dosage:Efpeglenatide_medium_dosage | 0 | 1.698372666 | NA | 1.698372666 | NA | NA | NA | NA |
| 88 | Dapagliflozin_high_dosage:Empagliflozin_low_dosage | 0 | 0.23894576 | NA | 0.23894576 | NA | NA | NA | NA |
| 89 | Dapagliflozin_high_dosage:Exenatide | 0 | -0.210473906 | NA | -0.210473906 | NA | NA | NA | NA |
| 90 | Dapagliflozin_high_dosage:Inject_semaglutide_high_dosage | 0 | -0.592280938 | NA | -0.592280938 | NA | NA | NA | NA |
| 91 | Dapagliflozin_high_dosage:Inject_semaglutide_low_dosage | 0 | 0.06367852 | NA | 0.06367852 | NA | NA | NA | NA |
| 92 | Dapagliflozin_high_dosage:Inject_semaglutide_medium_dosage | 0 | 1.596684756 | NA | 1.596684756 | NA | NA | NA | NA |
| 93 | Dapagliflozin_high_dosage:Liraglutide | 0 | 0.346511787 | NA | 0.346511787 | NA | NA | NA | NA |
| 94 | Dapagliflozin_high_dosage:Lixisenatide | 0 | -1.728568626 | NA | -1.728568626 | NA | NA | NA | NA |
| 95 | Dapagliflozin_high_dosage:Oral_semaglutide | 0 | 0.951759622 | NA | 0.951759622 | NA | NA | NA | NA |
| 96 | Dapagliflozin_high_dosage:Placebo_or_Control | 4 | 0.489108764 | 0.489108764 | NA | NA | NA | NA | NA |
| 97 | Dapagliflozin_high_dosage:Sotagliflozin | 0 | 1.562898575 | NA | 1.562898575 | NA | NA | NA | NA |
| 98 | Dapagliflozin_high_dosage:Tirzepatide_high_dosage | 0 | -0.050430256 | NA | -0.050430256 | NA | NA | NA | NA |
| 99 | Dapagliflozin_high_dosage:Tirzepatide_low_dosage | 0 | 0.310773138 | NA | 0.310773138 | NA | NA | NA | NA |
| 100 | Dapagliflozin_high_dosage:Tirzepatide_medium_dosage | 0 | 1.25247884 | NA | 1.25247884 | NA | NA | NA | NA |
| 101 | Dulaglutide_low_dosage:Dulaglutide_medium_dosage | 2 | 1.119787371 | 1.120016553 | 1.119562535 | 0.000454017 | -3.194711114 | 3.195619149 | 0.999777788 |
| 102 | Dulaglutide_low_dosage:Efpeglenatide_high_dosage | 0 | 1.550565906 | NA | 1.550565906 | NA | NA | NA | NA |
| 103 | Dulaglutide_low_dosage:Efpeglenatide_low_dosage | 0 | -0.08646099 | NA | -0.08646099 | NA | NA | NA | NA |
| 104 | Dulaglutide_low_dosage:Efpeglenatide_medium_dosage | 0 | 1.553030008 | NA | 1.553030008 | NA | NA | NA | NA |
| 105 | Dulaglutide_low_dosage:Empagliflozin_low_dosage | 0 | 0.093603102 | NA | 0.093603102 | NA | NA | NA | NA |
| 106 | Dulaglutide_low_dosage:Exenatide | 0 | -0.355816563 | NA | -0.355816563 | NA | NA | NA | NA |
| 107 | Dulaglutide_low_dosage:Inject_semaglutide_high_dosage | 0 | -0.737623596 | NA | -0.737623596 | NA | NA | NA | NA |
| 108 | Dulaglutide_low_dosage:Inject_semaglutide_low_dosage | 0 | -0.081664138 | NA | -0.081664138 | NA | NA | NA | NA |
| 109 | Dulaglutide_low_dosage:Inject_semaglutide_medium_dosage | 0 | 1.451342099 | NA | 1.451342099 | NA | NA | NA | NA |
| 110 | Dulaglutide_low_dosage:Liraglutide | 0 | 0.20116913 | NA | 0.20116913 | NA | NA | NA | NA |
| 111 | Dulaglutide_low_dosage:Lixisenatide | 0 | -1.873911283 | NA | -1.873911283 | NA | NA | NA | NA |
| 112 | Dulaglutide_low_dosage:Oral_semaglutide | 0 | 0.806416964 | NA | 0.806416964 | NA | NA | NA | NA |
| 113 | Dulaglutide_low_dosage:Placebo_or_Control | 3 | 0.343766106 | 0.343613364 | 0.344067381 | -0.000454017 | -3.195619149 | 3.194711114 | 0.999777788 |
| 114 | Dulaglutide_low_dosage:Sotagliflozin | 0 | 1.417555917 | NA | 1.417555917 | NA | NA | NA | NA |
| 115 | Dulaglutide_low_dosage:Tirzepatide_high_dosage | 0 | -0.195772914 | NA | -0.195772914 | NA | NA | NA | NA |
| 116 | Dulaglutide_low_dosage:Tirzepatide_low_dosage | 0 | 0.165430481 | NA | 0.165430481 | NA | NA | NA | NA |
| 117 | Dulaglutide_low_dosage:Tirzepatide_medium_dosage | 0 | 1.107136182 | NA | 1.107136182 | NA | NA | NA | NA |
| 118 | Dulaglutide_medium_dosage:Efpeglenatide_high_dosage | 0 | 0.430778535 | NA | 0.430778535 | NA | NA | NA | NA |
| 119 | Dulaglutide_medium_dosage:Efpeglenatide_low_dosage | 0 | -1.206248361 | NA | -1.206248361 | NA | NA | NA | NA |
| 120 | Dulaglutide_medium_dosage:Efpeglenatide_medium_dosage | 0 | 0.433242637 | NA | 0.433242637 | NA | NA | NA | NA |
| 121 | Dulaglutide_medium_dosage:Empagliflozin_low_dosage | 0 | -1.026184269 | NA | -1.026184269 | NA | NA | NA | NA |
| 122 | Dulaglutide_medium_dosage:Exenatide | 0 | -1.475603934 | NA | -1.475603934 | NA | NA | NA | NA |
| 123 | Dulaglutide_medium_dosage:Inject_semaglutide_high_dosage | 0 | -1.857410967 | NA | -1.857410967 | NA | NA | NA | NA |
| 124 | Dulaglutide_medium_dosage:Inject_semaglutide_low_dosage | 0 | -1.201451509 | NA | -1.201451509 | NA | NA | NA | NA |
| 125 | Dulaglutide_medium_dosage:Inject_semaglutide_medium_dosage | 0 | 0.331554728 | NA | 0.331554728 | NA | NA | NA | NA |
| 126 | Dulaglutide_medium_dosage:Liraglutide | 0 | -0.918618241 | NA | -0.918618241 | NA | NA | NA | NA |
| 127 | Dulaglutide_medium_dosage:Lixisenatide | 0 | -2.993698654 | NA | -2.993698654 | NA | NA | NA | NA |
| 128 | Dulaglutide_medium_dosage:Oral_semaglutide | 0 | -0.313370407 | NA | -0.313370407 | NA | NA | NA | NA |
| 129 | Dulaglutide_medium_dosage:Placebo_or_Control | 2 | -0.776021265 | -0.775949171 | -0.776403189 | 0.000454017 | -3.194711114 | 3.195619149 | 0.999777788 |
| 130 | Dulaglutide_medium_dosage:Sotagliflozin | 0 | 0.297768546 | NA | 0.297768546 | NA | NA | NA | NA |
| 131 | Dulaglutide_medium_dosage:Tirzepatide_high_dosage | 0 | -1.315560284 | NA | -1.315560284 | NA | NA | NA | NA |
| 132 | Dulaglutide_medium_dosage:Tirzepatide_low_dosage | 0 | -0.95435689 | NA | -0.95435689 | NA | NA | NA | NA |
| 133 | Dulaglutide_medium_dosage:Tirzepatide_medium_dosage | 0 | -0.012651188 | NA | -0.012651188 | NA | NA | NA | NA |
| 134 | Efpeglenatide_high_dosage:Efpeglenatide_low_dosage | 1 | -1.637026896 | -1.58618105 | -1.715025256 | 0.128844205 | -4.751236447 | 5.008924858 | 0.95873021 |
| 135 | Efpeglenatide_high_dosage:Efpeglenatide_medium_dosage | 0 | 0.002464102 | NA | 0.002464102 | NA | NA | NA | NA |
| 136 | Efpeglenatide_high_dosage:Empagliflozin_low_dosage | 0 | -1.456962804 | NA | -1.456962804 | NA | NA | NA | NA |
| 137 | Efpeglenatide_high_dosage:Exenatide | 0 | -1.906382469 | NA | -1.906382469 | NA | NA | NA | NA |
| 138 | Efpeglenatide_high_dosage:Inject_semaglutide_high_dosage | 0 | -2.288189502 | NA | -2.288189502 | NA | NA | NA | NA |
| 139 | Efpeglenatide_high_dosage:Inject_semaglutide_low_dosage | 0 | -1.632230044 | NA | -1.632230044 | NA | NA | NA | NA |
| 140 | Efpeglenatide_high_dosage:Inject_semaglutide_medium_dosage | 0 | -0.099223807 | NA | -0.099223807 | NA | NA | NA | NA |
| 141 | Efpeglenatide_high_dosage:Liraglutide | 0 | -1.349396776 | NA | -1.349396776 | NA | NA | NA | NA |
| 142 | Efpeglenatide_high_dosage:Lixisenatide | 0 | -3.424477189 | NA | -3.424477189 | NA | NA | NA | NA |
| 143 | Efpeglenatide_high_dosage:Oral_semaglutide | 0 | -0.744148942 | NA | -0.744148942 | NA | NA | NA | NA |
| 144 | Efpeglenatide_high_dosage:Placebo_or_Control | 1 | -1.2067998 | -1.263367522 | -1.134523316 | -0.128844205 | -5.008924858 | 4.751236447 | 0.95873021 |
| 145 | Efpeglenatide_high_dosage:Sotagliflozin | 0 | -0.133009989 | NA | -0.133009989 | NA | NA | NA | NA |
| 146 | Efpeglenatide_high_dosage:Tirzepatide_high_dosage | 0 | -1.746338819 | NA | -1.746338819 | NA | NA | NA | NA |
| 147 | Efpeglenatide_high_dosage:Tirzepatide_low_dosage | 0 | -1.385135425 | NA | -1.385135425 | NA | NA | NA | NA |
| 148 | Efpeglenatide_high_dosage:Tirzepatide_medium_dosage | 0 | -0.443429723 | NA | -0.443429723 | NA | NA | NA | NA |
| 149 | Efpeglenatide_low_dosage:Efpeglenatide_medium_dosage | 1 | 1.639490998 | 1.738649644 | 1.486990774 | 0.25165887 | -4.628877876 | 5.132195616 | 0.919500346 |
| 150 | Efpeglenatide_low_dosage:Empagliflozin_low_dosage | 0 | 0.180064092 | NA | 0.180064092 | NA | NA | NA | NA |
| 151 | Efpeglenatide_low_dosage:Exenatide | 0 | -0.269355573 | NA | -0.269355573 | NA | NA | NA | NA |
| 152 | Efpeglenatide_low_dosage:Inject_semaglutide_high_dosage | 0 | -0.651162606 | NA | -0.651162606 | NA | NA | NA | NA |
| 153 | Efpeglenatide_low_dosage:Inject_semaglutide_low_dosage | 0 | 0.004796852 | NA | 0.004796852 | NA | NA | NA | NA |
| 154 | Efpeglenatide_low_dosage:Inject_semaglutide_medium_dosage | 0 | 1.537803089 | NA | 1.537803089 | NA | NA | NA | NA |
| 155 | Efpeglenatide_low_dosage:Liraglutide | 0 | 0.28763012 | NA | 0.28763012 | NA | NA | NA | NA |
| 156 | Efpeglenatide_low_dosage:Lixisenatide | 0 | -1.787450293 | NA | -1.787450293 | NA | NA | NA | NA |
| 157 | Efpeglenatide_low_dosage:Oral_semaglutide | 0 | 0.892877954 | NA | 0.892877954 | NA | NA | NA | NA |
| 158 | Efpeglenatide_low_dosage:Placebo_or_Control | 2 | 0.430227096 | 0.404719137 | 0.481406925 | -0.076687788 | -3.93367472 | 3.780299143 | 0.968914612 |
| 159 | Efpeglenatide_low_dosage:Sotagliflozin | 0 | 1.504016907 | NA | 1.504016907 | NA | NA | NA | NA |
| 160 | Efpeglenatide_low_dosage:Tirzepatide_high_dosage | 0 | -0.109311924 | NA | -0.109311924 | NA | NA | NA | NA |
| 161 | Efpeglenatide_low_dosage:Tirzepatide_low_dosage | 0 | 0.251891471 | NA | 0.251891471 | NA | NA | NA | NA |
| 162 | Efpeglenatide_low_dosage:Tirzepatide_medium_dosage | 0 | 1.193597173 | NA | 1.193597173 | NA | NA | NA | NA |
| 163 | Efpeglenatide_medium_dosage:Empagliflozin_low_dosage | 0 | -1.459426906 | NA | -1.459426906 | NA | NA | NA | NA |
| 164 | Efpeglenatide_medium_dosage:Exenatide | 0 | -1.908846572 | NA | -1.908846572 | NA | NA | NA | NA |
| 165 | Efpeglenatide_medium_dosage:Inject_semaglutide_high_dosage | 0 | -2.290653604 | NA | -2.290653604 | NA | NA | NA | NA |
| 166 | Efpeglenatide_medium_dosage:Inject_semaglutide_low_dosage | 0 | -1.634694146 | NA | -1.634694146 | NA | NA | NA | NA |
| 167 | Efpeglenatide_medium_dosage:Inject_semaglutide_medium_dosage | 0 | -0.10168791 | NA | -0.10168791 | NA | NA | NA | NA |
| 168 | Efpeglenatide_medium_dosage:Liraglutide | 0 | -1.351860879 | NA | -1.351860879 | NA | NA | NA | NA |
| 169 | Efpeglenatide_medium_dosage:Lixisenatide | 0 | -3.426941292 | NA | -3.426941292 | NA | NA | NA | NA |
| 170 | Efpeglenatide_medium_dosage:Oral_semaglutide | 0 | -0.746613044 | NA | -0.746613044 | NA | NA | NA | NA |
| 171 | Efpeglenatide_medium_dosage:Placebo_or_Control | 1 | -1.209263902 | -1.098612289 | -1.350271159 | 0.25165887 | -4.628877876 | 5.132195616 | 0.919500346 |
| 172 | Efpeglenatide_medium_dosage:Sotagliflozin | 0 | -0.135474091 | NA | -0.135474091 | NA | NA | NA | NA |
| 173 | Efpeglenatide_medium_dosage:Tirzepatide_high_dosage | 0 | -1.748802922 | NA | -1.748802922 | NA | NA | NA | NA |
| 174 | Efpeglenatide_medium_dosage:Tirzepatide_low_dosage | 0 | -1.387599528 | NA | -1.387599528 | NA | NA | NA | NA |
| 175 | Efpeglenatide_medium_dosage:Tirzepatide_medium_dosage | 0 | -0.445893826 | NA | -0.445893826 | NA | NA | NA | NA |
| 176 | Empagliflozin_low_dosage:Exenatide | 0 | -0.449419666 | NA | -0.449419666 | NA | NA | NA | NA |
| 177 | Empagliflozin_low_dosage:Inject_semaglutide_high_dosage | 0 | -0.831226698 | NA | -0.831226698 | NA | NA | NA | NA |
| 178 | Empagliflozin_low_dosage:Inject_semaglutide_low_dosage | 0 | -0.17526724 | NA | -0.17526724 | NA | NA | NA | NA |
| 179 | Empagliflozin_low_dosage:Inject_semaglutide_medium_dosage | 0 | 1.357738996 | NA | 1.357738996 | NA | NA | NA | NA |
| 180 | Empagliflozin_low_dosage:Liraglutide | 0 | 0.107566027 | NA | 0.107566027 | NA | NA | NA | NA |
| 181 | Empagliflozin_low_dosage:Lixisenatide | 0 | -1.967514386 | NA | -1.967514386 | NA | NA | NA | NA |
| 182 | Empagliflozin_low_dosage:Oral_semaglutide | 0 | 0.712813862 | NA | 0.712813862 | NA | NA | NA | NA |
| 183 | Empagliflozin_low_dosage:Placebo_or_Control | 3 | 0.250163004 | 0.250163004 | NA | NA | NA | NA | NA |
| 184 | Empagliflozin_low_dosage:Sotagliflozin | 0 | 1.323952815 | NA | 1.323952815 | NA | NA | NA | NA |
| 185 | Empagliflozin_low_dosage:Tirzepatide_high_dosage | 0 | -0.289376016 | NA | -0.289376016 | NA | NA | NA | NA |
| 186 | Empagliflozin_low_dosage:Tirzepatide_low_dosage | 0 | 0.071827378 | NA | 0.071827378 | NA | NA | NA | NA |
| 187 | Empagliflozin_low_dosage:Tirzepatide_medium_dosage | 0 | 1.01353308 | NA | 1.01353308 | NA | NA | NA | NA |
| 188 | Exenatide:Inject_semaglutide_high_dosage | 0 | -0.381807033 | NA | -0.381807033 | NA | NA | NA | NA |
| 189 | Exenatide:Inject_semaglutide_low_dosage | 0 | 0.274152426 | NA | 0.274152426 | NA | NA | NA | NA |
| 190 | Exenatide:Inject_semaglutide_medium_dosage | 0 | 1.807158662 | NA | 1.807158662 | NA | NA | NA | NA |
| 191 | Exenatide:Liraglutide | 0 | 0.556985693 | NA | 0.556985693 | NA | NA | NA | NA |
| 192 | Exenatide:Lixisenatide | 0 | -1.51809472 | NA | -1.51809472 | NA | NA | NA | NA |
| 193 | Exenatide:Oral_semaglutide | 0 | 1.162233527 | NA | 1.162233527 | NA | NA | NA | NA |
| 194 | Exenatide:Placebo_or_Control | 1 | 0.699582669 | 0.699582669 | NA | NA | NA | NA | NA |
| 195 | Exenatide:Sotagliflozin | 0 | 1.77337248 | NA | 1.77337248 | NA | NA | NA | NA |
| 196 | Exenatide:Tirzepatide_high_dosage | 0 | 0.16004365 | NA | 0.16004365 | NA | NA | NA | NA |
| 197 | Exenatide:Tirzepatide_low_dosage | 0 | 0.521247044 | NA | 0.521247044 | NA | NA | NA | NA |
| 198 | Exenatide:Tirzepatide_medium_dosage | 0 | 1.462952746 | NA | 1.462952746 | NA | NA | NA | NA |
| 199 | Inject_semaglutide_high_dosage:Inject_semaglutide_low_dosage | 0 | 0.655959458 | NA | 0.655959458 | NA | NA | NA | NA |
| 200 | Inject_semaglutide_high_dosage:Inject_semaglutide_medium_dosage | 0 | 2.188965695 | NA | 2.188965695 | NA | NA | NA | NA |
| 201 | Inject_semaglutide_high_dosage:Liraglutide | 0 | 0.938792726 | NA | 0.938792726 | NA | NA | NA | NA |
| 202 | Inject_semaglutide_high_dosage:Lixisenatide | 0 | -1.136287687 | NA | -1.136287687 | NA | NA | NA | NA |
| 203 | Inject_semaglutide_high_dosage:Oral_semaglutide | 0 | 1.54404056 | NA | 1.54404056 | NA | NA | NA | NA |
| 204 | Inject_semaglutide_high_dosage:Placebo_or_Control | 2 | 1.081389702 | 1.081389702 | NA | NA | NA | NA | NA |
| 205 | Inject_semaglutide_high_dosage:Sotagliflozin | 0 | 2.155179513 | NA | 2.155179513 | NA | NA | NA | NA |
| 206 | Inject_semaglutide_high_dosage:Tirzepatide_high_dosage | 0 | 0.541850682 | NA | 0.541850682 | NA | NA | NA | NA |
| 207 | Inject_semaglutide_high_dosage:Tirzepatide_low_dosage | 0 | 0.903054077 | NA | 0.903054077 | NA | NA | NA | NA |
| 208 | Inject_semaglutide_high_dosage:Tirzepatide_medium_dosage | 0 | 1.844759779 | NA | 1.844759779 | NA | NA | NA | NA |
| 209 | Inject_semaglutide_low_dosage:Inject_semaglutide_medium_dosage | 1 | 1.533006236 | 1.530532646 | 1.536679131 | -0.006146485 | -4.798779763 | 4.786486793 | 0.997994418 |
| 210 | Inject_semaglutide_low_dosage:Liraglutide | 1 | 0.282833267 | -0.828066498 | 0.885091426 | -1.713157924 | -5.700388939 | 2.27407309 | 0.399720668 |
| 211 | Inject_semaglutide_low_dosage:Lixisenatide | 0 | -1.792247146 | NA | -1.792247146 | NA | NA | NA | NA |
| 212 | Inject_semaglutide_low_dosage:Oral_semaglutide | 0 | 0.888081102 | NA | 0.888081102 | NA | NA | NA | NA |
| 213 | Inject_semaglutide_low_dosage:Placebo_or_Control | 2 | 0.425430244 | 0.951391033 | -0.377216397 | 1.32860743 | -2.178365961 | 4.835580822 | 0.457768051 |
| 214 | Inject_semaglutide_low_dosage:Sotagliflozin | 0 | 1.499220055 | NA | 1.499220055 | NA | NA | NA | NA |
| 215 | Inject_semaglutide_low_dosage:Tirzepatide_high_dosage | 0 | -0.114108776 | NA | -0.114108776 | NA | NA | NA | NA |
| 216 | Inject_semaglutide_low_dosage:Tirzepatide_low_dosage | 0 | 0.247094618 | NA | 0.247094618 | NA | NA | NA | NA |
| 217 | Inject_semaglutide_low_dosage:Tirzepatide_medium_dosage | 0 | 1.18880032 | NA | 1.18880032 | NA | NA | NA | NA |
| 218 | Inject_semaglutide_medium_dosage:Liraglutide | 0 | -1.250172969 | NA | -1.250172969 | NA | NA | NA | NA |
| 219 | Inject_semaglutide_medium_dosage:Lixisenatide | 0 | -3.325253382 | NA | -3.325253382 | NA | NA | NA | NA |
| 220 | Inject_semaglutide_medium_dosage:Oral_semaglutide | 0 | -0.644925135 | NA | -0.644925135 | NA | NA | NA | NA |
| 221 | Inject_semaglutide_medium_dosage:Placebo_or_Control | 1 | -1.107575993 | -1.110322024 | -1.104175539 | -0.006146485 | -4.798779763 | 4.786486793 | 0.997994418 |
| 222 | Inject_semaglutide_medium_dosage:Sotagliflozin | 0 | -0.033786182 | NA | -0.033786182 | NA | NA | NA | NA |
| 223 | Inject_semaglutide_medium_dosage:Tirzepatide_high_dosage | 0 | -1.647115012 | NA | -1.647115012 | NA | NA | NA | NA |
| 224 | Inject_semaglutide_medium_dosage:Tirzepatide_low_dosage | 0 | -1.285911618 | NA | -1.285911618 | NA | NA | NA | NA |
| 225 | Inject_semaglutide_medium_dosage:Tirzepatide_medium_dosage | 0 | -0.344205916 | NA | -0.344205916 | NA | NA | NA | NA |
| 226 | Liraglutide:Lixisenatide | 0 | -2.075080413 | NA | -2.075080413 | NA | NA | NA | NA |
| 227 | Liraglutide:Oral_semaglutide | 1 | 0.605247834 | 1.127918415 | 0.001445219 | 1.126473197 | -3.585132803 | 5.838079196 | 0.639357349 |
| 228 | Liraglutide:Placebo_or_Control | 2 | 0.142596976 | -0.149757165 | 1.474351043 | -1.624108208 | -4.825130242 | 1.576913826 | 0.320013378 |
| 229 | Liraglutide:Sotagliflozin | 0 | 1.216386787 | NA | 1.216386787 | NA | NA | NA | NA |
| 230 | Liraglutide:Tirzepatide_high_dosage | 0 | -0.396942043 | NA | -0.396942043 | NA | NA | NA | NA |
| 231 | Liraglutide:Tirzepatide_low_dosage | 0 | -0.035738649 | NA | -0.035738649 | NA | NA | NA | NA |
| 232 | Liraglutide:Tirzepatide_medium_dosage | 0 | 0.905967053 | NA | 0.905967053 | NA | NA | NA | NA |
| 233 | Lixisenatide:Oral_semaglutide | 0 | 2.680328247 | NA | 2.680328247 | NA | NA | NA | NA |
| 234 | Lixisenatide:Placebo_or_Control | 1 | 2.217677389 | 2.217677389 | NA | NA | NA | NA | NA |
| 235 | Lixisenatide:Sotagliflozin | 0 | 3.2914672 | NA | 3.2914672 | NA | NA | NA | NA |
| 236 | Lixisenatide:Tirzepatide_high_dosage | 0 | 1.67813837 | NA | 1.67813837 | NA | NA | NA | NA |
| 237 | Lixisenatide:Tirzepatide_low_dosage | 0 | 2.039341764 | NA | 2.039341764 | NA | NA | NA | NA |
| 238 | Lixisenatide:Tirzepatide_medium_dosage | 0 | 2.981047466 | NA | 2.981047466 | NA | NA | NA | NA |
| 239 | Oral_semaglutide:Placebo_or_Control | 1 | -0.462650858 | 0.058312482 | -1.068160714 | 1.126473197 | -3.585132803 | 5.838079196 | 0.639357349 |
| 240 | Oral_semaglutide:Sotagliflozin | 0 | 0.611138953 | NA | 0.611138953 | NA | NA | NA | NA |
| 241 | Oral_semaglutide:Tirzepatide_high_dosage | 0 | -1.002189877 | NA | -1.002189877 | NA | NA | NA | NA |
| 242 | Oral_semaglutide:Tirzepatide_low_dosage | 0 | -0.640986483 | NA | -0.640986483 | NA | NA | NA | NA |
| 243 | Oral_semaglutide:Tirzepatide_medium_dosage | 0 | 0.300719219 | NA | 0.300719219 | NA | NA | NA | NA |
| 244 | Sotagliflozin:Placebo_or_Control | 1 | -1.073789811 | -1.073789811 | NA | NA | NA | NA | NA |
| 245 | Tirzepatide_high_dosage:Placebo_or_Control | 3 | 0.53953902 | 0.714439938 | 0.209928812 | 0.504511126 | -2.640686589 | 3.649708841 | 0.753223661 |
| 246 | Tirzepatide_low_dosage:Placebo_or_Control | 1 | 0.178335625 | 0.023256862 | 0.281887615 | -0.258630753 | -3.842329395 | 3.325067889 | 0.887516047 |
| 247 | Tirzepatide_medium_dosage:Placebo_or_Control | 1 | -0.763370077 | -1.080071781 | -0.584181021 | -0.49589076 | -4.504349323 | 3.512567803 | 0.808416564 |
| 248 | Sotagliflozin:Tirzepatide_high_dosage | 0 | -1.613328831 | NA | -1.613328831 | NA | NA | NA | NA |
| 249 | Sotagliflozin:Tirzepatide_low_dosage | 0 | -1.252125436 | NA | -1.252125436 | NA | NA | NA | NA |
| 250 | Sotagliflozin:Tirzepatide_medium_dosage | 0 | -0.310419734 | NA | -0.310419734 | NA | NA | NA | NA |
| 251 | Tirzepatide_high_dosage:Tirzepatide_low_dosage | 3 | 0.361203394 | 0.346135027 | 0.389577645 | -0.043442618 | -3.188891228 | 3.102005993 | 0.978404239 |
| 252 | Tirzepatide_high_dosage:Tirzepatide_medium_dosage | 2 | 1.302909096 | 1.062589241 | 1.62200758 | -0.559418339 | -4.023836504 | 2.904999827 | 0.751633661 |
| 253 | Tirzepatide_low_dosage:Tirzepatide_medium_dosage | 1 | 0.941705702 | 1.103301629 | 0.850259853 | 0.253041776 | -3.755578194 | 4.261661747 | 0.90153573 |

**eTable 7E: Side-splitting model inconsistency of primary outcome: subgroup of breast tumor**

|  | Comparison | No.Studies | NMA | Direct | Indirect | Difference | Diff_95CI_lower | Diff_95CI_upper | *p* value |
| --- | --- | --- | --- | --- | --- | --- | --- | --- | --- |
| 1 | Albiglutide:Bexagliflozin | 0 | -0.430708834 | NA | -0.430708834 | NA | NA | NA | NA |
| 2 | Albiglutide:Canagliflozin_high_dosage | 0 | -0.993348475 | NA | -0.993348475 | NA | NA | NA | NA |
| 3 | Albiglutide:Canagliflozin_low_dosage | 0 | -0.796866467 | NA | -0.796866467 | NA | NA | NA | NA |
| 4 | Albiglutide:Dapagliflozin_high_dosage | 0 | -0.199020153 | NA | -0.199020153 | NA | NA | NA | NA |
| 5 | Albiglutide:Dapagliflozin_low_dosage | 0 | -0.805926302 | NA | -0.805926302 | NA | NA | NA | NA |
| 6 | Albiglutide:Dapagliflozin_medium_dosage | 0 | 0.655996444 | NA | 0.655996444 | NA | NA | NA | NA |
| 7 | Albiglutide:Dulaglutide_high_dosage | 0 | -0.918883631 | NA | -0.918883631 | NA | NA | NA | NA |
| 8 | Albiglutide:Dulaglutide_low_dosage | 0 | -0.192946627 | NA | -0.192946627 | NA | NA | NA | NA |
| 9 | Albiglutide:Dulaglutide_medium_dosage | 0 | -0.220283329 | NA | -0.220283329 | NA | NA | NA | NA |
| 10 | Albiglutide:Efpeglenatide_high_dosage | 0 | -1.693352048 | NA | -1.693352048 | NA | NA | NA | NA |
| 11 | Albiglutide:Efpeglenatide_low_dosage | 0 | -1.595941932 | NA | -1.595941932 | NA | NA | NA | NA |
| 12 | Albiglutide:Efpeglenatide_medium_dosage | 0 | -0.387599015 | NA | -0.387599015 | NA | NA | NA | NA |
| 13 | Albiglutide:Empagliflozin_high_dosage | 0 | -0.226379204 | NA | -0.226379204 | NA | NA | NA | NA |
| 14 | Albiglutide:Empagliflozin_low_dosage | 0 | -0.169249462 | NA | -0.169249462 | NA | NA | NA | NA |
| 15 | Albiglutide:Ertugliflozin_high_dosage | 0 | -1.020408197 | NA | -1.020408197 | NA | NA | NA | NA |
| 16 | Albiglutide:Ertugliflozin_low_dosage | 0 | -0.44792574 | NA | -0.44792574 | NA | NA | NA | NA |
| 17 | Albiglutide:Exenatide | 0 | -0.670730871 | NA | -0.670730871 | NA | NA | NA | NA |
| 18 | Albiglutide:Inject_semaglutide_high_dosage | 0 | -0.27853165 | NA | -0.27853165 | NA | NA | NA | NA |
| 19 | Albiglutide:Inject_semaglutide_low_dosage | 0 | 0.125498627 | NA | 0.125498627 | NA | NA | NA | NA |
| 20 | Albiglutide:Inject_semaglutide_medium_dosage | 0 | -0.881077245 | NA | -0.881077245 | NA | NA | NA | NA |
| 21 | Albiglutide:Liraglutide | 0 | -0.639628293 | NA | -0.639628293 | NA | NA | NA | NA |
| 22 | Albiglutide:Lixisenatide | 0 | -0.815784681 | NA | -0.815784681 | NA | NA | NA | NA |
| 23 | Albiglutide:Oral_semaglutide | 0 | 0.146710036 | NA | 0.146710036 | NA | NA | NA | NA |
| 24 | Albiglutide:Placebo_or_Control | 5 | -0.393077766 | -0.393077766 | NA | NA | NA | NA | NA |
| 25 | Albiglutide:Sotagliflozin | 0 | -0.051924741 | NA | -0.051924741 | NA | NA | NA | NA |
| 26 | Albiglutide:Tirzepatide_high_dosage | 0 | -0.694370952 | NA | -0.694370952 | NA | NA | NA | NA |
| 27 | Albiglutide:Tirzepatide_low_dosage | 0 | -0.590439363 | NA | -0.590439363 | NA | NA | NA | NA |
| 28 | Albiglutide:Tirzepatide_medium_dosage | 0 | -1.011811753 | NA | -1.011811753 | NA | NA | NA | NA |
| 29 | Bexagliflozin:Canagliflozin_high_dosage | 0 | -0.56263964 | NA | -0.56263964 | NA | NA | NA | NA |
| 30 | Bexagliflozin:Canagliflozin_low_dosage | 0 | -0.366157633 | NA | -0.366157633 | NA | NA | NA | NA |
| 31 | Bexagliflozin:Dapagliflozin_high_dosage | 0 | 0.231688681 | NA | 0.231688681 | NA | NA | NA | NA |
| 32 | Bexagliflozin:Dapagliflozin_low_dosage | 0 | -0.375217468 | NA | -0.375217468 | NA | NA | NA | NA |
| 33 | Bexagliflozin:Dapagliflozin_medium_dosage | 0 | 1.086705279 | NA | 1.086705279 | NA | NA | NA | NA |
| 34 | Bexagliflozin:Dulaglutide_high_dosage | 0 | -0.488174797 | NA | -0.488174797 | NA | NA | NA | NA |
| 35 | Bexagliflozin:Dulaglutide_low_dosage | 0 | 0.237762207 | NA | 0.237762207 | NA | NA | NA | NA |
| 36 | Bexagliflozin:Dulaglutide_medium_dosage | 0 | 0.210425505 | NA | 0.210425505 | NA | NA | NA | NA |
| 37 | Bexagliflozin:Efpeglenatide_high_dosage | 0 | -1.262643214 | NA | -1.262643214 | NA | NA | NA | NA |
| 38 | Bexagliflozin:Efpeglenatide_low_dosage | 0 | -1.165233098 | NA | -1.165233098 | NA | NA | NA | NA |
| 39 | Bexagliflozin:Efpeglenatide_medium_dosage | 0 | 0.043109819 | NA | 0.043109819 | NA | NA | NA | NA |
| 40 | Bexagliflozin:Empagliflozin_high_dosage | 0 | 0.204329631 | NA | 0.204329631 | NA | NA | NA | NA |
| 41 | Bexagliflozin:Empagliflozin_low_dosage | 0 | 0.261459372 | NA | 0.261459372 | NA | NA | NA | NA |
| 42 | Bexagliflozin:Ertugliflozin_high_dosage | 0 | -0.589699363 | NA | -0.589699363 | NA | NA | NA | NA |
| 43 | Bexagliflozin:Ertugliflozin_low_dosage | 0 | -0.017216906 | NA | -0.017216906 | NA | NA | NA | NA |
| 44 | Bexagliflozin:Exenatide | 0 | -0.240022037 | NA | -0.240022037 | NA | NA | NA | NA |
| 45 | Bexagliflozin:Inject_semaglutide_high_dosage | 0 | 0.152177184 | NA | 0.152177184 | NA | NA | NA | NA |
| 46 | Bexagliflozin:Inject_semaglutide_low_dosage | 0 | 0.556207461 | NA | 0.556207461 | NA | NA | NA | NA |
| 47 | Bexagliflozin:Inject_semaglutide_medium_dosage | 0 | -0.45036841 | NA | -0.45036841 | NA | NA | NA | NA |
| 48 | Bexagliflozin:Liraglutide | 0 | -0.208919459 | NA | -0.208919459 | NA | NA | NA | NA |
| 49 | Bexagliflozin:Lixisenatide | 0 | -0.385075846 | NA | -0.385075846 | NA | NA | NA | NA |
| 50 | Bexagliflozin:Oral_semaglutide | 0 | 0.57741887 | NA | 0.57741887 | NA | NA | NA | NA |
| 51 | Bexagliflozin:Placebo_or_Control | 1 | 0.037631068 | 0.037631068 | NA | NA | NA | NA | NA |
| 52 | Bexagliflozin:Sotagliflozin | 0 | 0.378784093 | NA | 0.378784093 | NA | NA | NA | NA |
| 53 | Bexagliflozin:Tirzepatide_high_dosage | 0 | -0.263662118 | NA | -0.263662118 | NA | NA | NA | NA |
| 54 | Bexagliflozin:Tirzepatide_low_dosage | 0 | -0.159730529 | NA | -0.159730529 | NA | NA | NA | NA |
| 55 | Bexagliflozin:Tirzepatide_medium_dosage | 0 | -0.581102919 | NA | -0.581102919 | NA | NA | NA | NA |
| 56 | Canagliflozin_high_dosage:Canagliflozin_low_dosage | 3 | 0.196482007 | 0.270846331 | -0.0165421 | 0.287388431 | -1.70216072 | 2.276937581 | 0.777088773 |
| 57 | Canagliflozin_high_dosage:Dapagliflozin_high_dosage | 0 | 0.794328322 | NA | 0.794328322 | NA | NA | NA | NA |
| 58 | Canagliflozin_high_dosage:Dapagliflozin_low_dosage | 0 | 0.187422173 | NA | 0.187422173 | NA | NA | NA | NA |
| 59 | Canagliflozin_high_dosage:Dapagliflozin_medium_dosage | 0 | 1.649344919 | NA | 1.649344919 | NA | NA | NA | NA |
| 60 | Canagliflozin_high_dosage:Dulaglutide_high_dosage | 0 | 0.074464844 | NA | 0.074464844 | NA | NA | NA | NA |
| 61 | Canagliflozin_high_dosage:Dulaglutide_low_dosage | 0 | 0.800401847 | NA | 0.800401847 | NA | NA | NA | NA |
| 62 | Canagliflozin_high_dosage:Dulaglutide_medium_dosage | 0 | 0.773065146 | NA | 0.773065146 | NA | NA | NA | NA |
| 63 | Canagliflozin_high_dosage:Efpeglenatide_high_dosage | 0 | -0.700003573 | NA | -0.700003573 | NA | NA | NA | NA |
| 64 | Canagliflozin_high_dosage:Efpeglenatide_low_dosage | 0 | -0.602593457 | NA | -0.602593457 | NA | NA | NA | NA |
| 65 | Canagliflozin_high_dosage:Efpeglenatide_medium_dosage | 0 | 0.60574946 | NA | 0.60574946 | NA | NA | NA | NA |
| 66 | Canagliflozin_high_dosage:Empagliflozin_high_dosage | 0 | 0.766969271 | NA | 0.766969271 | NA | NA | NA | NA |
| 67 | Canagliflozin_high_dosage:Empagliflozin_low_dosage | 0 | 0.824099012 | NA | 0.824099012 | NA | NA | NA | NA |
| 68 | Canagliflozin_high_dosage:Ertugliflozin_high_dosage | 0 | -0.027059722 | NA | -0.027059722 | NA | NA | NA | NA |
| 69 | Canagliflozin_high_dosage:Ertugliflozin_low_dosage | 0 | 0.545422735 | NA | 0.545422735 | NA | NA | NA | NA |
| 70 | Canagliflozin_high_dosage:Exenatide | 0 | 0.322617604 | NA | 0.322617604 | NA | NA | NA | NA |
| 71 | Canagliflozin_high_dosage:Inject_semaglutide_high_dosage | 0 | 0.714816824 | NA | 0.714816824 | NA | NA | NA | NA |
| 72 | Canagliflozin_high_dosage:Inject_semaglutide_low_dosage | 0 | 1.118847101 | NA | 1.118847101 | NA | NA | NA | NA |
| 73 | Canagliflozin_high_dosage:Inject_semaglutide_medium_dosage | 0 | 0.11227123 | NA | 0.11227123 | NA | NA | NA | NA |
| 74 | Canagliflozin_high_dosage:Liraglutide | 0 | 0.353720182 | NA | 0.353720182 | NA | NA | NA | NA |
| 75 | Canagliflozin_high_dosage:Lixisenatide | 0 | 0.177563794 | NA | 0.177563794 | NA | NA | NA | NA |
| 76 | Canagliflozin_high_dosage:Oral_semaglutide | 0 | 1.14005851 | NA | 1.14005851 | NA | NA | NA | NA |
| 77 | Canagliflozin_high_dosage:Placebo_or_Control | 4 | 0.600270709 | 0.450957424 | 1.897353768 | -1.446396344 | -3.933370282 | 1.040577593 | 0.254330788 |
| 78 | Canagliflozin_high_dosage:Sotagliflozin | 0 | 0.941423734 | NA | 0.941423734 | NA | NA | NA | NA |
| 79 | Canagliflozin_high_dosage:Tirzepatide_high_dosage | 0 | 0.298977522 | NA | 0.298977522 | NA | NA | NA | NA |
| 80 | Canagliflozin_high_dosage:Tirzepatide_low_dosage | 0 | 0.402909111 | NA | 0.402909111 | NA | NA | NA | NA |
| 81 | Canagliflozin_high_dosage:Tirzepatide_medium_dosage | 0 | -0.018463279 | NA | -0.018463279 | NA | NA | NA | NA |
| 82 | Canagliflozin_low_dosage:Dapagliflozin_high_dosage | 0 | 0.597846314 | NA | 0.597846314 | NA | NA | NA | NA |
| 83 | Canagliflozin_low_dosage:Dapagliflozin_low_dosage | 0 | -0.009059835 | NA | -0.009059835 | NA | NA | NA | NA |
| 84 | Canagliflozin_low_dosage:Dapagliflozin_medium_dosage | 0 | 1.452862912 | NA | 1.452862912 | NA | NA | NA | NA |
| 85 | Canagliflozin_low_dosage:Dulaglutide_high_dosage | 0 | -0.122017164 | NA | -0.122017164 | NA | NA | NA | NA |
| 86 | Canagliflozin_low_dosage:Dulaglutide_low_dosage | 0 | 0.60391984 | NA | 0.60391984 | NA | NA | NA | NA |
| 87 | Canagliflozin_low_dosage:Dulaglutide_medium_dosage | 0 | 0.576583138 | NA | 0.576583138 | NA | NA | NA | NA |
| 88 | Canagliflozin_low_dosage:Efpeglenatide_high_dosage | 0 | -0.896485581 | NA | -0.896485581 | NA | NA | NA | NA |
| 89 | Canagliflozin_low_dosage:Efpeglenatide_low_dosage | 0 | -0.799075465 | NA | -0.799075465 | NA | NA | NA | NA |
| 90 | Canagliflozin_low_dosage:Efpeglenatide_medium_dosage | 0 | 0.409267452 | NA | 0.409267452 | NA | NA | NA | NA |
| 91 | Canagliflozin_low_dosage:Empagliflozin_high_dosage | 0 | 0.570487264 | NA | 0.570487264 | NA | NA | NA | NA |
| 92 | Canagliflozin_low_dosage:Empagliflozin_low_dosage | 0 | 0.627617005 | NA | 0.627617005 | NA | NA | NA | NA |
| 93 | Canagliflozin_low_dosage:Ertugliflozin_high_dosage | 0 | -0.22354173 | NA | -0.22354173 | NA | NA | NA | NA |
| 94 | Canagliflozin_low_dosage:Ertugliflozin_low_dosage | 0 | 0.348940727 | NA | 0.348940727 | NA | NA | NA | NA |
| 95 | Canagliflozin_low_dosage:Exenatide | 0 | 0.126135596 | NA | 0.126135596 | NA | NA | NA | NA |
| 96 | Canagliflozin_low_dosage:Inject_semaglutide_high_dosage | 0 | 0.518334817 | NA | 0.518334817 | NA | NA | NA | NA |
| 97 | Canagliflozin_low_dosage:Inject_semaglutide_low_dosage | 0 | 0.922365094 | NA | 0.922365094 | NA | NA | NA | NA |
| 98 | Canagliflozin_low_dosage:Inject_semaglutide_medium_dosage | 0 | -0.084210777 | NA | -0.084210777 | NA | NA | NA | NA |
| 99 | Canagliflozin_low_dosage:Liraglutide | 0 | 0.157238174 | NA | 0.157238174 | NA | NA | NA | NA |
| 100 | Canagliflozin_low_dosage:Lixisenatide | 0 | -0.018918213 | NA | -0.018918213 | NA | NA | NA | NA |
| 101 | Canagliflozin_low_dosage:Oral_semaglutide | 0 | 0.943576503 | NA | 0.943576503 | NA | NA | NA | NA |
| 102 | Canagliflozin_low_dosage:Placebo_or_Control | 2 | 0.403788701 | 0.460763383 | 0.095325021 | 0.365438362 | -1.903817668 | 2.634694392 | 0.752283114 |
| 103 | Canagliflozin_low_dosage:Sotagliflozin | 0 | 0.744941726 | NA | 0.744941726 | NA | NA | NA | NA |
| 104 | Canagliflozin_low_dosage:Tirzepatide_high_dosage | 0 | 0.102495515 | NA | 0.102495515 | NA | NA | NA | NA |
| 105 | Canagliflozin_low_dosage:Tirzepatide_low_dosage | 0 | 0.206427104 | NA | 0.206427104 | NA | NA | NA | NA |
| 106 | Canagliflozin_low_dosage:Tirzepatide_medium_dosage | 0 | -0.214945286 | NA | -0.214945286 | NA | NA | NA | NA |
| 107 | Dapagliflozin_high_dosage:Dapagliflozin_low_dosage | 3 | -0.606906149 | -0.432798864 | -0.807132991 | 0.374334128 | -2.348289614 | 3.096957869 | 0.787563507 |
| 108 | Dapagliflozin_high_dosage:Dapagliflozin_medium_dosage | 1 | 0.855016597 | 1.273661086 | 0.561887239 | 0.711773847 | -3.486494749 | 4.910042444 | 0.739668773 |
| 109 | Dapagliflozin_high_dosage:Dulaglutide_high_dosage | 0 | -0.719863478 | NA | -0.719863478 | NA | NA | NA | NA |
| 110 | Dapagliflozin_high_dosage:Dulaglutide_low_dosage | 0 | 0.006073526 | NA | 0.006073526 | NA | NA | NA | NA |
| 111 | Dapagliflozin_high_dosage:Dulaglutide_medium_dosage | 0 | -0.021263176 | NA | -0.021263176 | NA | NA | NA | NA |
| 112 | Dapagliflozin_high_dosage:Efpeglenatide_high_dosage | 0 | -1.494331895 | NA | -1.494331895 | NA | NA | NA | NA |
| 113 | Dapagliflozin_high_dosage:Efpeglenatide_low_dosage | 0 | -1.396921779 | NA | -1.396921779 | NA | NA | NA | NA |
| 114 | Dapagliflozin_high_dosage:Efpeglenatide_medium_dosage | 0 | -0.188578862 | NA | -0.188578862 | NA | NA | NA | NA |
| 115 | Dapagliflozin_high_dosage:Empagliflozin_high_dosage | 0 | -0.027359051 | NA | -0.027359051 | NA | NA | NA | NA |
| 116 | Dapagliflozin_high_dosage:Empagliflozin_low_dosage | 0 | 0.029770691 | NA | 0.029770691 | NA | NA | NA | NA |
| 117 | Dapagliflozin_high_dosage:Ertugliflozin_high_dosage | 0 | -0.821388044 | NA | -0.821388044 | NA | NA | NA | NA |
| 118 | Dapagliflozin_high_dosage:Ertugliflozin_low_dosage | 0 | -0.248905587 | NA | -0.248905587 | NA | NA | NA | NA |
| 119 | Dapagliflozin_high_dosage:Exenatide | 0 | -0.471710718 | NA | -0.471710718 | NA | NA | NA | NA |
| 120 | Dapagliflozin_high_dosage:Inject_semaglutide_high_dosage | 0 | -0.079511498 | NA | -0.079511498 | NA | NA | NA | NA |
| 121 | Dapagliflozin_high_dosage:Inject_semaglutide_low_dosage | 0 | 0.32451878 | NA | 0.32451878 | NA | NA | NA | NA |
| 122 | Dapagliflozin_high_dosage:Inject_semaglutide_medium_dosage | 0 | -0.682057092 | NA | -0.682057092 | NA | NA | NA | NA |
| 123 | Dapagliflozin_high_dosage:Liraglutide | 0 | -0.44060814 | NA | -0.44060814 | NA | NA | NA | NA |
| 124 | Dapagliflozin_high_dosage:Lixisenatide | 0 | -0.616764528 | NA | -0.616764528 | NA | NA | NA | NA |
| 125 | Dapagliflozin_high_dosage:Oral_semaglutide | 0 | 0.345730189 | NA | 0.345730189 | NA | NA | NA | NA |
| 126 | Dapagliflozin_high_dosage:Placebo_or_Control | 7 | -0.194057613 | -0.207402277 | 0.532873739 | -0.740276015 | -3.59429493 | 2.1137429 | 0.611189777 |
| 127 | Dapagliflozin_high_dosage:Sotagliflozin | 0 | 0.147095412 | NA | 0.147095412 | NA | NA | NA | NA |
| 128 | Dapagliflozin_high_dosage:Tirzepatide_high_dosage | 0 | -0.495350799 | NA | -0.495350799 | NA | NA | NA | NA |
| 129 | Dapagliflozin_high_dosage:Tirzepatide_low_dosage | 0 | -0.39141921 | NA | -0.39141921 | NA | NA | NA | NA |
| 130 | Dapagliflozin_high_dosage:Tirzepatide_medium_dosage | 0 | -0.8127916 | NA | -0.8127916 | NA | NA | NA | NA |
| 131 | Dapagliflozin_low_dosage:Dapagliflozin_medium_dosage | 2 | 1.461922746 | 1.253299094 | 1.965072941 | -0.711773847 | -4.910042444 | 3.486494749 | 0.739668773 |
| 132 | Dapagliflozin_low_dosage:Dulaglutide_high_dosage | 0 | -0.112957329 | NA | -0.112957329 | NA | NA | NA | NA |
| 133 | Dapagliflozin_low_dosage:Dulaglutide_low_dosage | 0 | 0.612979674 | NA | 0.612979674 | NA | NA | NA | NA |
| 134 | Dapagliflozin_low_dosage:Dulaglutide_medium_dosage | 0 | 0.585642973 | NA | 0.585642973 | NA | NA | NA | NA |
| 135 | Dapagliflozin_low_dosage:Efpeglenatide_high_dosage | 0 | -0.887425746 | NA | -0.887425746 | NA | NA | NA | NA |
| 136 | Dapagliflozin_low_dosage:Efpeglenatide_low_dosage | 0 | -0.79001563 | NA | -0.79001563 | NA | NA | NA | NA |
| 137 | Dapagliflozin_low_dosage:Efpeglenatide_medium_dosage | 0 | 0.418327287 | NA | 0.418327287 | NA | NA | NA | NA |
| 138 | Dapagliflozin_low_dosage:Empagliflozin_high_dosage | 0 | 0.579547098 | NA | 0.579547098 | NA | NA | NA | NA |
| 139 | Dapagliflozin_low_dosage:Empagliflozin_low_dosage | 0 | 0.63667684 | NA | 0.63667684 | NA | NA | NA | NA |
| 140 | Dapagliflozin_low_dosage:Ertugliflozin_high_dosage | 0 | -0.214481895 | NA | -0.214481895 | NA | NA | NA | NA |
| 141 | Dapagliflozin_low_dosage:Ertugliflozin_low_dosage | 0 | 0.358000562 | NA | 0.358000562 | NA | NA | NA | NA |
| 142 | Dapagliflozin_low_dosage:Exenatide | 0 | 0.135195431 | NA | 0.135195431 | NA | NA | NA | NA |
| 143 | Dapagliflozin_low_dosage:Inject_semaglutide_high_dosage | 0 | 0.527394651 | NA | 0.527394651 | NA | NA | NA | NA |
| 144 | Dapagliflozin_low_dosage:Inject_semaglutide_low_dosage | 0 | 0.931424929 | NA | 0.931424929 | NA | NA | NA | NA |
| 145 | Dapagliflozin_low_dosage:Inject_semaglutide_medium_dosage | 0 | -0.075150943 | NA | -0.075150943 | NA | NA | NA | NA |
| 146 | Dapagliflozin_low_dosage:Liraglutide | 0 | 0.166298009 | NA | 0.166298009 | NA | NA | NA | NA |
| 147 | Dapagliflozin_low_dosage:Lixisenatide | 0 | -0.009858379 | NA | -0.009858379 | NA | NA | NA | NA |
| 148 | Dapagliflozin_low_dosage:Oral_semaglutide | 0 | 0.952636338 | NA | 0.952636338 | NA | NA | NA | NA |
| 149 | Dapagliflozin_low_dosage:Placebo_or_Control | 2 | 0.412848536 | 0.883358288 | 0.143082273 | 0.740276015 | -2.1137429 | 3.59429493 | 0.611189777 |
| 150 | Dapagliflozin_low_dosage:Sotagliflozin | 0 | 0.754001561 | NA | 0.754001561 | NA | NA | NA | NA |
| 151 | Dapagliflozin_low_dosage:Tirzepatide_high_dosage | 0 | 0.11155535 | NA | 0.11155535 | NA | NA | NA | NA |
| 152 | Dapagliflozin_low_dosage:Tirzepatide_low_dosage | 0 | 0.215486939 | NA | 0.215486939 | NA | NA | NA | NA |
| 153 | Dapagliflozin_low_dosage:Tirzepatide_medium_dosage | 0 | -0.205885451 | NA | -0.205885451 | NA | NA | NA | NA |
| 154 | Dapagliflozin_medium_dosage:Dulaglutide_high_dosage | 0 | -1.574880075 | NA | -1.574880075 | NA | NA | NA | NA |
| 155 | Dapagliflozin_medium_dosage:Dulaglutide_low_dosage | 0 | -0.848943072 | NA | -0.848943072 | NA | NA | NA | NA |
| 156 | Dapagliflozin_medium_dosage:Dulaglutide_medium_dosage | 0 | -0.876279773 | NA | -0.876279773 | NA | NA | NA | NA |
| 157 | Dapagliflozin_medium_dosage:Efpeglenatide_high_dosage | 0 | -2.349348492 | NA | -2.349348492 | NA | NA | NA | NA |
| 158 | Dapagliflozin_medium_dosage:Efpeglenatide_low_dosage | 0 | -2.251938376 | NA | -2.251938376 | NA | NA | NA | NA |
| 159 | Dapagliflozin_medium_dosage:Efpeglenatide_medium_dosage | 0 | -1.04359546 | NA | -1.04359546 | NA | NA | NA | NA |
| 160 | Dapagliflozin_medium_dosage:Empagliflozin_high_dosage | 0 | -0.882375648 | NA | -0.882375648 | NA | NA | NA | NA |
| 161 | Dapagliflozin_medium_dosage:Empagliflozin_low_dosage | 0 | -0.825245907 | NA | -0.825245907 | NA | NA | NA | NA |
| 162 | Dapagliflozin_medium_dosage:Ertugliflozin_high_dosage | 0 | -1.676404641 | NA | -1.676404641 | NA | NA | NA | NA |
| 163 | Dapagliflozin_medium_dosage:Ertugliflozin_low_dosage | 0 | -1.103922184 | NA | -1.103922184 | NA | NA | NA | NA |
| 164 | Dapagliflozin_medium_dosage:Exenatide | 0 | -1.326727315 | NA | -1.326727315 | NA | NA | NA | NA |
| 165 | Dapagliflozin_medium_dosage:Inject_semaglutide_high_dosage | 0 | -0.934528095 | NA | -0.934528095 | NA | NA | NA | NA |
| 166 | Dapagliflozin_medium_dosage:Inject_semaglutide_low_dosage | 0 | -0.530497818 | NA | -0.530497818 | NA | NA | NA | NA |
| 167 | Dapagliflozin_medium_dosage:Inject_semaglutide_medium_dosage | 0 | -1.537073689 | NA | -1.537073689 | NA | NA | NA | NA |
| 168 | Dapagliflozin_medium_dosage:Liraglutide | 0 | -1.295624737 | NA | -1.295624737 | NA | NA | NA | NA |
| 169 | Dapagliflozin_medium_dosage:Lixisenatide | 0 | -1.471781125 | NA | -1.471781125 | NA | NA | NA | NA |
| 170 | Dapagliflozin_medium_dosage:Oral_semaglutide | 0 | -0.509286409 | NA | -0.509286409 | NA | NA | NA | NA |
| 171 | Dapagliflozin_medium_dosage:Placebo_or_Control | 0 | -1.04907421 | NA | -1.04907421 | NA | NA | NA | NA |
| 172 | Dapagliflozin_medium_dosage:Sotagliflozin | 0 | -0.707921185 | NA | -0.707921185 | NA | NA | NA | NA |
| 173 | Dapagliflozin_medium_dosage:Tirzepatide_high_dosage | 0 | -1.350367397 | NA | -1.350367397 | NA | NA | NA | NA |
| 174 | Dapagliflozin_medium_dosage:Tirzepatide_low_dosage | 0 | -1.246435808 | NA | -1.246435808 | NA | NA | NA | NA |
| 175 | Dapagliflozin_medium_dosage:Tirzepatide_medium_dosage | 0 | -1.667808198 | NA | -1.667808198 | NA | NA | NA | NA |
| 176 | Dulaglutide_high_dosage:Dulaglutide_low_dosage | 1 | 0.725937003 | 0.725937003 | NA | NA | NA | NA | NA |
| 177 | Dulaglutide_high_dosage:Dulaglutide_medium_dosage | 0 | 0.698600302 | NA | 0.698600302 | NA | NA | NA | NA |
| 178 | Dulaglutide_high_dosage:Efpeglenatide_high_dosage | 0 | -0.774468417 | NA | -0.774468417 | NA | NA | NA | NA |
| 179 | Dulaglutide_high_dosage:Efpeglenatide_low_dosage | 0 | -0.677058301 | NA | -0.677058301 | NA | NA | NA | NA |
| 180 | Dulaglutide_high_dosage:Efpeglenatide_medium_dosage | 0 | 0.531284616 | NA | 0.531284616 | NA | NA | NA | NA |
| 181 | Dulaglutide_high_dosage:Empagliflozin_high_dosage | 0 | 0.692504427 | NA | 0.692504427 | NA | NA | NA | NA |
| 182 | Dulaglutide_high_dosage:Empagliflozin_low_dosage | 0 | 0.749634168 | NA | 0.749634168 | NA | NA | NA | NA |
| 183 | Dulaglutide_high_dosage:Ertugliflozin_high_dosage | 0 | -0.101524566 | NA | -0.101524566 | NA | NA | NA | NA |
| 184 | Dulaglutide_high_dosage:Ertugliflozin_low_dosage | 0 | 0.470957891 | NA | 0.470957891 | NA | NA | NA | NA |
| 185 | Dulaglutide_high_dosage:Exenatide | 0 | 0.24815276 | NA | 0.24815276 | NA | NA | NA | NA |
| 186 | Dulaglutide_high_dosage:Inject_semaglutide_high_dosage | 0 | 0.64035198 | NA | 0.64035198 | NA | NA | NA | NA |
| 187 | Dulaglutide_high_dosage:Inject_semaglutide_low_dosage | 0 | 1.044382257 | NA | 1.044382257 | NA | NA | NA | NA |
| 188 | Dulaglutide_high_dosage:Inject_semaglutide_medium_dosage | 0 | 0.037806386 | NA | 0.037806386 | NA | NA | NA | NA |
| 189 | Dulaglutide_high_dosage:Liraglutide | 0 | 0.279255338 | NA | 0.279255338 | NA | NA | NA | NA |
| 190 | Dulaglutide_high_dosage:Lixisenatide | 0 | 0.10309895 | NA | 0.10309895 | NA | NA | NA | NA |
| 191 | Dulaglutide_high_dosage:Oral_semaglutide | 0 | 1.065593667 | NA | 1.065593667 | NA | NA | NA | NA |
| 192 | Dulaglutide_high_dosage:Placebo_or_Control | 0 | 0.525805865 | NA | 0.525805865 | NA | NA | NA | NA |
| 193 | Dulaglutide_high_dosage:Sotagliflozin | 0 | 0.86695889 | NA | 0.86695889 | NA | NA | NA | NA |
| 194 | Dulaglutide_high_dosage:Tirzepatide_high_dosage | 0 | 0.224512678 | NA | 0.224512678 | NA | NA | NA | NA |
| 195 | Dulaglutide_high_dosage:Tirzepatide_low_dosage | 0 | 0.328444267 | NA | 0.328444267 | NA | NA | NA | NA |
| 196 | Dulaglutide_high_dosage:Tirzepatide_medium_dosage | 0 | -0.092928122 | NA | -0.092928122 | NA | NA | NA | NA |
| 197 | Dulaglutide_low_dosage:Dulaglutide_medium_dosage | 3 | -0.027336702 | 0.2952863 | -0.433126132 | 0.728412432 | -2.055811305 | 3.512636169 | 0.608113291 |
| 198 | Dulaglutide_low_dosage:Efpeglenatide_high_dosage | 0 | -1.500405421 | NA | -1.500405421 | NA | NA | NA | NA |
| 199 | Dulaglutide_low_dosage:Efpeglenatide_low_dosage | 0 | -1.402995305 | NA | -1.402995305 | NA | NA | NA | NA |
| 200 | Dulaglutide_low_dosage:Efpeglenatide_medium_dosage | 0 | -0.194652388 | NA | -0.194652388 | NA | NA | NA | NA |
| 201 | Dulaglutide_low_dosage:Empagliflozin_high_dosage | 0 | -0.033432576 | NA | -0.033432576 | NA | NA | NA | NA |
| 202 | Dulaglutide_low_dosage:Empagliflozin_low_dosage | 0 | 0.023697165 | NA | 0.023697165 | NA | NA | NA | NA |
| 203 | Dulaglutide_low_dosage:Ertugliflozin_high_dosage | 0 | -0.827461569 | NA | -0.827461569 | NA | NA | NA | NA |
| 204 | Dulaglutide_low_dosage:Ertugliflozin_low_dosage | 0 | -0.254979113 | NA | -0.254979113 | NA | NA | NA | NA |
| 205 | Dulaglutide_low_dosage:Exenatide | 0 | -0.477784244 | NA | -0.477784244 | NA | NA | NA | NA |
| 206 | Dulaglutide_low_dosage:Inject_semaglutide_high_dosage | 0 | -0.085585023 | NA | -0.085585023 | NA | NA | NA | NA |
| 207 | Dulaglutide_low_dosage:Inject_semaglutide_low_dosage | 0 | 0.318445254 | NA | 0.318445254 | NA | NA | NA | NA |
| 208 | Dulaglutide_low_dosage:Inject_semaglutide_medium_dosage | 0 | -0.688130617 | NA | -0.688130617 | NA | NA | NA | NA |
| 209 | Dulaglutide_low_dosage:Liraglutide | 0 | -0.446681666 | NA | -0.446681666 | NA | NA | NA | NA |
| 210 | Dulaglutide_low_dosage:Lixisenatide | 0 | -0.622838053 | NA | -0.622838053 | NA | NA | NA | NA |
| 211 | Dulaglutide_low_dosage:Oral_semaglutide | 0 | 0.339656663 | NA | 0.339656663 | NA | NA | NA | NA |
| 212 | Dulaglutide_low_dosage:Placebo_or_Control | 2 | -0.200131139 | -0.337009669 | 0.036348144 | -0.373357812 | -3.215717205 | 2.469001581 | 0.796830746 |
| 213 | Dulaglutide_low_dosage:Sotagliflozin | 0 | 0.141021887 | NA | 0.141021887 | NA | NA | NA | NA |
| 214 | Dulaglutide_low_dosage:Tirzepatide_high_dosage | 0 | -0.501424325 | NA | -0.501424325 | NA | NA | NA | NA |
| 215 | Dulaglutide_low_dosage:Tirzepatide_low_dosage | 0 | -0.397492736 | NA | -0.397492736 | NA | NA | NA | NA |
| 216 | Dulaglutide_low_dosage:Tirzepatide_medium_dosage | 0 | -0.818865126 | NA | -0.818865126 | NA | NA | NA | NA |
| 217 | Dulaglutide_medium_dosage:Efpeglenatide_high_dosage | 0 | -1.473068719 | NA | -1.473068719 | NA | NA | NA | NA |
| 218 | Dulaglutide_medium_dosage:Efpeglenatide_low_dosage | 0 | -1.375658603 | NA | -1.375658603 | NA | NA | NA | NA |
| 219 | Dulaglutide_medium_dosage:Efpeglenatide_medium_dosage | 0 | -0.167315686 | NA | -0.167315686 | NA | NA | NA | NA |
| 220 | Dulaglutide_medium_dosage:Empagliflozin_high_dosage | 0 | -0.006095875 | NA | -0.006095875 | NA | NA | NA | NA |
| 221 | Dulaglutide_medium_dosage:Empagliflozin_low_dosage | 0 | 0.051033867 | NA | 0.051033867 | NA | NA | NA | NA |
| 222 | Dulaglutide_medium_dosage:Ertugliflozin_high_dosage | 0 | -0.800124868 | NA | -0.800124868 | NA | NA | NA | NA |
| 223 | Dulaglutide_medium_dosage:Ertugliflozin_low_dosage | 0 | -0.227642411 | NA | -0.227642411 | NA | NA | NA | NA |
| 224 | Dulaglutide_medium_dosage:Exenatide | 0 | -0.450447542 | NA | -0.450447542 | NA | NA | NA | NA |
| 225 | Dulaglutide_medium_dosage:Inject_semaglutide_high_dosage | 0 | -0.058248321 | NA | -0.058248321 | NA | NA | NA | NA |
| 226 | Dulaglutide_medium_dosage:Inject_semaglutide_low_dosage | 0 | 0.345781956 | NA | 0.345781956 | NA | NA | NA | NA |
| 227 | Dulaglutide_medium_dosage:Inject_semaglutide_medium_dosage | 0 | -0.660793916 | NA | -0.660793916 | NA | NA | NA | NA |
| 228 | Dulaglutide_medium_dosage:Liraglutide | 0 | -0.419344964 | NA | -0.419344964 | NA | NA | NA | NA |
| 229 | Dulaglutide_medium_dosage:Lixisenatide | 0 | -0.595501352 | NA | -0.595501352 | NA | NA | NA | NA |
| 230 | Dulaglutide_medium_dosage:Oral_semaglutide | 0 | 0.366993365 | NA | 0.366993365 | NA | NA | NA | NA |
| 231 | Dulaglutide_medium_dosage:Placebo_or_Control | 3 | -0.172794437 | -0.177523648 | -0.003346139 | -0.17417751 | -3.403828213 | 3.055473194 | 0.915818522 |
| 232 | Dulaglutide_medium_dosage:Sotagliflozin | 0 | 0.168358588 | NA | 0.168358588 | NA | NA | NA | NA |
| 233 | Dulaglutide_medium_dosage:Tirzepatide_high_dosage | 0 | -0.474087623 | NA | -0.474087623 | NA | NA | NA | NA |
| 234 | Dulaglutide_medium_dosage:Tirzepatide_low_dosage | 0 | -0.370156034 | NA | -0.370156034 | NA | NA | NA | NA |
| 235 | Dulaglutide_medium_dosage:Tirzepatide_medium_dosage | 0 | -0.791528424 | NA | -0.791528424 | NA | NA | NA | NA |
| 236 | Efpeglenatide_high_dosage:Efpeglenatide_low_dosage | 2 | 0.097410116 | 0.044665382 | 0.160275829 | -0.115610447 | -2.997938991 | 2.766718097 | 0.937339393 |
| 237 | Efpeglenatide_high_dosage:Efpeglenatide_medium_dosage | 2 | 1.305753033 | 1.295455347 | 1.331262355 | -0.035807008 | -3.434211898 | 3.362597882 | 0.98352406 |
| 238 | Efpeglenatide_high_dosage:Empagliflozin_high_dosage | 0 | 1.466972844 | NA | 1.466972844 | NA | NA | NA | NA |
| 239 | Efpeglenatide_high_dosage:Empagliflozin_low_dosage | 0 | 1.524102586 | NA | 1.524102586 | NA | NA | NA | NA |
| 240 | Efpeglenatide_high_dosage:Ertugliflozin_high_dosage | 0 | 0.672943851 | NA | 0.672943851 | NA | NA | NA | NA |
| 241 | Efpeglenatide_high_dosage:Ertugliflozin_low_dosage | 0 | 1.245426308 | NA | 1.245426308 | NA | NA | NA | NA |
| 242 | Efpeglenatide_high_dosage:Exenatide | 0 | 1.022621177 | NA | 1.022621177 | NA | NA | NA | NA |
| 243 | Efpeglenatide_high_dosage:Inject_semaglutide_high_dosage | 0 | 1.414820398 | NA | 1.414820398 | NA | NA | NA | NA |
| 244 | Efpeglenatide_high_dosage:Inject_semaglutide_low_dosage | 0 | 1.818850675 | NA | 1.818850675 | NA | NA | NA | NA |
| 245 | Efpeglenatide_high_dosage:Inject_semaglutide_medium_dosage | 0 | 0.812274803 | NA | 0.812274803 | NA | NA | NA | NA |
| 246 | Efpeglenatide_high_dosage:Liraglutide | 0 | 1.053723755 | NA | 1.053723755 | NA | NA | NA | NA |
| 247 | Efpeglenatide_high_dosage:Lixisenatide | 0 | 0.877567368 | NA | 0.877567368 | NA | NA | NA | NA |
| 248 | Efpeglenatide_high_dosage:Oral_semaglutide | 0 | 1.840062084 | NA | 1.840062084 | NA | NA | NA | NA |
| 249 | Efpeglenatide_high_dosage:Placebo_or_Control | 2 | 1.300274282 | 1.274926002 | 1.36305967 | -0.088133667 | -3.486311087 | 3.310043753 | 0.959458768 |
| 250 | Efpeglenatide_high_dosage:Sotagliflozin | 0 | 1.641427307 | NA | 1.641427307 | NA | NA | NA | NA |
| 251 | Efpeglenatide_high_dosage:Tirzepatide_high_dosage | 0 | 0.998981096 | NA | 0.998981096 | NA | NA | NA | NA |
| 252 | Efpeglenatide_high_dosage:Tirzepatide_low_dosage | 0 | 1.102912685 | NA | 1.102912685 | NA | NA | NA | NA |
| 253 | Efpeglenatide_high_dosage:Tirzepatide_medium_dosage | 0 | 0.681540295 | NA | 0.681540295 | NA | NA | NA | NA |
| 254 | Efpeglenatide_low_dosage:Efpeglenatide_medium_dosage | 2 | 1.208342917 | 1.152020811 | 1.268062151 | -0.116041341 | -3.395566592 | 3.163483911 | 0.944710597 |
| 255 | Efpeglenatide_low_dosage:Empagliflozin_high_dosage | 0 | 1.369562728 | NA | 1.369562728 | NA | NA | NA | NA |
| 256 | Efpeglenatide_low_dosage:Empagliflozin_low_dosage | 0 | 1.42669247 | NA | 1.42669247 | NA | NA | NA | NA |
| 257 | Efpeglenatide_low_dosage:Ertugliflozin_high_dosage | 0 | 0.575533735 | NA | 0.575533735 | NA | NA | NA | NA |
| 258 | Efpeglenatide_low_dosage:Ertugliflozin_low_dosage | 0 | 1.148016192 | NA | 1.148016192 | NA | NA | NA | NA |
| 259 | Efpeglenatide_low_dosage:Exenatide | 0 | 0.925211061 | NA | 0.925211061 | NA | NA | NA | NA |
| 260 | Efpeglenatide_low_dosage:Inject_semaglutide_high_dosage | 0 | 1.317410282 | NA | 1.317410282 | NA | NA | NA | NA |
| 261 | Efpeglenatide_low_dosage:Inject_semaglutide_low_dosage | 0 | 1.721440559 | NA | 1.721440559 | NA | NA | NA | NA |
| 262 | Efpeglenatide_low_dosage:Inject_semaglutide_medium_dosage | 0 | 0.714864687 | NA | 0.714864687 | NA | NA | NA | NA |
| 263 | Efpeglenatide_low_dosage:Liraglutide | 0 | 0.956313639 | NA | 0.956313639 | NA | NA | NA | NA |
| 264 | Efpeglenatide_low_dosage:Lixisenatide | 0 | 0.780157251 | NA | 0.780157251 | NA | NA | NA | NA |
| 265 | Efpeglenatide_low_dosage:Oral_semaglutide | 0 | 1.742651968 | NA | 1.742651968 | NA | NA | NA | NA |
| 266 | Efpeglenatide_low_dosage:Placebo_or_Control | 2 | 1.202864166 | 1.18654748 | 1.220170455 | -0.033622975 | -3.31291618 | 3.245670229 | 0.983966999 |
| 267 | Efpeglenatide_low_dosage:Sotagliflozin | 0 | 1.544017191 | NA | 1.544017191 | NA | NA | NA | NA |
| 268 | Efpeglenatide_low_dosage:Tirzepatide_high_dosage | 0 | 0.90157098 | NA | 0.90157098 | NA | NA | NA | NA |
| 269 | Efpeglenatide_low_dosage:Tirzepatide_low_dosage | 0 | 1.005502569 | NA | 1.005502569 | NA | NA | NA | NA |
| 270 | Efpeglenatide_low_dosage:Tirzepatide_medium_dosage | 0 | 0.584130179 | NA | 0.584130179 | NA | NA | NA | NA |
| 271 | Efpeglenatide_medium_dosage:Empagliflozin_high_dosage | 0 | 0.161219811 | NA | 0.161219811 | NA | NA | NA | NA |
| 272 | Efpeglenatide_medium_dosage:Empagliflozin_low_dosage | 0 | 0.218349553 | NA | 0.218349553 | NA | NA | NA | NA |
| 273 | Efpeglenatide_medium_dosage:Ertugliflozin_high_dosage | 0 | -0.632809182 | NA | -0.632809182 | NA | NA | NA | NA |
| 274 | Efpeglenatide_medium_dosage:Ertugliflozin_low_dosage | 0 | -0.060326725 | NA | -0.060326725 | NA | NA | NA | NA |
| 275 | Efpeglenatide_medium_dosage:Exenatide | 0 | -0.283131856 | NA | -0.283131856 | NA | NA | NA | NA |
| 276 | Efpeglenatide_medium_dosage:Inject_semaglutide_high_dosage | 0 | 0.109067365 | NA | 0.109067365 | NA | NA | NA | NA |
| 277 | Efpeglenatide_medium_dosage:Inject_semaglutide_low_dosage | 0 | 0.513097642 | NA | 0.513097642 | NA | NA | NA | NA |
| 278 | Efpeglenatide_medium_dosage:Inject_semaglutide_medium_dosage | 0 | -0.49347823 | NA | -0.49347823 | NA | NA | NA | NA |
| 279 | Efpeglenatide_medium_dosage:Liraglutide | 0 | -0.252029278 | NA | -0.252029278 | NA | NA | NA | NA |
| 280 | Efpeglenatide_medium_dosage:Lixisenatide | 0 | -0.428185665 | NA | -0.428185665 | NA | NA | NA | NA |
| 281 | Efpeglenatide_medium_dosage:Oral_semaglutide | 0 | 0.534309051 | NA | 0.534309051 | NA | NA | NA | NA |
| 282 | Efpeglenatide_medium_dosage:Placebo_or_Control | 1 | -0.005478751 | -0.053563443 | 0.036416252 | -0.089979695 | -3.886076538 | 3.706117148 | 0.962945651 |
| 283 | Efpeglenatide_medium_dosage:Sotagliflozin | 0 | 0.335674274 | NA | 0.335674274 | NA | NA | NA | NA |
| 284 | Efpeglenatide_medium_dosage:Tirzepatide_high_dosage | 0 | -0.306771937 | NA | -0.306771937 | NA | NA | NA | NA |
| 285 | Efpeglenatide_medium_dosage:Tirzepatide_low_dosage | 0 | -0.202840348 | NA | -0.202840348 | NA | NA | NA | NA |
| 286 | Efpeglenatide_medium_dosage:Tirzepatide_medium_dosage | 0 | -0.624212738 | NA | -0.624212738 | NA | NA | NA | NA |
| 287 | Empagliflozin_high_dosage:Empagliflozin_low_dosage | 5 | 0.057129741 | -0.086610242 | 0.279108952 | -0.365719194 | -2.27682395 | 1.545385563 | 0.707609035 |
| 288 | Empagliflozin_high_dosage:Ertugliflozin_high_dosage | 0 | -0.794028993 | NA | -0.794028993 | NA | NA | NA | NA |
| 289 | Empagliflozin_high_dosage:Ertugliflozin_low_dosage | 0 | -0.221546536 | NA | -0.221546536 | NA | NA | NA | NA |
| 290 | Empagliflozin_high_dosage:Exenatide | 0 | -0.444351667 | NA | -0.444351667 | NA | NA | NA | NA |
| 291 | Empagliflozin_high_dosage:Inject_semaglutide_high_dosage | 0 | -0.052152447 | NA | -0.052152447 | NA | NA | NA | NA |
| 292 | Empagliflozin_high_dosage:Inject_semaglutide_low_dosage | 0 | 0.35187783 | NA | 0.35187783 | NA | NA | NA | NA |
| 293 | Empagliflozin_high_dosage:Inject_semaglutide_medium_dosage | 0 | -0.654698041 | NA | -0.654698041 | NA | NA | NA | NA |
| 294 | Empagliflozin_high_dosage:Liraglutide | 0 | -0.413249089 | NA | -0.413249089 | NA | NA | NA | NA |
| 295 | Empagliflozin_high_dosage:Lixisenatide | 0 | -0.589405477 | NA | -0.589405477 | NA | NA | NA | NA |
| 296 | Empagliflozin_high_dosage:Oral_semaglutide | 1 | 0.373089239 | 0.019802627 | 0.465439634 | -0.445637006 | -3.566412751 | 2.675138739 | 0.779572088 |
| 297 | Empagliflozin_high_dosage:Placebo_or_Control | 5 | -0.166698562 | -0.003003897 | -0.50495098 | 0.501947084 | -1.396755562 | 2.40064973 | 0.604358971 |
| 298 | Empagliflozin_high_dosage:Sotagliflozin | 0 | 0.174454463 | NA | 0.174454463 | NA | NA | NA | NA |
| 299 | Empagliflozin_high_dosage:Tirzepatide_high_dosage | 0 | -0.467991749 | NA | -0.467991749 | NA | NA | NA | NA |
| 300 | Empagliflozin_high_dosage:Tirzepatide_low_dosage | 0 | -0.36406016 | NA | -0.36406016 | NA | NA | NA | NA |
| 301 | Empagliflozin_high_dosage:Tirzepatide_medium_dosage | 0 | -0.78543255 | NA | -0.78543255 | NA | NA | NA | NA |
| 302 | Empagliflozin_low_dosage:Ertugliflozin_high_dosage | 0 | -0.851158735 | NA | -0.851158735 | NA | NA | NA | NA |
| 303 | Empagliflozin_low_dosage:Ertugliflozin_low_dosage | 0 | -0.278676278 | NA | -0.278676278 | NA | NA | NA | NA |
| 304 | Empagliflozin_low_dosage:Exenatide | 0 | -0.501481409 | NA | -0.501481409 | NA | NA | NA | NA |
| 305 | Empagliflozin_low_dosage:Inject_semaglutide_high_dosage | 0 | -0.109282188 | NA | -0.109282188 | NA | NA | NA | NA |
| 306 | Empagliflozin_low_dosage:Inject_semaglutide_low_dosage | 0 | 0.294748089 | NA | 0.294748089 | NA | NA | NA | NA |
| 307 | Empagliflozin_low_dosage:Inject_semaglutide_medium_dosage | 0 | -0.711827782 | NA | -0.711827782 | NA | NA | NA | NA |
| 308 | Empagliflozin_low_dosage:Liraglutide | 0 | -0.470378831 | NA | -0.470378831 | NA | NA | NA | NA |
| 309 | Empagliflozin_low_dosage:Lixisenatide | 0 | -0.646535218 | NA | -0.646535218 | NA | NA | NA | NA |
| 310 | Empagliflozin_low_dosage:Oral_semaglutide | 0 | 0.315959498 | NA | 0.315959498 | NA | NA | NA | NA |
| 311 | Empagliflozin_low_dosage:Placebo_or_Control | 8 | -0.223828304 | -0.275263995 | 0.477243953 | -0.752507948 | -3.184214605 | 1.67919871 | 0.544166843 |
| 312 | Empagliflozin_low_dosage:Sotagliflozin | 0 | 0.117324721 | NA | 0.117324721 | NA | NA | NA | NA |
| 313 | Empagliflozin_low_dosage:Tirzepatide_high_dosage | 0 | -0.52512149 | NA | -0.52512149 | NA | NA | NA | NA |
| 314 | Empagliflozin_low_dosage:Tirzepatide_low_dosage | 0 | -0.421189901 | NA | -0.421189901 | NA | NA | NA | NA |
| 315 | Empagliflozin_low_dosage:Tirzepatide_medium_dosage | 0 | -0.842562291 | NA | -0.842562291 | NA | NA | NA | NA |
| 316 | Ertugliflozin_high_dosage:Ertugliflozin_low_dosage | 2 | 0.572482457 | 0.485508134 | 1.920736124 | -1.43522799 | -7.379124179 | 4.508668199 | 0.636029273 |
| 317 | Ertugliflozin_high_dosage:Exenatide | 0 | 0.349677326 | NA | 0.349677326 | NA | NA | NA | NA |
| 318 | Ertugliflozin_high_dosage:Inject_semaglutide_high_dosage | 0 | 0.741876546 | NA | 0.741876546 | NA | NA | NA | NA |
| 319 | Ertugliflozin_high_dosage:Inject_semaglutide_low_dosage | 0 | 1.145906824 | NA | 1.145906824 | NA | NA | NA | NA |
| 320 | Ertugliflozin_high_dosage:Inject_semaglutide_medium_dosage | 0 | 0.139330952 | NA | 0.139330952 | NA | NA | NA | NA |
| 321 | Ertugliflozin_high_dosage:Liraglutide | 0 | 0.380779904 | NA | 0.380779904 | NA | NA | NA | NA |
| 322 | Ertugliflozin_high_dosage:Lixisenatide | 0 | 0.204623516 | NA | 0.204623516 | NA | NA | NA | NA |
| 323 | Ertugliflozin_high_dosage:Oral_semaglutide | 0 | 1.167118233 | NA | 1.167118233 | NA | NA | NA | NA |
| 324 | Ertugliflozin_high_dosage:Placebo_or_Control | 2 | 0.627330431 | 0.539094425 | 1.994406751 | -1.455312327 | -7.397298138 | 4.486673485 | 0.631202675 |
| 325 | Ertugliflozin_high_dosage:Sotagliflozin | 0 | 0.968483456 | NA | 0.968483456 | NA | NA | NA | NA |
| 326 | Ertugliflozin_high_dosage:Tirzepatide_high_dosage | 0 | 0.326037245 | NA | 0.326037245 | NA | NA | NA | NA |
| 327 | Ertugliflozin_high_dosage:Tirzepatide_low_dosage | 0 | 0.429968833 | NA | 0.429968833 | NA | NA | NA | NA |
| 328 | Ertugliflozin_high_dosage:Tirzepatide_medium_dosage | 0 | 0.008596444 | NA | 0.008596444 | NA | NA | NA | NA |
| 329 | Ertugliflozin_low_dosage:Exenatide | 0 | -0.222805131 | NA | -0.222805131 | NA | NA | NA | NA |
| 330 | Ertugliflozin_low_dosage:Inject_semaglutide_high_dosage | 0 | 0.16939409 | NA | 0.16939409 | NA | NA | NA | NA |
| 331 | Ertugliflozin_low_dosage:Inject_semaglutide_low_dosage | 0 | 0.573424367 | NA | 0.573424367 | NA | NA | NA | NA |
| 332 | Ertugliflozin_low_dosage:Inject_semaglutide_medium_dosage | 0 | -0.433151505 | NA | -0.433151505 | NA | NA | NA | NA |
| 333 | Ertugliflozin_low_dosage:Liraglutide | 0 | -0.191702553 | NA | -0.191702553 | NA | NA | NA | NA |
| 334 | Ertugliflozin_low_dosage:Lixisenatide | 0 | -0.367858941 | NA | -0.367858941 | NA | NA | NA | NA |
| 335 | Ertugliflozin_low_dosage:Oral_semaglutide | 0 | 0.594635776 | NA | 0.594635776 | NA | NA | NA | NA |
| 336 | Ertugliflozin_low_dosage:Placebo_or_Control | 2 | 0.054847974 | 0.05495874 | NA | NA | NA | NA | NA |
| 337 | Ertugliflozin_low_dosage:Sotagliflozin | 0 | 0.396000999 | NA | 0.396000999 | NA | NA | NA | NA |
| 338 | Ertugliflozin_low_dosage:Tirzepatide_high_dosage | 0 | -0.246445212 | NA | -0.246445212 | NA | NA | NA | NA |
| 339 | Ertugliflozin_low_dosage:Tirzepatide_low_dosage | 0 | -0.142513623 | NA | -0.142513623 | NA | NA | NA | NA |
| 340 | Ertugliflozin_low_dosage:Tirzepatide_medium_dosage | 0 | -0.563886013 | NA | -0.563886013 | NA | NA | NA | NA |
| 341 | Exenatide:Inject_semaglutide_high_dosage | 0 | 0.392199221 | NA | 0.392199221 | NA | NA | NA | NA |
| 342 | Exenatide:Inject_semaglutide_low_dosage | 0 | 0.796229498 | NA | 0.796229498 | NA | NA | NA | NA |
| 343 | Exenatide:Inject_semaglutide_medium_dosage | 1 | -0.210346374 | -0.654145023 | -0.092310892 | -0.561834131 | -3.272727394 | 2.149059132 | 0.684592993 |
| 344 | Exenatide:Liraglutide | 0 | 0.031102578 | NA | 0.031102578 | NA | NA | NA | NA |
| 345 | Exenatide:Lixisenatide | 0 | -0.14505381 | NA | -0.14505381 | NA | NA | NA | NA |
| 346 | Exenatide:Oral_semaglutide | 0 | 0.817440907 | NA | 0.817440907 | NA | NA | NA | NA |
| 347 | Exenatide:Placebo_or_Control | 2 | 0.277653105 | 0.302333422 | -0.259500709 | 0.561834131 | -2.149059132 | 3.272727394 | 0.684592993 |
| 348 | Exenatide:Sotagliflozin | 0 | 0.61880613 | NA | 0.61880613 | NA | NA | NA | NA |
| 349 | Exenatide:Tirzepatide_high_dosage | 0 | -0.023640081 | NA | -0.023640081 | NA | NA | NA | NA |
| 350 | Exenatide:Tirzepatide_low_dosage | 0 | 0.080291508 | NA | 0.080291508 | NA | NA | NA | NA |
| 351 | Exenatide:Tirzepatide_medium_dosage | 0 | -0.341080882 | NA | -0.341080882 | NA | NA | NA | NA |
| 352 | Inject_semaglutide_high_dosage:Inject_semaglutide_low_dosage | 0 | 0.404030277 | NA | 0.404030277 | NA | NA | NA | NA |
| 353 | Inject_semaglutide_high_dosage:Inject_semaglutide_medium_dosage | 1 | -0.602545594 | -1.197283816 | -0.519834878 | -0.677448939 | -4.099393202 | 2.744495324 | 0.69800281 |
| 354 | Inject_semaglutide_high_dosage:Liraglutide | 1 | -0.361096643 | -0.754390806 | -0.324108972 | -0.430281834 | -2.958116563 | 2.097552895 | 0.738666123 |
| 355 | Inject_semaglutide_high_dosage:Lixisenatide | 0 | -0.53725303 | NA | -0.53725303 | NA | NA | NA | NA |
| 356 | Inject_semaglutide_high_dosage:Oral_semaglutide | 0 | 0.425241686 | NA | 0.425241686 | NA | NA | NA | NA |
| 357 | Inject_semaglutide_high_dosage:Placebo_or_Control | 7 | -0.114546116 | -0.087461177 | -0.705067512 | 0.617606335 | -2.039812519 | 3.27502519 | 0.648740937 |
| 358 | Inject_semaglutide_high_dosage:Sotagliflozin | 0 | 0.22660691 | NA | 0.22660691 | NA | NA | NA | NA |
| 359 | Inject_semaglutide_high_dosage:Tirzepatide_high_dosage | 0 | -0.415839302 | NA | -0.415839302 | NA | NA | NA | NA |
| 360 | Inject_semaglutide_high_dosage:Tirzepatide_low_dosage | 0 | -0.311907713 | NA | -0.311907713 | NA | NA | NA | NA |
| 361 | Inject_semaglutide_high_dosage:Tirzepatide_medium_dosage | 0 | -0.733280103 | NA | -0.733280103 | NA | NA | NA | NA |
| 362 | Inject_semaglutide_low_dosage:Inject_semaglutide_medium_dosage | 2 | -1.006575871 | -0.683982902 | -1.717664238 | 1.033681336 | -2.214083517 | 4.281446189 | 0.532754395 |
| 363 | Inject_semaglutide_low_dosage:Liraglutide | 1 | -0.76512692 | -3.042371899 | -0.121881491 | -2.920490408 | -6.556715855 | 0.715735039 | 0.115446945 |
| 364 | Inject_semaglutide_low_dosage:Lixisenatide | 0 | -0.941283307 | NA | -0.941283307 | NA | NA | NA | NA |
| 365 | Inject_semaglutide_low_dosage:Oral_semaglutide | 0 | 0.021211409 | NA | 0.021211409 | NA | NA | NA | NA |
| 366 | Inject_semaglutide_low_dosage:Placebo_or_Control | 2 | -0.518576393 | -0.187033715 | -1.063911913 | 0.876878198 | -2.140198956 | 3.893955351 | 0.568921475 |
| 367 | Inject_semaglutide_low_dosage:Sotagliflozin | 0 | -0.177423368 | NA | -0.177423368 | NA | NA | NA | NA |
| 368 | Inject_semaglutide_low_dosage:Tirzepatide_high_dosage | 0 | -0.819869579 | NA | -0.819869579 | NA | NA | NA | NA |
| 369 | Inject_semaglutide_low_dosage:Tirzepatide_low_dosage | 0 | -0.71593799 | NA | -0.71593799 | NA | NA | NA | NA |
| 370 | Inject_semaglutide_low_dosage:Tirzepatide_medium_dosage | 0 | -1.13731038 | NA | -1.13731038 | NA | NA | NA | NA |
| 371 | Inject_semaglutide_medium_dosage:Liraglutide | 0 | 0.241448952 | NA | 0.241448952 | NA | NA | NA | NA |
| 372 | Inject_semaglutide_medium_dosage:Lixisenatide | 0 | 0.065292564 | NA | 0.065292564 | NA | NA | NA | NA |
| 373 | Inject_semaglutide_medium_dosage:Oral_semaglutide | 0 | 1.02778728 | NA | 1.02778728 | NA | NA | NA | NA |
| 374 | Inject_semaglutide_medium_dosage:Placebo_or_Control | 3 | 0.487999479 | 0.538842663 | 0.386900605 | 0.151942058 | -1.986546826 | 2.290430941 | 0.889246545 |
| 375 | Inject_semaglutide_medium_dosage:Sotagliflozin | 0 | 0.829152504 | NA | 0.829152504 | NA | NA | NA | NA |
| 376 | Inject_semaglutide_medium_dosage:Tirzepatide_high_dosage | 0 | 0.186706292 | NA | 0.186706292 | NA | NA | NA | NA |
| 377 | Inject_semaglutide_medium_dosage:Tirzepatide_low_dosage | 0 | 0.290637881 | NA | 0.290637881 | NA | NA | NA | NA |
| 378 | Inject_semaglutide_medium_dosage:Tirzepatide_medium_dosage | 0 | -0.130734509 | NA | -0.130734509 | NA | NA | NA | NA |
| 379 | Liraglutide:Lixisenatide | 0 | -0.176156388 | NA | -0.176156388 | NA | NA | NA | NA |
| 380 | Liraglutide:Oral_semaglutide | 1 | 0.786338329 | -1.083960012 | 1.031229789 | -2.115189801 | -5.52823154 | 1.297851937 | 0.224494615 |
| 381 | Liraglutide:Placebo_or_Control | 6 | 0.246550527 | 0.226007476 | 0.601631738 | -0.375624263 | -2.454719644 | 1.703471119 | 0.723263094 |
| 382 | Liraglutide:Sotagliflozin | 0 | 0.587703552 | NA | 0.587703552 | NA | NA | NA | NA |
| 383 | Liraglutide:Tirzepatide_high_dosage | 0 | -0.054742659 | NA | -0.054742659 | NA | NA | NA | NA |
| 384 | Liraglutide:Tirzepatide_low_dosage | 0 | 0.04918893 | NA | 0.04918893 | NA | NA | NA | NA |
| 385 | Liraglutide:Tirzepatide_medium_dosage | 0 | -0.37218346 | NA | -0.37218346 | NA | NA | NA | NA |
| 386 | Lixisenatide:Oral_semaglutide | 0 | 0.962494716 | NA | 0.962494716 | NA | NA | NA | NA |
| 387 | Lixisenatide:Placebo_or_Control | 1 | 0.422706915 | 0.422706915 | NA | NA | NA | NA | NA |
| 388 | Lixisenatide:Sotagliflozin | 0 | 0.76385994 | NA | 0.76385994 | NA | NA | NA | NA |
| 389 | Lixisenatide:Tirzepatide_high_dosage | 0 | 0.121413728 | NA | 0.121413728 | NA | NA | NA | NA |
| 390 | Lixisenatide:Tirzepatide_low_dosage | 0 | 0.225345317 | NA | 0.225345317 | NA | NA | NA | NA |
| 391 | Lixisenatide:Tirzepatide_medium_dosage | 0 | -0.196027073 | NA | -0.196027073 | NA | NA | NA | NA |
| 392 | Oral_semaglutide:Placebo_or_Control | 6 | -0.539787802 | -0.832796605 | 0.535669687 | -1.368466292 | -3.821948303 | 1.085015719 | 0.274306335 |
| 393 | Oral_semaglutide:Sotagliflozin | 0 | -0.198634777 | NA | -0.198634777 | NA | NA | NA | NA |
| 394 | Oral_semaglutide:Tirzepatide_high_dosage | 0 | -0.841080988 | NA | -0.841080988 | NA | NA | NA | NA |
| 395 | Oral_semaglutide:Tirzepatide_low_dosage | 0 | -0.737149399 | NA | -0.737149399 | NA | NA | NA | NA |
| 396 | Oral_semaglutide:Tirzepatide_medium_dosage | 0 | -1.158521789 | NA | -1.158521789 | NA | NA | NA | NA |
| 397 | Sotagliflozin:Placebo_or_Control | 6 | -0.341153025 | -0.341153025 | NA | NA | NA | NA | NA |
| 398 | Tirzepatide_high_dosage:Placebo_or_Control | 4 | 0.301293186 | 0.129225394 | 0.619093799 | -0.489868405 | -2.884348788 | 1.904611977 | 0.68843925 |
| 399 | Tirzepatide_low_dosage:Placebo_or_Control | 3 | 0.197361597 | 0.27797856 | 0.080532025 | 0.197446535 | -2.311860249 | 2.706753319 | 0.877435392 |
| 400 | Tirzepatide_medium_dosage:Placebo_or_Control | 4 | 0.618733987 | 0.445818708 | 0.906398593 | -0.460579884 | -2.856394616 | 1.935234847 | 0.706329377 |
| 401 | Sotagliflozin:Tirzepatide_high_dosage | 0 | -0.642446211 | NA | -0.642446211 | NA | NA | NA | NA |
| 402 | Sotagliflozin:Tirzepatide_low_dosage | 0 | -0.538514623 | NA | -0.538514623 | NA | NA | NA | NA |
| 403 | Sotagliflozin:Tirzepatide_medium_dosage | 0 | -0.959887012 | NA | -0.959887012 | NA | NA | NA | NA |
| 404 | Tirzepatide_high_dosage:Tirzepatide_low_dosage | 3 | 0.103931589 | 0.14971762 | 0.032328103 | 0.117389517 | -2.41667182 | 2.651450855 | 0.927655757 |
| 405 | Tirzepatide_high_dosage:Tirzepatide_medium_dosage | 4 | -0.317440801 | -0.022349664 | -0.725822683 | 0.703473019 | -1.707966852 | 3.114912889 | 0.567479825 |
| 406 | Tirzepatide_low_dosage:Tirzepatide_medium_dosage | 3 | -0.42137239 | -0.210936218 | -0.667648939 | 0.456712721 | -2.090302184 | 3.003727625 | 0.725253105 |

**eTable 7F: Side-splitting model inconsistency of primary outcome: subgroup of vaginal tumor**

|  | Comparison | No.Studies | NMA | Direct | Indirect | Difference | Diff_95CI_lower | Diff_95CI_upper | *p* value |
| --- | --- | --- | --- | --- | --- | --- | --- | --- | --- |
| 1 | Dapagliflozin_high_dosage:Dulaglutide_medium_dosage | 0 | -0.585671613 | NA | -0.585671613 | NA | NA | NA | NA |
| 2 | Dapagliflozin_high_dosage:Liraglutide | 0 | 0.104811341 | NA | 0.104811341 | NA | NA | NA | NA |
| 3 | Dapagliflozin_high_dosage:Placebo_or_Control | 2 | 0.503352457 | 0.503352457 | NA | NA | NA | NA | NA |
| 4 | Dulaglutide_medium_dosage:Liraglutide | 0 | 0.690482954 | NA | 0.690482954 | NA | NA | NA | NA |
| 5 | Dulaglutide_medium_dosage:Placebo_or_Control | 1 | 1.08902407 | 1.08902407 | NA | NA | NA | NA | NA |
| 6 | Liraglutide:Placebo_or_Control | 1 | 0.398541116 | 0.398541116 | NA | NA | NA | NA | NA |

**eTable 7G: Side-splitting model inconsistency of primary outcome: subgroup of vulvar tumor**

|  | Comparison | No.Studies | NMA | Direct | Indirect | Difference | Diff_95CI_lower | Diff_95CI_upper | *p* value |
| --- | --- | --- | --- | --- | --- | --- | --- | --- | --- |
| 1 | Albiglutide:Canagliflozin_low_dosage | 0 | 1.167781858 | NA | 1.167781858 | NA | NA | NA | NA |
| 2 | Albiglutide:Dapagliflozin_high_dosage | 0 | -0.56556356 | NA | -0.56556356 | NA | NA | NA | NA |
| 3 | Albiglutide:Dulaglutide_medium_dosage | 0 | -1.572619251 | NA | -1.572619251 | NA | NA | NA | NA |
| 4 | Albiglutide:Empagliflozin_low_dosage | 0 | -1.920489698 | NA | -1.920489698 | NA | NA | NA | NA |
| 5 | Albiglutide:Ertugliflozin_high_dosage | 0 | 1.11314989 | NA | 1.11314989 | NA | NA | NA | NA |
| 6 | Albiglutide:Ertugliflozin_low_dosage | 0 | 1.07520164 | NA | 1.07520164 | NA | NA | NA | NA |
| 7 | Albiglutide:Exenatide | 0 | 1.632460986 | NA | 1.632460986 | NA | NA | NA | NA |
| 8 | Albiglutide:Inject_semaglutide_medium_dosage | 0 | -1.063922217 | NA | -1.063922217 | NA | NA | NA | NA |
| 9 | Albiglutide:Liraglutide | 0 | 0.70761391 | NA | 0.70761391 | NA | NA | NA | NA |
| 10 | Albiglutide:Placebo_or_Control | 1 | 0.027664281 | 0.027664281 | NA | NA | NA | NA | NA |
| 11 | Canagliflozin_low_dosage:Dapagliflozin_high_dosage | 0 | -1.733345419 | NA | -1.733345419 | NA | NA | NA | NA |
| 12 | Canagliflozin_low_dosage:Dulaglutide_medium_dosage | 0 | -2.74040111 | NA | -2.74040111 | NA | NA | NA | NA |
| 13 | Canagliflozin_low_dosage:Empagliflozin_low_dosage | 0 | -3.088271556 | NA | -3.088271556 | NA | NA | NA | NA |
| 14 | Canagliflozin_low_dosage:Ertugliflozin_high_dosage | 0 | -0.054631968 | NA | -0.054631968 | NA | NA | NA | NA |
| 15 | Canagliflozin_low_dosage:Ertugliflozin_low_dosage | 0 | -0.092580218 | NA | -0.092580218 | NA | NA | NA | NA |
| 16 | Canagliflozin_low_dosage:Exenatide | 0 | 0.464679128 | NA | 0.464679128 | NA | NA | NA | NA |
| 17 | Canagliflozin_low_dosage:Inject_semaglutide_medium_dosage | 0 | -2.231704075 | NA | -2.231704075 | NA | NA | NA | NA |
| 18 | Canagliflozin_low_dosage:Liraglutide | 0 | -0.460167948 | NA | -0.460167948 | NA | NA | NA | NA |
| 19 | Canagliflozin_low_dosage:Placebo_or_Control | 1 | -1.140117577 | -1.140117577 | NA | NA | NA | NA | NA |
| 20 | Dapagliflozin_high_dosage:Dulaglutide_medium_dosage | 0 | -1.007055691 | NA | -1.007055691 | NA | NA | NA | NA |
| 21 | Dapagliflozin_high_dosage:Empagliflozin_low_dosage | 0 | -1.354926138 | NA | -1.354926138 | NA | NA | NA | NA |
| 22 | Dapagliflozin_high_dosage:Ertugliflozin_high_dosage | 0 | 1.67871345 | NA | 1.67871345 | NA | NA | NA | NA |
| 23 | Dapagliflozin_high_dosage:Ertugliflozin_low_dosage | 0 | 1.640765201 | NA | 1.640765201 | NA | NA | NA | NA |
| 24 | Dapagliflozin_high_dosage:Exenatide | 0 | 2.198024546 | NA | 2.198024546 | NA | NA | NA | NA |
| 25 | Dapagliflozin_high_dosage:Inject_semaglutide_medium_dosage | 0 | -0.498358657 | NA | -0.498358657 | NA | NA | NA | NA |
| 26 | Dapagliflozin_high_dosage:Liraglutide | 0 | 1.27317747 | NA | 1.27317747 | NA | NA | NA | NA |
| 27 | Dapagliflozin_high_dosage:Placebo_or_Control | 2 | 0.593227842 | 0.593227842 | NA | NA | NA | NA | NA |
| 28 | Dulaglutide_medium_dosage:Empagliflozin_low_dosage | 0 | -0.347870447 | NA | -0.347870447 | NA | NA | NA | NA |
| 29 | Dulaglutide_medium_dosage:Ertugliflozin_high_dosage | 0 | 2.685769141 | NA | 2.685769141 | NA | NA | NA | NA |
| 30 | Dulaglutide_medium_dosage:Ertugliflozin_low_dosage | 0 | 2.647820892 | NA | 2.647820892 | NA | NA | NA | NA |
| 31 | Dulaglutide_medium_dosage:Exenatide | 0 | 3.205080237 | NA | 3.205080237 | NA | NA | NA | NA |
| 32 | Dulaglutide_medium_dosage:Inject_semaglutide_medium_dosage | 0 | 0.508697034 | NA | 0.508697034 | NA | NA | NA | NA |
| 33 | Dulaglutide_medium_dosage:Liraglutide | 0 | 2.280233161 | NA | 2.280233161 | NA | NA | NA | NA |
| 34 | Dulaglutide_medium_dosage:Placebo_or_Control | 1 | 1.600283533 | 1.600283533 | NA | NA | NA | NA | NA |
| 35 | Empagliflozin_low_dosage:Ertugliflozin_high_dosage | 0 | 3.033639588 | NA | 3.033639588 | NA | NA | NA | NA |
| 36 | Empagliflozin_low_dosage:Ertugliflozin_low_dosage | 0 | 2.995691338 | NA | 2.995691338 | NA | NA | NA | NA |
| 37 | Empagliflozin_low_dosage:Exenatide | 0 | 3.552950684 | NA | 3.552950684 | NA | NA | NA | NA |
| 38 | Empagliflozin_low_dosage:Inject_semaglutide_medium_dosage | 0 | 0.856567481 | NA | 0.856567481 | NA | NA | NA | NA |
| 39 | Empagliflozin_low_dosage:Liraglutide | 0 | 2.628103608 | NA | 2.628103608 | NA | NA | NA | NA |
| 40 | Empagliflozin_low_dosage:Placebo_or_Control | 1 | 1.948153979 | 1.948153979 | NA | NA | NA | NA | NA |
| 41 | Ertugliflozin_high_dosage:Ertugliflozin_low_dosage | 0 | -0.03794825 | NA | -0.03794825 | NA | NA | NA | NA |
| 42 | Ertugliflozin_high_dosage:Exenatide | 0 | 0.519311096 | NA | 0.519311096 | NA | NA | NA | NA |
| 43 | Ertugliflozin_high_dosage:Inject_semaglutide_medium_dosage | 0 | -2.177072107 | NA | -2.177072107 | NA | NA | NA | NA |
| 44 | Ertugliflozin_high_dosage:Liraglutide | 0 | -0.40553598 | NA | -0.40553598 | NA | NA | NA | NA |
| 45 | Ertugliflozin_high_dosage:Placebo_or_Control | 1 | -1.085485609 | -1.085485609 | NA | NA | NA | NA | NA |
| 46 | Ertugliflozin_low_dosage:Exenatide | 0 | 0.557259346 | NA | 0.557259346 | NA | NA | NA | NA |
| 47 | Ertugliflozin_low_dosage:Inject_semaglutide_medium_dosage | 0 | -2.139123857 | NA | -2.139123857 | NA | NA | NA | NA |
| 48 | Ertugliflozin_low_dosage:Liraglutide | 0 | -0.36758773 | NA | -0.36758773 | NA | NA | NA | NA |
| 49 | Ertugliflozin_low_dosage:Placebo_or_Control | 1 | -1.047537359 | -1.047537359 | NA | NA | NA | NA | NA |
| 50 | Exenatide:Inject_semaglutide_medium_dosage | 0 | -2.696383203 | NA | -2.696383203 | NA | NA | NA | NA |
| 51 | Exenatide:Liraglutide | 0 | -0.924847076 | NA | -0.924847076 | NA | NA | NA | NA |
| 52 | Exenatide:Placebo_or_Control | 1 | -1.604796705 | -1.604796705 | NA | NA | NA | NA | NA |
| 53 | Inject_semaglutide_medium_dosage:Liraglutide | 0 | 1.771536127 | NA | 1.771536127 | NA | NA | NA | NA |
| 54 | Inject_semaglutide_medium_dosage:Placebo_or_Control | 1 | 1.091586499 | 1.091586499 | NA | NA | NA | NA | NA |
| 55 | Liraglutide:Placebo_or_Control | 1 | -0.679949628 | -0.679949628 | NA | NA | NA | NA | NA |

**eTable 7H: Side-splitting model inconsistency of acceptability: drop-out rate (the outcome of drop-out rate here was calculated according to drop-out rate data from the original composition of subjects from included randomized controlled trials because there were no any studies provided specific information regarding drop-out rate data in female subgroup)**

|  | Comparison | No.Studies | NMA | Direct | Indirect | Difference | Diff_95CI_lower | Diff_95CI_upper | *p* value |
| --- | --- | --- | --- | --- | --- | --- | --- | --- | --- |
| 1 | Albiglutide:Bexagliflozin | 0 | -0.035513055 | NA | -0.035513055 | NA | NA | NA | NA |
| 2 | Albiglutide:Canagliflozin_high_dosage | 0 | 0.244857781 | NA | 0.244857781 | NA | NA | NA | NA |
| 3 | Albiglutide:Canagliflozin_low_dosage | 0 | 0.21912307 | NA | 0.21912307 | NA | NA | NA | NA |
| 4 | Albiglutide:Dapagliflozin_high_dosage | 0 | 0.013093617 | NA | 0.013093617 | NA | NA | NA | NA |
| 5 | Albiglutide:Dapagliflozin_low_dosage | 0 | 0.144888157 | NA | 0.144888157 | NA | NA | NA | NA |
| 6 | Albiglutide:Dapagliflozin_medium_dosage | 0 | -0.054533359 | NA | -0.054533359 | NA | NA | NA | NA |
| 7 | Albiglutide:Dulaglutide_high_dosage | 0 | 0.439587037 | NA | 0.439587037 | NA | NA | NA | NA |
| 8 | Albiglutide:Dulaglutide_low_dosage | 0 | -0.074317961 | NA | -0.074317961 | NA | NA | NA | NA |
| 9 | Albiglutide:Dulaglutide_medium_dosage | 0 | -0.089208877 | NA | -0.089208877 | NA | NA | NA | NA |
| 10 | Albiglutide:Efpeglenatide_high_dosage | 0 | -0.131260814 | NA | -0.131260814 | NA | NA | NA | NA |
| 11 | Albiglutide:Efpeglenatide_low_dosage | 0 | 0.065167958 | NA | 0.065167958 | NA | NA | NA | NA |
| 12 | Albiglutide:Efpeglenatide_medium_dosage | 0 | -0.118750109 | NA | -0.118750109 | NA | NA | NA | NA |
| 13 | Albiglutide:Empagliflozin_high_dosage | 0 | 0.01973641 | NA | 0.01973641 | NA | NA | NA | NA |
| 14 | Albiglutide:Empagliflozin_low_dosage | 0 | 0.027198525 | NA | 0.027198525 | NA | NA | NA | NA |
| 15 | Albiglutide:Ertugliflozin_high_dosage | 0 | -0.048012869 | NA | -0.048012869 | NA | NA | NA | NA |
| 16 | Albiglutide:Ertugliflozin_low_dosage | 0 | 0.059955325 | NA | 0.059955325 | NA | NA | NA | NA |
| 17 | Albiglutide:Exenatide | 0 | -0.234167172 | NA | -0.234167172 | NA | NA | NA | NA |
| 18 | Albiglutide:Inject_semaglutide_high_dosage | 0 | 0.15355559 | NA | 0.15355559 | NA | NA | NA | NA |
| 19 | Albiglutide:Inject_semaglutide_low_dosage | 0 | 0.150594136 | NA | 0.150594136 | NA | NA | NA | NA |
| 20 | Albiglutide:Inject_semaglutide_medium_dosage | 0 | 0.123514241 | NA | 0.123514241 | NA | NA | NA | NA |
| 21 | Albiglutide:Liraglutide | 0 | -0.04121633 | NA | -0.04121633 | NA | NA | NA | NA |
| 22 | Albiglutide:Lixisenatide | 0 | -0.119719826 | NA | -0.119719826 | NA | NA | NA | NA |
| 23 | Albiglutide:Oral_semaglutide | 0 | -0.203038362 | NA | -0.203038362 | NA | NA | NA | NA |
| 24 | Albiglutide:Placebo_or_Control | 7 | -0.167948368 | -0.167948368 | NA | NA | NA | NA | NA |
| 25 | Albiglutide:Sotagliflozin | 0 | -0.009266393 | NA | -0.009266393 | NA | NA | NA | NA |
| 26 | Albiglutide:Tirzepatide_high_dosage | 0 | 0.158391809 | NA | 0.158391809 | NA | NA | NA | NA |
| 27 | Albiglutide:Tirzepatide_low_dosage | 0 | 0.281561563 | NA | 0.281561563 | NA | NA | NA | NA |
| 28 | Albiglutide:Tirzepatide_medium_dosage | 0 | 0.140763882 | NA | 0.140763882 | NA | NA | NA | NA |
| 29 | Bexagliflozin:Canagliflozin_high_dosage | 0 | 0.280370837 | NA | 0.280370837 | NA | NA | NA | NA |
| 30 | Bexagliflozin:Canagliflozin_low_dosage | 0 | 0.254636126 | NA | 0.254636126 | NA | NA | NA | NA |
| 31 | Bexagliflozin:Dapagliflozin_high_dosage | 0 | 0.048606672 | NA | 0.048606672 | NA | NA | NA | NA |
| 32 | Bexagliflozin:Dapagliflozin_low_dosage | 0 | 0.180401213 | NA | 0.180401213 | NA | NA | NA | NA |
| 33 | Bexagliflozin:Dapagliflozin_medium_dosage | 0 | -0.019020304 | NA | -0.019020304 | NA | NA | NA | NA |
| 34 | Bexagliflozin:Dulaglutide_high_dosage | 0 | 0.475100092 | NA | 0.475100092 | NA | NA | NA | NA |
| 35 | Bexagliflozin:Dulaglutide_low_dosage | 0 | -0.038804906 | NA | -0.038804906 | NA | NA | NA | NA |
| 36 | Bexagliflozin:Dulaglutide_medium_dosage | 0 | -0.053695822 | NA | -0.053695822 | NA | NA | NA | NA |
| 37 | Bexagliflozin:Efpeglenatide_high_dosage | 0 | -0.095747758 | NA | -0.095747758 | NA | NA | NA | NA |
| 38 | Bexagliflozin:Efpeglenatide_low_dosage | 0 | 0.100681013 | NA | 0.100681013 | NA | NA | NA | NA |
| 39 | Bexagliflozin:Efpeglenatide_medium_dosage | 0 | -0.083237053 | NA | -0.083237053 | NA | NA | NA | NA |
| 40 | Bexagliflozin:Empagliflozin_high_dosage | 0 | 0.055249466 | NA | 0.055249466 | NA | NA | NA | NA |
| 41 | Bexagliflozin:Empagliflozin_low_dosage | 0 | 0.06271158 | NA | 0.06271158 | NA | NA | NA | NA |
| 42 | Bexagliflozin:Ertugliflozin_high_dosage | 0 | -0.012499813 | NA | -0.012499813 | NA | NA | NA | NA |
| 43 | Bexagliflozin:Ertugliflozin_low_dosage | 0 | 0.095468381 | NA | 0.095468381 | NA | NA | NA | NA |
| 44 | Bexagliflozin:Exenatide | 0 | -0.198654117 | NA | -0.198654117 | NA | NA | NA | NA |
| 45 | Bexagliflozin:Inject_semaglutide_high_dosage | 0 | 0.189068646 | NA | 0.189068646 | NA | NA | NA | NA |
| 46 | Bexagliflozin:Inject_semaglutide_low_dosage | 0 | 0.186107191 | NA | 0.186107191 | NA | NA | NA | NA |
| 47 | Bexagliflozin:Inject_semaglutide_medium_dosage | 0 | 0.159027296 | NA | 0.159027296 | NA | NA | NA | NA |
| 48 | Bexagliflozin:Liraglutide | 0 | -0.005703274 | NA | -0.005703274 | NA | NA | NA | NA |
| 49 | Bexagliflozin:Lixisenatide | 0 | -0.084206771 | NA | -0.084206771 | NA | NA | NA | NA |
| 50 | Bexagliflozin:Oral_semaglutide | 0 | -0.167525307 | NA | -0.167525307 | NA | NA | NA | NA |
| 51 | Bexagliflozin:Placebo_or_Control | 1 | -0.132435312 | -0.132435312 | NA | NA | NA | NA | NA |
| 52 | Bexagliflozin:Sotagliflozin | 0 | 0.026246662 | NA | 0.026246662 | NA | NA | NA | NA |
| 53 | Bexagliflozin:Tirzepatide_high_dosage | 0 | 0.193904865 | NA | 0.193904865 | NA | NA | NA | NA |
| 54 | Bexagliflozin:Tirzepatide_low_dosage | 0 | 0.317074619 | NA | 0.317074619 | NA | NA | NA | NA |
| 55 | Bexagliflozin:Tirzepatide_medium_dosage | 0 | 0.176276938 | NA | 0.176276938 | NA | NA | NA | NA |
| 56 | Canagliflozin_high_dosage:Canagliflozin_low_dosage | 3 | -0.025734711 | -0.059242224 | 0.154241742 | -0.213483966 | -0.987675144 | 0.560707212 | 0.588878485 |
| 57 | Canagliflozin_high_dosage:Dapagliflozin_high_dosage | 0 | -0.231764165 | NA | -0.231764165 | NA | NA | NA | NA |
| 58 | Canagliflozin_high_dosage:Dapagliflozin_low_dosage | 0 | -0.099969624 | NA | -0.099969624 | NA | NA | NA | NA |
| 59 | Canagliflozin_high_dosage:Dapagliflozin_medium_dosage | 0 | -0.299391141 | NA | -0.299391141 | NA | NA | NA | NA |
| 60 | Canagliflozin_high_dosage:Dulaglutide_high_dosage | 0 | 0.194729255 | NA | 0.194729255 | NA | NA | NA | NA |
| 61 | Canagliflozin_high_dosage:Dulaglutide_low_dosage | 0 | -0.319175742 | NA | -0.319175742 | NA | NA | NA | NA |
| 62 | Canagliflozin_high_dosage:Dulaglutide_medium_dosage | 0 | -0.334066659 | NA | -0.334066659 | NA | NA | NA | NA |
| 63 | Canagliflozin_high_dosage:Efpeglenatide_high_dosage | 0 | -0.376118595 | NA | -0.376118595 | NA | NA | NA | NA |
| 64 | Canagliflozin_high_dosage:Efpeglenatide_low_dosage | 0 | -0.179689823 | NA | -0.179689823 | NA | NA | NA | NA |
| 65 | Canagliflozin_high_dosage:Efpeglenatide_medium_dosage | 0 | -0.36360789 | NA | -0.36360789 | NA | NA | NA | NA |
| 66 | Canagliflozin_high_dosage:Empagliflozin_high_dosage | 0 | -0.225121371 | NA | -0.225121371 | NA | NA | NA | NA |
| 67 | Canagliflozin_high_dosage:Empagliflozin_low_dosage | 0 | -0.217659256 | NA | -0.217659256 | NA | NA | NA | NA |
| 68 | Canagliflozin_high_dosage:Ertugliflozin_high_dosage | 0 | -0.29287065 | NA | -0.29287065 | NA | NA | NA | NA |
| 69 | Canagliflozin_high_dosage:Ertugliflozin_low_dosage | 0 | -0.184902456 | NA | -0.184902456 | NA | NA | NA | NA |
| 70 | Canagliflozin_high_dosage:Exenatide | 0 | -0.479024953 | NA | -0.479024953 | NA | NA | NA | NA |
| 71 | Canagliflozin_high_dosage:Inject_semaglutide_high_dosage | 0 | -0.091302191 | NA | -0.091302191 | NA | NA | NA | NA |
| 72 | Canagliflozin_high_dosage:Inject_semaglutide_low_dosage | 0 | -0.094263645 | NA | -0.094263645 | NA | NA | NA | NA |
| 73 | Canagliflozin_high_dosage:Inject_semaglutide_medium_dosage | 0 | -0.12134354 | NA | -0.12134354 | NA | NA | NA | NA |
| 74 | Canagliflozin_high_dosage:Liraglutide | 0 | -0.286074111 | NA | -0.286074111 | NA | NA | NA | NA |
| 75 | Canagliflozin_high_dosage:Lixisenatide | 0 | -0.364577608 | NA | -0.364577608 | NA | NA | NA | NA |
| 76 | Canagliflozin_high_dosage:Oral_semaglutide | 0 | -0.447896143 | NA | -0.447896143 | NA | NA | NA | NA |
| 77 | Canagliflozin_high_dosage:Placebo_or_Control | 5 | -0.412806149 | -0.405087143 | -0.731056786 | 0.325969643 | -1.212940173 | 1.864879458 | 0.678027176 |
| 78 | Canagliflozin_high_dosage:Sotagliflozin | 0 | -0.254124174 | NA | -0.254124174 | NA | NA | NA | NA |
| 79 | Canagliflozin_high_dosage:Tirzepatide_high_dosage | 0 | -0.086465972 | NA | -0.086465972 | NA | NA | NA | NA |
| 80 | Canagliflozin_high_dosage:Tirzepatide_low_dosage | 0 | 0.036703782 | NA | 0.036703782 | NA | NA | NA | NA |
| 81 | Canagliflozin_high_dosage:Tirzepatide_medium_dosage | 0 | -0.104093899 | NA | -0.104093899 | NA | NA | NA | NA |
| 82 | Canagliflozin_low_dosage:Dapagliflozin_high_dosage | 0 | -0.206029454 | NA | -0.206029454 | NA | NA | NA | NA |
| 83 | Canagliflozin_low_dosage:Dapagliflozin_low_dosage | 0 | -0.074234913 | NA | -0.074234913 | NA | NA | NA | NA |
| 84 | Canagliflozin_low_dosage:Dapagliflozin_medium_dosage | 0 | -0.273656429 | NA | -0.273656429 | NA | NA | NA | NA |
| 85 | Canagliflozin_low_dosage:Dulaglutide_high_dosage | 0 | 0.220463967 | NA | 0.220463967 | NA | NA | NA | NA |
| 86 | Canagliflozin_low_dosage:Dulaglutide_low_dosage | 0 | -0.293441031 | NA | -0.293441031 | NA | NA | NA | NA |
| 87 | Canagliflozin_low_dosage:Dulaglutide_medium_dosage | 0 | -0.308331948 | NA | -0.308331948 | NA | NA | NA | NA |
| 88 | Canagliflozin_low_dosage:Efpeglenatide_high_dosage | 0 | -0.350383884 | NA | -0.350383884 | NA | NA | NA | NA |
| 89 | Canagliflozin_low_dosage:Efpeglenatide_low_dosage | 0 | -0.153955112 | NA | -0.153955112 | NA | NA | NA | NA |
| 90 | Canagliflozin_low_dosage:Efpeglenatide_medium_dosage | 0 | -0.337873179 | NA | -0.337873179 | NA | NA | NA | NA |
| 91 | Canagliflozin_low_dosage:Empagliflozin_high_dosage | 0 | -0.19938666 | NA | -0.19938666 | NA | NA | NA | NA |
| 92 | Canagliflozin_low_dosage:Empagliflozin_low_dosage | 0 | -0.191924545 | NA | -0.191924545 | NA | NA | NA | NA |
| 93 | Canagliflozin_low_dosage:Ertugliflozin_high_dosage | 0 | -0.267135939 | NA | -0.267135939 | NA | NA | NA | NA |
| 94 | Canagliflozin_low_dosage:Ertugliflozin_low_dosage | 0 | -0.159167745 | NA | -0.159167745 | NA | NA | NA | NA |
| 95 | Canagliflozin_low_dosage:Exenatide | 0 | -0.453290242 | NA | -0.453290242 | NA | NA | NA | NA |
| 96 | Canagliflozin_low_dosage:Inject_semaglutide_high_dosage | 0 | -0.06556748 | NA | -0.06556748 | NA | NA | NA | NA |
| 97 | Canagliflozin_low_dosage:Inject_semaglutide_low_dosage | 0 | -0.068528934 | NA | -0.068528934 | NA | NA | NA | NA |
| 98 | Canagliflozin_low_dosage:Inject_semaglutide_medium_dosage | 0 | -0.095608829 | NA | -0.095608829 | NA | NA | NA | NA |
| 99 | Canagliflozin_low_dosage:Liraglutide | 0 | -0.2603394 | NA | -0.2603394 | NA | NA | NA | NA |
| 100 | Canagliflozin_low_dosage:Lixisenatide | 0 | -0.338842896 | NA | -0.338842896 | NA | NA | NA | NA |
| 101 | Canagliflozin_low_dosage:Oral_semaglutide | 0 | -0.422161432 | NA | -0.422161432 | NA | NA | NA | NA |
| 102 | Canagliflozin_low_dosage:Placebo_or_Control | 4 | -0.387071438 | -0.403487457 | -0.223220883 | -0.180266573 | -1.117999904 | 0.757466757 | 0.706339619 |
| 103 | Canagliflozin_low_dosage:Sotagliflozin | 0 | -0.228389463 | NA | -0.228389463 | NA | NA | NA | NA |
| 104 | Canagliflozin_low_dosage:Tirzepatide_high_dosage | 0 | -0.060731261 | NA | -0.060731261 | NA | NA | NA | NA |
| 105 | Canagliflozin_low_dosage:Tirzepatide_low_dosage | 0 | 0.062438493 | NA | 0.062438493 | NA | NA | NA | NA |
| 106 | Canagliflozin_low_dosage:Tirzepatide_medium_dosage | 0 | -0.078359188 | NA | -0.078359188 | NA | NA | NA | NA |
| 107 | Dapagliflozin_high_dosage:Dapagliflozin_low_dosage | 3 | 0.131794541 | 0.068091896 | 0.447317874 | -0.379225977 | -1.479721497 | 0.721269543 | 0.499424792 |
| 108 | Dapagliflozin_high_dosage:Dapagliflozin_medium_dosage | 3 | -0.067626976 | -0.137896094 | 0.275917425 | -0.413813519 | -1.450883413 | 0.623256375 | 0.434174376 |
| 109 | Dapagliflozin_high_dosage:Dulaglutide_high_dosage | 0 | 0.42649342 | NA | 0.42649342 | NA | NA | NA | NA |
| 110 | Dapagliflozin_high_dosage:Dulaglutide_low_dosage | 0 | -0.087411578 | NA | -0.087411578 | NA | NA | NA | NA |
| 111 | Dapagliflozin_high_dosage:Dulaglutide_medium_dosage | 0 | -0.102302494 | NA | -0.102302494 | NA | NA | NA | NA |
| 112 | Dapagliflozin_high_dosage:Efpeglenatide_high_dosage | 0 | -0.14435443 | NA | -0.14435443 | NA | NA | NA | NA |
| 113 | Dapagliflozin_high_dosage:Efpeglenatide_low_dosage | 0 | 0.052074341 | NA | 0.052074341 | NA | NA | NA | NA |
| 114 | Dapagliflozin_high_dosage:Efpeglenatide_medium_dosage | 0 | -0.131843725 | NA | -0.131843725 | NA | NA | NA | NA |
| 115 | Dapagliflozin_high_dosage:Empagliflozin_high_dosage | 0 | 0.006642794 | NA | 0.006642794 | NA | NA | NA | NA |
| 116 | Dapagliflozin_high_dosage:Empagliflozin_low_dosage | 0 | 0.014104908 | NA | 0.014104908 | NA | NA | NA | NA |
| 117 | Dapagliflozin_high_dosage:Ertugliflozin_high_dosage | 0 | -0.061106485 | NA | -0.061106485 | NA | NA | NA | NA |
| 118 | Dapagliflozin_high_dosage:Ertugliflozin_low_dosage | 0 | 0.046861709 | NA | 0.046861709 | NA | NA | NA | NA |
| 119 | Dapagliflozin_high_dosage:Exenatide | 0 | -0.247260789 | NA | -0.247260789 | NA | NA | NA | NA |
| 120 | Dapagliflozin_high_dosage:Inject_semaglutide_high_dosage | 0 | 0.140461974 | NA | 0.140461974 | NA | NA | NA | NA |
| 121 | Dapagliflozin_high_dosage:Inject_semaglutide_low_dosage | 0 | 0.137500519 | NA | 0.137500519 | NA | NA | NA | NA |
| 122 | Dapagliflozin_high_dosage:Inject_semaglutide_medium_dosage | 0 | 0.110420625 | NA | 0.110420625 | NA | NA | NA | NA |
| 123 | Dapagliflozin_high_dosage:Liraglutide | 0 | -0.054309946 | NA | -0.054309946 | NA | NA | NA | NA |
| 124 | Dapagliflozin_high_dosage:Lixisenatide | 0 | -0.132813443 | NA | -0.132813443 | NA | NA | NA | NA |
| 125 | Dapagliflozin_high_dosage:Oral_semaglutide | 0 | -0.216131979 | NA | -0.216131979 | NA | NA | NA | NA |
| 126 | Dapagliflozin_high_dosage:Placebo_or_Control | 9 | -0.181041984 | -0.182969044 | 0.058517414 | -0.241486458 | -3.108765921 | 2.625793005 | 0.868888079 |
| 127 | Dapagliflozin_high_dosage:Sotagliflozin | 0 | -0.02236001 | NA | -0.02236001 | NA | NA | NA | NA |
| 128 | Dapagliflozin_high_dosage:Tirzepatide_high_dosage | 0 | 0.145298193 | NA | 0.145298193 | NA | NA | NA | NA |
| 129 | Dapagliflozin_high_dosage:Tirzepatide_low_dosage | 0 | 0.268467947 | NA | 0.268467947 | NA | NA | NA | NA |
| 130 | Dapagliflozin_high_dosage:Tirzepatide_medium_dosage | 0 | 0.127670266 | NA | 0.127670266 | NA | NA | NA | NA |
| 131 | Dapagliflozin_low_dosage:Dapagliflozin_medium_dosage | 3 | -0.199421516 | -0.199053265 | -0.254096034 | 0.055042768 | -5.35554363 | 5.465629167 | 0.98409201 |
| 132 | Dapagliflozin_low_dosage:Dulaglutide_high_dosage | 0 | 0.29469888 | NA | 0.29469888 | NA | NA | NA | NA |
| 133 | Dapagliflozin_low_dosage:Dulaglutide_low_dosage | 0 | -0.219206118 | NA | -0.219206118 | NA | NA | NA | NA |
| 134 | Dapagliflozin_low_dosage:Dulaglutide_medium_dosage | 0 | -0.234097035 | NA | -0.234097035 | NA | NA | NA | NA |
| 135 | Dapagliflozin_low_dosage:Efpeglenatide_high_dosage | 0 | -0.276148971 | NA | -0.276148971 | NA | NA | NA | NA |
| 136 | Dapagliflozin_low_dosage:Efpeglenatide_low_dosage | 0 | -0.079720199 | NA | -0.079720199 | NA | NA | NA | NA |
| 137 | Dapagliflozin_low_dosage:Efpeglenatide_medium_dosage | 0 | -0.263638266 | NA | -0.263638266 | NA | NA | NA | NA |
| 138 | Dapagliflozin_low_dosage:Empagliflozin_high_dosage | 0 | -0.125151747 | NA | -0.125151747 | NA | NA | NA | NA |
| 139 | Dapagliflozin_low_dosage:Empagliflozin_low_dosage | 0 | -0.117689632 | NA | -0.117689632 | NA | NA | NA | NA |
| 140 | Dapagliflozin_low_dosage:Ertugliflozin_high_dosage | 0 | -0.192901026 | NA | -0.192901026 | NA | NA | NA | NA |
| 141 | Dapagliflozin_low_dosage:Ertugliflozin_low_dosage | 0 | -0.084932832 | NA | -0.084932832 | NA | NA | NA | NA |
| 142 | Dapagliflozin_low_dosage:Exenatide | 0 | -0.379055329 | NA | -0.379055329 | NA | NA | NA | NA |
| 143 | Dapagliflozin_low_dosage:Inject_semaglutide_high_dosage | 0 | 0.008667433 | NA | 0.008667433 | NA | NA | NA | NA |
| 144 | Dapagliflozin_low_dosage:Inject_semaglutide_low_dosage | 0 | 0.005705979 | NA | 0.005705979 | NA | NA | NA | NA |
| 145 | Dapagliflozin_low_dosage:Inject_semaglutide_medium_dosage | 0 | -0.021373916 | NA | -0.021373916 | NA | NA | NA | NA |
| 146 | Dapagliflozin_low_dosage:Liraglutide | 0 | -0.186104487 | NA | -0.186104487 | NA | NA | NA | NA |
| 147 | Dapagliflozin_low_dosage:Lixisenatide | 0 | -0.264607983 | NA | -0.264607983 | NA | NA | NA | NA |
| 148 | Dapagliflozin_low_dosage:Oral_semaglutide | 0 | -0.347926519 | NA | -0.347926519 | NA | NA | NA | NA |
| 149 | Dapagliflozin_low_dosage:Placebo_or_Control | 3 | -0.312836525 | -0.361716156 | -0.067568576 | -0.29414758 | -1.401152899 | 0.812857738 | 0.602512212 |
| 150 | Dapagliflozin_low_dosage:Sotagliflozin | 0 | -0.15415455 | NA | -0.15415455 | NA | NA | NA | NA |
| 151 | Dapagliflozin_low_dosage:Tirzepatide_high_dosage | 0 | 0.013503652 | NA | 0.013503652 | NA | NA | NA | NA |
| 152 | Dapagliflozin_low_dosage:Tirzepatide_low_dosage | 0 | 0.136673406 | NA | 0.136673406 | NA | NA | NA | NA |
| 153 | Dapagliflozin_low_dosage:Tirzepatide_medium_dosage | 0 | -0.004124275 | NA | -0.004124275 | NA | NA | NA | NA |
| 154 | Dapagliflozin_medium_dosage:Dulaglutide_high_dosage | 0 | 0.494120396 | NA | 0.494120396 | NA | NA | NA | NA |
| 155 | Dapagliflozin_medium_dosage:Dulaglutide_low_dosage | 0 | -0.019784602 | NA | -0.019784602 | NA | NA | NA | NA |
| 156 | Dapagliflozin_medium_dosage:Dulaglutide_medium_dosage | 0 | -0.034675518 | NA | -0.034675518 | NA | NA | NA | NA |
| 157 | Dapagliflozin_medium_dosage:Efpeglenatide_high_dosage | 0 | -0.076727454 | NA | -0.076727454 | NA | NA | NA | NA |
| 158 | Dapagliflozin_medium_dosage:Efpeglenatide_low_dosage | 0 | 0.119701317 | NA | 0.119701317 | NA | NA | NA | NA |
| 159 | Dapagliflozin_medium_dosage:Efpeglenatide_medium_dosage | 0 | -0.064216749 | NA | -0.064216749 | NA | NA | NA | NA |
| 160 | Dapagliflozin_medium_dosage:Empagliflozin_high_dosage | 0 | 0.074269769 | NA | 0.074269769 | NA | NA | NA | NA |
| 161 | Dapagliflozin_medium_dosage:Empagliflozin_low_dosage | 0 | 0.081731884 | NA | 0.081731884 | NA | NA | NA | NA |
| 162 | Dapagliflozin_medium_dosage:Ertugliflozin_high_dosage | 0 | 0.006520491 | NA | 0.006520491 | NA | NA | NA | NA |
| 163 | Dapagliflozin_medium_dosage:Ertugliflozin_low_dosage | 0 | 0.114488685 | NA | 0.114488685 | NA | NA | NA | NA |
| 164 | Dapagliflozin_medium_dosage:Exenatide | 0 | -0.179633813 | NA | -0.179633813 | NA | NA | NA | NA |
| 165 | Dapagliflozin_medium_dosage:Inject_semaglutide_high_dosage | 0 | 0.20808895 | NA | 0.20808895 | NA | NA | NA | NA |
| 166 | Dapagliflozin_medium_dosage:Inject_semaglutide_low_dosage | 0 | 0.205127495 | NA | 0.205127495 | NA | NA | NA | NA |
| 167 | Dapagliflozin_medium_dosage:Inject_semaglutide_medium_dosage | 0 | 0.1780476 | NA | 0.1780476 | NA | NA | NA | NA |
| 168 | Dapagliflozin_medium_dosage:Liraglutide | 0 | 0.013317029 | NA | 0.013317029 | NA | NA | NA | NA |
| 169 | Dapagliflozin_medium_dosage:Lixisenatide | 0 | -0.065186467 | NA | -0.065186467 | NA | NA | NA | NA |
| 170 | Dapagliflozin_medium_dosage:Oral_semaglutide | 0 | -0.148505003 | NA | -0.148505003 | NA | NA | NA | NA |
| 171 | Dapagliflozin_medium_dosage:Placebo_or_Control | 3 | -0.113415009 | -0.191615389 | 0.202252755 | -0.393868144 | -1.376510336 | 0.588774048 | 0.432099675 |
| 172 | Dapagliflozin_medium_dosage:Sotagliflozin | 0 | 0.045266966 | NA | 0.045266966 | NA | NA | NA | NA |
| 173 | Dapagliflozin_medium_dosage:Tirzepatide_high_dosage | 0 | 0.212925169 | NA | 0.212925169 | NA | NA | NA | NA |
| 174 | Dapagliflozin_medium_dosage:Tirzepatide_low_dosage | 0 | 0.336094923 | NA | 0.336094923 | NA | NA | NA | NA |
| 175 | Dapagliflozin_medium_dosage:Tirzepatide_medium_dosage | 0 | 0.195297242 | NA | 0.195297242 | NA | NA | NA | NA |
| 176 | Dulaglutide_high_dosage:Dulaglutide_low_dosage | 1 | -0.513904998 | -0.376082844 | -2.111737425 | 1.735654581 | -1.061916667 | 4.533225829 | 0.223988485 |
| 177 | Dulaglutide_high_dosage:Dulaglutide_medium_dosage | 1 | -0.528795914 | -0.472604411 | -1.095049798 | 0.622445387 | -2.016675144 | 3.261565919 | 0.643891978 |
| 178 | Dulaglutide_high_dosage:Efpeglenatide_high_dosage | 0 | -0.57084785 | NA | -0.57084785 | NA | NA | NA | NA |
| 179 | Dulaglutide_high_dosage:Efpeglenatide_low_dosage | 0 | -0.374419079 | NA | -0.374419079 | NA | NA | NA | NA |
| 180 | Dulaglutide_high_dosage:Efpeglenatide_medium_dosage | 0 | -0.558337145 | NA | -0.558337145 | NA | NA | NA | NA |
| 181 | Dulaglutide_high_dosage:Empagliflozin_high_dosage | 0 | -0.419850627 | NA | -0.419850627 | NA | NA | NA | NA |
| 182 | Dulaglutide_high_dosage:Empagliflozin_low_dosage | 0 | -0.412388512 | NA | -0.412388512 | NA | NA | NA | NA |
| 183 | Dulaglutide_high_dosage:Ertugliflozin_high_dosage | 0 | -0.487599905 | NA | -0.487599905 | NA | NA | NA | NA |
| 184 | Dulaglutide_high_dosage:Ertugliflozin_low_dosage | 0 | -0.379631711 | NA | -0.379631711 | NA | NA | NA | NA |
| 185 | Dulaglutide_high_dosage:Exenatide | 0 | -0.673754209 | NA | -0.673754209 | NA | NA | NA | NA |
| 186 | Dulaglutide_high_dosage:Inject_semaglutide_high_dosage | 0 | -0.286031446 | NA | -0.286031446 | NA | NA | NA | NA |
| 187 | Dulaglutide_high_dosage:Inject_semaglutide_low_dosage | 0 | -0.288992901 | NA | -0.288992901 | NA | NA | NA | NA |
| 188 | Dulaglutide_high_dosage:Inject_semaglutide_medium_dosage | 0 | -0.316072796 | NA | -0.316072796 | NA | NA | NA | NA |
| 189 | Dulaglutide_high_dosage:Liraglutide | 0 | -0.480803367 | NA | -0.480803367 | NA | NA | NA | NA |
| 190 | Dulaglutide_high_dosage:Lixisenatide | 0 | -0.559306863 | NA | -0.559306863 | NA | NA | NA | NA |
| 191 | Dulaglutide_high_dosage:Oral_semaglutide | 0 | -0.642625399 | NA | -0.642625399 | NA | NA | NA | NA |
| 192 | Dulaglutide_high_dosage:Placebo_or_Control | 1 | -0.607535405 | -0.864443267 | 1.189372447 | -2.053815715 | -4.353859947 | 0.246228517 | 0.080093753 |
| 193 | Dulaglutide_high_dosage:Sotagliflozin | 0 | -0.44885343 | NA | -0.44885343 | NA | NA | NA | NA |
| 194 | Dulaglutide_high_dosage:Tirzepatide_high_dosage | 0 | -0.281195227 | NA | -0.281195227 | NA | NA | NA | NA |
| 195 | Dulaglutide_high_dosage:Tirzepatide_low_dosage | 0 | -0.158025473 | NA | -0.158025473 | NA | NA | NA | NA |
| 196 | Dulaglutide_high_dosage:Tirzepatide_medium_dosage | 0 | -0.298823154 | NA | -0.298823154 | NA | NA | NA | NA |
| 197 | Dulaglutide_low_dosage:Dulaglutide_medium_dosage | 5 | -0.014890916 | -0.005835476 | -0.306593826 | 0.300758351 | -1.309832267 | 1.911348968 | 0.714365368 |
| 198 | Dulaglutide_low_dosage:Efpeglenatide_high_dosage | 0 | -0.056942853 | NA | -0.056942853 | NA | NA | NA | NA |
| 199 | Dulaglutide_low_dosage:Efpeglenatide_low_dosage | 0 | 0.139485919 | NA | 0.139485919 | NA | NA | NA | NA |
| 200 | Dulaglutide_low_dosage:Efpeglenatide_medium_dosage | 0 | -0.044432147 | NA | -0.044432147 | NA | NA | NA | NA |
| 201 | Dulaglutide_low_dosage:Empagliflozin_high_dosage | 0 | 0.094054371 | NA | 0.094054371 | NA | NA | NA | NA |
| 202 | Dulaglutide_low_dosage:Empagliflozin_low_dosage | 0 | 0.101516486 | NA | 0.101516486 | NA | NA | NA | NA |
| 203 | Dulaglutide_low_dosage:Ertugliflozin_high_dosage | 0 | 0.026305092 | NA | 0.026305092 | NA | NA | NA | NA |
| 204 | Dulaglutide_low_dosage:Ertugliflozin_low_dosage | 0 | 0.134273287 | NA | 0.134273287 | NA | NA | NA | NA |
| 205 | Dulaglutide_low_dosage:Exenatide | 0 | -0.159849211 | NA | -0.159849211 | NA | NA | NA | NA |
| 206 | Dulaglutide_low_dosage:Inject_semaglutide_high_dosage | 0 | 0.227873552 | NA | 0.227873552 | NA | NA | NA | NA |
| 207 | Dulaglutide_low_dosage:Inject_semaglutide_low_dosage | 0 | 0.224912097 | NA | 0.224912097 | NA | NA | NA | NA |
| 208 | Dulaglutide_low_dosage:Inject_semaglutide_medium_dosage | 0 | 0.197832202 | NA | 0.197832202 | NA | NA | NA | NA |
| 209 | Dulaglutide_low_dosage:Liraglutide | 0 | 0.033101631 | NA | 0.033101631 | NA | NA | NA | NA |
| 210 | Dulaglutide_low_dosage:Lixisenatide | 0 | -0.045401865 | NA | -0.045401865 | NA | NA | NA | NA |
| 211 | Dulaglutide_low_dosage:Oral_semaglutide | 0 | -0.128720401 | NA | -0.128720401 | NA | NA | NA | NA |
| 212 | Dulaglutide_low_dosage:Placebo_or_Control | 5 | -0.093630407 | -0.097361826 | 0.004004161 | -0.101365988 | -1.602138279 | 1.399406304 | 0.89468297 |
| 213 | Dulaglutide_low_dosage:Sotagliflozin | 0 | 0.065051568 | NA | 0.065051568 | NA | NA | NA | NA |
| 214 | Dulaglutide_low_dosage:Tirzepatide_high_dosage | 0 | 0.232709771 | NA | 0.232709771 | NA | NA | NA | NA |
| 215 | Dulaglutide_low_dosage:Tirzepatide_low_dosage | 0 | 0.355879524 | NA | 0.355879524 | NA | NA | NA | NA |
| 216 | Dulaglutide_low_dosage:Tirzepatide_medium_dosage | 0 | 0.215081843 | NA | 0.215081843 | NA | NA | NA | NA |
| 217 | Dulaglutide_medium_dosage:Efpeglenatide_high_dosage | 0 | -0.042051936 | NA | -0.042051936 | NA | NA | NA | NA |
| 218 | Dulaglutide_medium_dosage:Efpeglenatide_low_dosage | 0 | 0.154376835 | NA | 0.154376835 | NA | NA | NA | NA |
| 219 | Dulaglutide_medium_dosage:Efpeglenatide_medium_dosage | 0 | -0.029541231 | NA | -0.029541231 | NA | NA | NA | NA |
| 220 | Dulaglutide_medium_dosage:Empagliflozin_high_dosage | 0 | 0.108945288 | NA | 0.108945288 | NA | NA | NA | NA |
| 221 | Dulaglutide_medium_dosage:Empagliflozin_low_dosage | 0 | 0.116407402 | NA | 0.116407402 | NA | NA | NA | NA |
| 222 | Dulaglutide_medium_dosage:Ertugliflozin_high_dosage | 0 | 0.041196009 | NA | 0.041196009 | NA | NA | NA | NA |
| 223 | Dulaglutide_medium_dosage:Ertugliflozin_low_dosage | 0 | 0.149164203 | NA | 0.149164203 | NA | NA | NA | NA |
| 224 | Dulaglutide_medium_dosage:Exenatide | 0 | -0.144958295 | NA | -0.144958295 | NA | NA | NA | NA |
| 225 | Dulaglutide_medium_dosage:Inject_semaglutide_high_dosage | 0 | 0.242764468 | NA | 0.242764468 | NA | NA | NA | NA |
| 226 | Dulaglutide_medium_dosage:Inject_semaglutide_low_dosage | 0 | 0.239803013 | NA | 0.239803013 | NA | NA | NA | NA |
| 227 | Dulaglutide_medium_dosage:Inject_semaglutide_medium_dosage | 0 | 0.212723118 | NA | 0.212723118 | NA | NA | NA | NA |
| 228 | Dulaglutide_medium_dosage:Liraglutide | 0 | 0.047992548 | NA | 0.047992548 | NA | NA | NA | NA |
| 229 | Dulaglutide_medium_dosage:Lixisenatide | 0 | -0.030510949 | NA | -0.030510949 | NA | NA | NA | NA |
| 230 | Dulaglutide_medium_dosage:Oral_semaglutide | 0 | -0.113829485 | NA | -0.113829485 | NA | NA | NA | NA |
| 231 | Dulaglutide_medium_dosage:Placebo_or_Control | 6 | -0.07873949 | -0.084258466 | 2.216284967 | -2.300543433 | -7.838479042 | 3.237392176 | 0.415530892 |
| 232 | Dulaglutide_medium_dosage:Sotagliflozin | 0 | 0.079942484 | NA | 0.079942484 | NA | NA | NA | NA |
| 233 | Dulaglutide_medium_dosage:Tirzepatide_high_dosage | 0 | 0.247600687 | NA | 0.247600687 | NA | NA | NA | NA |
| 234 | Dulaglutide_medium_dosage:Tirzepatide_low_dosage | 0 | 0.370770441 | NA | 0.370770441 | NA | NA | NA | NA |
| 235 | Dulaglutide_medium_dosage:Tirzepatide_medium_dosage | 0 | 0.22997276 | NA | 0.22997276 | NA | NA | NA | NA |
| 236 | Efpeglenatide_high_dosage:Efpeglenatide_low_dosage | 2 | 0.196428771 | 0.180305313 | 0.26093537 | -0.080630057 | -1.278827925 | 1.117567811 | 0.895070182 |
| 237 | Efpeglenatide_high_dosage:Efpeglenatide_medium_dosage | 3 | 0.012510705 | 0.012987508 | NA | NA | NA | NA | NA |
| 238 | Efpeglenatide_high_dosage:Empagliflozin_high_dosage | 0 | 0.150997224 | NA | 0.150997224 | NA | NA | NA | NA |
| 239 | Efpeglenatide_high_dosage:Empagliflozin_low_dosage | 0 | 0.158459339 | NA | 0.158459339 | NA | NA | NA | NA |
| 240 | Efpeglenatide_high_dosage:Ertugliflozin_high_dosage | 0 | 0.083247945 | NA | 0.083247945 | NA | NA | NA | NA |
| 241 | Efpeglenatide_high_dosage:Ertugliflozin_low_dosage | 0 | 0.191216139 | NA | 0.191216139 | NA | NA | NA | NA |
| 242 | Efpeglenatide_high_dosage:Exenatide | 0 | -0.102906358 | NA | -0.102906358 | NA | NA | NA | NA |
| 243 | Efpeglenatide_high_dosage:Inject_semaglutide_high_dosage | 0 | 0.284816404 | NA | 0.284816404 | NA | NA | NA | NA |
| 244 | Efpeglenatide_high_dosage:Inject_semaglutide_low_dosage | 0 | 0.28185495 | NA | 0.28185495 | NA | NA | NA | NA |
| 245 | Efpeglenatide_high_dosage:Inject_semaglutide_medium_dosage | 0 | 0.254775055 | NA | 0.254775055 | NA | NA | NA | NA |
| 246 | Efpeglenatide_high_dosage:Liraglutide | 0 | 0.090044484 | NA | 0.090044484 | NA | NA | NA | NA |
| 247 | Efpeglenatide_high_dosage:Lixisenatide | 0 | 0.011540987 | NA | 0.011540987 | NA | NA | NA | NA |
| 248 | Efpeglenatide_high_dosage:Oral_semaglutide | 0 | -0.071777548 | NA | -0.071777548 | NA | NA | NA | NA |
| 249 | Efpeglenatide_high_dosage:Placebo_or_Control | 3 | -0.036687554 | -0.036483131 | NA | NA | NA | NA | NA |
| 250 | Efpeglenatide_high_dosage:Sotagliflozin | 0 | 0.121994421 | NA | 0.121994421 | NA | NA | NA | NA |
| 251 | Efpeglenatide_high_dosage:Tirzepatide_high_dosage | 0 | 0.289652623 | NA | 0.289652623 | NA | NA | NA | NA |
| 252 | Efpeglenatide_high_dosage:Tirzepatide_low_dosage | 0 | 0.412822377 | NA | 0.412822377 | NA | NA | NA | NA |
| 253 | Efpeglenatide_high_dosage:Tirzepatide_medium_dosage | 0 | 0.272024696 | NA | 0.272024696 | NA | NA | NA | NA |
| 254 | Efpeglenatide_low_dosage:Efpeglenatide_medium_dosage | 2 | -0.183918066 | -0.175325431 | -0.218659302 | 0.043333871 | -1.157863409 | 1.244531151 | 0.943631102 |
| 255 | Efpeglenatide_low_dosage:Empagliflozin_high_dosage | 0 | -0.045431548 | NA | -0.045431548 | NA | NA | NA | NA |
| 256 | Efpeglenatide_low_dosage:Empagliflozin_low_dosage | 0 | -0.037969433 | NA | -0.037969433 | NA | NA | NA | NA |
| 257 | Efpeglenatide_low_dosage:Ertugliflozin_high_dosage | 0 | -0.113180827 | NA | -0.113180827 | NA | NA | NA | NA |
| 258 | Efpeglenatide_low_dosage:Ertugliflozin_low_dosage | 0 | -0.005212632 | NA | -0.005212632 | NA | NA | NA | NA |
| 259 | Efpeglenatide_low_dosage:Exenatide | 0 | -0.29933513 | NA | -0.29933513 | NA | NA | NA | NA |
| 260 | Efpeglenatide_low_dosage:Inject_semaglutide_high_dosage | 0 | 0.088387633 | NA | 0.088387633 | NA | NA | NA | NA |
| 261 | Efpeglenatide_low_dosage:Inject_semaglutide_low_dosage | 0 | 0.085426178 | NA | 0.085426178 | NA | NA | NA | NA |
| 262 | Efpeglenatide_low_dosage:Inject_semaglutide_medium_dosage | 0 | 0.058346283 | NA | 0.058346283 | NA | NA | NA | NA |
| 263 | Efpeglenatide_low_dosage:Liraglutide | 0 | -0.106384288 | NA | -0.106384288 | NA | NA | NA | NA |
| 264 | Efpeglenatide_low_dosage:Lixisenatide | 0 | -0.184887784 | NA | -0.184887784 | NA | NA | NA | NA |
| 265 | Efpeglenatide_low_dosage:Oral_semaglutide | 0 | -0.26820632 | NA | -0.26820632 | NA | NA | NA | NA |
| 266 | Efpeglenatide_low_dosage:Placebo_or_Control | 2 | -0.233116326 | -0.255262548 | -0.143505165 | -0.111757383 | -1.313134326 | 1.089619559 | 0.855328075 |
| 267 | Efpeglenatide_low_dosage:Sotagliflozin | 0 | -0.074434351 | NA | -0.074434351 | NA | NA | NA | NA |
| 268 | Efpeglenatide_low_dosage:Tirzepatide_high_dosage | 0 | 0.093223852 | NA | 0.093223852 | NA | NA | NA | NA |
| 269 | Efpeglenatide_low_dosage:Tirzepatide_low_dosage | 0 | 0.216393605 | NA | 0.216393605 | NA | NA | NA | NA |
| 270 | Efpeglenatide_low_dosage:Tirzepatide_medium_dosage | 0 | 0.075595925 | NA | 0.075595925 | NA | NA | NA | NA |
| 271 | Efpeglenatide_medium_dosage:Empagliflozin_high_dosage | 0 | 0.138486519 | NA | 0.138486519 | NA | NA | NA | NA |
| 272 | Efpeglenatide_medium_dosage:Empagliflozin_low_dosage | 0 | 0.145948634 | NA | 0.145948634 | NA | NA | NA | NA |
| 273 | Efpeglenatide_medium_dosage:Ertugliflozin_high_dosage | 0 | 0.07073724 | NA | 0.07073724 | NA | NA | NA | NA |
| 274 | Efpeglenatide_medium_dosage:Ertugliflozin_low_dosage | 0 | 0.178705434 | NA | 0.178705434 | NA | NA | NA | NA |
| 275 | Efpeglenatide_medium_dosage:Exenatide | 0 | -0.115417064 | NA | -0.115417064 | NA | NA | NA | NA |
| 276 | Efpeglenatide_medium_dosage:Inject_semaglutide_high_dosage | 0 | 0.272305699 | NA | 0.272305699 | NA | NA | NA | NA |
| 277 | Efpeglenatide_medium_dosage:Inject_semaglutide_low_dosage | 0 | 0.269344244 | NA | 0.269344244 | NA | NA | NA | NA |
| 278 | Efpeglenatide_medium_dosage:Inject_semaglutide_medium_dosage | 0 | 0.24226435 | NA | 0.24226435 | NA | NA | NA | NA |
| 279 | Efpeglenatide_medium_dosage:Liraglutide | 0 | 0.077533779 | NA | 0.077533779 | NA | NA | NA | NA |
| 280 | Efpeglenatide_medium_dosage:Lixisenatide | 0 | -0.000969718 | NA | -0.000969718 | NA | NA | NA | NA |
| 281 | Efpeglenatide_medium_dosage:Oral_semaglutide | 0 | -0.084288254 | NA | -0.084288254 | NA | NA | NA | NA |
| 282 | Efpeglenatide_medium_dosage:Placebo_or_Control | 3 | -0.049198259 | -0.049399541 | NA | NA | NA | NA | NA |
| 283 | Efpeglenatide_medium_dosage:Sotagliflozin | 0 | 0.109483715 | NA | 0.109483715 | NA | NA | NA | NA |
| 284 | Efpeglenatide_medium_dosage:Tirzepatide_high_dosage | 0 | 0.277141918 | NA | 0.277141918 | NA | NA | NA | NA |
| 285 | Efpeglenatide_medium_dosage:Tirzepatide_low_dosage | 0 | 0.400311672 | NA | 0.400311672 | NA | NA | NA | NA |
| 286 | Efpeglenatide_medium_dosage:Tirzepatide_medium_dosage | 0 | 0.259513991 | NA | 0.259513991 | NA | NA | NA | NA |
| 287 | Empagliflozin_high_dosage:Empagliflozin_low_dosage | 7 | 0.007462115 | 0.040841477 | -0.082542888 | 0.123384365 | -0.368985076 | 0.615753805 | 0.623317975 |
| 288 | Empagliflozin_high_dosage:Ertugliflozin_high_dosage | 0 | -0.067749279 | NA | -0.067749279 | NA | NA | NA | NA |
| 289 | Empagliflozin_high_dosage:Ertugliflozin_low_dosage | 0 | 0.040218915 | NA | 0.040218915 | NA | NA | NA | NA |
| 290 | Empagliflozin_high_dosage:Exenatide | 0 | -0.253903582 | NA | -0.253903582 | NA | NA | NA | NA |
| 291 | Empagliflozin_high_dosage:Inject_semaglutide_high_dosage | 0 | 0.13381918 | NA | 0.13381918 | NA | NA | NA | NA |
| 292 | Empagliflozin_high_dosage:Inject_semaglutide_low_dosage | 0 | 0.130857726 | NA | 0.130857726 | NA | NA | NA | NA |
| 293 | Empagliflozin_high_dosage:Inject_semaglutide_medium_dosage | 0 | 0.103777831 | NA | 0.103777831 | NA | NA | NA | NA |
| 294 | Empagliflozin_high_dosage:Liraglutide | 0 | -0.06095274 | NA | -0.06095274 | NA | NA | NA | NA |
| 295 | Empagliflozin_high_dosage:Lixisenatide | 0 | -0.139456236 | NA | -0.139456236 | NA | NA | NA | NA |
| 296 | Empagliflozin_high_dosage:Oral_semaglutide | 1 | -0.222774772 | 0.770638797 | -0.496508862 | 1.26714766 | 0.340141861 | 2.194153458 | 0.007381498 |
| 297 | Empagliflozin_high_dosage:Placebo_or_Control | 8 | -0.187684778 | -0.308564638 | 0.477994967 | -0.786559605 | -1.346417051 | -0.226702159 | 0.005894214 |
| 298 | Empagliflozin_high_dosage:Sotagliflozin | 0 | -0.029002803 | NA | -0.029002803 | NA | NA | NA | NA |
| 299 | Empagliflozin_high_dosage:Tirzepatide_high_dosage | 0 | 0.138655399 | NA | 0.138655399 | NA | NA | NA | NA |
| 300 | Empagliflozin_high_dosage:Tirzepatide_low_dosage | 0 | 0.261825153 | NA | 0.261825153 | NA | NA | NA | NA |
| 301 | Empagliflozin_high_dosage:Tirzepatide_medium_dosage | 0 | 0.121027472 | NA | 0.121027472 | NA | NA | NA | NA |
| 302 | Empagliflozin_low_dosage:Ertugliflozin_high_dosage | 0 | -0.075211394 | NA | -0.075211394 | NA | NA | NA | NA |
| 303 | Empagliflozin_low_dosage:Ertugliflozin_low_dosage | 0 | 0.0327568 | NA | 0.0327568 | NA | NA | NA | NA |
| 304 | Empagliflozin_low_dosage:Exenatide | 0 | -0.261365697 | NA | -0.261365697 | NA | NA | NA | NA |
| 305 | Empagliflozin_low_dosage:Inject_semaglutide_high_dosage | 0 | 0.126357065 | NA | 0.126357065 | NA | NA | NA | NA |
| 306 | Empagliflozin_low_dosage:Inject_semaglutide_low_dosage | 0 | 0.123395611 | NA | 0.123395611 | NA | NA | NA | NA |
| 307 | Empagliflozin_low_dosage:Inject_semaglutide_medium_dosage | 0 | 0.096315716 | NA | 0.096315716 | NA | NA | NA | NA |
| 308 | Empagliflozin_low_dosage:Liraglutide | 0 | -0.068414855 | NA | -0.068414855 | NA | NA | NA | NA |
| 309 | Empagliflozin_low_dosage:Lixisenatide | 0 | -0.146918351 | NA | -0.146918351 | NA | NA | NA | NA |
| 310 | Empagliflozin_low_dosage:Oral_semaglutide | 0 | -0.230236887 | NA | -0.230236887 | NA | NA | NA | NA |
| 311 | Empagliflozin_low_dosage:Placebo_or_Control | 10 | -0.195146893 | -0.213796514 | 0.309935889 | -0.523732403 | -1.420542053 | 0.373077247 | 0.252371047 |
| 312 | Empagliflozin_low_dosage:Sotagliflozin | 0 | -0.036464918 | NA | -0.036464918 | NA | NA | NA | NA |
| 313 | Empagliflozin_low_dosage:Tirzepatide_high_dosage | 0 | 0.131193284 | NA | 0.131193284 | NA | NA | NA | NA |
| 314 | Empagliflozin_low_dosage:Tirzepatide_low_dosage | 0 | 0.254363038 | NA | 0.254363038 | NA | NA | NA | NA |
| 315 | Empagliflozin_low_dosage:Tirzepatide_medium_dosage | 0 | 0.113565357 | NA | 0.113565357 | NA | NA | NA | NA |
| 316 | Ertugliflozin_high_dosage:Ertugliflozin_low_dosage | 2 | 0.107968194 | 0.111766945 | NA | NA | NA | NA | NA |
| 317 | Ertugliflozin_high_dosage:Exenatide | 0 | -0.186154303 | NA | -0.186154303 | NA | NA | NA | NA |
| 318 | Ertugliflozin_high_dosage:Inject_semaglutide_high_dosage | 0 | 0.201568459 | NA | 0.201568459 | NA | NA | NA | NA |
| 319 | Ertugliflozin_high_dosage:Inject_semaglutide_low_dosage | 0 | 0.198607005 | NA | 0.198607005 | NA | NA | NA | NA |
| 320 | Ertugliflozin_high_dosage:Inject_semaglutide_medium_dosage | 0 | 0.17152711 | NA | 0.17152711 | NA | NA | NA | NA |
| 321 | Ertugliflozin_high_dosage:Liraglutide | 0 | 0.006796539 | NA | 0.006796539 | NA | NA | NA | NA |
| 322 | Ertugliflozin_high_dosage:Lixisenatide | 0 | -0.071706958 | NA | -0.071706958 | NA | NA | NA | NA |
| 323 | Ertugliflozin_high_dosage:Oral_semaglutide | 0 | -0.155025493 | NA | -0.155025493 | NA | NA | NA | NA |
| 324 | Ertugliflozin_high_dosage:Placebo_or_Control | 2 | -0.119935499 | -0.116389458 | NA | NA | NA | NA | NA |
| 325 | Ertugliflozin_high_dosage:Sotagliflozin | 0 | 0.038746476 | NA | 0.038746476 | NA | NA | NA | NA |
| 326 | Ertugliflozin_high_dosage:Tirzepatide_high_dosage | 0 | 0.206404678 | NA | 0.206404678 | NA | NA | NA | NA |
| 327 | Ertugliflozin_high_dosage:Tirzepatide_low_dosage | 0 | 0.329574432 | NA | 0.329574432 | NA | NA | NA | NA |
| 328 | Ertugliflozin_high_dosage:Tirzepatide_medium_dosage | 0 | 0.188776751 | NA | 0.188776751 | NA | NA | NA | NA |
| 329 | Ertugliflozin_low_dosage:Exenatide | 0 | -0.294122498 | NA | -0.294122498 | NA | NA | NA | NA |
| 330 | Ertugliflozin_low_dosage:Inject_semaglutide_high_dosage | 0 | 0.093600265 | NA | 0.093600265 | NA | NA | NA | NA |
| 331 | Ertugliflozin_low_dosage:Inject_semaglutide_low_dosage | 0 | 0.09063881 | NA | 0.09063881 | NA | NA | NA | NA |
| 332 | Ertugliflozin_low_dosage:Inject_semaglutide_medium_dosage | 0 | 0.063558916 | NA | 0.063558916 | NA | NA | NA | NA |
| 333 | Ertugliflozin_low_dosage:Liraglutide | 0 | -0.101171655 | NA | -0.101171655 | NA | NA | NA | NA |
| 334 | Ertugliflozin_low_dosage:Lixisenatide | 0 | -0.179675152 | NA | -0.179675152 | NA | NA | NA | NA |
| 335 | Ertugliflozin_low_dosage:Oral_semaglutide | 0 | -0.262993688 | NA | -0.262993688 | NA | NA | NA | NA |
| 336 | Ertugliflozin_low_dosage:Placebo_or_Control | 2 | -0.227903693 | -0.22681008 | -1.073921602 | 0.847111522 | -8.968507693 | 10.66273074 | 0.865678971 |
| 337 | Ertugliflozin_low_dosage:Sotagliflozin | 0 | -0.069221719 | NA | -0.069221719 | NA | NA | NA | NA |
| 338 | Ertugliflozin_low_dosage:Tirzepatide_high_dosage | 0 | 0.098436484 | NA | 0.098436484 | NA | NA | NA | NA |
| 339 | Ertugliflozin_low_dosage:Tirzepatide_low_dosage | 0 | 0.221606238 | NA | 0.221606238 | NA | NA | NA | NA |
| 340 | Ertugliflozin_low_dosage:Tirzepatide_medium_dosage | 0 | 0.080808557 | NA | 0.080808557 | NA | NA | NA | NA |
| 341 | Exenatide:Inject_semaglutide_high_dosage | 0 | 0.387722762 | NA | 0.387722762 | NA | NA | NA | NA |
| 342 | Exenatide:Inject_semaglutide_low_dosage | 0 | 0.384761308 | NA | 0.384761308 | NA | NA | NA | NA |
| 343 | Exenatide:Inject_semaglutide_medium_dosage | 1 | 0.357681413 | 0.18530941 | 0.437530373 | -0.252220963 | -0.997646594 | 0.493204667 | 0.507221607 |
| 344 | Exenatide:Liraglutide | 0 | 0.192950842 | NA | 0.192950842 | NA | NA | NA | NA |
| 345 | Exenatide:Lixisenatide | 0 | 0.114447346 | NA | 0.114447346 | NA | NA | NA | NA |
| 346 | Exenatide:Oral_semaglutide | 0 | 0.03112881 | NA | 0.03112881 | NA | NA | NA | NA |
| 347 | Exenatide:Placebo_or_Control | 2 | 0.066218804 | 0.108461204 | -0.143759759 | 0.252220963 | -0.493204667 | 0.997646594 | 0.507221607 |
| 348 | Exenatide:Sotagliflozin | 0 | 0.224900779 | NA | 0.224900779 | NA | NA | NA | NA |
| 349 | Exenatide:Tirzepatide_high_dosage | 0 | 0.392558982 | NA | 0.392558982 | NA | NA | NA | NA |
| 350 | Exenatide:Tirzepatide_low_dosage | 0 | 0.515728735 | NA | 0.515728735 | NA | NA | NA | NA |
| 351 | Exenatide:Tirzepatide_medium_dosage | 0 | 0.374931054 | NA | 0.374931054 | NA | NA | NA | NA |
| 352 | Inject_semaglutide_high_dosage:Inject_semaglutide_low_dosage | 0 | -0.002961455 | NA | -0.002961455 | NA | NA | NA | NA |
| 353 | Inject_semaglutide_high_dosage:Inject_semaglutide_medium_dosage | 1 | -0.030041349 | -0.002560821 | -0.03502244 | 0.032461619 | -0.907903523 | 0.972826761 | 0.946057563 |
| 354 | Inject_semaglutide_high_dosage:Liraglutide | 1 | -0.19477192 | -0.422272226 | -0.179639667 | -0.24263256 | -1.406253187 | 0.920988067 | 0.682772872 |
| 355 | Inject_semaglutide_high_dosage:Lixisenatide | 0 | -0.273275417 | NA | -0.273275417 | NA | NA | NA | NA |
| 356 | Inject_semaglutide_high_dosage:Oral_semaglutide | 0 | -0.356593953 | NA | -0.356593953 | NA | NA | NA | NA |
| 357 | Inject_semaglutide_high_dosage:Placebo_or_Control | 8 | -0.321503958 | -0.314207351 | -0.542975609 | 0.228768258 | -1.043304032 | 1.500840549 | 0.724479785 |
| 358 | Inject_semaglutide_high_dosage:Sotagliflozin | 0 | -0.162821984 | NA | -0.162821984 | NA | NA | NA | NA |
| 359 | Inject_semaglutide_high_dosage:Tirzepatide_high_dosage | 0 | 0.004836219 | NA | 0.004836219 | NA | NA | NA | NA |
| 360 | Inject_semaglutide_high_dosage:Tirzepatide_low_dosage | 0 | 0.128005973 | NA | 0.128005973 | NA | NA | NA | NA |
| 361 | Inject_semaglutide_high_dosage:Tirzepatide_medium_dosage | 0 | -0.012791708 | NA | -0.012791708 | NA | NA | NA | NA |
| 362 | Inject_semaglutide_low_dosage:Inject_semaglutide_medium_dosage | 3 | -0.027079895 | -0.053660884 | 0.019959708 | -0.073620592 | -0.898129167 | 0.750887984 | 0.861075148 |
| 363 | Inject_semaglutide_low_dosage:Liraglutide | 1 | -0.191810466 | -0.086644797 | -0.216268857 | 0.12962406 | -0.874364214 | 1.133612333 | 0.800230149 |
| 364 | Inject_semaglutide_low_dosage:Lixisenatide | 0 | -0.270313962 | NA | -0.270313962 | NA | NA | NA | NA |
| 365 | Inject_semaglutide_low_dosage:Oral_semaglutide | 0 | -0.353632498 | NA | -0.353632498 | NA | NA | NA | NA |
| 366 | Inject_semaglutide_low_dosage:Placebo_or_Control | 4 | -0.318542504 | -0.292263019 | -0.436877704 | 0.144614685 | -0.792773783 | 1.082003153 | 0.762368865 |
| 367 | Inject_semaglutide_low_dosage:Sotagliflozin | 0 | -0.159860529 | NA | -0.159860529 | NA | NA | NA | NA |
| 368 | Inject_semaglutide_low_dosage:Tirzepatide_high_dosage | 0 | 0.007797674 | NA | 0.007797674 | NA | NA | NA | NA |
| 369 | Inject_semaglutide_low_dosage:Tirzepatide_low_dosage | 0 | 0.130967427 | NA | 0.130967427 | NA | NA | NA | NA |
| 370 | Inject_semaglutide_low_dosage:Tirzepatide_medium_dosage | 0 | -0.009830253 | NA | -0.009830253 | NA | NA | NA | NA |
| 371 | Inject_semaglutide_medium_dosage:Liraglutide | 0 | -0.164730571 | NA | -0.164730571 | NA | NA | NA | NA |
| 372 | Inject_semaglutide_medium_dosage:Lixisenatide | 0 | -0.243234067 | NA | -0.243234067 | NA | NA | NA | NA |
| 373 | Inject_semaglutide_medium_dosage:Oral_semaglutide | 0 | -0.326552603 | NA | -0.326552603 | NA | NA | NA | NA |
| 374 | Inject_semaglutide_medium_dosage:Placebo_or_Control | 5 | -0.291462609 | -0.369289468 | -0.176411362 | -0.192878105 | -0.734076997 | 0.348320786 | 0.484856802 |
| 375 | Inject_semaglutide_medium_dosage:Sotagliflozin | 0 | -0.132780634 | NA | -0.132780634 | NA | NA | NA | NA |
| 376 | Inject_semaglutide_medium_dosage:Tirzepatide_high_dosage | 1 | 0.034877568 | 0.08679189 | 0.018454125 | 0.068337765 | -0.712368662 | 0.849044191 | 0.863781883 |
| 377 | Inject_semaglutide_medium_dosage:Tirzepatide_low_dosage | 1 | 0.158047322 | 0.333769969 | 0.10184001 | 0.231929959 | -0.584507305 | 1.048367223 | 0.577679067 |
| 378 | Inject_semaglutide_medium_dosage:Tirzepatide_medium_dosage | 1 | 0.017249641 | -0.040000216 | 0.036100092 | -0.076100308 | -0.845478764 | 0.693278147 | 0.846283311 |
| 379 | Liraglutide:Lixisenatide | 0 | -0.078503496 | NA | -0.078503496 | NA | NA | NA | NA |
| 380 | Liraglutide:Oral_semaglutide | 1 | -0.161822032 | 0.234032951 | -0.22677555 | 0.460808501 | -0.634260058 | 1.555877061 | 0.409508212 |
| 381 | Liraglutide:Placebo_or_Control | 10 | -0.126732038 | -0.141069702 | 0.345605138 | -0.48667484 | -1.530763986 | 0.557414306 | 0.36093446 |
| 382 | Liraglutide:Sotagliflozin | 0 | 0.031949937 | NA | 0.031949937 | NA | NA | NA | NA |
| 383 | Liraglutide:Tirzepatide_high_dosage | 0 | 0.199608139 | NA | 0.199608139 | NA | NA | NA | NA |
| 384 | Liraglutide:Tirzepatide_low_dosage | 0 | 0.322777893 | NA | 0.322777893 | NA | NA | NA | NA |
| 385 | Liraglutide:Tirzepatide_medium_dosage | 0 | 0.181980212 | NA | 0.181980212 | NA | NA | NA | NA |
| 386 | Lixisenatide:Oral_semaglutide | 0 | -0.083318536 | NA | -0.083318536 | NA | NA | NA | NA |
| 387 | Lixisenatide:Placebo_or_Control | 1 | -0.048228542 | -0.048228542 | NA | NA | NA | NA | NA |
| 388 | Lixisenatide:Sotagliflozin | 0 | 0.110453433 | NA | 0.110453433 | NA | NA | NA | NA |
| 389 | Lixisenatide:Tirzepatide_high_dosage | 0 | 0.278111636 | NA | 0.278111636 | NA | NA | NA | NA |
| 390 | Lixisenatide:Tirzepatide_low_dosage | 0 | 0.40128139 | NA | 0.40128139 | NA | NA | NA | NA |
| 391 | Lixisenatide:Tirzepatide_medium_dosage | 0 | 0.260483709 | NA | 0.260483709 | NA | NA | NA | NA |
| 392 | Oral_semaglutide:Placebo_or_Control | 6 | 0.035089994 | 0.257882876 | -0.828923405 | 1.086806281 | 0.23222107 | 1.941391492 | 0.012682747 |
| 393 | Oral_semaglutide:Sotagliflozin | 0 | 0.193771969 | NA | 0.193771969 | NA | NA | NA | NA |
| 394 | Oral_semaglutide:Tirzepatide_high_dosage | 0 | 0.361430172 | NA | 0.361430172 | NA | NA | NA | NA |
| 395 | Oral_semaglutide:Tirzepatide_low_dosage | 0 | 0.484599925 | NA | 0.484599925 | NA | NA | NA | NA |
| 396 | Oral_semaglutide:Tirzepatide_medium_dosage | 0 | 0.343802244 | NA | 0.343802244 | NA | NA | NA | NA |
| 397 | Sotagliflozin:Placebo_or_Control | 6 | -0.158681975 | -0.158681975 | NA | NA | NA | NA | NA |
| 398 | Tirzepatide_high_dosage:Placebo_or_Control | 7 | -0.326340177 | -0.395818166 | 0.07356995 | -0.469388115 | -1.145581933 | 0.206805702 | 0.173661412 |
| 399 | Tirzepatide_low_dosage:Placebo_or_Control | 5 | -0.449509931 | -0.398690742 | -0.645824441 | 0.247133699 | -0.404890727 | 0.899158126 | 0.457556895 |
| 400 | Tirzepatide_medium_dosage:Placebo_or_Control | 7 | -0.30871225 | -0.318810487 | -0.247108944 | -0.071701542 | -0.757845543 | 0.614442459 | 0.83771683 |
| 401 | Sotagliflozin:Tirzepatide_high_dosage | 0 | 0.167658203 | NA | 0.167658203 | NA | NA | NA | NA |
| 402 | Sotagliflozin:Tirzepatide_low_dosage | 0 | 0.290827956 | NA | 0.290827956 | NA | NA | NA | NA |
| 403 | Sotagliflozin:Tirzepatide_medium_dosage | 0 | 0.150030276 | NA | 0.150030276 | NA | NA | NA | NA |
| 404 | Tirzepatide_high_dosage:Tirzepatide_low_dosage | 7 | 0.123169754 | 0.059431248 | 0.706702825 | -0.647271577 | -1.557494574 | 0.262951421 | 0.163391228 |
| 405 | Tirzepatide_high_dosage:Tirzepatide_medium_dosage | 9 | -0.017627927 | -0.029021534 | 1.236576044 | -1.265597578 | -3.834334241 | 1.303139085 | 0.334214453 |
| 406 | Tirzepatide_low_dosage:Tirzepatide_medium_dosage | 7 | -0.140797681 | -0.181703712 | 0.335147834 | -0.516851546 | -1.51418414 | 0.480481049 | 0.30976286 |

**eTable 7I: Design-by-treatment model and loop inconsistency of primary outcome: overall gynecologic tumor**

| Inconsistency model | chi^2^ | *p* value of Prob>chi^2^ |
| --- | --- | --- |
| design-by-treatment | 11.01 | 0.9983 |
| loop inconsistency | 2.26 | 0.9442 |

*Abbreviation: 95%CIs: 95% confidence intervals; GLP-1 agonist: glucagon-like peptide-1 agonist; NA: not applicable; NMA: network meta-analysis; OR: odds ratio; RCT: randomized controlled trial; SGLT2 inhibitor: sodium–glucose cotransporter 2 inhibitor*

**eTable 8: Heterogeneity of primary outcome: overall gynecologic tumor**

|  | Heterogeneity statistic | degrees of freedom | *p* | *I squared* | *Tau-squared* | Treatments used |  |
| --- | --- | --- | --- | --- | --- | --- | --- |
| AC - AA | 1.92 | 6 | 0.927 | 0.00% | 0 | AA: | Placebo_or_Control |
| AD - AA | 0.28 | 3 | 0.965 | 0.00% | 0 | AB: | Liraglutide |
| AE - AA | 1.34 | 4 | 0.855 | 0.00% | 0 | AC: | Albiglutide |
| AE - AD | 0.97 | 2 | 0.614 | 0.00% | 0 | AD: | Canagliflozin_low_dosage |
| AF - AA | 1.15 | 2 | 0.562 | 0.00% | 0 | AE: | Canagliflozin_high_dosage |
| AG - AA | 1.89 | 2 | 0.388 | 0.00% | 0 | AF: | Efpeglenatide_medium_dosage |
| AG - AF | 0.44 | 2 | 0.802 | 0.00% | 0 | AG: | Efpeglenatide_high_dosage |
| AH - AA | 3.76 | 9 | 0.927 | 0.00% | 0 | AH: | Empagliflozin_low_dosage |
| AI - AA | 0.01 | 1 | 0.909 | 0.00% | 0 | AI: | Ertugliflozin_low_dosage |
| AJ - AA | 0 | 1 | 0.969 | 0.00% | 0 | AJ: | Ertugliflozin_high_dosage |
| AJ - AI | 0.01 | 1 | 0.939 | 0.00% | 0 | AK: | Dapagliflozin_high_dosage |
| AK - AA | 4.57 | 8 | 0.802 | 0.00% | 0 | AL: | Dulaglutide_medium_dosage |
| AL - AA | 3.75 | 5 | 0.586 | 0.00% | 0 | AM: | Dulaglutide_high_dosage |
| AB - AA | 2.69 | 9 | 0.975 | 0.00% | 0 | AN: | Sotagliflozin |
| AN - AA | 1.72 | 5 | 0.886 | 0.00% | 0 | AO: | Exenatide |
| AO - AA | 0.11 | 1 | 0.739 | 0.00% | 0 | AP: | Oral_semaglutide |
| AQ - AA | 2.99 | 7 | 0.885 | 0.00% | 0 | AQ: | Empagliflozin_high_dosage |
| AQ - AH | 2.24 | 6 | 0.896 | 0.00% | 0 | AR: | Lixisenatide |
| AR - AA | 0 | 0 | . | .% | 0 | AS: | Inject_semaglutide_low_dosage |
| AP - AA | 2.43 | 5 | 0.787 | 0.00% | 0 | AT: | Inject_semaglutide_medium_dosage |
| AS - AA | 0.52 | 3 | 0.914 | 0.00% | 0 | AU: | Dapagliflozin_low_dosage |
| AT - AA | 3.72 | 4 | 0.445 | 0.00% | 0 | AV: | Dapagliflozin_medium_dosage |
| AT - AS | 1.08 | 2 | 0.581 | 0.00% | 0 | AW: | Inject_semaglutide_high_dosage |
| AU - AA | 0.2 | 2 | 0.904 | 0.00% | 0 | AX: | Efpeglenatide_low_dosage |
| AV - AA | 0.04 | 2 | 0.979 | 0.00% | 0 | AY: | Tirzepatide_low_dosage |
| AU - AK | 1.6 | 2 | 0.449 | 0.00% | 0 | AZ: | Tirzepatide_medium_dosage |
| AV - AK | 0.36 | 2 | 0.836 | 0.00% | 0 | BA: | Tirzepatide_high_dosage |
| AV - AU | 0.29 | 2 | 0.866 | 0.00% | 0 | BB: | Bexagliflozin |
| BC - AA | 0.92 | 4 | 0.921 | 0.00% | 0 | BC: | Dulaglutide_low_dosage |
| BC - AL | 3.22 | 4 | 0.522 | 0.00% | 0 |  |  |
| AM - AA | 0 | 0 | . | .% | 0 |  |  |
| AM - AL | 0 | 0 | . | .% | 0 |  |  |
| BC - AM | 0 | 0 | . | .% | 0 |  |  |
| AW - AA | 3.09 | 7 | 0.876 | 0.00% | 0 |  |  |
| AW - AB | 0 | 0 | . | .% | 0 |  |  |
| AX - AA | 1.23 | 1 | 0.268 | 18.60% | 0.4246 |  |  |
| AX - AF | 0 | 1 | 0.951 | 0.00% | 0 |  |  |
| AX - AG | 0.28 | 1 | 0.6 | 0.00% | 0 |  |  |
| AW - AT | 0 | 0 | . | .% | 0 |  |  |
| AQ - AP | 0 | 0 | . | .% | 0 |  |  |
| AP - AB | 0 | 0 | . | .% | 0 |  |  |
| AT - AO | 0 | 0 | . | .% | 0 |  |  |
| AS - AB | 0 | 0 | . | .% | 0 |  |  |
| AZ - AA | 5.14 | 6 | 0.527 | 0.00% | 0 |  |  |
| BA - AA | 2.78 | 6 | 0.836 | 0.00% | 0 |  |  |
| BA - AZ | 6.4 | 8 | 0.602 | 0.00% | 0 |  |  |
| AY - AA | 0.43 | 4 | 0.98 | 0.00% | 0 |  |  |
| AZ - AY | 4.34 | 6 | 0.63 | 0.00% | 0 |  |  |
| BA - AY | 2.86 | 6 | 0.826 | 0.00% | 0 |  |  |
| AY - AT | 0 | 0 | . | .% | 0 |  |  |
| AZ - AT | 0 | 0 | . | .% | 0 |  |  |
| BA - AT | 0 | 0 | . | .% | 0 |  |  |
| BB - AA | 0 | 0 | . | .% | 0 |  |  |

*Abbreviation: 95%CIs: 95% confidence intervals; GLP-1 agonist: glucagon-like peptide-1 agonist; NA: not applicable; NMA: network meta-analysis; OR: odds ratio; RCT: randomized controlled trial; SGLT2 inhibitor: sodium–glucose cotransporter 2 inhibitor*

**eTable 9A: GRADE of primary outcome: overall gynecologic tumor**

|  | Comparison | No.Studies | Direct |  | Indirect |  | NMA |  |
| --- | --- | --- | --- | --- | --- | --- | --- | --- |
|  |  |  | Estimate | Rate | Estimate | Rate | Estimate | Rate |
| 1 | Albiglutide:Bexagliflozin | 0 |  |  | 0.749671276 | ⨁⨁○○ Low | 0.749671276 | ⨁⨁○○ Low |
| 2 | Albiglutide:Canagliflozin_high_dosage | 0 |  |  | -0.606237053 | ⨁⨁○○ Low | -0.606237053 | ⨁⨁○○ Low |
| 3 | Albiglutide:Canagliflozin_low_dosage | 0 |  |  | -0.252562453 | ⨁⨁○○ Low | -0.252562453 | ⨁⨁○○ Low |
| 4 | Albiglutide:Dapagliflozin_high_dosage | 0 |  |  | -0.106259785 | ⨁⨁○○ Low | -0.106259785 | ⨁⨁○○ Low |
| 5 | Albiglutide:Dapagliflozin_low_dosage | 0 |  |  | -0.680518547 | ⨁⨁○○ Low | -0.680518547 | ⨁⨁○○ Low |
| 6 | Albiglutide:Dapagliflozin_medium_dosage | 0 |  |  | 0.770545977 | ⨁⨁○○ Low | 0.770545977 | ⨁⨁○○ Low |
| 7 | Albiglutide:Dulaglutide_high_dosage | 0 |  |  | -0.665927094 | ⨁⨁○○ Low | -0.665927094 | ⨁⨁○○ Low |
| 8 | Albiglutide:Dulaglutide_low_dosage | 0 |  |  | -0.064466221 | ⨁⨁○○ Low | -0.064466221 | ⨁⨁○○ Low |
| 9 | Albiglutide:Dulaglutide_medium_dosage | 0 |  |  | -0.061814022 | ⨁⨁○○ Low | -0.061814022 | ⨁⨁○○ Low |
| 10 | Albiglutide:Efpeglenatide_high_dosage | 0 |  |  | -0.455753354 | ⨁⨁○○ Low | -0.455753354 | ⨁⨁○○ Low |
| 11 | Albiglutide:Efpeglenatide_low_dosage | 0 |  |  | -0.846793051 | ⨁⨁○○ Low | -0.846793051 | ⨁⨁○○ Low |
| 12 | Albiglutide:Efpeglenatide_medium_dosage | 0 |  |  | 0.403745457 | ⨁⨁○○ Low | 0.403745457 | ⨁⨁○○ Low |
| 13 | Albiglutide:Empagliflozin_high_dosage | 0 |  |  | -0.503526392 | ⨁⨁○○ Low | -0.503526392 | ⨁⨁○○ Low |
| 14 | Albiglutide:Empagliflozin_low_dosage | 0 |  |  | -0.343433514 | ⨁⨁○○ Low | -0.343433514 | ⨁⨁○○ Low |
| 15 | Albiglutide:Ertugliflozin_high_dosage | 0 |  |  | -0.395252349 | ⨁⨁○○ Low | -0.395252349 | ⨁⨁○○ Low |
| 16 | Albiglutide:Ertugliflozin_low_dosage | 0 |  |  | -0.416896987 | ⨁⨁○○ Low | -0.416896987 | ⨁⨁○○ Low |
| 17 | Albiglutide:Exenatide | 0 |  |  | -0.489505066 | ⨁⨁○○ Low | -0.489505066 | ⨁⨁○○ Low |
| 18 | Albiglutide:Inject_semaglutide_high_dosage | 0 |  |  | -0.062432768 | ⨁⨁○○ Low | -0.062432768 | ⨁⨁○○ Low |
| 19 | Albiglutide:Inject_semaglutide_low_dosage | 0 |  |  | 0.200755665 | ⨁⨁○○ Low | 0.200755665 | ⨁⨁○○ Low |
| 20 | Albiglutide:Inject_semaglutide_medium_dosage | 0 |  |  | -0.450757971 | ⨁⨁○○ Low | -0.450757971 | ⨁⨁○○ Low |
| 21 | Albiglutide:Liraglutide | 0 |  |  | -0.334010289 | ⨁⨁○○ Low | -0.334010289 | ⨁⨁○○ Low |
| 22 | Albiglutide:Lixisenatide | 0 |  |  | -1.616750869 | ⨁⨁○○ Low | -1.616750869 | ⨁⨁○○ Low |
| 23 | Albiglutide:Oral_semaglutide | 0 |  |  | 0.144388872 | ⨁⨁○○ Low | 0.144388872 | ⨁⨁○○ Low |
| 24 | Albiglutide:Placebo_or_Control | 7 | -0.207765097 | ⨁⨁⨁⨁ High |  |  | -0.207765097 | ⨁⨁⨁○ Medium |
| 25 | Albiglutide:Sotagliflozin | 0 |  |  | 0.324778788 | ⨁⨁○○ Low | 0.324778788 | ⨁⨁○○ Low |
| 26 | Albiglutide:Tirzepatide_high_dosage | 0 |  |  | -1.068762555 | ⨁⨁○○ Low | -1.068762555 | ⨁⨁○○ Low |
| 27 | Albiglutide:Tirzepatide_low_dosage | 0 |  |  | -0.866479499 | ⨁⨁○○ Low | -0.866479499 | ⨁⨁○○ Low |
| 28 | Albiglutide:Tirzepatide_medium_dosage | 0 |  |  | -0.727819392 | ⨁⨁○○ Low | -0.727819392 | ⨁⨁○○ Low |
| 29 | Bexagliflozin:Canagliflozin_high_dosage | 0 |  |  | -1.355908329 | ⨁⨁○○ Low | -1.355908329 | ⨁⨁○○ Low |
| 30 | Bexagliflozin:Canagliflozin_low_dosage | 0 |  |  | -1.002233729 | ⨁⨁○○ Low | -1.002233729 | ⨁⨁○○ Low |
| 31 | Bexagliflozin:Dapagliflozin_high_dosage | 0 |  |  | -0.855931061 | ⨁⨁○○ Low | -0.855931061 | ⨁⨁○○ Low |
| 32 | Bexagliflozin:Dapagliflozin_low_dosage | 0 |  |  | -1.430189823 | ⨁⨁○○ Low | -1.430189823 | ⨁⨁○○ Low |
| 33 | Bexagliflozin:Dapagliflozin_medium_dosage | 0 |  |  | 0.0208747 | ⨁⨁○○ Low | 0.0208747 | ⨁⨁○○ Low |
| 34 | Bexagliflozin:Dulaglutide_high_dosage | 0 |  |  | -1.41559837 | ⨁⨁○○ Low | -1.41559837 | ⨁⨁○○ Low |
| 35 | Bexagliflozin:Dulaglutide_low_dosage | 0 |  |  | -0.814137498 | ⨁⨁○○ Low | -0.814137498 | ⨁⨁○○ Low |
| 36 | Bexagliflozin:Dulaglutide_medium_dosage | 0 |  |  | -0.811485298 | ⨁⨁○○ Low | -0.811485298 | ⨁⨁○○ Low |
| 37 | Bexagliflozin:Efpeglenatide_high_dosage | 0 |  |  | -1.205424631 | ⨁⨁○○ Low | -1.205424631 | ⨁⨁○○ Low |
| 38 | Bexagliflozin:Efpeglenatide_low_dosage | 0 |  |  | -1.596464327 | ⨁⨁○○ Low | -1.596464327 | ⨁⨁○○ Low |
| 39 | Bexagliflozin:Efpeglenatide_medium_dosage | 0 |  |  | -0.345925819 | ⨁⨁○○ Low | -0.345925819 | ⨁⨁○○ Low |
| 40 | Bexagliflozin:Empagliflozin_high_dosage | 0 |  |  | -1.253197668 | ⨁⨁○○ Low | -1.253197668 | ⨁⨁○○ Low |
| 41 | Bexagliflozin:Empagliflozin_low_dosage | 0 |  |  | -1.09310479 | ⨁⨁○○ Low | -1.09310479 | ⨁⨁○○ Low |
| 42 | Bexagliflozin:Ertugliflozin_high_dosage | 0 |  |  | -1.144923626 | ⨁⨁○○ Low | -1.144923626 | ⨁⨁○○ Low |
| 43 | Bexagliflozin:Ertugliflozin_low_dosage | 0 |  |  | -1.166568264 | ⨁⨁○○ Low | -1.166568264 | ⨁⨁○○ Low |
| 44 | Bexagliflozin:Exenatide | 0 |  |  | -1.239176342 | ⨁⨁○○ Low | -1.239176342 | ⨁⨁○○ Low |
| 45 | Bexagliflozin:Inject_semaglutide_high_dosage | 0 |  |  | -0.812104045 | ⨁⨁○○ Low | -0.812104045 | ⨁⨁○○ Low |
| 46 | Bexagliflozin:Inject_semaglutide_low_dosage | 0 |  |  | -0.548915611 | ⨁⨁○○ Low | -0.548915611 | ⨁⨁○○ Low |
| 47 | Bexagliflozin:Inject_semaglutide_medium_dosage | 0 |  |  | -1.200429247 | ⨁⨁○○ Low | -1.200429247 | ⨁⨁○○ Low |
| 48 | Bexagliflozin:Liraglutide | 0 |  |  | -1.083681565 | ⨁⨁○○ Low | -1.083681565 | ⨁⨁○○ Low |
| 49 | Bexagliflozin:Lixisenatide | 0 |  |  | -2.366422146 | ⨁⨁○○ Low | -2.366422146 | ⨁⨁○○ Low |
| 50 | Bexagliflozin:Oral_semaglutide | 0 |  |  | -0.605282404 | ⨁⨁○○ Low | -0.605282404 | ⨁⨁○○ Low |
| 51 | Bexagliflozin:Placebo_or_Control | 1 | -0.957436373 | ⨁⨁⨁⨁ High |  |  | -0.957436373 | ⨁⨁⨁○ Medium |
| 52 | Bexagliflozin:Sotagliflozin | 0 |  |  | -0.424892488 | ⨁⨁○○ Low | -0.424892488 | ⨁⨁○○ Low |
| 53 | Bexagliflozin:Tirzepatide_high_dosage | 0 |  |  | -1.818433832 | ⨁⨁○○ Low | -1.818433832 | ⨁⨁○○ Low |
| 54 | Bexagliflozin:Tirzepatide_low_dosage | 0 |  |  | -1.616150775 | ⨁⨁○○ Low | -1.616150775 | ⨁⨁○○ Low |
| 55 | Bexagliflozin:Tirzepatide_medium_dosage | 0 |  |  | -1.477490668 | ⨁⨁○○ Low | -1.477490668 | ⨁⨁○○ Low |
| 56 | Canagliflozin_high_dosage:Canagliflozin_low_dosage | 3 | 0.41740782 | ⨁⨁⨁⨁ High | 0.200574286 | ⨁⨁⨁⨁ High | 0.3536746 | ⨁⨁⨁⨁ High |
| 57 | Canagliflozin_high_dosage:Dapagliflozin_high_dosage | 0 |  |  | 0.499977268 | ⨁⨁○○ Low | 0.499977268 | ⨁⨁○○ Low |
| 58 | Canagliflozin_high_dosage:Dapagliflozin_low_dosage | 0 |  |  | -0.074281494 | ⨁⨁○○ Low | -0.074281494 | ⨁⨁○○ Low |
| 59 | Canagliflozin_high_dosage:Dapagliflozin_medium_dosage | 0 |  |  | 1.376783029 | ⨁⨁○○ Low | 1.376783029 | ⨁⨁○○ Low |
| 60 | Canagliflozin_high_dosage:Dulaglutide_high_dosage | 0 |  |  | -0.059690041 | ⨁⨁○○ Low | -0.059690041 | ⨁⨁○○ Low |
| 61 | Canagliflozin_high_dosage:Dulaglutide_low_dosage | 0 |  |  | 0.541770831 | ⨁⨁○○ Low | 0.541770831 | ⨁⨁○○ Low |
| 62 | Canagliflozin_high_dosage:Dulaglutide_medium_dosage | 0 |  |  | 0.544423031 | ⨁⨁○○ Low | 0.544423031 | ⨁⨁○○ Low |
| 63 | Canagliflozin_high_dosage:Efpeglenatide_high_dosage | 0 |  |  | 0.150483698 | ⨁⨁○○ Low | 0.150483698 | ⨁⨁○○ Low |
| 64 | Canagliflozin_high_dosage:Efpeglenatide_low_dosage | 0 |  |  | -0.240555998 | ⨁⨁○○ Low | -0.240555998 | ⨁⨁○○ Low |
| 65 | Canagliflozin_high_dosage:Efpeglenatide_medium_dosage | 0 |  |  | 1.00998251 | ⨁⨁○○ Low | 1.00998251 | ⨁⨁○○ Low |
| 66 | Canagliflozin_high_dosage:Empagliflozin_high_dosage | 0 |  |  | 0.102710661 | ⨁⨁○○ Low | 0.102710661 | ⨁⨁○○ Low |
| 67 | Canagliflozin_high_dosage:Empagliflozin_low_dosage | 0 |  |  | 0.262803539 | ⨁⨁○○ Low | 0.262803539 | ⨁⨁○○ Low |
| 68 | Canagliflozin_high_dosage:Ertugliflozin_high_dosage | 0 |  |  | 0.210984703 | ⨁⨁○○ Low | 0.210984703 | ⨁⨁○○ Low |
| 69 | Canagliflozin_high_dosage:Ertugliflozin_low_dosage | 0 |  |  | 0.189340066 | ⨁⨁○○ Low | 0.189340066 | ⨁⨁○○ Low |
| 70 | Canagliflozin_high_dosage:Exenatide | 0 |  |  | 0.116731987 | ⨁⨁○○ Low | 0.116731987 | ⨁⨁○○ Low |
| 71 | Canagliflozin_high_dosage:Inject_semaglutide_high_dosage | 0 |  |  | 0.543804284 | ⨁⨁○○ Low | 0.543804284 | ⨁⨁○○ Low |
| 72 | Canagliflozin_high_dosage:Inject_semaglutide_low_dosage | 0 |  |  | 0.806992718 | ⨁⨁○○ Low | 0.806992718 | ⨁⨁○○ Low |
| 73 | Canagliflozin_high_dosage:Inject_semaglutide_medium_dosage | 0 |  |  | 0.155479082 | ⨁⨁○○ Low | 0.155479082 | ⨁⨁○○ Low |
| 74 | Canagliflozin_high_dosage:Liraglutide | 0 |  |  | 0.272226764 | ⨁⨁○○ Low | 0.272226764 | ⨁⨁○○ Low |
| 75 | Canagliflozin_high_dosage:Lixisenatide | 0 |  |  | -1.010513817 | ⨁⨁○○ Low | -1.010513817 | ⨁⨁○○ Low |
| 76 | Canagliflozin_high_dosage:Oral_semaglutide | 0 |  |  | 0.750625925 | ⨁⨁○○ Low | 0.750625925 | ⨁⨁○○ Low |
| 77 | Canagliflozin_high_dosage:Placebo_or_Control | 5 | 0.330652581 | ⨁⨁⨁⨁ High | 1.008832996 | ⨁⨁⨁⨁ High | 0.398471956 | ⨁⨁⨁⨁ High |
| 78 | Canagliflozin_high_dosage:Sotagliflozin | 0 |  |  | 0.931015841 | ⨁⨁○○ Low | 0.931015841 | ⨁⨁○○ Low |
| 79 | Canagliflozin_high_dosage:Tirzepatide_high_dosage | 0 |  |  | -0.462525503 | ⨁⨁○○ Low | -0.462525503 | ⨁⨁○○ Low |
| 80 | Canagliflozin_high_dosage:Tirzepatide_low_dosage | 0 |  |  | -0.260242446 | ⨁⨁○○ Low | -0.260242446 | ⨁⨁○○ Low |
| 81 | Canagliflozin_high_dosage:Tirzepatide_medium_dosage | 0 |  |  | -0.121582339 | ⨁⨁○○ Low | -0.121582339 | ⨁⨁○○ Low |
| 82 | Canagliflozin_low_dosage:Dapagliflozin_high_dosage | 0 |  |  | 0.146302668 | ⨁⨁○○ Low | 0.146302668 | ⨁⨁○○ Low |
| 83 | Canagliflozin_low_dosage:Dapagliflozin_low_dosage | 0 |  |  | -0.427956094 | ⨁⨁○○ Low | -0.427956094 | ⨁⨁○○ Low |
| 84 | Canagliflozin_low_dosage:Dapagliflozin_medium_dosage | 0 |  |  | 1.02310843 | ⨁⨁○○ Low | 1.02310843 | ⨁⨁○○ Low |
| 85 | Canagliflozin_low_dosage:Dulaglutide_high_dosage | 0 |  |  | -0.413364641 | ⨁⨁○○ Low | -0.413364641 | ⨁⨁○○ Low |
| 86 | Canagliflozin_low_dosage:Dulaglutide_low_dosage | 0 |  |  | 0.188096231 | ⨁⨁○○ Low | 0.188096231 | ⨁⨁○○ Low |
| 87 | Canagliflozin_low_dosage:Dulaglutide_medium_dosage | 0 |  |  | 0.190748431 | ⨁⨁○○ Low | 0.190748431 | ⨁⨁○○ Low |
| 88 | Canagliflozin_low_dosage:Efpeglenatide_high_dosage | 0 |  |  | -0.203190902 | ⨁⨁○○ Low | -0.203190902 | ⨁⨁○○ Low |
| 89 | Canagliflozin_low_dosage:Efpeglenatide_low_dosage | 0 |  |  | -0.594230598 | ⨁⨁○○ Low | -0.594230598 | ⨁⨁○○ Low |
| 90 | Canagliflozin_low_dosage:Efpeglenatide_medium_dosage | 0 |  |  | 0.65630791 | ⨁⨁○○ Low | 0.65630791 | ⨁⨁○○ Low |
| 91 | Canagliflozin_low_dosage:Empagliflozin_high_dosage | 0 |  |  | -0.250963939 | ⨁⨁○○ Low | -0.250963939 | ⨁⨁○○ Low |
| 92 | Canagliflozin_low_dosage:Empagliflozin_low_dosage | 0 |  |  | -0.090871061 | ⨁⨁○○ Low | -0.090871061 | ⨁⨁○○ Low |
| 93 | Canagliflozin_low_dosage:Ertugliflozin_high_dosage | 0 |  |  | -0.142689897 | ⨁⨁○○ Low | -0.142689897 | ⨁⨁○○ Low |
| 94 | Canagliflozin_low_dosage:Ertugliflozin_low_dosage | 0 |  |  | -0.164334534 | ⨁⨁○○ Low | -0.164334534 | ⨁⨁○○ Low |
| 95 | Canagliflozin_low_dosage:Exenatide | 0 |  |  | -0.236942613 | ⨁⨁○○ Low | -0.236942613 | ⨁⨁○○ Low |
| 96 | Canagliflozin_low_dosage:Inject_semaglutide_high_dosage | 0 |  |  | 0.190129684 | ⨁⨁○○ Low | 0.190129684 | ⨁⨁○○ Low |
| 97 | Canagliflozin_low_dosage:Inject_semaglutide_low_dosage | 0 |  |  | 0.453318118 | ⨁⨁○○ Low | 0.453318118 | ⨁⨁○○ Low |
| 98 | Canagliflozin_low_dosage:Inject_semaglutide_medium_dosage | 0 |  |  | -0.198195518 | ⨁⨁○○ Low | -0.198195518 | ⨁⨁○○ Low |
| 99 | Canagliflozin_low_dosage:Liraglutide | 0 |  |  | -0.081447836 | ⨁⨁○○ Low | -0.081447836 | ⨁⨁○○ Low |
| 100 | Canagliflozin_low_dosage:Lixisenatide | 0 |  |  | -1.364188417 | ⨁⨁○○ Low | -1.364188417 | ⨁⨁○○ Low |
| 101 | Canagliflozin_low_dosage:Oral_semaglutide | 0 |  |  | 0.396951325 | ⨁⨁○○ Low | 0.396951325 | ⨁⨁○○ Low |
| 102 | Canagliflozin_low_dosage:Placebo_or_Control | 2 | 0.094443668 | ⨁⨁⨁⨁ High | -0.337135638 | ⨁⨁⨁○ Medium | 0.044797356 | ⨁⨁⨁⨁ High |
| 103 | Canagliflozin_low_dosage:Sotagliflozin | 0 |  |  | 0.577341241 | ⨁⨁○○ Low | 0.577341241 | ⨁⨁○○ Low |
| 104 | Canagliflozin_low_dosage:Tirzepatide_high_dosage | 0 |  |  | -0.816200103 | ⨁⨁○○ Low | -0.816200103 | ⨁⨁○○ Low |
| 105 | Canagliflozin_low_dosage:Tirzepatide_low_dosage | 0 |  |  | -0.613917046 | ⨁⨁○○ Low | -0.613917046 | ⨁⨁○○ Low |
| 106 | Canagliflozin_low_dosage:Tirzepatide_medium_dosage | 0 |  |  | -0.475256939 | ⨁⨁○○ Low | -0.475256939 | ⨁⨁○○ Low |
| 107 | Dapagliflozin_high_dosage:Dapagliflozin_low_dosage | 3 | -0.432798864 | ⨁⨁⨁⨁ High | -0.735548697 | ⨁⨁⨁⨁ High | -0.574258762 | ⨁⨁⨁⨁ High |
| 108 | Dapagliflozin_high_dosage:Dapagliflozin_medium_dosage | 1 | 1.273661086 | ⨁⨁⨁⨁ High | 0.599296054 | ⨁⨁⨁⨁ High | 0.876805762 | ⨁⨁⨁⨁ High |
| 109 | Dapagliflozin_high_dosage:Dulaglutide_high_dosage | 0 |  |  | -0.559667309 | ⨁⨁○○ Low | -0.559667309 | ⨁⨁○○ Low |
| 110 | Dapagliflozin_high_dosage:Dulaglutide_low_dosage | 0 |  |  | 0.041793563 | ⨁⨁○○ Low | 0.041793563 | ⨁⨁○○ Low |
| 111 | Dapagliflozin_high_dosage:Dulaglutide_medium_dosage | 0 |  |  | 0.044445763 | ⨁⨁○○ Low | 0.044445763 | ⨁⨁○○ Low |
| 112 | Dapagliflozin_high_dosage:Efpeglenatide_high_dosage | 0 |  |  | -0.34949357 | ⨁⨁○○ Low | -0.34949357 | ⨁⨁○○ Low |
| 113 | Dapagliflozin_high_dosage:Efpeglenatide_low_dosage | 0 |  |  | -0.740533266 | ⨁⨁○○ Low | -0.740533266 | ⨁⨁○○ Low |
| 114 | Dapagliflozin_high_dosage:Efpeglenatide_medium_dosage | 0 |  |  | 0.510005242 | ⨁⨁○○ Low | 0.510005242 | ⨁⨁○○ Low |
| 115 | Dapagliflozin_high_dosage:Empagliflozin_high_dosage | 0 |  |  | -0.397266607 | ⨁⨁○○ Low | -0.397266607 | ⨁⨁○○ Low |
| 116 | Dapagliflozin_high_dosage:Empagliflozin_low_dosage | 0 |  |  | -0.237173729 | ⨁⨁○○ Low | -0.237173729 | ⨁⨁○○ Low |
| 117 | Dapagliflozin_high_dosage:Ertugliflozin_high_dosage | 0 |  |  | -0.288992565 | ⨁⨁○○ Low | -0.288992565 | ⨁⨁○○ Low |
| 118 | Dapagliflozin_high_dosage:Ertugliflozin_low_dosage | 0 |  |  | -0.310637202 | ⨁⨁○○ Low | -0.310637202 | ⨁⨁○○ Low |
| 119 | Dapagliflozin_high_dosage:Exenatide | 0 |  |  | -0.383245281 | ⨁⨁○○ Low | -0.383245281 | ⨁⨁○○ Low |
| 120 | Dapagliflozin_high_dosage:Inject_semaglutide_high_dosage | 0 |  |  | 0.043827016 | ⨁⨁○○ Low | 0.043827016 | ⨁⨁○○ Low |
| 121 | Dapagliflozin_high_dosage:Inject_semaglutide_low_dosage | 0 |  |  | 0.30701545 | ⨁⨁○○ Low | 0.30701545 | ⨁⨁○○ Low |
| 122 | Dapagliflozin_high_dosage:Inject_semaglutide_medium_dosage | 0 |  |  | -0.344498186 | ⨁⨁○○ Low | -0.344498186 | ⨁⨁○○ Low |
| 123 | Dapagliflozin_high_dosage:Liraglutide | 0 |  |  | -0.227750504 | ⨁⨁○○ Low | -0.227750504 | ⨁⨁○○ Low |
| 124 | Dapagliflozin_high_dosage:Lixisenatide | 0 |  |  | -1.510491085 | ⨁⨁○○ Low | -1.510491085 | ⨁⨁○○ Low |
| 125 | Dapagliflozin_high_dosage:Oral_semaglutide | 0 |  |  | 0.250648657 | ⨁⨁○○ Low | 0.250648657 | ⨁⨁○○ Low |
| 126 | Dapagliflozin_high_dosage:Placebo_or_Control | 7 | -0.108314548 | ⨁⨁⨁⨁ High | 0.532873739 | ⨁⨁⨁○ Medium | -0.101505312 | ⨁⨁⨁⨁ High |
| 127 | Dapagliflozin_high_dosage:Sotagliflozin | 0 |  |  | 0.431038573 | ⨁⨁○○ Low | 0.431038573 | ⨁⨁○○ Low |
| 128 | Dapagliflozin_high_dosage:Tirzepatide_high_dosage | 0 |  |  | -0.962502771 | ⨁⨁○○ Low | -0.962502771 | ⨁⨁○○ Low |
| 129 | Dapagliflozin_high_dosage:Tirzepatide_low_dosage | 0 |  |  | -0.760219714 | ⨁⨁○○ Low | -0.760219714 | ⨁⨁○○ Low |
| 130 | Dapagliflozin_high_dosage:Tirzepatide_medium_dosage | 0 |  |  | -0.621559607 | ⨁⨁○○ Low | -0.621559607 | ⨁⨁○○ Low |
| 131 | Dapagliflozin_low_dosage:Dapagliflozin_medium_dosage | 2 | 1.253299094 | ⨁⨁⨁⨁ High | 1.927664126 | ⨁⨁⨁⨁ High | 1.451064524 | ⨁⨁⨁⨁ High |
| 132 | Dapagliflozin_low_dosage:Dulaglutide_high_dosage | 0 |  |  | 0.014591453 | ⨁⨁○○ Low | 0.014591453 | ⨁⨁○○ Low |
| 133 | Dapagliflozin_low_dosage:Dulaglutide_low_dosage | 0 |  |  | 0.616052326 | ⨁⨁○○ Low | 0.616052326 | ⨁⨁○○ Low |
| 134 | Dapagliflozin_low_dosage:Dulaglutide_medium_dosage | 0 |  |  | 0.618704525 | ⨁⨁○○ Low | 0.618704525 | ⨁⨁○○ Low |
| 135 | Dapagliflozin_low_dosage:Efpeglenatide_high_dosage | 0 |  |  | 0.224765193 | ⨁⨁○○ Low | 0.224765193 | ⨁⨁○○ Low |
| 136 | Dapagliflozin_low_dosage:Efpeglenatide_low_dosage | 0 |  |  | -0.166274504 | ⨁⨁○○ Low | -0.166274504 | ⨁⨁○○ Low |
| 137 | Dapagliflozin_low_dosage:Efpeglenatide_medium_dosage | 0 |  |  | 1.084264004 | ⨁⨁○○ Low | 1.084264004 | ⨁⨁○○ Low |
| 138 | Dapagliflozin_low_dosage:Empagliflozin_high_dosage | 0 |  |  | 0.176992155 | ⨁⨁○○ Low | 0.176992155 | ⨁⨁○○ Low |
| 139 | Dapagliflozin_low_dosage:Empagliflozin_low_dosage | 0 |  |  | 0.337085033 | ⨁⨁○○ Low | 0.337085033 | ⨁⨁○○ Low |
| 140 | Dapagliflozin_low_dosage:Ertugliflozin_high_dosage | 0 |  |  | 0.285266198 | ⨁⨁○○ Low | 0.285266198 | ⨁⨁○○ Low |
| 141 | Dapagliflozin_low_dosage:Ertugliflozin_low_dosage | 0 |  |  | 0.26362156 | ⨁⨁○○ Low | 0.26362156 | ⨁⨁○○ Low |
| 142 | Dapagliflozin_low_dosage:Exenatide | 0 |  |  | 0.191013481 | ⨁⨁○○ Low | 0.191013481 | ⨁⨁○○ Low |
| 143 | Dapagliflozin_low_dosage:Inject_semaglutide_high_dosage | 0 |  |  | 0.618085779 | ⨁⨁○○ Low | 0.618085779 | ⨁⨁○○ Low |
| 144 | Dapagliflozin_low_dosage:Inject_semaglutide_low_dosage | 0 |  |  | 0.881274212 | ⨁⨁○○ Low | 0.881274212 | ⨁⨁○○ Low |
| 145 | Dapagliflozin_low_dosage:Inject_semaglutide_medium_dosage | 0 |  |  | 0.229760576 | ⨁⨁○○ Low | 0.229760576 | ⨁⨁○○ Low |
| 146 | Dapagliflozin_low_dosage:Liraglutide | 0 |  |  | 0.346508258 | ⨁⨁○○ Low | 0.346508258 | ⨁⨁○○ Low |
| 147 | Dapagliflozin_low_dosage:Lixisenatide | 0 |  |  | -0.936232322 | ⨁⨁○○ Low | -0.936232322 | ⨁⨁○○ Low |
| 148 | Dapagliflozin_low_dosage:Oral_semaglutide | 0 |  |  | 0.824907419 | ⨁⨁○○ Low | 0.824907419 | ⨁⨁○○ Low |
| 149 | Dapagliflozin_low_dosage:Placebo_or_Control | 2 | 0.883358288 | ⨁⨁⨁⨁ High | 0.242170002 | ⨁⨁⨁⨁ High | 0.47275345 | ⨁⨁⨁⨁ High |
[truncated: 257,826 more chars]
