# Supplementary material for: The gynecologic tumor risk related to GLP-1 receptor agonists and SGLT2 inhibitors use: a network meta-analysis of 91 randomized controlled trials
Source: J Hematol Oncol. 2025 Nov 27;18:109. doi: 10.1186/s13045-025-01750-x (PMC12661836; doi:10.1186/s13045-025-01750-x)
Supplement: Supplementary file 2 — Supplementary Material 2 [file 13045_2025_1750_MOESM2_ESM.docx]

**Table 1A: League table of the primary outcome: overall gynecologic tumor**

| Bexagliflozin | . | . | . | . | . | . | . | . | . | . | 0.38 [0.08; 1.73] | . | . | . | . | . | . | . | . | . | . | . | . | . | . | . | . | . |
| --- | --- | --- | --- | --- | --- | --- | --- | --- | --- | --- | --- | --- | --- | --- | --- | --- | --- | --- | --- | --- | --- | --- | --- | --- | --- | --- | --- | --- |
| 0.65 [0.13; 3.42] | Sotagliflozin | . | . | . | . | . | . | . | . | . | 0.59 [0.30; 1.16] | . | . | . | . | . | . | . | . | . | . | . | . | . | . | . | . | . |
| 1.02 [0.08; 13.29] | 1.56 [0.18; 13.88] | Dapagliflozin_  medium_dosage | . | . | . | . | . | . | 0.28 [0.01; 7.00] | . | . | . | . | . | . | . | . | . | . | . | . | 0.29 [0.03; 2.77] | . | . | . | . | . | . |
| 0.71 [0.09; 5.75] | 1.08 [0.22; 5.39] | 0.69 [0.05; 8.74] | Efpeglenatide_  medium_dosage | . | . | . | . | . | . | . | 0.71 [0.14; 3.74] | . | . | . | . | . | 0.38 [0.08; 1.69] | . | . | . | . | . | . | . | 0.36 [0.07; 1.94] | . | . | . |
| 0.58 [0.10; 3.48] | 0.88 [0.27; 2.90] | 0.57 [0.06; 5.61] | 0.82 [0.14; 4.71] | Inject_  semaglutide_  low_dosage | . | . | . | . | . | . | 0.84 [0.28; 2.56] | . | 0.14 [0.01; 1.40] | . | . | . | . | 0.61 [0.17; 2.18] | . | . | . | . | . | . | . | . | . | . |
| 0.55 [0.09; 3.14] | 0.83 [0.27; 2.56] | 0.53 [0.06; 5.12] | 0.77 [0.14; 4.24] | 0.95 [0.25; 3.53] | Oral_  semaglutide | . | . | . | . | . | 0.62 [0.23; 1.67] | . | 0.98 [0.10; 9.52] | . | . | . | . | . | . | 0.98 [0.06; 15.78] | . | . | . | . | . | . | . | . |
| 0.47 [0.09; 2.41] | 0.72 [0.29; 1.81] | 0.46 [0.05; 4.04] | 0.67 [0.14; 3.25] | 0.82 [0.26; 2.60] | 0.87 [0.29; 2.56] | Albiglutide | . | . | . | . | 0.81 [0.44; 1.51] | . | . | . | . | . | . | . | . | . | . | . | . | . | . | . | . | . |
| 0.44 [0.09; 2.10] | 0.68 [0.31; 1.47] | 0.44 [0.05; 3.58] | 0.63 [0.14; 2.81] | 0.77 [0.27; 2.18] | 0.81 [0.31; 2.13] | 0.94 [0.46; 1.93] | Dulaglutide_  medium_dosage | . | . | 0.71 [0.18; 2.79] | 0.85 [0.59; 1.23] | . | . | . | . | . | . | . | 0.62 [0.02; 15.54] | . | . | . | . | . | . | . | . | . |
| 0.44 [0.09; 2.14] | 0.68 [0.30; 1.53] | 0.43 [0.05; 3.64] | 0.63 [0.14; 2.88] | 0.77 [0.26; 2.24] | 0.81 [0.30; 2.20] | 0.94 [0.44; 2.02] | 1.00 [0.56; 1.78] | Inject_  semaglutide_  high_dosage | . | . | 0.90 [0.57; 1.42] | . | 0.47 [0.04; 5.27] | . | . | . | . | 0.18 [0.01; 3.78] | . | . | . | . | . | . | . | . | . | . |
| 0.42 [0.09; 1.97] | 0.65 [0.31; 1.36] | 0.42 [0.05; 3.28] | 0.60 [0.14; 2.65] | 0.74 [0.27; 2.04] | 0.78 [0.31; 1.98] | 0.90 [0.45; 1.78] | 0.96 [0.60; 1.53] | 0.96 [0.56; 1.63] | Dapagliflozin_  high_dosage | . | 0.90 [0.67; 1.20] | . | . | . | . | . | . | . | . | . | . | 0.65 [0.10; 4.15] | . | . | . | . | . | . |
| 0.44 [0.08; 2.57] | 0.68 [0.22; 2.10] | 0.43 [0.05; 4.18] | 0.63 [0.11; 3.47] | 0.77 [0.20; 2.90] | 0.81 [0.23; 2.88] | 0.94 [0.31; 2.81] | 1.00 [0.40; 2.51] | 1.00 [0.36; 2.74] | 1.04 [0.40; 2.69] | Dulaglutide_  low_dosage | 0.89 [0.30; 2.59] | . | . | . | . | . | . | . | 0.48 [0.02; 12.13] | . | . | . | . | . | . | . | . | . |
| 0.38 [0.08; 1.73] | 0.59 [0.30; 1.16] | 0.38 [0.05; 3.00] | 0.54 [0.13; 2.32] | 0.66 [0.25; 1.76] | 0.70 [0.29; 1.71] | 0.81 [0.44; 1.51] | 0.86 [0.60; 1.24] | 0.86 [0.55; 1.35] | 0.90 [0.68; 1.21] | 0.87 [0.35; 2.14] | Placebo_  or_Control | 0.91 [0.47; 1.76] | 0.90 [0.61; 1.33] | 0.83 [0.49; 1.41] | 0.83 [0.28; 2.48] | 0.81 [0.27; 2.43] | 0.61 [0.16; 2.37] | 0.73 [0.30; 1.74] | . | 0.74 [0.32; 1.73] | 0.74 [0.49; 1.12] | 0.41 [0.04; 4.02] | 0.72 [0.39; 1.32] | 0.63 [0.18; 2.20] | 0.37 [0.05; 2.85] | 0.84 [0.24; 2.91] | 0.54 [0.21; 1.43] | 0.24 [0.05; 1.15] |
| 0.37 [0.07; 1.88] | 0.56 [0.22; 1.41] | 0.36 [0.04; 3.14] | 0.52 [0.11; 2.52] | 0.64 [0.20; 2.02] | 0.67 [0.23; 1.99] | 0.78 [0.32; 1.87] | 0.83 [0.40; 1.70] | 0.83 [0.38; 1.78] | 0.86 [0.43; 1.72] | 0.83 [0.28; 2.48] | 0.96 [0.51; 1.78] | Canagliflozin_  low_dosage | . | . | . | . | . | . | . | . | . | . | 0.66 [0.29; 1.48] | . | . | . | . | . |
| 0.34 [0.07; 1.60] | 0.52 [0.24; 1.13] | 0.33 [0.04; 2.74] | 0.48 [0.11; 2.15] | 0.59 [0.21; 1.63] | 0.62 [0.24; 1.60] | 0.72 [0.35; 1.48] | 0.76 [0.45; 1.29] | 0.76 [0.43; 1.36] | 0.80 [0.49; 1.29] | 0.76 [0.29; 2.04] | 0.88 [0.60; 1.29] | 0.92 [0.44; 1.91] | Liraglutide | . | . | . | . | . | . | . | . | . | . | . | . | . | . | . |
| 0.34 [0.07; 1.64] | 0.51 [0.22; 1.20] | 0.33 [0.04; 2.78] | 0.47 [0.10; 2.21] | 0.58 [0.19; 1.74] | 0.61 [0.22; 1.69] | 0.71 [0.32; 1.58] | 0.75 [0.40; 1.41] | 0.76 [0.38; 1.48] | 0.79 [0.44; 1.41] | 0.76 [0.27; 2.13] | 0.87 [0.53; 1.45] | 0.91 [0.41; 2.03] | 0.99 [0.53; 1.87] | Empagliflozin_  low_dosage | . | . | . | . | . | 0.70 [0.28; 1.77] | . | . | . | . | . | . | . | . |
| 0.32 [0.05; 2.05] | 0.49 [0.13; 1.77] | 0.31 [0.03; 3.26] | 0.45 [0.07; 2.78] | 0.55 [0.13; 2.39] | 0.58 [0.14; 2.39] | 0.67 [0.19; 2.37] | 0.72 [0.23; 2.27] | 0.72 [0.22; 2.34] | 0.75 [0.24; 2.33] | 0.72 [0.17; 2.97] | 0.83 [0.28; 2.48] | 0.87 [0.25; 3.05] | 0.94 [0.29; 3.00] | 0.95 [0.28; 3.17] | Ertugliflozin_  high_dosage | 0.98 [0.34; 2.80] | . | . | . | . | . | . | . | . | . | . | . | . |
| 0.31 [0.05; 2.01] | 0.48 [0.13; 1.73] | 0.31 [0.03; 3.19] | 0.44 [0.07; 2.72] | 0.54 [0.12; 2.34] | 0.57 [0.14; 2.34] | 0.66 [0.19; 2.32] | 0.70 [0.22; 2.22] | 0.70 [0.21; 2.29] | 0.73 [0.24; 2.28] | 0.70 [0.17; 2.91] | 0.81 [0.27; 2.43] | 0.85 [0.24; 2.99] | 0.92 [0.29; 2.94] | 0.93 [0.28; 3.10] | 0.98 [0.34; 2.80] | Ertugliflozin_  low_dosage | . | . | . | . | . | . | . | . | . | . | . | . |
| 0.30 [0.04; 2.09] | 0.46 [0.11; 1.86] | 0.29 [0.03; 3.27] | 0.42 [0.11; 1.63] | 0.52 [0.11; 2.49] | 0.55 [0.12; 2.49] | 0.63 [0.16; 2.50] | 0.67 [0.19; 2.42] | 0.67 [0.18; 2.49] | 0.71 [0.20; 2.49] | 0.68 [0.15; 3.10] | 0.78 [0.23; 2.66] | 0.82 [0.21; 3.23] | 0.89 [0.25; 3.20] | 0.89 [0.24; 3.37] | 0.94 [0.18; 4.87] | 0.96 [0.19; 4.98] | Efpeglenatide_  high_dosage | . | . | . | . | . | . | . | 0.61 [0.14; 2.70] | . | . | . |
| 0.30 [0.06; 1.59] | 0.46 [0.17; 1.23] | 0.29 [0.03; 2.64] | 0.43 [0.08; 2.14] | 0.52 [0.18; 1.48] | 0.55 [0.18; 1.72] | 0.64 [0.25; 1.63] | 0.68 [0.31; 1.50] | 0.68 [0.30; 1.55] | 0.71 [0.33; 1.52] | 0.68 [0.22; 2.14] | 0.78 [0.39; 1.59] | 0.82 [0.32; 2.10] | 0.89 [0.40; 1.98] | 0.90 [0.38; 2.14] | 0.95 [0.26; 3.48] | 0.97 [0.26; 3.56] | 1.01 [0.24; 4.14] | Inject_  semaglutide_  medium_dosage | . | . | 1.44 [0.24; 8.74] | . | . | . | . | 0.22 [0.01; 4.51] | 0.35 [0.01; 8.59] | . |
| 0.24 [0.02; 3.91] | 0.37 [0.03; 4.23] | 0.24 [0.01; 5.41] | 0.34 [0.02; 5.37] | 0.42 [0.03; 5.28] | 0.44 [0.04; 5.41] | 0.51 [0.05; 5.76] | 0.55 [0.05; 5.59] | 0.55 [0.05; 5.90] | 0.57 [0.05; 6.01] | 0.55 [0.05; 5.60] | 0.63 [0.06; 6.54] | 0.66 [0.06; 7.41] | 0.72 [0.07; 7.65] | 0.72 [0.07; 7.90] | 0.76 [0.06; 10.06] | 0.78 [0.06; 10.28] | 0.81 [0.06; 11.33] | 0.81 [0.07; 9.25] | Dulaglutide_  high_dosage | . | . | . | . | . | . | . | . | . |
| 0.29 [0.05; 1.50] | 0.44 [0.17; 1.15] | 0.28 [0.03; 2.50] | 0.40 [0.08; 2.02] | 0.49 [0.15; 1.64] | 0.52 [0.18; 1.55] | 0.60 [0.24; 1.53] | 0.64 [0.29; 1.41] | 0.64 [0.28; 1.47] | 0.67 [0.32; 1.43] | 0.64 [0.21; 2.01] | 0.74 [0.37; 1.49] | 0.78 [0.31; 1.97] | 0.84 [0.38; 1.86] | 0.85 [0.42; 1.75] | 0.90 [0.25; 3.28] | 0.92 [0.25; 3.35] | 0.95 [0.23; 3.90] | 0.95 [0.35; 2.55] | 1.18 [0.10; 13.44] | Empagliflozin_  high_dosage | . | . | . | . | . | . | . | . |
| 0.29 [0.06; 1.38] | ***0.44 [0.20; 0.98]** | 0.28 [0.03; 2.35] | 0.41 [0.09; 1.86] | 0.50 [0.18; 1.43] | 0.53 [0.20; 1.41] | 0.61 [0.29; 1.29] | 0.65 [0.38; 1.13] | 0.65 [0.36; 1.20] | 0.68 [0.41; 1.13] | 0.65 [0.24; 1.76] | 0.75 [0.50; 1.14] | 0.79 [0.37; 1.66] | 0.86 [0.49; 1.50] | 0.86 [0.45; 1.66] | 0.91 [0.28; 2.93] | 0.93 [0.29; 2.99] | 0.97 [0.27; 3.52] | 0.96 [0.44; 2.11] | 1.19 [0.11; 12.77] | 1.01 [0.45; 2.27] | Exenatide | . | . | . | . | . | . | . |
| 0.24 [0.03; 1.83] | 0.37 [0.08; 1.68] | 0.23 [0.03; 1.58] | 0.34 [0.05; 2.49] | 0.41 [0.08; 2.22] | 0.44 [0.09; 2.23] | 0.51 [0.11; 2.27] | 0.54 [0.13; 2.21] | 0.54 [0.13; 2.27] | 0.56 [0.15; 2.18] | 0.54 [0.11; 2.77] | 0.62 [0.16; 2.44] | 0.65 [0.15; 2.92] | 0.71 [0.17; 2.92] | 0.71 [0.17; 3.06] | 0.75 [0.13; 4.32] | 0.77 [0.13; 4.42] | 0.80 [0.13; 5.00] | 0.79 [0.17; 3.69] | 0.99 [0.07; 14.73] | 0.84 [0.18; 3.87] | 0.83 [0.20; 3.43] | Dapagliflozin_  low_dosage | . | . | . | . | . | . |
| 0.26 [0.05; 1.29] | ***0.39 [0.16; 0.96]** | 0.25 [0.03; 2.18] | 0.36 [0.08; 1.74] | 0.45 [0.14; 1.39] | 0.47 [0.16; 1.36] | 0.55 [0.23; 1.27] | 0.58 [0.29; 1.15] | 0.58 [0.28; 1.20] | 0.61 [0.32; 1.16] | 0.58 [0.20; 1.70] | 0.67 [0.38; 1.19] | 0.70 [0.35; 1.39] | 0.76 [0.38; 1.52] | 0.77 [0.36; 1.65] | 0.81 [0.24; 2.79] | 0.83 [0.24; 2.85] | 0.86 [0.22; 3.33] | 0.86 [0.34; 2.13] | 1.06 [0.10; 11.76] | 0.90 [0.37; 2.22] | 0.89 [0.44; 1.80] | 1.08 [0.24; 4.74] | Canagliflozin_  high_dosage | . | . | . | . | . |
| 0.23 [0.04; 1.35] | 0.35 [0.11; 1.12] | 0.22 [0.02; 2.19] | 0.32 [0.06; 1.83] | 0.40 [0.10; 1.52] | 0.42 [0.11; 1.53] | 0.48 [0.16; 1.50] | 0.51 [0.19; 1.42] | 0.51 [0.18; 1.46] | 0.54 [0.20; 1.45] | 0.52 [0.14; 1.91] | 0.59 [0.23; 1.53] | 0.62 [0.20; 1.93] | 0.67 [0.24; 1.87] | 0.68 [0.23; 1.99] | 0.72 [0.17; 3.05] | 0.73 [0.17; 3.11] | 0.76 [0.16; 3.58] | 0.76 [0.24; 2.38] | 0.94 [0.08; 11.68] | 0.80 [0.25; 2.58] | 0.79 [0.28; 2.20] | 0.95 [0.18; 5.02] | 0.89 [0.29; 2.68] | Tirzepatide_  medium_dosage | . | 0.86 [0.23; 3.31] | 0.58 [0.22; 1.54] | . |
| 0.20 [0.03; 1.62] | 0.31 [0.06; 1.51] | 0.20 [0.02; 2.47] | 0.29 [0.07; 1.17] | 0.35 [0.06; 1.98] | 0.37 [0.07; 2.00] | 0.43 [0.09; 2.03] | 0.46 [0.10; 1.99] | 0.46 [0.10; 2.04] | 0.48 [0.11; 2.05] | 0.46 [0.08; 2.48] | 0.53 [0.13; 2.20] | 0.55 [0.12; 2.62] | 0.60 [0.14; 2.63] | 0.60 [0.13; 2.75] | 0.64 [0.11; 3.85] | 0.65 [0.11; 3.93] | 0.68 [0.19; 2.40] | 0.67 [0.14; 3.31] | 0.83 [0.05; 12.89] | 0.71 [0.15; 3.47] | 0.70 [0.16; 3.09] | 0.85 [0.12; 6.10] | 0.79 [0.17; 3.67] | 0.89 [0.16; 4.92] | Efpeglenatide_  low_dosage | . | . | . |
| 0.20 [0.03; 1.17] | ***0.30 [0.10; 0.96]** | 0.19 [0.02; 1.89] | 0.28 [0.05; 1.58] | 0.34 [0.09; 1.30] | 0.36 [0.10; 1.32] | 0.42 [0.14; 1.29] | 0.45 [0.16; 1.22] | 0.45 [0.16; 1.26] | 0.47 [0.18; 1.24] | 0.45 [0.12; 1.64] | 0.52 [0.20; 1.31] | 0.54 [0.18; 1.66] | 0.59 [0.21; 1.61] | 0.59 [0.21; 1.71] | 0.62 [0.15; 2.63] | 0.64 [0.15; 2.69] | 0.66 [0.14; 3.09] | 0.66 [0.22; 2.01] | 0.82 [0.07; 10.12] | 0.70 [0.22; 2.22] | 0.69 [0.25; 1.89] | 0.83 [0.16; 4.34] | 0.77 [0.26; 2.31] | 0.87 [0.33; 2.27] | 0.98 [0.18; 5.40] | Tirzepatide_  low_dosage | 0.85 [0.32; 2.28] | . |
| ***0.16 [0.03; 0.90]** | ***0.25 [0.09; 0.72]** | 0.16 [0.02; 1.48] | 0.23 [0.04; 1.22] | ***0.28 [0.08; 0.99]** | 0.30 [0.09; 1.00] | ***0.34 [0.12; 0.96]** | ***0.37 [0.15; 0.90]** | ***0.37 [0.14; 0.93]** | ***0.38 [0.16; 0.92]** | 0.37 [0.11; 1.24] | ***0.42 [0.19; 0.96]** | 0.44 [0.16; 1.24] | 0.48 [0.19; 1.19] | 0.48 [0.18; 1.27] | 0.51 [0.13; 2.01] | 0.52 [0.13; 2.05] | 0.54 [0.12; 2.37] | 0.54 [0.19; 1.52] | 0.67 [0.06; 7.95] | 0.57 [0.19; 1.67] | 0.56 [0.22; 1.40] | 0.68 [0.14; 3.34] | 0.63 [0.23; 1.72] | 0.71 [0.31; 1.64] | 0.80 [0.15; 4.17] | 0.82 [0.35; 1.89] | Tirzepatide_  high_dosage | . |
| ***0.09 [0.01; 0.82]** | ***0.14 [0.03; 0.78]** | 0.09 [0.01; 1.23] | 0.13 [0.02; 1.11] | 0.16 [0.03; 1.02] | 0.17 [0.03; 1.03] | 0.20 [0.04; 1.06] | 0.21 [0.04; 1.04] | 0.21 [0.04; 1.06] | 0.22 [0.05; 1.07] | 0.21 [0.04; 1.28] | 0.24 [0.05; 1.15] | 0.26 [0.05; 1.36] | 0.28 [0.06; 1.37] | 0.28 [0.05; 1.43] | 0.29 [0.04; 1.97] | 0.30 [0.05; 2.01] | 0.31 [0.04; 2.26] | 0.31 [0.06; 1.72] | 0.39 [0.02; 6.38] | 0.33 [0.06; 1.80] | 0.32 [0.07; 1.61] | 0.39 [0.05; 3.10] | 0.36 [0.07; 1.91] | 0.41 [0.07; 2.53] | 0.46 [0.06; 3.82] | 0.47 [0.08; 2.89] | 0.58 [0.10; 3.35] | Lixisenatide |

Data presents as OR [95%CIs]. Pairwise (upper-right portion) and network (lower-left portion) meta-analysis results are presented as estimate effect sizes for the outcome of events of overall gynecologic tumor. Interventions are reported in order of mean ranking of beneficially prophylactic effect on events of overall gynecologic tumor, and outcomes are expressed as odds ratio (OR) (95% confidence intervals) (95%CIs). For the pairwise meta-analyses, OR of less than 1 indicates that the treatment specified in the row got more beneficial effect than that specified in the column. For the network meta-analysis (NMA), OR of less than 1 indicates that the treatment specified in the column got more beneficial effect than that specified in the row. Bold results marked with * indicate statistical significance.

**Table 1B: League table of the primary outcome: subgroup of intra-uterus tumor**

| Inject_  semaglutide_  low_dosage | . | . | . | . | . | . | . | . | . | . | . | . | . | 0.30 [0.03; 2.66] | . | . | . | 0.18 [0.01; 3.82] | . | . | . | . | . | . | . |
| --- | --- | --- | --- | --- | --- | --- | --- | --- | --- | --- | --- | --- | --- | --- | --- | --- | --- | --- | --- | --- | --- | --- | --- | --- | --- |
| 0.63 [0.06; 6.74] | Liraglutide | . | . | . | . | . | . | . | . | . | . | . | . | 0.48 [0.18; 1.29] | . | . | . | . | . | . | . | . | . | . | . |
| 0.51 [0.04; 5.92] | 0.82 [0.18; 3.77] | Sotagliflozin | . | . | . | . | . | . | . | . | . | . | . | 0.58 [0.18; 1.85] | . | . | . | . | . | . | . | . | . | . | . |
| 0.59 [0.02; 14.84] | 0.94 [0.07; 12.65] | 1.15 [0.08; 16.53] | Ertugliflozin_  high_dosage | . | . | . | . | . | . | . | . | . | . | 0.51 [0.05; 5.60] | . | . | 0.32 [0.03; 3.08] | . | . | . | . | . | . | . | . |
| 0.47 [0.04; 5.57] | 0.75 [0.16; 3.58] | 0.92 [0.17; 4.88] | 0.80 [0.05; 11.78] | Orforglipron | . | . | . | . | . | 0.81 [0.17; 3.86] | . | . | . | 0.77 [0.16; 3.64] | . | . | . | . | . | . | . | . | . | . | . |
| 0.44 [0.04; 4.44] | 0.70 [0.19; 2.60] | 0.86 [0.20; 3.61] | 0.75 [0.06; 9.56] | 0.93 [0.21; 4.04] | Inject_  semaglutide_  high_dosage | . | . | . | . | . | . | . | . | 0.70 [0.29; 1.68] | . | . | . | 0.30 [0.01; 7.46] | . | . | . | . | . | . | . |
| 0.57 [0.02; 19.37] | 0.92 [0.05; 17.57] | 1.12 [0.06; 22.78] | 0.98 [0.02; 38.51] | 1.22 [0.06; 25.05] | 1.31 [0.07; 23.89] | Bexagliflozin | . | . | . | . | . | . | . | 0.52 [0.03; 8.33] | . | . | . | . | . | . | . | . | . | . | . |
| 0.66 [0.01; 53.97] | 1.06 [0.02; 55.67] | 1.29 [0.02; 71.12] | 1.12 [0.01; 104.09] | 1.40 [0.03; 77.91] | 1.50 [0.03; 76.60] | 1.15 [0.01; 131.20] | Efpeglenatide_  low_dosage | . | . | . | . | . | . | . | 0.36 [0.01; 8.95] | . | . | . | . | . | . | . | . | . | . |
| 0.37 [0.04; 3.53] | 0.59 [0.18; 1.94] | 0.73 [0.19; 2.72] | 0.63 [0.05; 7.60] | 0.79 [0.20; 3.05] | 0.85 [0.29; 2.44] | 0.65 [0.04; 11.18] | 0.56 [0.01; 27.55] | Dapagliflozin_  high_dosage | . | . | . | . | . | 0.80 [0.42; 1.51] | . | . | . | . | . | . | . | . | . | . | . |
| 0.38 [0.03; 4.26] | 0.61 [0.14; 2.66] | 0.74 [0.15; 3.64] | 0.65 [0.05; 9.04] | 0.80 [0.16; 4.06] | 0.86 [0.22; 3.44] | 0.66 [0.03; 13.06] | 0.58 [0.01; 31.08] | 1.02 [0.29; 3.60] | Albiglutide | . | . | . | . | 0.78 [0.26; 2.33] | . | . | . | . | . | . | . | . | . | . | . |
| 0.36 [0.04; 3.37] | 0.57 [0.18; 1.85] | 0.70 [0.19; 2.59] | 0.61 [0.05; 7.26] | 0.75 [0.23; 2.51] | 0.81 [0.28; 2.33] | 0.62 [0.04; 10.69] | 0.54 [0.01; 26.36] | 0.96 [0.39; 2.33] | 0.94 [0.27; 3.29] | Dulaglutide_  medium_dosage | . | . | . | 0.79 [0.41; 1.49] | . | . | . | . | . | . | . | . | . | . | . |
| 0.35 [0.03; 4.08] | 0.56 [0.12; 2.61] | 0.68 [0.13; 3.56] | 0.59 [0.04; 8.63] | 0.74 [0.14; 3.97] | 0.79 [0.18; 3.40] | 0.61 [0.03; 12.40] | 0.53 [0.01; 29.24] | 0.93 [0.24; 3.58] | 0.92 [0.18; 4.58] | 0.98 [0.26; 3.72] | Canagliflozin_  low_dosage | . | . | 0.98 [0.28; 3.49] | . | 0.62 [0.15; 2.59] | . | . | . | . | . | . | . | . | . |
| 0.34 [0.02; 5.38] | 0.54 [0.07; 3.98] | 0.66 [0.08; 5.30] | 0.58 [0.03; 11.12] | 0.72 [0.09; 5.87] | 0.77 [0.11; 5.29] | 0.59 [0.02; 15.53] | 0.51 [0.01; 34.52] | 0.91 [0.14; 5.74] | 0.89 [0.12; 6.88] | 0.95 [0.15; 5.96] | 0.97 [0.12; 7.92] | Oral_  semaglutide | . | 0.88 [0.16; 4.95] | . | . | . | . | . | . | . | . | . | . | . |
| 0.32 [0.02; 6.21] | 0.51 [0.05; 4.92] | 0.63 [0.06; 6.49] | 0.55 [0.02; 12.69] | 0.68 [0.06; 7.17] | 0.73 [0.08; 6.60] | 0.56 [0.02; 17.42] | 0.49 [0.01; 22.57] | 0.86 [0.10; 7.24] | 0.85 [0.08; 8.46] | 0.90 [0.11; 7.52] | 0.92 [0.09; 9.68] | 0.95 [0.07; 13.64] | Efpeglenatide_  high_dosage | 0.87 [0.09; 8.37] | 1.03 [0.11; 10.01] | . | . | . | . | . | . | . | . | . | . |
| 0.30 [0.03; 2.57] | 0.48 [0.18; 1.29] | 0.58 [0.18; 1.85] | 0.51 [0.05; 5.60] | 0.63 [0.19; 2.08] | 0.68 [0.29; 1.58] | 0.52 [0.03; 8.33] | 0.45 [0.01; 20.92] | 0.80 [0.42; 1.51] | 0.78 [0.26; 2.33] | 0.83 [0.45; 1.55] | 0.86 [0.26; 2.80] | 0.88 [0.16; 4.95] | 0.93 [0.12; 7.06] | Placebo_  or_Control | 1.01 [0.10; 9.76] | 0.66 [0.21; 2.05] | 0.63 [0.11; 3.79] | 0.67 [0.20; 2.29] | 0.69 [0.33; 1.47] | 0.28 [0.06; 1.35] | 0.80 [0.17; 3.83] | 0.33 [0.01; 8.05] | 0.28 [0.01; 7.15] | 0.32 [0.01; 8.00] | 0.24 [0.04; 1.46] |
| 0.23 [0.01; 4.70] | 0.38 [0.04; 3.76] | 0.46 [0.04; 4.96] | 0.40 [0.02; 9.58] | 0.50 [0.05; 5.47] | 0.53 [0.06; 5.05] | 0.41 [0.01; 13.12] | 0.36 [0.01; 8.95] | 0.63 [0.07; 5.54] | 0.62 [0.06; 6.46] | 0.66 [0.08; 5.77] | 0.67 [0.06; 7.39] | 0.69 [0.05; 10.35] | 0.73 [0.09; 5.85] | 0.79 [0.10; 6.31] | Efpeglenatide_  medium_dosage | . | . | . | . | . | . | . | . | . | . |
| 0.21 [0.02; 2.37] | 0.34 [0.08; 1.47] | 0.42 [0.09; 2.01] | 0.36 [0.03; 5.03] | 0.45 [0.09; 2.24] | 0.49 [0.12; 1.90] | 0.37 [0.02; 7.27] | 0.32 [0.01; 17.33] | 0.57 [0.17; 1.98] | 0.56 [0.12; 2.58] | 0.60 [0.17; 2.05] | 0.61 [0.18; 2.09] | 0.63 [0.08; 4.80] | 0.66 [0.07; 6.58] | 0.72 [0.25; 2.08] | 0.91 [0.09; 9.40] | Canagliflozin_  high_dosage | . | . | . | . | . | . | . | . | . |
| 0.19 [0.01; 3.11] | 0.30 [0.04; 2.33] | 0.37 [0.04; 3.10] | 0.32 [0.03; 3.08] | 0.40 [0.05; 3.43] | 0.43 [0.06; 3.11] | 0.33 [0.01; 8.92] | 0.28 [0.00; 19.67] | 0.51 [0.08; 3.38] | 0.49 [0.06; 4.03] | 0.53 [0.08; 3.51] | 0.54 [0.06; 4.63] | 0.56 [0.05; 6.69] | 0.59 [0.04; 8.78] | 0.63 [0.11; 3.79] | 0.80 [0.05; 12.47] | 0.88 [0.11; 7.10] | Ertugliflozin_  low_dosage | . | . | . | . | . | . | . | . |
| 0.20 [0.02; 1.95] | 0.32 [0.08; 1.30] | 0.39 [0.09; 1.79] | 0.34 [0.03; 4.58] | 0.42 [0.09; 2.00] | 0.46 [0.13; 1.59] | 0.35 [0.02; 6.64] | 0.30 [0.01; 15.95] | 0.54 [0.17; 1.74] | 0.53 [0.12; 2.29] | 0.56 [0.18; 1.80] | 0.58 [0.12; 2.69] | 0.59 [0.08; 4.33] | 0.62 [0.07; 5.96] | 0.67 [0.25; 1.80] | 0.85 [0.09; 8.52] | 0.94 [0.22; 4.01] | 1.07 [0.14; 8.23] | Inject_  semaglutide_  medium_dosage | 0.96 [0.06; 15.41] | . | . | . | . | 0.22 [0.01; 4.51] | 0.35 [0.01; 8.59] |
| 0.20 [0.02; 1.98] | 0.33 [0.10; 1.12] | 0.40 [0.10; 1.57] | 0.35 [0.03; 4.29] | 0.43 [0.11; 1.76] | 0.47 [0.15; 1.42] | 0.36 [0.02; 6.29] | 0.31 [0.01; 15.40] | 0.55 [0.21; 1.44] | 0.54 [0.15; 2.00] | 0.57 [0.22; 1.50] | 0.59 [0.15; 2.36] | 0.60 [0.09; 3.94] | 0.64 [0.07; 5.51] | 0.69 [0.33; 1.43] | 0.87 [0.10; 7.89] | 0.96 [0.26; 3.49] | 1.09 [0.16; 7.53] | 1.02 [0.32; 3.29] | Exenatide | . | . | . | . | . | . |
| 0.16 [0.01; 2.11] | 0.26 [0.05; 1.42] | 0.32 [0.05; 1.92] | 0.28 [0.02; 4.43] | 0.35 [0.06; 2.14] | 0.37 [0.07; 1.87] | 0.29 [0.01; 6.31] | 0.25 [0.00; 14.61] | 0.44 [0.10; 1.99] | 0.43 [0.08; 2.48] | 0.46 [0.10; 2.06] | 0.47 [0.08; 2.88] | 0.48 [0.05; 4.39] | 0.51 [0.04; 5.91] | 0.55 [0.14; 2.16] | 0.70 [0.06; 8.42] | 0.77 [0.14; 4.36] | 0.87 [0.09; 8.31] | 0.82 [0.15; 4.41] | 0.80 [0.17; 3.77] | Empagliflozin_  low_dosage | 0.60 [0.14; 2.55] | . | . | . | . |
| 0.16 [0.01; 2.02] | 0.25 [0.05; 1.35] | 0.31 [0.05; 1.83] | 0.27 [0.02; 4.24] | 0.34 [0.06; 2.03] | 0.36 [0.07; 1.77] | 0.28 [0.01; 6.05] | 0.24 [0.00; 14.04] | 0.43 [0.10; 1.89] | 0.42 [0.07; 2.36] | 0.45 [0.10; 1.96] | 0.46 [0.08; 2.74] | 0.47 [0.05; 4.19] | 0.49 [0.04; 5.65] | 0.53 [0.14; 2.05] | 0.68 [0.06; 8.05] | 0.75 [0.13; 4.15] | 0.85 [0.09; 7.94] | 0.79 [0.15; 4.19] | 0.78 [0.17; 3.58] | 0.97 [0.27; 3.44] | Empagliflozin_  high_dosage | . | . | . | . |
| 0.10 [0.00; 4.63] | 0.16 [0.01; 4.46] | 0.19 [0.01; 5.74] | 0.17 [0.00; 9.09] | 0.21 [0.01; 6.30] | 0.22 [0.01; 6.09] | 0.17 [0.00; 11.76] | 0.15 [0.00; 21.87] | 0.26 [0.01; 6.85] | 0.26 [0.01; 7.55] | 0.27 [0.01; 7.13] | 0.28 [0.01; 8.52] | 0.29 [0.01; 10.95] | 0.30 [0.01; 13.46] | 0.33 [0.01; 8.05] | 0.42 [0.01; 18.90] | 0.46 [0.02; 13.37] | 0.52 [0.01; 20.34] | 0.49 [0.02; 13.86] | 0.48 [0.02; 12.72] | 0.59 [0.02; 19.28] | 0.61 [0.02; 19.77] | Lixisenatide | . | . | . |
| 0.13 [0.01; 1.69] | 0.20 [0.03; 1.21] | 0.24 [0.04; 1.63] | 0.21 [0.01; 3.62] | 0.26 [0.04; 1.80] | 0.28 [0.05; 1.58] | 0.22 [0.01; 5.11] | 0.19 [0.00; 11.67] | 0.34 [0.07; 1.71] | 0.33 [0.05; 2.10] | 0.35 [0.07; 1.78] | 0.36 [0.05; 2.43] | 0.37 [0.04; 3.64] | 0.39 [0.03; 4.86] | 0.42 [0.09; 1.88] | 0.53 [0.04; 6.92] | 0.59 [0.09; 3.70] | 0.67 [0.06; 6.88] | 0.62 [0.12; 3.23] | 0.61 [0.12; 3.21] | 0.76 [0.10; 5.79] | 0.79 [0.11; 5.90] | 1.28 [0.04; 44.04] | Tirzepatide_  medium_dosage | 0.62 [0.10; 3.82] | 0.44 [0.12; 1.60] |
| ***0.06 [0.00; 0.85]** | ***0.10 [0.02; 0.61]** | ***0.13 [0.02; 0.82]** | 0.11 [0.01; 1.83] | ***0.14 [0.02; 0.91]** | ***0.15 [0.03; 0.79]** | 0.11 [0.00; 2.60] | 0.10 [0.00; 5.95] | ***0.17 [0.04; 0.86]** | 0.17 [0.03; 1.05] | ***0.18 [0.04; 0.89]** | 0.19 [0.03; 1.22] | 0.19 [0.02; 1.84] | 0.20 [0.02; 2.46] | ***0.22 [0.05; 0.94]** | 0.28 [0.02; 3.50] | 0.30 [0.05; 1.85] | 0.34 [0.03; 3.47] | 0.32 [0.07; 1.55] | 0.32 [0.06; 1.60] | 0.39 [0.05; 2.91] | 0.41 [0.06; 2.97] | 0.66 [0.02; 22.40] | 0.52 [0.14; 1.85] | Tirzepatide_  low_dosage | 1.12 [0.23; 5.48] |
| ***0.06 [0.01; 0.77]** | ***0.10 [0.02; 0.52]** | ***0.12 [0.02; 0.71]** | 0.11 [0.01; 1.67] | ***0.14 [0.02; 0.79]** | ***0.15 [0.03; 0.67]** | 0.11 [0.01; 2.38] | 0.10 [0.00; 5.55] | ***0.17 [0.04; 0.72]** | ***0.17 [0.03; 0.91]** | ***0.18 [0.04; 0.75]** | 0.18 [0.03; 1.06] | 0.19 [0.02; 1.63] | 0.20 [0.02; 2.21] | ***0.21 [0.06; 0.78]** | 0.27 [0.02; 3.15] | 0.30 [0.06; 1.60] | 0.34 [0.04; 3.10] | 0.32 [0.07; 1.36] | 0.31 [0.07; 1.36] | 0.39 [0.06; 2.55] | 0.40 [0.06; 2.60] | 0.66 [0.02; 20.71] | 0.51 [0.17; 1.51] | 0.99 [0.31; 3.18] | Tirzepatide_  high_dosage |

Data presents as OR [95%CIs]. Pairwise (upper-right portion) and network (lower-left portion) meta-analysis results are presented as estimate effect sizes for the outcome of events of intra-uterus tumor. Interventions are reported in order of mean ranking of beneficially prophylactic effect on events of intra-uterus tumor, and outcomes are expressed as odds ratio (OR) (95% confidence intervals) (95%CIs). For the pairwise meta-analyses, OR of less than 1 indicates that the treatment specified in the row got more beneficial effect than that specified in the column. For the network meta-analysis (NMA), OR of less than 1 indicates that the treatment specified in the column got more beneficial effect than that specified in the row. Bold results marked with * indicate statistical significance.

**Table 1C: League table of the primary outcome: subgroup of cervical tumor**

| Bexagliflozin | . | . | . | . | . | 0.17 [0.01; 4.25] | . | . | . | . | . | . | . | . | . |
| --- | --- | --- | --- | --- | --- | --- | --- | --- | --- | --- | --- | --- | --- | --- | --- |
| 0.62 [0.02; 24.66] | Dapagliflozin_  high_dosage | . | . | . | . | 0.28 [0.05; 1.71] | . | . | . | . | . | . | . | . | . |
| 0.51 [0.01; 29.50] | 0.82 [0.04; 17.93] | Tirzepatide_  medium_dosage | . | . | . | 0.34 [0.01; 8.36] | . | 0.33 [0.01; 8.15] | . | . | . | . | . | . | . |
| 0.51 [0.01; 29.43] | 0.82 [0.04; 17.89] | 1.00 [0.04; 24.56] | Tirzepatide_  low_dosage | . | . | 0.34 [0.01; 8.38] | . | 0.33 [0.01; 8.17] | . | . | . | . | . | . | . |
| 0.50 [0.01; 46.78] | 0.81 [0.02; 32.26] | 0.99 [0.02; 57.57] | 1.00 [0.02; 57.71] | Sotagliflozin | . | 0.34 [0.01; 8.39] | . | . | . | . | . | . | . | . | . |
| 0.33 [0.01; 15.99] | 0.53 [0.03; 9.17] | 0.64 [0.02; 17.99] | 0.65 [0.02; 18.04] | 0.65 [0.01; 31.59] | Inject_  semaglutide_  high_dosage | 0.53 [0.06; 4.77] | . | . | . | . | . | . | . | . | . |
| 0.17 [0.01; 4.25] | 0.28 [0.05; 1.71] | 0.34 [0.03; 4.12] | 0.34 [0.03; 4.13] | 0.34 [0.01; 8.39] | 0.53 [0.06; 4.77] | Placebo_  or_Control | 0.99 [0.06; 15.91] | 0.97 [0.06; 15.63] | 0.84 [0.26; 2.76] | 0.32 [0.01; 7.85] | 0.35 [0.01; 8.54] | 0.37 [0.04; 3.19] | 0.32 [0.01; 7.77] | 0.29 [0.01; 7.17] | 0.29 [0.03; 2.83] |
| 0.17 [0.00; 11.87] | 0.28 [0.01; 7.60] | 0.34 [0.01; 14.08] | 0.34 [0.01; 14.12] | 0.34 [0.00; 23.47] | 0.52 [0.02; 18.10] | 0.99 [0.06; 15.91] | Exenatide | . | . | . | . | . | . | . | . |
| 0.17 [0.00; 7.74] | 0.27 [0.02; 4.34] | 0.33 [0.03; 4.02] | 0.33 [0.03; 4.03] | 0.33 [0.01; 15.30] | 0.51 [0.02; 10.76] | 0.97 [0.12; 7.94] | 0.98 [0.03; 31.69] | Tirzepatide_  high_dosage | . | . | . | . | . | . | . |
| 0.14 [0.00; 4.43] | 0.23 [0.03; 2.05] | 0.29 [0.02; 4.53] | 0.29 [0.02; 4.55] | 0.29 [0.01; 8.74] | 0.44 [0.04; 5.42] | 0.84 [0.26; 2.76] | 0.85 [0.04; 17.27] | 0.86 [0.08; 9.62] | Dulaglutide_  medium_dosage | . | . | . | . | . | . |
| 0.10 [0.00; 4.94] | 0.16 [0.01; 2.87] | 0.19 [0.01; 5.59] | 0.19 [0.01; 5.60] | 0.19 [0.00; 9.76] | 0.30 [0.01; 7.03] | 0.56 [0.06; 5.45] | 0.57 [0.02; 20.41] | 0.58 [0.03; 12.72] | 0.67 [0.05; 8.69] | Empagliflozin_  low_dosage | . | . | . | . | 0.29 [0.03; 2.82] |
| 0.06 [0.00; 5.55] | 0.10 [0.00; 3.83] | 0.12 [0.00; 6.83] | 0.12 [0.00; 6.85] | 0.12 [0.00; 10.97] | 0.18 [0.00; 8.92] | 0.35 [0.01; 8.54] | 0.35 [0.01; 24.11] | 0.35 [0.01; 16.37] | 0.41 [0.01; 12.57] | 0.61 [0.01; 31.09] | Canagliflozin_  high_dosage | . | . | . | . |
| 0.06 [0.00; 3.04] | 0.10 [0.01; 1.72] | 0.13 [0.00; 3.40] | 0.13 [0.00; 3.41] | 0.13 [0.00; 6.01] | 0.20 [0.01; 4.26] | 0.37 [0.04; 3.19] | 0.38 [0.01; 12.49] | 0.38 [0.02; 7.69] | 0.44 [0.04; 5.15] | 0.66 [0.03; 14.97] | 1.08 [0.02; 51.18] | Liraglutide | . | . | . |
| 0.05 [0.00; 3.05] | 0.09 [0.00; 1.85] | 0.10 [0.00; 3.52] | 0.10 [0.00; 3.53] | 0.10 [0.00; 6.02] | 0.16 [0.01; 4.47] | 0.31 [0.03; 3.68] | 0.31 [0.01; 12.75] | 0.31 [0.01; 8.12] | 0.36 [0.02; 5.72] | 0.54 [0.02; 15.68] | 0.89 [0.02; 51.29] | 0.82 [0.03; 21.89] | Ertugliflozin_  low_dosage | 0.98 [0.10; 9.48] | . |
| 0.05 [0.00; 2.95] | 0.08 [0.00; 1.79] | 0.10 [0.00; 3.41] | 0.10 [0.00; 3.42] | 0.10 [0.00; 5.83] | 0.16 [0.01; 4.33] | 0.30 [0.02; 3.56] | 0.30 [0.01; 12.34] | 0.30 [0.01; 7.86] | 0.35 [0.02; 5.54] | 0.52 [0.02; 15.17] | 0.86 [0.01; 49.61] | 0.79 [0.03; 21.18] | 0.96 [0.13; 7.35] | Ertugliflozin_  high_dosage | . |
| 0.04 [0.00; 1.62] | ***0.06 [0.00; 0.89]** | 0.07 [0.00; 1.79] | 0.07 [0.00; 1.79] | 0.08 [0.00; 3.21] | 0.12 [0.01; 2.22] | 0.22 [0.03; 1.57] | 0.22 [0.01; 6.60] | 0.23 [0.01; 3.99] | 0.26 [0.03; 2.59] | 0.39 [0.05; 2.77] | 0.64 [0.01; 27.34] | 0.59 [0.03; 10.79] | 0.72 [0.03; 17.02] | 0.74 [0.03; 17.70] | Empagliflozin_  high_dosage |

Data presents as OR [95%CIs]. Pairwise (upper-right portion) and network (lower-left portion) meta-analysis results are presented as estimate effect sizes for the outcome of events of cervical tumor. Interventions are reported in order of mean ranking of beneficially prophylactic effect on events of cervical tumor, and outcomes are expressed as odds ratio (OR) (95% confidence intervals) (95%CIs). For the pairwise meta-analyses, OR of less than 1 indicates that the treatment specified in the row got more beneficial effect than that specified in the column. For the network meta-analysis (NMA), OR of less than 1 indicates that the treatment specified in the column got more beneficial effect than that specified in the row. Bold results marked with * indicate statistical significance.

**Table 1D: League table of the primary outcome: subgroup of ovarian tumor**

| Bexagliflozin | . | . | . | . | . | . | . | . | . | 0.17 [0.01; 4.25] | . | . | . | . | . | . | . | . | . | . | . | . |
| --- | --- | --- | --- | --- | --- | --- | --- | --- | --- | --- | --- | --- | --- | --- | --- | --- | --- | --- | --- | --- | --- | --- |
| 0.58 [0.01; 32.10] | Efpeglenatide_  medium_dosage | . | . | . | . | . | . | . | . | 0.33 [0.01; 8.48] | . | . | . | . | . | . | 0.18 [0.01; 3.76] | . | . | . | . | . |
| 0.58 [0.01; 32.01] | 1.00 [0.04; 23.19] | Efpeglenatide_  high_dosage | . | . | . | . | . | . | . | 0.28 [0.01; 7.17] | . | . | . | . | . | . | 0.20 [0.01; 4.39] | . | . | . | . | . |
| 0.52 [0.01; 28.31] | 0.90 [0.03; 27.01] | 0.91 [0.03; 27.06] | Inject_  semaglutide_  medium_dosage | . | . | . | . | . | . | 0.33 [0.01; 8.11] | . | . | . | . | . | 0.22 [0.01; 4.53] | . | . | . | . | . | . |
| 0.37 [0.01; 11.35] | 0.65 [0.04; 9.54] | 0.65 [0.04; 9.56] | 0.72 [0.05; 10.20] | Dulaglutide_  medium_dosage | . | . | . | . | . | 0.46 [0.13; 1.64] | . | . | . | 0.33 [0.03; 3.16] | . | . | . | . | . | . | . | . |
| 0.50 [0.01; 46.78] | 0.87 [0.02; 48.37] | 0.88 [0.02; 48.47] | 0.97 [0.02; 52.29] | 1.35 [0.04; 40.66] | Sotagliflozin | . | . | . | . | 0.34 [0.01; 8.39] | . | . | . | . | . | . | . | . | . | . | . | . |
| 0.37 [0.01; 15.55] | 0.64 [0.03; 14.14] | 0.64 [0.03; 14.16] | 0.71 [0.03; 15.17] | 0.99 [0.10; 9.39] | 0.73 [0.02; 30.73] | Tirzepatide_  medium_dosage | . | . | . | 0.34 [0.01; 8.36] | . | 0.33 [0.01; 8.17] | . | . | . | . | . | . | 0.35 [0.04; 3.35] | . | . | . |
| 0.38 [0.01; 16.89] | 0.65 [0.03; 15.54] | 0.66 [0.03; 15.57] | 0.72 [0.03; 16.69] | 1.01 [0.10; 10.59] | 0.75 [0.02; 33.37] | 1.02 [0.06; 16.90] | Canagliflozin_  low_dosage | 0.34 [0.01; 8.44] | . | 0.47 [0.06; 3.65] | . | . | . | . | . | . | . | . | . | . | . | . |
| 0.31 [0.01; 11.52] | 0.53 [0.03; 10.21] | 0.53 [0.03; 10.23] | 0.59 [0.03; 10.94] | 0.82 [0.10; 6.41] | 0.61 [0.02; 22.75] | 0.83 [0.06; 10.78] | 0.81 [0.07; 9.44] | Canagliflozin_  high_dosage | . | 0.50 [0.09; 2.80] | . | . | . | . | . | . | . | . | . | . | . | . |
| 0.27 [0.01; 14.56] | 0.47 [0.02; 13.84] | 0.48 [0.02; 13.87] | 0.52 [0.02; 14.42] | 0.73 [0.05; 10.07] | 0.54 [0.01; 28.77] | 0.74 [0.04; 15.44] | 0.72 [0.03; 16.26] | 0.89 [0.05; 16.12] | Oral_  semaglutide | 1.06 [0.04; 26.11] | 0.32 [0.01; 8.02] | . | . | . | . | . | . | . | . | . | . | . |
| 0.17 [0.01; 4.25] | 0.30 [0.03; 3.36] | 0.30 [0.03; 3.37] | 0.33 [0.03; 3.58] | 0.46 [0.14; 1.48] | 0.34 [0.01; 8.39] | 0.47 [0.07; 3.20] | 0.46 [0.06; 3.51] | 0.56 [0.10; 3.05] | 0.63 [0.06; 6.60] | Placebo_  or_Control | 1.16 [0.30; 4.52] | 0.98 [0.06; 15.67] | 0.78 [0.17; 3.49] | 0.71 [0.11; 4.53] | 0.63 [0.08; 5.12] | 0.39 [0.04; 3.51] | 0.67 [0.07; 6.17] | 0.61 [0.23; 1.65] | 0.49 [0.08; 3.12] | 0.50 [0.12; 1.99] | 0.34 [0.06; 2.07] | 0.11 [0.01; 2.02] |
| 0.15 [0.00; 4.63] | 0.26 [0.02; 3.92] | 0.26 [0.02; 3.92] | 0.29 [0.02; 3.85] | 0.40 [0.07; 2.18] | 0.30 [0.01; 9.14] | 0.40 [0.04; 3.97] | 0.40 [0.04; 4.28] | 0.49 [0.06; 3.94] | 0.55 [0.05; 5.72] | 0.87 [0.25; 2.97] | Liraglutide | . | . | . | . | 2.29 [0.09; 56.76] | . | . | . | . | . | . |
| 0.14 [0.00; 5.57] | 0.25 [0.01; 4.97] | 0.25 [0.01; 4.98] | 0.28 [0.01; 5.33] | 0.39 [0.05; 3.17] | 0.29 [0.01; 11.01] | 0.39 [0.06; 2.68] | 0.38 [0.03; 5.63] | 0.47 [0.04; 5.38] | 0.53 [0.03; 9.89] | 0.84 [0.14; 4.84] | 0.96 [0.11; 8.23] | Tirzepatide_  low_dosage | . | . | . | . | . | . | 0.71 [0.11; 4.51] | . | . | . |
| 0.13 [0.00; 4.62] | 0.23 [0.01; 4.02] | 0.23 [0.01; 4.02] | 0.26 [0.02; 4.30] | 0.36 [0.05; 2.40] | 0.27 [0.01; 9.13] | 0.36 [0.03; 4.17] | 0.36 [0.03; 4.47] | 0.44 [0.05; 4.19] | 0.49 [0.03; 7.96] | 0.78 [0.17; 3.49] | 0.90 [0.13; 6.25] | 0.93 [0.09; 9.37] | Empagliflozin_  low_dosage | . | . | . | . | . | . | . | . | . |
| 0.12 [0.00; 4.23] | 0.21 [0.01; 3.67] | 0.21 [0.01; 3.68] | 0.23 [0.01; 3.93] | 0.33 [0.07; 1.61] | 0.24 [0.01; 8.35] | 0.33 [0.03; 3.82] | 0.32 [0.03; 4.09] | 0.40 [0.04; 3.84] | 0.45 [0.03; 7.29] | 0.71 [0.16; 3.21] | 0.82 [0.12; 5.73] | 0.85 [0.08; 8.59] | 0.91 [0.11; 7.65] | Dulaglutide_  low_dosage | . | . | . | . | . | . | . | . |
| 0.11 [0.00; 4.99] | 0.19 [0.01; 4.62] | 0.19 [0.01; 4.63] | 0.21 [0.01; 4.96] | 0.29 [0.03; 3.19] | 0.21 [0.00; 9.86] | 0.29 [0.02; 5.05] | 0.29 [0.02; 5.34] | 0.35 [0.02; 5.22] | 0.40 [0.02; 9.22] | 0.63 [0.08; 5.12] | 0.72 [0.06; 8.24] | 0.75 [0.05; 11.57] | 0.81 [0.06; 10.63] | 0.89 [0.07; 11.74] | Albiglutide | . | . | . | . | . | . | . |
| 0.11 [0.00; 4.27] | 0.20 [0.01; 3.79] | 0.20 [0.01; 3.80] | 0.22 [0.02; 2.26] | 0.30 [0.04; 2.40] | 0.22 [0.01; 8.43] | 0.30 [0.02; 4.01] | 0.30 [0.02; 4.28] | 0.37 [0.03; 4.08] | 0.41 [0.02; 7.02] | 0.65 [0.12; 3.63] | 0.75 [0.11; 5.06] | 0.78 [0.07; 9.09] | 0.84 [0.09; 8.19] | 0.92 [0.09; 9.05] | 1.04 [0.07; 15.64] | Inject_  semaglutide_  low_dosage | . | . | . | . | . | . |
| 0.11 [0.00; 4.46] | 0.19 [0.02; 2.11] | 0.19 [0.02; 2.11] | 0.21 [0.01; 4.30] | 0.30 [0.03; 2.60] | 0.22 [0.01; 8.82] | 0.30 [0.02; 4.28] | 0.30 [0.02; 4.56] | 0.37 [0.03; 4.37] | 0.41 [0.02; 7.98] | 0.65 [0.11; 4.00] | 0.75 [0.08; 6.73] | 0.78 [0.06; 9.73] | 0.84 [0.08; 8.82] | 0.92 [0.09; 9.74] | 1.04 [0.06; 16.62] | 1.00 [0.08; 12.11] | Efpeglenatide_  low_dosage | . | . | . | . | . |
| 0.11 [0.00; 3.03] | 0.18 [0.01; 2.51] | 0.18 [0.01; 2.51] | 0.20 [0.02; 2.67] | 0.28 [0.06; 1.31] | 0.21 [0.01; 5.98] | 0.29 [0.03; 2.49] | 0.28 [0.03; 2.70] | 0.34 [0.05; 2.45] | 0.39 [0.03; 4.94] | 0.61 [0.23; 1.65] | 0.71 [0.15; 3.43] | 0.73 [0.10; 5.50] | 0.79 [0.13; 4.76] | 0.86 [0.14; 5.26] | 0.98 [0.10; 9.94] | 0.94 [0.13; 6.80] | 0.94 [0.12; 7.47] | Dapagliflozin_  high_dosage | . | . | . | . |
| 0.10 [0.00; 3.45] | 0.17 [0.01; 3.00] | 0.17 [0.01; 3.01] | 0.19 [0.01; 3.21] | 0.27 [0.04; 1.79] | 0.20 [0.01; 6.82] | 0.27 [0.05; 1.51] | 0.27 [0.02; 3.34] | 0.33 [0.03; 3.13] | 0.37 [0.02; 5.95] | 0.58 [0.13; 2.60] | 0.67 [0.10; 4.67] | 0.70 [0.16; 3.11] | 0.75 [0.09; 6.23] | 0.82 [0.10; 6.89] | 0.93 [0.07; 12.22] | 0.89 [0.09; 8.69] | 0.90 [0.09; 9.44] | 0.95 [0.16; 5.72] | Tirzepatide_  high_dosage | . | . | . |
| 0.09 [0.00; 2.81] | 0.15 [0.01; 2.42] | 0.15 [0.01; 2.42] | 0.16 [0.01; 2.59] | 0.23 [0.04; 1.40] | 0.17 [0.01; 5.56] | 0.23 [0.02; 2.48] | 0.23 [0.02; 2.67] | 0.28 [0.03; 2.49] | 0.31 [0.02; 4.79] | 0.50 [0.12; 1.99] | 0.57 [0.09; 3.66] | 0.59 [0.06; 5.56] | 0.64 [0.08; 4.92] | 0.70 [0.09; 5.44] | 0.79 [0.06; 9.78] | 0.76 [0.08; 6.90] | 0.76 [0.08; 7.51] | 0.81 [0.15; 4.46] | 0.85 [0.11; 6.56] | Exenatide | . | . |
| 0.06 [0.00; 2.32] | 0.10 [0.00; 2.08] | 0.10 [0.00; 2.08] | 0.11 [0.01; 2.23] | 0.16 [0.02; 1.34] | 0.12 [0.00; 4.58] | 0.16 [0.01; 2.22] | 0.15 [0.01; 2.36] | 0.19 [0.02; 2.27] | 0.21 [0.01; 4.14] | 0.34 [0.06; 2.07] | 0.39 [0.04; 3.49] | 0.41 [0.03; 5.04] | 0.44 [0.04; 4.57] | 0.48 [0.05; 5.05] | 0.54 [0.03; 8.62] | 0.52 [0.04; 6.28] | 0.52 [0.04; 6.77] | 0.55 [0.07; 4.35] | 0.58 [0.06; 6.09] | 0.68 [0.07; 6.67] | Inject_  semaglutide_  high_dosage | . |
| 0.02 [0.00; 1.44] | 0.03 [0.00; 1.45] | 0.03 [0.00; 1.45] | 0.04 [0.00; 1.56] | 0.05 [0.00; 1.17] | 0.04 [0.00; 2.84] | 0.05 [0.00; 1.68] | 0.05 [0.00; 1.75] | 0.06 [0.00; 1.79] | 0.07 [0.00; 2.91] | 0.11 [0.01; 2.02] | 0.13 [0.01; 2.99] | 0.13 [0.00; 3.94] | 0.14 [0.01; 3.74] | 0.15 [0.01; 4.12] | 0.17 [0.00; 6.33] | 0.17 [0.01; 4.94] | 0.17 [0.01; 5.23] | 0.18 [0.01; 3.89] | 0.19 [0.01; 4.98] | 0.22 [0.01; 5.57] | 0.32 [0.01; 9.99] | Lixisenatide |

Data presents as OR [95%CIs]. Pairwise (upper-right portion) and network (lower-left portion) meta-analysis results are presented as estimate effect sizes for the outcome of events of ovarian tumor. Interventions are reported in order of mean ranking of beneficially prophylactic effect on events of ovarian tumor, and outcomes are expressed as odds ratio (OR) (95% confidence intervals) (95%CIs). For the pairwise meta-analyses, OR of less than 1 indicates that the treatment specified in the row got more beneficial effect than that specified in the column. For the network meta-analysis (NMA), OR of less than 1 indicates that the treatment specified in the column got more beneficial effect than that specified in the row.

**Table 1E: League table of the primary outcome: subgroup of breast tumor**

| Dapagliflozin_  medium_dosage | . | . | . | . | . | 0.28 [0.01; 7.00] | . | . | . | . | . | . | . | . | . | . | . | . | . | . | 0.29 [0.03; 2.77] | . | . | . | . | . | . | . |
| --- | --- | --- | --- | --- | --- | --- | --- | --- | --- | --- | --- | --- | --- | --- | --- | --- | --- | --- | --- | --- | --- | --- | --- | --- | --- | --- | --- | --- |
| 0.60 [0.06; 6.08] | Oral_  semaglutide | . | . | . | . | . | . | 0.98 [0.06; 15.78] | . | . | 0.43 [0.14; 1.35] | . | . | . | . | . | . | 2.96 [0.12; 73.21] | . | . | . | . | . | . | . | . | . | . |
| 0.52 [0.06; 4.85] | 0.86 [0.24; 3.13] | Albiglutide | . | . | . | . | . | . | . | . | 0.67 [0.30; 1.51] | . | . | . | . | . | . | . | . | . | . | . | . | . | . | . | . | . |
| 0.59 [0.05; 7.51] | 0.98 [0.17; 5.77] | 1.13 [0.21; 6.02] | Inject_  semaglutide_  low_dosage | . | . | . | . | . | . | . | 0.83 [0.13; 5.30] | . | . | . | . | . | . | 0.05 [0.00; 1.18] | . | . | . | . | 0.50 [0.08; 3.10] | . | . | . | . | . |
| 0.49 [0.05; 4.71] | 0.82 [0.22; 3.09] | 0.95 [0.29; 3.09] | 0.84 [0.15; 4.58] | Sotagliflozin | . | . | . | . | . | . | 0.71 [0.30; 1.69] | . | . | . | . | . | . | . | . | . | . | . | . | . | . | . | . | . |
| 0.44 [0.05; 3.85] | 0.73 [0.23; 2.33] | 0.84 [0.31; 2.32] | 0.74 [0.15; 3.64] | 0.89 [0.31; 2.57] | Empagliflozin_  low_dosage | . | . | 1.09 [0.33; 3.61] | . | . | 0.76 [0.40; 1.43] | . | . | . | . | . | . | . | . | . | . | . | . | . | . | . | . | . |
| 0.43 [0.05; 3.36] | 0.71 [0.24; 2.07] | 0.82 [0.34; 1.99] | 0.72 [0.16; 3.28] | 0.86 [0.34; 2.22] | 0.97 [0.47; 2.00] | Dapagliflozin_  high_dosage | . | . | . | . | 0.81 [0.55; 1.19] | . | . | . | . | . | . | . | . | . | 0.65 [0.10; 4.15] | . | . | . | . | . | . | . |
| 0.42 [0.05; 3.57] | 0.69 [0.22; 2.16] | 0.80 [0.31; 2.10] | 0.71 [0.15; 3.35] | 0.85 [0.31; 2.32] | 0.95 [0.42; 2.13] | 0.98 [0.51; 1.87] | Dulaglutide_  medium_dosage | . | 0.74 [0.12; 4.75] | . | 0.84 [0.49; 1.43] | . | . | . | . | . | . | . | . | . | . | . | . | . | . | . | . | . |
| 0.41 [0.04; 3.99] | 0.69 [0.19; 2.44] | 0.80 [0.24; 2.65] | 0.70 [0.13; 3.90] | 0.84 [0.24; 2.90] | 0.94 [0.37; 2.40] | 0.97 [0.37; 2.56] | 0.99 [0.35; 2.79] | Empagliflozin_  high_dosage | . | . | 1.00 [0.34; 2.95] | . | . | . | . | . | . | . | . | . | . | . | . | . | . | . | . | . |
| 0.43 [0.04; 5.18] | 0.71 [0.13; 3.90] | 0.82 [0.17; 4.04] | 0.73 [0.10; 5.40] | 0.87 [0.17; 4.39] | 0.98 [0.22; 4.38] | 1.01 [0.24; 4.17] | 1.03 [0.26; 4.10] | 1.03 [0.20; 5.30] | Dulaglutide_  low_dosage | . | 0.71 [0.13; 3.99] | . | . | . | . | . | 0.48 [0.02; 12.13] | . | . | . | . | . | . | . | . | . | . | . |
| 0.39 [0.05; 3.39] | 0.65 [0.21; 2.05] | 0.76 [0.29; 2.00] | 0.67 [0.14; 3.15] | 0.80 [0.29; 2.21] | 0.90 [0.39; 2.04] | 0.92 [0.48; 1.79] | 0.94 [0.44; 2.01] | 0.95 [0.33; 2.69] | 0.92 [0.21; 4.01] | Inject_  semaglutide_  high_dosage | 0.92 [0.53; 1.60] | . | . | . | . | . | . | 0.47 [0.04; 5.27] | . | . | . | . | 0.30 [0.01; 7.46] | . | . | . | . | . |
| 0.35 [0.04; 2.82] | 0.58 [0.21; 1.59] | 0.67 [0.30; 1.51] | 0.60 [0.14; 2.57] | 0.71 [0.30; 1.69] | 0.80 [0.43; 1.48] | 0.82 [0.56; 1.20] | 0.84 [0.50; 1.42] | 0.85 [0.35; 2.06] | 0.82 [0.21; 3.22] | 0.89 [0.52; 1.54] | Placebo_  or_Control | 1.06 [0.07; 16.92] | 0.95 [0.21; 4.20] | 0.96 [0.09; 10.69] | 0.76 [0.15; 3.76] | 0.88 [0.21; 3.63] | . | 0.80 [0.49; 1.30] | 0.74 [0.42; 1.30] | 0.66 [0.11; 3.93] | 0.41 [0.04; 4.02] | 0.63 [0.26; 1.55] | 0.58 [0.17; 2.01] | 0.58 [0.14; 2.52] | 0.64 [0.15; 2.78] | 0.64 [0.29; 1.42] | 0.31 [0.03; 3.00] | 0.28 [0.05; 1.73] |
| 0.35 [0.02; 5.89] | 0.59 [0.07; 5.00] | 0.68 [0.09; 5.31] | 0.60 [0.05; 6.55] | 0.71 [0.09; 5.73] | 0.80 [0.11; 5.88] | 0.83 [0.12; 5.71] | 0.85 [0.12; 6.04] | 0.85 [0.11; 6.90] | 0.82 [0.08; 8.52] | 0.90 [0.13; 6.43] | 1.01 [0.15; 6.68] | Efpeglenatide_  medium_dosage | . | . | . | . | . | . | . | . | . | . | . | . | . | . | 0.32 [0.03; 3.10] | 0.27 [0.04; 1.69] |
| 0.33 [0.03; 4.30] | 0.55 [0.09; 3.33] | 0.64 [0.12; 3.47] | 0.56 [0.07; 4.55] | 0.67 [0.12; 3.77] | 0.76 [0.15; 3.79] | 0.78 [0.17; 3.63] | 0.80 [0.16; 3.86] | 0.80 [0.14; 4.54] | 0.77 [0.10; 5.86] | 0.84 [0.17; 4.12] | 0.95 [0.21; 4.20] | 0.94 [0.08; 10.47] | Ertugliflozin_  low_dosage | . | . | . | . | . | . | . | . | . | . | 0.62 [0.14; 2.66] | . | . | . | . |
| 0.34 [0.01; 8.15] | 0.56 [0.04; 7.63] | 0.65 [0.05; 8.23] | 0.57 [0.03; 9.59] | 0.68 [0.05; 8.84] | 0.77 [0.06; 9.23] | 0.79 [0.07; 9.07] | 0.81 [0.07; 9.52] | 0.82 [0.06; 10.62] | 0.79 [0.05; 12.58] | 0.86 [0.07; 10.13] | 0.96 [0.09; 10.69] | 0.96 [0.04; 20.49] | 1.02 [0.06; 17.26] | Bexagliflozin | . | . | . | . | . | . | . | . | . | . | . | . | . | . |
| 0.29 [0.03; 3.24] | 0.48 [0.10; 2.35] | 0.55 [0.13; 2.42] | 0.49 [0.07; 3.31] | 0.58 [0.13; 2.63] | 0.66 [0.17; 2.60] | 0.68 [0.19; 2.46] | 0.69 [0.18; 2.64] | 0.69 [0.15; 3.18] | 0.67 [0.11; 4.24] | 0.73 [0.19; 2.82] | 0.82 [0.24; 2.82] | 0.82 [0.09; 7.82] | 0.87 [0.13; 6.00] | 0.85 [0.06; 12.75] | Tirzepatide_  low_dosage | 0.86 [0.18; 4.19] | . | . | . | . | . | . | . | . | 0.81 [0.14; 4.56] | . | . | . |
| 0.26 [0.02; 2.79] | 0.43 [0.09; 1.98] | 0.50 [0.12; 2.02] | 0.44 [0.07; 2.82] | 0.53 [0.13; 2.20] | 0.59 [0.16; 2.16] | 0.61 [0.18; 2.03] | 0.62 [0.18; 2.19] | 0.63 [0.15; 2.67] | 0.61 [0.10; 3.61] | 0.66 [0.19; 2.34] | 0.74 [0.24; 2.32] | 0.74 [0.08; 6.72] | 0.78 [0.12; 5.11] | 0.77 [0.05; 11.04] | 0.90 [0.26; 3.10] | Tirzepatide_  high_dosage | . | . | . | . | . | . | . | . | 0.98 [0.21; 4.66] | . | . | . |
| 0.21 [0.00; 12.18] | 0.34 [0.01; 13.16] | 0.40 [0.01; 14.49] | 0.35 [0.01; 15.64] | 0.42 [0.01; 15.47] | 0.47 [0.01; 16.52] | 0.49 [0.01; 16.47] | 0.50 [0.01; 16.57] | 0.50 [0.01; 18.54] | 0.48 [0.02; 12.13] | 0.53 [0.02; 18.22] | 0.59 [0.02; 19.59] | 0.59 [0.01; 31.47] | 0.62 [0.01; 28.04] | 0.61 [0.01; 42.98] | 0.72 [0.02; 29.47] | 0.80 [0.02; 31.77] | Dulaglutide_  high_dosage | . | . | . | . | . | . | . | . | . | . | . |
| 0.27 [0.03; 2.32] | 0.46 [0.15; 1.36] | 0.53 [0.21; 1.34] | 0.47 [0.10; 2.10] | 0.56 [0.21; 1.49] | 0.62 [0.29; 1.35] | 0.64 [0.35; 1.18] | 0.66 [0.32; 1.33] | 0.66 [0.24; 1.81] | 0.64 [0.15; 2.72] | 0.70 [0.34; 1.42] | 0.78 [0.49; 1.25] | 0.78 [0.11; 5.47] | 0.83 [0.17; 3.94] | 0.81 [0.07; 9.43] | 0.95 [0.25; 3.57] | 1.06 [0.31; 3.64] | 1.32 [0.04; 45.24] | Liraglutide | . | . | . | . | . | . | . | . | . | . |
| 0.27 [0.03; 2.29] | 0.44 [0.14; 1.39] | 0.51 [0.19; 1.36] | 0.45 [0.10; 2.12] | 0.54 [0.19; 1.50] | 0.61 [0.26; 1.39] | 0.62 [0.32; 1.22] | 0.64 [0.30; 1.37] | 0.64 [0.22; 1.83] | 0.62 [0.14; 2.72] | 0.68 [0.31; 1.47] | 0.76 [0.43; 1.32] | 0.75 [0.10; 5.42] | 0.80 [0.16; 3.92] | 0.79 [0.07; 9.31] | 0.92 [0.24; 3.57] | 1.02 [0.29; 3.65] | 1.28 [0.04; 44.39] | 0.97 [0.47; 2.01] | Exenatide | . | . | . | 0.52 [0.05; 5.78] | . | . | . | . | . |
| 0.23 [0.01; 3.59] | 0.38 [0.05; 2.98] | 0.44 [0.06; 3.15] | 0.39 [0.04; 3.94] | 0.47 [0.06; 3.40] | 0.52 [0.08; 3.48] | 0.54 [0.09; 3.37] | 0.55 [0.09; 3.57] | 0.55 [0.08; 4.10] | 0.54 [0.06; 5.12] | 0.58 [0.09; 3.80] | 0.66 [0.11; 3.93] | 0.65 [0.05; 8.83] | 0.69 [0.07; 7.11] | 0.68 [0.03; 13.68] | 0.80 [0.09; 7.03] | 0.89 [0.11; 7.42] | 1.11 [0.02; 56.59] | 0.84 [0.13; 5.35] | 0.86 [0.13; 5.64] | Lixisenatide | . | . | . | . | . | . | . | . |
| 0.23 [0.03; 1.57] | 0.39 [0.07; 2.12] | 0.45 [0.09; 2.19] | 0.39 [0.05; 2.93] | 0.47 [0.09; 2.38] | 0.53 [0.12; 2.38] | 0.55 [0.14; 2.12] | 0.56 [0.13; 2.42] | 0.56 [0.11; 2.88] | 0.54 [0.08; 3.77] | 0.59 [0.13; 2.59] | 0.66 [0.17; 2.61] | 0.66 [0.06; 6.83] | 0.70 [0.09; 5.30] | 0.69 [0.04; 10.98] | 0.81 [0.13; 5.11] | 0.89 [0.15; 5.34] | 1.12 [0.03; 48.12] | 0.85 [0.20; 3.62] | 0.87 [0.20; 3.84] | 1.01 [0.11; 9.65] | Dapagliflozin_  low_dosage | . | . | . | . | . | . | . |
| 0.23 [0.02; 2.20] | 0.39 [0.11; 1.43] | 0.45 [0.14; 1.42] | 0.40 [0.07; 2.13] | 0.47 [0.14; 1.57] | 0.53 [0.19; 1.49] | 0.55 [0.22; 1.36] | 0.56 [0.21; 1.49] | 0.57 [0.17; 1.90] | 0.55 [0.11; 2.70] | 0.60 [0.22; 1.60] | 0.67 [0.29; 1.52] | 0.66 [0.08; 5.24] | 0.71 [0.13; 3.87] | 0.69 [0.05; 8.83] | 0.81 [0.18; 3.58] | 0.90 [0.22; 3.69] | 1.13 [0.03; 41.20] | 0.85 [0.33; 2.21] | 0.88 [0.33; 2.38] | 1.02 [0.14; 7.32] | 1.01 [0.20; 5.00] | Canagliflozin_  low_dosage | . | . | . | 0.76 [0.28; 2.10] | . | . |
| 0.22 [0.02; 2.18] | 0.36 [0.09; 1.49] | 0.41 [0.11; 1.51] | 0.37 [0.08; 1.65] | 0.44 [0.12; 1.65] | 0.49 [0.15; 1.60] | 0.51 [0.17; 1.49] | 0.52 [0.17; 1.61] | 0.52 [0.14; 2.00] | 0.50 [0.09; 2.75] | 0.55 [0.18; 1.68] | 0.61 [0.22; 1.68] | 0.61 [0.07; 5.22] | 0.65 [0.11; 3.92] | 0.64 [0.05; 8.67] | 0.75 [0.15; 3.68] | 0.83 [0.18; 3.81] | 1.04 [0.03; 39.70] | 0.79 [0.26; 2.37] | 0.81 [0.27; 2.44] | 0.94 [0.12; 7.32] | 0.93 [0.17; 5.10] | 0.92 [0.25; 3.38] | Inject_  semaglutide_  medium_dosage | . | . | . | . | . |
| 0.19 [0.02; 2.33] | 0.31 [0.05; 1.77] | 0.36 [0.07; 1.84] | 0.32 [0.04; 2.44] | 0.38 [0.07; 2.00] | 0.43 [0.09; 2.00] | 0.44 [0.10; 1.91] | 0.45 [0.10; 2.04] | 0.45 [0.08; 2.41] | 0.44 [0.06; 3.14] | 0.48 [0.10; 2.17] | 0.53 [0.13; 2.21] | 0.53 [0.05; 5.66] | 0.56 [0.14; 2.33] | 0.55 [0.03; 9.06] | 0.65 [0.10; 4.26] | 0.72 [0.12; 4.46] | 0.90 [0.02; 39.48] | 0.68 [0.15; 3.05] | 0.70 [0.15; 3.23] | 0.81 [0.08; 8.01] | 0.81 [0.11; 5.81] | 0.80 [0.16; 4.12] | 0.87 [0.15; 4.96] | Ertugliflozin_  high_dosage | . | . | . | . |
| 0.19 [0.02; 2.05] | 0.31 [0.07; 1.46] | 0.36 [0.09; 1.49] | 0.32 [0.05; 2.07] | 0.38 [0.09; 1.63] | 0.43 [0.12; 1.60] | 0.44 [0.13; 1.50] | 0.45 [0.13; 1.62] | 0.46 [0.11; 1.97] | 0.44 [0.07; 2.65] | 0.48 [0.13; 1.73] | 0.54 [0.17; 1.72] | 0.54 [0.06; 4.94] | 0.57 [0.09; 3.76] | 0.56 [0.04; 8.09] | 0.66 [0.18; 2.34] | 0.73 [0.22; 2.39] | 0.91 [0.02; 36.43] | 0.69 [0.20; 2.41] | 0.71 [0.20; 2.57] | 0.82 [0.10; 6.95] | 0.81 [0.13; 4.91] | 0.81 [0.19; 3.35] | 0.88 [0.19; 4.08] | 1.01 [0.16; 6.30] | Tirzepatide_  medium_dosage | . | . | . |
| 0.19 [0.02; 1.77] | 0.32 [0.09; 1.13] | 0.37 [0.12; 1.12] | 0.33 [0.06; 1.70] | 0.39 [0.12; 1.23] | 0.44 [0.17; 1.16] | 0.45 [0.19; 1.05] | 0.46 [0.18; 1.16] | 0.46 [0.14; 1.49] | 0.45 [0.09; 2.15] | 0.49 [0.19; 1.24] | 0.55 [0.26; 1.17] | 0.55 [0.07; 4.19] | 0.58 [0.11; 3.08] | 0.57 [0.05; 7.11] | 0.67 [0.16; 2.84] | 0.74 [0.19; 2.92] | 0.93 [0.03; 33.36] | 0.70 [0.29; 1.71] | 0.72 [0.28; 1.85] | 0.84 [0.12; 5.85] | 0.83 [0.17; 3.98] | 0.82 [0.34; 1.96] | 0.89 [0.25; 3.16] | 1.03 [0.21; 5.13] | 1.02 [0.25; 4.07] | Canagliflozin_  high_dosage | . | . |
| 0.11 [0.01; 1.49] | 0.18 [0.03; 1.20] | 0.20 [0.03; 1.26] | 0.18 [0.02; 1.61] | 0.21 [0.03; 1.36] | 0.24 [0.04; 1.38] | 0.25 [0.05; 1.33] | 0.25 [0.05; 1.41] | 0.25 [0.04; 1.64] | 0.25 [0.03; 2.08] | 0.27 [0.05; 1.51] | 0.30 [0.06; 1.55] | 0.30 [0.06; 1.54] | 0.32 [0.03; 2.91] | 0.31 [0.02; 5.74] | 0.37 [0.05; 2.85] | 0.41 [0.06; 2.99] | 0.51 [0.01; 24.25] | 0.38 [0.07; 2.12] | 0.40 [0.07; 2.24] | 0.46 [0.04; 5.20] | 0.45 [0.05; 3.85] | 0.45 [0.07; 2.82] | 0.49 [0.07; 3.35] | 0.56 [0.06; 4.91] | 0.56 [0.07; 4.15] | 0.55 [0.09; 3.33] | Efpeglenatide_  low_dosage | 0.96 [0.14; 6.70] |
| 0.10 [0.01; 1.27] | 0.16 [0.03; 1.00] | 0.18 [0.03; 1.04] | 0.16 [0.02; 1.36] | 0.19 [0.03; 1.13] | 0.22 [0.04; 1.14] | 0.22 [0.05; 1.09] | 0.23 [0.05; 1.16] | 0.23 [0.04; 1.36] | 0.22 [0.03; 1.75] | 0.24 [0.05; 1.24] | 0.27 [0.06; 1.27] | 0.27 [0.06; 1.26] | 0.29 [0.03; 2.45] | 0.28 [0.02; 4.92] | 0.33 [0.05; 2.38] | 0.37 [0.05; 2.50] | 0.46 [0.01; 21.11] | 0.35 [0.07; 1.74] | 0.36 [0.07; 1.85] | 0.42 [0.04; 4.41] | 0.41 [0.05; 3.24] | 0.41 [0.07; 2.34] | 0.44 [0.07; 2.79] | 0.51 [0.06; 4.13] | 0.51 [0.07; 3.47] | 0.50 [0.09; 2.76] | 0.91 [0.22; 3.81] | Efpeglenatide_  high_dosage |

Data presents as OR [95%CIs]. Pairwise (upper-right portion) and network (lower-left portion) meta-analysis results are presented as estimate effect sizes for the outcome of events of breast tumor. Interventions are reported in order of mean ranking of beneficially prophylactic effect on events of breast tumor, and outcomes are expressed as odds ratio (OR) (95% confidence intervals) (95%CIs). For the pairwise meta-analyses, OR of less than 1 indicates that the treatment specified in the row got more beneficial effect than that specified in the column. For the network meta-analysis (NMA), OR of less than 1 indicates that the treatment specified in the column got more beneficial effect than that specified in the row. Bold results marked with * indicate statistical significance.

**Table 1F: League table of the primary outcome: subgroup of vaginal tumor**

| Placebo_or_Control | 0.67 [0.01; 50.92] | 0.60 [0.03; 12.12] | 0.34 [0.00; 25.52] |
| --- | --- | --- | --- |
| 0.67 [0.01; 50.92] | Liraglutide | . | . |
| 0.60 [0.03; 12.12] | 0.90 [0.00; 174.33] | Dapagliflozin_high_dosage | . |
| 0.34 [0.00; 25.52] | 0.50 [0.00; 228.43] | 0.56 [0.00; 107.75] | Dulaglutide_medium_dosage |

Data presents as OR [95%CIs]. Pairwise (upper-right portion) and network (lower-left portion) meta-analysis results are presented as estimate effect sizes for the outcome of events of vaginal tumor. Interventions are reported in order of mean ranking of beneficially prophylactic effect on events of vaginal tumor, and outcomes are expressed as odds ratio (OR) (95% confidence intervals) (95%CIs). For the pairwise meta-analyses, OR of less than 1 indicates that the treatment specified in the row got more beneficial effect than that specified in the column. For the network meta-analysis (NMA), OR of less than 1 indicates that the treatment specified in the column got more beneficial effect than that specified in the row. Bold results marked with * indicate statistical significance.

**Table 1G: League table of the primary outcome: subgroup of vulvar tumor**

| Exenatide | . | . | . | . | 0.20 [0.01; 4.19] | . | . | . | . | . |
| --- | --- | --- | --- | --- | --- | --- | --- | --- | --- | --- |
| 0.63 [0.01; 51.86] | Canagliflozin_  low_dosage | . | . | . | 0.32 [0.01; 7.86] | . | . | . | . | . |
| 0.59 [0.01; 49.10] | 0.95 [0.01; 87.69] | Ertugliflozin_  high_dosage | . | . | 0.34 [0.01; 8.30] | . | . | . | . | . |
| 0.57 [0.01; 47.27] | 0.91 [0.01; 84.43] | 0.96 [0.01; 89.16] | Ertugliflozin_  low_dosage | . | 0.35 [0.01; 8.62] | . | . | . | . | . |
| 0.40 [0.01; 19.04] | 0.63 [0.01; 34.55] | 0.67 [0.01; 36.49] | 0.69 [0.01; 37.90] | Liraglutide | 0.51 [0.05; 5.59] | . | . | . | . | . |
| 0.20 [0.01; 4.19] | 0.32 [0.01; 7.86] | 0.34 [0.01; 8.30] | 0.35 [0.01; 8.62] | 0.51 [0.05; 5.59] | Placebo_  or_Control | 0.97 [0.06; 15.57] | 0.55 [0.12; 2.64] | 0.34 [0.01; 8.26] | 0.20 [0.01; 4.21] | 0.14 [0.01; 2.76] |
| 0.20 [0.00; 11.94] | 0.31 [0.00; 21.50] | 0.33 [0.00; 22.70] | 0.34 [0.00; 23.58] | 0.49 [0.01; 19.31] | 0.97 [0.06; 15.57] | Albiglutide | . | . | . | . |
| 0.11 [0.00; 3.38] | 0.18 [0.01; 6.23] | 0.19 [0.01; 6.58] | 0.19 [0.01; 6.84] | 0.28 [0.02; 4.91] | 0.55 [0.12; 2.64] | 0.57 [0.02; 13.70] | Dapagliflozin_  high_dosage | . | . | . |
| 0.07 [0.00; 5.57] | 0.11 [0.00; 9.95] | 0.11 [0.00; 10.51] | 0.12 [0.00; 10.92] | 0.17 [0.00; 9.32] | 0.34 [0.01; 8.26] | 0.35 [0.00; 23.88] | 0.61 [0.02; 21.45] | Inject_  semaglutide_  medium_dosage | . | . |
| 0.04 [0.00; 2.97] | 0.06 [0.00; 5.33] | 0.07 [0.00; 5.63] | 0.07 [0.00; 5.84] | 0.10 [0.00; 4.91] | 0.20 [0.01; 4.21] | 0.21 [0.00; 12.68] | 0.37 [0.01; 11.11] | 0.60 [0.01; 49.67] | Dulaglutide_  medium_dosage | . |
| 0.03 [0.00; 2.00] | 0.05 [0.00; 3.58] | 0.05 [0.00; 3.78] | 0.05 [0.00; 3.93] | 0.07 [0.00; 3.28] | 0.14 [0.01; 2.76] | 0.15 [0.00; 8.49] | 0.26 [0.01; 7.36] | 0.42 [0.01; 33.38] | 0.71 [0.01; 49.20] | Empagliflozin_  low_dosage |

Data presents as OR [95%CIs]. Pairwise (upper-right portion) and network (lower-left portion) meta-analysis results are presented as estimate effect sizes for the outcome of events of vulvar tumor. Interventions are reported in order of mean ranking of beneficially prophylactic effect on events of vulvar tumor, and outcomes are expressed as odds ratio (OR) (95% confidence intervals) (95%CIs). For the pairwise meta-analyses, OR of less than 1 indicates that the treatment specified in the row got more beneficial effect than that specified in the column. For the network meta-analysis (NMA), OR of less than 1 indicates that the treatment specified in the column got more beneficial effect than that specified in the row.

*Abbreviation: 95%CIs: 95% confidence intervals; GLP-1 agonist: glucagon-like peptide-1 agonist; NMA: network meta-analysis; OR: odds ratio; RCT: randomized controlled trial; SGLT2 inhibitor: sodium–glucose cotransporter 2 inhibitor*
